# Supplementary material for: Mechanistic insight into the role of AUXIN RESISTANCE4 in trafficking of AUXIN1 and LIKE AUX1-2
Source: Plant Physiol. 2023 Sep 30;194(1):422–33. doi: 10.1093/plphys/kiad506 (PMC10756756; doi:10.1093/plphys/kiad506)
Supplement: kiad506_Supplementary_Data [file kiad506_supplementary_data.zip › Suppemental Dataset S1/5445e4f6eaa2385e-AtAXR4/psi-blast.html]

Content-Type: text/html


```
Reference sequence (query): AtAXR4___
Identities normalised by aligned length.
Colored by: property
```

```
HSP processing: ranked
Search cycle: 3
```

```
                                                                                                         1 [        .         .         .         .         :         .         .         .         .         1         .         .         .         .         :         .         .         .         .         2         .         .         .         .         :         .         .         .         .         3         .         .         .         .         :         .         .         .         .         4         .         .         .         .         :         .         .  ] 473
     AtAXR4___                                              bits   E-value  N 100.0%   1:473               MAIITEEEEDPKTLNPPKNKPKDSDFTKSESTMKNPKPQSQNPFPFWFYFTVVVSLATIIFISLSLFSSQNDPRSWFLSLPPALRQHYSNGRTIKVQVNSNESPIEVFVAESGSIHTETVVIVHGLGLSSFAFKEMIQSLGSKGIHSVAIDLPGNGFSDKSMVVIGGDREIGFVARVKEVYGLIQEKGVFWAFDQMIETGDLPYEEIIKLQNSKRRSFKAIELGSEETARVLGQVIDTLGLAPVHLVLHDSALGLASNWVSENWQSVRSVTLIDSSISPALPLWVLNVPGIREILLAFSFGFEKLVSFRCSKEMTLSDIDAHRILLKGRNGREAVVASLNKLNHSFDIAQWGNSDGINGIPMQVIWSSEASKEWSDEGQRVAKALPKAKFVTHSGSRWPQESKSGELADYISEFVSLLPKSIRRVAEEPIPEEVQKVLEEAKAGDDHDHHHGHGHAHAGYSDAYGLGEEWTTT    
   1 UniRef50_Q9FZ33        Protein AUXIN RESPONSE 4 n=...   295     3e-78  1 100.0%   1:473     1:473     MAIITEEEEDPKTLNPPKNKPKDSDFTKSESTMKNPKPQSQNPFPFWFYFTVVVSLATIIFISLSLFSSQNDPRSWFLSLPPALRQHYSNGRTIKVQVNSNESPIEVFVAESGSIHTETVVIVHGLGLSSFAFKEMIQSLGSKGIHSVAIDLPGNGFSDKSMVVIGGDREIGFVARVKEVYGLIQEKGVFWAFDQMIETGDLPYEEIIKLQNSKRRSFKAIELGSEETARVLGQVIDTLGLAPVHLVLHDSALGLASNWVSENWQSVRSVTLIDSSISPALPLWVLNVPGIREILLAFSFGFEKLVSFRCSKEMTLSDIDAHRILLKGRNGREAVVASLNKLNHSFDIAQWGNSDGINGIPMQVIWSSEASKEWSDEGQRVAKALPKAKFVTHSGSRWPQESKSGELADYISEFVSLLPKSIRRVAEEPIPEEVQKVLEEAKAGDDHDHHHGHGHAHAGYSDAYGLGEEWTTT    
   2 UniRef50_P34913        Epoxide hydrolase 2 n=12 Ta...   264     4e-69  1  12.6%  20:417   160:544     -------------------KPEPQIYKFLLDTLK---ASPSEVVFLDDIGANlmVTILVQDTDTALKELEKVTGIQLLNTPAPLPTSCNPSD--MSHGYVTVKpvRLHFVELGS--GPAVCLCHGFPESWYSWRYQIPALAQAGYRVLAMDMKGYGESSAPPEI----EEYCMEVLCKEMVTFLDKLGLSQAVFIGHDWGGMLVWYMALFYPERVRAVASLNTPFIPANPNMSPLESIKANPVFDYQLYFQEPGVAEAELEQNLSRTFKSLFRASDESVLSMHK---------VCEAGGLFVNSPEEPSLSRMVTEEEIQFYVQQFKKSGF-RGPLNWYRNMERNWKWAC-KSLGRKILIPALMVTAEKDFVLVPQMSQHMEDWIPHlrGHIEDCGHWTQMDKPTEVNQILIKWLDS--------------------------------------------------------    
   3 UniRef50_P34914        Epoxide hydrolase 2 n=25 Ta...   257     5e-67  1  11.8%  20:417   160:543     -------------------KPEPQIYNFLLDTLK---AKPNEVVFLDDFGSNlmVTILVHNTASALRELEKVTGTQFPEAPLPVPCNPNDVS----HGYVTvpGIRLHFVEMGS--GPALCLCHGFPESWFSWRYQIPALAQAGFRVLAIDMKGYGDSSSPPEI----EEYAMELLCKEMVTFLDKLGIPQAVFIGHDWAGVMVWNMALFYPERVRAVASLNTPFMPPDPDVSPMKVIRSIPVFNYQLYFQEPGVAEAELEKNMSRTFKSFFRASDETGFIAVHKAT--------EIGGILVNTPEDPNLSKITTEEEIEFYIQQFKKTGF-RGPLNWYRNTERNWKW-SCKGLGRKILVPALMVTAEKDIVLRPEMSKNMEKWIPFlrGHIEDCGHWTQIEKPTEVNQILIKWLQT--------------------------------------------------------    
   4 UniRef50_Q6Q2C2        Epoxide hydrolase 2 n=7 Tax...   254     6e-66  1  13.8%  20:417   160:544     -------------------KPDPQIYKLMLDTLK---AEPNEVVFLDDVGTHlmATILVRDTDTALRELEKVTGVQLLQTP-ALPPTSCDPSALSHGYVLIKPGVRLHFVEMGS--GPAVCLCHGFPESWFSWRYQIPALAQAGFRVLAVDMKGYGESSAPPEI----EEYSLEVLCKDMVTFLNKLGLSQAVFIGHDWGGVLVWNMALFYPERVRAVASLNTPFMPSNPNVSPMEIIKANPVFDYQLYFQEPGVAEAELEQNLDRTFK------NFFRAHDETFLTTNRVREL---GGLFVGTPEEPSLSRLVTEEDIQFYVQQFKKSGF-RGPLNWYRNMERNWQWGC-KGSGRKILIPALMVTAENDLVLHPKMSKHMENWIPHlrGHIKDCGHWTQIDKPAELNRILIEWLET--------------------------------------------------------    
   5 UniRef50_UPI000180BDB7 PREDICTED: similar to solub...   245     3e-63  1   9.5%  19:418    92:492     ------------------RKPDTEIYKL---TCKKLGVVPTEVVFLDDIGLNLKSASQLGIRCIKVTNTNTAIKELESatAPKDKTAFPPASHPdtHSYINVTpvKLHFVEAG--EGPAIILLHGFPDFWYGWRNQIPALVQAGYRVIVPDQRGFGESSCPPRI----QDYGQKYLCDDVLKILDVLCIPQATVVGHDWGGSLAWNLALIYPDRFKAVCGINTPFFPINPKRNPMVSMmnPGSFDYQLYFQEPGVAEKEFELDVERSLTLIllEKSEDDTIKKEKSGFWSTSNVRARGGMLVGAPSVAVRSPYLTDYDMRYYVKNFKRTGF-RGPLNWYRNYELNWSWMK-RFAGRKIIIPALMVTASHDLVLKPEYSVGMEKRIplSRLHIERCSHWTMQEQPYKLNCGLIKWLDEV-------------------------------------------------------    
   6 UniRef50_B2MWN2        Soluble epoxide hydrolase-l...   241     4e-62  1  10.7%  18:418   161:549     -----------------KRKPDQSIFN---EALKKLGVKAEEAVFLDDLGPNviSTVLVKDTSAALKELQEVTGIDVFQEAKPVSVH---HERVPHSYATTRSGVKFHYVDIGS--GPPVIFCHGFPESWYEWKSQIPAVAAAGFRVIAMDMKGYGESSNPPEI----EEYTLERMCKDMAEFMDTLCIPQATFIGHDWGGFFVWNYATHYPDRVSAVGGICTPFFPANDTMNPWENINKNPGLyyQLYFNEVGPPEAEIEANVEKFVKAFMRRPLELKEIGFSVAGVRAKGGIMAGIPDDINSTL-------LTEDDVQYYVKQFKTCG-LRSMLNWYRTMEVNWKF-NHRAIGRKLYMPALMVTCAWDEVLPPSVSKFMDPFVVNlrAHIEDSGHWASLEQPKKLNKILVDWLNKV-------------------------------------------------------    
   7 UniRef50_B3S705        Putative uncharacterized pr...   240     1e-61  1   9.8%  20:427   163:558     -------------------KPDAKLY---EYVCNKMNVQPSEVIFLDDSTKN--CVGAEKLGMKTIIVKNSFQGLKDLAELLNIDFNRKVVPYCDISRFAHgeGIKTHYVECG--HGPPVIFCHGWPECWYSWRYQLAHIAELGYRAIALDQRGFGESSCPRAV----EEYTTEKIISDLLHLMDTLGLPNVTLVGHDWGGFIVWICALRYPERIRAVAGVNTPYIPIDVSASPLT-KMKKNPHTFDYQLYFQDEGVAEAEFEKDIERTFLCLFRGTSEEDKLSLTRKVDTSNVRERGGFLVGFPEKPRRSVILSEEDLKFYVDQFTRTGF-RGGINWYRNLDRNWEWNCRVAN----RKPSLMVTAKYDKVLYPALSKLMTPWVPNLTRKELECGHWTQLECPNELNQVLGDWLQDVHRSATMTPR----------------------------------------------    
   8 UniRef50_A7HW23        Alpha/beta hydrolase fold n...   236     1e-60  1  10.8%  94:417     3:320     ---------------------------------------------------------------------------------------------FPEPRYIDTNGIKMAVYEDGPKDGVPVLLSHGWPELAYSWRHQIPALAKAGFRVIAPDQRGYGNTGGPKGEENVPL-YDIEHLTGDLTGLLDALEIDKAVYCGHDWGGMVVWQSALMHPDRVAGVIGVNTPFLPRSPVDPIMAMRMMFGEDMYIVYFQKFGVAEQKVGADTKRTLNFLYRRSG---ITMQEWEKLPSSDKNLAFIKALDTPESEWRGKPLLNEEELAYYTAAFEKSGW-EGGINWYRNFTRNW--ERSAGLVQKVTAPALMISAADDVVLSPAMTEGMEQYVPDLEKHIidCGHWTQAEKPEELNRLMIDWLKR--------------------------------------------------------    
   9 UniRef50_A8UAB3        Putative uncharacterized pr...   227     8e-58  1  11.1%  93:415     2:284     --------------------------------------------------------------------------------------------GMQEHKSIETNGIKLHVVQQGPEDGQLVILLHGFPEFWYGWNSQMSELANKGFRVWAPDQRGYNLSDKPKKVS----DYRTDHLAADIAGLIKASGKEKVILVGHDWGGIVAWRVAREYPELLQKLIILNAPHELAMSNQ---------------------------LLTHPLQILKSSYIAFFQLRGLPEKLFGMSNWKVVEKALVASSRK-------GTFSEEDLQKYRTAWSQPGAMRSMINWYRALVSNYTSSDVP---SRVTVPTFLVWGAKDQFLGPELARKSLEFCDDGRGVLLgaTHWVHHEEPERVNKLILDFI----------------------------------------------------------    
  10 UniRef50_B5GUK2        Epoxide hydrolase n=3 Tax=A...   223     1e-56  1  16.0%  92:429     5:323     -------------------------------------------------------------------------------------------AEFPEPTLIQVNGVELEVFEAGREngKPIVLCHGWPEHAFSWRHQMPVLAAAGYHVIAPNQRGYGNSSRPTEVT----DYDIEHLTGDLVALLDHYGYEDATFAGHDWGAFVVWGLTLLHPDRVNKLINLSLPYQERGepWIEVMETFLGGDFYFVHFNRQPGVADAAFDANTSQFLrlYRKNVPPAPPEPGMAFINLAEAETPLGEPI-------------MSDSDLAVYVSAFETSGF-TGGINWYRNLDRNW--HQLADADPIIKQPALMIYGDQDFAIPR--FERLAEFVPNVEVVGLDCGHWIQEEMPEETNQVISEWLERIRPARAGVVRQG--------------------------------------------    
  11 UniRef50_B2IV02        Alpha/beta hydrolase fold p...   221     4e-56  1  13.5%  97:415    10:285     ------------------------------------------------------------------------------------------------HEYITTNGVKLHYVTQG--EGPLMLMLHGFPEFWYSWRHQIPEFA-QNFKVVALDLRGYNDSDKP----NEQSAYVMDEFIKDVEGVIKGLGYQKCVLVGHDWGGAIAWNFAYSHPEMLEQLIILNLPH----------------------------PAKFAQGLRTPQQLLRSNYIFLFQLPWVPELILQSSDYQAIETAFKG------TAVNKSAFTQADIDAYKNAAAKRGALTAMLNYYRNIFQQRMLNPNWGVL---EVPTLMIWGENDTALGKELTYDTAAYVRNFQIkiPDCGHWVQQEQPELVNQYMREFL----------------------------------------------------------    
  12 UniRef50_A0YH83        Epoxide hydrolase n=2 Tax=m...   221     5e-56  1  12.4%  48:418     1:363     -----------------------------------------------MLGQTTLVALLVVLTNCCMVLIVQADNQFSKKQTVAVENAPIDDVTF---RYIKTNGITMRIAEMGD-TGPLILMAHGWPESWYSWRHQIRFLAAAGYRVVAPDMRGYGKTDAPLDV----NSYDITTLAGDMIGVLDALGEEQATMVGHDWGAIVAAYSTLLYPERFSSLIIMSVPHQGRgpPIPALKARFRNDFFYMLYHNEAGGIAEAEYDRDPRALLSRLYLSPDSLRQEPTVTNPLRSAGGWIPRLGAAVN------LPDWLQQRDLDYLVAQYQASGF-RGGLNYYRNLDRNWEITATL-VDAKIEVPTLFIAGEKDGVitKDMLRamKTAIPNLhiLVPGVGHWIQQEAPDETNQAMIKFLEAL-------------------------------------------------------    
  13 UniRef50_B5X3M6        Epoxide hydrolase 2 n=4 Tax...   221     7e-56  1   9.7%  79:418   225:547     ------------------------------------------------------------------------------SYPASCNPEEVSHGYVTIKPNV-----KLHYVEMGA--GPPVMLCHGFPESWYSWRYQIPALADAGFRVVSLDMKGYGQSTAPA----NIEEYSQEQICQDLVTFMDKMGIPQVTLVGHDWGGSVVWNMAQCHPERVRAVASLNTPLFPVDPSKDPMEFMKTVPIFNYQLYFQKPGVAETEMEKDLARTFKIFFHGNGDKDNVPTI-----NTAGVCDRGGLFVGLPDEIPRSSILSETALQFYITQFKDKGF-RGPLNWYRNVVSNWKWLCSRPR-AKLLMPALMVTTGKDPVLLPVFSRGMENMIPNlrGHIEECGHWTQMERPSELNTILISWLKDT-------------------------------------------------------    
  14 UniRef50_Q8IUS5        Epoxide hydrolase 4 n=28 Ta...   218     3e-55  1   9.5%  28:417     2:356     ---------------------------ARLRDCLPRLMLTLRSLLFWSLVYCYCGLCASIHLLKLLWSLGKGPAQTFRRPAREHPPACLSDPSLGTHCYVRisGLRFHYVAAGERGKPLMLLLHGFPEFWYSWRYQLREFKSE-YRVVALDLRGYGETDAP----IHRQNYKLDCLITDIKDILDSLGYSKCVLIGHDWGGMIAWLIAICYPEMVMKLIVINFPH---------------------------PNVFTEYILRHPAQLLKSSYYYFFQIPWFPEFMFSINDFKVLKHLFTSHSTGIGRKGC--QLTTEDLEAYIYVFSQPGALSGPINHYRNIFSCLPL-----KHHMVTTPTLLLWGENDAFMEVEMAEVTKIYVKNyrLTIleASHWLQQDQPDIVNKLIWTFLKE--------------------------------------------------------    
  15 UniRef50_B3S706        Putative uncharacterized pr...   216     1e-54  1  11.0%  20:420   127:513     -------------------RPDPKFYQHALKEmeVISVESTEQYCKLATSIGMTGIVAeqPTVLKKLSELTGINFSKLSRPAACNPDSVTHCY-----FTTTSGVKIHFVEKG--NGPAVILSHGFPEFWYTWRHQIPFLARLGYRVIALDHRGYGESDQPP----NIDDYSMKLVNQDIIDLMDNLNIHQAVLIGHDWGSVVVWEAALRFPDRIKAVASLNLGYFPPHPEYDFTQLVQapQQFDYCLYFFDEGVAEVELEKDVDRTLRYIYSDTTPKVY---------LLAAIETSSRGLLAGLPDELPKCSFMSDKEFNYCVKNYKKNGFHYPLNWYRNEAINWNNIKSIERY-KIYQPALMVTAEYDPILLPELTNGMEEYVPNLTRANLLCGHWTQSECPAELNRVLSKWLPKVSA-----------------------------------------------------    
  16 UniRef50_Q9SD45        Epoxide hydrolase-like prot...   214     4e-54  1  15.9% 100:419    10:320     ---------------------------------------------------------------------------------------------------IKTNGIWLNVAEKGDEEGPLVLLLHGFPETWYSWRHQIDFLSSHGYHVVAPDLRGYGDSDSLPS----HESYTVSHLVADVIGLLDHYGTTQAFVAGHDWGAIIGWCLCLFRPDRVKGFISLSVPYFPRDPKLKPSDFFKIFGDGLYITQFQKPGRAEAAFAKH-----DCLSVMKKFLLITRTDYLVAPPDTEIIDHLE------IPSTIPDWITEEEIQVYAEKFQRSGF-TGPLNYYRSMDMNWEILAPWQD-SKIVVPTKFIAGDKdpNGTMEygEVFKIVVPNLEIVVIEGghFIQQEKSEQVSQEILSFLNKLS------------------------------------------------------    
  17 UniRef50_A8F9M3        Possible alpha/beta family ...   214     7e-54  1  12.0% 100:415     9:285     ---------------------------------------------------------------------------------------------------IQTNGVTLHTAMAGPEDGPLLILLHGFPEFWYGWKNQIMPLAEAGYRVVVPDQRGYHLSDKPEGI----ESYVLDQLRDDIVGLIKTlgNQKAIVGGHDWGGAVAWHLASTRSQYVEKLIIVNMPH---------------------------PRVMMKVLPFYPPQWKKSSYIAFFQLPNVPEVALQENHFQKLDEAIGLTAR-------PHLFTKEDVSSYKLAWTQPGAMTSMLNWYRAIKKGGFEKPIS---KRILVPVRMIWGMEDKYLSRKLAKETMKICPNGQLIFvdASHWINHEKPEVVNKLILEFL----------------------------------------------------------    
  18 UniRef50_C3Z1C7        Putative uncharacterized pr...   211     5e-53  1  13.5%  60:415     1:318     -----------------------------------------------------------MFFLRVIRSPMAVLSYRVRETPPAVLQDPALGTHEYVRLGTAK--IRLHYVAAGDRSKPLMVCLHGFPEFWYSWRHQLKAF-GKDYRVVAVDMRGFGDSDKPPAV----EEYKVDKMAGDIIDLIEALGYGSCTLVGNDGGAIIAGRVAMERPDLVTKLVAMNGPVSLS--------------------------VVRRYARSRPKQRLKFWYNFLAQLPYLPEMCLGFNDLKTLDV---VFRGKKMGCRNKDAFTDEDLEAYKYASSQPGALTGVVNFYRANGMRPPKEPTTG----IRCPSLLIWGDQDGVLDTDLAREMAESvpR-QCIIEGASHFVQQDVPDTVNQLMTDFL----------------------------------------------------------    
  19 UniRef50_C7P3M9        Alpha/beta hydrolase fold p...   210     8e-53  1  12.6%  85:417     3:301     ------------------------------------------------------------------------------------SQTPTFDEQAVTHERATVNGVGLHYVIAGPADGDLVVLLHGFPEFWYSWHNQLPSLADAGYRVVAPDLRGYNRSEKPPNVA----DYRVDELVADVAGLIEHCGRERAHVVGHDWGGLIAWHVGSDRPEVVDRLAVLNAPHPTS---------------------------YERLLRSSPTQIAKSWYVFYFQLPVLPELGFRLNDYAAVESILGETVR-------PDAFTDEDVERYKSALATPGALTAAVNYYRALGRQRARSMLtePDRSVDVPTLLLWGERDFALDTELTEGLDEWVDDLRvrIPDASHWVQFDAPERVDGALVDFFTD--------------------------------------------------------    
  20 UniRef50_O81299        AT4g02340 protein n=57 Tax=...   208     3e-52  1  13.1%  96:416     5:314     -----------------------------------------------------------------------------------------------EHTTISTNGINMHVASIGS--GPVILFVHGFPDLWYSWRHQLVSFAALGYRAIAPDLRGYGDSDAPPSR----ESYTILHIVGDLVGLLDSLGVDRVFLVGHDWGAIVAWWLCMIRPDRVNALvsVVFNPRNPSVKPVDAFRALFGDDYYICRFQEPGEIEEDFAQVDTKKLITRFFTSRNPRPPCIPKSVGFRGLPD-------------PPSLPAWLTEQDVRFYGDKFSQKGF-TGGLNYYRALNLSWELTAPWTGL-QIKVPVKFIVGDLDITYNIPGTKelKKHVPFLqvVMEGVGHFLHQEKPDEVTDHIYGFFK---------------------------------------------------------    
  21 UniRef50_O31581        AB hydrolase superfamily pr...   208     6e-52  1  14.2%  99:416     8:284     --------------------------------------------------------------------------------------------------FVNTNGITLHVAAAGREDGPLIVLLHGFPEFWYGWKNQIKPLVDAGYRVIAPDQRGYNLSDKPEGI----DSYRIDTLRDDIIGLITQFTDEKAIVIGHDWGGAVAWHLASTRPEYLEKLIAINIPHPHVMKTVTPL---------------------------YPPQWLKSSYIAYFQLPDIPEASLRENDYDTLDKAIGLSDRPAL-------FTSEDVSRYKEAWKQPGALTAMLNWYRALRKG----SLAEKPSYETVPYRMIWGMEDRFLSRKLAKETERHCPNGHLIFVdaSHWINHEKPAIVNQLILEYLK---------------------------------------------------------    
  22 UniRef50_C1YUK2        Predicted hydrolase or acyl...   207     6e-52  1  14.1%  96:421     4:323     -----------------------------------------------------------------------------------------------EHRDVEVNGIRLHVAEQGS--GPLVLLLHGFPESWYSWRHQFAPLAGAGYRVAAPDQRGYARSDRPEAV----DAYTLPHLVGDVVALVSALGEESAVVVGHDWGAPVAWATAMMRPDLVRGVAGLSVPPVPPAMMPSVSSSRAVYGDGFYQAYFQEPGVADAELAADPASTLRRLLVGASGDAPFDQPRLWIVPEGESALGSLPEPEEL-----PSWLTEEDLAAFTADYSDPDAFTGPLNWYRNIDRNQGLMS-PFQGRVIDVPALYVGGDKDLVRAMRGVPELLENLshAGVTLPGCGHWTQQERPEEVNAALLDFLAAVHGG----------------------------------------------------    
  23 UniRef50_D0NS42        Epoxide hydrolase, putative...   207     7e-52  1  11.9%  88:416     3:321     ---------------------------------------------------------------------------------------RAVWEQWPHAFMETPEGIRLYYVDVGPRDGLPVVMVHGWPDLWFGWRHQIQALSPK-YRLIVPDVRGFGQSSTPA----NVEAYGAKNISNDLMALLDELNIAKAVFIGHDWGGSHVWRTCLYHPERVIAVCGVCTPYIPPSKTYVPLEVVVEKVPQFKYQQFLatEVSSKVLDASPRRL----LTAMLRKPSEMGPRASRMSLPKMLKAVDSDADHPVFTQRSTLLSDAELNYYVEQYSISKF-ASTCQTYATGKIDFENE--RGLPKVIKHPSLFIGAAKDAVLKPEMARGMAKVIPNLEtvIEDAGHWVLWEQKEEVNAILSEWLA---------------------------------------------------------    
  24 UniRef50_A9RHZ9        Predicted protein n=1 Tax=P...   207     8e-52  1  11.2%  96:417    12:320     -----------------------------------------------------------------------------------------------RHRTVKTNGISMHIVEQG--EGPMVLLLHGFPEFWYSWRFQIPALAEAGYRVVAPDVRGYGETDAPKNPHV----YTSCHLVGDLVGLLDALEEKRVFVVGHDWGAKLAWDLCLLRPDCVKAVICLSVPFFPRSPKRSSIRGYYETVGEGFYMCRFQKPGR---AERDFARIGTTATLSKLLFP--PRNSFIAPKDKELMESIP------MPKKLPAWISDADLRYYAQTYEKSGW-TGALNVYRAIEKSWELQSPWTNVG-VKTAALFIVGDKDLVMGFPGVKsfKSFVPNleVVTLKGGHFIQQEQPARVNELIITFLHE--------------------------------------------------------    
  25 UniRef50_A9GRP4        Epoxide hydrolase n=1 Tax=S...   206     1e-51  1   9.0%  94:416     1:281     ---------------------------------------------------------------------------------------------MTEESYFNVNGVRLHVVSAGPATGKPVVLLHGFPDHWYGWRHQIGPLAERGYRVIVPDQRGYNLSEKPSGT----DSYKIARLAGDVIGILDALALDRVSLVGHDWGGAVAWWVAANHVERVERLAILNCP--------------------------------------HFSTFQRALLSFEQFKRSWYIYLFQIPHLAEALCRLEggLVKLGLAARPGTFSERELASYFEAWGRPDAMRGMLSWYRALGREVFSRFPR---TQIDCPVLLLWGARDPFLSASMVEPSMTFCKDGRSVLfdETHWLHWEAPERVNGLLLEWIE---------------------------------------------------------    
  26 UniRef50_B9HFA7        Predicted protein n=5 Tax=M...   206     1e-51  1  13.9%  97:419     8:319     ------------------------------------------------------------------------------------------------HQRIKTNGIWLHVVEKGS--GPLVLLLHGFPEFWYSWRHQITFLANHGYHAVAPDLRGYGDSDSPLSP----NSYSVLHLAGDLVGLLDYFGEQQAFVVGHDWGAVIGWHLSLFRPDRLKGLIAISVPYFPRDPVAKPIEFFRGNFGDEFYISQFQEPGR---AERAFARYDYLTVMKKFLMINKTDPLVAPSGMEIIDYLQTPAV-------LPPWITEEELQVYADKFEESGF-TGPLNYYRAMDLNWELSAPW-QGAKVTVPTKYIVGDkdTYGTReqGDTFKGLVPDLEVVILEGghFIHEERAHEVSQEILTFLQKLS------------------------------------------------------    
  27 UniRef50_Q5WK07        Putative uncharacterized pr...   206     1e-51  1  10.9%  97:416     6:287     ------------------------------------------------------------------------------------------------HHIVETGGHTFHIVSAGQEDGELVLLLHGFPEFWYGFRHQINALARAGYRVIVPDQRGYNQSDKPKDIK----AYTLDVLRDDCVAFIKAFGRKQAYLIGHDWGGAVAWHLAASKPEVVKKLVAINIPHPAEMRV---------------------------ALKKHPLQLFRSAYMLFFQVPHAPEKLLAARDFAYLEAG-------MTKTANERAFTKAELRHYKLAWRQPGALKGMLNWYRAIRFQGVDRKTEIDLP-VSVPTRIIWGANDQFLSKALAQASLKRCSNGDAVLVDGahWLHHEHPEIVNHLMLEHLQ---------------------------------------------------------    
  28 UniRef50_UPI0000E219FF PREDICTED: epoxide hydrolas...   206     1e-51  1  10.6%  20:417   160:512     -------------------KPEPQIYKFLLDTLK---ASPSEVVFLDDIGANlmVTILVQDTDTALKELEKVTGIQLLNTPAPLPTSCNPSD--MSHGYVTVK---------------------------------IPALAQAGYRVLAMDMKGYGKSSAPPEI----EEYCMEVLCKEMVTFLDKLGLSQAVFIGHDWGGMLVWYMALFYPERVRAVASLNTPFIPANPNVSPLESIKANPVFDYQLYFQEPGVAEAELEQNLSRTFKSLFRASDESVLSMHK---------VCEAGGLFVNSPEEPSLSRMVTEEEIQFYVQQFKKSGF-RGPLNWYRNMERNWKWAC-KSLGRKILIPALMVTAEKDFVLVPQMSKHMEDWIPHlrGHIEDCGHWTQMDKPTEVNQILIKWLDS--------------------------------------------------------    
  29 UniRef50_Q136P7        Alpha/beta hydrolase fold n...   206     1e-51  1  13.8%  94:418     1:314     ---------------------------------------------------------------------------------------------METTRTVAVNGIELFLREQG--EGPLVLLCHGWPELSYSWRHQIAALAEAGFRVVAPDMRGFGRSSAP----QPIEAYSIFDLVGDMVALVAELKETRAAIIGHDWGAPVAWHAALFRPDLFTAVAGLSVPPPWRGRGRPLETLRKGGVTNFYWQYFQAPGVAESELERDVALTMRGILGGGFSDPVRSLF---------IPEGGGFVGEIASDRSLPDWLSEAELALFVEAYKASGF-RGGLNWYRNIDRNWDLTAPW-QGAQIRQPSLFIAGSKDPVISDAMsaGIERVLPNIqlIIDGAGHWIQQERPNEVNAALIAFLKKV-------------------------------------------------------    
  30 UniRef50_B4X5B0        Hydrolase, alpha/beta fold ...   206     1e-51  1  14.5%  94:418     5:319     ---------------------------------------------------------------------------------------------FTRERQVPANGLDFFVAEAGEAGAPLVLCLHGFPECWASWRYQLPVLAQSGYYAVAPDLRGYGFTGGPKEV----DAYRQSLLVEDVMALIQALGYESAILMGHDWGCGLAWQVARQYPQAIKAVIGMSVPYGGppEPPTAAMRKLFEDRFFYMLYFQQPALPEQELEADVAHSLRCIFHGLSAD--------GIADFNVKPDDTGFLQSMPVPETQPRWMREEDLAYYVERFEHSGF-TGPINWYRAMDASW-QESRDDSNWQITMPTLFIGGLQDPvfGQKALQRMPDYIPDLRTVMLdcGHWIQMEQAAEVNREILAFLEEI-------------------------------------------------------    
  31 UniRef50_Q0IIS3        Epoxide hydrolase 3 n=3 Tax...   206     2e-51  1  11.9%  24:417     2:360     -----------------------QLYLSRLLLIVTRTALRVTGVFFWVLVYVAALLAAVSYIPDALRLLTRGPLSAFRWGPRKAAPACLTSSAHGQHGYIRmsGIRFHYVASGDKRNPLMLLLHGFPENWYSWRYQLDEFS-NGYRTVAIDLRGFGGSDAP----SRLEDYKMEILLQDLQDLIRGLGYSRCVLVGHDWGGTLAWTFAVRHRDMVTHLIVMNAPHPSAFHD---------------------------YVLSHPSQLFSSRYVFLFQLPLIPEILLSLRDFEHIKKPLTDATHGI--QNVECKLSKEEVEAFVYYPSQKGALTPPLNYYRNLFGFFPV-----KAQDVLVPTLLLWGEHDAFLEAAMVPEMQQYvpFRAEIIPNASHWLQQDRPQEVNKIIRDFLKE--------------------------------------------------------    
  32 UniRef50_A6H695        Abhd9 protein n=3 Tax=Theri...   205     3e-51  1  10.6%  13:418    49:415     ------------SVVVPERKDMPEFVVTALLAPSRLSLKLLRALVMSLVYLAALVAAFVYSCIALTHVMCRPRRgrQRLSPPECLRDPTLGEHCFL--TLRSSGLRLHYVSAGHGNGPLMLFLHGFPENWFSWRYQLREF-QSHFHVVAVDMRGYSPSDAPKEVDC----YTIDLLLDDIKDTILGLGYSKCILVSHDWGASLAWEFSIYYPSLVERMVVANGPPMS---------------------------VIQEYSIHHIGQIFRSNYMFLFQLPWLPEKLLSMSDFQILKDTFTHRKNGI------PGLTPSELEAFLYHFSQPGCLTGPINYYRNVFRNFPLEP-----KKLSTPTLLLWGEKDFAFQQGLVEAIGRhvPGrsHILPGSGHWIPQSHPQEMHQYMWAFLQDL-------------------------------------------------------    
  33 UniRef50_Q218G9        Alpha/beta hydrolase fold n...   205     4e-51  1  10.6%  94:414     4:325     ---------------------------------------------------------------------------------------------MPPLQFASSNGIRIGYYDAGPadTPPLVLCHGWPELAFSWRHQIKALSEAGIRVIAPDQRGFGASDRP----QPVEAYDIEQLTGDLVGLLDHLAIDKAIFVGHDWGGFVVWQMPLRHPTRVAGVVGINTPHTapPNDPVAIFRKRFGDSMYIVQFQDPAREPDRIFASRVEQTFDVFMKKPLPRKQGVVAEGLAAADGDAAKPNLAFPQMVLAYDASRdlSPQEKQVFVDTYSATGF-TGGINWYRNMTRNWQRSADLDLI--VRVPSLMIMAENDAVLPPSAADGMEKLVPDLeyLVRDCGHWTQQEKPDEVSAKLIEW-----------------------------------------------------------    
  34 UniRef50_B9H579        Predicted protein n=2 Tax=P...   204     6e-51  1  14.2%  96:418     5:319     -----------------------------------------------------------------------------------------------EHITVATNGINMHVASIGT--GPVILFLHGFPELWYSWRHQLLHLSSLGYRCVAPDLRGYGDTDAPESAS----QYTGLHVVGDLIGLLDSFGIDQVFLVGHDWGAMIAWYLCLLRPDRVRALVNLNVAFMARDPKTINPMEVLKSIYGEDYYACRFQEPGPGEIEKDFAQVA---------TAKMLRIFFSSFGPKPLIVPKETGFRGIPDPPCLpgFSEEDIDFYANKFNQKGF-TGALNYYRAINQTWDLMAPWIGV-KIQVPVKFIIGDQDINYHLPGLKefKKDVPRLEEVVVMGghFPNQARPEEVSEHIYSFFKKL-------------------------------------------------------    
  35 UniRef50_A9EWE7        Putative uncharacterized pr...   204     8e-51  1  13.4%  97:416    10:290     ------------------------------------------------------------------------------------------------HRFTEHGGVRLHYVEAG--DGPLVVLLHGFPEIWYSWRHQIPALVEAGYRVIAPDMRGYNLSDKPEGVA----AYAIAELTADVAALIQASGAEraAVVGHDWGGGVAWGFAARYPALLERLVVLNCPHPERLVAGFRTAR----------------------------QLRKSWYMFFFQLPKLPELAAQKDDFAWLRAAVKDDPRR------PGAVSDEDLAHYVKAWSEPGALTAMINYYRALFRPGSLGELG-RQPRIDVPVQVIWGEHDRYLGSELAAPDPALVPNARvrIPDASHFVHYDRPEKVNQLLLEFLR---------------------------------------------------------    
  36 UniRef50_B3QKG9        Alpha/beta hydrolase fold n...   203     1e-50  1  14.5%  94:417     1:313     ---------------------------------------------------------------------------------------------MASTRTITANGIELFLREQG--EGPLVVLCHGWPELSYSWRHQIGALADAGYHVVAPDMRGFGRSSAPQAV----EAYSIFDLVGDMVALVAELGETRAAIIGHDWGAPVAWHAAQFRPDLFAAVAGLSVPPPWRGKGPPLDQLRAAGITNFYWQYFQKPGVAETEFERDVASTMRGILCGGFADPGRSLF---------VPEGRGFIGRSAASLPLPPWLSEAELAFFIEQYKQSGF-RGGLNWYRNIDRNWELTSPWQGAP-IHQPAAFIAGSNDPVISDKMsaAINRVLPNlkLIIDGAGHWIQQEKPAEVNAALIEFLKE--------------------------------------------------------    
  37 UniRef50_C7PVF9        Alpha/beta hydrolase fold p...   201     4e-50  1  14.4%  98:431     4:318     -------------------------------------------------------------------------------------------------RSVMVGGLRMNVVEAGSGPD-VVVLLHGFLNYSYSWRHQVRDLAAAGYRVLAPDLRGYGETGCPEDV----EQYSMLHLVADVVGLLDAFGIERATVVGHDWGSVLAWHTALLRPDRVRGVVGLSVPYVPRADISLLTAMRERFGDLYYMQYFQEVGPADRELARDVAATFRSVLSSGSEP------------WNPVLPEGGGWLDCLPDKGIPDWLTAADLDAHVAAFERSGF-TGALNWYRNIDRNHELTAAW-QGAKIQVPALFLYGTRDAFGRyeLIDDLPRSVPDLrvVRLDAGHWVQQERPDEVTAALVDFLER---DRDRDGARPMP------------------------------------------    
  38 UniRef50_Q0VSG4        Hydrolase, alpha/beta fold ...   201     5e-50  1  13.8%  94:420     3:319     ---------------------------------------------------------------------------------------------FTRERQVSANGLDFFVAEAGVAGLPLVLCLHGFPECWASWRYQLPVLAQSGYYAVAPDLRGYGFTDAPKDV----EAYRQSKLVEDVMALIRVLGYDSAILIGHDWGCALAWQVARCYPKSIKAVIGMSVPYGGPapPTAAMRKLFEDRFFYMLYFQQTGLPEQELEADVSHSLRCIFHALSAD---------GIADFNVKPDDTGFLQSMPVPEAQPRWMREEDLAYYVERFEHSGF-TGAINWYRSMDASWEESRNASNW-QIPMPILFIGGLQDPvfSQKALQRMPDYMPDLRTVMLdcGHWIQMEQASEVNREILAFLEEVDG-----------------------------------------------------    
  39 UniRef50_Q2JPE5        Hydrolase, alpha/beta fold ...   200     1e-49  1  13.5%  82:415     7:301     ---------------------------------------------------------------------------------PRLPPEVAAKMGTWQHGYALTNGIQLHYVTQG--EGELAILLHGFPEFWYSWRHQIPVLAQR-FRVVAPDMRGYNDSDKP------DHGYDLDTLTEDIRGLLSHFGARRAVVVAHDWGGAIAWHWAQFFPEEIRKLAVLNSPH---------------------------PACFRREFLSNLDQLRRSWYLFFFQLPWLPEWVLQWDLGDWVRRIFQE------TSVRKSAFTRHDLKLYQEALSKPKVLTSALNYYRQLFNLPTLQNllQPMRQILAPTLLIWGEEDFALSRELTEGMDPFFPNgkEYIPECGHWAQQEAPQTVNRLLMEFL----------------------------------------------------------    
  40 UniRef50_Q9S7P1        Os01g0255000 protein n=6 Ta...   199     1e-49  1  13.5%  90:416     2:320     -----------------------------------------------------------------------------------------AAAEVRHRTVEVASGVRLHVAEAGPEDGPAVLLVHGFPELWYSWRHQMRALAARGFRAVAPDLRGYGDSDAPPGR----DSYTVLHLVGDLVALIADVGQPRVFVAAHDWGAAVAWQLCLLRPDLVTAFVALSVEYHppTRSPVQTLRAVCGDGHYICFFQKPGVAEAEFGRGDIKCLLKKFYGMRKAAPLI------------IPPGKTLFDSIDSDGTCPAWLSEEDISYYAEKFEKTGF-TGGLNYYRCIDLNWELTAPWTGVP-IKVPTKFIVGDQDLTYNIPGVKdlKACVPNLevIMEGVAHFINQEKPDEVSDHICGFFS---------------------------------------------------------    
  41 UniRef50_UPI00005870D7 PREDICTED: hypothetical pro...   199     2e-49  1  13.9%  45:417    14:335     --------------------------------------------YLPVYILGIPIVLLMVLKGLFY----NGPRWLFYEKERRERPACMQDPQLGTHKFVQVKNLKLHVVESGDAKNPLMLFLHGFPECWYSWRHQIRAF-NKDYHCVAFDMRGVGESDGPPG----KRNYTSDLITGDVCELIQVLGHETCILVGHDWGGLIGWKFAAQYPQMVERYIAMNIPH---------------------------PDRFSELLTSHLPQIIMSWYIFFFQIPWFPER-----------AVKMGDYNMIDEECKHGETTDEDIQAFKYSISKPGRTHTFLNYYRN-----ELTLLFNKTGVVKTPTLLIWGTADNYLHTKLSYDTEKFCPSLKVERIEGghFIQQDKPDLVNQLMRKYLER--------------------------------------------------------    
  42 UniRef50_Q0CB76        Predicted protein n=1 Tax=A...   199     2e-49  1  11.0%  98:416    39:350     -------------------------------------------------------------------------------------------------HTTQINGKTYGYLLAEPEGGytILLIHGFPNLSISWRRQIPTLLSMGFRVICPDCIGYGASDAPKDTLAP---YSMKSHADDFKELIRSLSCEKVIVGGFDWGALLAYRLALWHPEVVSHLFTLCVPHVGIPTEWVDPETRVKKDPSLAYTLQFISG----------DVEKHSQTSDGVKHVLNALYGGQLPNGDFGTSLEGLKFDLIPHLERSILLDKGELEFYVDQFSKSG-LAGPLNFYRCGQQNHEDDQvrGQDEKIHCPTMFIWPSNDLIITREMAESMAKFVPDLTFKEigASHFAMWERPAEVNKILRDWLA---------------------------------------------------------    
  43 UniRef50_Q8DKE1        Tll0918 protein n=13 Tax=Cy...   198     4e-49  1  12.4%  92:421    10:293     -------------------------------------------------------------------------------------------EGPWIHKFIVSNGIRLHYVTQG--EGELVLLLHGFPEFWYSWRHQIPVLAQK-HKVVALDLRGYHLSDKPQDTAS----YVLDELILDIVGVIDGLGYRRCHLVGHDWGGMVAWGVAYAVPERMQTLSVLACPH------------------------------PAKFQQLNFEQWLRSSYMLLFQLPWLPEILLEWGGYGAIAQIFRWA------AVNQQAIRPLDIARYQDAAAQRGALSGMLNYYRAGLQSLYSREW----GVLDVPTLMLWGRQDPTLGIELTYGTEAYVKELKIQYLdcGHFVQQEQPDLVNEYLLEWLETVSAP----------------------------------------------------    
  44 UniRef50_C3KBE5        Putative epoxide hydrolase ...   198     4e-49  1  11.0%  94:421    13:326     ---------------------------------------------------------------------------------------------PMSEQLICVNGIELSVHIAGPEDGRPIWLLHGFPECWHSWREQIPALAAAGYRVFAPEMRGYGQSGSPAEIA----DYDLLTLCGDIQQAMDHFGHAQVVMVGHDWGAVVAWHLALLEPERITRLITLSVPFAGRARRpiQIMRELYADRFNYILYFQEPGVAEQELNADIERTLRLFMQD------QDVFLQSKPANARLLEGVPA------PGALPHWCSQADLDVYVHTFAEHGF-RGPLNWYRNFERNWQLTESLA-GKQVLQPTLFLIGDRDpvFEAHTLKRMPESVPHLehVLLNCGHWIQNEQGQQVNALMLGFLGKAGAA----------------------------------------------------    
  45 UniRef50_A3Q6Z4        Alpha/beta hydrolase fold n...   198     6e-49  1  13.8%  94:419    35:339     ---------------------------------------------------------------------------------------------TPTERLVGTNGVRLRVVEAGERGAPVVVLAHGFPELAYSWRHQIPVLAEAGYHVIAPDQRGYGESSRPEAVT----DYDIVALTGDLAGLLDDVGAQRAVVVGHDWGSPVVTNFALLYPDRVAGMVNLSVPPVPRadPPTQIWRKTFGDHFFYILYFQEPGVADADLGRDPRQ------------------SLQRMLALEGFSAPAPDLADNPLPPLPEWMSQEEFDQYADVFTRTGF-TGGLNWYRNFDRNWELTATTPAATI-TVPTLFIAGSADPVLSFTPRHRVTDLVTgrEVLLDGAGHWLQQERPDEVNALLLEHLAGLE------------------------------------------------------    
  46 UniRef50_A9RVE8        Predicted protein n=1 Tax=P...   196     1e-48  1  29.9%  27:467    61:509     --------------------------RKAQESAQRVVVRNENPFFFWLWFMGIMSTIMLVFVVGTSI-LNSNVKIEILSMPTGVREHFEAGKQVKVEVGRDRSPVKMFVRASVDPHaeTVLLLHGLGSSSFLYRQLLPLLAAKQLAAVAVDLPGAGLSEKSYVASEATDNLSEDDhlQIRLEALKELLVEGFFPKSSDESSEHEFLRKLKGSTNLKGKKLLALGSDELGHALDQIVASLEVAPVHLVMHDTGVEVGLAWASQNPSKVASLTLVDSVPrpSLPFWLFTIPLVGPVIAHSDLAFRWVLRSCCLHYVTAETAKDYIYLLRSSNGLRALMKSWTASNSSMDVAEWAGNSNLEDVPKQILWASGWSKEWQKEGEQLAKNLPGAAFRSHHGSRWPQEDAAPEIAEMIAELVKSLPPSAAQPEMGELLEDVARKNGNPAPHQSYSSQggHNHGHLQHSHDHDLG------    
  47 UniRef50_A9AZ89        Alpha/beta hydrolase fold n...   196     1e-48  1  12.7%  96:417     4:286     -----------------------------------------------------------------------------------------------EHATALINGLNLHYVRAGS--GPLVVLLHGFPEFWYSWRHQIPALAE-THTVVALDQRGYNISDKPA----LWQHYTIDLLIDDVRALIEHLGFERATIVGHDWGAAVAWMFAMRYHGYLERLVIMNVPH---------------------------PLLMAKGLLTNPRQMLRSWYMFAFQIPHLPEWSAKRNNFQVLTKALTGAVQR------PGVFSAEDIQVYHQAWGRPGALRAMINWYRAYIRQGSRYLRQLSTHHIHVPTLLIWGTADTALGQELTYGTERYVTDLRIRYldVSHWVQQEAPNEVNAVLIPFVRG--------------------------------------------------------    
  48 UniRef50_B8FAJ5        Alpha/beta hydrolase fold p...   196     1e-48  1  13.6%  50:417     5:313     -------------------------------------------------IKALIKTAIVFVCAAAFFGCGLTTD----KIPPGVIKDATYHDY-----YLEVDGVSYHYEEY-KGDGPVVFLVHGFGSSTYTWRDVIPPLSNQGFHVIALDMKGFGWSDKPLG-----DDYTPYNLMEEVNAFMEAKGLSQVVYAGNSLGGFVGAMLTIEHPDKVKKLILVDAAGEPMPDRPTVIK----------------------------------------------MARWVHAAEAMKLTAGSWIINWNLTSAVYDkvTKERVQAYYERMQTVGAVDAMVSLAQNTDFN-SLYSFVGCLSFIEQPTLIIWGEEDTWIPVACAYKYNKDIPGSIliIPKCGHIPQEEKPEVTAKYIGDFARD--------------------------------------------------------    
  49 UniRef50_Q0AKN4        Alpha/beta hydrolase fold n...   196     2e-48  1  13.6%  94:425     4:323     ---------------------------------------------------------------------------------------------FPAPQRIATNGIELSVHLAGPEAGQPLLLVHGWPELAYSWKNQISVLAEAGYRVIAPDLRGFGGSDCPDGI----DAYAIDALIADLTGLLDALGHEKAVWVGHDWGGIITWHAAMLAADRFDGVIGVNTPHLPRGAQPPTEAFREIGGEDHYILRFQEPGFAEGVFEGKEDDFFAFIFGAPPKVGKLEDYYPEITHIPAQFAAFDGRAEKRIV------VRPEDRAIYADAYRKTGFGPG-INLYRNFDANW--QRMGGVDHRIALPCLMVSAELDFMLPPKLAGWMPALCKDVEFAVLdcGHWTMWEQPDALNGYMLEWLERRFPASGRA------------------------------------------------    
  50 UniRef50_B6HJJ1        Pc21g19510 protein n=4 Tax=...   196     2e-48  1  11.6%  83:426     2:351     ----------------------------------------------------------------------------------PADKIRVSGDSRIEYKTATLNGHHYSYILSQPKSGqtVFLIHGFPDLSMGWRYQIPMLVDMGFRVVAPDCLGYGRTDAPDDFTP----YSHKSCAADIKSLATHLGETQIILGGHDWGAALAYRIALWHPELVSHLFTVCVPYARPMAQNISIEDLVRSVTPHFAYQLQFKSGEVEKVIRSKDEVRQFLLALYGGRTEAGEFGF--------DANKGVLLDKIGQLKPSRLLSEEELEFYTNEFTRSGIHgsLVNWYRTRDVNYE-DELAILGREIQVPTLFIQGLRDQALPPHLGKSMAKQVPRLTLKQVNTSHWALWEKPEEVNGIIGTWLKDQSSADGRAG-----------------------------------------------    
  51 UniRef50_A9CIL2        Epoxide hydrolase n=5 Tax=B...   195     3e-48  1  11.8% 114:418    46:351     -----------------------------------------------------------------------------------------------------------------AGQGPLVLLCHGFPETKYAWRHQIEAFARAGYRVVAPDMRGYGKTEAPERP----DQYTVFHTVGDLVALLDALGEQQAVVVGHDWGATVAWQAALMRPDRFRAVVALSVPMMGLPPMPPSRIFPQDDKSLFYTLYFQDPDGAEAEFGRDVALTLRKLIFAASGEAGPRLPGDSTPNPFGMVSRSMGLLESLPEPAapDWLPAPDFDRMVRDFQASGF-RGGLNYYRNLDRNWELQRLAAGL-KITVPALFMIGERDTGLSIPGmaEMPTLVPDLrhTISGAGHWLQQERPKEVSTAILRFLESI-------------------------------------------------------    
  52 UniRef50_Q0IQI9        Os12g0118400 protein (Fragm...   194     6e-48  1  15.9%  11:418    96:540     ----------PRSLQYPPSTPHPLHSTAFSHSLRPRRPNGPPPAFASAEFPGSVPDSAQMPPRRRRRRSVAGIDQDDLLDPDALAD--------PDSSFYEINGVRVHHKVCTHEdgLPIVLLHGFGSSVFSWTHIMRPLAraGAKVLAFDRPAFGLTswSGDDTKPINPYSMAFSVMATLAFIDQLGAKKAVLVGHSAGCLVAVEAYFEAPERVAALVLVAPAIFVPVFRRKGVKEnnRIWGKFLELCLWIAGFLMNMIRAIGGVVRSLYYKSVVAVLRSSVGVMLVRLIMDKFGILAVRNAWY----DPSKVTDHVIQGYTKPLRSRGWEMALLEYTISMIMDSISspVSERLSEISCPVLVVSGDTDRLVPRWNTERVARAIPGAGFEVinSGHLPQEERPEEFVSVVERFLRRA-------------------------------------------------------    
  53 UniRef50_C6XBB8        Alpha/beta hydrolase fold p...   194     7e-48  1  12.1%  46:417     1:326     ---------------------------------------------MLRYLRIAKLTIALGLVATATAIPLS------LQAAPTVSSQGSAATKAVSYHTVDIDGVKVFYREAGPANAPVVLLLHGFPSSSHMFRNLIPQLAER-YHVIAPDYPGYGQSDQPA---MDKFTYSFDNLAMVIDKLTIRLGLGKFAIYVQDYGAPVGYRIASAHPEKISAIVVQNGNAYEEGIDNQFWVPVKDYWKNKSEANAAKLRPILELAATKWQYTEGFRNPAAN-------------------------------------VSPDAWMMDQAYMDRPGNKDIQLELFYSYGTNPPhaEWQAYFRRYQPPMLIVWGKNDKIFPAAGATPYLRDLPNAELHLLDTGHFALEEDGDEIARLMGNFLDR--------------------------------------------------------    
  54 UniRef50_Q1GL29        Alpha/beta hydrolase fold n...   194     8e-48  1  13.4%  94:421     7:314     ---------------------------------------------------------------------------------------------FPAPQRIKLNGITLEVFEAGSHngNPIVLCHGWPELAYSWRAQIPALVAAGYHVLAPNQRGFGASSRPADVT----DYDITRLTGDLAALLEHFGYEAATFVGHDWGANVVWSMALLHPERVVRLINLALPYQTrpTPWIEFIEALFGADHYFVHFNRQFGVADAILDQNADQFLrlFRKNLTMVPSEPGMMMINLARAETPLGDPL-------------ISAEDLEVFVSAFKASGFTPG-INWYRNMDRNW--HILAEIDPVIRHPALMIYGLQDPIPP---SENLSEFVPNVAIRSLDCGHWIQQERPEETTQVMLEWLKAQDHA----------------------------------------------------    
  55 UniRef50_Q9H6B9        Epoxide hydrolase 3 n=9 Tax...   193     1e-47  1  11.6%  22:418     1:358     ---------------------MPELVVTALLAPSRLSLKLLRA-FMWSLVFSVALVAAAVYGCIALTHVLCRPRRGCCGRRRSASPACLSDPSLGEHGFLNlsGLRLHYVSAGRGNGPLMLFLHGFPENWFSWRYQLREFQSR-FHVVAVDLRGYGPSDAPRDVDC----YTIDLLLVDIKDVILGLGYSKCILVAHDWGALLAWHFSIYYPSLVERMVVVSGAPMS---------------------------VYQDYSLHHISQFFRSHYMFLFQLPWLPEKLLSMSDFQILKTTLTH------RKTGIPCLTPSELEAFLYNFSQPGGLTGPLNYYRNLFRNFPLEP-----QELTTPTLLLWGEKDTYLELGLVEAisRFVPglEAHILPggHWIPQSNPQEMHQYMWAFLQDL-------------------------------------------------------    
  56 UniRef50_Q0BZI5        Putative epoxide hydrolase ...   192     2e-47  1  14.6%  96:416     7:316     -----------------------------------------------------------------------------------------------TQRRVATNGIELNIAEAG--EGPLVLLLHGFPESWYSWRHQFAPLAAAGYHVVAPDMRGYGKSDKPPEIT----DYVQTEVIKDVIGLIPALGYDNAVVIGHDWGAPTAWSTALFHPDKVRAVGGLSVPFMPRSpqPMPMLREIYKGQFFYQLYFQEPGVAEAEFEKDMHTALRKFLIMAAGETD-------LTTLAPKTEDDDLLTSLPYPETLPKWLTAADLDFYVSEFTASG-MRGPINYYRNHDLHWQLTEGAPM--EIHQPAMFIAGTADGVVMMAAAAIEahFVKDLRimIPGIGHWTQQEAPEAVNETILEFLR---------------------------------------------------------    
  57 UniRef50_Q3M1E7        Zinc-containing alcohol deh...   192     2e-47  1  13.2%  99:431     7:297     --------------------------------------------------------------------------------------------------FRQVGDVEVFYREAGSSNAPVILLLHGFPTASHMFRNLIPLLADR-FRLVAPDLPGFGQTKAPPRGM---FDYTFDHLADVIEGFVDALSLDQYVLYIFDYGAPIGLRLAMRHPERISAIISQNGNAY-----LEGFSDEWGSWETYWREPSAANREACRSSL-TPDTIRNWQY---------------------------------GIGADPNLLSPDGYELDIAYMGRAGAEEIQLDLILDYRSNVAlpAFQSYFREYRPPLLAVWGRHDPAFLPAGAAAYQRDLPDAKIHLLDAGHFALETHAEEVATLIRAFLDSTIGSTLRLERENLP------------------------------------------    
  58 UniRef50_C3Y9Y3        Putative uncharacterized pr...   191     4e-47  1  10.3%  70:417     7:327     ---------------------------------------------------------------------RRGPRAFFGYKVRQDRPKCLDDPELGTHGYVRlsGLRFHYVAAGERGKPLMLCLHGFPECWYSWRHQLKEF-RNSYRVVAPDLRGYA--TYYVDIATENGGFSLahLTNDVQELIEALGYSSCTLVAHDWGGIIAWLFAVDHPDLVDKLIVMNAPH---------------------------PGTFMKYMRTHFSQFRRSWYVFFYQLPWLPENMVKAFDFQLI---YGAFKGKGMGCKRRDAVTEEDLEAFKYTFARPgnISAAINYYRAM---LKQPPRSYKGTKVTCPTLMIWGDQDAALEVGLTEGTDKFVPDFTLkvPGASHWVQQDQPEVVNGIMRDWLKA--------------------------------------------------------    
  59 UniRef50_Q2QYI1        Hydrolase, alpha/beta fold ...   191     4e-47  1  15.9%  11:418     6:450     ----------PRSLQYPPSTPHPLHSTAFSHSLRPRRPNGPPPAFASAEFPGSVPDSAQMPPRRRRRRSVAGIDQDDLLDPDALAD--------PDSSFYEINGVRVHHKVCTHEdgLPIVLLHGFGSSVFSWTHIMRPLAraGAKVLAFDRPAFGLTswSGDDTKPINPYSMAFSVMATLAFIDQLGAKKAVLVGHSAGCLVAVEAYFEAPERVAALVLVAPAIFVPVFRRKGVKEnnRIWGKFLELCLWIAGFLMNMIRAIGGVVRSLYYKSVVAVLRSSVGVMLVRLIMDKFGILAVRNAWY----DPSKVTDHVIQGYTKPLRSRGWEMALLEYTISMIMDSISspVSERLSEISCPVLVVSGDTDRLVPRWNTERVARAIPGAGFEVinSGHLPQEERPEEFVSVVERFLRRA-------------------------------------------------------    
  60 UniRef50_Q21277        Protein K07C5.5, confirmed ...   191     5e-47  1  10.7%  97:414    56:336     ------------------------------------------------------------------------------------------------HKFVQLKNIRMHYVEEGPADGDVLLMVHGFPEFWYSWRFQLEHF-KHTHRCIAIDMRGYNTTDRPSGIS----DYNLTHLVEDIRQFIEILELKRVTLAAHDWGAIVCWRVAMLHSNLIDRLVICNVPHPFAFFEVYNMSKEQRNKSWYIYLFQ------------------------SQYIPEIAMRSNKMKMLEAMFRGSKAGIR------NSENFTDEDMLAWKHVFSQPGGTTGPLNYYRDLFNAPAIPR---KLQIVQPKVLILWGDEDAFLDKKGAELSVQFCRDCRVQMigASHWVQQDQPQLVNVYMEQF-----------------------------------------------------------    
  61 UniRef50_C1B7J7        Epoxide hydrolase n=6 Tax=C...   191     6e-47  1  10.3%  96:415     5:318     -----------------------------------------------------------------------------------------------TERSVTVDGFRWQISELG--DGPPVILCHGFPGLGYSFRHQMRALAASGYRAIAPDMPGYGGTDVPRDI----DDYTNERVSDALIGLLDALELDRAVFVGHDFGAPVAWTVALRHRERVSRLVLLAVPYMPDRFppSELYASLARKHFLHIHYFQEPGVADRELDGDPRGFLQ-RLFFALSGEYRYLDIWQHPSEGNGYLDVLPA----APPLPWTWLTEDEFEHYVEVFTRTGF-TGGLNWYRAYDANWE-RSARVGVAQIEVPTLFVAGANDPVVAMSGAqrMRDTVPDLRGVHlgAGHFVQLERADEVNELLLSFV----------------------------------------------------------    
  62 UniRef50_A1C823        Epoxide hydrolase n=5 Tax=T...   190     8e-47  1   9.7%  88:418     7:326     ---------------------------------------------------------------------------------------DVLGDPRIEYRSADVNGKNYGYLYSEPESGttIVLIHGFPDLSLGWRYQIPMLIDMGLRVIAPDCLGYGRTDAPEDIAL----YSHKQCADDIKELALQLGASKILVGGHDWGAALAYRVALWHPDLVTHLFTVCVPYAPPAKKYYPLEEFAATIAPHFTYQLQFKSGELEKAIQSEEDIKKFLCALYGGRTDTGEVAF--------DAGLGVLLENMFRVRPSRLLSEEELEYYAREFSRNG-LRGPLNWYRTREINYE-EELAILHRRITAPVLFIQALKDAALPAHLGSGMTKTIPHLTFQQVDTSHWALWERPKEVNEMIAWWLEEV-------------------------------------------------------    
  63 UniRef50_A4GI13        Epoxide hydrolase n=2 Tax=e...   190     1e-46  1  14.0%  99:419     8:328     --------------------------------------------------------------------------------------------------FIESNGINLRLAMMG--EGPLVIFCHGWPESWYSYRHQLPAVAAAGFKAVAYDVRGYGESDKP----YEIEAYTMRNMTNDVIGIIDALGYETAITIGHDWGGPIALHTAALNEDRITATGTMSVPFTGRGPMPadLWKEVYKDNFFYQLYFQKEGIAEAEFESDLPKSLFVTYTNSDGRGMKNNLEKGQSGLVPKKDKHSTFLEGMEVFDdpSWFTQTDLDYFVSQFEMSG-LRGPFNRYRA--QNIDWNELSDLDKKLSQPAFFVTGTLDPvsDEPLLDRIKANYENLLfeLEGIGHWTQQEAPEEVNKLILKFLKEVS------------------------------------------------------    
  64 UniRef50_Q850X4        Os03g0829200 protein n=13 T...   189     1e-46  1  12.5%  96:416    12:331     -----------------------------------------------------------------------------------------------RHRTVEANGISIHVAEAGGEGGDgvLFLHGFPELWYSWRHQMEHLAGRGFRCLAPDLRGYGDTDAPPEI----ESYSAFHVVGDLVALLDALGLAKVFVVGHDWGAIIAWYMCLFRPDRVTALVNTSVAFMRHVFIRSGADAIKTTDHFHKAY-----GPTYYICRFQEPGVAEEEFAPAHARHIIRRTLCNRFTVHKAGKPESEESPPPPPLPLPAWLTEEDIDYFAAAFERTGF-TGGINYYRNMDRNWEMAAPWAD-AKVQVPTKFIVGDGDLTYHYAGIQdlKAEVPLLevVIPGAGHFIQQERAEEVSDLIYNFIT---------------------------------------------------------    
  65 UniRef50_C3V8M7        Epoxide hydrolase n=1 Tax=u...   189     1e-46  1  12.6%  84:417     1:342     -----------------------------------------------------------------------------------MRNNYESPMFSITEHVAKHDGHATFYLACGAADAPPIIFVHGWPDLSIGWRHQLPAFAALGFRAIAPDMRGYGRST----VYMRHEDYAVQHAVSDMLALLQSLGRDKALWVGHDWGSPVVWSLAGQHPERCVGIASLCVPYLPEGFAPQTLIPLVDRTVYPQDQLFAGQWDYQLLHEENFDRARsvRALFRHGDPAGVGKPAPSAFTRKTGDFFGGAGRAPDVPLDTSVLTDVDCCAYVAALERTGFF-GPDSWYRNAASNMEYAKQARDGGRLSLPALFLHATYDFIcnSHFVDPMRKACSNLTEVHVKSGHWMAQEQPVAVNRALARFIGE--------------------------------------------------------    
  66 UniRef50_B9LP96        Alpha/beta hydrolase fold p...   189     2e-46  1  14.1%  77:424    22:328     ----------------------------------------------------------------------------VEALDETVPPDEIPEEVPGKSRVVDVGDVRLHVVEAGPEDGKLLVLLHGFPEFWYGWHETIVSLANAGYRVVVPDQRGYNLSEKPSAVS----DYRIDALARDVVGLIDAYDRETAAVAGHDWGAAVGWWLALHHADRVSEFVAVNVPH---------------------------PTVFERALRTSWDQRLKSWYVLAFQLPKLPEAVASAGNWR-------LAVRGLRESSDPGTFSGEDFRRYRRAWNREGAFEAMVNWYRAIVRDRPTPATE----TVEVPTLVIWGAKDRFLSTRLAGESVDRCADGRLLILdaTHWVVHEEPHRVAKAIADHADPLPPGARE-------------------------------------------------    
  67 UniRef50_A7NLX2        Alpha/beta hydrolase fold n...   189     2e-46  1  13.0%  96:417     6:287     -----------------------------------------------------------------------------------------------EHHYLNANGIRFHVVRAGNGD-RLLLLLHGFPEFWWSWRHQIEVFA-AHYTVVAPDLRGYNETEKPA------RGYELHVLVQDVVELIQTLGFQRAYVAGHDWGGMIAWSLAIARPERVERLIALNMPH---------------------------PARFYEELQRNPEQRRRSRYILFFQIPWLPEAILSANHGAAFDRIFRS------TPIDRAVFDNETIRRYKQAMARPGALTAALNYYRAIGRHGAGDLFRGTGMRVRAPTLLIWGEQDVAFAPEVVRETQRFVPDLRIclPHASHWVQQVAPDEVNAAMKAFLEA--------------------------------------------------------    
  68 UniRef50_UPI000175F46E PREDICTED: similar to epoxi...   189     3e-46  1  13.4%  43:415    23:376     ------------------------------------------VYYIVVYGtgLTACLILIRIAWTGLKDPYGTFQwvRKKPPACLQDPELGDHAFLKG--RSSGLRFHYVTKGDHKKPLMLFLHGFPENWYSWRHQLLEFSGD-FHTVALDLRGCGASDAP----VRLEDYLLEALLYDIRDTVDQLGHTSCILVGHDWGGMLAWHFALERPDMVQLLIVMNAPHPASWLDAVLRRPSQLLRSGHACFFQLPL---------LPEFVLSLEDFKSDGCTPSLTGVIGLPVAK--LVRSLFCGKNVGIRNRSRRLTESQLEGYLYPLSQPGGLTAPLNYFRSLLSNTLY-----KHQDVAVPCMLIWGEADNILVEGMSGGTRPYVRGPvhTIPECSHWVQQDQPEIVNKLIWDFV----------------------------------------------------------    
  69 UniRef50_A6F4D1        Alpha/beta hydrolase fold p...   188     3e-46  1  11.5% 106:418    15:290     ---------------------------------------------------------------------------------------------------------RFAWREGGAPGGKALVMIHGWPESSYCWEHVAAYL-KAGFRIIAPDLRGLGDSERSPDI----EHYRKQEMAQDVISLLDQLGIDEFQLVGHDWGGIVAQEVALAIPDRVQRLVIMNIAIINNLKGNQEVIEKVR---------------------------------AGSGATYWYQHFMQTPGLPEAmgAEETWLGHFLRTWNREPFPRDAFDEYVRTFRIPGTPGTSSNYYRTFHEDAKRWATTA-GHTWLMPALYIYGNKDKVIIPEYLNHIEECFADLRVEEVEAGHFLQEEQPEAVAGYLNDFLDTA-------------------------------------------------------    
  70 UniRef50_B8FEZ4        Alpha/beta hydrolase fold p...   187     9e-46  1  14.3%  98:419     7:279     -------------------------------------------------------------------------------------------------RFVRVNGVRIHYTEL-PGPGEDVLLIHGFASSSYTWQEMAPLLHKQGYNVWALDLKGFGYSEKPKSGK-----YDPFSLMEDVVDWMDAVGLEKAVIVGNSLGGGIASLMSLVYPEKVSKLVLINALAPYDIPHPLIIRLSHFPLAPRLAGLVVTREVVRYYLKQ--------------------------------------------VFYNPRFVTPEKVQAYYEPLRSPGCLYAQTLAARAMDPKPFLRFMGDGY-SVKAPVLVIWGEDDRWIPLHYGQQLLEQnsGTFVVLPECGHMPQEEKPVDTAKAILDFMKDVP------------------------------------------------------    
  71 UniRef50_B8IW29        Alpha/beta hydrolase fold p...   187     1e-45  1  12.8%  73:431     8:323     ------------------------------------------------------------------------AGLAAASIASTSPQAVPSVSVTTTYHRTEVDGVSIFYREAGPKDAPTIVLLHGYPSSSRMYDPLLPLLADR-YHLIAPDYPGFGHSDAPP---PSQYCYTFDHLAETTHNLLTKLGVGQYVLFMQDYGGPVGFRIALAHPEQVRALIVQNANAYAEG----------------LGVKWKGIADYWRDPAAHPGQLDAFTSLEGAKQ------------------------RHLGNSPNVERYSPDTWMDEYAMLSRPGSREIQGALFYDYRTNVayPAWQAWMREHKPPTLVIWGRYDPSFIVPGAEAYKRDLPDAEIRILDAGHFALDEQADEVARLTRDFLEHHLSPRDPTGMQRAR------------------------------------------    
  72 UniRef50_A7RFZ6        Predicted protein n=1 Tax=N...   186     1e-45  1   9.8%  19:418   156:540     ------------------RKPDPEMYKL---VCKKFKVQPNEVLFLDDLPSNlmATILVKDPISALRTVKEITGIDVLKAYPTPACTPSSVPHSIVQLKT---GIMMHYVDIG--HGHPIILLHGFPELWYIWKYQG--------TIIS-----YgkSD--AKLACTQLLYSPSDKiqDIISLMDLLLLRQVAVIGHDWGGMVAWDLALHHPDRIRAVANMTPFMPPNPDINPLLGLKAKPGVFQYQLYFHQEGPPEAEFEKDLRRTFKLFLRNAS------EMEQIKLSTSDVLERGGFFVGTPEKVERSCMITEEDLDYFVENFRKTGF-RGPLNWYRNMEVDWKW-NLKIHPRKILQPVLMITSGKDPVILPAFAANMGAHVPNLSlhIEESSHFTPIDRPLELNNMLLDWLKRI-------------------------------------------------------    
  73 UniRef50_B8N040        Epoxide hydrolase, putative...   186     2e-45  1  13.3%  94:424     4:339     ---------------------------------------------------------------------------------------------ITDHNVVYADGKKIHYLAAGPANGPLVLFIHGWPGSAITWKAQIDAFASVGFRAIAPDMPGYGQSTARRVA----DDYCQEAIVEGMLALLADTGRDAAIWVGHDWGAGVTSSVATQHPEVVKALVNMSVPFHTIERGWQGFLPYVNRELYPADEYEFGQWDYMKNWEENFEKTVEWFDSDIAGmpPSSRFAPLFATVRKSGWMGGAPKPPSVEMTGPPVLPAEVFDSFVQDMQRTGFWAGSAYYLHHARNAEYN---GKREGKLDQPVLFIHDARDVIctSRLVEPMRENCSNLTEVTIDAGHFAQYEKPEEVHAAIFRFIVEELPSEWP-------------------------------------------------    
  74 UniRef50_Q18GM0        Epoxide hydrolase-related p...   186     2e-45  1  11.0%  96:414    26:304     -----------------------------------------------------------------------------------------------TSKLVDVGDVTLHCVLAGPSDGDVVLLLHGFPEFWYEWHEYILPLAAAGYRVVVPDQRGYHRSERPDAIS----AYHLDALATDICGILDAIGAKKTHIVGHDWGAFVSWWVALHHPARLRTLSVINVPHPTAFKQTLW---------------------------RNPKQVLRSWYVGFFQLPRVPEALAQVGNWASVTAM-------MQRSSRDGTFDSTDFERYRTAWGLDGAYEAMINWYRAVVRSNPQPVQT----QISTPTMLLWGAQDTFLTRSLAEKSAQYCTNSEVVMFeaTHWihEEQTQVQNRLLDHF-----------------------------------------------------------    
  75 UniRef50_B7RWQ6        Hydrolase, alpha/beta fold ...   186     2e-45  1   9.9%  97:419     5:321     ------------------------------------------------------------------------------------------------HQYASINGIRVHYVDQG--QGMPVILCHGFPHLWFSWHRQITALAAAGYRVIAPDMRGMGQTDAPQDP----RYYDIDHIIGDLIGLLDHLQLEQSVFAGLDFGAFAIYDLALRHPERVMAVIGLeaAPHNPQESPLTEYRRMGEQHFLHIEYFREPPRADEELAAQPRRFLHKVFYTLSGSANYFDCFKYPPETAYIDAMEEP------PPLPWSWLSEQELDYFVEEYTRSGF-TGGLNWYRSMDMKWQQRKPFEGVRS-AVPAYFLGSENDVDLEGFHGEdmRAIFPDLRQvvSDAGHMVQLEASEKVNAILLDYLAEIA------------------------------------------------------    
  76 UniRef50_Q7NDH6        Gll4259 protein n=1 Tax=Glo...   186     2e-45  1  11.9%  96:422     2:288     -----------------------------------------------------------------------------------------------RHAFLEANDLRFHYAEQGMPDAPLVLLLHGFPDFWYSWRHQIPVLGE-HFRVVAPDLRGYHLTDKPAG------GYDLLTLSDDVRELILALGAREAIVVGHDWGGAIAWVFAHRCPAMCTKLVILNAPH---------------------------PLRFAEELRSNPQQLLKSWYILFFQLPWLPERLIEWNDYDFIEAAFRHA------EVRPGSFSDAEIRRYKQAAAMPGAMTCALQYYRSALSAPPPPETYNQP--VACPTLLIWGERDFALETRLTRNLERFVSGpvERLPDCSHWVHQEDPTGVNQLLVPFLTQPGRQA---------------------------------------------------    
  77 UniRef50_D1H8J5        Whole genome shotgun sequen...   186     2e-45  1  11.6%  99:416    81:361     --------------------------------------------------------------------------------------------------FWTWRGHKIHYVEQG--EGFPIVLIHGFGASAFHWRYNIPELAKR-YKVYAIDLLGFGWSDK------AIIEYDAMVWKDQVEDFLKEIVKEQAILVGNSLGGFTALATAAELPEQVVGVALLNSAGQFGDIKGETIKSEETVLQTYFliFQRIVLGFLFWQAKQPARIESVLKS---------------------------------VYINASNVDDYLVESITMPAADPNAGEVYYRLMTRFMMNQSKYTLNSVLSKLSCPLLLLWGDLDPWVGPAKANRIKEFYPNTSLVNLQAGHCPHDEVPELVNGALIEWLS---------------------------------------------------------    
  78 UniRef50_A5U3V7        Epoxide hydrolase EphB n=16...   186     2e-45  1  14.5%  96:420     4:356     -----------------------------------------------------------------------------------------------VHRILNCRGTRIHAVADSPPdgPLVVLLHGFPESWYSWRHQIPALAGAGYRVVAIDQRGYGRSSK----YRVQKAYRIKELVGDVVGVLDSYGAEQAFVVGHDWGAPVAWTFAWLHPDRCAGVVGISVppGSPFGERRPSDYHLELAGPGRVWYQDYFAVQDGIITEIEEDLRGWLLGLTYTVSGEGMMAATKAAVDamAEGARLKDAFVYPETMPAWFTEADLDFYTGEFERSGFG-GPLSFYHNIDNDW--HDLADQQGklTPPALFIGGQYdtIWGAQAIERAHEVMPNYrhMIADVGHWIQQEAPEETNRLLLDFLGGLRP-----------------------------------------------------    
  79 UniRef50_A4JMP7        Alpha/beta hydrolase fold n...   186     2e-45  1  13.0%  96:417    10:289     -----------------------------------------------------------------------------------------------THHTLTANGIRQHYLDAGS--GPVVVLLHGFPETSFAWRHQIPVLA-RTYRVIAPDLRGYGETDKPAS------GYDKRNMARDIVGLLDRLGIDKVALVGHDRGARVATRLVKDHPERVDRLVVMDNVPTRIVAENMRPETARA---YWFFLFHLVADLPETLIAGNEEPWLRHFFSD--------------------------------WCYNPHTIEGADFDTYVKAYKRPGAVRGAMSDYRANAEDVA-QDKADADVRIACPTMAIWGEDFYAVgfDMKAVWESMATNLRAeiARCGHLPQEEQPERVNALLLEFLSG--------------------------------------------------------    
  80 UniRef50_UPI0000D5579A PREDICTED: similar to AGAP0...   185     3e-45  1  12.5%  38:446     2:367     -------------------------------------ARDLRIVTISPWEILKLHFFTFIFGVwwIWDPKSFFINQQRDNPPACLVDSSLGQ----HKYIKLKGVKFHYVESGSEDRPLVLLLHGFPDCWVSWRHQIPTLS-QHFRVVALDLKGFGDSDKPSSRKT----YRIDMILEELRQLIISFGVSSCIVVGHDIGALLGWCLAHQFPEVVEKLVAVSCPHPNIYRTNLHTSWNYRW-------------------------------LNFVQLPYFPEVDALRNDVKIITEYHRHL-----------PANDTFLDAYKYAFCRKEDWTGPLNYFRNLLFI----EIAENTRTIQVPVVLVTGDRDKFIKLESIVKSTDFCEKFYVKIVdaGHFPHQEKYQQFNEILLKYlrNVPNGLERSPSKGIMNrmGAVSSTVKYGNS---------------------------    
  81 UniRef50_Q21147        Caenorhabditis epoxide hydr...   185     3e-45  1  11.9%  22:415    56:400     ---------------------HDLAFGSFSYYLTMFLYRILVRLLQFYYFVKFSAILFLGFAVKGRSLFEKKQR----EKPNVLEGWD--------SRYIKLKKVRLHYVQTGSDDKPLMLFIHGYPEFWYSWRFQLKEFADK-YRCVAIDQRGYNLSDKPKHV----DNYSIDELTGDIRDVIEGLGYDKAIVVAHDWGGLVAWQFAEQYPEMVDKLICCNIP---------------------------RPGSFRKRIYTSWSQFRKSWYMFFYQNEKIPEMLCSADDMKMLELC---FRAKEIGIQNNKNFTDEDLEAWKYSFSMNGafKYPINYYRNIFNakQQADLV-----LEMPTLIIWGTADGALDIEAAVDSLNTLKQGTmkIEGASHWVQQDEPEMVNEHIKKFL----------------------------------------------------------    
  82 UniRef50_B0XJ30        Abhydrolase domain-containi...   185     3e-45  1  10.6%  48:416    12:335     -----------------------------------------------LYGIGAFYALQFVLQTALQFLADPDLAAWLAKK-RSVAPALLGDPRYGTHRFVEVNGVKLHYVEKGDPGKPLMLFLHGFPEFWFSWRHQLEEFSKDYWTV-AVDLRGYGQSEGFDDRA----AYQIDVIVEDIRSLVRALGRDRVIVVGHDWGAVLGFQFVSKHMDMVDRYVMMGGPSLNGIR---------------------------RLLLISWQQFRMSWYTFFFLIPWLPEFYIWGKDFGYIEQNMGGF------------LTKSELEAYKYTFSQRKAVTRAIDYYRENLSFLRKEQPLPKLENHS-PGLYLIAEHDQFISLRSGRVMERSLPNLWYRIVPggHYMQQESHRLVNKMLRDFLD---------------------------------------------------------    
  83 UniRef50_B2WB19        Epoxide hydrolase 2 n=2 Tax...   185     3e-45  1  11.1% 103:418    13:339     ------------------------------------------------------------------------------------------------------NGETTHYYEGGSKEGTPLIFIHGWPDIAESWEHQLSHFAasKYRVIAPDMRGYGDSTAPT----TKEAYSLSVLVSEMVEFVEQLGINKAIWVSHDWGSGLTNALAAHHPELFIGVANLCVPYRSVELGFDYLVSMVNRDIYPEDEYKWGQWEYMKYYQLYPEESVKMFDSAVEglKQDHSQSYGKPSPMARIIRDGGLFGGHPEKIPDIPLEATMLDEHHYASLVRggSFGPVAWYLNHEANAEYGKSEKNGGVLEFPVLYIDGKYDAVctPKMGESQKSATKKLTVETIEAGHWVQLEKPKEVNEALEKWLSTL-------------------------------------------------------    
  84 UniRef50_A5G7L9        Alpha/beta hydrolase fold n...   185     3e-45  1  12.2%  98:416    16:315     -------------------------------------------------------------------------------------------------RTVEIDGQSIFYREAGSREAPTLLLLHGFPTSSHMYRDLIPALADL-FHLVAPDYPGYGNSSIPR---VDEFDYTFDNLSEILDKFTVKLGLERYSLYLMDYGAPIGFRLAAKYPERVESLIIQNGNAYDEGIDNNFWEPIKEYWKDRGAINKGLDNEWWKNIKA----------------AYKQPNMSNEDALRFLLTLEATQWQYTNGVRNKEAISPDNWHVDQRLLDRPGNAEIQLQLFYDYGSNPPlpSWQAYFRQHQPRTLIVWGANDEIFPAAGAHPYKRDLKNLDFHLLNTGHFALEEDGDVIAKHICEFLA---------------------------------------------------------    
  85 UniRef50_B8HXY2        Alpha/beta hydrolase fold p...   185     3e-45  1  14.0%  88:416     1:290     ---------------------------------------------------------------------------------------MTDPANRWQHQFIQTNKIRLHCVTQGEGD--LVLLLHGFPEFWYAWRFQIPALA-RYFKVVVPDLRGHNDSDKPAS------GYDLSTLAADVLGLIQALGYEKAYIVGHDCGGLLAWHLAQKFPQVVQRLAVLN-APHPDRLFRDWLGNLEHLSRNWYLFALQVPGLPEYLIRHNLRRFLQDWFGQHSIR--------------------------------KAAFSSETMQIYQSALEKAGSLTAVLHYCRDLLSPPSwpQLLRQPKPIAIPTLVLWGKEDNLFSPALTEGLERWvpFKLKVLPECGHWAQQEVPGIVNREILDFFR---------------------------------------------------------    
  86 UniRef50_B1K6N9        Alpha/beta hydrolase fold n...   185     4e-45  1  14.4%  92:417     6:338     -------------------------------------------------------------------------------------------RHPLVSHTLNMTGQSTHYLESGPADGPLMFFLHGWPSMSLMWRAQMEAFAADGWRCVAPDLRGYGGSFAPEA----NDAYTIEAAVTDMTQLHDHLGGTPAIWVGHDWGSVVVAELSAHEPERSRGVVLTSLAYQPDGHALSTIVPLVDRTIYPADAYPDGQWDYYRYYMTHfdRTASLASIFRSGNPDGIGKVSPNAVVSRNGGRFGAAHCAPPTRPDPALWPPADFEALVASFNTHGFRPA-CAWYLNDEANIDYARNAPDGGRLSQPVLFVNGEFDQICSirQGDPMRAACADLTVTTMPAGHWLPLECKALHIEVIRAWLRS--------------------------------------------------------    
  87 UniRef50_Q5WCE1        Putative uncharacterized pr...   184     6e-45  1  12.7%  97:418    14:300     ------------------------------------------------------------------------------------------------HRYAHVNGIQLHYVEGGEQHSNTVVLLHGFPQSWVLWRFVIPDLVKR-YRVLAVDLRGYGDSDKPEGI----EGYTKANMAKDIYDLVTHLRLEKVTLIGHDRGARVARRFALDYPDYVASLCLIDILPLEYVYDNLSASEAAKKYWHWVFQVVAELPEAL-------------------------IEGREEVYLGALFKRTPHLLEQLKA--------DGVWDEYVRIWKQPGGFQAALNDYRAalDLPNYRVEKEMNKNINQPCLLLWGENGNLAGQPVlkEVATMVTGKELA--GCGHYVPEEQPDAMMQELVPFLEDI-------------------------------------------------------    
  88 UniRef50_Q1VYH1        Alpha/beta hydrolase fold p...   184     6e-45  1  11.9%  49:417     1:324     ------------------------------------------------MKNNFKNIITLFFTVGTLFSTMYATSQETVKIPEP-----STFLQEVDHGYANSDGVKIHYAEVG--KGPLIIMIHGFPDYWYTWRHQMEVLS-KDYHVVAIDQRGYNKSDKPKGV----ENYSLKKLVGDVAAVIHHFGKEKAIIVGHDWGGAVAWQFAIHLPQMTDKLVILNVTHPNGMR---------------------------RELATNPVQQESSSYARKfgTPDDPTILFGKPMTAENLASWVK--------------DPEVRIHYIEAYQRSDF-TAMLNYYKANYARPPYQKAWEdlPKLKMSVLIFHGLDDWAINAHGLNNTWEWLedMTLvvPGASHFVQQDASKLVSTTLQWWLAS--------------------------------------------------------    
  89 UniRef50_B2JS18        Alpha/beta hydrolase fold p...   184     6e-45  1  11.8% 107:419     1:307     ----------------------------------------------------------------------------------------------------------MHYVDEG--QGPLVILLHGFPYLWYMWRRQIPALAAAGYRVVVPDQRGFGQSERPDAI----EAYDISQSVGDMVGLMKVLGESSAVIIGHDLGAWVAQAAAMLRPDLFRGLVMLNTPVPPRGspTIALNEMARGKGYHHLYFQELGTPDREMSNDTRKTLRSIFYSVSGSAVGAERWRIFFEPGQPLLDAFTDPKD------FPSWLSSRALDYYVDEYSRTGF-TGAINYYRCRDRNWEITAFLD-GAVVRQPSMFISGAADASLEPAPIRalEAYLPGLrvLLPGVGHSAAEERVDQVNELLLDFLRGLS------------------------------------------------------    
  90 UniRef50_D2RS70        Alpha/beta hydrolase fold p...   182     2e-44  1  11.0%  78:413     2:298     -----------------------------------------------------------------------------ATMPSTWTETDAEltVDAESTVREVNEVRLHVVVAGDEDDPLVVLLHGFPEFWYGWRHQLEPLVDAGYRVLVPDQRGYNLSEKPAAV----RAYRISECSRDIVDLIATEGRDSARVVGHDWGGMVAWDLALRRPSAVDRLAIVNAPH---------------------------PTVYRQHLLANPDQLRRSVYAACFQLPLVPEFVCRRGDFRFLE-------RALRETAAPGTFTDVELERYRRAWRRDGAISGMLDWYRANGRNPPSPP----LEQVETPTLIVWGEDDTALTTPLAVDSDQRCHRSRLELleTSHWVQHERPARLTELLLE------------------------------------------------------------    
  91 UniRef50_Q0IWN4        Os10g0498300 protein (Fragm...   182     2e-44  1  13.0%  73:419     1:329     ------------------------------------------------------------------------TSAARRELEPPGSHGHGGAAAADRARPPPRAGLTLHVAQAGKGELGTVVFLHGFPEIWYSWRHQMLAVAAAGYRAVAPDWRGYGLSDQPPEPEAAE----YDDLVEDLLAILDALAVPKAFLVGKDFGAMPAYSFALCHPNRTCGVMCLGIPFGVNSSSLNTLPE-----GFYILRWAQPGRAEADFGKYDIRRVVRTIYI-----------LFSRNEIPIAKEDQEIMDLADLSTPLPEWFSEEDLDVYSSLYEKSGFRYPLQMPYRSMHQNKPIGD-----AKFQVPVFVVMGEKDYVFKIPGIesMEKHAPDLKItiPEGSHFVQEQFPEFVNELLLSFLKDHP------------------------------------------------------    
  92 UniRef50_Q15ZT2        Alpha/beta hydrolase fold n...   182     2e-44  1  12.3%  94:419     1:273     ---------------------------------------------------------------------------------------------MSTTKYVTVEGHQIAYEEMG--EGTPLLLFHGIPTNRTLWRNVMPQLSSQ-YRVIAPDLLNYGESDMP-----QDTDVSINAQSRIMSKFMGALGISRANIAGHDIGGGVAQLMAVKHPEKVDAIVLIDSVCFDSWPIPE---------------FTPLLEPGVEEKTTTDELV--SILKDFLPKGVY----------------------------DQSVMTEELVRLYVGQWSSDQGKAAMFRNLRRLNKEYTQA-IAGELKHLPHKTLILWGDHDNFQKPEYAPQLAQTIPNASLVWLeaGHWSIDEQPEKVTKLISDFLQQTE------------------------------------------------------    
  93 UniRef50_B9DGW5        AT4G36530 protein n=13 Tax=...   182     2e-44  1  11.0%  99:416    83:363     --------------------------------------------------------------------------------------------------FWEWRGHKIHYVVQG--EGSPLVLIHGFGASVFHWRYNIPELAKK-YKVYALDLLGFGWSDK------ALIEYDAMVWTDQVIDFMKEVVKEPAVVVGNSLGGFTALSVAVGLPEQVTGVALLNSAGQFAAESRKREEADETVITKFIviFQRVVLGFLFWQAKQPSRIESVLKS---------------------------------VYIDSTNVDDYLVESISKPATDPNAGEVYYRLMTRFLTNQSRYTLDSVLSKMTCPLLLVWGDLDPWVGPAKAEKIKAFYSNSSLVHLQAGHCPHDEVPEAVNKALLDWLS---------------------------------------------------------    
  94 UniRef50_C2AN44        Predicted hydrolase or acyl...   181     4e-44  1  11.3%  96:432     4:344     -----------------------------------------------------------------------------------------------TPHTVRTERHTTGYLASGPEDGPLLVFCHGWPELSYSWRHQLTALGGLGFRCIAPDMRGYGASSVPAA----KDAYRREEIVADMLELLAGVGREAAIWIGHDWGAPVVWNIATHHPGVVDAVASLNVPHFPSGGpyPADEYPYGQWDYQVHYLKHFDEVTAQFESDIPHFIAALFRVGEPSHLDRPAPTAVVSRNGGWFGGGAVPR----VPIDPAVLTTEDHAVYTAAFERNG-MAGPNSWYVNGAADAEYAARELNHGRIDVPVLFLHGRYDatVRSSLADPMRAACANLTEVILDCGHWTAEEKPAEVSASLARWIATRVPGRWPAPAENHPA-----------------------------------------    
  95 UniRef50_B2WHB7        Epoxide hydrolase n=2 Tax=P...   181     4e-44  1  10.8%  84:417     8:335     -----------------------------------------------------------------------------------LQPINPEEDSRVQHKTAVLNGNTYHYLYAEPQSghTVFLIHGWPDLSIGWRYQIPHLVELGFRVVAPDMMGYGGTDAPQVPPNPINLYGLKQASDDIAALAKVVEAPQIILGGHDWGGFVVWRAAQWHPNLVSHVFSVCTPYAPPREQFASIEDLVKGPLPQFGYQIHLASGEVEKIVKNETTIRQFLQGMYGGKGPNDEPVF--------VPEKGVVAENLGLVGESRILNGKELDFYVKQYLRHG-IHPTLNWYRTRRINFEEDKGLLGKEKVMQPVLFIQATYDSVLKPEMSKSMDEFVPNLTRGEVEASHFALTQKPVEVNGIIQKWLET--------------------------------------------------------    
  96 UniRef50_B8LY11        Epoxide hydrolase, putative...   181     4e-44  1  14.8% 119:415    56:340     ----------------------------------------------------------------------------------------------------------------------TVFLLHGFPDLAIGWRYQIPHLLNLGFRVVAPDQLGYGRTDAPDDPK----EYAFKKIAADLAQLAHQLGESRIVLCAHDWGAALAYSIYHHQHSLISHIITVCVPYSPPRTNYIPLEELVDKFLPSLGYQLQFRSGEVEKAVRSKDDVRQFLTIMYGGR--------TAEGEGGWSASKGVKLDKLGSFRESPLLKGEDLEYYATEFSRYG-IHGPLNWYRLTEINFNDEKQYASTPKIEVPLLFIQALRDPALPPemGKSMGKAIPDLTVKKLNTSHWALTEDPRGVNEAVEFFL----------------------------------------------------------    
  97 UniRef50_B0T6N2        Alpha/beta hydrolase fold n...   180     8e-44  1   8.1%  90:424     4:343     -----------------------------------------------------------------------------------------PLPDMPPVRFAPVNGLQMAYYEVARPSNppIVLCHGFPELAFSWRHQLRALETAGRWAIAPDQRGYGLTGGpeGAPDPVESYDADQLTSDLAALLDTLGAKQAIWCGHDWGAIIAWQMALRQASRTAAVISMNVPFQPHGRTDPIEQLRAHFGEQTYTVEFQTAETPDAVLNADVRRTVSFFMRRDWGGEDVRRGVGEGYAFAAFPFVRMLDQYDPAADERPSILTDEEFGVFVRMFEQTGF-TGPINWYRNLSRNWRTS--AALARRVDVPALMIMAEFDPVLPPAACDGMERYCGRLEKVLVgsGHWTQQEQPQQTNHFLLDWLDRHFPVPGA-------------------------------------------------    
  98 UniRef50_A4QX26        Putative uncharacterized pr...   180     9e-44  1  12.0%  99:415    11:337     --------------------------------------------------------------------------------------------------FEHSGDKTTFYWSAGPSNGPLVILIHGWPANGETWKPQLLALGGLGFHAVAPDTRGYGRSSVPAGP----EAYALEHHVSDMVALLAHLRRDKAVWIGHDWGAGLVWAFAAMQPDKCVGVCCLTVPYAVLGQGIELLAspYAQWDYMAFHNEQPEASAAQLAANVPNTVKTLYRAGSPETYRKPAFSAMMRKMGGWFGSPTAGAPDVPLEATLFAQDEPAYERMVAEFQKNGF-EAPNDYYRNFDKNSEYLAKAPNGGRLAFPVLFIGAQWDSIVdsRLAEPMRELCDDLTEVTIEAGHWVAMEKPQETNAALVRWL----------------------------------------------------------    
  99 UniRef50_Q6MC44        Putative uncharacterized pr...   180     9e-44  1  13.8%  43:418     7:315     ------------------------------------------RFIFPSFTILAVLFTLYFFTAISLLEAEKQDELKKGKLFWEWQTPQGNWQ--------------VHYTEHGSGSKH-LLLIHGFRAHSFTWRYLIEPLTQAGYHVWTIDLIGYGLSDKP-----LNAAYDADFFIEQLKSFMDAKQISSAHLIGSSMGGGLALNLTLDYPEKVSSLTLINALGYPLDLPFYLYLTRHLDPLWFSFVSPPVIRIGLKQIVFDPDTV-----------------------------------------------SEEQVLAYSFPYQFPGGTQASLTTLKQFDKQKLV-DLSQRYHSLKHPLLIIWGDKDKLIPITHYERFVKEFPQAdlLIPNCGHIPHEEKPILVTETILEFLGKH-------------------------------------------------------    
 100 UniRef50_B1WP98        Putative alpha/beta hydrola...   180     1e-43  1   8.9% 115:416    23:289     ------------------------------------------------------------------------------------------------------------------GKGYPLLLLHGYPQTHVMWHKIAPNLAQE-FTVIIPDLRGYGDSDKPIG-MTDHYNYSKRVMAQDQIEVMAQLGYDQFYLVGHDRGGRVAHRLTLDYPNKVKKLALLDIAPTY--QMYSETDQEFAKVYYHWFFLIQPFPFPETLINQNPDFFLTHCLQS--------------------------------WSKTDRPFSSEAMAEYLRCFRDPNTVHGTCEDYRASATIDLEHDRTDMDKKIECPLLVLWgtYDVITS------WQKRATNVQGKAVNCGHFLPEESPEETYQYLHDFFS---------------------------------------------------------    
 101 UniRef50_Q1IK78        Alpha/beta hydrolase n=1 Ta...   180     1e-43  1  12.5%  98:417    12:292     -------------------------------------------------------------------------------------------------RTLNVEGLEVFYREAGPRDAPTALLLHGFPSSSHMFRNLIPMLADK-YHVVAPDFPGYGESSAPP---VNEFDYSFESFATITEKFTEKLNLSSYILYLSDIGASVGFHLAVRHPERVMAMIVQN-------------------------------------ADAHVEAINKAFLKTGLSDYWEDRSERSAQLLLDWLLTIEGTKWHYLHGvdPSKISPDNWVIDQAYLDRPGNKSIQLSLLYNAKRNLDAYawQEYFRKHQPPMLITWGKNDGIFTVEGAELYKRDIPSAELHLIDTGHFALEEEVDRIGSLIHEFLDR--------------------------------------------------------    
 102 UniRef50_A1ZHQ0        Hydrolase, alpha/beta fold ...   180     1e-43  1  10.2% 116:420    24:291     -------------------------------------------------------------------------------------------------------------------DGLVVVLLHGWPETSYCWEAMLDYL-PKNHRLIRPDLRGLGNSDRSLARP----AYLKNEMAADIWRILDKLEVNQVVLVGHDWGGAIVQEMAISQPKRVQAMVLMNILLINNLKGNLK---------------------------------AREAHLQNDNLPNWYHLLYQQENLPERLIeeEVWLRYFLRMSKQRLFPEEAIEEYLRAFKIPDTPTTSAYYYRTLPKDKKRWATYEE-HKFTMPSLYVYGKRDSVIIPSFLEHIDDCFEHITVKEFDAGHFVHEELPEEVAQALNDFLAEIEG-----------------------------------------------------    
 103 UniRef50_C6AVN0        Alpha/beta hydrolase fold p...   180     1e-43  1  12.7%  96:417    45:324     -----------------------------------------------------------------------------------------------TYHTVDVDGTKIFYREAGPADAPVVLLLHGFPTSSHMFRNLIPILADK-YHVIAPDYPGFGQSDAPDHTK---FAYTFQHYADIVDSLLGKLDIKEYAMYVMDYGAPVGYRLALKHPDRVAALIVQNGNAYEEG------------------LKEFWDPIKAYWKNDTPENRQALLVLFKP---------------------ETTVFQYTDGMSDLAHIDPDNWGHDQALLDRPGNNDIQLDLFHDYGTNVPLYPEFQKFFRDKKptLIVWGKNDKIFPADGASPYLRDLPHAEMHLLDTGHFALEDKLPEMAPLIHDFLDR--------------------------------------------------------    
 104 UniRef50_Q12G35        Twin-arginine translocation...   179     1e-43  1  11.8%  37:417    16:356     ------------------------------------GANSHRRQFLNLTAIGLTGAAALAalVSAASSTTSAINSKGTPMSTSNLQNASANAPRASFHSVEADGVKVFYRESGPAQAPVILLLHGFPASSHMFRELIPRLASK-YRVIAPDLPGFGFTSVPDTR---HYAYSFDSLARTTEAFVEALGLKRYALYVFDYGAPVGFRLALAHPDRVTAIVSQNGNAYEEGLGEAW------------------APFQQYWAAPNSENRKALLSALTLETTHWAYTH---------------------GLPNPQAVAPESYQLDAALMARPGNSEVQLDLFLDYASNlrYPAFQAYFREVKPRLLAIWGKYDQLFIPAGAHAFKRDNPNAVVKLLDASHFALETHVEEIAAEMHVFLRS--------------------------------------------------------    
 105 UniRef50_Q096Y8        Epoxide hydrolase n=1 Tax=S...   179     1e-43  1   9.8% 105:420    11:333     --------------------------------------------------------------------------------------------------------HRTSYWTGGPANGPLMIFIHGWPELSSMWWHQLECFSALGFRCVAPDMRGYGGSSQP----SSLGDFALEHIVEDMLELLGGLGAERALWIGHDWGAPVVWSLASHHAGKCIGAVNLCVPYLANGFAPPNLVPLVNRTLYPEAQYPAGQWDYMLFYEKDFEAVRAVFEANVPALFKALIRRGSAEAVrrDGGWFGGASQAPVLPRDEAVLSQEALDRYVEAFETNGFF-GPNAWYMNHRQNMAYAAKAPGEGKLRLPVLFLHAAYDAVcdSRLAEPMRRDCLDLTEAVLETGHWMVQEQPERVNAAIARWLAARVP-----------------------------------------------------    
 106 UniRef50_Q01398        Haloacetate dehalogenase H-...   179     2e-43  1   9.7%  99:417    10:292     --------------------------------------------------------------------------------------------------TVTVDGVDIAYTVSG--EGPPVLMLHGFPQNRAMWARVAPQLAE-HHTVVCADLRGYGDSDKPKCLPDRS-NYSFRTFAHDQLCVMRHLGFERFHLVGHDRGGRTGHRMALDHPEAVLSLTVMDIVPTY--AMFMNTNRLVAASYWHWYFLQQPEPFPEHMIGQDPDFFYETCLFG-------------------------------WGATKVSDFDQQMLNAYRESWRNPAMIHGSCSDYRAAATIDLEHDSADIQRKVECPTLVFYGSKGQmqLFDIPAEWAKRCNNTTNASLPGGHFFVDQFPAETSEILLKFLAR--------------------------------------------------------    
 107 UniRef50_B2AVU5        Predicted CDS Pa_7_1770 n=1...   179     2e-43  1  14.6%  89:423     7:343     ----------------------------------------------------------------------------------------PNDPRVKHHTTTLPSGHTYHYLEALPSSGTpvVLIHGFPDLSFGWRHQIPFLTSLSLRVIVPDMLGYGLSSAP----SPIPPYSYKSLSTDLASLIQLVSPSHpiLGGHDWGGAIVWRLALRHPALILGVFSICTPYNAPNSqpKKVLIDQFLPNFAYQLQFEDPTLENKIQAAGRDGIRKFLNILYGSKDSKTGKGHFVANVGIDLSLFDEGVKVEKSELLSEEELEFYVDQY-----ERNGVHAPMNYYRTWDVNYeaDLVKEGKSKVEVPGMILTATRDTALPPKMADNMEQFFPRGLirEVEANHWATWENPEEVNKAIGEFVGELLKGRA--------------------------------------------------    
 108 UniRef50_A3FNW7        Esterase n=1 Tax=uncultured...   178     4e-43  1  12.2% 107:417    18:269     ----------------------------------------------------------------------------------------------------------IHYEEVNPAGSPPVLLLHGLGSAGADWFFQFEALSGAGFRVLAPDLRGFGRSSAPPKI-------TVKAMADDTAIFLKKLNAHPAHVVGISMGGTVALQLALDHPELVSKLVLVNT-------------------------------------------FARLKFTSPKEVLYLLTRLLVASLMGP---------EKQAEMVARRVFPHPGQETLRNNLRQRILHTNPYS-YRSALQSLQKFDVSHRLKELKMPVLVITGAEDTTVPPKVQEEMAKAIPNARHVVVEggHGIIADNPQTFNRILIEFLRG--------------------------------------------------------    
 109 UniRef50_UPI0000525771 PREDICTED: similar to Caeno...   178     4e-43  1  13.2%  45:415     4:346     --------------------------------------------LLVRWITGVLFFAIGFLWAVflFGGKKGITSFFEKKDHSKLPDVAKAVDVQHDFVSTKSGMKFEYVHAGEADKPLMLCMHGFPECWYSWRYLIEKFRDR-YHIVAFHSRGYGNSDKPKEI----NKYHMNYLVNDVAEIIEALGYPRATLVAHDWGGAIAWEVPKYFPHLVDKVIIMNAPNTTAARKGIGFS-----------------------------QFLKSWYICLYQMPYLPELLVQSKDFGLLGSAFNG--KKMGIKNKSNKLTREEIDVYKHYIG--NNISGPLNYYRAINPIYRGFGLKAKTRKVVHPVLLIWGDQDAALGKELAELAKGGATDITVaiPGASHWVNQDEPEKVHEQMEKFL----------------------------------------------------------    
 110 UniRef50_A7HQ88        Alpha/beta hydrolase fold n...   178     4e-43  1  11.9%  99:417    12:289     --------------------------------------------------------------------------------------------------VAHLDRVALHYAEMGPEDGPLVILLHGFPDSCIGWRHQMPALAKAGFRVIAPDQRGYGVSGKPRGVK----AYDLDELAEDIVALATHFGETRLRVVGHDWGAGVAWWLCSTRGEAMEKAAMINAPH---------------------------PAIWKDAMYKDKAQRKKSRYVKAFRLRVLPEMMIRRKNF-------SALVEALKGSARPGAFDEAEFDAYRKAWREPKAITSMVHWYRALLK----KKLPAKPAPIATPVLLIWGEKDEFADVSLAKRSIALcrGGTLFFAEGTHWVHREEPQPINDMLITFLRS--------------------------------------------------------    
 111 UniRef50_A5WII9        Epoxide hydrolase ephF n=13...   177     5e-43  1  10.6%  89:417     5:294     ----------------------------------------------------------------------------------------PALEGVEHRHVDVAEGVRIHVADAGPADGPAVMLVHGFPQNWWEWRDLIGPLAADGNRVLCPDLRGAGWSSAPRSR------YTKTEMADDLAAVLDGLGVAKVKLVAHDWGGPVAFIMMLRHPEKVTGFFGVNTVAPWVKRDLGMLR---NMWRFWYQIPMSLPVIGPRVISDPKGRYFRLL---------------------------------TGWVGGGFRVPDDDVRLYLDCMREPGHAEAGSRWYRTFQTREMLRWLRGEyaRVDVPVRWLHGTGDPVITPDLLDGYAERASDFEVELVDsgHWIVEQRPELVLDRVRAFLAA--------------------------------------------------------    
 112 UniRef50_B9JML0        Alpha/beta hydrolase protei...   177     6e-43  1  14.6%  96:416     8:283     -----------------------------------------------------------------------------------------------THHTAVVDGVRLHYVRAGSGD--PLVLLHGWPQSWLEWRHIIPTLASR-FTVIAPDMRGFGDSDKPTS------GYDKRTVAQDIRKLVQQLGFSEINLVGHDIGMMVAYEYAASHPAEVRRLAVLEAGL--PGLGLEALFDAALFPQFWHFGFFMAPGVAETLVAGHEKMFLRHFIRHLA--------------------------------YDTYAVTEDDINEYADRMGAPGALRASFEHYRAFPIDARNNQENAR-TKLTMPVLAIGGDhdQVG--HIMRPLADKVETA--VIEHSGHWIPEEQPKQLADLLTGFFS---------------------------------------------------------    
 113 UniRef50_B6Q6P2        Epoxide hydrolase, putative...   177     7e-43  1  12.4%  98:420    24:337     -------------------------------------------------------------------------------------------------RKANLNGRNYAYIYGEPASgaTIFLLHGFPDLAVGWRYQIPHLLNLGFRIVAPDQLGYGRTDAPDD----FREYAFKKIAADFALLAKQLGESRIVLCGHDWGAALVYRIYHHQRALISHIITVCVPYSAPRANYIPLDDMVARFLPNFGYQLQFRSGEVEKVIRTRDDVQQFLLSLYGGRTAEGEIGWS--------AEKGIKLNKVGNFRPSQLLKGQDLEFYTTEFSRHG-IHGPLNWYRLAEINFNDEKEHASTPKIEVPLLFIQALKDLALPPemSKSMGQMIPSLTVEKLNTSHWALTEDPKRVNEAIGRFLEKHFP-----------------------------------------------------    
 114 UniRef50_C7MX36        Predicted hydrolase or acyl...   177     7e-43  1  11.8%  91:429     3:292     ------------------------------------------------------------------------------------------ARQTLITDSAHVNGVDLAYVDKGEGD--PVVLLHGFPDSHYLWRHQIDPLVEAGFRVIAPDLRGFGESSKPQEI----EAYEMRVLVNDIVGLTQHLGFSKVHLVGHDWGAAIAWMYAFLMPRRVDHLAVFSVGHPGVFSTPSIEQRKA----SWYMLFYQFPGVSEQLLRRNGWRLFKEIMG-----------------------------------------TEGDYQRYPRELAKPGALTAALNYYRANRSPAAELRADRNFPPVLAPTLGVWSTGDQAMLEEGMTGSAKFvpWRYERVEDSSHWIPLDQPELTTKLLTGFLGTEKPATMPTRRRR--------------------------------------------    
 115 UniRef50_B0CER8        Hydrolase, alpha/beta fold ...   177     7e-43  1  10.7%  89:416     3:292     ----------------------------------------------------------------------------------------AALEQTPTSQYWQWQGWRIHYVQQG-EQGPCVVLLHGFGASTDHWRKNIEVLS-RYYRVWAVDMLGFGRSEKP------DMDYTGDLWRTQMQAFCEEVIQEPVFIAGNSLGGYASLCFAVDCPKWTRGVVLLNCAGSFTEVEPKPELPAWRQQLQD----------------------RRKTVMRSRPVINVMSYFLFNRM------RKPDNIRKALSQVYKDQ--SAVTDRLVEEIYQPSLDKGARGVFAGVFKSPPGRKLDELLQSLDRPLFLLWGAADPWMTPQKAEKFQQFYPAADLVLVDAGHCPHDERPEVINAELHQWIQ---------------------------------------------------------    
 116 UniRef50_C1F0U7        Hydrolase, alpha/beta fold ...   177     7e-43  1  15.0%  92:411     9:287     -------------------------------------------------------------------------------------------QEPTHHRTATVNGRKVFYREAGDPSSPTIVLLHGLPTSSQMFRELIPTLADQ-FHLIAPDYIGFGHSEAP---SRQEFTYTFDNLAAHVRGLLAQLGVRSYILYMQDYGGPIGFRLFAENPEQVEGFIIQN-------------------------------------ANAYFEGVGDLPKQALGPLWNNRNAETEKP-AKEFLSLPSTKFHWTVGAKNEENINPDNWVLDQALLDRPGTQTYQIDLLENYKTNVASYESWQAAFRAHQptLIVWGKKDPFFIPAGAEAYLRDLPNAKLVWLDAGHFVLDENLRTVASEI--------------------------------------------------------------    
 117 UniRef50_A3VJF9        Putative epoxide hydrolase ...   177     8e-43  1  11.2%  98:418     5:320     -------------------------------------------------------------------------------------------------TFIETNGIRLATRIEG--DGPLVILVHGFPETAYSWRKQASPLVEAGYRVCIPDVRGYGNSDAPEAVS----AYAMEVMTRDFLGLAQALSEVPAVIVGHDWGAPLAWNTARLFPEQFRAVAGLSVPYAPPGdaPIDLYHKLFTDKGRFFYQVYFQDEGVAEAELEADVEDSLAKFYYAWSGDCPPNGWPN----DKAHGDPVLKGLPRPDLPLPWLTQDDLDRYAADFRTSGF-RGPLNRYRNQREDHAFLKAHPSNPIIQQPSLFLYGDRDPvtPPEDL--LPKTLADLrhRLPGVGHWTQQEAPEAVNKALIDWLATL-------------------------------------------------------    
 118 UniRef50_Q2J800        Alpha/beta hydrolase fold n...   176     2e-42  1  12.5%  42:416     2:316     -----------------------------------------SCAVARIYMLSIISLFLLGFLAVSFFAYGVEPGRNLKETPSFLKE--------RTTRYMDIGDTRLHYLKQGA--GRPVILLHGGGTWLYSFRKNIDELA-REHTVYALDMPGHGFTT-----YQDPATLTLAGFATLLKDFLAKQGIVQADFIGSSWGGGWALYFAEVYPDEVGRIVLIDATGTAEIAAHDGSSWKYLSYPLVGELLVH-------------------FFSFGNVRKDIHENLFSNP----------------------SAVSDAEIRQIYIPITYSRNLKAQYILQRNLN----WSEVDRHLEKVHNETLVIWGKDDRYIPIKFGQNLEARLPNARFviENAGHLPHEEQPERVNALLLKFLQ---------------------------------------------------------    
 119 UniRef50_Q7UHE5        Probable oxidoreductase-put...   176     2e-42  1  12.9%  96:421     9:285     -----------------------------------------------------------------------------------------------RQQTLSLDGvnVAYTDVG--EGEPLLLLHGIPTWSFLFHDVIDTLS-QHYRVIAPDMIGYGYSDRRD-----QFDRSIEFQADFVERFLEHLDVDSAHFVAHDIGGGVALILADRKPELVRSMVLSNSVAYDSW------------------------PVDEMLALGHP----------------------RNAKMKPEEMTEKLVESFQFGLSRPERLTEEFKEGIVTPYQERDGIVSLVRNAASLNTNHTTP-LTSRLGQMQQPTLLLWGEDDKWQPISTAEQLVKDMPNAELhmKNCSHWVPQDNPEEFASATLEFLQKIPVA----------------------------------------------------    
 120 UniRef50_C5FMH2        Epoxide hydrolase n=9 Tax=O...   176     2e-42  1  11.4% 102:418    21:339     -----------------------------------------------------------------------------------------------------VNGTTYGYILATPPDgaTVFLIHGFPDLSMGWRYQIPTLLDLGLRVVAPDCIGYGRteAPQFTPESAWRYGYMQCAKDMKELARQLGSSQVILGGHDWGGAVVYRIALYEPAFVTHLFSVCTPYWPPTKEFQPLEKVVEKIPFFGY-QVQFANGGLENILRSKEQIRQFLNAIYGGR--------TANGEGALSIEKGVLLEKLPGIQQTPLLSKSEIDYYVDEFSRTG-LHGPLSWYRTREQNWK-EEQGFLGRSIDIPVLFILATNDTALRPELSRNIDRNLKNLTKGEVVASHWALWQAPEACNALIKRWFESV-------------------------------------------------------    
 121 UniRef50_B8G5B1        Alpha/beta hydrolase fold p...   175     3e-42  1  11.9%  97:415    11:281     ------------------------------------------------------------------------------------------------ERFVTVDGFRLRVLTAG--QGPVVLLLHGFVVSADDWMPTIQTLATAGYCAIAPDALGFGKSDKPGGAV-----YTLRRYADLNAGVLTAFGVEHAAVIGHSMGGKHALATTILHPHRVERLVIVDSEGFMRLPLFMRKGGSLPFLGEAIATLSSFPFVVNMQLRAAFAQPDR-------------------------------------------YITPELVVRGQKTLSDPAIRNTMISLSRFYDANDLHGslWPRLAEIRQPTLIIWGKEDRLFPVKCAYEAKRALPHARliIPNCGHFPMIEATDHFHQLVLAFL----------------------------------------------------------    
 122 UniRef50_B8IV05        Alpha/beta hydrolase fold p...   175     3e-42  1  10.8%  93:417    10:292     --------------------------------------------------------------------------------------------PEPTTRSIRVRGMVMHMAEAGPEDGPLALLLHGFPEFWYGWRHQIGPLAAAGLRVVAPDQRGYGATGKPTDLGP----YHLDELAADVIALADAFGRDRIRVVGHDWGGLVAWRVAAQYSERIDRAAILNAPH---------------------------PDVFMDYVRRHPSQALRSSYIGFFQLPWLPETMLRAGDFALL-------RRALVTSSRPGTFEAATLDRYAAAWAEPGALTGMLNWYRALRL-----PRRPAPSPVQPPVLVLWGERDTALEAGLAEDSLARCADGRvrFPDATHWVQHEEPEAVNRALVGFLTA--------------------------------------------------------    
 123 UniRef50_B2UC04        Alpha/beta hydrolase fold n...   175     3e-42  1  12.2%  89:416     4:308     ----------------------------------------------------------------------------------------ATAAAITESQFAdpNGTRLHYASAGKRGAPLMLFVHGFPEFWYEWEAQLAAFGATHF-AVAPDMRGFNLSSKPAAV----DAYRPKFLVQDLEQFIAALGYERAIVVAHDWGGAICWNLAIQHPELVERLVIVNSPH---------------------------PWVFANALLSDPAQQASSAYMNWLRQPGSEEALAANEF------EKLEGFFSGMGQPVAEWFTPEVRARYHAAWSQPggLTGGVNYYRASpaPLRIDQMPPEAFIVKVPTLVIWGEMDMALPKSLLNGLERFIPDMRlrIPDGTHWVVHEQPERVNALIRSFVS---------------------------------------------------------    
 124 UniRef50_A5B8Q6        Putative uncharacterized pr...   174     4e-42  1  12.2%  96:407     6:271     -----------------------------------------------------------------------------------------------RHQRIKINGIWMHIAEQGT--GPLVLLLHGFPQFWYSWRHQMGCLANKGYHVVAPDMRGYGDTDSPVSPTS----YTVFHLVGDIIGLIDHFGEQKVVVVGADWGAVAAWHLSLFRPDRVKGL-------------------------------------EPGRAERAFARYDYLAVMKKFLLINKTDLLIAPPGMEIIDYLETPSL-------LPTWITEEELGVYADKFQESGF-TGGLNYYRAMDLSWELLAPW-QGSKITIPSKLIFGDKDIGFKDGGTKEYIelVPDHEVVILDGHHFIQEEKPQQL------------------------------------------------------------------    
 125 UniRef50_A8WBX9        Epoxide hydrolase n=1 Tax=u...   174     5e-42  1  11.8%  96:417     8:301     -----------------------------------------------------------------------------------------------AHDRISVAGLKLHCARAGSRtrPPLLFLHGFPESWYIWRGLMRRFAPDRL-VLAPDMRGYNLSDKPGDV----EAYRAKHLIADVIGLLDQYGIGKCVLIGHDWGGIVAWAAALAHPERFAKLVILNAPH---------------------------AAIFARLLANDPAQQRASGYIVKYRTPEAEAYLGRDDCAELRKSITEPALRA-------GHMTPEDAAAYLAAWRQPGAIAGMVNYYRAMRMAPPDRKTGETPKpvRVPTLVVWGMQDRFLLPQCLDGLERYVPDLQVVRldASHWLIRERPAEVERALAEFVRS--------------------------------------------------------    
 126 UniRef50_C1VE51        Predicted hydrolase or acyl...   174     5e-42  1  11.7%  91:416    16:297     ------------------------------------------------------------------------------------------ADHRVESTYRNVNGVQLHVVAAGETDAPLVVLLHGHPDFWYGWRAQIISLVEAGFRVVVPDQRGCNLSEAPTGI----DPYRTPNLCDDIRELIHDEGRESAHVVGHDFGAYVAWNLALRQPSVVNRLGILNVPH---------------------------PTVYRDTLRASLRQIVRSWYVWFYQVPKLPEWMLSRNEMENMVASLE-------VTSNQGTFDEETIHRYRAAWRHTGVGPR-INWYRGFRRSERPAR-----NTVTQPTLICWGEDDIALVPAMAQKSIDYCENGQLRmpDASHWVHHER-EEVTEALLQHLS---------------------------------------------------------    
 127 UniRef50_B2T9C4        Alpha/beta hydrolase fold n...   174     6e-42  1  15.0%  99:418    12:323     --------------------------------------------------------------------------------------------------TLQVGALRMRVASQGS--GPLVLLCHGFPESWYSWRHQLAALAAAGFRAVAPDMRGYGGTDAPPDA----DSYTMLHLVGDMVELVNVLGESKAVIVGHDWGAPVAWNSAMLRPDLFRAVVGMSVPFHPPAREDLLGALDRQGIRTFYMQYFQTPGVAEREFEADPEaiRRITFSMSGDGPERVVAGILVPGASFLDSTVDP-------ETLPGWLTSEEIAYVAGEFARTGF-RGGLNWYRAIRRSSELMAAW-RGAVIRQPSMFVAGARDDVLKFPGSQalTRVLPGLrhILDGAGHWIQRERSAEVNDLLVAFLRGL-------------------------------------------------------    
 128 UniRef50_A4XZ24        Alpha/beta hydrolase fold n...   174     7e-42  1  10.0%  97:417     5:313     ------------------------------------------------------------------------------------------------HRLLAVNGIELSLYSTGPEHGKPVWLLHGFPECWYAWHPQIEALAAAGYRVFAPEMRGYGA----SSAPADPAAYDLLTLCGDIQAAMDLLAQHQVAVVGHDWGAPVAWHLALLEPERIKTLGALSVPFGGRprPAIELMREAYAGRFHYILYFQQPGLAEAELDEDIGRSLRLLLGGLGDALLATDKPADARLFDGMPEGLP----------LPPWCNEATFAHYRRTFERHGF-RGALNWYRNFERNWQRTEHLAEL-QITQPTLFLLGENDPifEAPTLKRMADKVPRLERhlPGAGHWLQGECAERVSALLLDFLAR--------------------------------------------------------    
 129 UniRef50_C7PNK7        Alpha/beta hydrolase fold p...   174     9e-42  1  11.8%  99:420    53:332     --------------------------------------------------------------------------------------------------TVSVGDYHIFYREGGSSDKPTIIFLHGFPSSSRMWQPLLEKLSAD-YHVIAPDYIGFGHSSQPP---VDSFDYTFDNLSDYTDRFITQLGLNRFILVQQDYGGPIGMRIAEKHPEKIQAIIVQNAVSHNEGLSPLWETRKAFW--------------------ANKGQYYDAVKKNFISFEATRQRHIGSTPVPERI--------------NPDNYTDEYL-----FLNQPGMADIQLELFYDYQSNVryPRWQEWLSKHQPKMQVIWGQYDPSFTVAGAWKYKENVPDAEVHIVEGGHFALDEAEPVILSLMQDFLKRITA-----------------------------------------------------    
 130 UniRef50_Q8Z0Q1        Alr0039 protein n=10 Tax=Ba...   173     1e-41  1   9.3%  95:416     5:289     ----------------------------------------------------------------------------------------------FEQTIVDTTEARINLVKAG--HGAPLLLLHGYPQTHVMWHKIAPLLA-NNFTVVATDLRGYGDSSRPASVP-HHINYSKRVMAQDQVEVMSKLGYEQFYVVGHDRGARVAHRLALDHPHRVKKLALLDIAPTH--KMYRTTDQEFATAYYHWFFLIQPDNLPETLIGANPEYYLRKCLE--------------------------------KWGKDFSAFHPQALAEYIRCFSQPAVIHATCEDYRAAATIDLEHDELDMKQKISCPVLVLWGEKGIIGryDVLATWRERAIDVSGQSLPCGHFLPEEAPEETYQAIYNFLT---------------------------------------------------------    
 131 UniRef50_A7F7S2        Putative uncharacterized pr...   173     1e-41  1  12.4% 100:421    17:331     ---------------------------------------------------------------------------------------------------ADVNGRTYSYILAEPQNGatIFLIHGFPDLAFGWRYQVPFLQDLNYRVVVPNMQGYASSSSPQEVS----AYTYKTAADDVAALAKVIGATSIILGGHDWGGATVYRIAIHYPKLISAVFSICTPFFPPQQEYRSLT---VLPNFKYQLQFQGPDVEREIVGK--DKLRSMLNALYGGRTADGELGFSV--------AEGVLFSNLEKLGHTPLLSKEELDFYAEQYAING-IRGPLNWYRTGELNFEDERELAPllKVDIPTMFVAGSRDAALPPAMGEGMEKWFGEGKlkEVDTSHWALWEKPEEVNGYIAEFLQETFGG----------------------------------------------------    
 132 UniRef50_B3RUT0        Putative uncharacterized pr...   173     1e-41  1  10.6% 105:416    32:307     --------------------------------------------------------------------------------------------------------LRIHYVANGEKGKPLMLFIHGYPQFWYCWRHQLKEF-GKDYYAVAVDNRGYGDTGRPT----HMEDYDIKELIEDVKDIIVGLGYEKCTLVAHDWGGFIAWHFAHFHPEYLEKLIIMNAPH---------------------------PRIFKQYMETHITQALKSWYIFFYQIPYLPEFVESSSDYELIDIIMTSVIHS--DPKTKDIFTKDDEECYKYNLSRPSCLTSAINYYRQ---NVFIPPLPFKYDKITAPTLIIWGEKDDALNKELNNGIAKYVDSVILkmAEGTHWISEQFPEETNKHMRDFLQ---------------------------------------------------------    
 133 UniRef50_UPI000023D9E9 hypothetical protein FG1119...   173     2e-41  1  12.3% 100:417    11:326     ---------------------------------------------------------------------------------------------------TTTDGLKYTYDTVPaeNKATVLLIHGYPATRHDWKHQIQDLSAAGYGVVAPDCLGYGDSDKPLEV----EAYKLKRISKHITEILDKEGLNKVIGLGHDWGAGVLSRLAVWHPDRFEKFVFLSTGYNPPGIPMD--VDAINANGLKHYGKMPFGYWYFFNSLDAPS-LAASHLESFFSLVFPTENLKWIDNLGALGSARTWLNNNIITPL-PKYMTEEDRANWLAEFSKPNATVGSMNYYKALLRGVNVEdlTDEDRTLRVPVLTIGGTQDTIARPEYQRMNTEPFAAAGYtaVDAGHWMMYEDREGVKKALLEFIQE--------------------------------------------------------    
 134 UniRef50_C7PDD5        Alpha/beta hydrolase fold p...   172     2e-41  1  15.4%  98:415     8:283     -------------------------------------------------------------------------------------------------RTVDIDGLNIFYRESGAPSRPVLLLLHGFPSSSHMFRDLIADLSG-TYHIVAPDYPGFGQSSAPD---VKQFSYTFDNLSVVIERFIDRLQLRNISMYIQDYGGPIGLRIASRRPELIRSLIIQNANAY---------NEGLGDALEPLVAYIRDPGAAQEKEAR--------FFLTSAATKWMY----------------------LNGATDKTRISPDSYIIDQYYLDRPGNDEIQLALFRDYGSNIALYDEWHRYFKEHQptMVVWGKNDAMFIAPGGNAYRKDLPNAAIIQIDGGHFLLEEHHEQVAGLIKGFI----------------------------------------------------------    
 135 UniRef50_Q0BWN8        Hydrolase, alpha/beta fold ...   172     2e-41  1  12.4%  46:431     2:331     ---------------------------------------------LSRFLAVILTLGVILFAGWWSLKRDDIPYDQLETIYANSDSRYLALD----------DEKRIHFRDVGPRDAPAIILVHGFSASLHTWEPWVTDL-KRDYRVISLDLPGHGLSRCLD-----NDAIGMDQFVDVIYRVASALKVDRFTLAGNSMGGGAAWNYALAHPERLDGLVLVDAAGWPRTEGES----------------------------DDRPFVFRLLEIG-----------IARKLVKDLDMSSLIRPGLEDSFSDPSFVTDEMVDRYGSLARAPCHREALLAL--SAGRGTRAEATPELLSAITTPTLVMHGEQDNVIPFSHGEQFAAAIPGAELKlpNVGHLPQEEIAAQSLEDLRAFLTARVHGAAPASESELP------------------------------------------    
 136 UniRef50_A9RB19        Predicted protein n=1 Tax=P...   172     2e-41  1  15.2%  97:418     7:286     ------------------------------------------------------------------------------------------------HRHVQLSELKVFYREAGDPSKPTMVLLHGFPTSSHMFRRLIPLLASE-MHVVAPDLPGFGFTESPPAT---NFTYSFDHLANTVEELIQHLRITRFFVYIMDYGAPTGLRLMESHPERILGLVVQNGNAYEEGLTPFWVPLRKM------------------WKNRTPETEAPL---------------------REFLTLEATQWQYVTGMKDVSKISPDNWVNDMFFLQQSGHDQIQLDLFYDYQSNLPRYpfHTTFRKHQFPALIVWGQNDPIFGVEGAKAYLRDLPHAELVLLDSGHFVLEDHLEEVAAHMVRFVKAV-------------------------------------------------------    
 137 UniRef50_A3VT85        Alpha/beta hydrolase fold p...   171     4e-41  1  13.6%  81:419     3:302     --------------------------------------------------------------------------------PSPLSPHLTPREGIITE-MVTVGEMSFEVDMCGGGD-TLALCLHGFPEHNFAWRYQLPALAQMGYRVWAPNLRGYGRTTRPDGVA----AYGLDHLLDDIARLIDASGAKRTVLIAHDWGGIIAWTFAALQLRPLHKFVAMNIPH--PNALLKTRTLEQALKSYYILLFQIPRLPELALTANRAGLIRRIF---------------------------------QLSSTKPEMFPKEVTDIFRENVLDPGAITAMLNYYRAFFRHPLSAHGrkDLPKIITPSLLIWGEKDLALTKKSALNSADYVEDLQYalPDGSHWIQQERPDEVNAAMKAFLGPAP------------------------------------------------------    
 138 UniRef50_C1F611        Hydrolase, alpha/beta fold ...   171     4e-41  1  14.0% 105:426    21:300     --------------------------------------------------------------------------------------------------------LSIFYREAGQQDNPVVLLLHGFPTSSHQYRGLIDRLSDK-YRVIAPDLPGFGFSDAPDAAT---FSYTFDHLAEIMQGFVDSLGLTRYAIYVFDYGAPVGFRLALAHPERVAAIISQNGNAYEEGLSDGW---------------------------------------NPIRAYWQDPSQQNRDQLRAFLQAGTTQFQYTHGEADSTHIAPESYTLDQHFLDRPGNQEIQLDLFRDYQNNLAlpQFQQYLRTHRPPVLAVWGRNDPFFLPAGAEAFRRDVPDAEVHLLDAGHFVLEAHLDEAARIIRNFLARTLDTAQGAA-----------------------------------------------    
 139 UniRef50_C7QIB3        Alpha/beta hydrolase fold p...   171     4e-41  1  11.2%  86:415     7:293     -------------------------------------------------------------------------------------PTIAKATPELTHRFVTARGVRFHIAESGPADGTPLVLLHGFPQHWYVWRHVLPHLA-ATHRVLALDLRGCGWSEA------TEHGYSTGNLAEDVIAVLDALGIDRAAVVGHDWGGWVGFVAALRHPERIRALASVNMTHLWP------------------------------IQRRVLPNLWRMWHTAFIEHPPIGRLVLRHTGFAGFLLRH--------WSGDPTLWEREDPRIYTDLLRDPARARAvvNAAYVWREIFGHPLGRYRKARLSVPTLIIGGERDVVIPPAVLEGGERHADELAVVVLPGGHLLPEEQPSAVAEELLRFL----------------------------------------------------------    
 140 UniRef50_A7IDW4        Alpha/beta hydrolase fold n...   171     5e-41  1  13.0%  58:426     8:334     ---------------------------------------------------------LIAIGATALCLGLPLPAARSAPPAPAPGASQTVLSPTVHYRSKTIDGVDVFYREAGPADAPVLLLLHGFPSSSHMFRNLIPLLADR-YRVIAPDYPGFGHSAVPDRA---RFSYGFAAYAGLVDRLMGELHASSYALYVMDYGAPVGYRLALMHPERVTALVVQNG--NAYEEGLSPFWTPLKAYWADGAQANRDALRAGLTAEATRAQY-------------------------------------VNGVADISRIDPDTWLVDQALLDRPGVDEIMLDLFKDYASNVAlpAFHAFFRERHPPTLIVWGARDEIFPSAGAKAYLKDLPRAELHLLDSGHFALEDKGSEIAALMRDFLSRSLPVKGDRP-----------------------------------------------    
 141 UniRef50_A0B0F8        Alpha/beta hydrolase fold n...   171     6e-41  1   9.5%  83:420    11:304     ----------------------------------------------------------------------------------PAPGLPAGFDRRFSRRYAQVDDVRLHYVTGGPDDGELVVLLHGWPQTWYTWRHVMPVLAQEGYRVVAVDYRGAGESDKPLG------GYDKASMAGDIRALVRQLGATRIHLVGRDIGVMVAYAYAAQRPAEIVKLAMLDVPVPGTRIWDEAKARADPQIWHFGLHQQRDIAELLIAGKEH------------------------------------AYILDFYKKRAHVALSNDDIAVYADAYAAPGALRAGFELYRAFPQDETQFK-AFMKHKLPMPVLALAGDKSNGAKedMAKELALDVRGA--VAPNTGHWLPDENPAFLTRQLLDFFREPAP-----------------------------------------------------    
 142 UniRef50_A2QB93        Catalytic activity: an epox...   171     6e-41  1  12.9% 100:418    14:328     ---------------------------------------------------------------------------------------------------TLSDGTTYGYVSVPASEgpTFVFLHGYPSTSYDWRHQIAFLQKGGYGVIAPDLLGYGDTDKPTDLK----AYRLKVMSQHVIEILDRENVSRAVLVAHDWGVGLASRVAYYYPQRFYGFVSIAVAYNIPGpaLLKHTESIFGYPVFGYWLWHNTDEAADEMAAHPESAFSLI---------FPQDPATWKTDLAPIGKAAEWVRSGKTTPLPSWLTQEDYDIHHRVFS-KGGYAGPLSWYKAAIRGINTEdiAEGEGKLTVKYLYIAAKQDYVCRMEVGSHVTEKntDSRIEVVDAGHWVQLEKPDVVNELLVKFAEEV-------------------------------------------------------    
 143 UniRef50_C7MUA8        Predicted hydrolase or acyl...   171     6e-41  1  14.8%  97:421     3:349     ------------------------------------------------------------------------------------------------QRTVTANGQRFHYAEQG--EGPLVLLLHGFPESWHSWSHQIPMIAEAGYRAVAPDLRGYGRSSKPRRV----DDYRITELVADCVGLVEALGETEAVVVGHDWGSMLAWTAAWTRPDVFRGVVGLSVAFGGRGLLPVAGVSSLGELRPSEVHRVIAGpdMYTFFRDQFHSFSGDVYPADHQPpsPEEVLEFTRASGAVVDRGSRFRSGLVSPETLPEWLARDLDFYVAEYERTGLHHA-LNWYRCMDLDWELLAPYE-GRPIEVPAMFIGSDLDVAT-LWGAEAIANfvPRltVILERCGHWITREAPVATGEAIVRFLRTLSSA----------------------------------------------------    
 144 UniRef50_B2IGH0        Alpha/beta hydrolase fold n...   171     6e-41  1  13.2%  94:419     4:295     ---------------------------------------------------------------------------------------------PVTTRLIEANGLIFEIDECLPANGDtaLCLHGFPESKFSWKHQLPLLGTLGYRAWAPNLRGYGGSSRPKSV----GAYGLDHLVADVEGLIAASKAEETLLIGHDWGAIIAWHCAMRKIRTLSRLVIMNVPH---------------------------PTRYIEEIAVNPAQRRKSWYVLFFQLPRIPEFVLTRNKARAISGII------LKMAVDRSRFPAEVLDQYRANALIPGAMTAMLNYYRAAFRAGRKA-MAPSQNRVEVPTLIIWGEEDGALEKSLVFGTERFVSDLTVRFLPgsHWVQQEAPETVNAILRAWLTHVP------------------------------------------------------    
 145 UniRef50_Q1DFS1        Hydrolase, alpha/beta fold ...   171     7e-41  1  12.1%  47:444     5:368     ----------------------------------------------RAFTALLLAAGLSSAGCVRSYAHQPALSFQDLDYTSEGSKQPWPVKRIPLPRTANQYGmqIAYVDMPgpDAKAVVFIHGLGSYLKFWRAQLDAFQKQGYRVIAVDLPGYGKSDKP-----GTFPYTMEAMADAVLELVDGLGLDKPVLAGHSMGGQTSLSFAIRYPESLSGLVLASPAGFEKFSWREK---------------------------------------------EWFARAMSSEFIKSAPEASIWGSVRQANFMHWRPELEWLIEERVRLAKSPEFDAYAYANVRTVRGLSHNDFVRDNLHRVTVPTVIIYGTDDRLIprGIMEYGASHIPGAKLvlEGCGHTVQLDCAERFNEAAFAFVRDAAAGRIEVPSAPAKEEAPRPESPPAPA-----------------------------    
 146 UniRef50_C8VQU3        Epoxide hydrolase, putative...   171     7e-41  1  10.2%  92:415     7:312     -------------------------------------------------------------------------------------------PGFSSQYVTTAHGARIFVRVSPTQDKPPLLLVHGFPQTHAEWHKLTPLLTP-HFTVVLVDLRGYGASSIPASANGS--GYTKRLMGQDCLSVMDQLGyqRFAVVGHDRGARVAYRLAFDNPERLSKVVVVDIVPTAAM--------------FARFGNPTAGLKAYHW----------LFLAQPEPFPEKMIGKEDKGRL-----FLEQALSSWTAAGTLQAFSETAMERYREAYCDEQRIHATCEDYRAGAYFDRVYDEEDLKkkIRVPVLAVWGEEGGFTGPKKSEawQRYCVDLRGKGLNCGHFIPEEDPQALADEILQFL----------------------------------------------------------    
 147 UniRef50_UPI0001793373 PREDICTED: similar to epoxi...   171     7e-41  1  13.5%  96:437   108:419     -----------------------------------------------------------------------------------------------THSYVKVKGVKFHYVECGDPKGMIVILLHGFPSCWISWHHQIPTLS-KHFRVIAVDLKGFGDSDKPSARKS----YRVENLVNELAVFLSMLGVdkCHVIGHDLGALLGWYLVHLWPNCVSKFVAISCPHPNVHWEYLPPSSFFNKNW-----------------------------ICFSQLPYLPEMDALQSDLKIINQCYQHL------SKNKENDDLSYIDAYKYTFSRTEDWTGAINYFRNLPFYRIEPrlNKEEDMLQVPCLLITGSKDVSVQMESFIKSTEFLKTSTVRIIdaSHFPHQEQPKVVNDLLTSYLVRVKVVQKeqPRQNSGGIVGRM------------------------------------    
 148 UniRef50_A7IDW3        Alpha/beta hydrolase fold n...   171     8e-41  1  12.4%  83:422     8:309     ----------------------------------------------------------------------------------AVRPAAAGSTPVAtyRRAAVGGLDIFYRDAGPADAPVIVLLHGFPSSSHMFRNLIPALADR-YRVIAPDYPGFGYSSAPA---PEDFAYSFDALADVVETLLADLGVRRYALYLQDFGGPVGMRIAARHPERVTGLIVQNSV-------------------------------------ANLEGFAASAVGTFGPYWADRNAETEKPLRGFLTAETTRFQYEHGASARADRVSPDAWQHDQWLLDRPGNDRIQLelYRYQDNVGAYPQWQAYLRAHQPNILVTWGDNDPFFTAAGRDLFKALVPATEVHAYDAGHFALETHGPEIAAAIRAFLARVAPAA---------------------------------------------------    
 149 UniRef50_Q2NCH7        Alpha/beta hydrolase fold n...   170     9e-41  1   9.5%  98:424     5:303     -------------------------------------------------------------------------------------------------RVTLENRIELDVVDTGPRDGPALIFLHGFPESHRTWRRQIAHFSDR-YRCIAPDQRGYHGSSKPEGV----ENYTPDKLIGDVFLLADALAVERFTIVGHDWGGALAWGVAMrqQNRVERAILCN--------------------------------------AAHPLIYSKLLWTDAQQRAAGQYMRVYRDTSRDDEIRERGLLALLeaTGALPDDDSEERAAIFEDWSDGKAAIGMLNWYRATPMAVPPADAppKVPKLTIPTLVVWAMDDHALSPGNIERLEDHVEDLTLviEDCGHFLLWQEPEQVNAAMESFLATHLQPRSP-------------------------------------------------    
 150 UniRef50_D2LBT3        Alpha/beta hydrolase fold p...   170     9e-41  1  15.4%  81:418    20:310     --------------------------------------------------------------------------------PVMVAQQGTAAAAVSYKTVRTPAGlrLAVTESGR--GKPILLLHGFATSSYTWQGVMPDLA-RKHRVIAVDLRGFGASDKP-----LDDKYSVFDQADVIQAFIEQENLKDLTIVGHSFGGGVTLALALrlRSRIRNIVLVDSVAYKQP------------LPIFFRMLQVPGLAEVGMALVPPEVQSEQGLK--------------------------------LAYYDHEKITERSITEYASPLRSSAAKHALVKTVEQIM-PPNIDEIALSYSTIRVPTLVVWCDEDKVVPSVFGQRLKADIPTAELVMfkCGHMPQEEKPAETARAIESFLARH-------------------------------------------------------    
 151 UniRef50_A7HSU0        Alpha/beta hydrolase fold n...   170     9e-41  1  12.9%  47:434     3:339     ----------------------------------------------KRLFAGTLTLVVLALAVIVISVLRFDISREELA---------GKYATGASQFVTLPGGGSMHVRDEGNREGPALLLVHGSNASLHTWEPWVASL-GDTYRIVSMDLPGHGLTGRIPG-----DDYSREGMTQSVHELTEILGIDRFAIAGNSMGGGVAALYALEHPAQVSALILVDAAGVPVPRNDDEVPLAFRIARMPVISNVMRYVLPRSLVEEGVRKV----------------------------------------FVDQSKVTDEMVARYFDLSLHEGNRDAT-RIRFASYAGRDEEAFAARLGGIEMPTLVLWGDKDGLIPVSAAHEFKARIPQAELAIFevGHVPMEEVPEESAAVVRDflAETSPSAGGMAAAGVMAEE---------------------------------------    
 152 UniRef50_UPI000192564B PREDICTED: similar to Abhyd...   170     1e-40  1  11.9%  39:417    10:341     --------------------------------------KRLSYYCLVLACSAMYIFSYFVNLVRLFVNGKRCITKNRASPPSCLVK-----PELGVHGYLTlnDLKIHYVAKGKPGNPLVLFLHGFPEFWYSWRFQLQDLSDDLY-VIAVDMTGYGDSSKP----QNLDRYHVNEIAEDMKEVILELGYNSCILVGHDWGGVVSFSVAHNFPEVVNKLVVVNVPHSRS------------------------------FKKASFKQLWCFTYIYFFQLPFIPEWILSSNDFS--------LLNYIKSMANSNNFTASDLEAYKYCF-QKGGFTFPLNYYRNLYNQVYTS--RGPFKKIVPPTLLIWGVNDPICLLESGRLSLEALENGKIeiENASHAPHYDQPKVFNSTLMKFISS--------------------------------------------------------    
 153 UniRef50_Q07MT0        Alpha/beta hydrolase fold n...   170     1e-40  1  12.1%  96:417     4:282     -----------------------------------------------------------------------------------------------RSRRLIANGIDFAIDEAGEGD-TVALLLHGFPESREAWHRQMPALAQLGWRVVAPDLRGYGETSRPSERS----AYRIEHLTGDVAALFDQLGGRRNVLVGHDWGGVIAWQSAMQGRVKLDGLVILNAPH---------------------------LEVYRRALRSNWRQALRSWYVLFFQLPWLPERLMTAGQGAALVRGLQR---------QSPNFPPEVLEIYRRNVIQPGAATAMLNYYRANLAQFGGAGGSPV---ITTPTLVIWGENDVALDVSLTERLDEFVADLTLHRLPgsHWVEHDAADRVNELISVWARS--------------------------------------------------------    
 154 UniRef50_Q1JYN8        Alpha/beta hydrolase fold n...   169     1e-40  1  11.0% 100:418     3:264     ---------------------------------------------------------------------------------------------------AQINDTEIFYTESGDPTKTSVVFIHGFPFSHAIWQKQIKALGDD-FHCIAYDFRGMGES------CVGDGQYSLEGHVDDLVALLDFLQIDQAVIVGLSMGGYIALRALQRNPERFLAVALCDT--------RSEEDDNAARIKRANAAQSVKKEGAAAFAEGFLPAVFSEASITNNVPGVGMIKQIISKNAPLAIAG-------------------------------------------NLIAMAARTDTTASLKDIAVPTLILVGEKDKLTTPEDARNLQNQIKGSVlvVPDAAHLSNLENPEFFNARLLEFLHST-------------------------------------------------------    
 155 UniRef50_B4SG63        Alpha/beta hydrolase fold n...   169     2e-40  1  13.5%  92:418    19:301     -------------------------------------------------------------------------------------------QTSPEKRFIEVNGFKVHYRMAGSG-KPLVVLLHGSFLSLRSWRLVFDELAKTT-TVLAFDRPAFGYTSRPLPSKATGVSYTPEAQSDLVIALIKKLGFSKAVLVGNSTGGTLALLTALRHPQHVEGVVL------------------------------AGAMIYSGYATSEVPAFMKPVMKAMTPVFSRLMKFLITRLYDRNIRGFWH---------NKERLGDAVLAEFRSDLMHGNWSRAFWELFLETHHL----QLEKRLKTMSLPSLVITGEHDLTVKTEESIRLARELPCAElvVPDCGHLPHEEQPEAFLVAVRKFLKRV-------------------------------------------------------    
 156 UniRef50_Q0HH49        Alpha/beta hydrolase fold n...   169     2e-40  1  12.0%  94:444    26:339     ---------------------------------------------------------------------------------------------PFKRHFLSRNGNKLHYINEG--QGEPVVMVHGNPSWSFYYRNLVSAL-KDTHQCIVPDHIGCGLSDKPDD---SGYDYTLKNRIDDLEALLDSLKveNITLVVHDWGGMIGMGYAARYPERIKRLVILNTGAFHLPDTKPLPLA---------------------------------------------LWICRNTLLGTVLVRGFNAFSSIASYVgrQPMSKYIREAYVAPFNSWANRISTLRFVQDIPLKPGDrdIAASLPKfkVPTLICWGLQDFVFDKHFLAKWREHMPHAQVHEfdCGHYILEDASDEVITHIKHFMAEaePEVQQVETESVTEPEVETQAPQAAH-----------------------------    
 157 UniRef50_B0WIU7        Epoxide hydrolase n=3 Tax=C...   169     3e-40  1  11.1%  27:439     3:384     --------------------------KSNQYDILRIVPKRQ---IFKIYLRCLVA--NLILLIKWLFESVKNRVNQTTIIRRAIERTEFPNRQpgRHSYVKLENTKLHYVEAGNRSNSTLLLLHGFPDCWLGWRYQISELS-QYFHVVALDLKGFNDSDKP----MLRFEYTPKKVCEDLRKFLIAISAKSVSIVGHDLGAIIGWLFAHTNPEMVDKFISVATPHPNLMWDNLPKNSPFNRRWLEFIQLPYLPEMEMKY---NAERMLKKCYPH-------------------------ILKEKMYMTGSNGSVINNLMEAYKYIFSQTDDWRGPLNYYRNFLFFRVKE-----GETVQCPCLIITGNDDRFYKLESVVKSSDFCENFIivIEQSGHAPHQEMSTEFNSLLLKFlrMLVRSPENAATTPTRGIVGRVFG----------------------------------    
 158 UniRef50_A6RVD3        Epoxide hydrolase n=3 Tax=S...   169     3e-40  1  10.6% 100:421    17:332     ---------------------------------------------------------------------------------------------------ADLNGRTYSYILGEPQNGatIFLIHGWPDMAFGWRYQIPALQALNFRVVVPNMQGY----SSSSSPQELSAFTYKTAADDVAALAKAIGATSIVLGGHDWGGAIVYRIALHYPKLISAVFSVCTPFFPPALKYTPMT---VLPNFKYQLQLKGPDVEREIQGKEKLRLMLNALYGGRSAEKELGFN----------VTEGVLFQNLEKLGPSPLLSTEELDHYAEQYAING-MRGPLNWYRTGELNFEDeeLAEEFhkVEIPTMFIGGSRDAALPPSMAENMQQWFGEGKLreVDASHWALWEKPEEVNAYIKDFLTKVVGG----------------------------------------------------    
 159 UniRef50_O52866        Soluble epoxide hydrolase n...   169     3e-40  1  12.3%  96:419     6:286     -----------------------------------------------------------------------------------------------THHQAMINGYRMHYVTAGS--GYPLVLLHGWPQSWYEWRNVIPALAEQ-FTVIAPDLRGLGDSEKP------MTGFDKRTMATDVRELVSHLGYDKVGVIGHDWGGSVAFYFAYDNRDLVERLFILDMIP-GLIKAGDSFPIPVALMINHIFFHGGNPDWATALISKDVNLYLRRFLTT------------------------------LDYNYSPNVFSEEDIAEYVRVNSLPGSIRSGCQWY-ATGLREDTENLAKATDKLTIPVIAWGGSHFLGDIRPAWQEVAENVEGG--AVENCGHFVPEEKPQFVIDTALKFFAPLR------------------------------------------------------    
 160 UniRef50_B8FK90        Alpha/beta hydrolase fold p...   169     3e-40  1  12.8%  46:421     1:321     ---------------------------------------------MKFFRLMLVLPLLFSMYGCSFKYNPVDYNTSII-------QNEIETAGIGKSSFIEIDNLNMHFIESGEGD--PIILIHGWLCWGAFWKKITPSLSEK-FHVYALDLIGHGLSDKP---VDDNFSYSTEAQARRVVEFMKKKSITNAVIVGHSMGGEIAAKTAIMAPDRVSAAVLICAAGM----------------------------------QDNPQSLPSYIRIARAMHLEPIIALFFSEPAIRQFTKD-------LMFYSENPMPEEFVKDVV--LANLTGKNAKKAVYKVTVEGLFKDFLNERCAEMKTKTLVISATDDLIVPPAMGREYNSLLPDSTYLEFdaGHMLPWERPEDVSRAILAFCGRVDQA----------------------------------------------------    
 161 UniRef50_A8GAF4        Alpha/beta hydrolase fold n...   168     4e-40  1  11.0% 102:418    28:294     -----------------------------------------------------------------------------------------------------VNGHRIATGIHGTGN--PLVLVHGTPAHSIIWRDLLPSLTAAGFRVHIYDLLGYGASERP-----LSADTSIAAQADLLIDLLDHWQLESTHVFGHDIGGALSLRAAFGHSRRFRSLTIADICSYDSWPSPSWRGIRDNYHQYAVMDASQ-----------HEQTLARQLKMAVF---------------------------------NKSRMSGELLQHYLAPIVGVVGQPAFYQHQIAHYDARYTEDFAQRLPELRLPVQILWGENDEWQPVSYAYRLQGDIPGARlvIPQAGHFLMEDAPETVAQRLVTFIHSL-------------------------------------------------------    
 162 UniRef50_A7RNK1        Predicted protein (Fragment...   167     5e-40  1  12.1% 128:417     1:255     -------------------------------------------------------------------------------------------------------------------------------QFWYSWRYQLQWLSE-NYRVVAMDMRGYGESDHPKGR----GEYVMTKLTQDVREVMSGLGFSSCILACHDWGGFIGWTFAHQFPDMVERLIIVNCPH---------------------------PMAAEKYVFTHPSQLLRSWYVYFFQLPYLPEFWIALNNYE---VFEDMFRGEQMGVRKPGSLTDEDLAAYRYMFSREGALTPPLNYYRNVLVRTKSDRDLFK-ARFRKPVLVIWGTGDRALESGMLEGTADFAPNLTIRYVdaSHWVQQEEPEVVNECIHDFLTA--------------------------------------------------------    
 163 UniRef50_D2BB50        Alpha/beta hydrolase fold p...   167     6e-40  1  12.3%  96:421    17:294     -----------------------------------------------------------------------------------------------AHRSVDVDGVRVFYREAGPRDAPTLLLLHGFPSASHQFARLIDALGTR-YHLVAPDYPGFGHSEAPAG-----FGYSFDRLADVVEGFVDRLGLDRFVLYAFDFGAPVGFRLAVRRPEWIAGLVVQNGNVYEE-------------------GLSEAARGFVALGPGEEEQVLPLLTLEATRGQYEGGASDPAAIAPDGWTLDQHFLDL------------------------PGRKEIQIDLAFDYKSNVAlpEWQAWLREHRPPTLIVWGRNDPFFVEEGARAFLKDVPGAELHLFDTGHFALEEKLPQIAPLIADFLERAGEG----------------------------------------------------    
 164 UniRef50_D1ZTT1        Whole genome shotgun sequen...   167     6e-40  1   9.7%  78:418     1:381     -----------------------------------------------------------------------------MSSTPDFPPKLVPNDPRVLSKTTTINGKKYHYLIsgGGDAPpvLLVHGWPDLSFGWRYQVPFLLSLGLRVIVPDLPGFGRSDSPQELT----AYSYKNVTRDLLEllSEVPGGkiIVGGHDWGGAVAWRFALWYPELTRAVLSVCTPFWApaGGFVPLKQMVDTVLPNFRYQLQLSGPEFEAQVKGKEKIRQFLNGVYGGKGPNGEPVFTT--------EKGCLFENLPKMGKTPLLDDREMEYYVKEVadKGSMRGGVNWYRTTQVNYEEE--RELLKqvEPPALFIQATRDTALPPAMSEGMERYFekLMKREVDATHWALWEQPVKVNQAIAEFLGGV-------------------------------------------------------    
 165 UniRef50_A4XVU4        Alpha/beta hydrolase fold n...   167     7e-40  1  10.8%  97:420    42:309     ------------------------------------------------------------------------------------------------HQQIRVRDLNIHYYRGGPADGETLVLIHGFAADKDNWLRFSRHLTDR-YQVIALDLPGFGDSDRPAG------SYDVGTQAERLASILEALGIERAHLLGNSMGGHIAALYAARYPQRTQSLALFDNAGVEAPIKSEFFTLLERGQANPLVVHQ----------AEDFQRLLQFVFVEPPYLPESLKRHLAERSMANREHYEQVF--------------QQLVERYI--------------------------PLEPELPKIEAPTLLLWGEQDRVLHVSSIEVMRPLLRHSSvvMPGVGHAPMLERPQESALLYRRFLQGLTP-----------------------------------------------------    
 166 UniRef50_Q9ZER0        Haloalkane dehalogenase n=7...   167     8e-40  1  15.2%  93:415     8:290     --------------------------------------------------------------------------------------------FPFDPHYVEVLGERMHYVDVGPRDGTPVLFLHGNPTSSYLWRNIIPHVAP-SHRCIAPDLIGMGKSDKP------DLDYFFDDHVRYLDAFIEALGLEEVVLVIHDWGSALGFHWAKRNPERVKGIACMEFIRPIPTWDEWP-----------------------EFARETFQAFR------------------TADVGRELIIDQNAFIEGALPKFVVRPLTEVEMDHYREPFLKPVDREPLWRFPNELPIAGEPANIVAllHQSPVPKLLFWGTPGVLISPAEAARLAESLPNCKTVDIGPghFLQEDNPDLIGSEIARWL----------------------------------------------------------    
 167 UniRef50_A3JB27        Alpha/beta hydrolase n=1 Ta...   167     9e-40  1  11.6%  90:417     6:293     -----------------------------------------------------------------------------------------PADFPYPSHFADVLGSRMHYVEHGNGD--PLLFLHGQPTWSYLWRKVLPELEGKG-RLIAVDLIGYGMSDKPDIP------YDIDDHIRYLDGFIEALGLDRITIVCHDWGSFFGFHYAHRHPERIKGLAFMEAMLNPIPGYDA---------------------------------------FDPQTRAFFQTLRSSQANAERMMMDENQFVENILPAMICRPLERQELDAYRAPWTDRQSRRILCTFPQNLCIGKEPASVyeWLGQTDLPKLLIHAEPGFLIPAPAVDQYRQQLPNLETAFVGSghYIQEDQPQKIGQAIAQWMDR--------------------------------------------------------    
 168 UniRef50_B3EC74        Alpha/beta hydrolase fold n...   167     9e-40  1  14.6%  99:421    18:296     --------------------------------------------------------------------------------------------------VIDIDGLQVHCLTEG-IGKPVLFFLHGSFLSVRSWRFVFERLSER-YTVIAIDRPAFGRTDRPVPVVGKFNPYSPEGQADLVVAILEKLGHRQAVLVGNSTGGTIALLTALRYPDKISGLVLADPMVYSG-----------------YATSEFPAWLYPVFKAATPAGAQLSKLMIGLVFNKLHRTFRHEN----------------------SRPGNDLLTAYRRDLMQGRWGRAFWELLLSSHNL----DLEAQLGRINVPALVITGAQDRMVKPDETIRLSRALSRALliIPDCGHLPQEEKPDIFIAAVSEFLDKHIGA----------------------------------------------------    
 169 UniRef50_B2VVR4        Soluble epoxide hydrolase n...   167     9e-40  1  13.0%  81:419     5:307     --------------------------------------------------------------------------------PKSCDADDWQQRIASITTY-SGEEIRLNYIYSPdgNKpvILLIHGFPQTSYQFRHVITEFAKAGYRVVAPDYRGAGNSSKP------LTGYHKTQMAEDLCILIkhLKisKIHIVGHDIGGMIAFAYASRYPDDVASVVWGECP--------------------------LPGTSFYESVKGSPE----LFHFVFHQVPDLPEFLI----AGKEREYLKHFFDKLQYNSL--AITPADLDHYALAYSQPGAIRAGLELYRAFedAEENRTWLARHGKVKVPTLLLMGEK--CLLAQAAKSMaeYHETAEIVMinAAHWVAEEDPEAFIARILGFIEKLP------------------------------------------------------    
 170 UniRef50_Q2SJ56        Predicted Hydrolase or acyl...   167     1e-39  1  11.9%  46:439     5:345     ---------------------------------------------LLLSFCLLFVVGLSGCAGLKPSLKASPTLANALGVADIPLKELKKQYADKDSRFMEVDDLQIHYRDVG--EGPTIVLLHGIMSSLHTWEGWIEEL-RKNYRVIALDLPGYGLTGGPEDADDFDEDYVYTRFSK----FIRRLELTRFSLAGNSFGGYLSARYAAEHPEQVEKLILVDPVGYPQEHTPKVFDLATMPVVGTLANYVQPP----FLVTRNVEQV----------------------------------------YGDPKRISQDNLYRYVHMSQRPGARKIYVRTMRIMKEAAAEQRNLP-FADIRSPTLLMWGEADRWVPIKLAERWRGDVRNIKFiyPDVGHVPMEeaYQTVQDAIV-FLSDL--QQAPTRSEPSIEELESILQ----------------------------------    
 171 UniRef50_A6G7B3        Putative uncharacterized pr...   167     1e-39  1   9.4% 114:420   492:765     -----------------------------------------------------------------------------------------------------------------SRERAAVLCLHGFPDNARSFRHQLPALAAAGYRVYAPTLRGYEPSSQPE-----DGDYSLAALAGDVLAWLDELGLERAHLIGHDWGAAVTYTVGALAPERFWSLTTLAVPHPAQFVRGVARLPRQVLNSWYMLFFQVPGAPELALRRRDWALIRQLWAAWSPG----------------------------------YTLPPEEWAALRETFEAPGVDRAMLAYYRRNLVPTFVDGLRgdEAVIRVPTLALTGADDGCIDTRLYEGvaREFpgFRlrVEGAGHFLHQERPEAVNALVLDWLAEHDP-----------------------------------------------------    
 172 UniRef50_Q7RZL5        Predicted protein n=1 Tax=N...   167     1e-39  1  11.0%  74:418     2:364     -------------------------------------------------------------------------AASTADFPPKLTPHDPRV----VSKTATINGKKYHYLFspPSDGstVLLIHGWPDLSFGWRYQVPFLVSLGLRVIVPDLPGFGRSESPAELT----AYSFKSVTHDLLELVESEVpgGIIVGGHDWGGAVAWRFALWFPDVTRAVFSVCTPFWApaGEFVPLKQKVETVLPNFRYQLQLSGPEFEAQVKGEEKIRQFLSGMYGAKGPDGERVFTT--------EKGCLFENLPKMGKSPLLDEQEMDYYVKEVadGGSMRGGLNWYRTSEVNY--EDERELLKqvEPPALFVLATRDAALPPAMAQGMERFFdkLMRREVEASHWALWEQPVKVNQAIAEFLGGV-------------------------------------------------------    
 173 UniRef50_C9SVI2        Epoxide hydrolase n=1 Tax=V...   166     1e-39  1   8.4%  96:417    14:330     -----------------------------------------------------------------------------------------------ERKEAKVRGKTYSYLHGKPEGGEtvLLVHGFPDFSFGWRYQIPQLQAMGYEVIAPDTLGYGHTDAPDGI----EPYTQKEMAHDLAALAEalGppRVVLGGHDWGGSLVWRTAMYHLELVVGVFSICTPYFPPNPRYFSLESLVNSGRLPTFRYQLQFISGDVEavKTTDDIRQTLVSIYGGRTPDGQFGFS--------AEKGFKFDLFGQVGSSPHVSKEEEDHYVENFARNG-LHGPLNWYRTRELAFADepFVEKGWRFEVPCFFASATKDLALTPEMAKGMGAHFDDLTTKSVDAGHWALWQRADEINAMLKAWFAE--------------------------------------------------------    
 174 UniRef50_UPI0001B4C886 alpha/beta hydrolase n=1 Ta...   166     1e-39  1  13.9% 102:417    10:277     -----------------------------------------------------------------------------------------------------VDGVRIAYRDRG--EGEPVVFVHGTPSYSYEWRNVVPHVEAVGHRAITYDLLGYGRSERP-----VDRDTSVAAQTDLLGGLLDALGIEQATIVAHDIGGAIGQRFAIAHPERVRRLVIIDTVSYDSWPSETWRKLIEEQVEEHMQLPQ------------------------------------------EAFDALLTRQLEMTVADGAATMTGDILDAYLAPHRSALGRVSFFEHQVRHYDSRYTEEITDQLASLTMPVRILWGERDQWQPLTYARQLAEGIPGARlvIPGAGHFVMEDAPERVTEEILDFLAD--------------------------------------------------------    
 175 UniRef50_A0YHI4        Putative epoxide hydrolase ...   166     1e-39  1  10.3%  99:418    62:341     --------------------------------------------------------------------------------------------------RIAANGYQFRARVAgaADKPAVILLHGFPVSSAMWVRLIGPLVESGYRVVAFDQRGY----SPGARPTELRDYRIDELSSDVVAVADALGVETFHLIGHDWGAAVGWSTVMQYPQRIESWTALSIAH---------------------------PMAFSEALANDRDQQSRSGYFALFVTPWLPEALFSFNNFQMLGAAYSGML-------------DEQKSEYVRLFSEPGALTAALNWYRAMTIRHQGAEQSS--PEVSIPTLFMWGNDDPTAGRLAVDMQKQYMTgyQKVELDAGHWLLTDQPRAVVRAVIDHLGTH-------------------------------------------------------    
 176 UniRef50_A4AHZ3        Putative hydrolase n=2 Tax=...   166     1e-39  1  11.9%  97:416    14:292     ------------------------------------------------------------------------------------------------HGRALVNGVRLHYMTAG--QGEPLFLLHGVPKTSYHWRHVIPLLTP-HYTVIAPDLRGLGDSEHPQG------GFDMANMAKDIADLATHLGYDTFNLVGEDWGASTAYQVAAQWPERVKKLV-----------------------YQEMILPGFGLEDYSFLTRENVSTFVWLWHINFYSVPDFPEFLIT----GREKEYFNYFIKH--ETHNPQAITPDALDEYVRCYSSPGGLRSMFNIYRATLDDAD-QNHQSAKTKLKMPVLAVGSQY--FIGKDNERQMRQVAENVEAAILPWGHQLAEESPEELAAIYLNFFQ---------------------------------------------------------    
 177 UniRef50_A1DK35        Alpha/beta hydrolase n=1 Ta...   166     2e-39  1  13.4% 106:419     2:271     ---------------------------------------------------------------------------------------------------------RIAYGIYGEQTAEPVVLLHGTPSSSLIWRNVLPHLTSANYRVYVFDWLGYGLSERP---WDPSIDTSISGQVPILEDLLAHWGLESAHVVAHDIGGGIAQRFAIFSPQRIRSLTLVD--------------------------------------------VVSFDSYPSARTKKQMEDRLEALIRAPEEKHREHYRDWLLSAVHKrqFESSTLDTFLGYISGPVGQASLYQHQVRFYNPKHTLEIADRLGElkLPVKLIWGANDGWQVPDWAHKLHEAIPGSQLEIidCGHFSPEDQPEQLAELLVTFLKEHS------------------------------------------------------    
 178 UniRef50_Q2SAV4        Predicted Hydrolase or acyl...   166     2e-39  1  14.4%  85:431     3:299     ------------------------------------------------------------------------------------DQWINREEYPFASHYIELGPGRMHYVDEG--QGKPIVMLHGNPTWSFLYRNMIKGLSGQ-FRCIAPDHLGFGLSDKP-----GDWSYLPQDHARNLEILIKKLNLQDITLVVQDWGGPIGLSYALNHPQNVRRLVIMNT----------WMWSAHGDVKYETFSTLMGGLFG-----------------------------------RFMIKRFNVFVSVLLKQAVQKKLSADIYRHYSEPLKNPAERKGCWVFPKQiaDEWLEELWEKRANIADIPTLLLWGMKDFAFQERELRTWMQLLNHHTAVTfdAGHFLPEDKGVEVCPLIADFLQDAEPTFQREASAPAP------------------------------------------    
 179 UniRef50_B4WEL7        Hydrolase, alpha/beta fold ...   166     2e-39  1  11.6% 116:408    24:313     -------------------------------------------------------------------------------------------------------------------NAPLVLLIHGFPELGISWRAQVEALSAAGYHVVAPDMRGYGGTDKPKGV----DACSILHLVGDMVDLVRALGKQSAVVVGHDWGAPVAWHCALLRPDLFTAVAGLSVPFQppPTTVMAILSERAGLGDLYISRFQAADAHLALEADPATTLRKLFWSYDGATP------ASKRATGFMARGVGLLDSIDDGADLPPWMTPAHFTEYVEAF-TAGGFDAPLNWYRAIDLNWSLTAFAQE-QRILQPALFIVGEDDPVRHYAGSAelKDWVPNLtvVLPGAGHWIQQERPDEVT-----------------------------------------------------------------    
 180 UniRef50_A5D9Y1        Putative uncharacterized pr...   166     2e-39  1  11.3% 103:403    14:274     ------------------------------------------------------------------------------------------------------DGTNVFYREAGSSDKPVILLLHGFPSSSHMFRDLIPVLAP-HFRVLAPDLPGFGYTE-----TSTSYKVTFAAIADTIDQFLSKLKINKFYVYIFDYGAPTGFRLALKHPERVSGIVTQNGNAYVEGIENDFWQPIREYWKK---------------SKDDPTFFEALSKFIEDPANVTVQYTEGEP--------------------NASGIDPSSWTLDQALLARPGQTKLQVELFHDYQSNVElpKFQEYLRTSNVPVLVIWGKNDKIFPPVGAEAYKRDVKNLEFHYVDGGHFALESH----------------------------------------------------------------------    
 181 UniRef50_Q88EC6        Hydrolase, alpha/beta fold ...   166     2e-39  1  11.5%  99:421     3:263     --------------------------------------------------------------------------------------------------YFEHEGCTLHYEEYG--QGEPLVLLHGLGSSCQDWELQVPLFS-RHYRVILMDIRGHGRSDKPAD------GYQIATFSADLLALLEHLHTGPVHFVGLSMGGMVGFQFAVDHPQWLRSLCIVNSAPEVKRRTRSDWAWWLKRWSLARILSVETVGKGLAERLFPKPQQADL--------RQKMAQRWARNDKRAYLKSFDAIVDWG---------------------------------------------VQERIGQIHCPTLVIAADHD-YTPIQLKERYVALIPNARLVVvdSRHATPLDQPEVFNQTLLQFLAAASTS----------------------------------------------------    
 182 UniRef50_C7ZBR8        Putative uncharacterized pr...   165     3e-39  1  10.4%  99:416    17:329     --------------------------------------------------------------------------------------------------TASIRGKTYHYLRADPETEptIFLIHGFPDLGFGWRYQIPHFASLGYQVVAPDMLGFGDTDAPDQPS----QYALKSIAEDIKELANAIVKdrIILGGHDWGGAVVWRTAMWFPSLVQGVFSIGTPFTPPSSVFLSPDDAARSKgiNSRYQSQFRGTEIEDKIRDEERVRQFLNAMFRGTSPEGEVGFSV--------TEGVLFGNLPKLGQSRLISGDDLDHYTSKYFRKGelKGPLNWYRTRAHNY-FDELQLLLKPIkmPALFLAATRDEESPPSMSDGMDQYFEDLTRGEVEASHWALWESPAEVNKQVSAWLE---------------------------------------------------------    
 183 UniRef50_D2QYX7        Alpha/beta hydrolase fold p...   165     3e-39  1  11.6% 100:417    22:299     ---------------------------------------------------------------------------------------------------VQLRNAELEIASAGDPTAPLVVLLHGFPESYLGWRHQIVPLAAAGFRVVAPNQRGYAGSSKPPHV----RDYRLDHLAGDVADLIHQQGRSQAIVVGHDWGAAVAWYLAATIPAVVAKLAILNVPH---------------------------PLVMRKVLRTSLRQFFKSWYMLVFQLPWLPELMARRHQFAPLRKALTESSRA-------GTFTDAELAAYAELWSIPGAYTTMLHWYRAAFRYGQET---AMPPRITPATLMLWGCRDQFLAREMAQPSIDCCDQGTLhfEEATHWIQHEEPHRVNELLLEFFRR--------------------------------------------------------    
 184 UniRef50_D2QIH9        Alpha/beta hydrolase fold p...   165     3e-39  1  11.2%  95:416    27:311     ----------------------------------------------------------------------------------------------FTNQYATVNGVRLHYVEGGT--GIPLVCLPGWPQTWYSYKPVAVELA-KTYRVIIVDIRGMGSSEKPQS------GYDKKTMAADIAALLQQLGLTNVHLMGHDIGGMVAMSFAFNYPQFTQKLIVLDGSHPSEGLMQMSLIPPRG-------------------------TFAGKMDADRPYAWWMGFNQVKELPEKILAGRFQYLLDWLFQYvdDSKMSPLERAVYASSYNDAESIRAANAWYQTFQQDIDDAQTYQPLA---MPVLGI-GSYVSYNYMKMGLPYVAK--DAQVviLDSGHYLFEERPAQVLDAVLRFLA---------------------------------------------------------    
 185 UniRef50_A9GSN5        Family membership n=1 Tax=S...   165     3e-39  1  15.4% 107:417     1:261     ----------------------------------------------------------------------------------------------------------MRVLVAGAGNGPALLLIHSFLVSHLEFDDVIDTLAQR-FHVIAPDLPGFGESEKP---SPARYAYGIETFAEAVADLIAAFGVGRAHLVGHAMGAAVAITLAANHPELVQRLVLEDALCYPFPMSFKMKLPLLPVVGGIVFKQLHGRGSFRSYFRDDVFRADA-------------------------------------------AVPLSRVDRHYDLFNAPSARESAHAVLRAV---LDARPVVARLTRITAPTLVVWGRDDRIFPAASAQRLAREISGAMLEIMDAGHSPHEERPGEFVALVTQFLEG--------------------------------------------------------    
 186 UniRef50_Q48DA3        Arylesterase n=13 Tax=Bacte...   165     3e-39  1   9.9%  86:417    78:359     -------------------------------------------------------------------------------------PINPTSRGICMSTFNAKDGTEIYYKDWG--EGKPVLFSHGWPLDADMWEYQMEYLSSRGYRTIAFDRRGFGRSSQP------WTGYDYDTFADDIAELIEHLDLHDVTLVGFSMGGgvTRYIAKYGSERVAKLALLGSVT-----------------------------PFFLKTDDNPEGVDKSVFDGIKEGLLKDRAQFISDFATP------------FYGLNKGQKVSEGVQAQTLNIALLASLKGTLDCVTAFSA----TDFRADMAKIDVPTLVIHGDGDQVVPFEagKRAAELIKGAElvYPGAPHGFAVTHAEALNKDLLTFVQG--------------------------------------------------------    
 187 UniRef50_Q2KHF5        Putative uncharacterized pr...   165     4e-39  1  12.7%  89:418    27:347     ----------------------------------------------------------------------------------------PAGVDAMRHSATVASGNTYSYLlaGTPKG-TIFLLHGFPDLSYGWRYQMPALTQLGYQVVAPDMLGYGRTSAPKDL----GAYTFKKMTDDLAGLAKQIAPgkIILGGHDWGAAMVYRVALWNPDLVKGLIAVTTPYSAPTAQYVDVADAVKAGLTNFGYQVAMRDPALDAKLQTRDQIRQMLLAFYGAQTPQGQPGFT--------AEKGLIFENLPTLGSTPLLSAADLDYYVNEYAR-NTVAAPLHWYRTAKLNwdEQSLVAAGGKIKVPTLFITATQDTALPASLSVGMEKYFDKLTRGTVDSSHWALWQGAKNVTTQIATFVASV-------------------------------------------------------    
 188 UniRef50_B1MKU6        Possible epoxide hydrolase ...   164     5e-39  1  11.1%  96:420    13:290     -----------------------------------------------------------------------------------------------EHQYVQAGDVKIHVAVAGPQDGRAVVLLHGWPENWWMWHKVIPALAAAGYRVHAMDLRGAGWSEVAPA------GYEKEQFASDVLAAADALGLESFDLVGHDWGGWTAQLVALKAQSRIRRLVVLNIPPVW----------------------------------QQPGRVARYAHKLA----------YQLVVGAPVVGPLSHLSPTLWWFIRRSGMPKESVDFFRSCFRDRDRRVAGSKIYRSMVGKEFANLARgdDQKLTMPVRVLFGLDDVAVHPSLLDGFKDHADDLNieVPNCGHFIVDEQPELVVKWLSEVLAEPAP-----------------------------------------------------    
 189 UniRef50_B3E601        Alpha/beta hydrolase fold n...   164     5e-39  1   8.7% 102:422     8:270     -----------------------------------------------------------------------------------------------------INGNMIAYDDVGT--GPAVMLIHGFPLNRSMWRPQLGDLVAAGYRVITSDLRGFGESDAPDGT------YSMDLFSDDLISLLDHLEIEQAVAAGMSMGGYVLFNLLARYPERISGAVFVVT---------------------------------------------------RSVADDEAGRARRLQLATELLTFGPQVVADSFHPLMFAPGTVEARPKLAEEVYDWMVANASRGLAGGLLAMRERPDVTPLLPSITTPSLVIAAEQDKACPLEHPHMIANNISASQlvIADAGHLVNLEQPNGFNHCLLEFLRKVAPTA---------------------------------------------------    
 190 UniRef50_A1T968        Alpha/beta hydrolase fold n...   164     5e-39  1  15.4%  99:430     7:295     --------------------------------------------------------------------------------------------------YLDLHGDRVAYREAG--QGPAVLLIHGMGGSSLTWKALLPHLATR-YRVIAPDLLGHGQSDKPRG------DYSLGAFAVWLRDLLDLLGIARVTLVGHSLGGGVAMQFVHQHPDYCERLVLISSGGLG------------------------------------PELGRSLRLLSTPG----IELLLPVAAAPSVVAVGERVRSWLGARGLASPEVGETWNAY-ASLSDPQTRTAFLRTLRSVVDAQGQaaLNRLHFTLGLPTLLIWGDQDRLIPPAHGEAAHAALPGSRLVILPagHFPQVEAPLAVADTLDDFIAnhGPPRHADHAEAD-------------------------------------------    
 191 UniRef50_B6QJ15        Epoxide hydrolase, putative...   164     5e-39  1  14.0%  98:418    12:329     -------------------------------------------------------------------------------------------------TTAVRDGTKYSYIYIPAAekPAFLLLHGFPSTSYDWRHVVPLLQELKYGILAPDLLGYGDSDKTTDV----EAYSLKRMADHILELVVGEGLERVVGVGHDWGSTLLSTAALAYPSRFSGLVFTAVGYSPPAPFDLqmVREYIGYPAFAYMEFFNEDDAAAVCDSNPASVTSMLYSTT---PEDWKAHMGTEGGAKGWVISG------RVAPPPSWMSDEEVATHVQIL-RKGGYTGPLNWyrGIDRPFHAQFTTDESKNLNIPALVVTAKYDYGSRPeqIPAAEKHLSIRRIEMFDCSHWIPLEKPKELVALLEEFARSL-------------------------------------------------------    
 192 UniRef50_B0U7K5        Alpha/beta hydrolase fold n...   164     6e-39  1  12.3%  96:419    11:290     -----------------------------------------------------------------------------------------------ARRFADLDGVSLHYLDAGAGDA--VVLVHGIPQTSHEWRHVIPRLAGR-YRVIAPDLRGLGDSSRPAT------GYDKKTLAGDLWQLVrhLGIERFFLVGHDWGGPVAFSLAAQHPEAVRRLAILDVVIPGDGADFSQ------GGRRWHHAFFRTLDLPEALCAGREHLVLRWLFDHYGQRP--------------------------------TSIPEADQQEYLRTYTQFGAFRAMLAFYRALpdAEDNREMLARAGKLRMPVLALGGEEGFGRGSECIASLRRVAEDVrgTVPACGHWIPEEQPERLCDELLRFFGEEP------------------------------------------------------    
 193 UniRef50_Q08XN2        Alpha/beta hydrolase fold n...   164     6e-39  1  12.8% 105:425    14:297     --------------------------------------------------------------------------------------------------------LRMQALQAGPSNGPLVLLLHGFPESSESWREVLPVLGDAGFRAVAPDLRGYGGTDRPKS------GYDIDTLARDIQQLARYLQPDraHVVGHDWGGVIAFHLAAWHPETVDRLVAVNAPHMEVMVRN----------------------------LRNPAQLLRSWYIAYFQVPWLPERHLSKQGGAAVARLIRRAL------VNPAQVSEERLARLAGNFSRPEAASAALAYYRQAARRLLFQRGSRRTPRIRAPFRLIWGKEDDALGLELTQGLEPWFEHPPQvlPGVGHFAPLEAPGQVAALIREHLSVSPGAATPR------------------------------------------------    
 194 UniRef50_B7KDZ2        Alpha/beta hydrolase fold p...   164     6e-39  1  12.8%  91:427     1:296     ------------------------------------------------------------------------------------------MERMIAQPCVTVAglTTEYLTAG--EGSPLLLLHGVGDSAYSWQWVIPALA-RTHRIYAPSLPGFGASDKPK------IEYSPEFFTAFVKAFLDTLDIQQASVVGNSLGGLVSIRLALSSPSRVNALVLVD---------------------------SAGLGRTLNVAMRGLTLPGTAKILGSFGRTSVGAKIWSWSFSALTLANPTRAKRDWLDRIYNMAKDPAYLEATVSALKNENTIAG----------QRDHEIMLDQLSKLNIPTLVIWGQNDRVLPVNHAHTAISRLPQGQLKIldCGHIPQIEQPEAFEAALSSFLNEVVDKATTAPS----------------------------------------------    
 195 UniRef50_B3S707        Putative uncharacterized pr...   164     6e-39  1  13.3%  37:277   157:388     ------------------------------------VVESMEPYCKLATSLGITEIVAvqPTVLKKLSELTGINFSKLSRPVACNPDSVTHCY-----FTTTTGVKIHFVEKG--NGPAIILCHGFPESWYSWRYQIPFLARLGYRVIALDQRGYGESDQPPNV----EDYTMRIINQDVIDLMDTLNIPQAVLIGHDWGSFVVWDTALHFPDRIKAVASLNVGYFPPHPQYNFIqlQPDPKQYDYFLYLQDEGVAETEMEKDVDRTLRYLYSDTTP----------------------------------------------------------------------------------------------------------------------------------------------------------------------------------------------------    
 196 UniRef50_B4SVX2        Haloacetate dehalogenase H-...   164     7e-39  1   9.8% 103:418    25:303     ------------------------------------------------------------------------------------------------------NGLTVRVAIGGS--GSPLVLLHGHPQNHTTWRKVAPTLA-QNHTVILPDLRGYGDSDKPTSDPAHRT-YSKRTMAQDIVMLMDALGFSRFAFVGHDRGGRVGHRLALDYPDRVTCCTFIDIAPTATMYALTD--KSFATRYFWWFFLIQPFPLPETMIAHDPAFFLRKHISG--------------------------------QLKIEGATSQEAFNEYLRCYQNPEMIHAICEDYRAAATIDLDDDAADTSARIRCPLQLLWGGLGTVGQldVVGTWKEKALNVQGEALPCGHSPQEECPEYFIQKLQAFLHSV-------------------------------------------------------    
 197 UniRef50_C7Z8Q7        Putative uncharacterized pr...   164     7e-39  1  14.6%  43:418   351:676     ------------------------------------------RHLAELVVFKVVPALVKEFRIEAVLPPEDDGRSYFLSM---LTGHLIMVQPI-VTENALIRGSRIAYGVYGT--GTPVVLLHGTPSSSLIWRDVVPKLVEKGFKVHVFDLLGFGLSERP---WDSAVDTSMTGQVPILEGLLSLWGLDKTHVIAHDIGGGIAQRLAVFSPERVLSLTLIDVVCFDS----------------------------------YPSKRTKQQMQNGLESLIKASDNDHRAHFREWLLS---------AVKNPAKLEQSSLDSYLEYISGPIGQPSLFEHQVRHYDPKHTLEVTPRLGElkLPVQLIWGADDAWQVVDWAHKLQEAIPGSEliVEDAGHFSPEDQPERISELLVSFLNKH-------------------------------------------------------    
 198 UniRef50_Q4P750        Putative uncharacterized pr...   164     9e-39  1  12.8%  82:417     5:375     ---------------------------------------------------------------------------------TASRFYVTPLTTPPSQPDATPTQRRYHYLDYQPPTGVplLLLHGFPDFSHGWRSVIAPLRLAGFRLIVPDLLGYGLSSAPPTSAASAPAGTLPRLAedLNGLLdhLGSdrVIVLCHDWGSWLGWRFAQWYPHRVMGVCGLCVPFQAPTSKPIPIDSVLKSVPSFGYQKFFSEERSTKIIEEHLDRFLAIilFSADSANEQELRDWHLQGKLERLLTMPEFSHIPLKDLGRSIMDKQLLDQYITLFRSRG-MEGPLSWYRTREINwdRALTSKSLPE-SLPALLIMPNDDVAVPPALGRTMKKHVTQTEFvvAGSGHWIQNEVPGIVVQTFKQWVDR--------------------------------------------------------    
 199 UniRef50_D1I5V8        Whole genome shotgun sequen...   164     9e-39  1  12.7%  97:420     6:313     ------------------------------------------------------------------------------------------------HKHVEVGGLKLHVAETGTGPK-AVLFLHGFPEIWYSWRHQMVAAAAAGYRAIAFDFRGYGLSQHPPEP----EKASFGDLVVDVIGVMDCLGINKAFLVGKDFGAMPAFHVAVVHPERVSGVITLGIPFSLPG--VSAIQMHLLPKGFYVQRWREPGRAEADFGRFDVKTVIRNIYI-----------LFCGSELQVASDDQEIMDLVNPSTPLPPWFTEDDLKVYSSLYENSGFRTALQVPYRTLAEDCGITD-----PKITAPGLLIMGEKDYALKLPgsEKVKEFMPNLEIimAEGNHFVQEQLPEQVNQLLITFLNKHST-----------------------------------------------------    
 200 UniRef50_B8FKC5        Alpha/beta hydrolase fold p...   164     9e-39  1  11.7%  92:417   591:883     -------------------------------------------------------------------------------------------DFPVEFKTMEIEGNRMNYVDEG--KGETLLMLHGNPTWSYLYRHFIKDL-KKDYRCIALDHLGYGLSDKP-----HYEDYSMEAHIRRLGAFIEKLGLKDITLICQDWGGIIGLSYAARNKDLFSRLIPMNTTGFLPGSPKEFIQ--CLGAWAFPYLWSYKVPIAGKKMAMDWNVFLKAGMHLGIVN-------------------------------SKRQLHKRAMDGYLYPFQMVRDRTAIMKSVRQVPMGPLdhDTGRRLAGWNVRTQVIWGMKDPVFVPWFMEKFEEMLPNhsVKIATAGHFLQDDEPEIIIREIRRFLKE--------------------------------------------------------    
 201 UniRef50_Q1J1B2        Alpha/beta hydrolase fold n...   163     1e-38  1  13.2%  96:375     5:235     -----------------------------------------------------------------------------------------------TEHQVVVNGVRLHCVAAGPEDGPPVLLLHGFPEFWRAWERQIGPLARAGFRVVVPDLRGYNLSEKPPGVA----AYRVSTLQKDVAALIHALGYRRSHVVGHDWGGIIAWALAIRQPEVVDRLVILNAPH----------------------------PAAARRMLRLPRQWLRSWYIFFFQLPWLPEHFLLRSG------------RKMLRGTNPAAYTPQDRKLYQRAWEQPGAAIAMINYYRALPRFGNV-----KGNEVRVPTLVLWGERDMVLRA--------------------------------------------------------------------------------------------------    
 202 UniRef50_A4T7V8        Alpha/beta hydrolase fold n...   163     1e-38  1  12.1% 105:425    14:286     --------------------------------------------------------------------------------------------------------TDLHVDDSG-GDGRPVVLIHGWPLHSDSWAHQVDALRDAGYRVITYDRRGFGRSDKP------LIGYTYESLSDDLSAVLEELDLTDVTLVGFSMGGgvAAYCARKGVERIRSVVFASSVTPFMSARKDNPEGPLGKLEA-----------------------ARMAASLTTDQDAFFEQFMTDVFSADGELQVTEQERQEALSMCGDADKPATL---------------------ACMAAFGGTDFREDLPKVTVPSLVIHGDSDATVPFEggKRTYELLPDGRLHVIAGghGIPVSHADEFNAVLLEFLEQDFADGRGR------------------------------------------------    
 203 UniRef50_D2QNX8        3-oxoadipate enol-lactonase...   163     1e-38  1   9.3% 107:471     3:299     ----------------------------------------------------------------------------------------------------------INYKLQGTPNSPVLIFSNSLGAEMMMWDELLPFLLP-YFRVLQYDTRGHGGSTTTPGP------YTIDRLGQ---DVID-------------------------------------------------LMDSLNIEQAFFCGLSMGGLIGQWLGIHQPHRFKKLVLSNTGAKIGNDER-----WNGRIATITEHGMQAIADDTMERWFTEGFRSTNPQRVAdaMFLRSPVTGYAACCAAIRDADFRDNLSQLSVETLVITGDEDPVTNVEQAEFLRDNIPNAQLAILPARHLSSTELPQQYAQVLIDFL------IGESTFDKGMHVRRTVLGDAHVDKANSQITDFTADFQQFITNYAWGEIWT--    
 204 UniRef50_A8VQB1        Shikimate kinase n=1 Tax=Ba...   163     1e-38  1  13.6%  52:418    20:324     ---------------------------------------------------LILAGLLVVFMILPFLIPVSEQDGFAQDTP------------FEDSAFAEVDGVWLHYRISEPEEAetVLFVHGLGGSTYSWRYQVEPFTEAGYRVIRVDLPVFGYSDRQRGL-----EHSMANRSMWLWGLLDELETEEVHLAGHSMGGGVITQMALDEPERIRSLIYVAGAVYNEPR--------------------------------------------SSFILDFPPVQRGIEVIAPRIAFTEARIRGILDSAYGQPVNDELVHAYLDTFNVQGTPGAWVDLLRSTTSVD-----TERLHELGHPALLLWGEDDSWVSVQEGEMLRDALPNASMAVLEggHMPMETDYPWFNDHVIAFLDEL-------------------------------------------------------    
 205 UniRef50_C7PGQ3        Alpha/beta hydrolase fold p...   163     1e-38  1  10.7%  53:415     1:305     ----------------------------------------------------MKKLLSLLFAIAFLLPARSNTIQDSK---------PAAPPANFKHAYAKVNGINIHYVIGGS--GEPLVLLHGFGQNWYMWNRLLPELS-KHFTVIAPDLPGLGESGKPAG------GYDKKTMATYIHGVVKELGYNHIKLAGHDIGLMVAYAYAAQYSDEVTKLALMDALLPGVEPVWSQVKGA-----AWWFGFFSFPASGELVAGR-----ERLFLTNF----------------------------WPVVGHAKDPFTPEEVNEFVRAYSTKGSTTGAFHWFGAFEQD-AKDNQEFMKTKLKMPLLAMGGEygAAFLEDHCKLVAEHVRGAN--IKGSGHWVVQENTAQVQKELLDFF----------------------------------------------------------    
 206 UniRef50_A4QT55        Putative uncharacterized pr...   163     1e-38  1   9.2%  80:416     1:357     -------------------------------------------------------------------------------MASANLAKLAPNDPRVEHKDVQVNGRTYHYMLARPPAGatILLVHGFPDLGLGWRHQVPVLAAQGFQVVVPDMLGYGGTDAPQDV----EPYRHKSIIDDLLALLDslGprRVVLGGHDWGGQIVWRFTEWYPERIAATFSVCTPFFPPMPSFIDLSTLTQIIpfKYQLQFASEEVVDRTTGPDGKPSRAVIRDFLNALWGGAGTIQAADAPKGFTAAEGVNFSIIKDLPQAQLMSVEELDFYADQYSRNG-MRGPTNWYRLRKLNFEdaEETGDEPKIKTPSLFISASRDVALPPAMSANMDMCFESLTRGHVDASHWALVEAASEVNATILSFLD---------------------------------------------------------    
 207 UniRef50_A9B630        Alpha/beta hydrolase fold n...   163     1e-38  1   9.1% 100:418     4:284     ---------------------------------------------------------------------------------------------------AAINGITLNYEVAG--QGAPLLLIMGLGGTTDGWRKELPFLT-KHFQVIYYDNRGVGKTTAPTDFT----AYSMEHFAADAIGLLDHLGIERAHVWGVSMGGMIAQHVALNYPERVQGLVLGCTLPHYANDP-----------------------------ANIPALPDEFHAEASNLAPEAWVLELMLSGTQKTAHEVMQDSVRFNFAPAFVEAHPEVIEEYIegMQNHGPAHGFMGQWSAIM----NHSTIERLPKLQHPTLVQHGDADVLVPIGNGRLLAQIIPNAEflIPGSGHCYFIEQPELASQGVQAFLQSL-------------------------------------------------------    
 208 UniRef50_Q479B8        Alpha/beta hydrolase fold n...   163     2e-38  1   9.8%  96:421     7:298     -----------------------------------------------------------------------------------------------VTRDVDVGATRIHVRVRENEGRPPLLLLHGYPETHAMWHKVASLLQDR-FSLVLPDLRGYGDSGMPASQADAGNQ-SKRVMAQDMAELMTALGYQRFHVAAHDRGARVLHRLCLDHPGRIQTACIMDIA--PTATTFALTNQALATSYFHWFFLIQAAPLPENMIAADPASWLKGCLSR-------------------------------WSMGNEEAFDPAVVSEYVRCFSNPEAIRCSCDDYRAAAGIDLQHDGEDAERRISCPLLVLWGNKGFVGryDVVALWREKALDVSGKGLPCGHFLPEELPNDVARELVEFIARHSSA----------------------------------------------------    
 209 UniRef50_C7QAM5        Alpha/beta hydrolase fold p...   163     2e-38  1  14.8%  81:417    40:334     --------------------------------------------------------------------------------SSPTASSLEQMPAGFSEHKTDVGGIGIDYVIGG--HGPTLVLLHGYPQTWYEWRDVMPALAE-HYTVIAPDLPGAGRSDAPAT------GYDKKDLAADIHGLLAEIGHDhiRLVGHDIGTMVAYSYAAAYPRDVTKLVLSEAPIPDPSIYTYPSLTADGPGAWHFGFFALTNGLPEQLISGREELWTSKFVDDL--------------------------------EVVKGAVTPNDIDVFSSYLKDPAHLEAGLQWFRTLPQDMTNDAVYQK-TKLTMPVLAIggSLGKAVP-DQVRQYAKHVTG--VVVPDSGHWMYEEHPAEMAHILLDFLGR--------------------------------------------------------    
 210 UniRef50_A0Y7N1        Hydrolase, alpha/beta hydro...   162     2e-38  1  13.5%  51:421     1:329     --------------------------------------------------MKIIKITVVLLLAILLCAGVFLYKGEL-----PQREVDAKYTGETSQFFTMENGARIHFRDEGNSAGPAVVLVHGSNASLHTWEPWVEIL-GDSYRIITMDLPAHGLT-----GAVPDNDYGAQAQLRTVDAVVRHVGLDKFTLGGNSMGGGVTWRYTLAHPEKVEAMLLIDSSGLPQFR-------------------QQLEAKAEELGQEKKEAPVFFSLMGKPWFRAIAKYIDPYWMTKQGVNS---------AYNYSPVVTEQLIQRYYELALRAGSRDATLSRFSSPRKAEEPVDAT----RFNVPALVMWGKEDSLISVDVAQLFDAALAQSTLVIYDsgHVPMEEIPDRSAEDVSAFLQSIYGA----------------------------------------------------    
 211 UniRef50_B4R801        Epoxide hydrolase n=3 Tax=A...   162     2e-38  1  14.2%  94:418     1:321     ---------------------------------------------------------------------------------------------MPQFRMVDAGEVRIRAALKG--EGPLVVLVHGFPESWFSWRHQIDPIADAGFTACAIDVRGYGGSDKP----HPVEAYAMERLTADVAGVVRALQPgpAILIGHDWGAPIVWNTALARPESVRAVGGLSVPYLgpSRPFTEVFDALFTQKGRFFYQAWFQKEGPPEAEAEADVRGFLRKFYYAISGDAPPGAWPQKPAGATLLEGLIDPDPF-----PAWLTENELDYYEAEFRESGF-RGPINRYRNHERDFAW-LQGFQGRTIEQPALFIGGTKDPASTadPIAAMRPHVPNLvhMLEGCGHWTQQERPEEVTRLILEWLKGL-------------------------------------------------------    
 212 UniRef50_Q02CG0        4-carboxymuconolactone deca...   162     2e-38  1  11.5%  98:471     2:304     -------------------------------------------------------------------------------------------------PFATSQGCRIYYRLEGSAQKPLLVLVHALGVDHGLWDPQMPALL-RYFQVLRLDLRGHGASDAPAG------DYTIAQLAGDVLATVTR---ERFSYCGLSLGGMIGQWLGALHSDRLERMILANTSPRTPDPS--------------LFEQRRRTVLSEGLAAIEPAVMGRFFSSPSG--------------------------------------TAADSIRTTLLATDRAGYAGCCAAIRDM-------DNRPLLPAIQVPTLVIGGDADVSTPwgHGDLLASQIPGARVAKIEAAHLSNLGRPSTFTNAMLEFL---LPEPAADRLEAGLAVRRSVLGDAHVDRAIANTTDFTREFQELITHYAWGTVWT--    
 213 UniRef50_C0QEV2        DhaA n=1 Tax=Desulfobacteri...   162     2e-38  1  12.0%  93:417    36:320     --------------------------------------------------------------------------------------------YPFVPHFMTLDRHKLHYLDLG--KGSPVVMVHGNPTWSFYFRRLARDLSV-NHRVIVPDHMGCGLSDKP---STRDYDYTLASRVRDLDRLIQSLDLgkITLVVHDWGGMIGCAWALRHLDRIDRIIITNTSGFHLPG-----------------------------AKRFPLRLWLIKYLPWFAIPG--------------IQGLNLFARAALYMAPKQSLSTTVRQGLTAPYNSWKNRIATLKFVQDIPLSPRDKsvDTHLEGltVPMMILWGRHDFVFDLSFLDEWNKRFPHAQTHieDAGHYLFEDKPDETSNLIKKFIEE--------------------------------------------------------    
 214 UniRef50_Q2NCM5        Hydrolase, alpha/beta hydro...   162     2e-38  1   9.7%  51:430     4:323     --------------------------------------------------LLKILAVIVALLVIAFFILRTPDTDP--------EEMRAKYGTAPSQFVTLANGQELHLRDEGPRDAMPIVLLHGSNADMSTWQPWAEAL-RNDYRVIRFDQIGHGLT-----GAAFDEDYSGDRFIESVHLVADRLDLDRFVLAGNSMGGGIAMGFAMRHPERLSGLVLVDAGGAP--------------------------------IRREGGGNLAFTLAQMPVVGNVMSAMLPRSIVERSLKQS---------VSKTEIVDDAMVDRYFDMARYPGNRAAT----RARFSQERVPFEAEQVARVTVPTLVMWGEEDSLIPYTAAGWFMEHLPNATLayPGIGHLPQEEAPERSVADLKAWLAGI--AGNEPAIEPA-------------------------------------------    
 215 UniRef50_B5GAU8        Epoxide hydrolase n=18 Tax=...   162     2e-38  1  14.2%  88:419    32:353     ---------------------------------------------------------------------------------------MTTLAAPARHRLFDLPGGRVHAVEQG--EGPLVLLLHGFPESWYAWRHQLPALAAAGYRAVAVDARGYGRSSRPEG----SEAYRALALTGELVALVRALGEERAVLAGHDWGATLAGQAGLLRPEVFRAVALLGVPYTPPGGppSEVFASMGGEEEFYVPFLQRPGAEAEMERDVRGWLAGFYATLSGRSGSVPPGPFFVP-------RGHAMRERFVTGPLPSWLGEDVLDFYAAEFERTGFGGALAR-YRVMDRDWEDLAAWEGEP-LRQPSLFLAGREDaaWLSDAVDAFPHTLPGLrhLLDDCGHWVQQERPEEVNTLLLEWLADVE------------------------------------------------------    
 216 UniRef50_B9JMN1        3-oxoadipate enol-lactone h...   162     3e-38  1  10.6%  95:415     5:282     ----------------------------------------------------------------------------------------------FEHHTVRVKDIRMHYVRAG--KGEPLVLLHGWPQNWASWKRIIPVLSD-HFTVIAPDMRGFGATSKAEA------GYDTNNVADDIRELVGSLGFERIFLAGHDWGAAVAYSYAAQFQQQVRKLAIFEMVLPGFGIMEEAMTPQPNGNFLWHMGFQSVPDIPFTLISGREDLYLRWFFQTYA--------------------------------YDPSAITSTETEEYVQAMTNVGALRAGLQYYQSYFTSAAQNAQHKQ-HKLTIPVSAWAGEA--CLGpqCLDMAATDVTGG--VIERCGHWIGEERPDFVASQLVEFF----------------------------------------------------------    
 217 UniRef50_A6G385        Alpha/beta hydrolase fold p...   162     3e-38  1  14.2% 104:427    21:308     -------------------------------------------------------------------------------------------------------GLRVRWYERGpaSAPTILCLHGFPDLSVSFREQLEGLSER-YRVVAPDLRGYGGTDAPKRV----RDYDLDLLVRDVVELIDALGEERVHLVGHDWGAVIAWEVAQRHGGRLRSLTTINCPPY--QILFKEMYRPKQLLNSWYMFLFQLPVVPELYVSRDPDQLIRKQMRETAVHEQVFTAERLEPRIRQIRERGLPG----------------------------------LNYYRALARRLPRRLRP-----IHVPTKLIWGTQDHALGQWFADeyRGWVDDFEVviDDGAHWVHEERAEKVNACIRAQVEAVEAARSQREA----------------------------------------------    
 218 UniRef50_A4RE82        Putative uncharacterized pr...   162     3e-38  1  12.2%  73:417    34:346     ------------------------------------------------------------------------IQEELGRKSSNMESIPESRQLPAVHQSttADGVTVFFRHAGppTAPTIVLLHGFPSSSFMFRNLIPHLALAGYRVFAPDLPGFGFTDVDPARG---YHYTFQNLAATFEAFVDALHLRRFALYVCDLGAPVGLRFALRRPDALAALVTQNGNLYREGLARPFWAPFEAYWAQPTPSHCPPARDALRCLVEYPATRWRYTQGSPSLA---------------------------------RRVSLEPCDLDQTLLDRPGIKEVQLDLFHDYRSNVElpAFQRYLAQSVVPVLAVWGRHDPIYTPDGAVAFRRDVRDMEIRWIDAGHFALEGNEVDVAGAMCEFFDT--------------------------------------------------------    
 219 UniRef50_C5CLY4        Alpha/beta hydrolase fold p...   162     3e-38  1  12.8%  95:423     4:294     ----------------------------------------------------------------------------------------------PRHRYISVGGTQIFYREAGPPDAPALLLLHGFPSSSVQFRHLLWTLADR-WRLVAPDLPGLGFSAVPDHR---HFAYTFAHLAEVIEGFVRAVGIEPRALYLHDYGAQTGFRLLASAALRPAALVIQNSEAYFDAGRTDAWSSAEAYWRDP--------------SDANRERMRASLLNEDDIRREFVEQ---------------------LPAHIAERIDPAVIRLACDHVQRPGVADALLDLHRDYGSNVAfpAIQAHLRRARLPTLILWGRGDQYYRPAQAEAFRQDLPDATIEYFDGGHWLLETHPGEVAAAIRDFLQRHLAPPA--------------------------------------------------    
 220 UniRef50_B1XKC6        Polyketide synthase n=2 Tax...   162     3e-38  1  12.6%  39:411 2385:2708     --------------------------------------KTIDPALADKWRSITLPAALQLDTIQL-----AETFAYDLPQEPQLTPQTQSLPSM-VERFVTVRGLETCLCEWGDRHQPLVLLLHGILEQGASWQLIAPQLAAQGYWVVAPDLRGHGKS-------AHAQSYSMLDFLADVDALAKQLGDRPFTLVGHSMGSIIGAMYAGIRQTQVEKLILVETIVPNDIDDAETGNHLTTHLDY---------------------------LAAPPQHPIFPSLEVAARRLRQATPQLPKDLSAFLTQRSTKSVEKGVQWRWDAFLRTRAGIE---------FNGISRRRYLALLKDIQAPITLIYGDQSEFNRPADLQAIQAALPQAQRLTVAGGHNLHFENPQAIAQIV--------------------------------------------------------------    
 221 UniRef50_B7GB27        Predicted protein n=1 Tax=P...   162     3e-38  1  12.3%  92:416    31:323     -------------------------------------------------------------------------------------------STPWLHKEAVVNGTRTHWVEAGRQGDPLVVLLHGFPAFWYTWSSTIIVLADAGYRVVAPDLRGVNLSER------VGVGFDLHTLSEDCSELLDMLEVEKCILVGHDWGGMIAAATAARFPYRVEKVVLLHSVP-------------------------MQALELSRLPWSHRIRLRSLVFFSKFTWLARTVFSFHRAWL-------------LSRYIQGTGVDDRTLDVYRDALGRPGSIFCWMQYIRTLPTSLEQAN---KLGKILSPVLFVSGRhsLWLPSDCYRRLNEWVEGstVTLPSCGNWSPHDAPDELNQVLLRFCA---------------------------------------------------------    
 222 UniRef50_Q0ASX8        Alpha/beta hydrolase fold n...   162     3e-38  1  12.3%  50:415     3:313     -------------------------------------------------FLIAVFLGLALLAGIVVYVLNRPATGTEAPAEGALADSVDSPWWTDADRLVDVDGVRTRVRIEGPDDAPALIMVHGFSHSLESWDAWADALSAD-YRVVRMDLPGHGLT-----GPDPQARYSVPQTVEFLDGLMGALEIDRATLIGNSLGGLVAWRQAAANPDRVDRLVLLSPGGFSINGVTEDPVPVPMGVQFYLIQAPQPIITAATGAL----------------------------------------------YGDPSAMPPGTAERVLELMRGDGVGQAMVERLRVF----TLPEPGADLAQVQAPTLIMWGEADVIIPPAHGPQFATAIPDARLITYPggHVVHEEAPASTLADLRAFL----------------------------------------------------------    
 223 UniRef50_Q1N148        Predicted Hydrolase or acyl...   162     3e-38  1   9.7%  96:426    19:302     -----------------------------------------------------------------------------------------------ESQYIEVNGLTIHYRDEGS--GPPLLLLHGVASSLHTWDAWTNQL-KNKYRVIRIDMPGFGLT----GPDSVSDAQTPEYMNRVINGLVDQLGIQRFFLVGSSLGGYFAWNYAAAYPERLYKMALLSPVGYP--QDMPFWLGFASFPGLHWITPTMMPRFLVHWTAES-------------------------------------------AYADEDKLDESVKQRYFDFTQRRGNRESYVQHFLQFRELAHGEDKPQKVKDVLTPTLLMWGAQDDWIPLDVMREFYRDLSYSDYIVYEggHLPMEELPLQTARDVDHflRKVDNHPQETA-----------------------------------------------    
 224 UniRef50_C8SGH4        Alpha/beta hydrolase fold p...   162     4e-38  1   9.9%  54:415     3:317     -----------------------------------------------------LSFLSGFLIAVLAAGPSTSASAPHSGVPLGQSTDLSA---RIVDGKADVNGVAYHYLLA--AGGptVVLLHGWGTTSYMWRFVMPQLVARGYTVLAPDLRGLGDTAKPAA------GYEKAAIAEDIRALVNQLNLGPVvlVGHDMGGMVAYSYAAQHPGEVTTLAIVDVPLPGIEPWNELIQ---GPRTWHFRFHSVRDVPEMLIAGRELEYLKWF---------------------------------HNAEGVNTRAFDTEADEVYGRSYAQPGALRAGFEYYRAFPRDEEQNKAFAK-HKLTMPVLGISGSGGLamYEGHLRHVAENVRA--VVVEGSGHWVPEEQPAAVTRALIEFL----------------------------------------------------------    
 225 UniRef50_B0CFU2        Hydrolase, alpha/beta fold ...   161     4e-38  1  10.3%  88:417     1:279     ---------------------------------------------------------------------------------------MKPTEPLPEDRYVKVGSVNTRYWQIGD-SGRAVILLHGGGGYIELWKYNIFELA-KHHRIYAFDMVGAGRSERP------NTDYTYDFMAQFTREFMKVLDIPKADLIGTSAGGGVALTFALKFPALIDRLVLAGSAGLGPE---INLLLRITAIPGLGKLLSSPTKSGLRML------CKQSVYNSNLITEEMVDEFYQMAILPGAAAATINLGRSIFS--------------IWGQFSQP---------------------ITERLQTITAPTLIIWGQQDPMVPVSHGQNAAQIMPNARLEieECGHWSSIEHPQKFNQIILGFLSS--------------------------------------------------------    
 226 UniRef50_D2QF94        Alpha/beta hydrolase fold p...   161     4e-38  1  12.5%  91:417     1:283     ------------------------------------------------------------------------------------------MSYIKAGTDASGHDIKLFYQDLGtaAGSTVVLIHGWPLSHEMWDYQLAELPAHGLRVVAYDRRGFGKSSQP------WDGYDYDTLADDLKAVLDELDLQNVTLVGFSMGGGEVARYMSRHGGARVAKVAFVSAVTPY----------------------------LLKTEDNPDGVDKDVFDEITENLKKDRAAFLQ-----TFGKQFYGVNLISKPVSQAHLDGDFARAYVA------SHKATLECAKSFAE----TDFRDDLAQIQVPALVIHGDSDKTVPIEagERTANALPNAQYIVYDGahGLFMTEKDRLTQDLLSFIQE--------------------------------------------------------    
 227 UniRef50_B2AIK0        2-hydroxy-6-ketonone-2,4-di...   161     4e-38  1  11.0%  71:424     2:300     ----------------------------------------------------------------------SATSQHTPSTPPSTSAKPASLHppVGRFVTTARGLRLHCLDTGA--GEPVVFIHGSgsGHSNFRHNVPAFAAAGFRTVVVDLPGYGLSSKPD-----DVEYTLDFFVAALREQLLALELPRCVLVGNSLGGAIALQYALDYPEHVSRLVMMAPGGVEERETYFEMPGIQRMVSLFTGGHM------------NPDTMRQLL---------------------------------QLLVHDAGLVTDALVDERMAVCR---------EQPREVLATMKVPNLTARLGEIRCPVLGFWGTEDQFNPASGAAKFLSGCADARFVMIncGHWVMVEHAAYFNRECLGFLADTAPRGAA-------------------------------------------------    
 228 UniRef50_Q1DFU0        Hydrolase, alpha/beta fold ...   161     5e-38  1  10.1%  63:420    13:313     --------------------------------------------------------------VLTACGPAPESDSNDMRLAPEGAEVASTEqtPRERSVRLRTGVTLRYVEQGLPLGPAVVFLHGFSDSNHTWDLNLRTF-PRNHHVYVLDQRGHGDSTRPA------CCYTQQSFAADVDAFLEAVGERSAILVGHSMGSFIAQQVALDFPRRVKGLVLVGSAPTVAGNPVAL-------------------------------ELKSIVDTFEGTVDPEFIRAFQESTFVRPVPASYINTMVSESSKVPARVWQDALDGLLA------------------------EDHSARLNHIRVPVLVIGGDQDGFFSVADQQALVDALPDAEFKlpNTGHAPHAEQPRRFNQDVHRFLREVTR-----------------------------------------------------    
 229 UniRef50_B5HTB9        Alpha/beta hydrolase n=1 Ta...   161     5e-38  1  14.5%  89:420     3:286     ----------------------------------------------------------------------------------------PAPEETHAGRLVDVAGGQVRVYERGRPDGPALVFLHGFLTSAFTWRNIHPAFTG-HYRVILVDLPGSGDTPAP-----GDGRWDAAHWVRLVEELLDELGIGSAVFVGSQMGGSLAAWFAARRPERVDRLVLMAAGALGESEANLTLYRLLA-------------------------------------------HRWLGAWIARLFPKGMFERRWLAAHGPGYHPEPGVVDRYFRQLRRQGAQMARVGLQLRLSYGERFDALAEPISGLAVPTLLLFGAEDRLVPVSTGRRFESLLSDATLVvpDCGDFPQEEKPKEITEAISGFLAKADG-----------------------------------------------------    
 230 UniRef50_D1P580        Alpha/beta hydrolase n=4 Ta...   161     5e-38  1   8.1%  48:416    14:334     -----------------------------------------------IFDKLLLVFLMFFSLHSTVTFGMNNIKQPAAT---AIASTIQADEDNFEHHYVMVNGLRLHYVTAGT--GEPVLLIPGWPQTWYTWRYVMTELAANGYMAIAVDPLGTGYSDKPES------GYDTGAAATTLHEMMTQLGHTQYSIVGHDIGMWVGYALASDYPEEVKKIALMEAVIPGLAPAPSLFVSPEENIFLWHFMFNQVRDLPEMLTAGKESEYLNFIFDN-----------------------------------WAYRRDRVAAQTYIDAYASPGGLRAGFAYYRAIPQTIQQNKLRAQ-KKLTMPVLAI--GADHATRDAPQLTLQGRANQLQGAMLgcGHFVTEECPEQLMSYLLPFLK---------------------------------------------------------    
 231 UniRef50_A1HM47        Alpha/beta hydrolase fold n...   161     5e-38  1  11.1%  99:423     2:263     --------------------------------------------------------------------------------------------------FAKVNGINLYYEDQG--QGPALVFIHGLGENASSWKRQIEFFS-KSFRTIVVDLRGHGRSD------DGAEFITMDILAKDVLALLDQLGIEKAHFVGHSMGGLINQEIAAHNLHRMLTMTLSDAAGYYPPPLGTTGLE---------------------------ERLKRIDTLS-------------------MEEVAEAITNSACRPEAPEWLKVEVRQMFAANRKQP---------YRQATISTLKADYRQYHARMKVPTLLLVGQFDKTTPLSYAQFLNEAIIGSKliIPDAAHMTKVENPAVYNRALAEFLAPYEPNAA--------------------------------------------------    
 232 UniRef50_A0AZ59        Alpha/beta hydrolase fold n...   161     6e-38  1  12.4%  95:416    28:309     ----------------------------------------------------------------------------------------------FRHRFDTVDGVRLHFVEGGRADGETIVLLAGFPESWYAWRRVMPLLADE-FRIVAPDLPGQGDSDRP------LVGYDTQTVAATLARLLERQNIARFYLAAHDVGAWVAYPFAAMYPESVKRLALLDAGIPGVTLPAALPIEPGNAWRTWHFAFHTVADLPETLIAGKEREYLDWFLR--------------------------------RKAANPESFSDADVDEYLRVFTRDGGLRAGLAFYRAVSESSAQNRKLQALGKLKMPVLAVSADQGSIPDMAglEHVAEEVTAATIAY--SGHFIPEEQPQALARELRDFFR---------------------------------------------------------    
 233 UniRef50_Q98I15        Hydroxymuconic semialdehyde...   161     6e-38  1  13.7%  82:423    30:332     ---------------------------------------------------------------------------------AALRENRSPEEAAPAGafATVDGLKIHYQVWGPLDGPPLLLFHGTASWAETYRDIAAPLGEQGFRVIAPDMPPFGYSQRPA-----DQDYSRAAHAKRVLGFADALGLRHFSVGVHSYGGGGVIEAAFSAPARIDSLILLDVAIGLGQTEAPALPLASLLDRDWP---------------------RQLLTASTFTNPLMTGPGLRKFV------------------ENDDLVTAERIAIYTRPLNVKGTTNAVGHWLVSGLYHDERKSLAAdyRAFTPPVLVIWGRDDSTTPLAQGEEIASLFAHAELAVLDgnHIPQVERPHDVVRLIGNFLKRTLPAAR--------------------------------------------------    
 234 UniRef50_A9A0U9        Alpha/beta hydrolase fold n...   161     6e-38  1  12.7% 104:425    53:323     -------------------------------------------------------------------------------------------------------GLKIHYRDEG--QGPVLILIHGVCASLHTWDGWVEEL-KDHYRIIRVDLPGFGLS-----PLTDKNIYERQRAVAVIEEMVKTMGLDRFSIAGNSLGGHVAWIYTHAHPERVEKLILIDSAGF--QMKMPWILKFASTWPVSMVSRRMMPKVILYEAVNQ-------------------------------------------VYGDPRRMEKGTRERYFELAMRKGAKSDYVDIFKKLDHELSKRSVSDGIDEISVPTLVMWGDKDTWIPYAEsaRWRHALPDARFIVygAGHVPMEEIPVETARDARAFLSGRITGARAD------------------------------------------------    
 235 UniRef50_D0L2E7        Alpha/beta hydrolase fold p...   161     6e-38  1  11.8%  82:420     5:308     ---------------------------------------------------------------------------------PPGPVLRPVTDTDVRTEYHTIHGYRRAYRIAGS--GPALLLIHGIGDNSSTWNEVIPILA-QHYTVIAPDLLGHGKSDKPRA------DYSVPAFANGMRDLLVVLGHTKVTVVGHSLGGGVAMQFCYQFPRFVERLVLVAAGGVTRDVHPA------------LRLISMPVAHQLLSMLRVPGVVPGLKLAAKGVVGVPLQTALPDSIAPRRVLNDHEDLIRVLADLADAKASAAFLR----------TLRAVVDWRGQSITMLDRCYLTERL-----PVLIVWGDDDTVIPYHHAELAHAAIPHSQLefVGSGHFPFHDDPERFCRVVIDFMQTHEP-----------------------------------------------------    
 236 UniRef50_D1Z7Y1        Whole genome shotgun sequen...   161     6e-38  1  12.2%  81:416     3:349     --------------------------------------------------------------------------------PDALTPHDPRVTHHFtsTSHTTESITYHYLLagQPTG-TVLLLHGWPDLSLGWRYQVPFLLSLGLRVIIPDMLGYGLTSSPASPT----EYSLKKLSSHLAHIIREvkPERVLLGAHDWGAFLGWRLAIYFPQLIKGIFAFCIPYTPPETKVTTLEEHVRKHPELGYQLQNAGGGIERVVGEDKKKLRGWLNAMFGGLSEDTGVMAFDPW-------KGLDLDKLEEVEASPLVGEEVVDFLWYEYSRNG-IQGPMNWYRTREINLQDepLAEEYaqFQVPAMIVMVGHDPALPPTLTDGMEKYFAKgkEVIPEASHWVLIHTPEEVNKLVGEFLE---------------------------------------------------------    
 237 UniRef50_B8HXG3        Alpha/beta hydrolase fold p...   161     7e-38  1  10.0%  99:427    14:307     --------------------------------------------------------------------------------------------------TWLWRGYRINYVQKGERGAN-LVLVHGFGASTDHWRKNIAELSQD-YRVWAIDLLGFGRSEKPA------IAYTADLWRDQLQDFCASVVQAPVFIAGNSLGGYTVLCLAVDAPAQVEGVILLNCAGPFSDAGEQTPKSPWQQQLAQVQRQILQLPFAIDILSFV------------------------------LFHYTRQKSRIRQILLQVYKDPTAVTDRLVEEIYRPAFDPGALGVFAAVFKSPPGRKLDQLLQALDRPLLLLWGTADPWMTPAKAEKFCQYFPQATLTWVDAGHCPHDELPQVVNPAIHDwlQALPASQGTSGA----------------------------------------------    
 238 UniRef50_Q64AD0        Predicted hydrolases or acy...   161     7e-38  1  11.3% 100:417     4:261     ---------------------------------------------------------------------------------------------------VDVRDIEINYHEEGT--GFPLVLIHGLSGDQAGWVWVMPEFS-KHYRTIAPDVRGHGDSGKPDMP------YSIQQFSADIFALFQKLEIRQAHLLGFSMGAAIAQQFVLDYPERVKSLILVSTFSHTDVH-------------------------------LHKAFIRLRKSLDRGGYSTFFDEMVKLAFNPDFVTANTHFMEEVKAMGIKINSPTAIAHATEACMK---------------------FNVKNRISQISVPTLIISGREDTFTPLAISEQIHQSIHSSQWKILEggHNIYIEKPAVLAQVVLEFLGR--------------------------------------------------------    
 239 UniRef50_Q9KZR5        Putative hydrolase n=2 Tax=...   161     8e-38  1  10.5%  87:420     4:292     --------------------------------------------------------------------------------------QRPRTYDPPRPRTVTVDGARVACWEAGPPDAEPVVLLHGYPADHRCWRHQVPPLSAR-HRVITPDLLGWGASDRPLHLS-----FDYDTEVARVGRLLDALDLDSVNLVGHDYGGFLSLGFTQNHPDRVRRLALLNTRAHGT------------------------------FTRRWYAVFGLLGLLGRNPLLSGPARHLPYAALHR---------RSLAPLVRAGHLDARVVDGYLDWMAAPEGRRWLLHYFGDY-RTPARPELRRRLSGITCPTAVVWGRADPYLSPAIATGLAGSVPRAELTMldAGHWVMDERPADVTRALGRLLERTTP-----------------------------------------------------    
 240 UniRef50_Q1GRR5        Alpha/beta hydrolase fold n...   161     8e-38  1  11.1%  50:417    25:335     -------------------------------------------------VLIPLAVLAALVIGFLLLLTPDTDREAMIAKYGGPDAAFAAGPA----------GQRIHYRDQGRRDGPAIMLLHGSNASLHTWEPLVERL-GADYRIVTLDLPGHGLTGAIPGR-----DYDADAMMEAVDVVAAKLGLHHFVLGGNSMGGWIAWRYALAHPARVDALLLIDAAGMPLRRGEKAPESNVGF--------------------------------------RVLEYPFGRWLATRITPRMLVEQSLLGSVERQDIVDAAMIDRYWELLRFPGNREATVIRARA----SREPEMAARVGEITAPTLILFGDKDRIINPSAAKTFNERIAGSEVVLLPggHLPMEEAPDQVATAIADFLTR--------------------------------------------------------    
 241 UniRef50_C6D399        Alpha/beta hydrolase fold p...   160     9e-38  1   9.3% 105:415    14:276     --------------------------------------------------------------------------------------------------------IELYFEDLGA--GKPVILIHGWPLSGRSWENQVPALIEAGYRVITYDRRGFGQSSQP------WNGYDYDTFAADLHKLIEHLDLKDVTLVGFSMGGgvARYVGAYGTSRVKRAVL---------------------------------------AAAVPPFFYKSDEHPEGGLDEATIQSFHDGLKKDRVAFLDGFTHNFFKAGDRTDLVSESFRAYNVDIASFASPKGTLDCTDAFGR----TDFRGDLAKFDIPLLVIHGDSDAIVPLEVSGQlhEAVAGSQLVVIEGghGLNATHSEEFNTALIHFL----------------------------------------------------------    
 242 UniRef50_A4XQ24        Alpha/beta hydrolase fold n...   160     9e-38  1  11.3%  81:417    29:315     --------------------------------------------------------------------------------PDRPLDELKARWAPPPSQFVELDGLSVHLRDQGRRDDppIVLLHGTSASLHTWEGWVTELAKQR-RVISLDLPGFGLT-----GPFPDGDYRLERYTGFLLTLLDRLQVPRAVLVGNSFGGQLAWRFALAHPERSARLVLVDAAGYPRNAESVPIGFRLAGVP------------------------------------------ALAPVMSHLLPRAMIESSLRNVYGDPDKVDDELVERYYQLTLREGNRQA----LRQRFAQAPSGELHERIGELQLPTLIIWGGRDRLIPPDNAERFAADIAGSQLVLfdLGHVPQEEDPQRTVAVLLAFLAR--------------------------------------------------------    
 243 UniRef50_A4FAZ0        Alpha/beta hydrolase n=1 Ta...   160     1e-37  1  13.1%  98:417     3:272     -------------------------------------------------------------------------------------------------RNVIVDGVRIAYRCRG-GDGLPLVLLHGTPSHSAIWRHVIPELEAGGHSVLAYDLLGFGASERP-----VDRDTSVTAQARLLGELLTTLGIGRCVLVAHDIGGAIGQIFATARPDRVDGLVLVDPVSYDSWPSRTW---------------------------------------------QHIIRDHLDDYAAMPPRDFEEMLTRQLRMTVTGEMADEVLETYLAPHRGPVGRVSFFEHQVRHYDSAPTERVAPLLGELPMPVRILWGEDDRWQPVAYGERLASDIPHAELvvPGAGHFLMEDRPHRVAEEILRFASR--------------------------------------------------------    
 244 UniRef50_A8ZTD0        Alpha/beta hydrolase fold n...   160     1e-37  1  11.5%  80:419     1:299     -------------------------------------------------------------------------------MATPVDKTPFAHLYPFDSHFVEINGWNCHYLDEGA--GEPLLMLHGNPTWSFYYRELVKRFSPD-FRVVCPDHIGCGLSDKPPAD---QYGYRLKDRVNDIETLVNHLNLDGITLIVHDWGGFIGCAFALRNLEKIKRVVITNTAAFLKISGKP-----------------------------IPARLRVLRHIAPFAVP--------------AVLGANLFARAALYMAPKKKLPPDVKAGLIAPYNSWANRIATLKFVQDIALSEKDPSYenLYKLSGFPMLICWGLHDFVFDTDYFAEWQRRFPHAEShfETAGHYLLEDEPEQVGDRIKAFLKKHP------------------------------------------------------    
 245 UniRef50_B8FLR5        Alpha/beta hydrolase fold p...   160     1e-37  1  11.7%  92:417     5:325     -------------------------------------------------------------------------------------------AHYHPEKFTKNGDIEIAYDAFGDPQAKPLLLIMGLADQMITWhkLCRVLADSGFFVIRFDNRDIGRSTWLEDEGAPntPYTLKDMALDALAVLDALGLESAHVMGASMGGMIGQALALDYPERVRTLVSLISAPDILPNKN--------------VLKGMGKDIGDWIAGRDSD-----FQTISPSPAVFPFLLNPMPTDREGFVDFYVGLNLTVNGKGAPLNLEESRRHAQELYDRGLCPNGAL---RQLAGILASPSRNQRLKSLKTPTLVIHGAKDPLIPVRLGMETARLIPGARLKIinMGHWLPLPVWGELLESLENFWDA--------------------------------------------------------    
 246 UniRef50_A1ZZI8        Hydrolase, alpha/beta hydro...   160     1e-37  1  10.5%  46:417     1:309     ---------------------------------------------MLKIIRYIFFTLVILLAGVFIWAYRSDIAVEQLKTRYA----------PPPSEFVSIQGMQVHYREEG--KGFPLVLIHGTGASLHTWQGWVDQL-KQHYRVIRFDLPAFGLT-----GPHPQHDYKISTYVKFVQALLQKKGIKKCHIAGNSLGGNIAWRFALAYPDRVGKMILLDASGIPLKKKRKKLWIM---------------------------QLARTPIVNWVMRYATPRAIFRKNLLE--------------VYSDDAKVSPALITQYQQLTLRKGNREAFIQ-----RAKTPVEDRSEDIPRINTHTLIMWGKDDAWIPLELAYAFKEKLPNNQLIipNVGHVPMEEIPLKTAQDALHFLEA--------------------------------------------------------    
 247 UniRef50_B8EM96        Alpha/beta hydrolase fold p...   160     1e-37  1  12.9%  99:424     6:289     --------------------------------------------------------------------------------------------------FITVDDHRLFYREAGDPKNPAILLLHGFPSSSHMFRDLIPLLAE-NYHVIAPDLPGFGFTEVRRDPP---FKFSFDTLAEVIAAFTDAVGIRRHAIYVFDYGAPVGFRLALARPEQITAIISQNGNAY-EEGLSEGWNPIQAYWREPTAEHRAALRAFLTLEATKWQYVHGV--------------------------------------ADEAAVAPESYTLDAALMARPGNDEIQLDLFLDYASNVAlpAFQDYFRRERPPLLAIWGCNDPFFLPAGAEAYVKDNPKAEVRFLDTGHFALETHSAEIAARMIDFLDRSAVAAAA-------------------------------------------------    
 248 UniRef50_D1N6X5        Alpha/beta hydrolase fold p...   160     1e-37  1   9.6% 100:463    10:329     ---------------------------------------------------------------------------------------------------ADENGvrIAYFD-NEAAGQPVLLIHGFAEFSCTWEPVLEYLPPD-FRYIRLDVKGFGYSSK-----NDPDRLSLFDFTRSTADFIRSLDLKDLVLIGHSMGGAISSLiySDVRSRVDKLVLIDSAG---------------------------------MFEQVPAFIETLAAMS-----------HDNPLLRFANEDLMAYLIMQQAYASEMKISQELVGAYAEAMRLPGSRECVIAAARQFL-IPNVPAFQQDLRKLRLPTLIIWGAQDRIIPMEDGEKFHDCIAGSRlvLPECGHSPQEECPEETGRLIADFLLDAPPAPAPETStpAKAAAPVVESRSLRERSMDQLRQLRDVYKLRMTR----------    
 249 UniRef50_A8HWZ4        Beta-ketoadipate enol-lacto...   160     1e-37  1  11.8%  98:471     9:321     -------------------------------------------------------------------------------------------------PFITIKDVTLHYKLSGPRKgpVLVFSNSLGTDFRIWDAVARAFAP-SHTVLLYDKRGHGLSE---------------------------LGTAPATIASH-------------------------------AADLAGLMDALGLGPAAICGLSVGGMIAQALAAARPDLVRALILCDTAPKIGTPE-----SWNTRIAAIAQDGLSSLLDPVMERWFTPAFRRADNPDYarMMFARQSGEGYAATCAAIRDADLTEEARRIAVATLCVVGDQDGSTPPDLVAAMAALIPGARYEVigAGHIPCVEQPAALIALMRDFLAGLRPAADDRYAQGM-ETRRAVLGDAHVERANAQTTDFDRPFQQLITEGAWGTVWS--    
 250 UniRef50_B6IW13        Hydrolase, alpha n=1 Tax=Rh...   160     1e-37  1  14.1%  59:426     4:315     ----------------------------------------------------------VLLGFLAFVLLIVAATAVWLYTPDLPREEVEALYLRDPGDYREIAGQRLHVRVEG--EGPAVVLIHGFGSSLLTWDAWARDLATD-HRVVRFDLPGHGLS-----GPAPDDDYGIDRSVALIGALLDALGLERATLVGNSLGGLAAWRFAAARPDRVEKLVLIAAGGFVPPG---------------------------------------------AAYGQRIEVPPAFRAMRSVLPESMVRASLASMYGDPARLDPETVRRYWTMMRAPGVRDALVRRL----EDFTTEDPVPLLARIPAPTLVMWGARDVMVPATDAARFAGALPDARVVipDLGHVPMEEAPERTLADLRAFLAGVPVGTAPAA-----------------------------------------------    
 251 UniRef50_B8HY25        Alpha/beta hydrolase fold p...   160     1e-37  1  13.3%  90:420     6:299     -----------------------------------------------------------------------------------------AASSVAITSTWQWHGHAIRYQQAGDR-GVPVVLIHGFGASSDHWRKNLPDL-GLHHRVYSIDLLGFGRSAKPQ--PGADVAYTFPTWASQILDFCREIIGEPVFLVGNSIGCIVALQAAIEAPEWVRGIAMLNCS---------------------LRLLHERKRISLPWYRRWGAGVLQKLLAYPSLGQLFFAQLANARTIRKIL---------LKAYGRKSAVTEELVQLLLQPAADPGAAEVFLSFISYSQGPLP----EELLAQVTCPVLILWGAADPWEPIELGRALGQFPVVEQFTPLEggHCPQDEAPEIINPLLQQWLASHCP-----------------------------------------------------    
 252 UniRef50_D2RRT4        Alpha/beta hydrolase fold p...   159     1e-37  1  11.8%  99:436     6:302     --------------------------------------------------------------------------------------------------VATVGDCRIAYRRAGT-SGPPVVLCHGAgdATVSWRHAIDALSED-YRVYAIDWPEYGRST-------GSVTHTIETYVDVLAGFLESLPYERVSLVGISMGGGAALGYALERPDRIEQLALVDSYGLGGRLPNALPWKLLSRVP----GMTEFGKIAAGATTDSVRLVLDSLVADSGG------------------------------------LSDEFVDDARAKLMEPGSIQAFKEFQGnsYDGRVATNFVDDLESLSVPTLLIHGEEDPLVPLEWSVRAAELIPEAElvIENCGHWAPRERPERFNESLRNWlrRCAPTPepLAEMPGITRVGD-------------------------------------    
 253 UniRef50_Q6AJW5        Related to haloalkane dehal...   159     1e-37  1   9.8%  92:416     4:283     -------------------------------------------------------------------------------------------EYPFQSNYFEIGGQRLHYVDEG--HGPVIVLVHGNPTWSFYYRRVISLLS-KTHRIIAVDHMGCGLSDKP-----QDYSYTLQTHRQNLFQLLEHLQIEKYSLVVHDWGGAIGVGCAAFAPERVEKLVVLNTAAFRSTHIPLRISLCRAPLFGEYLVRGLNGFAWPASFMAVQKR-----------------------------------------------LSKEVVAGYLAPYSNWEKRVAVYGFVHDIPLNSAHPSYgeALVARQVPTLILWGGKDFCFNDHFYRQWCERVPYAevYYENGGHYILEDEFADIAPRLERFFT---------------------------------------------------------    
 254 UniRef50_B8HNQ0        Alpha/beta hydrolase fold p...   159     2e-37  1  11.4%  84:417   119:409     -----------------------------------------------------------------------------------VSTDLDQPYFEQPKQYWLWRDWRISyhVAHPQSSIPIILLHGFGGSIGHWRHNIPAL-GAHHQVYALDLLGFGASEKPVTP------YSIQLWAEQVYEFWRDFIRVPAVLVGNSIGSLTCLTIANHHPEMVRGVAMISLPDQLNQQPSAPWLDSLRAM-----LTSPLILQPLFHLIRHPCIVKHW---------------------------------AKLAYARREAITDELVEILTAPARDKNAALAFVSMLKSMLSPRFSPQVRPLLQSLAIPSLLLWGQQDRMIPLSLGRHLAACNPILRLIELeaGHCAHDECPDRVNWELLHWIET--------------------------------------------------------    
 255 UniRef50_D2PSE5        Alpha/beta hydrolase fold p...   159     2e-37  1  13.8%  96:414     9:288     -----------------------------------------------------------------------------------------------EHHRAHVNDVQLHYLLARspVDEAVVLLHGWPQTSYAWRKVIPGLSAR-YDVVAPDLRGLGDSQR-------RGPYDKTTLARDVAELVEQLGHRRIHVVAHDMGATVGYALAHQEQQRVASLVVLEMLLPGFGLEEAASLREGGTTFWHLAFHLAEGGHAEALTQGREATYLHRFYTDS--------------------------------LYDPTSLTAEDRAHYLRAYEAPGAMHAGFEWYRTLFDDARDNRRRVQERLLDIPVLAIGGAhdRV--RESLAQVARSVQG--ETWEHCGHYPHEEQPERLVTRLLEF-----------------------------------------------------------    
 256 UniRef50_A3J807        Probable oxidoreductase-put...   159     2e-37  1  12.5% 105:416    29:289     --------------------------------------------------------------------------------------------------------VTIRYIDTGQGFKGTVLLMHGIPTWGYLYHAVIPLLAQAGYRVIAPDFLGHGWSDRRD-----RFDRSFQDQARMIIALLDSLQLDAVDVVGHDTGGAVALILAIEHQPRVNRLVITNSVCYDSFDDDM--------------------------------------------LDFGHPMRWKPRPVSDLIAALEESL--AMGLSNPTRLTPEFCAGILAPWSSEAGKLSLIRNASALNANQTMA-LVDRHGSISAPTMILWGMDDPWQKSEDGMRLSNEIPGALFklIGASHWVQQDAPEEFTEALLEFLA---------------------------------------------------------    
 257 UniRef50_C1BP15        Haloalkane dehalogenase n=1...   159     2e-37  1  12.8%  12:416     8:378     -----------KILVPPGNSKSPSIINwtTFGILYVVFILE---VLNVFKIIVMGVVRFFFAGRSPV---------LLRTPETQFQNLEKLAYNFEPHYLELdggrMHYVDEGSRTGEVILCLHGEPSWSFLYRKMIPELVREGYRVIAPDLIGFGRSDKFTSVHQYSFELHMNSIKRLLEELNLLNDKSHITLVCQDWGAALGLRVVKDCESSFSRLVIMNASLPTGDITFDDYGDP----------------------SKRPSSFVSIV------------NFAPLLLWKSLMKIVDRNIPVYGLFRLMEGFSSEVAHAYSAPFPHPLYAAGPASFPRLLPTTKdnMETRHFLKTWEKPALLMFGDRDLSTGP-YQRLFKNIIKDSTMkiVGGGHYMLETHAQELTEHIVDFLK---------------------------------------------------------    
 258 UniRef50_Q8DHQ3        Tlr1892 protein n=7 Tax=Cya...   159     2e-37  1  12.5% 100:418    16:293     ---------------------------------------------------------------------------------------------------WTWRGHRIRYSVNGS--GAPVVLVHGFGASIGHWRKNIPALTAAGYRVYALDLLGFGASAKP------DLAYSLDLWAELLADFGQAHVGEPVVWVGNSIGGLLCLMMAARYGHTCRGVSVLNCAGGLNHRPNenWTQSLFTAIFRALVASPIIGHLIFHQIRQPERIRK-------------------------------------TLTQVYANPEAITDELVELLHRPAMDAGAKEVFARVISAPPGPKIVDLLPHIQVPILVLWGEVDPWTPVshFEAHQERLPIRIERLPHTGHCPHDDRPELVNPILIEWLQQL-------------------------------------------------------    
 259 UniRef50_C6BG14        Alpha/beta hydrolase fold p...   159     2e-37  1  10.0%  95:423    48:329     ----------------------------------------------------------------------------------------------PQSRYLPLMGTRVHYTDEGAANSetLLLIHGFGASLHTWDGVLPQLTRR-YRVIRLDLPPFGITGPLRDAQGRPRTMELPLYRDFIDAFVDTLGLSKLTLIGNSLGGMVSWDFAVRHPGRIEKLVLIDSAGFPM--KLPIYIDLFNHIGVRMTSPWMLPEGIIRAATRD-------------------------------------------VYGDPSRVSEPTLRRYADFFYADGARQAIGKMVPKFRFDDVDTS---GLASIRVPTLILWGQRDRWIPPAHAGEFARRIPGATLRMYPagHIPMEEDPVRVGTDLCAFLDQGRASTR--------------------------------------------------    
 260 UniRef50_C3JK77        Hydrolase n=4 Tax=Rhodococc...   159     2e-37  1  14.0%  99:420     3:284     --------------------------------------------------------------------------------------------------FRTIHGYRRAFRIAG--EGPAVLLLHGIGDNSSTWTEIIPHLAKK-YTVIAPDLLGHGRSDKPRA------DYSVAAYANGMRDLLSTLGIEHVTVIGHSLGGGVAMQFSYQFPHMVDRLVLVSAGGVtdVHPLLRLMSVPVVNEVVKLLRIPGAMPLVRMAGNLAGVVHGSNLRPGTMLHDTPDLIRVLADLP----------------------DPTAYEAYLRTL------RAVVDWRGQVVTMLDRCYLTENL-----PVQLIWGDDDSVIPVSHAHLAHAAMPNSRLEVFRggHFPFRDDPMRFLQIVEDFLSSTAP-----------------------------------------------------    
 261 UniRef50_D2PLU4        Alpha/beta hydrolase fold p...   159     2e-37  1  15.4%  95:418     7:290     ----------------------------------------------------------------------------------------------PRHRSRKVDGLTVHYREAGAPCSTAVVLLHGFPTSSYSFRAVLPVLGRHAY-VVAPDLPGFGFSDAPP---LGEYDYTFERLSHVIEALVDDLGVQRYVLYVTDFATPVGYLMAMRHPERVLGLVVQNGNTH------------------------------EAGLNDAWDAPRRFWQDPSEQNREALPEWLNFDGTREQYLGG-------LPEQLHELHPPESWHLDWERMSRPGNLDVQFqfYDYRNHVARFPQIAAYHQQHQPPCLLIWGRHDPFFDIAEVLAYHQEMAVLDAHIFDGGHLVLETHAEEVAALLVTFVEDL-------------------------------------------------------    
 262 UniRef50_D2R4R2        Alpha/beta hydrolase fold p...   159     2e-37  1  13.4%  99:418     3:264     --------------------------------------------------------------------------------------------------TATIDTRTMAYHEAG--QGPALLLVHGFPLDHSMWNEQIASLSNQ-YRVIAPDLRGFGKSDGAGEVT------TMADFADDVAKLVDHLGiePIHFCGLSMGGYVAWQFFLRHRAKLASLMVCDS----------------------------------RAAADSPEAAEG-----------------RRKTASKVLAEGSAVVADAMLPKLFGEWVRSAMPEVVEATDRVMRRTSPVAVAAALGGMAARIDFTPHLAKVDLPTLIVCGEHDVISPLAEMKTIADAIPGAKfaVEGAGHMSPLEKPQIVSGAIREFLTSA-------------------------------------------------------    
 263 UniRef50_Q13J50        Putative alpha/beta hydrola...   159     2e-37  1  13.0% 100:415     9:275     ---------------------------------------------------------------------------------------------------TTVRGQTISYLELGDGP-RTLLLLHGITSDASNWLSTMPALAQRGWRVIAPDQLGFGQSSKPSVPVRP------RTLSDMVAPLLDALGLEMVSIVGQSMGGHVAGLFAAQYPERVEALVLVNAGYGLALPEVKDPRD---------LGHAVTPGGLWALNPATRDDSRRLLEM---------------------------------VFHDQGLVTEELIDGFYADRLGKG-DGAVIRSISESWARREDTLESAFTGLERRPVLVIQARQDKVAPYHLGRAIHEGIAGSRFVVldCGHAPPLEAPEVFNKTLTEFL----------------------------------------------------------    
 264 UniRef50_C1BDZ5        Putative hydrolase n=1 Tax=...   159     2e-37  1  13.4%  96:417    17:284     -----------------------------------------------------------------------------------------------VQRTVDVDGVATRVVEAGS--GPSLILVHGTGGHLEAYARNVRDLA-KDFHLVLYDMPGHGYSDKPDRP------YTIDYLSDHLVALMDALSIDRADLSGESLGGWVAAWTAAHHPTRVRGMVLNTPGNITNKPEVMAGLKESSMK---------------AVREASPQTVRARVE---------------------------------WLFHDTSLVTDELVGLRLRIYSQSGFETAMANILAVQDWEYRRPFVWswCDRILAPTLLLWTDHDPTAAVEEASLLETLISGSRlvIEGAGHWPQWEKPDEFNEAHIRFLKG--------------------------------------------------------    
 265 UniRef50_A5V750        Alpha/beta hydrolase fold n...   159     3e-37  1  12.4%  96:418     6:277     -----------------------------------------------------------------------------------------------EKRTVQVNGTPMAYVEQG--EGEPLIAIHGGgsGISNYRRNIAPLAVGGRRVIVPDLPGYGDS-----PSQPTADAIYEGFADNILGLMDALGIEKASFIGNSLGGGTTLSLALRHPHRVNRMVLMGPGGGYS----------LSPHPTEGLLRMVHFYEGEGPTREKLDKVLDLLVFD------------------------------------RSMITPDLVEERYKACVRPDTMANPP--LRGRGANPKDDLWRQPLNELQHRTLIIWGREDRVLSFDNALVFLKSIPNAELHVFptGHWVQWERADQFNDMVNDFLDRA-------------------------------------------------------    
 266 UniRef50_A9P0T9        Putative uncharacterized pr...   159     3e-37  1  10.9% 100:421   102:391     ---------------------------------------------------------------------------------------------------WEWKGYKINYVVEGDGRGPTLLLVHGFGASLGHWRRNIRVLAER-YTVYAIDLLGFGASDKPT-----DFNYTMEGWAELLLDFSRDVIQAPTVLIGNSVGSLACLIAGSEAPqlVRGIVLLNCAGGMNNKAIVDDWRIKFITPLLWLIDFLL-----------------------------KQRKIASALFERLKTRENLMNVLSAVYSNKASVDDELIEV----IKKPADYPGALDVFVSVVTGPPGPNPISLIPNISIPILVLWGDEDPFTPLdyFSSLPSLLPSVQFFILRggHCPHDDRPDLVHEKLLTWLDSLHAT----------------------------------------------------    
 267 UniRef50_A9WG96        Alpha/beta hydrolase fold-c...   159     3e-37  1  13.0%  96:418     2:275     -----------------------------------------------------------------------------------------------RSRWLRINGYQLHWIEAG--QGPAVILLHGFAGSCADWEPTVDWLARQGYRALAVDALGFGRSEKP-----VNAPYGLHLQSDLYAGLLTALGIERAVFVAHSMGGKYALATALRHPQRIARLVLIAT------------DGFIKPSPLTIIGGWPVLGETILWLSAHPAVVRAFLAAAF--------------------------------HNPEPYLTADLIERGRAAISGTANRRALTALSRRYAadLTLTGLRARLGELRVPTLLVWGEHDRVFSFDYARIANREIPGSQlvIPHCGHFPHIEAVRPFRGLLNGFLAPV-------------------------------------------------------    
 268 UniRef50_B5H2T8        Alpha/beta hydrolase fold-c...   159     3e-37  1   9.9%  62:415     6:325     -------------------------------------------------------------AAAPDTAPDTAPDTAPDTDPLSDVALARRLGPGFTSRHADVNGVRLHYVDGGT--GEPLILLGGWPQTWWQWHKVMPELARR-YRVIAVDLRGMGGSSKPED------GYDKKTMARDIRELIRHLGLTAATVAGHDIGAMVAHAHAANHPEATTRVVLLDVAH--PDASLSTLTLLPGPDQHTGSDIEAGARTYLWWFAFN---------------------QLRGLPEQLLEGRSRPLVDWLFGYMAHDPTTLDELSrySRAYSTPEAIRAGNRWYQAYNQDIDEERS---YAPLTMPLLALGGDRSNY------AYLRDLLpdVTVVEvdCGHYIPEEQPQAVIDALTAFI----------------------------------------------------------    
 269 UniRef50_C1E4Q6        Predicted protein n=1 Tax=M...   159     3e-37  1  15.1% 106:417    23:296     ---------------------------------------------------------------------------------------------------------DVHYVSAGDDTAPAVLMLHGFPDNHATWRGQIPALVRAGFRVIAPDLRGYGRSGKPAGV----DAYGPAPVTDDLAALLDHERVDSaaVVGHDWGAAVAYAFAARHPTKLQKLSILNGVH---------------------------PAVFLDFVLSNPTQFLKSYYIGFFQLPALPEFVLSLADFA--------MLRLVFAIDPAKPLARSDVDTLVEACSTPGAMHAALNYYRAAGPA---HSLWSDVGKCPVPVQVQWGARDraIPPADVAPLAKK---SVVSHPDATHWVQWDEPDAVSDALIEFLNE--------------------------------------------------------    
 270 UniRef50_Q1R166        Alpha/beta hydrolase n=2 Ta...   159     3e-37  1  10.7%  82:418    17:311     ---------------------------------------------------------------------------------DHAPGGRASAAPQVHYRVEQVGDVKVFYREAGDPAAPAVLLLHGFAASSYMWRDVIEALAD-GYHVIAPDLPAFGYTEAPARG---QYDYTFANLTKTIERFTDQLKLTRYAMAVHDYGAPVGWRLALAHPSRITAIISQNGNAYEEG--------------------LSAGWEPIRKYWNDPTAENRQALSDFPTPASIKWQY-------------------VEGVSDTSLVAPDAYTLEGAQILRPGMADIQLDLLLDYATNVEqpAFQAYFREYQPPLLAVWGEHDPFFLPPGAEAWKRDIPDADIRLYDTGHFALETHSEVIIPTIRQFLDDH-------------------------------------------------------    
 271 UniRef50_A7HQJ5        Alpha/beta hydrolase fold n...   158     3e-37  1  12.5%  99:417    10:293     --------------------------------------------------------------------------------------------------YIEANGLAFEVDMCGNGEKFA-LLLHGFPESKFSWRYLMPVLAQLGYTVWAPNLRGYGGSSRPKGRA----AYEMPHLLDDVAGLIDAARArpATLVAHDWGGVIAWTFALAKTRPLERFIVMNLPHPTLFTKG----------------------------LRTWAQFKRSWYVFFFQIPWLPERLMLARNAHAIAEAFRGM------AIDKSRFPDSVLDHYRKNAQIPGAMTAMINYYRANFRGTLPKEWTD-PPMLETPTLMIWGEEDAALGKELTYGTEELVRDITIrlPQVSHWVQQEAPEAVNAMVRAWIEG--------------------------------------------------------    
 272 UniRef50_D1C9Y3        Alpha/beta hydrolase fold p...   158     4e-37  1  12.8%  53:420    71:376     ----------------------------------------------------VIPVGGMLGIIADEATPDEAIQELIAEFEATFVPPAEAGDTGPVPEQVTVGDRTIRYLRHG-EDGPAVILIHGFGGDLNTWLFNQEALAG-GRTVYALDLPGHGGSSKDVGDG------SLDVLAGTVAGFMDALGIERAHLAGHSMGGATAMAVATAHPERVASLTLIASAGLGPEIN----------------GDFIEGFIAAGSRRQMTPVLTMLFADQSLVTRQFVEDTLRSKRIDGVDQALRTLADRLF----------------------PGGRQATV--------------LAPALAGLDVPVLGIWGREDQILPVDHANALPS---HARVEIIEggHSVQMEAASEVTRLIDRFLAEVDA-----------------------------------------------------    
 273 UniRef50_B8G7U7        Alpha/beta hydrolase fold p...   158     4e-37  1  10.9%  98:419    60:335     -------------------------------------------------------------------------------------------------RFVELNGVVHHYQDVGPSDGEPLVLIHGWDCSAFWWHHVIDPLAQAGYRVISYDLKGHGFS-----ANDPRQQYTVAGFSADLQELIRILDLGSVHLAAFSLGAFIGLHVAAHHPDMVRSLIFFNFSLLPYNKVASAFVPWLLDTVFNKVL---------------------------------------RPIERRNLWWLPFIYARLVMAQNTPPVSDIRLGTLALRYCDPAAVRVSA---TELSRPEILTAVAEQAKMIRQPLLLVAGANDPIMRPADGRKLVALTQNGslEVPKCGHLILFELPEHVIQIMRLFLKGVR------------------------------------------------------    
 274 UniRef50_B7K182        Alpha/beta hydrolase fold p...   158     4e-37  1  10.7%  99:415    16:292     --------------------------------------------------------------------------------------------------TWTWQGHRINYTVKGT--GQPILLIHGFGASIGHWRKNIPILAENGYRVYALDLLGFGGSAKPP------LEYTLELWCEQIKDFWQVHVDQPAVFVGNSIGGLLSLMMMATYPEITKGGILINCAGGLNHRPDenLPLRLIMGAFAKLVNSPVTGKFIFNSIRQKHRIRSTLY-------------------------------------QVYRDRNAVTDELIDILYQPSCDAGAQQVFASVLTAPPGPSPNDLLPQVQHPLLVLWGTDDPWTPITGAKIYQERaqNTQFYPInaGHCPHDETPTQVNQLILNWL----------------------------------------------------------    
 275 UniRef50_A5UVR1        Alpha/beta hydrolase fold n...   158     4e-37  1  14.6%  82:417    22:307     ---------------------------------------------------------------------------------TPLRERLAPADLPGVQHFVRVGEYDLHYTDEGPRDAPVVLLIHGFAAWAFAWRAQRSALIAAGRRVVTIDMIGYGASSRPAAPV-----YSTHDQALLILQALDILGITTFDVVGHSFGGRVAFQVALLAPERVRTIVAICPEAFTVGRPPIATFAQLPLIGLALSYYILAPSLVGVGLRSLSKR--------------------------------------------DDWLTDEVIAGYAAPLYVRG--TAAAQVWQARSPKDGSLPVPANLSSIRPPTLLLWGDGDTVFPVDEGQRLERILPDARLIveRTGHLPYEERPADVNEAIVRFLTG--------------------------------------------------------    
 276 UniRef50_Q5YZG2        Putative hydrolase n=1 Tax=...   158     4e-37  1  12.8%  98:417    10:281     -------------------------------------------------------------------------------------------------RETTVDGVRVAYVTAGR--GEPLVLLHGWPEDHRAWSHQLAPLA-AVRRVIAPDLPGWGASDR-----DVRLSHDYDSEVDRLARLLDALGLDRIDLACHDYGGFLGLGFLHRYPHRVRRFALLNSRAHGTFTWPYRL------------------------------------LFGAFTTLASHRATRRLLTVRAMYPLHRWGLRGYVRNGS---FSPGLLDDYLSMLRTPEGLRWYAHFWAGYRVRVR-PELAAGLAAVDCPTTVIWGRRDPAIPLRTARELASRIPGAHLVLLDADHFVMEQRPAEVTEALLAWLDR--------------------------------------------------------    
 277 UniRef50_D1YWR9        Hydrolase n=1 Tax=Methanoce...   158     4e-37  1  14.6%  97:418     2:260     ------------------------------------------------------------------------------------------------EEYVEVGGLRIHYMVEGSGSA--VILVHGNGLSAGQWKLNISPLA-KYYKVYAPDLPGFGLSDKP------DLDYGVDYYVGFLKEFMDSIGVHKASLVGNSMGGAVVAKFAARCPDRVAGIVLSDPT---------------GFVPESLSRNKELYNAFLGLMIRSRRLYCRPMLHNS---------------------ANMRLLEDTQLVTDSKESRDAFVKNCKSILRYDGSYAGTLMAISA-------------------PALIIWGEDDLLLPAEDAEKYRGLIAGSKvlIERSGHMPNVETHAEFNAAVLSFFGGI-------------------------------------------------------    
 278 UniRef50_A6EQQ1        Putative alpha/beta hydrola...   158     4e-37  1  14.5%  92:416    35:321     -------------------------------------------------------------------------------------------PPITTYNYENIDGVKLFYRESGSRENPTIVLLHGYPSSSHSYRNLIPMLST-HYHVIAPDNLGSGYSDHLD---PNTTDYTFDLLSEYTSKLLEKLKVNNYIMYMQDFGAPVGYRMMMKDPKRIDALIVQNANAYIEG--------------------------------LTPKRQDFFRTAQTDTSKEKYDFLYSITGKDAIINKQYLFD---LDSTNMNIQSPDAWTHDLSFLSSEKDRKIQVELFQDYYTnlRYPKWQKMLLENQPKTLIVWGKKDLKFNANGAKAYLKDLPNAELHLIDAGHFAAEEKTAEIAKHILTFLD---------------------------------------------------------    
 279 UniRef50_A6C3S0        Alpha/beta hydrolase n=1 Ta...   158     4e-37  1  13.3% 102:417    25:282     -----------------------------------------------------------------------------------------------------VNGIRMRALIAGS--GPPLLLVHGFPLSHKMWLPQIEYFQSR-HTVIAPDLRGFGESDISSGIV------TMEQHARDLNSLLKELNidPVVFCGLSMGGYIAWEFWKHFADQLHALILCDT----------------------------------RADADSEEGVNNRLKMADLVLRHGPEAI-STAMIPNLISESSQQKQPHISENLIAMIEAADSEGIAASQKGMAARS----------------DYSNQIENIRIPTLFIVGSDDRLTPPEIMLRMSSQVPGSKYFEVgvGHMAPMEAPEAVNREIDHFLTS--------------------------------------------------------    
 280 UniRef50_Q98E28        Mlr4436 protein n=1 Tax=Mes...   157     6e-37  1   9.1%  76:426     2:309     ---------------------------------------------------------------------------PATAHAAPSIGADYGVPAGFSSRFATANGVRLHYVIGGS--GSPVLLIHGWPETWYEWRKVMPQLA-AGHTVIAVDLRGFGWSDVTAS------GYDRRTLAEDLYQLMRQLGYPKATVVGHDWGAPVGYAYAATHRDAVDRLVIAEGAPDGPWTHQQ--TTPFAHNPFWFFGFFEIPNYAETVLAGRETPFLDWFYRNRGF------------------------------HVVPGGFSDDDVAYFEEAYARPGRFAASLNLYRTIDQDIADNAVLSQSP-LTIPVMAIGADHGIGQGVPAG--MRHVASNVTPVLMGagHFLTEERPTAFVEIVETFLAGRPVAPAWAP-----------------------------------------------    
 281 UniRef50_C7I3I6        Alpha/beta hydrolase fold p...   157     6e-37  1  10.2%  96:418     4:296     -----------------------------------------------------------------------------------------------HHDVILSDGQRLHVASSGKVGRPLLMFAHGFPECWAAWQTQLAAFGQDHF-CLAPDLRGYNLSGKPEGIA----AYRVQPLMDDFLGLMRHFGYEQCVLVAHDWGGAVAWSLAAREPRSLSKLVIINSPHPIP---------------------------FARALAYNPKQQAASQYML-----RFREADAAEFLLQDDCARLLALFRHPITG--KSALDETEIALYKQAWNRPGAMDAMLNYYRASPLVPPTAGQAGAAglVPMPTLVIWGEQDQALLPVLLEDLGAVVPDLhyRVPGASHWVVHEEPDAVEAAIRSFLAAT-------------------------------------------------------    
 282 UniRef50_P53750        Uncharacterized hydrolase Y...   157     6e-37  1  12.7% 103:417    15:289     ------------------------------------------------------------------------------------------------------DGVKVWYREAGAAGNPTILLLHGFPTSSNMFRNLIPLLAGQ-FHIIAPDLPGFGFTETPE-----NYKFSFDSLCESIGYLLDTLSIEKFAMYIFDYGSPVGFRLALKFPSRITGIVTQNGNAYEEGLDDRFWGPLKEYWKSY---------------QSDPVFVKSLIPYLEDPANVICQYHDGVPAIE--------------------SVDPAAYTLDIALIQRTGQTDIQLRLFFDYQNNilYPAFQKFLRDSKIPVLVAWGANDTIFSVAGAEAYRKDVDNLKVVYYDTGHFALETHVVAIAEEIISMFAE--------------------------------------------------------    
 283 UniRef50_B0DKL8        Predicted protein n=1 Tax=L...   157     6e-37  1  12.9%  48:434     8:375     -----------------------------------------------LMKRLLLSTALVASVVMASFKPANYPKSSVT---------CKATRRAPVKEEVD---ITLKYVEVNPSAPTTIVMVHGWPSLWSTWSNQIQEFQGD-YHLIVPDLRGFGESTHPGDTKSSG---TMGDMVGDLVCILEQAKVHSAICMGHDWGSTVCYEAARARPDIFTAVIGAVVPYIPSAGSFVPIKHLTKALptYQVYFDAQTESAVAELDANIRRATRATLRTAAIPPPDAFLQSSESFLAAWDDHEELSPVAF--------FSQEEEDYFVEQYGHQGFKYTLQFYTQENRFQSWELAHSQGNHTIPQPVLAIYPKQDPVANWTVVAQLLkfLPTLTTQLMDGAHWPHLENPREFNAFVRKWLANLSIASNDKTEIPHGDEL---------------------------------------    
 284 UniRef50_Q0SH24        3-oxoadipate enol-lactone h...   157     6e-37  1  11.6% 111:471    13:313     --------------------------------------------------------------------------------------------------------------RSGAADAPVVVLLGSLGSNRSMWDPQIAALSYEC-RVVAVDQRGHGESPAPDGP------YSVRDLSEDVL----------------------------------------------------ALLDSLGVDAAHFVGLSMGGAIAQWLGAHAPRRVLSLSLLCTAAKFGEPQAWIERAAASRTDGPESLADAVVARWFslAKRDPEFVRHYREMIASTSP-----EGYAACCDALADWDFTADLSRISAPTLVIAGEEDPSTPPSVMQILADGITEARFEVlpAAHVANLEQAGAVTALLREHiaGYARGRRAAHAQGMTVR-RSILGDAHVDRSVAGTTDFTAPFQDFITRTAWGDIWS--    
 285 UniRef50_A4CMZ6        Putative epoxide hydrolase ...   157     8e-37  1  11.5%  94:415     1:275     ---------------------------------------------------------------------------------------------MTTFRQINTGTLEFDCLEAGDPAGEAVIFLHGFPETHFMWRRLMAELASEGFYCIAPNMRGY----SPNACPKGVAHYGIRHLRQDVADWADALGIDRFHLVGHDWGAAVGWQATFHFDQRILSWTALSVPH---------------------------GRAFAKALKTDKVQRKKSSYIRWFLLPRIPEWMIRKDDFAKFRR-------------LWKRSAPEEVENYLSVFRRKASLTAALNYYRANFGRRKMKPTGP----VSVPTLFIWGNRDLAIGAFAARANANYVDgyTFLEIDGGHWLIQENYPEVARAVRHHL----------------------------------------------------------    
 286 UniRef50_A7HTF1        Alpha/beta hydrolase fold n...   157     8e-37  1  12.2%  45:454     3:357     --------------------------------------------ILKGVGILTAALLLIMLVSFWAAGRARPILDEAARSSLA-------AKGLAYSFIETADG-TVHYRLEGPEDAPLVVLVHGFSTPSFVWDNYFKPLTESGYRVLAYDNFGRGLSDRPRAV------YDAELLDRQLVNLLDALKVdrIDLVGYSMGGAIATIFTARHTERVRSLTLIAPAGLGVAMD------------------------------DNVELLKRPLLGDWIVR----------------LFGTRLFYSAAAQEAKSAPNPGAFLEGFNRQLEYRGYGDALLSTMRHYPLAASEASF-AKVGRSAVPVLVIWGEADEIVPYEYSTRLMELMPEAQLryPAAGHNITFAQPELVSGLLLDFLrvTSAGAGGKPRGPLARLEPRKCG-SCSKDSADTAGDMT-------------------    
 287 UniRef50_D2MI58        Protein containing alpha/be...   157     8e-37  1   9.7%  67:417     6:308     ------------------------------------------------------------------IVSAAAPIAVVTTASPLCNPSRVDLLHRHTVHTTIVNGHHLAYLDHG--KGPPAILIHGLGGSMWHWEHQQVSLA-RSCRIMTPDLLGSGLSEKPEGI------YSPAFLLDTFHTFMDHLRIEKAVLIGSSMGAGIAIGMSLEHPDRVAKLVLIGGFPAN-------------------ILDNMQSSRTKRFIKHRPA-------------------LWLSKLGSRITGRWSIKLILKEIIHNQALISPMVVERVHRLRFQPGFFQAMYSQLDQIP--VWETTFAQRLADIPHATLILWGAYDKVFPLTVGQTLHATIPHSslVAPNSGHLPQWENPDFVNSALLKFLDG--------------------------------------------------------    
 288 UniRef50_A6FNN3        Haloacetate dehalogenase H-...   157     8e-37  1   9.0%  95:416     7:289     ----------------------------------------------------------------------------------------------FTDHTARVNGVTIAYSSGG--DGPPLALLHGFPQTRAMWAHIAPGLA-RTHRVICPDLRGYGASSKPDDV----GEYSFRQMARDICALMDQLGHARFALAGHDRGGRVAHRLALDAPHRLTRLCVMDIVPTHTL--LADLKVAVARAYYHWFFLAQPAPFPETMIGHDPDAYFQSCLLG-------------------------------WGGARLTDFDPGQLAAYRAAWRDADTIRGMCNDYRAALDHDVTDDACDLNQRIACPFLVLYGADGAMARqdVAATWEAKALETQAQTIPGGHFFPDTAPKATLAALSAFFD---------------------------------------------------------    
 289 UniRef50_B0TAQ6        3-oxoadipate enol-lactonase...   157     9e-37  1   9.5% 102:418     6:282     -----------------------------------------------------------------------------------------------------ANGVRLSYEVYGPEEAPALLFTHGAGWDHRQWLPQVEPFS-RHYRVILWDVRFHGRS-------GCCDLADLDDFSRDQMALLDHLGVEQAVLVGCSMGGHISLRTAILYPERVAALVLMGTPVTSAYNWRNRLAITLQEGSLKKFFSELIDISLQGFSFFFYGLTLRFATMEKIASMHAKGLSLFHPDLEEYFYGVT--------------------------------VQHPKERWNLIWEIACRMETLADLSKVACPTLVIEGDADWMMRRQH-RFICEAIPQACHriPDAGHATNLDNPEAVNDCIADFLRQT-------------------------------------------------------    
 290 UniRef50_D1B8Z4        Alpha/beta hydrolase fold p...   157     1e-36  1  12.3% 104:420     6:265     -------------------------------------------------------------------------------------------------------DVQLHYEVHGS-EGPPLLMIAGLASDVSSWQSVLPELSKR-FRVILVDNRGVGRS------KPEDPEISIGLMADDCAALIDHLGYGPVHVLGHSMGGMVAMDLAIRHPQKARSLVLAATGDRVSGRNAMLFSDLADRYDRGGDLSDF--------------------------YRTILYWILSPPFFED------------------RGMVDEAVD-YLLAYPYKQSPRSFRGQVEAITSFRGLD-----LGRIQCPTLVLAGERDLLFPLEEARDLARRLRGAQIRVlgAAHSVHEERPAEFVAAVEGFIKGVDR-----------------------------------------------------    
 291 UniRef50_Q7SHI0        Predicted protein n=2 Tax=S...   157     1e-36  1  10.4%  81:416     3:339     --------------------------------------------------------------------------------PDALTPHDPRV----THHYITIPslTYHYLLANPDSaaTVLLLHGWPDLSLGWRYQVPFLLSLGLRVIIPDMLGYGLTSAPSSPS----EYSLKNLSFHMTHIIRQVNpqPVLLGTHDWGAFLGWRLALYYPELIKGIFAFCIPYSPPETKVTSLEEHIERHPELGylQNARGEVEEAVNGGSKERLRGWLNAMFGGLAKEDG------GVMAFDPWKGVDVAKLEEVGGSPLVSEEVMDFYVEEYSRNG-IRGPMNWYRTREINLEDELPLAEkwQFQVPAMIVMVGHDPALPPELTDGMEKYFAKgkEVIPEASHWVLIHTPEEVNKLVGEFLQ---------------------------------------------------------    
 292 UniRef50_C1VD75        Predicted hydrolase or acyl...   157     1e-36  1  12.0%  92:417     4:284     -------------------------------------------------------------------------------------------PHGATRRETALDGLRLSALVAGDPASPPVVLIHGGGLDSasWCELIPALTDD-YRVYAIDLPGYGHSDEPERVP------TTDYYVRVLERFLEAEEIDAPALVGVSLGGGVALGYALGHPEDVSAVVAINS---------------------YGLGDSVPGGPLGALFVRVP--------------------YLSELSWRAIARSRTVAYFAVRAIVAYGNVRPHVVDQVYEEAQRNDGSAWRTFQRAEIGFTGLRTNYVDDLPNLSMPTLFIHGEDDKLVPSSWSVRAESLVPNSEVRipECGHWPPREQPQRVNSLVRLFLQS--------------------------------------------------------    
 293 UniRef50_Q5R0U5        Alpha/beta superfamily hydr...   157     1e-36  1  12.3%  99:416    35:310     --------------------------------------------------------------------------------------------------YEQVDNVRVFYREAGNENDPTVLMLHGFAASSFMWRDVIDALADD-YHVVALDLPAFGFTEV---SGDDEYSYTFANLTETVEKFIEQKELTKFALAVHDYGAPVGWRLAVDNPEKITAIVSQNGNAYEEG--------------------LADGWKPIKKYWNNPTQENREALSDFPTPESIKWQYEE-------------------GVSDLSKVSPDGYTLEGFHISKPGMADIQLDLLLDYASNVEqpEYQAYFRKHQPPLLAVWGKNDPFFLQEGAKAWKRDIPDAEIHLYDTGHFALETHHSVIIPVIKQFLD---------------------------------------------------------    
 294 UniRef50_O06734        AB hydrolase superfamily pr...   157     1e-36  1   9.4% 105:414    11:265     --------------------------------------------------------------------------------------------------------VTLFVEDIG--HGRPIIFLHGWPLNHKMFEYQMNELPKRGFRFIGVDLRGYGQSDRP------WEGYDYDTMADDVKAVIYTLQLENAILAGFSMGGAIAIRYMARhgADVDKLILLS--------------------------------------AAAPAFTKR------PGYPYGMRKQDIDDMIELFKADRPKTLADLGKQFFEKKVSPELRQWFLNLMLEASSYGTIHSGIA-----LRDEDLRKELAAIKVPTLILHGRKDRIAPFDFAKELKRGIKQSELvfANSGHGAFYEEKEKINSLIAQF-----------------------------------------------------------    
 295 UniRef50_D2Q3L3        Alpha/beta hydrolase fold p...   157     1e-36  1  12.3% 109:417    19:291     ------------------------------------------------------------------------------------------------------------YRVGG--DGPPVLLLHGFPQTHACWHQVAPELS-KTHTVVVPDLRGYGGSS--PARQDSVAAYGKRAMAKDQLELMEALGHDRFSVVGHDRGGRVGYRLALDHPERVDKLVALDIVPTADMWAAAAGEPEEKLEDWHWNVLSRPEPE--------PENRLTPDYLFPDGSPSISPHEIA--------------------------LDPDALADYLSALDRPSVRHAMCQDYRAGATVDRELDEQDLarTIECPVLVLWGSadLPQWFDPMQTWSRWAPNLQGEGLGCGHFIPEESPKTLLDRLTTFLDA--------------------------------------------------------    
 296 UniRef50_A0QXG0        Hydrolase, alpha/beta fold ...   157     1e-36  1  12.9% 103:419     7:286     ------------------------------------------------------------------------------------------------------GNLEFEVSEAGPSDGATVILLHGFPQRNSSWEPIVGRLTAHGFRCLAPNQRGY----SPGARPPRRRDYRVGELVEDVVALIDASGADRVHLVGHDWGAAVAWGVASYVPARLATLSALSVP--------------------------------------HPQAFLRAMTTSRQGLASWYMYLNQLPRLPERllGRDGGAARMVRTLQRSGQTREIAERDARAMAEPGALTAALNWYRAMFLSTSDPRAKhaKRKITVPTLYVWSDRDIAITEKPARETANYVAgyRFETLHgsHWIPEERPDEVADLLLEWFAAHP------------------------------------------------------    
 297 UniRef50_UPI0001B580CE hydrolase n=1 Tax=Streptomy...   157     1e-36  1  12.8%  97:416     2:264     ------------------------------------------------------------------------------------------------QTVLLPSGARIAYDDLGPQSKIPVLLVHGHPFDRSMWGPQADYLARNGHRVVVPDLRGYGESTGPAVNG-------LEDFAEDVLGLADRLGLGRFVLGGLSMGGQIVMQTIRDYPERIAALLLADT--------------------------FAEPETAEGKVTRNQAADRVLAEGMTAYADELLPKMVSPGTFRENPEVTGHVQKMMRGAPAAGAAA-------------------------ALRARAKRPDYRDSLKQVSVPTLVAVGSEDEFTPVADAQLLHQLVGNSELVVLdaGHLPNLERAAEFNDVFGRFLD---------------------------------------------------------    
 298 UniRef50_B5JX61        Lipase 3, putative n=1 Tax=...   156     1e-36  1  12.4%  97:419    43:310     ------------------------------------------------------------------------------------------------EHRIELGDYEYHYLDNEKTQKPLIVMVHGFTADKDNWGRM--ALFMRDYHIIALDLLGHGDSSRPA-----DGDYTIEAQVARVKAFADALDLPKFHLIGNSMGGWISGVYAAKHSDDLLSVTLLDNAGVEQPNPSDMMQRLQAGESIPLV----------------------------------------------MDGPDDADLFFEFAFEEPPFMTPGVKKAYAE---RSAQHAELYRYIHDQFTSENNPMLTPMLSQIDVPVQIIWGDKDRILDVSSIDVMTPELEGETVVieNCGHVPMLERPKETADHLDAFIQSLD------------------------------------------------------    
 299 UniRef50_UPI0001C319BB alpha/beta hydrolase fold p...   156     1e-36  1  11.5%  96:417    12:291     -----------------------------------------------------------------------------------------------THRDVTVRGIRLHVAEGGPQDAPPVLLLHGWPQHWWMWRDVLVDLA-RDHRVIAPDLRGLGWSDAPR------RGYVKQELADDNIALLDALGLDRVDLIGHDWGAFTGFLICLTAPERIGHFLGCSIPHIWPPRERPSLRRLSQL---------------------------------------WYQVVLAAPGVGQGLMRQGELTRKVLTAARThrYSDYELEQFVSVLKQPEHALASAQYYRSFLLHELKPLASGAfrPLSTKTLLLWGTDDPILRGAKTDEHRAFAPEMEirVPDTGHFLPEERPGLVVERARALFAR--------------------------------------------------------    
 300 UniRef50_Q082C7        Alpha/beta hydrolase fold n...   156     1e-36  1   9.4%  96:415     8:327     -----------------------------------------------------------------------------------------------EHKNILVDNVNIHYVESetALRPTIIFLHGFPEYWGTWSAQLNFFSP-YYRVIAPDLPGYNLSDKPDDVSF----YAVPNLIGFMAKFIAAISpqPVILVAHDWGGAIAWPLAAFHAQLISKLIIVN--------------------------------------AAHPSTFTREMMNNPLQRKKSAYIHQLISASGESLLTQDDYreDIMVSTNPSVFTADVKARYKQVWGQQGAINGMLQYYRAMPQLAENDTDNQRCkrINVPTLILWGEQDLAFVNENLDDIHHYVPNCIIKRfdTSHWLQHERPDEVNVAINNFI----------------------------------------------------------    
 301 UniRef50_A9B1M2        Alpha/beta hydrolase fold n...   156     1e-36  1  13.2% 101:417     5:275     ----------------------------------------------------------------------------------------------------TVNDQSIHVWEEGASDKQVALLIHGWSSSWFAMSPLFPILSR--YRCLAVDLPGYGQSPK------GDDPVSIVAYTDLLADLLRQVTDQPAVLIGHSMGGMISLTMSLRHPELIERMVLICPTISGRLSMFINM-----------------------------------FLGPLVVLERVPFADRVTSLIETRIWRITDRVMKPASYAERSAISSEAHERIKADVRRPGQGQIRAECYRAMREN----DLRGQLQKISHPSLYLWGMEDNTVPLRDASSVVAELPDADLriPNAGHWPHFEALVTTQRYVRAFLST--------------------------------------------------------    
 302 UniRef50_A0YFT3        Alpha/beta hydrolase fold p...   156     1e-36  1  10.1%  99:416    40:330     --------------------------------------------------------------------------------------------------YAEFDDVRLHYVTTetEEKTLILFLHGFPLFWYSWRNQLEAFGED-YLAVAMDGRGYNLSSKPDNVS----DYTLDKLVEDVRQMAAHlgDKKFILVGHDWGGGVAWAFAQAHPDKLHSLVVENAP------------------PFNLLLSLLQTHEEQRKASTYMERIKSQQGMDALVADDFAVM-----------------VGFFDRQVDQGIVSAADIAMYKKAWGQPGAMNSSINWYRANLPDPdaDFWPSREARVdvPTLLIWGEEEAVFTKDFLTIIPGYVDNLTivIPQAGHTPSTERADLFNKTMRSFIE---------------------------------------------------------    
 303 UniRef50_B2IDA7        Alpha/beta hydrolase fold n...   156     1e-36  1  11.8%  53:415    12:322     ----------------------------------------------------LIGTSGIIFGPALAMMCAPGAMAQSMALRKAPDATLPKGG---TSQFVQINGIRLHYVSTGS--GPAVILLHGWPQTWFAWRATMERLSS-HFTVIAPDLRGVGLSERTPT------GYDKRTIASDIAALIGHAAGGRAHVVAHDMGGKVAYMLANLHPESVEKLVLVDCLIPGTEN-----MDALRGGAWHYGFHMAPEIPEMLTKGRERDYIAAQIR---------------------------------AWSYKKDAVSEMAISEFARHYASPGGMTVGFAYYRALRDDAALA-ASFEGRSIDMPVLAIGGRHG--VGTKLADALSKQARDLTAVieDSGHFVADEMPDFFCDQLKRFL----------------------------------------------------------    
 304 UniRef50_A9SX85        Predicted protein (Fragment...   156     2e-36  1  14.8%  91:421    25:373     ------------------------------------------------------------------------------------------AHPPSDSKTLRHNGEEVNKATTRERNNLPAVLLHGFGASLFSWQRVLKQLAAidSSVVAFDRPGFGLTSRPKPLAGPAepYSVKFSAKAALSFAEFLHSEQVILIGHSAGCLVAFQAYLDAPEKVAAMVFIAPAIAAPIVMGEIMKATSASIPVLKALQIITAAVQALFQMLLPENFVKNVGSGISSVRDRLVTAVIRSSLTVWLVVRVVMDRFGTTGVRMAWYDPfvLQGYTKPLQCKDWDKALLEFVLAMAVSPSAspLGKRLKEVTCPVLVVTGDTDRLVPGWNARRLADALPNAEFALikCGHLPQEETPDELLTIIERFIAKTFAS----------------------------------------------------    
 305 UniRef50_C5CVP1        Alpha/beta hydrolase fold p...   156     2e-36  1  14.9%  83:418     5:300     ----------------------------------------------------------------------------------SSTPTLTADAHAVVHRFADLDGMQVFYREAGPRDAPVLLLPHGYPCSSFQYRRLMPLLADR-WRLVAPDYPGFGLSDTPPAEA---FGFDFDAYAGFLEAFCRKLGLDRYALYLHDYGSQIGLRLAIRHPERVAAVIIQNGDIY---------EDTLGPKYRAIQAFWRDPSASNRAVLEK----------------------------AVNEGGFRAEFVGEVPEAQAAQIPPDLWKLHWPLMNTPQRRELARQLMEGLKENLEwpRYQAFLREHQPPALIVWGPNDGYMPEASARAYLRDLPKAELVLTDGGHWLLETHLAQVVPVIRRFLSAA-------------------------------------------------------    
 306 UniRef50_A4JJ64        Arylesterase n=2 Tax=Proteo...   156     2e-36  1  12.5%  97:417     2:273     ------------------------------------------------------------------------------------------------SRITTPDGTEIYYNDWGT--GQPIVLVGGWLVSADMWEYQAHFLASNGFRVIAYDRRGFGRSSQP------WSGYDYDSLTSDLDELIQQLDLTDAILVGYSMGGgvARYIGTRGSKRIAKVVLLSATTP--------------------------------------------LLMAGADNPAGVPRDIFDGLRAGVLHDRADFLVNFGRAFTGADHDASVVTQAMLDWTFNMAITASIKATHDCIASFSETDLRPDLKKFDIPTLVIHGGADPVVPIDLSGKvaELIPGARLVVYEGahALYYTHKDRLNQDILDFARS--------------------------------------------------------    
 307 UniRef50_P0A572        Uncharacterized protein Rv2...   156     2e-36  1  12.4%  94:449    16:325     ---------------------------------------------------------------------------------------------PPTLQFRTVHGYRRAFRIAGS--GPAILLIHGIGDNSTTWNGVHAKLAQR-FTVIAPDLLGHGQSDKPRA------DYSVAAYANGMRDLLSVLDIERVTIVGHSLGGGVAMQFAYQFPQLVDRLILVSAGGVTKDVNIVFRLASLPMGSEAMALLRLP-----------------LVLPAVQIAGRIVGKAIGTTSLGHDLPNVLRILDDLPEPTASAAFGR--------------TLRAVVDWRGQMVTMLDRCYLTEA-----IPVQIIWGTKDVVLPVRHAHMAHAAMPGSQLEIFEggHFPFHDDPARFIDIVERFMDTTEPAEYDQAALRAL--LRRGGGEATVTGSADT------------------------    
 308 UniRef50_A4IJ41        At3g10840 n=4 Tax=rosids Re...   156     2e-36  1  13.8% 119:415   132:445     ----------------------------------------------------------------------------------------------------------------------PMILLHGFGASVFSWNRVMKPLARlsSKVLAFDRPAFGLTSRIFHpaKPLNPYSMVYSVLTTLYFIDVLAADKAILVGHSAGCPVALDAYFEAPERVAALILVAPAIFAPRPVATTDAGENRDKEAPTSNFLGTLVELTKGVIRAVLRVVTGMANMLSSLYKKALAAFLRSFLGVMLVRMagVTAVRNAWYDSKQVTDHVVQGYTKPLKAKGWDKALVEFTVATLTDNNGSelSKRLQEIKCPVLIVTGDTDRIVPAWNAERLARAIPGSVFEVikCGHLPQEEKPDEFISIVAKFL----------------------------------------------------------    
 309 UniRef50_A4INN3        Hydrolase, alpha/beta fold ...   156     2e-36  1   9.1%  47:418     6:353     ----------------------------------------------RRFFIGAVFAILLFVIGSYFYTNVTYMDKPLKAMRKA---------GFTEKQVTLKDGTVLNYGEGPDNGKPPLLLIHGQGMTWEDYAKSLPALSER-YHVFAVDCHGHGESDW------NPEKYSVKAMAADFAEFIKTVIGDKVILSGHSSGGMVAAWIAAHYPELVLGLVIEDSPFFATEPGRREKTFAWVYGFQQYEDFKKQDEIDDYFEYSLKHSYWKKVFGDFL------WNRFAKDAVAYHKKHPNEPVHLFYLPPNINRIFESATYPYDRRFGETFYDNSWFEDY----------DQAEVLAKIECPTVFVKadGdaALTDEDAQRVVELLQNGKLVKVDtgHDIHYDKPVEFTNIMIDFLDEV-------------------------------------------------------    
 310 UniRef50_A4XYI7        Alpha/beta hydrolase fold n...   156     2e-36  1  11.9%  97:415    39:310     ------------------------------------------------------------------------------------------------SRYVEIDGFNVHYRDEGSRDKPALVLIHGVVASLHTWDGWAEAFSP-HYRIIRFDVPGFGLT-----GPARDGEYSAERMIRVLGLLLDYLQVDKADIAGNSLGGYIAWNFALAQPQRVGKLVLIDPAGYPMAKVPWMI-----------------------AAAALPGA--------TVAMPLWMPRALIAQGIKEV-------------YGEPGRIKPGVVDRYYDLSRRPGNRRGMMDIFRVLlnRQELHTTPQRVAGIQAPTLLMWGERDRWISPRHVPLWQRDLPGIQvvYPGVGHIPMEEIPEQSAADAMRFL----------------------------------------------------------    
 311 UniRef50_B8FG90        Alpha/beta hydrolase fold p...   156     2e-36  1  10.8%  94:417     1:288     ---------------------------------------------------------------------------------------------MAVSRTVKsdGTRIHYRTEG--EGPVMCACNGIGVSTFFWNYLVKYFS-KSHRVIVWDYRFHGKS----GPGPDYDGLTMETNARDLLAVLDDAKADKAVLLGHSMGVQTILEFYRQNPDRCAALIPVLGAY-------GRPLDTFLGTDIFKYLFAFGFYPMFLFPGAAQKAVRTGLKVLFSDYVAWPGA-------------------RLTGLVNFQHIRKKDLHLYLDHLKTLD-----LRAFMAMAKHMQEHSARAILPEIKVPVLVIAGEDDLFTPLEISKDMHGMIPNSelVIPRGSHAALVEQPELMNLRIEKFLRE--------------------------------------------------------    
 312 UniRef50_C6J6U1        Hydrolase n=1 Tax=Paenibaci...   156     2e-36  1   9.4% 100:418     4:261     ---------------------------------------------------------------------------------------------------VTVNGTTIAYEQQG--QGETVVLLHGFCGSSAYWEKVQPLLAEQ-YQVIAPDLRGHGATTAPVG------AYTIDQMADDVAGLMEALGITKYTLLGHSMGGYTALSLAQRYADRLNAFGLIHSTGYPDSEEAKerLQAVSRIRSEGITHFVDGLVPGLFA---PDNVSKLGAEVDRVKEIGYKTPPQGATGAALAMRERPDRRDVMESTAL-------------------------------------------------PLLLVAGENDTVVP--MARLFTTEGPNVtaVIKGAGHMSMYEAPEQLTAVIADFMRSV-------------------------------------------------------    
 313 UniRef50_C7ZES2        Putative uncharacterized pr...   156     2e-36  1  10.2%  84:417     2:326     -----------------------------------------------------------------------------------AETTPPGLEGIVQHRYAQVGSHRYHYLHAKPSGKltFFLFHGFPDISYGWRYQITLLHSLGYEVVAADMLGFGRSSAPTDLK----EYAAKLHADEMATLAGRVAPgrIFAGGHDFGVFVAYRLAIYHPDLVQGLFVAAIPFLPLETKFEFADRIGDESHSAIGYMRQFGGPEFH---KNFVGQDKIWHLLSA-----CYNGTTPEGEPGGSPEVGLILDRLPRIGLSPIVTEEELAYIVSEYGDS--VQGPFNWYRLSKINFDADLPAAEKGNIrmPTLFVAGKDDPYLPPSITDGMKSYFDDLELHEVDGTHWVLWDARDKVNELLTKFLSR--------------------------------------------------------    
 314 UniRef50_Q2N7C0        Probable hydrolase n=2 Tax=...   156     3e-36  1  11.2% 100:402     4:278     ---------------------------------------------------------------------------------------------------VTANGITIHYDDHGPKDAEPILLIMGFGAQMTLWpeLVEALTGHGFRVIRHDNRDVGLSQKFDGVKAPgvPYTLADMADDSAGVLDALGIGQAHVVGGSMGGMIAQHVAARHPDKTKTLTSIFSTTGNPKLPPAKKE-------------------AIQTLITRPKSLEEEVLVEHGIKLSRAIGSPGYPAEEERLRERVREGVRRSVYPEGP--------------------------TRHLSAIVADGDRREMLKSITAPTLVLHGEDDPLVPVEGGRDTAAHIPGAKIKTIPggHDLPLE-----------------------------------------------------------------------    
 315 UniRef50_B9L566        2-hydroxy-6-oxo-6-phenylhex...   156     3e-36  1  11.2%  96:415    17:282     -----------------------------------------------------------------------------------------------RQYYVQAGGIRTRVLEGG--DGPALVFIHGTGGHLEAYARNFAGLTP-YFRMITYDMVGHGYSEKPDRP------YTPDYLAEHLIGLLDALGIERAHLSGESLGGWVAAWAAAHFPERVDRLILNTPGNITNKPDVM-----------QRVKESSLKAVREASYETVRARLEWLFY-------------------------------------DKSQVTDELVAIRFRIYTQPGFERAMENIVAVQDWEIRKQYAwsWCNKIRARTLLLWTDHDPTGGLDEAELLLQWIPGSRLHVidAGHWPQWEKPEEFNEVHRSFL----------------------------------------------------------    
 316 UniRef50_D0VYM9        Putative uncharacterized pr...   155     3e-36  1  10.0%  97:426    20:291     ------------------------------------------------------------------------------------------------RHVVQVGDHRVVYSEGG--QGEPVVLLHGFGASADSWNRFAKPLTKR-YRVIAPDQPGWGASTRIESAS-----YGYPAQVERLHQFLSTLGLKRVHLVGHSMGGFIASAYAARYPDEVITLGLIAPHGMVEPEPSELFRDVAKGDNW------------------------------------------------PVATTRPEFDRLLNNVFAKRPYAPKAVLNYLADHAIRNSAKSA-KIFAEMQTN--NPALADRLANITAPALIIWGDQDRVLHVSCADLFRQGIKSSEviIPGSGHMPLVENASACSRAWLAFIDKSRQARGAAA-----------------------------------------------    
 317 UniRef50_Q0BUS9        3-oxoadipate enol-lactonase...   155     3e-36  1  10.1%  94:471     1:311     ---------------------------------------------------------------------------------------------MFLRTGELAGDLNIHLTVDGPEGAPAFLLLHALGATGAAWDGMAAELA-RSYRVIRPDLRGHGLTEVTQGP------YSIDMLARDALAVLDALGVDEAHVAGISLGGMVAQMLAAIAPARVRSLILCDTG-------------------------LAMAEPSRFLDRAALARAEGMALLADQVLPGWV---------------TEEFLTDPASHGLYTMLCRTDPEGYAAA-----------------CEALSVADLSDHAEAILAPSLVLVGDQDRSTPIPMAEALRDRLGAAFTIIENAAHMICTEQPGQVVEAIHRFI---GAQAQADALTRGLATRRAVMGEEFVQGAFDHASPFERDFQTWMTEAAWGGPWS--    
 318 UniRef50_Q024P3        Alpha/beta hydrolase fold n...   155     3e-36  1  11.9%  46:416     1:310     ---------------------------------------------MKKWWFVVAAVAMLALVVVPRIIRYRNANLETRQLNSVVREKLPG------SFVQLPDGV-MHYEVAGPVDGRLVVLVAGFSAPYAVWDATFAGLTQAGFRVLRYDHFGRGYSDRPDAR------YDPEFFDKQLQDLLDALKvgKVDLVGEDMGGAIAAAFANRHPARANRLVMIDPGYRTGYEVPIRLRMPL-SRDYGIMLRASTMADAQMNDFVHPERFSSY------------------------------------------------LATYREQMRYRGFRKAILSTMLNYWVEDSTAEYR-QLGKIGRPVLLLWGVADESSRIELSAKVREDIPQAEFHVidAGHRPALERPEIVNPILIEFLK---------------------------------------------------------    
 319 UniRef50_B3T8K3        Putative alpha/beta hydrola...   155     3e-36  1  10.8%  95:418    56:330     ----------------------------------------------------------------------------------------------PVDRTVKANGLNFHYLEWGDPANPTLVMLHGVSQQAHSWDFVSLALSPD-YHVIAVDQRGHGDTDWAADGV-----YSLDSMQADIDGVVEVLGLNDFNLMGHSMGGRNSFIWASRHPGMLRSLTIVDTGPETQRRGQDRIRD-FRELPNNLDSFQEFADRVKEYTGRTEEQVLGALKYSIRQTPDGKWSWKWDPETRNRTRSGD-------------------------------------------DPNWTTEKLWECVQAVDCPSLVVRGSRSDIFAEETLAKMGQVMTDCTTETinAGHLVQGDNPVDFIAAARAILSRT-------------------------------------------------------    
 320 UniRef50_A3UC50        Putative hydrolase n=1 Tax=...   155     3e-36  1  11.7%  50:417     8:306     -------------------------------------------------GVILLAVLVIAFFAWTLRPLDAGAVQSQ--------------WMTEADRFVTIAGEDWRVRETGPENAPALVLIHGFSHSLESWDAMAAELDDR-YRIIRFDLPGHGLT-----GPRDDKAYAVPDTVAQVSALLDDIAPESFALGGNSLGGLIAWRYAADHPDRVTHLVLMDPGGYPNLGVGDEPAPIPDAMRAYLTLAPEAGVQYATSTL----------------------------------------------YADPSRVTPEQLERIRAMMRVEGNGQALIERIEQF----TLPDPNPDLARISAPTLILWGDSDAMIPATHGPRFDAAIPSSRLVLmnTGHVPMEEWPVETAALVEGFLAE--------------------------------------------------------    
 321 UniRef50_A8I0H8        Predicted protein n=1 Tax=C...   155     3e-36  1  12.0% 100:425    50:352     ---------------------------------------------------------------------------------------------------WEVNgrKVHYIKAGRTGDPVVVLVHGYGASSYHWRYNVPALAEAGYQVYAVDLLGFGYSEKAREDYTNGELWS-SQVAAFIREVVsaAGGAPVVLAGNSLGGYVSLATAaeGPELVRAVALLNGAG---------PFKESEEKAAAEAAEWEDGNWFAAAKRSVANAVKRGVMYFAFLRTKTPERIREV---------------LQMVYVDHSSVDDDLVTSIENPAKDPAASEVF--FLVSHSTRGPPRYVDGLLQRLQVPLLLLWGDKDPWITPARAQKIINLYPSAVKVGLNSGHCPHDDTPAEANAALLNWLSGLPPVGGAS------------------------------------------------    
 322 UniRef50_A1VS27        Alpha/beta hydrolase fold n...   155     4e-36  1  13.7%  97:417     2:273     ------------------------------------------------------------------------------------------------HYVTAGDGTQLYVKDWGS--GPPVILLHGWPLSSDSWDDQAMAIAEAGFRAIAYDRRGFGRSSQP------WTGYDYNTLTDDLAAVITQTGVQDAALVGFSMGGGEVARYMSRHAGRSVSKAALISSVVPY----------------------------MRQTDDNPEGTDQGVFVE----------MLQAMKDDRAQFFATFFREFFGVSLMSHPASDEVID-----WARSMAMQASLKATLECAKSFATTDFRPDLAAFKVPTLIIHGNEDNTVPIDagRAAAKGIANARLIEYDGahGVQVTHKDRLSDDLIDFLRD--------------------------------------------------------    
 323 UniRef50_A9KFS4        1,3,4,6-tetrachloro-1,4-cyc...   155     4e-36  1  14.7%  96:414    14:291     -----------------------------------------------------------------------------------------------ASRFVTVKGAKMHYIETG--QGEPVLFIHGMPTSSYLWRNIIPKLADKAH-CVALDLIGMGESDKP------DIDYTVNDHISYVECFIEALGLRNITLVMHGWGSVIGFDYARRHPKNIKALAFFESHIRPTTD-----WDMLSLPVQQLATLLHRPGASYRAI-----------------------------------VEQNYLINKLLPASMMRKLSDEEMANYRRPFLTKESRKPLWQYLQDLPLGDSKRPvsQWLKQAPQPKLMMYAVPGFVTTMAAVQWAKEHLPNLTLVEfdVLHFAQESIPDIFSEELRKW-----------------------------------------------------------    
 324 UniRef50_C0ZAH7        Putative uncharacterized pr...   155     4e-36  1  11.7% 103:417     9:262     ------------------------------------------------------------------------------------------------------NGIKIAYVEEGTGDS--LVLIHGFCGSSAYWHKLVPLLS-KTHRVIAIDLRGHGDSSAPDEP------YSIERFADDLALLVEELGLAKIHLFGHSLGGYVTLAFANQYADKLASFGLVHSTPYPDDDAAKANRDKGADnrQNGMEPFIKALVPKLFAPSHIDTMR----------------------------------------------------EEVQLAKEIGFATSPVGAVQTLIAMRDRVDRNHVLQETTLPVLLVAGTEDQIIPAEKTFTVDKNNVE-QVLLPDAGHMGMVESPEKMAEAFKSFLNR--------------------------------------------------------    
 325 UniRef50_P66778        Probable oxidoreductase eph...   155     4e-36  1  13.6%  90:456     2:335     -----------------------------------------------------------------------------------------PATQQMSRLVDSPDGVRIAVYHEGNPDGPTVVLVHGFPDSHVLWDGVVPLLAER-FRIVRYDNRGVGRSSVPK----PISAYTMAHFADDFDAVIGELSpePVHVLAHDWGSVGVWEYLRRPGASDRVASFTSVS---------------GPSQDHLVNYVYGG-------LRRPWRPRTFLRAISQTLRLSYMALFSVPVVAPLLLRVALSSAAVRRNMVGDIPVD---QIHHSETLARDAAHSVKTYPANYFRSFSSSRRGRAIPIVDVPVQLIVNSQDPYVRPYGYDQTARWVPRLWRRDIKAGHFSPMSHPQVMAAAVHDF------ADLADGKQPSRALLRAQVGRPRggDTLVSVTGAGSG-----------------    
 326 UniRef50_A8FU65        Alpha/beta hydrolase fold n...   155     4e-36  1  12.6%  79:419     7:323     ------------------------------------------------------------------------------TLRTPDDAFVGLNDYPFEANYRQVDGMRLHYIDEGKENAKAIFLLHGQPSWSYLYRHMIPLLVDAGYRVIAPDLIGFGKSDKP----MDSETHTYANQVKWMGDFVRQLGVNNASAFMQDWGGMIGLRVLAQQPQWLDRLVLANTALAEMSKLEKFMVPKILKLLA--------------------------VMAGKPSLEKFMGNINYGNWAGYFRHAESLQIGQIIQSLTTRVLNEDEMRAYDAPFPTPQYYAAPRKmiVASELDEVNADW-EKLKRWNKPMLTLFSDKDPFLADQGYdkFQKNFSGahMTVENASHFLQEDQSKLLATRLSCWLQQTS------------------------------------------------------    
 327 UniRef50_B9M4F8        Alpha/beta hydrolase fold p...   155     4e-36  1  10.6%  97:417    18:303     ------------------------------------------------------------------------------------------------RLTLQANGRYVHAL--GAGDGPLVIAVHGFPDLPISFRHQIPVLAANGYRVVAPCLRGYSESE-----PESNGPFEVAILIQDLLALIDRLTDQPVTLIGHDWGTSVVRGAAIMAPAKVARIVSMSVPT---------------------------AGNFSRALLINPVQQRRSWYMYFFQLPFAEMAVAQDDCT---------FIEQLWQDWSPGWQCpaIMDEIKAAFRQPAVLKAALGYYRsaLQRPELADIRKRLGdiPVPAMHLHGADDGCIGEETTTGMeaFIKHFEKHIIpaGHFVHQEQPDVVNRLILAFLNR--------------------------------------------------------    
 328 UniRef50_B2AVA6        Predicted CDS Pa_7_3580 n=2...   155     4e-36  1  11.1% 104:418    29:388     -------------------------------------------------------------------------------------------------------GLTFHVLKAGKPGKPLVLFTHGYPELAYSWRKVLPAVAEAGYFCVAPDQRGYGRTTGWPRRNFEDTdwSLTNLVRDLVCLVYKLGYTQvsIVGHDFGAVSAAVAALIRPDMFLSTVHMSHPYHPPSTPPLSslAALSPPRKHYKWYNSTAPAAKDwlEAFLRGYFHLKSADWEKNEPYPLDAWTADQLAKMPEYYIMRRDQTMPTMVhrWLSKDEMELYCSEWKRTGF-QGALNWYRAQTVgaAGDMWLFAGKKIEVPVAFISGVKDWGNYQRPgeEWVkmFRGATLV-EGAGHWVQQEQPDAVIREILKFLQSL-------------------------------------------------------    
 329 UniRef50_Q9K3Q1        Putative hydrolase n=3 Tax=...   154     5e-36  1  11.3%  93:418     7:291     --------------------------------------------------------------------------------------------TPVRTGRAAVNGTSLHYRAAGS--GPAVVLLHGVPKTSYHWRHLVPKLTP-HYTVVAPDLRGLGDSARPAD------GYDSATMSDDIAELMNHLGHESYAVVGEDWGAVIGYQLAARHRDRVTALVFAEALFPG-----------------------FGFEDHTALTAENVAGGMHLWHLGFYFQPDIPEMLIAGH------ERELITYMIKFERSRPDSATPEAIDEYVRCYSMPGGIRAMLAVYRAMLVDAEQNRRAAR-KKLDIPVLALGG--SAFIGDRNESQMRLMAHDVTGHVFDAGHDLAEEVPDEMADVLLPFLATH-------------------------------------------------------    
 330 UniRef50_B5JRY4        Lipase n=1 Tax=gamma proteo...   154     5e-36  1  13.4% 106:426    62:331     ---------------------------------------------------------------------------------------------------------ELTYSVGGIAGRPTLVLLHGFSGDRNNWNRVAQQL-QQDYRLIIPDLPGHGESS-----LHPRDDYSSAEMASILRDFIDQLGIERYFIAGHSMGGGLAVQWSVFRPQQVQGLILINSAGIYEHNGSAVMAQIERGNNPLLVQKA--------------GDLKRVLDVVTYQPPFIPKRLLG--------------------------------EYEAQQIARAATYQKVMDSLMKTQEHLGASMFHRALAAIPSPSLVVWGREDAIFDVGVTEELLEALRDPTLVTFdvGHMSLLEAPWRTADAIRQFVEKTRAATGENA-----------------------------------------------    
 331 UniRef50_A0R6Z0        Epoxide hydrolase n=1 Tax=M...   154     5e-36  1  13.7% 103:415    15:284     ------------------------------------------------------------------------------------------------------DDLKMHYVRAGA--GEPVVLLHGWPQTWYAWRKVIPLLAGE-FEVIVPDLRGCGDTSKPSG------GYDKKTVAHDVRRLVETLGHSAVHVVGHDIGAAVAYAYAAQWPSEVQTMTFIESSLSGFGQEELMDVANGGSWHF---GFNMAGDISEDLVRG-----------------------------RERLLIEYWMRRTTVGVVDPTSVEPDALDEYARAMAQPGGLRGSFALYRAIPQDRADNRELGR-TKLAMPVLAVEGEHGQahSTETMKKVADHVTSA--VIARAGHYPAEENPAELAAALRGFL----------------------------------------------------------    
 332 UniRef50_D2BBB8        Hydrolase or acyltransferas...   154     6e-36  1   9.9%  55:457    14:383     ------------------------------------------------------AIVGVASAGLATAAFAKRYAVGRIRLRPDLEANEPLGerGRPTTVVTSDGVSLYAEVDGADDAptVVFCHGYTLNLDSWHYQRRDL-GDSYRLVLWDQRSHGRSPRTTA-----EDSTIDRLGDDLAEVIAALVPGPCVLVGHSMGGMTVMALADRHP------------------------ELFGDKIKGVALISTSSGKLAEISLGLPALLAKAVHKVTPGTVSMLSRGGTLVDRGREAGNDVAFLarHLGFGDSKNVSPTVVD-----FAESMIRSTPFEVIANFYPALMSHDKLNALSVldVPTSIMVGDKDWLTPVDHSRAMAEALPRAQfeVPDSSHLVQLERPTVVNDALRDLLKRAA---ADSAADGAIDKAADSATDGAIGNAADGGADRAIDG----------------    
 333 UniRef50_UPI0001B50386 alpha/beta hydrolase fold p...   154     6e-36  1  12.6%  96:420    13:287     -----------------------------------------------------------------------------------------------RHAYVQTGDITTRALQAGPEDGEHVVFLHGTTGHLEAFVRNIPAYVERGYRCHAIDMLGHGYTGKPDHPAE------IPAYVDHLIAYLDAVGARRAHLVGESLGGWVGSWLASEHPDRVASLQLLCMGGTKV----------------------------------NPEVMERLKTSTRNAVLNDDISDTRKRL-------------ELLWANWDEELGEELTQVRYEIYHHPDFQANVDNLLALQEVDRRERNlpDRMARIKAPTLVVWGNKNPFGAVPEAEAITEAIPGARLEIfeCGHWPQHEKAELYNPLSLDFLDEASR-----------------------------------------------------    
 334 UniRef50_B2MWN3        Soluble epoxide hydrolase-l...   154     6e-36  1  12.8%  19:415   157:547     ------------------RKPDPNIF---HEALARLGAEASQAVFLDDSEVNTKAA--EALGITSILVQDPKTALEQLKIVTGIdpITVHPDEVTHSYVTTRSKVRFHFTELGS--GPPVVLCHDFEEDWEAWRSLMPELAIAGFRAIALDLKGFGESSKPTDT----EQYTLKILCRDMTEFLDALGIAQVTLIGKGMGSAFAWTFANHTTDRVRAVAGINTSPSvpDTLYGDQIKRIGSLQDYIKFRHCDRNNNNNPDIEMEQFYRIATCHSSDTNPTKHDRCLQGIDVKDFELSTKASMNCLCIEADKRG--KKQLGGHHRSTKSKSKSRGA-NWFRNNAANWEW-NRRLNGRMLLIPALVVTSGQGSPKDVPDVSELKKWIPEVEhhVSGC-----eeRSSELNRILRKWL----------------------------------------------------------    
 335 UniRef50_B2ICN8        Alpha/beta hydrolase fold n...   154     6e-36  1  12.9%  51:423    13:431     --------------------------------------------------RAVLAMGAASLVAASLRPAHANPMG-GTDLTIWTYGEGTLPTGVRSRMIHGVNGLNVHILEAgsPSRPLALLLHGFPDLAYGWRHLIPILAEAGYHVVAPDQRGFGRTtwVNDYDAPLEPFSFLNMARDALGLVAALGYRraMLVGHDLGSPVAAYCALARPDVFPSVVLMssSVQPNTENQKLAAALAALDPPREYYQQYLSTRAANDDMWHpaFLRAFFYVKSADWPGNKPHPLKARTAAELARMPTYYVMDLGRTMPQtwLTEPELGVYTEEYDRTGFQGALQAYRVFSDPDLNAELRLFSGKTIDVPSLFIGGKSDWGTYSAPGakAATRMGGMEL-IDGAGHWIQQEQPVRLGALLLAFIKEVGGAGR--------------------------------------------------    
 336 UniRef50_A9W2P8        Alpha/beta hydrolase fold n...   154     7e-36  1  10.8%  96:420     5:294     -----------------------------------------------------------------------------------------------RHDTVTGNGMQLHVARAGT--GSPLVLLHGWPEFWLTWEPVMARLADR-FSLIAPDLRGFGASEKPDHGPSDKAG--PEVHAADIVGLLDALGLARAGIVGHDVGASVGQALGRSHPDRLTGLFFFDCPYPGIGPRLAQPEMLAEIWYQSFHLKPFAAEVVGASRESCRAYIGHFLRHWAGGNPSAF---------------------------------DAVLDAFTDNFLVPGNLQGGFNWyrQAGRLAMLRGEAPALPPIRVPTCIRWGTQDPLFPYAWTDRLGETFSNLDLafEGAGHFPHRERPEEAAAAIAAFFGRLEA-----------------------------------------------------    
 337 UniRef50_B3QMF1        Alpha/beta hydrolase fold n...   154     7e-36  1   9.9%  94:416    41:315     ---------------------------------------------------------------------------------------------MEKSHFVEVGGLLHHYHDSGSEQPrtVLLIHGWDCWWMWWHHIIRALNAEGYRTIAYDMKGHGWSE-----NDPENRYQIDDFARDLDGLVRAIGLEAFHVAAFSFGPFVALDYAHRYPNSVQSMTFFNFGYLPNNEVISKVAPVTLNFIFNQMMRKLTWWLPAYM-------FARLALSKNSIMMHDINVGFESLGLC--------------------------------------ASEAIEQTTRQITALETTEMLPEKVRALKVPVLFVAGEGDGIMRCENARKLQEIAESGtlCVPDCGHLITLELPETASEIILGHIS---------------------------------------------------------    
 338 UniRef50_C1XR65        Predicted hydrolase or acyl...   154     8e-36  1  13.8%  80:406     1:282     -------------------------------------------------------------------------------MSTTLPQFDVPESLRLYRREVTAGGLRFHLYDAGR--GPAWVLLHGLADEADSWRHVIPALA-QTHRVIAPDLPGFGRTERP------QRAYTPGFFVRAVAALLEELGLGEVALVGNSLGAEIAARLALERPRLVNRLVLVDGPSLGGGVSPALLR-----------------MLVPGLGERYYTRLRASQDEAYATLRPYYADLEALPPEDRAFLR----------------------ERVWARVWSDGQRRAFFSTLraLASLTGGSRFREALKHLQVPTLIVWGEKDYIVPVAVGQALAALIPKAKlvIPSCGHLPQQEKPEE-------------------------------------------------------------------    
 339 UniRef50_B1WUQ2        Esterase n=6 Tax=Chroococca...   154     8e-36  1  12.3% 100:415    23:294     ---------------------------------------------------------------------------------------------------IKVRGVD-HYYEwgIIEKPVMVFVHGWGGSARYWRSTAEALSD-NYDCLLYDLRGFGRSKLPENPLNLS--YDLEEYAEDLKALLDGLGLDKVYMNSHSMGASVGALFSTMYPEKVERSILTCNGIFEY----------------------------------NKLAFDTFHLFGGYVVKFRYNWFLKVP-FAPRLFMSRFLHRPIDKLEKIAFLEDFILADY----------EAALGTIYTSVSKKAVETMPKKFAEISVPTLLVSGEKDIIIPAAMGREAAKLNDKIEYVEIatAHFPMLEDKETYLSHVREFL----------------------------------------------------------    
 340 UniRef50_Q8YVC2        All2056 protein n=22 Tax=Cy...   154     8e-36  1  10.1%  89:415     6:307     ----------------------------------------------------------------------------------------APSTTPIPGQYWQWRGHKIYYVRAGepQRPPLLLVHGFGASTDHWRKNITGLCDD-FEVFAIDLLGFGRSAKPK------LQYGGDLWRDQLHDFISEVIGQKAVLAGNSLGGYACLCVAAQLPESAAGVVLLNSAGPFSETQATPEPEALQSQIQ-------------------PPKQSSPLQKRFGNSVKWIFRQPLAQFLLFQYVRQGWVIRRTLEKVYLDK--SAITNQLVEEIARPAYDVGALDVFVSVFSSPQGEKVDVLLKQLTCPLLMLWGEADPWMnrERSQKFRLYYPELTEHFLRAGHCPHDEVPNQVNPLLQEWV----------------------------------------------------------    
 341 UniRef50_Q2JLM4        Hydrolase, alpha/beta fold ...   154     9e-36  1  10.9%  89:426     4:299     ----------------------------------------------------------------------------------------TSASATPISAQWIWRGQSVHYVKQG-EQGQPLLLVHGFGASTDHWRKNIPELA-QHYQVYAIDLLGFGRSAKP------NWDYRAEIWRDQLRDFCQQVIRRPVVGIGNSLGGYVVLSFAAEWPEWVRGVVLLNGAGGFSTLKGSPTGWR----------QWIGGLVGWGL----RQRLVSYLLFQYLRQPHVIRAKLKQ------------------VYYDPAAVTDQLVEAIHRPTQDPGAADVFVALMRGGQKGRYVDEL---LCSLVRPLLLIWGERDPWMrrERSKLYRAHYPQAVEYFLEAGHCPHDERPEEVNALIHRWIETGIPATTSPP-----------------------------------------------    
 342 UniRef50_Q2HA22        Putative uncharacterized pr...   154     9e-36  1  10.1%  82:416     6:313     ---------------------------------------------------------------------------------DKLTPTDPRVQHHTYTIPASPHNTTYHYLLAEPPSGTpaLLIHGFPDLSFGWRYQVPHLTAQGLRVIVPDLPGFGRSDAPADIA----AYSYKRVVEDLLAVVRRVqvEKFVLGGHDWGGAVAWRFALWYPEVLRGVFSV-----------------------------QFEGTEVDEAVVGREKIRAFLSVMYGARREDGQPVFGVAKGLEVGLLEPAKI------GESPLVSREEMDFYADEYLKNG-MRGPLCWYKNGKVNFDEErlEQGRTKVTVPALMVVATRDAALPPAMSASMDDLCTDLVKKEVNASHWALWEAAADTNKHISEFLA---------------------------------------------------------    
 343 UniRef50_UPI0001C315CE alpha/beta hydrolase fold p...   154     9e-36  1  13.0%  96:418     7:290     -----------------------------------------------------------------------------------------------RHRSTEVAGVRVFYREAGPDDSptLVLLHGFPGSSQMFRHVIEPLADLGVRVVAPDMPGFGLSSLPE----PRGPYTFAWIADVVEEWLSGLGVTDKILYLHDYGSAVGYQLATRSPSTVRGLVVQNGN-------------------------------------AHEEGLGKQWDASRDYWSDPTEENRRRIEPWLHYEGTKHQYLWALPERLAELVPPETWELDWRAISRGDGLDAHWELFTDYRTHVArpEIARYHREAEPPTLIVWGVHDPYYDLDEVLAYHRVLPTAESHLLDAGHYLLETHSREFVSHARPFIARL-------------------------------------------------------    
 344 UniRef50_UPI00016C4973 hydrolase, alpha/beta hydro...   154     9e-36  1  11.9%  83:419    23:319     ----------------------------------------------------------------------------------PPFPNPAMPLFPFVSRVATRRGLRMAYLDEGRGD--TVVMLHGNPTWGFYYRNLVLSLRE-SYRCVVPDHIGCGLSDKP---GTDRYDYSLKSRIDDLEWLLEERGLTrlTLVVHDWGGMIGMGFAARHPERVKRIVAMNTGGFPLPAGKK-------------------------------------------LPPSLWLGRNTKLGAWLITRRNWFCKFAAKWCATKRPLPADVKAGYLAPYDTPAHRVAVLKFVQTIPLAPSDpaAASLEKFRDTPTLLLWGMKDFVFDRHFLEEWQRHFPHAEVhwPDCGHYLLDDAGELAITRVWEFLQRHP------------------------------------------------------    
 345 UniRef50_A4SZ48        Alpha/beta hydrolase fold n...   154     1e-35  1   9.4%  99:417     5:287     --------------------------------------------------------------------------------------------------YIQANKLQFAYQEYG--DGPLVILLHGFPDIATTWSHQVPALVAQGYRVVTPYLRGYTPTEIPEGGF-----YDKATLVEDIAGLIKGlgGKPVHLVGQDWGAIIAYAVLAAYPELISRAVVMAVP--------------------------------------HPGQVTESLVNPKHIHRSFHWWFFQLPDLPeiLANDQAFIDYLWAYWtpSHRDEEHIVAVKETLEKPRVLSATLAYYRAMfaDPALTAVREKmrLIQVPTLALCGRED--LRAELMLEQSQYFTnyEFKLVdaGHFLHREQPEAVTRLIIDWLKR--------------------------------------------------------    
 346 UniRef50_UPI0000E46F08 PREDICTED: similar to LOC52...   154     1e-35  1  16.3%  44:245    23:216     -------------------------------------------IILFLMFIMSVCVGMTFFLGALIF---KGPSWVFKKKQMDVRPDCLSEPSLGTHKFIALKDLKLHVVESGDSKNPLMLFLHGFPECWYSWRHQIRAF-NKDYHCVAFDMRGVGESDAPAGVS----NYTMDKLVGDVCDLIKVIGHSSCVLVAHDWGGLIAWEFAARYPDMVDKYIPMNISHPDRFVEYGTLLFRWACHGFR------------------------------------------------------------------------------------------------------------------------------------------------------------------------------------------------------------------------------------    
 347 UniRef50_Q6DND9        CurN n=1 Tax=Lyngbya majusc...   153     1e-35  1  12.6%  57:422    11:340     --------------------------------------------------------ATFAAGTAGLVAVAAGVPVAGRAQSQPPLQLPISSEFPFAKRTVEVEGATIAYVDEGS--GQPVLFLHGNPTSSYLWRNIIPYVVAAGYRAVAPDLIGMGDSAKP------DIEYRLQDHVAYMDGFIDALGLDDMVLVIHDWGSVIGMRHARLNPDRVAAVAFMEALV--PPALPMPSYEAMGPQLGPLFRDLRTADVGEKMVLDGNFFVETI----------------------------------LPEMGVVRSLSEAEMAAYRAPFPTRQSRLPTLQWPREVPIGGEPAFAEawLMASPIPKLLFHAEPGALAPKPVVDYLSENVPNLEVRFVGAghFLQEDHPHLIGQGIADWLRRNKPHA---------------------------------------------------    
 348 UniRef50_A9DHK5        Haloalkane dehalogenase n=1...   153     1e-35  1   9.9%  46:417     1:325     ---------------------------------------------MSNLLKIAAVTTLIALSSTLVVAEDAAPKLPAVAVSEELVID---------RREIAVLGSTMSYLEEGEGD--VVVFLHGNPSSAYLWRNVIPYVSS-THRVIAPDLIGMGESGKP------DIAYTFADHARYLEAFVDELNLPAITLVSHDWGSALAWDFARRNPDKVVRLAFMEGVLPP-------------AFPQDSYQAMGEEMGGMFRALRDPEK------------------------GREMVMEGNMFVESILPMMANRPLGEAAMETYRAPYSTVDSRLPTWMWPREVPINGMPadIQTFMGKTDMPVLLAYAEPGVLVPPQAVPFYTDLIDNLETAFVGQghFIQEDQPDAIGRAIADWLRR--------------------------------------------------------    
 349 UniRef50_Q1CVZ5        Hydrolase, alpha/beta fold ...   153     1e-35  1  15.0% 105:430     9:271     --------------------------------------------------------------------------------------------------------LSLYFEESG--EGTPVLFLHGLGSSGRDWESVAPRLTGR-HRVIVPDARGHGRSGKPPG------AYGVPRFARDIAGLCDALGLTGVHVVGLSMGGMMGFQLAVDRPELVRSLVIVNSGPELVARTLRR--------------KFEFGLRLTLLKLLGPSVLAKVLAPKLFPKPEQ----------------------EALRQRALEIFSANDPDAYLRATKGLVGWSVM-----------------RHLGGITCPVLVLASDRD-YTPVSTKQAYVDLLPNARLQVldSGHASPHDQPDKVADAVEAFLAGVEDAAA-DARQPG-------------------------------------------    
 350 UniRef50_C5D033        Alpha/beta hydrolase fold p...   153     1e-35  1  11.2%  95:415    48:320     ----------------------------------------------------------------------------------------------FRSGSAEVNGTRIHYRIGGS--GPTVVLLHGYAETGHMWNPLMPLLA-KTHTVVVPDLRGAGDSSKPET------GYGKKNMAVDIHELVRSLGIRSVSIVGHDIGLMVAYAYAAQFPSETDKVVLMD-AFLPGIGEWQNVWLLRDLWHFHFHGATPLALV--------------------KGRERIYFEHFWNDFAAD----------------SKHSVPEADRQFYARAYAQPGGMRAGFEYFKAFEQDAAEFAELGKTP-LPMPMLVLSGEkgGTFLIEQGKMVATNVQG--VLVKGSGHWLMEEAPEQVIPALINFL----------------------------------------------------------    
 351 UniRef50_A8N3E1        Putative uncharacterized pr...   153     1e-35  1  12.5%  98:418    43:360     -------------------------------------------------------------------------------------------------RFNVSRGFSYSYYTVPPAEgpTLIFLHGFPSTSEDWLRQVAYFKPKGYGLIIPDMLGFGRSDKP----LNVESYAGSLLAKDVVEIIEAESATDVAVIGHDWGSLVTSSLANRYQDRFTGFAFMTVGYNNPNGTLFDAELTRDATKEQL-GYEINGYWWFFSSPESPAIIRDNIDAFYDVVWAKNPYPAWRYHIAPT-GALQSFLESGKRLPRSKSQSKEEYRRSQERFLKSG-YEAPTMAYRYLMSKLAGEDVKSAPEdvQKPVLFLGAGQDAIAIPEFHQPTPDVMPNATSKILDSGHWIMFDAEKEVNKELEAWIKGL-------------------------------------------------------    
 352 UniRef50_Q70K85        Putative epoxide hydrolase ...   153     1e-35  1  13.9%  98:413     2:273     -------------------------------------------------------------------------------------------------HTFTSDSLTLQVTEHGSEDGEPIVLLHGFPQDSTSWTAVAEILAETGFRVLAPNLRGY----SPGARPSGRAAYGSNQLVSDVVALLDAKGLDTAHVVGHDWGGALLWTLRKTHPHRIASATVISTP--------------------------------------HPAALAWSFTHSTQALRSWYIAAIALPAVPELFLRH-----RLAQFIGSTGLPADRAQYYQNRMQEPGAATATLNWYRQMMIEQIRPPKRpaGKTGPPPTAYVWGSSDAFYTRAVAERTSHVVRDLTfqEIEGGHWLPETHPSELAEAIRR------------------------------------------------------------    
 353 UniRef50_C1YMQ0        Predicted hydrolase or acyl...   153     1e-35  1   9.5% 103:417     2:258     ------------------------------------------------------------------------------------------------------RGINVTYTDRGA--GYPTLLIHGHPFDRTMWEPQVRALAGRGYRVIAPDLRGYGSSTVVPGTT------TLDTFARDLDALLDHLGLDVVSVVGLSMGGQIALELYRLFPDRVDSLTLAATNPS----------------------AETERGRAARVALAERLRAEGMRGYGDEVLVGMMTADNVREL-------------------------PAVADHVRAMMYAAPPEGAAA----ALLGRARRQDYAPLLRRVSAPTLLVGGQHDVFTPPDLTESMHVLVPDSVVEIigAGHLPNLERPERFNEVLRRFLDR--------------------------------------------------------    
 354 UniRef50_A4FBT1        Alpha/beta hydrolase fold n...   153     1e-35  1   8.4%  97:417     5:284     ------------------------------------------------------------------------------------------------EEVASVNGLRMHYRRAG--EGPGLVLLHGWPQTGHCWRRVVPELA-RTHTVIAPDLRGYGRTDKPRG------GYDKRTMAADVSALVEALGFTSVQVVGHDRGARVAHRWGLDRPGQVERLAVLDIVPTREMWRRLDADLAPGYWHWLFHLQ--------------PDLPERLAGADVAGYLGHFFEHWTY---------------------NRHGLEPAAVEEYVRAFSVPGALRAGFDDYRASFPLDAEHDDADAarRLPMPVLALWGSTGLPARLPMLDIWRDYADDVRGtiAECGHFLAEERPEEVLRHLREFLTS--------------------------------------------------------    
 355 UniRef50_D1YX01        Putative uncharacterized pr...   153     1e-35  1  11.7%  98:421     2:269     -------------------------------------------------------------------------------------------------PIIKISDIDMYYEEHG--EGEPLILISGYSADHTSWAHLIPVFSKK-YRTIVFDNRGVGQTVIPDTP------FTIDDMADDTAALLDALNIEKAHVIGVSMGGRIAQAIALRHPKKLKSLVLCSTTARVPPRTRFALGMMADALAKGNIGHEFHDMMMLSW-----------------------------------------------TFSDRAFSSPEFMNRIRAGASSGRVRPLPANMVRQLQAGYQF-DTSLRLGEIKTPTMVIHGNEDILFPISYGRELAAGIPGAKFVElgAAHSAYLEAADRFVPAAMSFLAWVDAS----------------------------------------------------    
 356 UniRef50_C5DFH7        KLTH0D15180p n=1 Tax=Lachan...   153     1e-35  1  14.2%  97:414     6:275     ------------------------------------------------------------------------------------------------HEIAFVNGIRLHYVRAG--KGEPVLLVQGWPQTWYEWHCVIPLLVAGGHEIIAVDMRGSGDSDKP------DSGYDSNSVAEDPHALVKHLGFEKIRVVSHGNGARVSYAYAANYRSEVKSLVFLESKILGIESDDDAEKE------YWHFGFHQAPDLAQLLVTGRERKYLSYFFKTYSYDPNST--------------------------------TESDLDEFVRCYSAPGGLRGGFEFYKAFPESACQSRELAV-RKLDIPVLAWGGSH---CMGEISYRSMTLVAnaGGIIPEYGHWIPDEKPDFIAKEILAF-----------------------------------------------------------    
 357 UniRef50_C6WM89        Alpha/beta hydrolase fold p...   153     1e-35  1  13.5%  96:421     7:291     -----------------------------------------------------------------------------------------------RHRYATVDGRRLFYREAGDPVKPAVVLLHGFPTSSSMYRDLIPALAAD-HHVIAPDHLGFGLSDAPP---VTEFDYTFDALADLTAALLARLGLTRYAIVVQDYGAPVGWRLLLRDPAAITAVITQNGNAYEEGLVPEFW-----------------GPVAEYWRERTPET---------------------EAGVRAALTEEAIRWQYLTGAPDPELVDPATWRRDHALVSRPGNDLAQLSLFADYATNVAlpRLHEVFRATRVPLLAVWGEHDEIFGPAGARAFATDLPDAEIHLLDGGHFLLETAAAEVAPLVLRFLAERVPA----------------------------------------------------    
 358 UniRef50_A6SMZ9        Putative uncharacterized pr...   153     2e-35  1  14.1% 110:414    14:282     -------------------------------------------------------------------------------------------------------------VESGPSDAPTLLLLHGFPSSSHQFRNLIPLLSKK-YHILAPDIPGFGFTTVPE---SLNYKYTFDALTTSIESFLRELKVQKYSIYLFNYGGPIGLRLAIRQPDAIQSIISQNGNAYTEG------------------LGGFWAPLEQMWATKTPEELASSRESLKGVISLDFTKWQYVTGTPE---------------ANLHKIAPESYTLDYALISRPGNTDIQLDLFYDYRTNIDlpEFQKFFRDTNVPILAVWGKNDEIFVPAGAEGFKKDSDNVVVEYLDAGHFALETNVEEISDAILRF-----------------------------------------------------------    
 359 UniRef50_C6CSA0        Alpha/beta hydrolase fold p...   153     2e-35  1  11.4%  92:415    12:274     -------------------------------------------------------------------------------------------RTSGVSFFTTGEGIRIAFRIDGAAGKPILMLANSIATSMNMWDGQIARFTE-HFRVLRYDYRGHGDSDTPAGP------YSMDRMGRDVIELLDSLQIGRVHFLGLSLGGAVGQWLGIHAPERISRLVLSNTSSYLGPSE------------------QWNGLIDSVLQAENLNGFADMFINNWFPSPMIESESEIVAAFRQMVLAT-----------------------------RAEGIGGSWAAIRDM-------DLRRTAALISSPTLVIAGQHDTVTLPSHGELIAETVPHAKLLILPAVHLPNVEFPDAFEKAVIDFL----------------------------------------------------------    
 360 UniRef50_A5VE07        Alpha/beta hydrolase fold n...   152     2e-35  1  12.1%  95:417     5:276     ----------------------------------------------------------------------------------------------PQSHFADVDGVKYHYHDIGT--GEPLILIHGGgsGWSNYSRNVEALS-RQFRLIIPDLVGYGQSDKPVLEGPRFGIY-----AKGMLGLMSRIGIEKAHVVGNSLGGGTAIKMALEAPDRIGRLILMGPAG---------------------------------------------LLAPSTPMPTLGVRQIMEFYGGEGPTREKLRAFLQTMVYDSSFLTEELLDGRIAAATQPDLLANPPLGRGGPPPIEAL--WKENLAGITHDTLILWGREDRVNPMDMHLTLLAQLPNAQFTVfrCGHWVQWEKAKAFNALVASFVRG--------------------------------------------------------    
 361 UniRef50_B5HDV4        Hydrolase n=1 Tax=Streptomy...   152     2e-35  1   9.2% 101:415     2:260     ----------------------------------------------------------------------------------------------------TVNGAAIHYSDQGPADGVPVVLVHGHPFNRSLWAPQTEALTAAGYRVITPDLRGYGESEVVPGKTL------LSDFADDIAGLLDRLGLERVVVGGVSMGGQIAMEFQRSYAPRVRALVLSDTSPVAETEEGKAFRNSLA----------------------------------------------DRLLAEGMDGYADEVIDKMLAAYNVTAMPD-----VAAQVLTMMRTTAPEGAAAALRGRAERPDYRDTLAAVKSPVLIVVGTDDAYTPVSDAEAMRDLIPHATlvIEDAGHLPGSEQPALVNKGLLYFL----------------------------------------------------------    
 362 UniRef50_O31243        Epoxide hydrolase n=3 Tax=R...   152     2e-35  1  11.7%  90:405     4:283     -----------------------------------------------------------------------------------------RRPEDFKHYEVQLPDVKIHYVREGA--GPTLLLLHGWPGFWWEWSKVIGPLAE-HYDVIVPDLRGFGDSEKPD--LNDLSKYSLDKAADDQAALLDALGIEKAYVVGHDFAAIVLHKFIRKYSDRVIKAAIFDPI----QPDFGPVYFGLGHVHESWYSQFHQLDMAVEVVGSSREVCKK-----------YFKHFFDHWSYRDELLTEEELE------------------VHVDNCMKPDNIHGGFNYYRANIR-PDAALWTDLDHTmdLPVTMIWGLGDTCVPYalIEFVPKYYSNYTMeiEDCGHFLMVEKPE--------------------------------------------------------------------    
 363 UniRef50_B8GW03        Alpha/beta hydrolase fold p...   152     2e-35  1  12.5%  59:419     8:317     ----------------------------------------------------------LAIALGVLIAVAVGAWFLLQRPDIPYATLEAKYGYADSRYMALPGGVRAHYRDLGPRDAPAIVLVHGYSASTHAWDAWAVRLS-KTYRVIVLDLPGHGLT-----RTSGPYVAGRQGFVAVVDALTARLGLTRFTLAGNSMGGAVAWTYAVDHPDKVQRLVLVDAAGWPSEKSGGALIFTILRHPI------------------------------------------GRAVLKDVDTRPLVAQGLRSGYWDQSLVTPALIDRYVELSRGPGHRDILL----SLQSGPRRDATVAELSTIHAPTLIMFGQEDRIIPAADGEKFHKAIPGATLIlpGVGHVPMEQIPDRSAADLEAWLAAHP------------------------------------------------------    
 364 UniRef50_Q0U622        Putative uncharacterized pr...   152     2e-35  1  10.4% 101:418    11:328     ----------------------------------------------------------------------------------------------------TSRGYTYTYYTagDKSLPTLFFSHGWPDHATMWKNVATSLRPTGHPVIIPDLLGYDGTDKPTDPA----AYKWNLMTQDLIEIIDTEEVSKVISIGHDWGSVCASRLYNHHPDRVVGLVLLNVAYQPPTRQPFDLDRANAMM-QQIFGNPLFSYWYLLIAPDGPEIllDRLHNAMHGTAETMKKFFTTPNALREYLVNGGDDFEIRPYARDPKFKQEFIDRFTR-----DGFEGAVCWYKANTENHQYeqLPQTADKVNVPVLYMGGKDDAVCRPEAmaIQAGLLPQLEqnMIDAAHWTPYEASGEVAGNIKSWLGKH-------------------------------------------------------    
 365 UniRef50_B1XKS0        Hydrolase, alpha/beta fold ...   152     2e-35  1  11.3% 100:418    48:326     ---------------------------------------------------------------------------------------------------WTWRDQQIRYTVQG--EGQPLLLIHGFGASIGHWKHNIPALAAHGYQVFALDLLGFGASAKPA------WDYSLDLWQDLLRDFWQAKIQQPTVFVGNSIGGLLSLAMLANYPDLCAGGVLINCAGGLNHRPDE-------------------LALPLRVVMGTFAKLVSSRLTGPFIFNQVRQKSRIKNTL-------------YQVYGDRQAVTDELVEMLYEPSCDPGAQQVFASVITA----PPGDSPTELLPKRQHSLLVLWGDRDPWTPIKGsqDLAAQNAGVEFhiPGAGHCPHDENPSLVNSLILDWLQRL-------------------------------------------------------    
 366 UniRef50_B0L3I5        Lipase/esterase n=1 Tax=unc...   152     2e-35  1  12.8% 100:420     3:264     ---------------------------------------------------------------------------------------------------VTVngVKIHWNEEGA--GEPLLLIMGLGYPSAAWYRTRPALS-RKYRTIALDNRGVGQSDMPPGP------YPIPVMASDAAAVLDAAGVESAHVFGISMGGMISQEFALQYPNRVRSLILGCTAAGGP---------------------------------------------NAVRADQDATNMVMARGAMTQEEAAQAAIPFIYDPSTPRNVIDEDLAIRRPWFPRQEAYNAQLQGIIAWESDS-------RLDKITAPTLVIHGDSDRLVPPGNGKRIAEKIPGARLVMLraSHIFTTDQPAAAQKAILEFLQEHST-----------------------------------------------------    
 367 UniRef50_B7KDK4        Beta-ketoacyl synthase n=3 ...   152     3e-35  1  12.1%  73:411 2462:2760     ------------------------------------------------------------------------PHETVLPSPPRRGVGGEVISIPMQENYLTIRGLKLCLCSWGPEDGELILCIHGILEQGAAWEEVATRLAQKGYRVIAPDLRGHGKSDH----VGNGGSYNLIDFLGDLDAIATHLTDKPFTLVGHSLGSIIAAMFTSIRPEKVKHLVLVETVLPTEVHEGDTVEQLA-------------------------------THLNYLSSPPKHPVFPDVETAAKRLQTATPAMSEQLAMKLAKRITQAGEGGIQWRWDSLLRTRAGIEF-----NGINRSRYLSLLKQIQAKITLIYGDQSDFNRPEDLQLQQQTMSQANRIVVNGGHNLHLEAFEELANII--------------------------------------------------------------    
 368 UniRef50_UPI00016C3D4D alpha/beta hydrolase fold p...   152     3e-35  1   9.1% 100:419     5:265     ---------------------------------------------------------------------------------------------------TLPSGPTIGYDDTGGGSAPPLVLLHAFPLDRSMWQPQLAALA-AHARVIAPDFPGFGESS--------PGAFTVDSAADLVAEFLGALGIGKAVVGGLSMGGYVALAFARRHADKLAGLILADT---------------------------------------------------RAGVDDSSARENRTKSIELTREKGSAALFEGMAAKVLSDSTRDNKPEVVERLKGVAAKQPAESVIAALVALRDRPDANPGLKGVTVPTLVLVGEHDGVTPPLSSANLAAQIRGSTlhIPGAGHLSNVENPDAFNAAVRNFLAATA------------------------------------------------------    
 369 UniRef50_Q6D2X2        Putative hydrolase n=1 Tax=...   152     3e-35  1  10.8%  23:402   103:429     ----------------------PQNFRPLDFSISDIIAHNDSVLVLGRFRHTVLStaVGSDMIIHFKVSGSKITRYQMFEDSALLSRAFDASDEWQLQQVRINGTLYRYRDIG--EGPTLIFAHGLFANHEIFSAQFQILS-KSYRCIVLDMPGHGLSE------YDPAGWKLDDLSRDLALMIQELSLGKVTFIGQSQGGMVAIRLAAHYPQLVSGLVLIGTS----------------------------ARAEFPERLQNWHRQRDILLTGSEHAREDLFKKIQSHINNEAWLQNNQGEAARERRIMLSHNRTGLALALDAAVFERG-------------------DITELLADISAPTLIICGEQDTATPVELSQEIATLIADASILILatGHHPPTE-----------------------------------------------------------------------    
 370 UniRef50_Q0SD10        Probable hydrolase n=1 Tax=...   152     3e-35  1  14.9% 107:415    16:280     ----------------------------------------------------------------------------------------------------------LRFVDVG--EGPPVVLVHGLLGSHESWAPQISRLAKK-HRVVAPDLFGHGQSDKPSG------DYSLSAHAATLRDLMDHLGISSAAFVGHSLGGGIVMQLSYLFPERVDRLCLVSSGG-------------LGREVSVFLKAATLPGSELVLPLLASDRLRRTT--------ENILKHLGRLGLPVQPSRSATETWRSLETVSDKSSRAAFLASTRAVVGLRGQTVSAKQHFSKFE---------------SLPSLLVWGGRDRMIPASHAENLRRVVPHSRVEipRAGHFPQLDEPELFFRVLDEFL----------------------------------------------------------    
 371 UniRef50_C1YQS0        Predicted hydrolase or acyl...   152     3e-35  1   9.8%  95:421     9:298     ----------------------------------------------------------------------------------------------FARRTVDAGGVRVNVRLGG--DGPPLLLLHGYPQTHLIWHRVAPLLADR-FTLVMTDLRGYGDSDKPASD-ERHAPYAKRAMARDQYLVMRGLGFDSFAVAGHDRGGRVGHRLALDHPEAVTALCALDIVPTRYAFEHADAEFALGYH--HWFFLAAGNGIPEHLIGQDPDFWVR--------------------------------TRMNARHHGGTPFAPAAVDEYVRCFRDPAAIHASCEDYRAAASIDLEHDRADAGRRLECPVLLLWGRHSFvrHYDVPDAWSGYARDVRGHALDCDHYVPEEAPEATARHLRAFLGGGSAP----------------------------------------------------    
 372 UniRef50_A4SXI5        Alpha/beta hydrolase fold n...   152     3e-35  1  10.3%  98:416    14:298     -------------------------------------------------------------------------------------------------TVPSADgiEIACQIGGS--GPALLLLHGFPQTKAIWHKVGPALA-KNYTVVIPDLRGYGASSKPNG-KDDHSTYSKRSMAADQHALMKELGHEQFFLLGHDRGGRVSHRLAMDFPQSVKRLMVLDISPTLAMYENTTMQFARG--YWHWFFLIQPAPIPEAMIAANPEFWLK---------------------------------KHMGRHAGTGIFSADRWTEYLSAVSDPQCMHAMCEDYRAAASIDLVHDREDRakKLVMPLRVLWGEhdDWSNASKSEVTGKALA--------CGHYIPEELPDTLIEEARTFFS---------------------------------------------------------    
 373 UniRef50_B4UX89        Hydrolase n=7 Tax=Streptomy...   152     3e-35  1  13.9%  72:416     7:309     -----------------------------------------------------------------------APNRSCDQNPVPGLPLYDLAG--FTHRWVDAEGIRLHAVEGGRPAGPTVVLLAGFPQTWWAWRKVMPGLSER-FRVIAIDLPGQGHSDRPQG------GYDTHTVASRVQAALTALNVPKYWLVAHDIGAWAAFSLALKYEERLNGVALLDAGIPGITLPDSIPTDPDRAWKTWHFAFHLVPELPETLLTGRERDYVEWFLK--------------------------------VKALSPNTFDGAEIDHYAAAIAAEGGLSASLAYYRDAAESARRNHDALERGHLTIPVLGISSSHGSI--PDMAASVSPWAENAtvVIPQAGHFIPDEQPDATLDALTAFID---------------------------------------------------------    
 374 UniRef50_C7YVQ8        Putative uncharacterized pr...   152     3e-35  1  11.0%  90:417     2:306     -----------------------------------------------------------------------------------------ALELPRRESFAARSGNSYSYIHIQPTSrtTLLLLHGFPSTLSDWIHQIRYFSSEGYGILAPDLLGYGSSSKPSDV----HQYRLKAMGDELIELLDHLNLQKVVGIGHDFGATLLSRLAVYHPDRWTCLVFLAVG--PPRLGTPFDVNMINKMTKEFLGFEMLGYIPWIADPATPSSLEK--HAEAAMSLIFCRDRQAWDEWFHPLGKMKQFVTEDRRLPIGPWYTDDLQKEHLKAFGVSDGYKGASRWYRMWMDNLFAPDEKGFEnqISQP-----------------QMLASWAPKLQTVKLDAGHWIHLERPEETNTAIQQFLEA--------------------------------------------------------    
 375 UniRef50_UPI000187D95A hypothetical protein MPER_1...   152     3e-35  1  12.8% 100:419    10:327     ---------------------------------------------------------------------------------------------------VTKRGLHYEYFYSPapHKPTLFFLHGFPSQSHEWHRQVEHFKPLGYGILAPDMLGAGRTSRPLD----SKDWKLNLMAADLVDILEAAKLEQVIGIAHDWGCAALSRLSILFPERFLGFVWLGLSFLEPITEKFELDAAMAITKEYLGYEGYSYWKFFEQQDSAKTIQD---HVDSFIQLLYPKDPSTWLTYMALPGKTAEWIENDMKPGFPDYLSMEDIDDIRRNVLDNGGIKSSLNWYISQIESNDLEDNLNIPKelQSPSLFIAASRDYICTPKRGKdmARYASEVKIVDVDTGHWVHLEASDKVNAEIEQWLNELE------------------------------------------------------    
 376 UniRef50_C0AED1        Putative hydrolase n=1 Tax=...   152     3e-35  1   9.9% 114:417    35:308     -----------------------------------------------------------------------------------------------------------------RGSGEPLLMLHGYPQTHLMWHKVAPALAE-NYTVVLTDLRGYGDSSCPPDG-ENHLGYSKRAMARDQVEVMRQLGFERFYLAGHDRGARVCHQMLLDYPEKVRRAVLLDI--IPTSEMYARTDKEFASIYFHWFFLILPNDLPEKLLAGNPEAWIRRTAGS------------------------------LGSRGGGDAFPEDVMAHYVEKFSNPAVIHATCEDYRAGATVDLEHIAAAGArkIPVPLLVLWAA--PYLrpDVLGIWRQHAADVtyPVPGCGHFIAEEAPEDTIKAIRHFFDD--------------------------------------------------------    
 377 UniRef50_D1A847        Alpha/beta hydrolase fold p...   151     4e-35  1  12.7%  92:423    12:308     -------------------------------------------------------------------------------------------EGPWKHRAVSAGGTRFHVAEAG--EGPLVLLLHGFPEFWWAWRHQLVSLSAAGFRAAAVDLRGYGGSDKPP------RGYDLVTLAGDAAGLIRALGEACATVVGHDWGGLLAWTMAVHRPKVVQRLVVASAPHPLRLRQAVRTQPRRQARAARHSLGFQLPMWPERRLLRDDAALVG---------------------------------RMLHEWSGPGWPDESTERRIRQAIQIPGVAHSALEYHRWLIRSLPRPDGIRYARsiQAPTLQIHGALDPLTPPSGAQGSGRYvpYRWRLIAEAGHFPHEERPEAFDAVLLEWLKDSGAPGR--------------------------------------------------    
 378 UniRef50_B8KIC4        Haloalkane dehalogenase 2 n...   151     4e-35  1  10.0%  85:425     4:306     ------------------------------------------------------------------------------------RYSPPPALYPFESRWLDVDGHQLHYIDEGPRDAPVLLMVHGNPTWSFYYRKLVLRLRDR-YRCIALDHIGCGLSDKPDDA---HYDYHLAQRIADLDALVQATIGdpLSLVVHDWGGMIGFAWAAQRVERIERCVILNTAAFPMPADKKFPFALWLGGRTALGAF--------------------------------------------LIRGVNAFSAMAARIAFKKPVSKDVRSGYTGPYNSWKNRIATVRFVQDIPLEESDrdTAAQLHRfdKPALLVWGLKDFVFDETFYRDWQRYLPNAELhlADCGHYVLEDGGEPLLDKIDAFLASTQAALAGS------------------------------------------------    
 379 UniRef50_B3QQW0        Alpha/beta hydrolase fold n...   151     4e-35  1   8.0% 120:418    15:256     -----------------------------------------------------------------------------------------------------------------------VLLLHAFPVSADMWEHQLAPLAESGYRVIAPYVYGF-------DTSPSRPGWSMDDYAHDLARLIRALGWSSATVVGLSMGGYQAMAFYRLYPELTDSLVFCDTRANADAPEALAARQEFRTV-------VMEKGAEEAAARMVPNFFAPETY--------------------------------------------ESNEPLVEKTRQGIIRQTPEAISEAMRAIAEREDSTELLNEITCPTLIVCGMDDKVTPPETAAEMHALIPGSKLeiPDAGHLSNLEQPAMFNGILLEHLRSL-------------------------------------------------------    
 380 UniRef50_A5VD37        Alpha/beta hydrolase fold n...   151     4e-35  1  13.0%  71:415    13:300     ----------------------------------------------------------------------AATMPAFPAMASVEAPAAASRSPAAADRFVTVEGIRFRVREEGPRSAPPIVLIHGFTFSLESWDGWAADLA-RDHRVIRFDLAGHGLST-----PDPRGRYGTAARVRQLGKLLDRLGVAHATIVGNSFGGLVAWNFAVAHPRRVDRLILVDSAAYSINGVTEKPVPVPDMMRGYLLDPKPAA----------------------------------------------VAFSAGTIFAHPERLTPDRLALMRTMIARNG------PALVAHLEQFTLPDPQGPLGRITAPTLILWGRADKVIPVAQADQLAAAIKGSKLIIydVGHAPQEEASAASIADVRAFL----------------------------------------------------------    
 381 UniRef50_A4S9C0        Predicted protein n=2 Tax=O...   151     5e-35  1  13.4%  84:419    13:313     -----------------------------------------------------------------------------------AQRYGVDERYWRWRAPRTNETHAIRYTEagDGDGPAVVLVHGFGGNADHWRRNVNALAATGKRVYAIDLLGYGYSDKppMLREQNEIYCFETWGKQIEDFLDEVVGTPAYVACNSVGGVAGLQAAVDAPTKVRGVVLMNISLRG--------------------LHVSKQPAIIRPFVKALQRTLRETSVGKSFFGSVAKARTVKNILCEA-----------------YGDSAQVTDELVEAILSPGLREGAAEVFLDFISYSGGPLPEELLPRCDVPVRMFWGDKDPWENIDQGRKLYASYADKFIPLPGVGHCPQDEAPELVNRLLVECVDEWE------------------------------------------------------    
 382 UniRef50_A7HTR1        Alpha/beta hydrolase fold n...   151     5e-35  1  12.2%  96:414     2:290     -----------------------------------------------------------------------------------------------TQKLVKANGISINCEDRGPADGTPILFVNGYTSTMMSwdELMDGLRDKGFRVIRYDNRDVGKSEKFSGLPkpDMPYTLADMAADGIGVLDALGIKRAHVMGISMGGMIVQRMAIHHPERLLSVTSIMSSTGNPD------------------------------------------LPKASDEAMKALQAMPPSEEREDVIRHRMKGRRVYQSPAYPKSEEDLYTFVAREYDHMYYPEGGIRQYAAIMGDGSR---VAELGKVRVPFLVVHGHADPLVPHAGGEDTAKSVPGAKLVSIDggHDLPVELCPQYVDLIATH-----------------------------------------------------------    
 383 UniRef50_D2PZP5        Alpha/beta hydrolase fold p...   151     5e-35  1  11.9% 104:422    18:307     -------------------------------------------------------------------------------------------------------GHRLAVRRQGAG-GVPLLLIHGFPCTSRIWSHNLVPLAEAGFDVVAPDLRGYGDSDF-----APDDFYDFNAFTTDLTGLFDTLGWDRAVVAGHDLGAMIAIDLANRHPDRVDRLVILDDSMPDLKD-------------------------AFAAAGIPPLDLKPAVYDYQRRQGHHADALVAELDTPERRRRYVAEFYGHRLWCPPDAFGEEDLAFLTEPYGDAARLRASFVDYEIVMGTRPLSAPEMTDRPVRQSTLVLIGEDQVTVGDHIEERCAIAFPEAvfWVKGAGHFMPWERPDTVNRAIRSFCGDLLVAR---------------------------------------------------    
 384 UniRef50_Q7NMH0        Glr0796 protein n=1 Tax=Glo...   151     5e-35  1  11.4%  99:422     3:280     --------------------------------------------------------------------------------------------------YLDIDGTKHFYTLSGKPKPepLVLVHGWCGSSRYWEPVAETWAERTV-CLRYDLRGFGQS---PLTPDSNQNFSLEAYSRELLALIDRLGFERIDLNAHSMGASIGLAFANLFPERVRKLVLTCTGVFQYEA----------------------------------------LLFETFYLASNVVIAVRPAWMVNFAVARKAFQRRFVYKLLEDRWARLFIEDYVVA-----HQAAARGTLYSSIGKHATYEMPDAFRKLAVPTLLISGEYDQIIPAAGAKRAVKVNPGLTliIPQTGHFPMLEAPGAYLESMNSFLESPGSGA---------------------------------------------------    
 385 UniRef50_Q13BX0        Alpha/beta hydrolase fold n...   151     6e-35  1  10.8%  82:415     6:298     ---------------------------------------------------------------------------------DRSAPHARAYMQNPESRFYRSHGLRLHYADWGNDTAPPLLLIHGGADHGRSWDHLARSLRPD-FHVVAPDLRGHGDSDWTLGGS-----YSLPEYIYDLTRLPAFEGRGPITVIGHSMGGMVGLIYAGTFPEKVARLVVLDGVTVLPNAPKPPAHERTRKWIGQLDALEQ---REPRRYRDIEEAAAQMQAHNKRLTPELALHLATHGVKQNDDGSYSWKFDPYQRVMAPHRLWPD--------------------------------DHVALWARIACPTLLLFAGES-FLGDAASAGIADHFQQARVevADAGHWLQHDRPDEVLRLIREFL----------------------------------------------------------    
 386 UniRef50_B8HT37        Alpha/beta hydrolase fold p...   151     6e-35  1  12.1%  64:417    11:330     ---------------------------------------------------------------GASASASVRPQSSGSTTPAEVIPVPSWRDRLGYERNWFWRGWQTRYTYLRPAdaPPLILLHGFGASIGHWRHNLSVLA-QSHPVYALDMLGFGASQKVIAP------YSIQFWVEQVYHFWKAFIGRPVVLIGNSIGSLVCLAAAAQYPEMVAGIAMLSLPDTSIREEMLPATVRPIVAAIEGLFTSPLLLKALFYYVRRPKIVRPW---------------------------------AAIAYANSVAVTDELVEILVGPAQEKGAAGAFAAIIKAMTSRGFGPKVKAVLPGLDIPILLIWGQQDRMIPPLLGRQFANYNPRVKLVEleAGHCPHDEVPDRVNAEILTWLAA--------------------------------------------------------    
 387 UniRef50_A5EQU9        Putative triacylglycerol li...   151     6e-35  1  12.8%  95:414     3:266     ----------------------------------------------------------------------------------------------FADKRLSIRGINIAYWEAGS--GEPLVLLHGMGVDRGTLLDVAGKLTG-HYRVILPDLPGFGDSDKPE-----RADYGISAQVDNLREIIKALGLHRVHLGGHSMGGWISAGFAASSPEMVESLWLISAAGTSELDHSLPMEAFRRGEYV--------------LCCRSPSELRGVMHLAMFKLPRLPYCVWQALGRRAAANYALHT-----------------------------------RIFARIMQDISGYNLEERLPKITAPTLIVFGDSDRLVPPSVLRTFKRLIPNSRSILlnVGHVPQMEAVDRCASDYLAF-----------------------------------------------------------    
 388 UniRef50_D1VM84        Alpha/beta hydrolase fold p...   151     7e-35  1  11.0%  89:414     6:287     ----------------------------------------------------------------------------------------VASAPPVLHRTVDVGGIRVFYREAGDPGRPAIVLLHGFPSSSSMYRNLIPLLAP-HFHVLAPDYPGSGNSEFPAEDKWVP---TFANLADVMEQWVEKVGLDRFVFYMQDFGGPVGFRLAVRHPDWVAGLVVQNAN-----------------------LFLEGFPPQQAAAPGDEDQAGQESMADKTVTVEFAKFLYQ------------------AGARDPELLSPDAWNLDAYALTHPDSQRIQRALITDYPTNIAlpAWQAFLRERQPRTLIAWGRNDQFFVPAGAEAIHQAVTDSELRFFDTSHFALEEDLVPIAQAIVDF-----------------------------------------------------------    
 389 UniRef50_B0U8T6        3-oxoadipate enol-lactonase...   150     9e-35  1   9.7% 100:470     4:317     ---------------------------------------------------------------------------------------------------IEANGTRLHYELSGPSGAPVVAFSNSLGTAMAMWDPLVPHLRGR-YRVLRYDTRGHGASPARDAPIA------VEDLADDLLGLLDALGIGRAHVVGLSLGGMTGQALAMRAPERVLSLTLMATAAEMPSEQSWN------------------------------ERAATVRAEGTAAIVDATMERWFSPDFPRTSPGAVEPVRRQFVETDR-------------------------AGYAVCCNAIGRMDLRPGLGRITAPTLVIAGRDDPATPPAKSEEICAGIRHAELVVLPGahLLAVERPDATASHLLAFLDRHRGAAGAATGAVAFAerRGVLGEVHVDRSLAAAGSFAGPWQDFITRIAWGEIW---    
 390 UniRef50_A8LF43        Alpha/beta hydrolase fold n...   150     9e-35  1  13.0%  92:444    17:337     -------------------------------------------------------------------------------------------DGPWRHRDVSTNGTRLHVAEAG--EGPLVLLLHGFPQFWWTWRSQLRDLPRAGYRVVAADLRGYGASDKPP------RGYDAFTLADDVAGLVRALGERDAVIVGHDWGGLLGWTTAVRRPRVVRALAVIGMPH--------------------------PLRIRRQIAADPRGQGLASCHLAAYQLPWRPERRLVADGAAHVG-------ELLRSWGGPGYPAPETEARYRLAMRIPGVAHSSLEYHrrSLFRPDGARFAELLRtvRAPTLQLHGRLDRCFLPSTAAGSDRHVAAssWRLYDggHFPHEEDPDTVTRDLITWLAETAPEQAPtaAEPAPDAEPAAEPDAEPD-----------------------------    
 391 UniRef50_A6RY56        Putative uncharacterized pr...   150     1e-34  1  11.0% 105:414    29:305     --------------------------------------------------------------------------------------------------------VTIHYILCHPPSNVtiLLIHGYPETSYQFRHVITPLADTGYTVVAPDYRGAGESSHPRK------GYEKVQMATDLHEVIqdLDidKIHVVGHDIGGMVAHAYAAQFPKDTASVAWGECPLPGTDAYNNAVKEGITGGLWHFVFHWQTDLPEALTAG------RENIYIKSFYDRLCYNA---------------------------SAFSPNDVEHYASKFAQPGGMRAGFDLYRAFHQDAEDNLRMLKGGKCKVPSMSLMGEKSFLATIANqeQMYETTSSAS--IPGSGHWCAEENPAGFVEAVLKW-----------------------------------------------------------    
 392 UniRef50_A7HHE1        Alpha/beta hydrolase fold n...   150     1e-34  1  13.3%  98:420     2:264     -------------------------------------------------------------------------------------------------PIAHVNGTDLHYRDAGTAHKDVLLLLHAFPLHSGMWLRQIAALEGR-WRIVAPDYRGLGQS------APRGEASTMQVLAEDVRALLQHLRIERAAVAGLSMGGYLSLELYRQIPGFFRGLALCDT----------------------------------------------RAGADTDEGKAGREKFAQTAIERGLEWVADEMIPKLLRPEPDPAVAKEVRD-----LIRRGTPAGVAAAQRGMAQR---PDSTETLAKITCPTLVLVGAEDTLTPPAESEKMAKAIKGAKLvvKKAGHLANLEATAAFNAALQEFLDGLPA-----------------------------------------------------    
 393 UniRef50_Q0VLQ1        Hydrolase, alpha/beta fold ...   150     1e-34  1  11.4%  99:422     5:296     --------------------------------------------------------------------------------------------------TVASNGIELFYESRGPENGEPMVFVMGLSAQMVFwdTLLDALAAKGYRVIRFDNRDVGKSTQIRKplPVESPYTLHDMVADTVGLLDALNIERAHFVGASMGGMISQLMAGTHPERVLSLTSIMSSNNSSLLPPPK-----------------------------PSALRVLIAPRVKVETEEQFVTFGLEMMSKLAGTLPQGK-------------EELAAMYRAAWARGINPRGIRNQFLAITATG---SLSKTLKQIQCPTTVIHGGADPLIRPAGGKASARAIRGAKLVIIPggH----DFPPSVIDRIGELIAETAGRA---------------------------------------------------    
 394 UniRef50_Q6C598        YALI0E19899p n=1 Tax=Yarrow...   150     1e-34  1   9.3%  86:419     2:347     -------------------------------------------------------------------------------------AHLTKEYYEPFHHDVILGGKRWHYLdeGKDNGRVLVLVHGFPDFWYGWRHQIPVFRKRGHRIILPTLMGFPGSEVPEPPAMEEFeyGFKFFADCMAELLKKLNIKSATFLGHDWGAHYVPKVWAYHPEIVDAISSACWYYQVPEPEWVPLTDFSDKWPTTKYQLQFGG------------------------DAVNNIGPGMIPFFLRRSYTVGANFDGEPDPEAPMHMTEEEFAVYEEHFseKRSLAGPFTYYRSRKLNWEQDKENFLDktVNVPYLYIGSTNDIALIPEMSMHLDEYVEKGKLTRepTSHWALFEAPDQINKIYVDWLDKLD------------------------------------------------------    
 395 UniRef50_A3TT13        Probable hydrolase n=1 Tax=...   150     1e-34  1  10.5%  99:412     3:290     --------------------------------------------------------------------------------------------------TAQVNGIEIWYEELGPEDGKPILMISGVGTQSTRwaDFVDLLVARGYRVIKMDNRDIGLSQKfaAGKTPDIPYTLSDMAADGAALLDHLGIDKAHVCGFSMGGMIVQLMAIEHPDKVLSMSSVMSNSGNPD------------------LPKPTDAAMAALTRPRPDALKD----------------------RDAFINSRIETDHVIGSPAYPIPDEDLRAQAEADLDRSYHPTGFARQYAAI---LATRDRRAELRNLTMPVAVIHGTDDPLVPVTGGRDTAENCPTADLIEIPggHNFPHEVYEAVLDGIE-------------------------------------------------------------    
 396 UniRef50_Q0C3I4        Haloalkane dehalogenase n=3...   150     1e-34  1  12.2%  77:419     3:332     ----------------------------------------------------------------------------VMRTPDARFENLPDYPFAPHYHNVTAPdlRMHYLDEGPRDGEILLCLHGQPSWSYLYRKMIPLLTAAGYRVLAPDLIGFGKSDKPGAI----EDYSYSGHAAWLEEWMLALDLTGLTLVCQDWGGLLGLRLAGMHPDRFKRLVVANTGLPDSRQLAPQMSEMLGMLYPQVPVPSAS-DVGDAFRSGAPGAFLFWV---------KYAAEAPDFAVRDVFGLLSDISDDAVLNGYEAPFPDETYLAGARRFPSLVPL-----LPNGAEERAKNDAAWAVLEKFEKPVLTAFADDDPVTKGGEA-AFQTRIPGAkvTISGGGHFLQEHRPEAFSQAIITFMRANP------------------------------------------------------    
 397 UniRef50_Q28P20        Alpha/beta hydrolase n=1 Ta...   150     1e-34  1  11.6%  82:417    14:312     ---------------------------------------------------------------------------------ATVARLGIGAEYRFAHHFAHTPMGTMHYVDEGTGD--PVLLLHGNPTWSYLYRKFIPALA-QTHRVIAPDHIGFGLSDKPEA----EGDYTLDAHIQNLEALVQQLDLTNITLVMQDWGGPIGLGMAARHPARIKALVVMKTFGFYPPIDGVDP--------------------------------------DKLKLPPPLLMMRAKGIGDFLVRRLGFFERQVMTMATATKRKGPSKRAYRDIFRTYAERAGVMAFPRMIPANTGHpeTGPYIDQFDGPAHIFWGMKDPLIPVGALTAWKKRLPQAGVTEFaaRHYLQDDVPDQLIPELVEFLNR--------------------------------------------------------    
 398 UniRef50_Q4KAX4        Epoxide hydrolase-related p...   149     2e-34  1  10.2%  96:417    26:302     -----------------------------------------------------------------------------------------------VHRYVRVDGQRIHCVIAGT--GRPVLLIPGWPQTWYAWRQVIQALAAQGFQAIAVDPLGSGQSDRPE------HGYDTGHVAAVLHQVMGQLGFERYSVVGHDVGMWIAYALASDHPQAVERLALTEAVIPGLADAPPIFVAPEQNIFLWHFMFNQLRDLPEALIAGRERAYLNYIFDH-----------------------------------WSHRREAVAAEVYIEAYAAPGGLRAGFAYYRAIPETVRQNQLRAQ-RPLTMPVLAihATNDA--PLLTLQGHANDLRGA--IVEDCGHFIMEEAPEEFLALLLPFLQE--------------------------------------------------------    
 399 UniRef50_Q1QBB9        Haloalkane dehalogenase n=1...   149     2e-34  1  12.0%  80:420     4:303     -------------------------------------------------------------------------------LRTPDSRFANLPDYNFDPHYLMVDdlRVHYLDEGPRDADPVLLLHGEPSWCYLYRKMIPILTAAGHRVIAPDLPGFGRSDKPASRT----DYTYQRHVNWMQSVLDQLDLNNITLFCQDWGGLIGLRLVAENPDRFARVAAGNTMLPTGDHDLGEGFRKWQQFSQEIPQFHVGGTI---------------------------------------------------KSGTVTKLSQAVIDAYNAPFPDESYKEGARQFPLLVPSTPDDpaAWIELSKWTKPFITLFSDSDPVT--AGGDrmQKIIPgaHTTIANGGHFLQEDQGEKVAKLLVQFIHDNPR-----------------------------------------------------    
 400 UniRef50_C3KB79        Putative hydrolase n=1 Tax=...   149     2e-34  1  13.2%  98:422     7:281     -------------------------------------------------------------------------------------------------FFTNRSGLRLHYLRWGAPTGVPLVLLHGLRAYAQTWESLVQALGE-GYCIFALDQRGRGLSDW-----AEPSSYHTQSYVEDLEDLIAHVGLRRFVLLGHSLGGANALEYARQHPGRLMGLLIEDIGPGSSTQGDGAARIRREMGQTPLQFESWDAARAF--------------------------WKASRPGLSEQGLASRLAHSMQERDGVIGWRHDQ-------------------QGIAEARLSIEPTDLWPAVRSLDCPSLFIRGGRSDFLPPAMLQAMCASNPKVqvEVADASHYVHDDQGEVFNRLVADFLSSLTHAA---------------------------------------------------    
 401 UniRef50_Q123C8        Alpha/beta hydrolase fold n...   149     2e-34  1   9.0%  95:416     7:310     ----------------------------------------------------------------------------------------------FESRSFEVNGASIQAraLGDAPRPALLLIHGFPQSHVMWHRVAQRLA-QHYFLVMPDLRGYGDSSKTPGLPDHS-NYSKRNMAHDMVAVMSALGVDRFFLCGHDRGARVAHRLALDHAARVIKLCVIDVAPTLDMYEGRGMTEPYMAFAQAYYHWFH--------------------MLQPAPLPEIMMGANHL----ETAKAYLHAKLGGWGSSGLGYIEPEALAEYERCFCNAEALHTACEDYRASARIDLEHDRESRAhkVACDMLVLSGERGVVhpMALWQEQCSGI--VAGHTLQAGHFIPEEQPQAVAQALLEFFA---------------------------------------------------------    
 402 UniRef50_B0U7A6        Haloalkane dehalogenase n=1...   149     2e-34  1  12.0% 104:419    18:301     -------------------------------------------------------------------------------------------------------GIRMRYLDEGPRDAAVVVMLHGNPSWSYYWRHLVAALRD-GYRCIVPDHIGMGLSDKPGDAPgpRYDYTLQSRVDDLDALLRHVGIDDvtLAVHDWGGMIGFGWALAHAVQVRRLVITNTAVFPMPMSKKMPWQIALGRDWRFGEWMVRGLNAFAL--------------------------------------------GAAWLGVETRLPRAVRRAYLAPYNSWANRISIIRFMQDIPCAPGDRAWPLLEAaaDRPVFIGWGMQDIVFDHHFLDEFRAALPCAQVQVfdAGHYVLEDKSSVLVPAIRAFLDAHP------------------------------------------------------    
 403 UniRef50_D1WS01        Alpha/beta hydrolase fold p...   149     2e-34  1  10.6%  64:422    27:352     ---------------------------------------------------------------AAPAEQATGPADSVSPAGRAAAGGPVRLDGPWTHRDVAANGARFHIAEMG--EGPLVLLLHGFPQFWWTWRHQLPALADAGFRAVAMDLRGVGGSDRTP------RGYDPANLALDVTGVIRSLGEPDAALVGHDLGGYLAWTAAVMRPKLVRRLAVSSMPHPRRWRSSMLSDFAQSRAGSYVWGFQRPWLPERQLLADDAALVGSL-------------------------------IQDWAGPRTPEFPDEEALDVYRRAMSIPSTAHCSIEPYRWMVRSMARPDgrRMKRPVRVPTLHLHGSLDPAVRTRSSAGSGQYvpYRWRLFDGVGHFPHEEDPIGFSTELINWLKDPEPDR---------------------------------------------------    
 404 UniRef50_B1ZTA2        Alpha/beta hydrolase fold n...   149     2e-34  1  12.1%  80:417     1:294     -------------------------------------------------------------------------------MTSSLPGWLARLYPFTPRSFTTPRGARMSYVDEGPRSAEAVLMLHGNPTWSFYYRELITALVP-TLRCIAPDHVGMGLSDKPAG-----YDYSLATRIDDVAALVAQLGLTRVHLVVHDWGGAIGFGFAARHPALIGKLVILNTAAFASRHIPRRIALCKVPVIGPLLVRGLNGFAGPA----------------------------------------------TWMAMHRRALTPDEKRGYLWPYSSWANRVAVSAFVRDIPMSPACRSWaaLPHFRDRPALILWGGQDFCFNDHFLARWRELLPAAEvrIADAGHYVLEDAREEVIPQITTFFRG--------------------------------------------------------    
 405 UniRef50_A4A9U6        Epoxide hydrolase n=2 Tax=u...   149     2e-34  1   9.3%  99:421     8:297     --------------------------------------------------------------------------------------------------TLAHGNLRFSAIEGG--NGPLVILLHGFPDTLHTWGEQMHVLAEAGYRVIAAATRGY-----EPQSQPEDGDYSSAALAGDVLAWIDQLGASAAHLVGHDWGASIAYTTAMAAPERLLSLTTMSVPH---------------------------AGRFLAEIDKHPKQLRLSWYILFFQAPGIAEYVVKRKDFA--------FLRWLWKTWSPGwfEEEEFQIVVAAFRAPGVLKSALGYYRAAVGMDSLRGRKSYDGsiEVPTLGMTGKNEGCIAAEVFSAMMrdFPKgvVEVPDAGHFPHRERPEFVNALLLDWLATHDEA----------------------------------------------------    
 406 UniRef50_B2TF21        3-oxoadipate enol-lactonase...   149     2e-34  1  10.6%  98:417     2:261     -------------------------------------------------------------------------------------------------PYAAVNGTELHYRIDGDRHgpWIVLSNSLGTDLSMWASQVAALS-KHFRVLRYDTRGHGHSEAPKGP------YTIEQLTGDVLGLMDTLKIARANFCGISMGGLTGVALAARHGNRFERVVLCNTAA-------------------------RIGSPEVWVPRAAKARSEGMLALADAVLPRWFTADYIER------------------------------EPVVLALIRDVFVHTDKEGYASNCDAIDAADLRPEAPGIKLPALVISGTHDLAATPAQGRELAQSIPGARYVELDASHISNIEKADAFTKTVVDFLTE--------------------------------------------------------    
 407 UniRef50_B7JW73        Alpha/beta hydrolase fold p...   149     2e-34  1  10.8% 100:415     8:280     ---------------------------------------------------------------------------------------------------INVRGVEHYYEWIGQpsKPIMVFIHGWAGSTRYWRKTAQALSDR-YNCLLYDLRGFGRSKLPEIPVNLS--YDLEEYAEDLRGLLDALNIERTYIQSHSMGASVATLFTTMYPERVERVILTCNGIFEYDERAFTAFHRFGGYVVKFRY--------------------------------------------PWFLKVPFADQLFMARFLHRPISKIDRVAFLEDFIIAD-YEAALGTIYTSVSKKAVETMPQKFAEIAVPTLLISGEKDIIIPAEMGRIAAELNKNINYviPKTAHFPMLEDESTYLATVRDFL----------------------------------------------------------    
 408 UniRef50_C6TM11        Putative uncharacterized pr...   149     2e-34  1  13.8%  96:334     5:231     -----------------------------------------------------------------------------------------------EHRTVEVNGIKMHVAEKG--EGAVVLFLHGFPELWYSWRHQILALSSLGYRAVAPDLRGYGDTEAP----STVNSYTCFHLVGDIIALIDSLGVDKVFLVAHDWGAIIGWYLCLFRPDRIKAYVCLSVPFRppKQKTVDFFHSLYGDDYYICRFQEPGKMEAEMAGVDTAYLMKNILTTRKTGPPTFPK-----------GEYGTGFNPVIPDTLPSWITQEILINMLLNSTRLASLEA-------------------------------------------------------------------------------------------------------------------------------------------    
 409 UniRef50_B4UYA7        Putative uncharacterized pr...   149     2e-34  1  12.6%  85:417     4:292     ------------------------------------------------------------------------------------PPTAPRGRRKTVQDFTTGDGVRLAYRDSG-GEGTPLLMLHGWGQTQEMFRHQFEEFS-AGRRVVTVDLRGHGRSAKP------HHGYRIARLSRDVLELVEHLGLSAFDALGWSMGASVWWSFIDQYgdHIRRLVIVD----------------------------------------QPAAVAAVPWMTEAEQRESGAIFDVTGLLDLGAALAGPDGRRARQEFVRSMFTGDTTDAALLAFVAEEIESTPAYAGVPLLFDHCAQDWRDVLPRIDRPTLVL-GCEGSHVDPHSQRYIAARIPAAELHVFpaSHFPFLENPDEFNTVLEKFLTR--------------------------------------------------------    
 410 UniRef50_C1D241        Putative chloride peroxidas...   149     3e-34  1  10.9%  87:417    19:292     --------------------------------------------------------------------------------------QAGGAEAGRQEGMLDVNGARIHYVSQGA--GTPMLLLHGYPLSGELFARNRDALAAAGFRVITIDHRGYGQSTAPASNPGN-----LQTYAADALAVMDQLNVPKAIVGGMSMGGPIAFEMWRTAPERFSGLILINTIANPAG----------------IVEQNIWKGMAQKASTFGPQSLAPELLKDMLTGQ-----------------------------------TRATRPADVAFLTTIVKQASVAADVAGATVLATRPDSIPTLKTITVPTLILEGREDTVYPPEYSLKMQQNIPGSALviPGAAHAAIYEKAAAANQAILTWART--------------------------------------------------------    
 411 UniRef50_A6SIW9        Putative uncharacterized pr...   149     3e-34  1  12.5%  90:417     2:334     -----------------------------------------------------------------------------------------ASSDFPTRTHTTTRSFTYSYIHIQppSKSYILFLHGFPSSSRDWRHQITYFSSKGYGIIAPDLLGFGNTSKP----LSMHSYKFKDMASDLYEILSIHSIstAIGVGHDWGSPMLSRMVNYYPELFKKLVFLDVGYQAPDGPAFNFEteAAGFEIFGYFFLMNEEDGTELLNKNPESVMSLYYSLDSEIGKEYMG---------ATGGFRKWLEGGMLAPYPDFIKSEDVEHFKNMFSPkgGFGGAKNWYTMQLHGINEGDDKAITKevHQPTLLITSTNFISSGADFPSQMRPYIPNFRVERWECGHWIQLEKSDETNALLGEFFEE--------------------------------------------------------    
 412 UniRef50_B6IQI9        Hydrolase, alpha n=8 Tax=Pr...   149     3e-34  1  10.8%  46:424     3:314     ---------------------------------------------LSLPFLVVLVASLFGLGVWLHTPDRPRDELEALYLRSPAD-------------YVQAAGMRLHVRDDGPRDAPAVVMLHGFGASLHTWEGWAQGLAGP-FRVVRFDLPGFALT-----GPDPTGDYGDERAMVVLEALLDRLGIARASLIGNSIGGRIAWKFAALHPDRVEKLVLVSPDGFASPGFEYGRKAEVPGILNLMRFILPTAAVRANLQPAY---------------------------------------------GDPAVLTDQLTTRYRDLMLAPGVRDAMF----ARLEQVMLEPPEPLLRRIQAPTLLLWGEKDAMIPVSNAADYARALHDSrvTFPDLGHVPQEEAPARSLEPVRKFLAEGYPGLDG-------------------------------------------------    
 413 UniRef50_B4WLL5        Hydrolase, alpha/beta fold ...   149     3e-34  1  11.2%  77:415     2:307     ----------------------------------------------------------------------------VATAPDPITPspFVVPPARNGAYWTWSGYRIHYVHAGepERPPLLFIHGFGASTDHWRKNISGLQDR-FEVYALDLIGFGRSSKPSS------GYSPQLWRDQISTFIKDIIGRPVVVAGNSIGGYSSLYTGATCPAEVVGVCMLNGVGSFSEQQLDQ--------------------------APNPFREAIGQLIKTVVLSPFPSWVVFQ------FVRKKSYIRRTLEQVYVNK--EEVTEQLVEDVYRPATDPEAPAAFAALFKAERGEYVDVLLSQMSCPLLLIWGDADPWmtYSRGELFQKHYANLEEHHINAGHCPHDDAPVEVNALLSEWV----------------------------------------------------------    
 414 UniRef50_Q28P25        Alpha/beta hydrolase n=1 Ta...   149     3e-34  1  13.2%  76:417    20:324     ---------------------------------------------------------------------------PVAAQDTGGAQQPISAEFPFELQTVEVLGSNMAYVDTG--DGPVVLFIHGNPTSSYLWRNVIPHVAED-HRAIAIDLIGMGASDKP------DIDYTFQDHYAHLEGFIDALELTDITLVLHDWGGGLGTYYAANNSDNVRAIAMMEAAAPPALPIPDW------------------AMVADQQTRETFQAFRDPVM------------------GPQIILEQNGFVEGLLPATILRTLSDAEMDAYRAPFPTPESRQPVLMWPNEIPIEGTPArvAAWLTTSEQPKLILYASPGLIWSPEVADFAARTFNNTEARFVGAghFIQEDQPEAIGRNLSDWLRD--------------------------------------------------------    
 415 UniRef50_A5VBI9        Alpha/beta hydrolase fold n...   149     3e-34  1  12.1%  96:418     5:321     -----------------------------------------------------------------------------------------------THRHARINGIAMHWVEQG--EGPTIVLCHGFPHIWLSWRHQIPVLAAAGWRVIVPDMRGMGQTEAPAD----HRLYDVPHVTGDLVGLLDHLGLEQAVFAGLDFGIFAIYDLAYLHPDRVWAVIALENPAYPDSPDKAPLAEAAEWAKEHFVHIDYFRPVGPADAALDAAPREFLRRVLYALSGDFHYLDVWKHPPGTAYLDALPE----APPLPWPWLSEWELEWYVADYARSGF-TGGLNWYRAMDLRWAQRAAWRHAPT-TRPFFFIGSENDVDLEAWHGDDPLGAIphADVrlPHAGHLIQLERANEVGRLMVEFLGEL-------------------------------------------------------    
 416 UniRef50_A7HQ29        Alpha/beta hydrolase fold n...   149     3e-34  1  11.5% 105:417    52:327     --------------------------------------------------------------------------------------------------------LRLHYVDWGNEGAPPLLMVHGGRDHCRNWDWTAQAMRDR-YHIIAPDLRGHGDSQW-----MVGSGYELNDYVYDIAQLIHQKGLAPLTIIAHSMGGAVALRYAGLYPETVKKLIVIEGLGPSPKMLSERKEISADQRIRNWI------DEVRKLSSRQPRRYDSI-----------------EDAYKRMQEENPHLSPERARHLTVHGVNQNEDGTYSWKFD---------NYVRAFSPMGFDEETSTKlgRITCPTLLIRGTESWASDP-VADGRAKHFQNAKVvvEGAGHWVQHDKLDEFLRLTDEFLAE--------------------------------------------------------    
 417 UniRef50_B2IBQ5        Alpha/beta hydrolase fold n...   149     3e-34  1  12.8%  54:420    71:377     -----------------------------------------------------LPVGSLIAIIADRETSEDEIDRFIDKFQAEfeQDKSETVAAPEPRVIEVSGQKIRILEIGNGEGAPIVFVHGFGGDLNNWMFNQPPLAEK-HRTIAIDLPGHGGSVKETSG-------DIASLAKSVGETLDALGVASAHFVGHSLGGAIILALALERPTLVASLSLISPAALGDVINTDYTDG---------------------FITADRRKTLKPVLQLLFADP--------------------------------SLVSNDMIDDLIKFKRLDGATTALTAIASafKEGRQSLNLLSRLAEIKAPVEILWGKQDQVVPMVDPGQLPGTI--AFHPLEKSGHMPQMEQSAEVNGLIGDFITKVET-----------------------------------------------------    
 418 UniRef50_B3TA11        Putative alpha/beta hydrola...   149     3e-34  1   9.5%  93:419     5:276     --------------------------------------------------------------------------------------------REPVDGYIEVDGLRLHYLDWGNEDAQPMVLLHGLQDCARSWD-VFAASARYSHRVLALDHRGHGDSDWSTA-----DRYRLRDYVADLEGLVEHLRLQSVVLIGHSAGGRNAMLYSIDHSDVVEALVVVDIDPDAINPESQRMFGLYNNETDEW--------------------------------------------------GSVEAVVERLRSRQPNSSEDTLAHQALHMTRELSSGGRVWKRDRQLLAAYERPDLWAVWSRLTCPTLIVRGRQSPLLTHDVAVRMREAIPRARleLEGGGHWFYQEFPGAFEATVRWFLQDPP------------------------------------------------------    
 419 UniRef50_O31168        Non-heme chloroperoxidase n...   149     3e-34  1  10.9% 105:416    14:277     --------------------------------------------------------------------------------------------------------IDLYYEDHGA--GQPVVLIHGFPLSGHSWERQSAALLDAGYRVITYDRRGFGQSSQPTT------GYDYDTFAADLNTVLETLDLQDAVLVGFSMG----------------------------TGEVARYVSSYGTARIAKVAFLASLEPFLLKTDDNPD----------GAAPKEFFDGIVAAVKADRYAFYTGFFNDflDENLGTRISEEAVR------NSWNTAASGGFFAAAAAPTTWYTDFRADIPRIDVPALILHGTGDRTLPIetARVFHKALPSAEYVEVEGahGLLWTHAEEVNTALLAFLA---------------------------------------------------------    
 420 UniRef50_C7P1A7        Alpha/beta hydrolase fold p...   149     3e-34  1  10.0%  96:415    10:279     -----------------------------------------------------------------------------------------------TTLSVDGDDVTVRYLTAG--DGPPLLFLHGIGLDAasGRYALPALAEE-YTVYALDFPGHGESEKPRRT------YTTDYYVDTLSAFVDELGLAGASVVGVSMGGAVALGHALDGG-RPERLVLVDSY----------------------------GLGADAYWRTGASLALRIPFADSLLWGSM---------------GTRAAVRTSLRTMSGATLPDELVDDVYETIS-PATMRTLRSWQRhfQADGLRTDYTERLSELDVPTLLVHGSADPLLPVSWSQRASELLPDGqlAAEGCGHWPHREHPERFNRAVTAFL----------------------------------------------------------    
 421 UniRef50_C8NXG4        Putative uncharacterized pr...   149     3e-34  1  13.8%  83:416     4:305     ----------------------------------------------------------------------------------PISPNTVALPGPFEHVMLHTRGIRLHAVVAGDPRDPLVLLIHGTFGAWLDFRHVIEPLAQEGFHVAAVDMRGYGMSDKPPSRPGDDTL----LAVGDIDGMITALGHERAHLVGHDTGGALSWVYSAAYPHRVDSLVAVSAAHPYDLRSYMRRRPWQLIYMTTRVAIGRLPSWFLRWFAGAIPQVWRSELTINTQPGFHRSDAFT-----------------------------ETLALRVQAASIHNALRGIIHNSRMLTplISLPNKANANAPTLAPVLLIHPPQDVWTRIDELSSARTRGPLARTSIPGAKNVPHVENPQGFVEALTNFLS---------------------------------------------------------    
 422 UniRef50_Q0JWC8        Putative hydrolase n=2 Tax=...   149     3e-34  1  10.7%  53:415     1:318     ----------------------------------------------------MLALGTGATALAVTGSPAAAHPGPHPGPVPSDRELARSLPGGFRSRHATVGGVRLHYVSGG--HGEPLLLVPGWPQTWWAYRKVMPQLARR-YHVIAVDLRGMGGSDKPAG------GYDKKTMAADLHALVRGLGHRQVNVAGHDIGSMVAFAFAANHPEATRKVALLDTPHPDQSEYEMRILCRPGTGTTLW-------WWAFNQLQALPEQLMH--------------------------GRMRHVIDWLYalADQSLVGDLDRDIYANAYNSPQAVRAGTRWYQACHQDITDQ---AGYGKLTMPVLGIGG---NFTFEDLRNKLTAQATD--VHMVRAshYLPEEEPDVVAGALLDFF----------------------------------------------------------    
 423 UniRef50_Q1AZR4        Alpha/beta hydrolase fold n...   149     3e-34  1  10.8% 104:418     8:265     -------------------------------------------------------------------------------------------------------GVTMSYREMGAGAEDPVILLHAFPLNGRMWEPQVAALAGER-RVITPDYPGFGR--APRTPAQPDVRY----YAEEVRSLLDRLELERVVLGGLSMGGYVAFECLRLFPERIAALVLADTRPDPDT---------------------------EEMKESRRELARRVAEEGVGVLAQTQPRRLLCERTLEERPEVVERVKGMILE------------------STPGGVVAALGAMR------DRPDSTPLLESIRVPTLVIGGEEDAISTPETMGEMAKKIPHSRHVvpRAGHLSNLENPEGFNAALGELLRSL-------------------------------------------------------    
 424 UniRef50_B1G753        Alpha/beta hydrolase fold n...   148     3e-34  1  12.0%  97:429    60:342     ------------------------------------------------------------------------------------------------SKFIRIMGADVHYVDEGRGD--IIVMIHGFASSLHTWNRVADEL-KHAYRIIRLDLPPFGVTGPLRSSTGAIETMDLPTYQRFIDTFLQALGITRATFIGNSLGGLISWDYAVRHREAVERLVLIDSAGFP-----MRLPIYIGLFNSALVRASSPRWLPEAIIKSAVRNV----------------------------------------YGDPRKIDAVTLRRYVEFFHGEGTRAAIGKMVPTLDFAELDTNV---LTTLALPTLVLWGAKDRWIPTAHAAEFAQRIPGAKSVMYPggHIPMEEAPERVLTDLRAFLGTHAAPKAGAVARA--------------------------------------------    
 425 UniRef50_A8DJC3        Homoda-hydrolase n=1 Tax=Ca...   148     4e-34  1  13.3%  72:417    15:309     -----------------------------------------------------------------------ALREQRETKPPYPAVFATMREFAERARFILLGSTWHHVYEYGPVDGPAVLLLHGWDCSSYWFYRLAPALGKRGFRTIAFDFRGHGFSD-----AAPDNDYTLPTLAQDTLCLADCLGVQQFHLVSFSLGSAVALLVGSLAPERVGRQVVMNFGLFDYVALRAQFLPAILKTIFDTLRRIPSKTFVYRYV--------SLTLAQREVTWCDVEMGFTSLVYCDSQAAFLA-------------VTDLVQQERVAQVQRAAGDA--------------------------QPTLVITGEFDPVVPFADSLRLAQTIPAAHLHIlrTGHLVLFERPDEIERLTGDWLTA--------------------------------------------------------    
 426 UniRef50_C8ST97        Alpha/beta hydrolase fold p...   148     4e-34  1   9.9% 105:418    12:295     --------------------------------------------------------------------------------------------------------LRIAHFSGGPQYGVPVVLLHGWPDDATTYARISPALHDAGFRTFSPWLRGCGPTSFLSKETMRSGEIT--AMAQDVLDFADALGLSRFAIVGHDWGARIAYLLASVFPDRIKCCAALSLGWQPGRLVTPSPEQAKAFWYQWFMATERGA----EFVRKNRKEFARFQWDSWSPLG---------------------------------WFDDAIFDEVAKSFENPDWAEVTLHSYRVRWEEAEPDPRYAELAriTVPTLTLHGGDDRVVLPRSSEGLEGHFTAsrLVLDAVGHFPTREAPQEVSRLLVNFLDSH-------------------------------------------------------    
 427 UniRef50_Q83ZF0        2-hydroxy-6-oxo-6-(2'-carbo...   148     5e-34  1  12.7%  97:420    15:301     ------------------------------------------------------------------------------------------------SFWVDLLGTEVRYRQaeAGAGEPVVLLHGVSGHAETWVRNVAVL-GRDFRVHAIDMLGHGFTDKP------QIEYSIRALAEHVLGFLDEIGASRAHLVGQSLGGWVAAFLAVHHPERVASLVSVTGAGLQVDADGATLTENVG----------RQVAEATTKALDTP-------------------------------TREKVRTRLEWLVHDPSVVTDELVETRYRIYASPDFAATAGDMVAAFTSRPRPEElaERLATINCPTLVLWTRQNPTMPWPVGEAASRIIPDATFrmEDAGHWPQFEKPAEFHAVVGGFLRSVTA-----------------------------------------------------    
 428 UniRef50_B8GZF4        Dihydrolipoamide acetyltran...   148     5e-34  1  11.8%  90:419    13:294     -----------------------------------------------------------------------------------------PREALMTEQWITVGDLRIRYVDSG-GEGIPVFLLSGIGASLEFWSNQLEALGER-LRLIAWDYPGHGLSD------GDGRSHDPDRYAAFALDVMNALGLERVVAVGNSLGGAIALRMAGLAPDRVAGLMLASPAMMGPEVFL-------------------------------PFRLMSLPLLGELM--SKPGKLSVEQQIAALFHDSASATEALRRIVWRNVHKDGAPQALLATMRETLWIGGV--------RKVHWARSRALLKSATCPILFIHGKQDVVLPFQQSIDCAKLNPRAEvvIDGCGHTPQIEIPETFNAEMKAFARRVD------------------------------------------------------    
 429 UniRef50_Q944I9        At2g26740/F18A8.11 n=1 Tax=...   148     5e-34  1  14.1%  96:279     2:188     -----------------------------------------------------------------------------------------------EHRKVRGNGIDIHVAIQGPSDGPIVLLLHGFPELWYSWRHQIPGLAARGYRAVAPDLRGYGDSDAPAEISS----YTCFNIVGDLIAVISAleDEKVFVVGHDWGALIAWYLCLFRPDRVKALVNLSVPFSFRPTDPsdRMRAFYGDDYYICRFQEFGDVEAEIAEVGTERVMKRLLTYRTPGP--------------------------------------------------------------------------------------------------------------------------------------------------------------------------------------------------    
 430 UniRef50_C1E7V5        Predicted protein n=2 Tax=M...   148     5e-34  1  15.4% 108:418     4:289     -----------------------------------------------------------------------------------------------------------AYRRRGN-SGKPVVLIHGFGVSSFQYREQLSALSKNN-KVYALDLVGFGKSDQP------DLEYCMEFWRDQVVDFVDNVVGEPAVLVGNSIGSLTAIHAAAKKPECTTGIVLLNCAGGMNNKVKRMPGDFDGFGWQY-----------------------KAVIPIFNVVLAIIDFVLKTPVAKPLFDNVRNEESVRNALKGVYKDSSRVDDALVQSICTAAEREGAFGAFVRILTGPPGPRPEELMPNVKCPMLILWGDNDTITPPdlGQYFMKlrPNTTLKVFEggHCLQDDNPGAVNPVIKEWVAAL-------------------------------------------------------    
 431 UniRef50_A6UA38        Alpha/beta hydrolase fold n...   148     5e-34  1   9.7%  97:417    15:272     ------------------------------------------------------------------------------------------------RTVVLPNALRLAYIEMGDPNGVPILLLHGFTDSARSWSLAAPYLAP-GFRVVAADLRGHGNSDQPEGC------YTIPELANDVRLLMVALDLAPCHLVGHSLGGRLVQALAERWPHLVRKIVLMSTS--------AALRERQGWLWENIQMLSDPIDPESAFIR---------------------EWCSGAGLIDVDFLAHARRESAALPSRIWHSIYYEQLAY----------------------------DPSPLLQDISAATLILRGEKDTIATEEHQVQMKDAIAGAEIilPEHGHNIHWEAPEKVAHLILTFLDR--------------------------------------------------------    
 432 UniRef50_B6JCJ6        Hydrolase n=1 Tax=Oligotrop...   148     5e-34  1   9.1%  92:421    11:280     -------------------------------------------------------------------------------------------PPLPERQIAATPGGTISYREAG--QGPALFLLHGMNGNSRSWTQQLAAFSD-SYRVIAWDAPGYGQSD-PVLPEADAYAAQAAH-------LLDHLNVEKAHVIGHSMGGVIAERFCARHLDRAASLVLSGTHWGNAVPE--------------------GTPLAAKYARRLRELEEMSAEEYGKVRAGKMVPSSPSPSPDVL----------------------DELAGVASEMKREGLFNGG--------QMVEKTDNRPFLSTLRLPVLILTGSRDPVVTPQRSEAMMEYLPSARTvlPDVGHAAYLEAPEAFNRAVREFLATVERS----------------------------------------------------    
 433 UniRef50_B3QIH0        Alpha/beta hydrolase fold n...   148     5e-34  1  11.1%  86:419     2:302     -------------------------------------------------------------------------------------PDLADLFPGFGSEWINTSSGRIFARVGG--DGPPLLLLHGFPQTHVMWHRVAPKLAER-FKVVVADLPGYGWSDMPESD-EQHTPYTKRAMAKQLIEAMEQLGHVHFALAGHDRGARVSYRLALDSPGRLSKLAVLDILPTY--EYWQRMNRAYALKIYHWSFLAQPAPLPENLLGGDPDFYVKAKLAS------------------------------WTRAGDLSAFDPRAVEHYRLAFADPMRRHVMCEDYRAGAYADFEHDKIDVEakIPVPMLALWgaQSAATPLDVWRKWASDVQGA---PIESGHFLPEEAPDQTAEALVRFFSAAP------------------------------------------------------    
 434 UniRef50_Q4V060        Haloalkane dehalogenase n=9...   147     6e-34  1  12.1%  90:419     4:299     -----------------------------------------------------------------------------------------PGYSFTPKRLDVRPGIAMSYLDEGPSDGEVVVMLHGNPSWGYLWRHLVSGLSDR-YRCIVPDHIGMGLSDKPDDAPdpRYDYTLQSRVDDLDRLLQHLGigPITLAVHDWGGMIGFGWALSHHAQVKRLVITNTAAFPLPPEKPMPWQIAMGRHWRLGEWF--------------------------------------------IRTFNAFSSGASWLGVSRRMPAAVRRAYVAPYDNWKNRISTIRFMQDIPLSPADQAWSLLERSAQapAFIAWGLRDICFDKHFLAGFRRALPQAEvaFDDANHYVLEDKHEVLVPAIRAFLERNP------------------------------------------------------    
 435 UniRef50_A1UQA3        Alpha/beta hydrolase fold n...   147     6e-34  1  12.7%  96:425    32:316     -----------------------------------------------------------------------------------------------ETRFVDVDGVRTRTIQAG--EGPDLILLHGGGGHAEAFARNVTALS-RHFRVHALDLLGHGLTRGCEVAPNR------KDYVSHLLGYMDQEGIDRAHLVGESLGGWIAAWTALEHPDRVDRLIYLCGA----------------------RLTLEVGADAEARTAAGRAELARVT----------------RQFLADPSPANVRERMAWLFHHPDRDLTDELVALRWALYQSEESRSALTNATAPPSAATAEDNlaERLTSLTRPTLVLWTSHNPSATVEFGRRAAELIPGAEFamEDCGHWPQWERPEEFNQILTNYLQGDRESRGQR------------------------------------------------    
 436 UniRef50_A5VBJ1        Alpha/beta hydrolase fold n...   147     6e-34  1  11.5%  96:418     9:351     -----------------------------------------------------------------------------------------------ERKIPVGDGLHLHAVEAG--EGPLLLMIHGFPGLAWSWRHQMLPFAAAGFRAVAIDSLGYGGSDRPLDPAL----YASDRMQAYLLALLDHYGADRAVVIGQDFGAQYAWNLAVRAPGRVRALVATIPYDYDLAGRagAAERLPPGAPARPDMASPDHRPSERFAAMAKAHFVHFHYFQTVGPADRELGGALADYLRRSFHALSAAGDLWAWKALPswLSEAEFAAFVARYDHADPArgGLNSYRTADRNWEIGRAWAD-ADVTVPTLMLLGAADpaFFPDWEDRLRRRVPGLavAVEGAGHLVQQEKPEAFNAAVLDFLGGL-------------------------------------------------------    
 437 UniRef50_D2R463        Alpha/beta hydrolase fold p...   147     7e-34  1   9.2%  93:418     9:294     --------------------------------------------------------------------------------------------YPFQSHYFEREKLRYHYVDEgsAAKRPLLFVHGNPTWSFYWRELIAGL-KADYRCIAVDHIGCGLSDKP-----QDYPYSLAKHTENLVALVEKLDLKNCTLLAHDWGGAIGLGCAAQLRERFSQLVLFNTAAFPPPYIPKRIQLC-----------------------------RVPILGTLGVRGLNLFARAAITMATEEPQRMTAAVKAGFLAPYNNWHNRVAIDAFVKD----------IPWTRAHPTYQVLEDLETKLAKIThqQLLVWGMRDWCFRSECLDRLKRTFTQAEELRFdaGHYVVQDRPAEILERLHTFLAQV-------------------------------------------------------    
 438 UniRef50_Q2S803        Predicted Hydrolase or acyl...   147     8e-34  1   9.4%  88:397     1:283     ---------------------------------------------------------------------------------------MTHAYYHPREQYLKVNSIQICYEELGDPDGEPMLLIMGLACQMTAWPpfLEPLIEAGYRLIRLDNRDIGHSsrSKLGLPVTASYTLYDMADDAIALLDALNIERAHLVGVSMGGMISQIVAARQPQRIKSLTLMMTSNNSPKQPMPDLGTLWRINGGGVRGHHQ-----------------------------------------EAALRRGVAFWETVQSPSFPTPKERILQRIARDYQRSYRPKGIVRQMRAILATGSLEKLA---CNITMPTLILHGSADPLMKPRNGHMLKQSIADAKLEMIPggH----------------------------------------------------------------------------    
 439 UniRef50_B8KMA2        Putative epoxide hydrolase ...   147     8e-34  1  13.6% 100:416     8:280     ---------------------------------------------------------------------------------------------------ILINDVSYHVYDEGDSD-RVVLLLHGMPDTSALWEHQAASLVAAGFRVIAPDMLGYGETDKPQATA----RYAAEHILADMLALFEHLKIAKMDVVGHDWGAFVAWELVLLKPELFRKHVALAVGHPGLMFGNKSPESVKENWYMYL---NTQEDTAALYAENNFQFMRESFI-----------------------------------------PSHPDLDEVTARMSDPLAMNAMLNWDRANQVASFYLAHGreYGNCQVPTMGVWSSGDTYLWETWMTDTETLM-DAEwrIEGASHWMMMDEPETVSKLILEWFS---------------------------------------------------------    
 440 UniRef50_Q0VL04        Oxidoreductase, putative n=...   147     8e-34  1  10.6% 100:459     9:330     ---------------------------------------------------------------------------------------------------VSRNGIDLAVRTWSPDKSPTVVLVHGYPDASHVWEKVAERLS-RDFKVVAYDVRGAGNSSTPKNRA----AYKLSQLRGDLHAVMDAVCPdkVHLVGHDWGSVQTWESVTE----------------PNAEHRIASYTTLSGPCLDHVGQWMNARLREQKIGAVLNQLAHSWYIGAFQLPVLAPALWKVGLAKVW----PQVLRRTENLYSEASVT------------QLQDGTHGIELYRA---NMLPCLLKPRERYTKVPVQLIVAREDNYVRPAMLEDLPQWTEQLWRRELDCGHwpLLQHPDVTANWIREFIHHIEGAPAAGPLQRSKVTTGHPTGPDSGKRVIITGAGSGIGRET--------------    
 441 UniRef50_A0YAD1        Putative hydrolase n=1 Tax=...   147     9e-34  1  12.1%  95:415    13:306     ----------------------------------------------------------------------------------------------FERFYFTHSNCQLSFVDFGSPDKPALILLHGMRDHALSLLNVAQAL-KNDFHVVALDMRGHGRSDNP-------GIYTMIHYVADVRALVQYCGLDKPVIVAHSMGGHIASRYSAAFPDEVDRLILLDGMGPPDWTDKPDIN-HLKVGLRHGVDAVSSLYIEGRQMPDRNEAIRRLRDNNPLLSAD---------------------------------LAEIIVEHGVDTHSQGGIRWSFDPSVQMIWHTFPHHESEDVWRGIDCPVLIVTGSddqvFYTASLQRKQQLFTDARhvVDGAGHMLHYDQPEQLNGVLRDFL----------------------------------------------------------    
 442 UniRef50_A5UU73        Cyclic nucleotide-binding p...   147     9e-34  1  13.7% 101:429     5:288     ----------------------------------------------------------------------------------------------------TIDHQAIYLEESGPENAPYAFLIHGWASSSYTWKPILPALSRR-YRCIAIDLPGFGRSPVPLHPP------TIPWYADLVARLIDYFSpqPVLLLGHSMGGQIGATLALHYPLIVERMVLLNPALSGRLSTRVNLL----IGPHVLAERFLPLEWLLHLLARTPLDYTDYLLKPSN-------------------------------FAERAQVSDEDYQRIRADARRPGQGR----MRAACFQAMRQGDLRGKLAAVEPPALVIWGAEDNIVPLRDAGVVAAEWPAADLRipNAGHWPQFEQPDATLRHIALFL-GLPPAGAALPADA--------------------------------------------    
 443 UniRef50_A9B5A1        Alpha/beta hydrolase fold n...   147     1e-33  1  10.0% 100:418     4:261     ---------------------------------------------------------------------------------------------------IDVRGHSLMYDDGG--DGEVVLCIHGFPFNRSMWDEARLALASR-YRVLSPDLRGFGESS-------GSESWTLDDQANDLIELLDQLGIDRVAVLGLSMGGYIALNLARRYPERLWAMVLIDT----------------------------------------------------KATSDNYDAKQNRLKTAETALREGAAPIAAQMLPKLLSPANADDQRLIERLNSMMLTTNPKTIASAAHAMASRPDSTPYLSTMALPSMVIVGNDDQITTPNDAHAMVAALPHASLviPDAGHMSVLEQPEIAYGAIRVFLEQA-------------------------------------------------------    
 444 UniRef50_UPI0001AF5581 haloalkane dehalogenase n=1...   147     1e-33  1  11.6%  76:412    42:328     ---------------------------------------------------------------------------EVFRTPEGRFAHLPG--YDFTPHYVDVDGLRIHYLDEGPREGTPIVCFHGEPSWVYLYRKMIGPLVAAGHRVIAPDYAGFGRSDKPTDPNW----YTFDRHVQIVSAVLDRLDVQQATAVVQDWGGPIGLRWAVEHSDRVAALAIFNTGLFTGRVSKGFLSWR-------------------EFAEKNPDLPVGMVIQGATA----------------------------------TDLADDVVAAYDAPFPTPESKAGAARFPLLVPITGDdrAVIDQLSRWQKPALVAFSDQDPIFppRAGQVFCDLIPGaqVRIEGASHFLQEDRGELLAHEVL-------------------------------------------------------------    
 445 UniRef50_Q1IU18        Alpha/beta hydrolase n=1 Ta...   147     1e-33  1  12.9%  93:418    11:288     --------------------------------------------------------------------------------------------QTTEHDFTLPTGHRMHYRKVG--NGPPVVLIHGLIASSFSWRFNLPALA-QHFTCYAVDLLGMGDAERPSGV-----DVSPRGLAEGLVAFLKAQSGGPWSIIGTSHGGAVAI-------------------------WLARLARAAGLELNRLVLSAPANPWSQ-----HGRRLAPFAAHPISRAVVKASRWAYIPVRRMTFSRM---------YGDRRLITKETLAGYARPLKIKGTVPHCLDLLKNWVRNVD--ELEGVMRGIDVPTLLVWGTKDRLVYFSSAQRMLETIPDARLliEGAGHLPYEERPEEWNAAVVPFLRDA-------------------------------------------------------    
 446 UniRef50_Q59695        Dihydrolipoyllysine-residue...   147     1e-33  1  11.0%  84:418   100:370     -----------------------------------------------------------------------------------VAEGGADQAQGPAPQKAEVGGRLLRWFELGGEGGTPLVLVHGFGGDLNNWLFNHPALAAER-RVIALDLPGHGESAKALQRG------DLDELSETVLALLDHLDIAKAHLAGHSMGGAVSLNVAGLAPQRVASLSLIASAGLGEAIN----------------GQYLQGFVAAANRNALKPQMVQLFA-------------------------------------DPALVTRQMLEDMLKFKRLEGVDEALRQLALAIADGDrrHDLRSVLG--QHPALVVWGGKDAIIPASHARK----GPEAelVLPEAGHMVQMEAAEQVNQQMLAFLRKH-------------------------------------------------------    
 447 UniRef50_B5K859        Soluble epoxide hydrolase n...   147     1e-33  1  11.0%  97:414    14:299     ------------------------------------------------------------------------------------------------HGRARVNGIRMHYVIAG--QGEPLLLLHGTPKTNYYWHKLIPYLTE-YFTVVAPDLRGFGYTDKPP----VSEGYDSRTNATDLAELMSYLGHETFHVHGEDRGAEFAYVLAATQRDRVKTLSFCEMLLSGEGLEEASFFNLENITAQFRQSGVWVWHIPFFWIPHLPE-----MLINGKEREFWEFWIK-------------------AETWNPNSITDEAMNEWVSCLKSPGGLRGTLETYRAAFKNAEIVREL-RTEKLTLPIMTVGAPE--FFGPLVEEQMVRLSNGVErvFEECGHSIALEAEERLAQMLREF-----------------------------------------------------------    
 448 UniRef50_B6Q3A3        Epoxide hydrolase, putative...   147     1e-33  1  12.5% 104:416    15:299     -------------------------------------------------------------------------------------------------------DVTIHGVHGGS--GPALLLLHGFPQTHHIWHRVADELA-KSYYVVAIDLRGYGRSSKPDGG-EGHVRYGKKAMAKDAVGVMNElgEDSFYVCAHDRGARVTHRLCVDYPHLVKKAILLDI--CPTLAMYTQTTQAFAQAYFHWFFLIQQSPLPEISISTNAQVWVEIFMGG--------------------------------RYAGLAPFSEECLAEYKANLADYETVHAMCEDYRASATIdeAEEDIAAGTKVQCPVRVLWGKYGVvvLEEWRKVSTQSVDG---KAIESGHYIPEERPEEVIANVLEFLK---------------------------------------------------------    
 449 UniRef50_B1MHV4        Probable hydrolase n=1 Tax=...   146     1e-33  1  12.2%  99:417     6:278     --------------------------------------------------------------------------------------------------TFTNDGLTFDVIDEGALDGPVIVALHGFPERATMWEEVIPQLTGAGFRVLAPNQRGY----SPGARPVGVRNYAVDKLEGDIIALVDQAGVRKFHVLAHDWGAAVAWQLAGNHADRVQTLSILSVPHP---------------------------------------RAFQDAMLRGQLLHSWYMLLFQIPRLPEWLFGFNETEAFKKFGKSWMKVSDRLAERIRELFSEPGATTATVNWYRAMRYSLRSPARPSR-----VPTLFIWSDGDSAVTRAAADATPKYvpYRFEVFEGVSHWIAEERPLETAALVLEHIAE--------------------------------------------------------    
 450 UniRef50_C8RV15        Hydrolase n=3 Tax=Corynebac...   146     1e-33  1  12.5%  89:417     8:306     ----------------------------------------------------------------------------------------AADPHTPHATFVHSRGVRLRVDVQGPRNAPLVLLIHGFGGGAFDWHPLMRELAGEDLRLAAVDLRGYGRSDKTP------RGYDLTTAASDMAGVIRGLGHTTATVVGHGFGGMVAWTLVAHNPERVRSFVTLS-----------AINPLLRFRRILAQPFSQPHFARRLLSAQLPRLPERKLLADNAAAAEKIFRAGVAPGFRDTDAYFRSAT--LRREAMQVDKVAHLSCEYLRWPFRSRFRPEAMRFERTFP------------ATTPVPLLAVDGNMDPIYDESLAQKSARKADTAEhvLYGVGHYPHVEDPPCVAELLREHLRA--------------------------------------------------------    
 451 UniRef50_D2SAQ7        Alpha/beta hydrolase fold p...   146     1e-33  1  12.5%  94:422    10:288     ---------------------------------------------------------------------------------------------PVTEGTVTVAGIRSPYLTAGPLDaeAVVFVHGNPGPAEDWRRLVAR-TGVFARAVAPDMPGFGGADKPDG-----FVYTVDGYARHLAGVLDELGVSRAHLVLHDFGGPWGLAWAADHPDRFASATLVGIGVLRGYRWHS------------------------------------------------MARIWRTSGLGELFLRTspASRLLLQRGSPRGLPRDVVERLHRSLKDPGTQRAVLRLYRATDVAAVSEDLHRRLQGLDRPVLAVWGRHDPYLPVAYAERQRETFPGAQVVVldSGHWPMYDDPVGFEQTVLPFLARVTAGA---------------------------------------------------    
 452 UniRef50_A6G9J1        Putative hydrolase n=1 Tax=...   146     1e-33  1  12.4%  85:456    41:365     ------------------------------------------------------------------------------------SEPWSPPSYWDDEHFVEIAGERICYLEAGPpdAptLVFVHGWSGNLQNWWDQYEHFS-LDHHVVVFDAPGHGKSERDPEVS-----YTPELYVEVLDGLFDALDIERATVIGNSAGGYIAAEFAIEHPEHVERLVLSDSTGTRHLGSVAPVLPFLNARWLQIANLTSG----EHYPGQDPKSQARQTFVASFEGTVEEAPYLRV----------------------------------LADLLRPSYARIP----------------KEALASIEAPTLVLWGDDDPIVPVKAMETFEDNIPTVtyVIHLGGHTPMMTSPDEFNCALRTFLSQAplTVQRRRDRLAGKEWGPRYDSAALVDAAESSTPAGQP-----------------    
 453 UniRef50_Q7WZF2        HOPDA hydrolase n=1 Tax=Bac...   146     1e-33  1  10.9%  97:417     7:276     ------------------------------------------------------------------------------------------------EKYVDVDGIKTHYWEVG--NGEPVIFLHGGGAGAdnWHHIMPHFANEGFHVLALDMVGFGFTDTPD---PQEFEYSNDSRIQHVIGFIQALNLRQVNLVGNSMGGAATLGVAMKRPELVKKMVLL--------------------------------GAAGRLKPQNGKESDALQAILNYEYSKEQMYQIARSLTNESYVVSEELVNYRLKLTERSGV----MRAYQAIMK----------WVKENGMYYEDELFR----RIKHQTLIIHGKNDKVVPVADSWEMLNLLENASlvVPNCGHWVMLEYPEEFVTITVHHLKR--------------------------------------------------------    
 454 UniRef50_A0Z5E8        Probable hydrolase n=1 Tax=...   146     1e-33  1  12.7%  97:427     3:308     ------------------------------------------------------------------------------------------------ERYVDANGICLAYDDFGNANDPAILLIMGMATQMTAwlSFCQGLADRGFYVVRFDNRDVGLSEKMLAAYIPStsYKLGDMANDAIGLMDALNLDQAHVVGVSMGGMISQILAAKHPARVLSLTSIMSSSGDPNLPRARKQVTRALLKRYL-------------------------------------------GFVKPSLESTMDFQRLIGSPGFPQDDGELEEKVTAAFQRSFYPAGFARHMAAIMASGSRVDL---LKTITAPSLVIHGSDDVLVPVACGIDTAKQISNARLEIIEggHNLPEsvpeLTAGHVSAVTSALSPSAASGAA----------------------------------------------    
 455 UniRef50_Q1R0U3        Carboxymuconolactone decarb...   146     2e-33  1  11.6%  99:470     3:313     --------------------------------------------------------------------------------------------------FLTVAERTIAYRDSGEAALPAVLLAHPLGMSQDVWEDVVAALRGR-FRCVTWDLPGHGASS------GLTEAVTPRALAEDALALADALGVARFHFVGTSIGGVVGQQLLIQCPERLADVVLTNTGAVIGTPDNWH-------------------------ARASRVRQEGLAAMADEIVPRWFSEAFAQGNAATYTGWKTQLAR------------------------------TDGESYARLCEMLADTDFSGQLRGVAGPVRLLGGREDLSTPPDTLKALASQFGDARLEVLEsaHVPSVEVPEAVAKRLRTWLGDAPVGAEGVSYAAGLETRKQVLGEAHVERASENATTLDAPFQNMITRLAWGELW---    
 456 UniRef50_B5HZJ3        Alpha/beta hydrolase n=3 Ta...   146     2e-33  1  12.2%  90:417     4:270     -----------------------------------------------------------------------------------------WSEPQPDSRFVVVDGLQIHYKRAGR--GPTLVLLHGSASSLQHFDRVAALLSE-SFDVIRPDLPGCGLT-----GPRTDRDYRVPAYAATGADFLETLGVPRYAVAGNSLGGNIAWNLALDHPERVTGLVLVNATGYPEKEVPAGIRLARNPLLRPLLRRVMPRGAIERNLRAH-------------------------------------------VGPHSTIVDDAMVDRAHQLMNRPGNRSAFVDF-----CNTDQPDRSAQIPSITTPTLVLRSAG------VDGQHFTRDIPGaeLVHPHSGHLLPEEEPQWVSDAIAQFLRS--------------------------------------------------------    
 457 UniRef50_Q31PT8        Haloalkane dehalogenase n=1...   146     2e-33  1  14.1% 101:419    23:296     ----------------------------------------------------------------------------------------------------TVGSLEWFYREAEppAKLPVVCLHGLVSQSYGWRQVLPAIADAGFRAIAPDWIGAGFSSQPERR---EFAYTPEAFVSALEAWRAELGIERLHLVVQGYLGAVGLLWAAQHPDRVERIALFNTPFY------------------------------------------------AGASVPWKIRQLGLPLAGEMLCQDPLTVDRVLEGGGGYRVEDADLDVYRRPysSSAAGRALLATVRQLDQARVSTEIASALAQWSQPILVGWGDRDPWLTWVEAEAAAAKLPSAEFvfEETGHYPQEDWPEKLCQALLLFLRRAD------------------------------------------------------    
 458 UniRef50_Q2GWN9        Putative uncharacterized pr...   146     2e-33  1   9.9%  95:416    27:302     ----------------------------------------------------------------------------------------------TPSTLTTRDGIRLTYTQTGPRSGPPILFITGWRQAAIEWRKQSAYFSAAGFRVTTFDLRGHGESE------ESYFGYRISRFAADLDDLLTHLNLHQVAIAGHSMGCSVAWAWWDQYPASRKRVA-----------------------KFVFVDQPAAMVKNPAWSDEYAAEISSIFTPLELFGLANDMSAQLEPLVRGMFTPAVSETDLQWVLEQNRKMSDENSA--------------------ALLVDHAFADWRDVLPRITAKSLVLAGEVS-VFPPRGVEWVAQQIPGAetEAEKGSHFVFWENPERFNEVVEAFLR---------------------------------------------------------    
 459 UniRef50_A6G0Q3        Alpha/beta hydrolase fold p...   146     2e-33  1  10.9%  93:433    10:312     --------------------------------------------------------------------------------------------YPFAEHYFEvpGLRMHYVDEGPREgpVVVMVHGNPTWSFYWRRLIKALSPQ-FRVVAPDHMGCGKSDKPDDA---RYPYRLGRRIEDLGKLIAHLRlgPIHLMVHDWGGMIGMGWAHAHEAEVDRIVLLNTAAFGLPPAKAMPGTLRLTRTGLGTVLVRGFNAFSRGANRY--------------------------------------------CVTRRPLDPAVAHGLIAPYDSWANRRAVLRFVQDIPLAPRDpeVGEALPKFdhPVLICWGMRDFVFDKHFLEVWERKLPSADVhfEDCGHYVLEDAGEEIEALVREFMA---PAVVPTAAQDSAHK----------------------------------------    
 460 UniRef50_Q60DW4        Os05g0419000 protein n=7 Ta...   146     2e-33  1  11.7%  99:423   102:394     --------------------------------------------------------------------------------------------------TWTWRGMRMNYLVRG--EGPPLLLVHGFGASVGHWRRNIGVLSE-SYTVYAIDLLGFGASDKPPG-----FSYTMETWAELILDFLDEVVRRPTVLVGNSVGSLACVIAAAdsRDLVRGLVLLNCSGGMNNKAIVDDW-----------------------------RIKLLLPLLWLIDFLLKQRRIASALFERVKDRSNLKDILLSVYGNKDAVDDELVEIIRGPADGEGALDAFVSTVTGPPGPSPIALMPAVAARS--PVLVLWGDRDPFTPIdvGRYFSAllPNVTLHMLEggHCPHDDRPDLVHAKLLPWLDTLPSTTA--------------------------------------------------    
 461 UniRef50_A0R6X8        Epoxide hydrolase n=3 Tax=A...   146     2e-33  1  10.3%  98:416     7:286     -------------------------------------------------------------------------------------------------RTATVNDVDLAYRIIGDGP-EPIVLLHGWPQTGQCWRKVARPLAER-YTVIVPDLRGYGASG------LASSGYDKQTTAADLSALLRHLGLDSAVVVGHDRGARVAHRWALDRPSEISALALLDV---LPTRVVMRSFDRQSATAMWHWFFHLQPDLPELLVAGNTEAYLRYFLRG---------------------------------VLESGAVDEPTFRHYVDAYSDPAHLTATFEDYRAGFTTDLALDDADHArrLQCPLLLLWGAEGALASADVLGIWREHHAdvTGTPVPGGHYVPEEAPQAVLDALHHFLE---------------------------------------------------------    
 462 UniRef50_Q39XQ6        Alpha/beta hydrolase fold n...   146     2e-33  1  13.8%  98:415     6:278     -------------------------------------------------------------------------------------------------TFVYGPGVDISYRVVG--HGppVVFVHGFAAARTTWNDIVPLFPAERFTLYLIDLKGFGFSSKPRTGS-----YAIEEQAAVTTAFLKAKGLSRVILTGHSLGGAIALLVTLQARDrvARLILVACS-------------------------------------AYPQKLPRL-------MGWLRIPFLARIGMALIPVRTIVRYTLARVFHDTRAITPERIRRYEHCFGRRGMAGVLIRSARAIDPDSYGA-ITARYREIDIPTLIVWGKEDRIVRIGQGKRLAEEMPDARLAVIdcGHNPHEERPRETLAAIMEFL----------------------------------------------------------    
 463 UniRef50_B1ZPH9        Alpha/beta hydrolase fold n...   146     2e-33  1   9.1% 102:420    14:293     -----------------------------------------------------------------------------------------------------AQGIALHAAAIGR--GPLMLFLHGFPECWCAWHRQLPLF-GRMFRAVALDLRGYNLSDMPPNRA----DYALPLIVDDVRRVIRALSPDraVIVGHDWGGIAGWVLARESPELIERMVIINAPH---------------------------PAVFRRELKRSLRQVLASSYAGFFQLRGVAEVVLRAFNFAALRAMV------YRTSAKPDMFPAELRRAYLDTWRRPGWLTAGLNYYRN-PSALTKEAAASGQARIAVPTLVLWGDRDPALRPSNLEGLGSFVAHLTLHrpAATHWIVHEEPDWVNAAIGDFITTVQP-----------------------------------------------------    
 464 UniRef50_Q5FTP6        Putative peroxidase n=1 Tax...   146     2e-33  1   8.4%  98:417     3:269     -------------------------------------------------------------------------------------------------HITTSDGTRIHYEEQG--HGRPLVMLHGWTFSGRFFHRNVGPISEQA-RVITVDLRGHGRSEKP------QHGYRISRLAADLRDLLVALNLQDVTVLGWSIGCPIIW-------------------------SYLELFGSERIRNAIFVQQTPKQYYSADWKLGHAACFDQGGLTAVQQQ---------------LTLTPDEFDRGNLESCICHPLPEDEKALLLREMAQS-----PAHARSAMMADHTTQDWRDILPRFTLPALMMIARKDKVLNPDGPAWVAQHMPNCtvEFEDSAHMVFLDETEKFNRTVLDFLKR--------------------------------------------------------    
 465 UniRef50_C2AP89        Predicted hydrolase or acyl...   146     2e-33  1  11.1%  80:440     1:320     -------------------------------------------------------------------------------MSPEVLPFRSR-PYAPELRFAEIHGHQRAYRMGGS--GPVVLLLHGIADNSLVWEQVMVQLTDR-YTVIAPDLLGHGLSDRPRA------DYSVAAFANGMRDLLCYLGVDRASVVGHSLGGGVAGQFAYQFPDMVERLVFVAPGGVDHDVSPLLRLLSLPFSEQVVALTALPGA----------KQILGAALDVAAALPVPARADLVQ-----------------LRLALDRMPNTASPHAFARTLRSVVDLRGQVVTMRDRCYLTA-----------EVPTLVARGTDDSIIPAAHAEVLRATLPAATVTLFEggHFPMMEAPERFLEVLTGFLTSTEPArrDGLRSAAGVRMPEPAQE---------------------------------    
 466 UniRef50_Q5Z2X4        Putative hydrolase n=1 Tax=...   146     2e-33  1  10.3%  52:429    10:362     ---------------------------------------------------MLVTSMKTLAGVRSTEPLTAAYRAALRSRTYATASLNKPAAPVEIVPVTTVDGARLRVHAYGPADGEVIVLIHGWSCCIEYWHPQINALADR-YRVICYDQRGHGESTLGRVAPCA------DTLADDLATVLDAtrGRRAVLVGHSMGGITLQAWAARYPEQVPDRAAAVVLANTASKDIRFETDLLPLLNKPLAVANRPLTVFGRGV-RMPMVLAETLLTAPVPLPGGPMSPLVTAVLKSRVMTRNATADQAAFALGIVRSCRPLTRGLHAA-------------------ALADMDLGDAAKNLTVPTTVIAGLHDHLLPERMNRTIVEALKsaDYQVWPTGHLSNVEAADRFNAE----LAKIAASAYRPQADA--------------------------------------------    
 467 UniRef50_Q1J3W1        3-oxoadipate enol-lactonase...   146     2e-33  1  12.6%  98:471     2:310     -------------------------------------------------------------------------------------------------TFLDTGSLVLHLQSIGSEAASPLVFLNPIGSDLRVWDEVAGELRS-SFRIIQYDLRGQGLSDVPGG------EYSLEHHV----------------------------------------------------ADLLGLLDALGLEQVALVGCSLGGVIAQALALSHPARVARLVLLDTLPRIGTAE-SWQTRIAQVSAEGLPALAPTLIRRWFAPAFFQE--QPTAAHGYTTLLARSPQDGYLGSCAALRDADLTAQLQTLRIPTLVLCGEHDISTPPDACRALADQI-GARFALigAAHLPMVERPHEVARLLRDFLGEKTATGR---YDQGMAVRRRVLGDAHVDRANAAVTDLDGDFQTFITEYAWGGPWS--    
 468 UniRef50_A8LAB3        Alpha/beta hydrolase fold n...   146     2e-33  1  10.6% 107:416    20:285     ----------------------------------------------------------------------------------------------------------VHYYEAGDPAGPPVVLLHGSgtGWSNFSGNIGVIADAGFYVLAPDMPGWGDSAAVATKEMDHD--------ADLVGFLDVLGLEKVALVGNSMGAHTAIRFATLHPGQITHLVTMGASLG-------------KGLPAGLFGPQDGPS----------EGLKVLVKAYRDPSPENMKALVEIMTFDKARFATPALTKARSE----------------AAAARPDHLRNYVEGLADGAPIPFKVD-RSLIPSIAVPTLLIHGRDDRVLHFETSLWLCANIRNSRLVLLncGHWAMIEHADEFNHLVINFLT---------------------------------------------------------    
 469 UniRef50_C3JU79        Epoxide hydrolase n=3 Tax=R...   146     2e-33  1  13.0% 100:400     9:262     ---------------------------------------------------------------------------------------------------IPVGDLTFDVLISGPDDGIPLVALHGFPESAASWGKVTPLLTAAGYRVIAPNQRGY----SAGARPEGVDAYKIEHLVADVVGLLDALGLSDAHLVGHDWGSAVAWQVAGRHPNRIRSLTAISVPHPTAFGWALREDEDQKQRSSYIGLLRQEGKAEEVLSEDNFRRLRAMF---------------------------------------DGQIDPEFADEHIRVLSGEGALTGALNWYRAMTRDFGDID------RTTVPTTYVWSTGDTALGRAGAERCGEFVDayEFVVLDGghWIP-------------------------------------------------------------------------    
 470 UniRef50_A4FCS3        Hydrolase n=4 Tax=Actinomyc...   146     2e-33  1  10.1%  83:417    19:309     ----------------------------------------------------------------------------------GAPGLPDGFTDTFSSRWVQVGDLHLHAVTGG--DGPPLLLLAGWPQTWYAWRELMPALA-RDFSIVAPDPRGVGLSGKP------DDGYDTGTLAADMVELMSALGHDRFAMVGHDVGMWTGYALAADHPERLERLAVAEAVIPGLAPSPPLFAPQESIDRLWHFGFNRLGSLNEKLVE----------------------------------GRERLFFGYQFATKAVRPLPEHAIDLYIETLadPDALRASFEFYRALDTTIEQNE-RRRQRKLTMPVLTIAGARS--VGELVGKTLAPVAEDVtiVLPDCGHFPAEEAPQDMLVVLNDFLAS--------------------------------------------------------    
 471 UniRef50_B4V9Q5        Short chain dehydrogenase n...   146     3e-33  1  12.1%  96:433    10:315     -----------------------------------------------------------------------------------------------RERRVSTGGVELCVVELGEADRPTVVLVHGYPDSKEVWSEVAERLATR-FHVVLYDVRGHGRSTAP---VPLRGGFTLEKLTDDFLAVADAVSPDrvHLVGHDWGSVQGWEFATTARTEGRIASFTSMSGPSLDH--------------------FGHWIKKRMARPTPRRAAQLL---NQGAKSWYVYMLHTPVLPELAWRGPLGKRWPKILQRIEKVPSGSYPT----ASLPSDAAHGAWLYRDNVRSRMRRPRADAYA--HVPVQLITPTGDAFLSERLYDDLELWAPDLLRRTLPAKHWVPRTRPDQLAAWITDFVTSREEPATRAPEQRAPGR----------------------------------------    
 472 UniRef50_A8LCY0        Alpha/beta hydrolase fold n...   145     3e-33  1  12.9%  98:422     2:278     -------------------------------------------------------------------------------------------------PTMLVNGIALHYDEYGDGADPLVVLVMGTGGKGQVWthQVPALVEAGYRVVTYDNRGI--SPVPGQTATSCPDIVVPELVDDLAALIERFG-APAHVIGTSLGARVVQELVLERPDLVCRAVAMGGHARIDDVGQMLTRGEQELFDQKIVLPKAYKAAVDALLNLSPATLR-----------------------------------------------DETKSNDWLALLEYSAVPASSGERGQLGASERLGDRGDAYRAITRPLLVIGFADDLMIPAHLGRELADIVPTARYvvPDTGHYGYLEQPEAVNRLILDFLAEPEGSA---------------------------------------------------    
 473 UniRef50_B8PH43        Predicted protein n=1 Tax=P...   145     3e-33  1   9.4%  99:417     9:322     --------------------------------------------------------------------------------------------------RTTTRGLKYNYFAagDAFKPTLLFVHGFPSTSHDWRLQVAFFKNAGYPIIVPDLLGYGSTDKPADSAL----YRYSAMCADIVEILDLEGARDVIAIGHDWGSILTSRLAVFHKERFLAFAFLAVGYSPSAADFdaLTKQALGYETFGYWRFFSEDGTDRVIKDHSESFLSIVH-------------AADPQIWKTDFCPSGALKTWLTADKKCAVGSYLNDKDKEIFKqdQGLGGALNWYRIMLLGIAAEdiAEPQMLIEQPVFFGEAKHDAvvPRLYLPQMQKLCTNLTVREYDCGHWVHLVLADDVNSALADWISG--------------------------------------------------------    
 474 UniRef50_Q1GWQ0        Alpha/beta hydrolase fold n...   145     3e-33  1  11.1%  51:419     1:311     --------------------------------------------------MKILLFLIVAPLIALALLYFLFPEQLVRMMRALMRK-----RARVVRRSITVDGRNWPYLEGGDPSKPTLVMVHGFGADKDHWIFYAPWMT-RDYHLIAPDLPGFGENDR-----DGALPFDVASQAARLKAFLDALGVDRPHLGGNSMGGWIALRFAIDYPDRLRTLTLMNNAGVVGENESELQKLAATRDYNPLVLANLDDAD---------------RLIAFVVRKPTYVPARLKPVIYADALRHRELLDRIFWI---------------------------------IADEMEQAPLNGELDKVTVPTLILWGRHDKLIDVSCVAVLEKGIANSRAHIFevAHVPMIEDPKATAEVQRAFLADHP------------------------------------------------------    
 475 UniRef50_Q1D6Z6        Hydrolase, alpha/beta fold ...   145     3e-33  1   9.4%  89:417     3:290     ----------------------------------------------------------------------------------------PGPRYFHQDRLRVPDGAQLYYQVQGDGL-PGMVFCDGLGCDGFAWKYLAPYLV-RNHRVLRWHYRGHGRSTVPEDRSRIGMLYT----CDDLRRMLDAVGMERVVLFGHSMGVQVALEFQRRYAQRVCGLVLVCGSYGNPLDTFHDSTALKRAFP-----------LIRRVVERYPEQSARIIHT--------------------VLRTELAVQLAITLEMNRERIARNDLAPYFDHLARMDPV-----VFVRTLESMAEHNAWDHLPHVDVPTLVVAGGQDKFTPGWISEEMADRIPGAELeiPEGTHTTPLEAPGRVEARVERFLRE--------------------------------------------------------    
 476 UniRef50_UPI0001C31AAE alpha/beta hydrolase fold p...   145     3e-33  1  12.0%  85:417    18:299     ------------------------------------------------------------------------------------RAAWMEIDWPAQTRSLTVDGGRANVVELGA--GPPMLFVHGLSGCWQNWLEQLPVFA-ASHRVIAVDLPGFGDSELPEREP------SIPGYARFLDRLCDRLGvePAVVVGNSMGGFVAAELAIAVPERVERLVLVSAAGISSDRVQRRPVL----------------------------TTARAIALVTAWGASRHEAFARRPGLRRVALSFVAR--------HPERMPAPLAFELMRGSGRPGFLPALDALL--------GYPLRERLPQVACPTLIVWGEDDRVIPVKDASRFERLIPGArvVLPDTGHVAMLERPVVFNGLLRSFVDE--------------------------------------------------------    
 477 UniRef50_C8RYP0        Magnesium chelatase accesso...   145     3e-33  1  13.5%  88:420     3:284     ---------------------------------------------------------------------------------------PADWPHRDASRFVVCKPHLWHVQEMGT--GPLLLLIHGAGGSTHSWRQLMPLLA-QHFRVVAMDLPGQGYS-----RSGARSRLGLDGMSTDLLHLCAHEGWQPDAIIGHSAGAAIALRLTELLPHKPRAVIGINAALSNFDGVAGWLFPLLA--------------RALTLAPFVPQIFARI--------------------------SGTPAKVNSLLAYTGSPLDTEGKALYQRLVSNPGHVDATL----AMMSQWKLDGLLARLPAMTIPLLLIAADQDGAVPCATSRNWAARMPNAEYVEIPggHLVHEEVPKAVAGLILSFLARHPA-----------------------------------------------------    
 478 UniRef50_B8MFR2        Proline iminopeptidase, put...   145     4e-33  1  11.4%  99:415     4:297     --------------------------------------------------------------------------------------------------YITINGARLAYEISGPETAPLMITLHGgmGDHRSDYKIYSQLNDR-LQVLSFDYRGHGQSSRTK-------PYTFEQLVDDIEGIRQqlGAeqVIICGGSFGGFLALHYAIKYASRVSRLIL---------------RGAAASHHRISAFSFQQHALLANLTTDEEDSIKSL-----EKRLHKAPNFSKEMLRDKVFGAYEDDEEFKLVYFAMMPLYKEVFDANAALKACRDTVCFAESHNELYSEKEKYFDYTKKLASITAKTLVIVGENDWICPPENSRIIAERIPGAelVVSDANHSVHIEKPETVLGRIKEFL----------------------------------------------------------    
 479 UniRef50_C0W573        Alpha/beta family hydrolase...   145     4e-33  1  14.8%  92:421     8:303     -------------------------------------------------------------------------------------------PGPWTHRDLSAGGTRFHAALAGpgTGPLVVLLHGFPECWWTWRHVMPALAREGHRVAALDLRGFGGSDRPPS------GYDLVSLADDVHGAIRALGHESAVVVGHGLGGHVAWVTANRFSSTVRGIVPVSAP--------------------------------------HPVAVRSLRGRLLSGAALQYL-SFKVPLLPERTLATQAGMERLLRSWAGPRTRaeAAGYYAALLARPGAAHSALETLRDML--LGRQTLAALEPAVQVPVMSVQGQVDPVQPAQAYARDTHHVAGrqVTIHGVGHFPQEEAPNQLTEVLLPFLADLVPA----------------------------------------------------    
 480 UniRef50_B0BYW6        Alpha/beta hydrolase fold n...   145     4e-33  1  12.8%  84:428     1:319     -----------------------------------------------------------------------------------MSTSRTWQQQIGHQRDWAWRGWQIRYtmRGQQeqPPMLLLHGFGASIGHWRFNIPVFAKDR-TIYALDLLGFGASEK------VSTDYLVTLWVEQVHDFWQTYIRTPMVLVGNSLGSLVSLTAAALYPEMVAGLAMLTLPDTSVLKNPSWVK----------------PAIAPLKLALNPVAAFAKALFTAPPIFNPFFQFIRQPKIIRSWVKK------------AYIDTTSVEDDLVDILSSPAYDQGAADALRAMVNTMSKPQVpeMLPQLTIPILLVWGQQDVMIPPKLGPLFARCNPRIQLVElhAGHCPHDECPDRLNPILLDWLAAHFPIQQPQPEQ---------------------------------------------    
 481 UniRef50_Q0CP98        Predicted protein n=1 Tax=A...   145     4e-33  1   9.7%  77:416    18:320     ----------------------------------------------------------------------------LLNSPPHWKKHNAWIKTTDD----DAPSVRISYIDCAPsaKGVLLLIHGFPQTSYQFRHVIRPLADEGFRVIAPDYRGAGQSSHP------SHDFRKSTMARDLFVLidHLHIepIYLVGHDIGGMIAHAYVSMYPQHVACIIWGECPLPGTSC-------YLANKRMPEQFHFMFHCAPDDLAPSLVAGREKMYLNHFFSRL----------------------------SFNADAISAADCEYYALQYSQPGALRCAFEVYRAFEEDAKENLESLRlgKCMRPVLILSGSESR--HAAEAKDMVTEVYGGDivVEDSGHYIAEENPAGFVQAVLAFVS---------------------------------------------------------    
 482 UniRef50_Q2SFX3        Predicted Hydrolase or acyl...   145     5e-33  1  11.1% 100:423    23:284     ---------------------------------------------------------------------------------------------------VNAGNINTAYLSAGA--GAPVICLHGAGAGAVTWYPSLGALS-QHYHVIAPDIVGYGESDKPDAP------YDRPYFSTWLQDFMAALDISKAHIVGLSQGGAIALQFALDCPDKVDKLVLVDTAALGAQPSLRPMVGMIWLNSFPSAWANRFFAPSLLFDTNNRD----------PNHAHYSIEVLKRPGGKNAFTQGRGAAVAALPEEALRRIHNETL--------------------------------------------IIWGEQDQLFAIEHGEAAAKLMPNAKLhiPRAGHLPLMDQPELFNQALLDFLTSTTPATA--------------------------------------------------    
 483 UniRef50_A1SD68        Alpha/beta hydrolase fold n...   145     5e-33  1  12.2%  82:417     2:289     ---------------------------------------------------------------------------------TAVAPAPVERSGIRESDVAIAGGRHLRVLEAGEPGMPALLFLHGSgtARANWENVMAGLGDR-FHCIAPDILGFGESSH-ADPQPQGFKANAEVRIDALLQMLDALGVSRVTVVGNSMGGMYSLRLCQLRPDLVEKMVLMGSGGMPGLAPTPQ-------------------------------------LIKLVTFFDDPSLEAMTALLTEFVHDKTAFGDRI-----------EDFAAGRMALVTRPEIETAHRAM--FGEGEMLSFSPAELARLDVPTLVVHGRQDVIVPIECSLYLAEHLPQADlvMNNCGHWTQVEQPDRFRTILRDFADG--------------------------------------------------------    
 484 UniRef50_Q2KZX4        3-oxoadipate enol-lactone h...   144     5e-33  1  11.6%  98:417    14:271     -------------------------------------------------------------------------------------------------PYADLSQVRLYYVVDGPADAPALVLSNSLGTNADMWARQVPALA-HHFRVVRYDTRGHGRSSVPDG------EYCFEQLAGDVVELLDHLGVARAHFCGLSMGGPTGLTLALNHPERIDRLVLCNTAARIGSAQGWSDR--------------IAAVEKQTLAAMAPAVVERWLTEGYRQ---------REPGLAQVLVDMLRRTPDAGYQANCAALRDADLRPRLAEIHTR--------------------------------TLVIASTHDLAATPADGQYLAAHIPGARYVELDTSHISNWEDPDSFTRHVLAFLTE--------------------------------------------------------    
 485 UniRef50_A9AX81        Alpha/beta hydrolase fold n...   144     6e-33  1  11.1% 102:415     6:264     -----------------------------------------------------------------------------------------------------VNGVRYYYEERGSGD-QVIFFGHGLMYHWRIFEPQMEYFAAKGFRCIAIDWRG----QGQTEGGGTLDDYSMYRLGADAYQLLSDLGIKRVHWVGVSMGGMIGLRLYPKHPELFLSFTLIDSSAGDAPELLDGY------------------------------RQMGDAYLAYGLIPPLQEGLDAVFYTPKMAERHPQVL------------------AYWHAYWTNADRESL---YKAIIPVIDRDDVTDTISQIAVPTLIVVGVEDMSTPPAKSEAMQQLIANAQLriDDASHMSILEQPEAITAAMAEFI----------------------------------------------------------    
 486 UniRef50_D0LEC3        Alpha/beta hydrolase fold p...   144     6e-33  1  11.7%  83:415     2:307     ----------------------------------------------------------------------------------APDPSSVRISGDWHHFDVRANGVRFHAVerGLTDRPLILLLHGFGEFWWSWRHQLVSLTDAGYRAVAVDLRGYGDSDKPP------RGYDGWTLAGDTNGLVRALGHTDATLVGHADGGLVCWATATLHPRVVNRIAVIASPHPRALRHSVVRESEQRSEFLGGFLRNQLPRLGERELTRN---------DGAFIADYFATRSSAAWQATDDYTETLARNRSAVQIPYVAHCSLE----YRRWAFRSQFRPDGFRFMELMNQ------------RLHIPVLALRGADDPFILDGVMAASERWAAHLKYriEGSAHFAHQEQPDAVTEQLGKLL----------------------------------------------------------    
 487 UniRef50_A8ZZZ3        Alpha/beta hydrolase fold n...   144     6e-33  1  11.2%  99:420    22:297     --------------------------------------------------------------------------------------------------VFESNNASIYYEDTGA--GAPVIAVHGLIENTAYWsgVTARLAG-HYRVIAMDMRGHGRT----VTTGDNPGYDADTVAGDIEALADYLGLDRFYLLTHSTGGFAGARWAMEHSDRLAGLVLTDTTSATCPFPGTPEERVIFFEKFAASFERQTWEEVIAYAKRKP-----------------------FPFFRGIAEHPDNGAMWDMALEIIRRGDRNRIAAFVRSFYQ------------------DPDMKVEGLRQIRCPVLVLVGEKDDLF-IEPGRVMAEAIPTCRHVVLEggHMTAIEAPDRLARELLDFLAACPG-----------------------------------------------------    
 488 UniRef50_D0VYM7        Putative uncharacterized pr...   144     6e-33  1  13.0%  94:417     4:293     ---------------------------------------------------------------------------------------------PVSRRIALSTGLRYHVLEWgpALDHPVVLVHGFLDLAWTWEAVAQSDLAQHFHLIAPDLRGHGDSDR----VGPGGYYHFMDYLADLEDVIAQLGRSRVSIVGHSMGGLVAAYYTGVYPSRVSKLALLEGLGPPDSDWSAWPDRLSAWIAAWKRQRERGGSKRYLSIE---EAAARLRATDPKLSVPMSQRLAEVSTVRTDDGRLIFKHDPLXLTPAPYLFMVDGARSLWQ--------------------------------RITCPTLLVDGKDTEfySPEEAQRRQSVFPNARHvlPDAGHMMQRHQPEQLAAILDEFLRT--------------------------------------------------------    
 489 UniRef50_B3EK63        Alpha/beta hydrolase fold n...   144     6e-33  1  12.1%  97:418    44:317     ------------------------------------------------------------------------------------------------SRFVKINGVVHHYHVSGPQDaeTVLLVHGWDCWWMWWHRVIRELNEKGVRTIAYDLKGHGWSD-----PDPGQDYSISSFSHDLAEMVRQLEVKEFHIAAFSFGPFVVLDYAQKTTDRVKSIVVYNFGYLPNNAFLERLAPSV------------------------------LTLTFNTVLRKIHWWRLIYVYARVVLARNPVSFHDIMVGQKSLELCCPEV---------------VQKTTKQITSREVTRSLPDIVNSIDIPVLFVAGAGDTIMSSHNTRKLSGYAKNGSYvvPKCGHLITLELPGTASDLIISHMKSV-------------------------------------------------------    
 490 UniRef50_A5VE39        Alpha/beta hydrolase fold n...   144     6e-33  1  12.2%  94:422     1:270     ---------------------------------------------------------------------------------------------MTVRSAVVAGTPRLAFSFAG--EGELVVMLHGVGGNRRNWLSQIEALSPACL-AVAWDARGYGDSD------DYAGELSFGDVADDLLRLLDHFDRRRAHLVGLSMGGNIAMEFALRHPDRVASLVLADT--------------------DRGMQHIPASEREAFLALRREPILAGVALDELAV----------------------PIVASLLGEGAVPTARAEMIDSISRLHRDS---------YLKALKATVDFDVVGKLDRIGVPTLVIVGEEDRLTPVEEARAICAEIAGSElvIPGAGHVSNVEQPERFSQALLRFLRSVGAAP---------------------------------------------------    
 491 UniRef50_B5J801        Hydrolase, alpha/beta fold ...   144     7e-33  1   9.2%  98:412     9:281     -------------------------------------------------------------------------------------------------HKADIKDIKMHYTVAG--EGPAIVLIHGWPHTNYGWRKVQAKLAE-NYTVIAPDIRGAGYTSRPET------GYDGDNIADDIRTLvdHLGFDKVVVVGHDWGSVWAYHYAAQFRDEVVGLGNFEMMIPGTGGHEAGMQPKPDGKFFWHMNWHSVPTYPEELIRGNERKYLDYYYTEFS--------------------------------YNPYSVTIEALNEYERAFTQFGSLRSGLEIYKKFWEHAAQAEKH-MQTKLEMPVIAWGGAadWCV--ESMQHVATDVSGGG--IPECGHWVAEEQPDFVANEIR-------------------------------------------------------------    
 492 UniRef50_Q67RA1        3-oxoadipate enol-lactone h...   144     8e-33  1   7.5% 100:418     4:264     ---------------------------------------------------------------------------------------------------IRANGITLHYQWRGQEDGPVLVLINGLLTDLSSWNGHLPHFTP-YFRVLTYDCRGQGASEKPDDGP-----YTPRLHADDLKGLLDGLGVEKAALLGVSSGACVALSFAGRWPERVSALVLANGYGAADTAMQVKLNSWLRAM----------------EAGGGPLRFDVSVAWIWGATFLNRHYEALRPWREKGTALPAHAVLHLIRGAMDH-------------------------------------DALVQAANITCPTLLMTGDEDVLTPLRYSRALEQRIPGSRVVvpEAGHCMFLEQVDLFSRTALDFLRQA-------------------------------------------------------    
 493 UniRef50_A9FPA8        Putative secreted hydrolase...   144     8e-33  1  11.0%  88:415    41:322     ---------------------------------------------------------------------------------------ASAAPRIRSGTFTTSDGVALNYLEAGR--GRPLLLLHGWSQSAALYRAQILAFS-RHHHVFALDWRGHGASEKPE------HGYRIARFSRDLHEFICGMRFKHLSVLGHSMGCGALYSYWDNYGGrlDRLVLVDVGP----AMTAWPDWSAEEAAEAGALFTPQSLHETAAALRGPDGIA--------------------------------TTYGLVATMFTPGYPADQLALVVEQ-----NLKLPRAYAAKLLVDQAEQDWRDVIPRIDVPTLVVGGEASIFNPISQ-QWVADQIPGAELIleGGSHFMWLENPEKFNEDVLDFL----------------------------------------------------------    
 494 UniRef50_C6CWT3        Alpha/beta hydrolase fold p...   144     8e-33  1  10.8%  90:413     9:285     -----------------------------------------------------------------------------------------PMEYPFTDRWLPYRDGYIHYLDEG--QGPTVLLLHGNPTWSYLYRNVIKELRED-FRLVAPDYPGFGMSKAPTG-----YRFTPQEQSAAIQDLINHLDLKDMIVVVQDWGGPIGLNYAVRHRNNLRGIVVMNTWAWPAE-----------IMPMKLFSLAMGGWPIGRWLQTRRNFFAKIIVPHGIHHAE--------------------------------KVTDSLRKAYTDPFPTPKSRIPTWVFPRQIraRKWLTDIESKLSNldLPAQILWGTKDSafPIEQMAKWQRYLpnETEILEDASHYVQEDRPDRVVAAIRR------------------------------------------------------------    
 495 UniRef50_Q0VNU3        Hydrolase, alpha/beta fold ...   144     9e-33  1  12.7%  79:417    28:339     ------------------------------------------------------------------------------AEPVALDAQLSEYAYppVQRLTVADqlEMAYMDVQpaNGETVLLLHGKNFSGAYWKSTMDALLEQGYRVVVPDQIGFGKSSKPD-----RFQYSFQVLADQTRRLLDSLEIERASVVGHSMGGMLATRFALMFAQRTDSLTLVNPIG-------------LEDWKQKQVPWQSVDAWYEGELKKTPEKVKAYMTRSYFDGQWKADYEPLLALQQGWIRGPDYPLIAWNSALTYDMIYSQPVVHEFDRLAVPTLLIIGNRDRTALGSNRAPEAMQDKLG--------LYG--------ALGRQAAQSIPEASLVELDggHVPQYEAFEDYIKALTTFLAS--------------------------------------------------------    
 496 UniRef50_Q3AZ37        Alpha/beta hydrolase superf...   144     1e-32  1  12.3%  62:427     4:340     -------------------------------------------------------------AVSANALQPAEPWCPEAETSAWGHHHEWTWRRQPSRRNQN-QQWTCHWRVLGPSHGPALVLLHGFGASSGHWRRIAPKLAAQGWQVFSLDLLGFGASEQSGIRQGGPLDNRI--WGQQTAAFLQEIVQRPAVLVGNSLGGLSALTTAVLTPDLVRALVAAPLPDPALLQPVPRRRSPWRRRWQRGWLSLIVQLIPLQWIVPVIARSKLIRLGLQGAYTCSITNDLDLQQLICRPARRPTAARALRAMTLGMGLRP---------------------------SGATAPALLEQLATTQLPMLMLWGRNDRFVPLSVGQKVVDQHPWVELKVLncGHCSHDEDPNQFLNALLPWLDRNLGNSRPAGD----------------------------------------------    
 497 UniRef50_Q6CAZ1        YALI0C23224p n=1 Tax=Yarrow...   144     1e-32  1   8.5%  93:421     9:345     --------------------------------------------------------------------------------------------YEPFHRNAVLNGKRWHYVdsGsgRGKTLLLVHGFPDTWYGWRHQVPVFRKHGFRLLIPSLLGFPRSEAPLTHPGVATgyTADFFAKSMVQLLDQLGIDKVCSFGHDWGAVFAPRLWLNYPERVECVSSACWYYQMPMEGFVDLKDFAEVAPSLRYQLYWGGDAPQEVIQK--------------PLKEFMDRMYHHPTENHLLTSEAEYNNILKEFQYGGKTIAPMFPLYKSRKLAYDIDE------RDFLTKGRTEEDLA----VDVPYLFIGAEFDIALQPGMESVLEGYVKEGLLewVPCGHWVLFEEPEKMNKIYIDFLKKVFGS----------------------------------------------------    
 498 UniRef50_P64304        Haloalkane dehalogenase 2 n...   143     1e-32  1  11.3%  88:413     7:283     ---------------------------------------------------------------------------------------PDPQLYPFESRWFDSSRGRIHYVDEGT--GPPILLCHGNPTWSFLYRDIIVALRDR-FRCVAPDYLGFGLSERPSG-----FGYQIDEHARVIGEFVDHLGLDRYLSMGQDWGGPISMAVAVERADRVRGVVLGNTWFWP----------------------------ADTLAMKAFSRVMSS------------------PPVQYAILRRNFFVERLIPAGTEHRPSSAVMAHYRAVQPNAAARRGVAEMPKQILA--ARPLLARLAREVPapTLLIWGMKDVAFRPKtiPRLSATFPDHVLvlPNAKHFIQEDAPDRIAAAIIE------------------------------------------------------------    
 499 UniRef50_B1MF26        Putative hydrolase, alpha/b...   143     1e-32  1  11.6% 105:422    12:301     --------------------------------------------------------------------------------------------------------LDVEVRRHGNpdKLPVVLLHGWPDSVATWTHVIPALASAGHQVYAPSVRGTGQTKFRYADTPRTG--RLGDLLRDLFAVCDQLKLERFHLVGHDWGARTAFACGIVAPHRIASLSAISAGWHPAGHGP--------------------------------------ALSSLQAQRFWYQWLLNTPAGADLLRRERRELVELIWRDWSpkFSDQEFDDTFTSLQSSDWLEVTLSYYRARWgdDPFEKQIAQATAISVPSLVLHGDSDNCVLPIASEGMNQHFPhsRLTTPGVGHFPQREDPLWVSGALLQWFKEWGNAR---------------------------------------------------    
 500 UniRef50_B5IY25        Hydrolase, alpha/beta fold ...   143     1e-32  1  13.9% 100:416    12:286     ---------------------------------------------------------------------------------------------------VDVAGAAIHARIGGS--GPPLLLLHGYPQTHVMWHVIVDELA-KSHSVVMADLRGYGDS------VAYDGDFTFRAMARDQVDLMKHLGFARFDAVAHDRGARSAHRMVLDYPEAVRSIALLDI--LPTLDVWRTMDDWLAKRYYHWMFLSQPGYMPQTLINSNPVMFLHAALLGLS--------------------------------GKKDAFDPLALAEYERAAKNPDVVAAWCGDYFEGAGTDIDHDRADMGRTNDIPCLVLWGSQGVVahVDPLMAWQAWFPKAEGHAIDAGHFLVEEKPTEVLRSLEKHLS---------------------------------------------------------    
 501 UniRef50_A9N3R7        Putative uncharacterized pr...   143     1e-32  1   8.5%  90:417    43:311     -----------------------------------------------------------------------------------------SATTMDKDKFVEVNGIKIHYVEEG--EGPPLLLIHGGGLTAKSWQGLAKE-ASRYFRVIMPDSRGHGLTNNPQGT------FSYDLMAEDTAAFVKALKLEKPLVMGYSDGGMVVLKLTSRYPDLARAAIVGG-----------------ATHRFATAHYMQGMEIFYGKGMPQGQLTDRDLDKMASDAPGMVKFYQNMHHPEQKDYWRTFLEGVWPMWTTPTSLTEEEV------------------------------------KKIHIPVLLLDGDRDEFFTVEEVTELYRLLPQAEMtiPGSGHAIFQtkTPLFYALVLEFLQR--------------------------------------------------------    
 502 UniRef50_B0C147        Alpha/beta hydrolase fold n...   143     1e-32  1  12.2% 100:418     4:283     ---------------------------------------------------------------------------------------------------ALIRGHD-HYYEWVTQdkPVMVFLHGWGGSSRYWRPIAQALAAD-FDCLLYDLRGFGQSQDSLRASRDVETYTIQSYVEDLEVLLDALEISQVFLQAHSMGSTIGALFLNQYPQRVQQAILACSGLFEYDEEEFRQFHQVGEWVVRLR--------------------------------------------PRWLAQVPLLDRLFMARFLHRPIAADQRRGFVSDYVGADAVAALGTLLSAVSESVATA-MSAEFAQLQTPTLIVSGDKDQIVPADSGEEAAALNPNIDYvmAQTGHFPMLEDTSTYLQHIHHFLTPV-------------------------------------------------------    
 503 UniRef50_Q39XD9        Alpha/beta hydrolase fold n...   143     1e-32  1  12.1% 105:418    10:268     --------------------------------------------------------------------------------------------------------VTIHYEDEGS--GFPLVFVHGWAMSGKVWAFQKPL--ADHFRLITLDLRGHGKSSAAPG-------YAFSDFAADLAALFDRLGLERAALVGWSLGAQVALEAVPLLEDRVAALVLVAGTPKFTTADGW-----------------------------------------PHGLPPHEARGLGLRLKRAYDATLGDFFRQMFAEG---ELSHDQYQRIVREIVIPRHLPDP-GAVQACLVTLAGGDHRNLLPAIAAPTLVIHGDRDAICSPSAGRYLAEGIPGARFllEGTGHAPFLSQPERFNQELSRFLTEV-------------------------------------------------------    
 504 UniRef50_B1M8R1        Alpha/beta hydrolase fold n...   143     1e-32  1  10.8%  83:426     7:390     ----------------------------------------------------------------------------------PPLDPAALPAGIRARFVEGVNGLRMHVLEAGepDRPLILLVHGFPEIAYSWRKVMPALAAAGYHVVAPDLRGYGRtdAPVAFADDLAPYRLHNHLLDLLCLMSALTRDraALVGHDYGSWVCGYCALARPDLFGTVALMSAPfrTPADDPIHAALAALPRPRTHYHRYYASRRAAADMERSPQGLAAFLRAYFHHKSADWPDNRPRALagRTMPATVAPEMPPRDGADCAWLTDAELAVYAAEYARSGFQGALQGYRCRMDGSIDRDLARFAGRRIEVPLLFVSGARdpFQVPGALGRMEEAAGAGLlfVAGAGHWVQQERPQAVVDLLLDALARGGAGHRTGA-----------------------------------------------    
 505 UniRef50_C9ZB30        Putative hydrolase n=2 Tax=...   143     2e-32  1  11.3% 112:421    19:279     ---------------------------------------------------------------------------------------------------------------AGPADGRPVLLLHGFPRTAVLWEEQVAALGDQGFRAVAPDTRGY----SPGVRPMRADAYGVSELVGDVLAVADALAWNRFDLVGHDWGGAIAWWTAARHAERLRTLTTVSTPHPNALGEAHRTDDDQRRRSQYQQDWRSPDTEARFLADD----------------------------------------AALLRSLYQGKVADHHVDSYVQRLSEPGALSAALNYYRA-------DRPDAAVGKIQVPTLYVWGTEDVALGSTAAHATEKWvpYSFHALQGISHWVPDEAPEVLTSLIIDHLNEHAPA----------------------------------------------------    
 506 UniRef50_A4TD98        Alpha/beta hydrolase fold n...   143     2e-32  1  11.8%  83:455    12:351     ----------------------------------------------------------------------------------AGSGPAVELPRGRVVDVRSRDGVRLHAEVFGPEDGYPIVLAHGITCAIPVWAHQIADLS-RDHRVIAYDHRGHGRSGVPPRR----GGYSLDYLASDLDAVLEaaPGERAVIAGHSMGGIAISSWSERLPHRVAERADGVALINTTTGDLLRNVNLLPVP-----------------ARFADARVRAAGGVIRTFGGTSMVRVADLPS------------RRFVSMIAVGRDADPAIADFVFELFHRTPPAGRGGWARVLVDHLGPRHIG--LDNLTVPALVIGSTHDRLLPVSSARHIASAAPnaRFVELAGGHCAILERPEEVNQQLRWLVDSVSRSTRASAATGDQARASEATGDQARASAATGDQARAS------------------    
 507 UniRef50_A4ETX5        Haloalkane dehalogenase n=1...   143     2e-32  1  14.4% 107:418     1:274     ----------------------------------------------------------------------------------------------------------MAYIDEGEGD--PIVFLHGNPTSSYLWRNVMPHLAGMG-RLIAPDLIGMGDSEKLPD--SGPACYTYVEHRKYLFALLEQLGveNVTLVIHDWGSGLGFHWAHMHPEAVKGIAFMEGIVAPVPGWD-----------------------------------------SFPEAPREIFQALRSPAGEEMVLEKNLFVEAILPGSILRKLSEEEMQEYRRPFANAgdRRPTLTWPRQIPIGGDPADVtaWLRETEIPKLFVNAEPGALI-AGPVRDYVRAFPNLSEVTVKGSHFIQEDSPDEIGAAIRDWIKAL-------------------------------------------------------    
 508 UniRef50_B1Z5J5        Alpha/beta hydrolase fold n...   143     2e-32  1  12.1%  97:417     2:273     ------------------------------------------------------------------------------------------------STFTTRDGVSIHYKDWGA--GRPVVFIHGWPLNADMWDVQMHHLASNGFRAIAYDRRGFGRSGQP------WTGYDYDTLADDLATLIETLGLRDVTLVGFSMGGGEVARYIGRHGTRHIAKAVLIGSVTPLIARRDDHPDGVDVAMFDGIRS-------------------------------------------AIVADRPAFFEQFWPLFTGSNRADSTISRATLDWTSFMALQAGLKGTLDCVRAFSETDFRSDLDKFDVPTRVIHGDDDqaPLALTAAATAARVPHATlvYEGAPHALYLTHAQRLNDELLAFVSA--------------------------------------------------------    
 509 UniRef50_Q0RZ78        Probable hydrolase n=1 Tax=...   143     2e-32  1  11.0%  83:426    98:400     ----------------------------------------------------------------------------------GVLAPAADLPTRARVRETSVFGVRTGYLDAGPPGGPPVVLLHGLGATNASMLPVLADLAAE-HRVLAPDLPGFGSSAAP------NWDYDPVQLQRWLRAFLDTVDAPASAVIGHSLGGRVALELALRNPDTVRSLVLLCPALAFRRRQLT------------------------ALARRIPADLARLPIAFPHRLLHEGTRGACRTLFADPDR-------------VPRHWFDAAADEWELTLRDTGHRRALWSATLalDEPFGDRGLWNRVAQLAVPTLCVWGADDPLVPARFADHLTATAPQVrvTLPDCGHLPQFEWPEATGALIDDFLTRTARHPRTAA-----------------------------------------------    
 510 UniRef50_A1UC19        Alpha/beta hydrolase fold n...   142     2e-32  1  12.5%  63:422    26:353     --------------------------------------------------------------SLAGVTVARSVARRVTDDDPYSAEDFELLDADRSSVVTTTDGVPLAVREVGPADAptVVFAHGFCLRMGAFHFQRARLSedQVRMVFYDQRGHGQS-----GEAPPDTYTVTQLGQDLEAVLAVVAPrpVVLVGHSMGGMTVLSHARQYPQRYPTRVVGAAIISSAAEGVSR--------------------SPLGEILKNPALEAVRFAARYAPGTVHRTRGVARAVIGPILRAASYGDEKISPS--------------VVAFSEKMMHDTPIPTLVEFLHALEVHDETGGLTTlkVPTLIACGDRDLLTPMEYSQEMAAALPKSELVIVdaGHLVQLEHPEQIDDALVRLVERATPSR---------------------------------------------------    
 511 UniRef50_A1WW50        Alpha/beta hydrolase fold n...   142     2e-32  1  15.8%  97:419    21:307     ------------------------------------------------------------------------------------------------SRFITvdGLQLHYVTAgdSDKPAWLLLHGFSFSTVTWEPLLPSLGSDRYTV-AYDQIPYGLSDKPDYRGEGPNPFTLEADVAHLFSLMDELGQEQAVLVGNSAGGVIALEAARQAPERVAGLVLINPMAALERPTLPKWLAQL------------------------------------------PQAKRLSLLGGRWLGRSTELLER--SYYDTDAITPEREARFSLHTAMAGWDRAWGQLMHRSLTDALQ--VRGPLEGVETPTQVIISVEDEVIPAADSHRVADALPNAERVElaCGHLPQEECPAETAAAIEQWRERHD------------------------------------------------------    
 512 UniRef50_C6WRS4        Alpha/beta hydrolase fold p...   142     2e-32  1  10.7%  69:415    27:333     --------------------------------------------------------------------PAGPEAAPEIPAHEVITREDPEFAGRFRHEFADVDGVRMHYVTGGS--GSPVVLLHGWPQTWYGWWEVMPELAE-HHTVYAIDLPGLGDST---GTPSGYDKATLARYAHTLVA--DRLGVRDAAVVGHDLGAAVAFQYAAQFPQDTARLGYLDLPLPGPAIGETAYRSLSWHIAFHSQPQVPEAVVGDDV----------------------------REYLSLFYPQVSFNGTAFGGDATTSPFSEAEIDEYARTYREPGALSGGFELYRSLGADVRAAESAPPL---DVPTLVLAagQSDAIRATVAPRLTNVVRSAD--VPNAGHWLVEENPEFVTAELLAFL----------------------------------------------------------    
 513 UniRef50_A1C881        Epoxide hydrolase, putative...   142     2e-32  1  11.4%  74:414     6:386     -------------------------------------------------------------------------DLPSIPLPPGVSSHYLKSP---------ANDLTYHFLSAGNQGDPLILLYHGFPETSFCWRHILPYLAQAGYYAVAPDTRGSGRTTTptESADELRQYQLTNTARDTVIFIQALGYSTVhlVGHDAGAVLVAWVALTRPDLIQRVIIASHPYNGPPadIHAALLKLPHPRKHYKWYYAGPAAAADLQGASVDELRalCAWTAAELAQMPYYYVMPADSGMAAAVQRQLQRSGHEPDCSPWLTDAELEFLAHEWNRTGF-QGALNWYraTNPAWQRDLQLYAGKKLACPSLSILGRQdpGVLEAVGSKCLDY--RGEVWIEEAGHWMQQEQPEGVARHILKF-----------------------------------------------------------    
 514 UniRef50_Q13KN4        Putative lactone hydrolase ...   142     2e-32  1  10.2%  98:419     3:262     -------------------------------------------------------------------------------------------------RRADLNGIQTHYRLTGKAGAPTVILSHGLAADLSMWAPQLDMLS-RSFSVLCYDIRGHGGTSATPG------DYSLALLADDVIALMDSLGIASAHYVGLSLGGMIGQQLGAWHGERLASLTLCATTSNAPKASWDARVHE---------------ARARGIAPLVEATVDRWVTPAFKREQPELMEQMRRMVLGTSL----------------------------------DGYAGSAAAIRDMELAP-------VLGRIGVPTLVIAGEADTSTPLPILEHIADSIPAAtlTVPEAAHMPTMERPELCNPALERFLLAHS------------------------------------------------------    
 515 UniRef50_D2PUR6        Alpha/beta hydrolase fold p...   142     2e-32  1  13.6%  92:423    12:306     -------------------------------------------------------------------------------------------DGPWSHSLVAANGARFHVAECGTADAPLVLFLHGFPEFWWAWRHQLPVVAAAGYRAVAMDLRGYGASDKTP------RGYDPFTVSADVSGVIRSLGASDAVVVGHGWGGFVAWSAAVLAPRQVRAVAAVAAPHPLMLLTSGQLRAAVQMGWFQL-----------------PILPERRLLAHDGIHIEQLLRAWSAAGGDFPDAEASRRYRAALQVWPAPHCALEYHRWFVRSRLRSDGRRYSTRMRE----------------PIGVPVLQIHGGLDGAVAPAASVTPAQYVTglRYELLTtgHFPHEEAPEQFNQLLLDWLPGVSPTTP--------------------------------------------------    
 516 UniRef50_Q1NCY2        Putative uncharacterized pr...   142     2e-32  1  12.5%  99:419     3:267     --------------------------------------------------------------------------------------------------TINANGIQLHYEIEG--EGPPLLLISGLGGHSGVWATVKPLLTP-HYKVITFDNRGTGQSDVPPGP------YTIDQMGDDAAALIEGLGLGPVHVVGWSLGGSVLQSLLIRHGRLLRRAVLLSAFPSYTAVQDGWLDCL-------------------------------LSLKRSDVSPIAQAIIGMAWGFTPRFLFDHDFVEAAARVGVEQDPTP-----------------VTTEGFEAQAHGLRRYDSRVDLPTVATPTLVLIGAEDTLTPPAQSVEMAALIPGAKLQipRGGHGMVLEYTQDTVGAILAFLGDAA------------------------------------------------------    
 517 UniRef50_Q2JFD3        Alpha/beta hydrolase fold n...   142     2e-32  1  12.0%  96:452    61:385     -----------------------------------------------------------------------------------------------VTTVIAGDGVPLYIEETGPSDAptLVFVHGFCVTADSWILQQRALTDLG-RMVFYDQRAHGRS-----GPSEVKNCTIDALADDLFRVieRVPRGPIILVGHSMGGMTVLGLADTHPE---------------------------LFDDRIVGVALLATSAGELARLTFGLPASVTGVVRRVLPGFAVGMRHAPSLLERARRRGSDLSWELTRRIGFGST-ELPPSVVSFLETMVADTPIPVIAAFLPTLLDHDRLAAAERLAEIPTVLIVGDVDLMTPLAHSRTLAEALPRAElvEEGAGHAIMLERPDVVNAAIRE-IARQATAPRAMPRPRSGSAQTGRSAPAQTGRSAPAQTG---------------------    
 518 UniRef50_C6CXW3        Alpha/beta hydrolase fold p...   142     2e-32  1  10.9%  98:419     4:276     -------------------------------------------------------------------------------------------------HKVNVNGIDMVYEECGQENEKAIILLHGFCGSSHYWHNVCPLLTD-HYRVIMPQLRGHGETSVPDNV------YTMEVMADDINALIEKLGIDKTVVFGHSLGGYVTLALADKYPDKLAGFGLIHST-----------------------------------ALPDTEEVKRKRYQDISFIQKEGIEPYVHDLIPKLFKE------------SKRQDMPDEVEKVKEA----GLKMSAKGAIHTLEGMMMRPDRSAMLHEACYPILLVAGAEDEVVSPDDTFSvdKETPKSTYEYpgVAHMSLVEAPEQLARVIANYLRTLS------------------------------------------------------    
 519 UniRef50_A1KPZ9        Possible epoxide hydrolase ...   142     2e-32  1  14.5%  82:418     2:323     ---------------------------------------------------------------------------------AAPDPSMTRIAGPWRHLDVHANGIRFHVVEagQPEGplVILLHGFGSFWWSWRHQLCGLTGA--RVVAVDLRGYGGSDKPP------RGYDGWTLAGDTAGLIRALGHPSATLVGHADGGLACWTTALLHSRLVRAIALISSPH--------------------------PAALRRSTLTRRDQRHALLPTLLRYQLPIWPERLLTRNNAAEIERLVRA---RGCAKWLASEDFSQAIDHLRQAIQIPAAAHCALEyvRSQLRSEGRRFIRAMTQQLGMPLLHLRGDADPYVLADPVERTQRYAPHGRYiiAGAGHFSHEEAPEEVNRHLMRFLEQV-------------------------------------------------------    
 520 UniRef50_B3QSS4        Alpha/beta hydrolase fold n...   142     3e-32  1  12.2%  94:413    41:312     ---------------------------------------------------------------------------------------------IEQSCFIELNGTVHHYHDSGPRDAkvVVLIHGWDCWWMWWHKVIGVLNEQGIRTIAYDLKGHGWSD-----DDHVEDYSLSSYSKDLRCFVSALGLTRYHIAAFSLGPFIALDYAMQYEQDIATMTFFNFGYFPNSPFLSKFIPIF------------------------------LPLVFDKVLRKIKWWPPLYLYARITLARNPATREDIIIGVDSLKFISSE---------------AIRQTAEQISQIEVTENLPTQVAQIKTPILFVAGKGDQVVMWKNTQKLYEFAANGRFVVikCGHLITIELPQRTAELIAE------------------------------------------------------------    
 521 UniRef50_C6CVI2        Alpha/beta hydrolase fold p...   142     3e-32  1   9.3%  97:418     9:274     ------------------------------------------------------------------------------------------------HTITLPSGISLAYYDSKTVQQPVVVLLHGYCGSSAYWEQVVEPLA-RHARIIALDLRGHGRSS---GETGPEETNAMELYADDLSAMLDQLKINKACVLGHSLGGYITLAFAERFEEKLSAFGLVHSTPLPDSDAAKENRdaVAAIKKDGVGAFVEGLVPKLYSPDnpDQVERSIAIGRGTSAS-----------------------------------------------------GAIGAAKGMKERPDRTEV---LKQTSLPVLLLAGEQDQIIPVERTF----VIDGarEKLAEAGHMGMVERPEAFAEAVLRFINKL-------------------------------------------------------    
 522 UniRef50_A0LBW2        Alpha/beta hydrolase fold n...   142     3e-32  1   7.3% 107:421    13:282     ----------------------------------------------------------------------------------------------------------LHYRVTGEADAPPWVFLHGLMGAGQNWRRIVRGM-QQGRQILTYDQRGHGRSAKPA------QGYALEDYANDLLMLVDALGWSRFVLVGHSLGGRVALCFAHAYPQRLRGLVIVDIGPGGQNQIADRSAKLLEMIPTPFADK-----------AAAKHYFAHTFAQQAHAAGWGAVARLGAFLYSNLHVSEQGQVDW--------------------RFYKPG--------MLASVAAPAQQPRWDQLHGLSMPTLLMRGEHSDYLSPETFRQMVACNAHIqvQIPQAGHWVHADQPEAFMAQLHHFEQRLSGG----------------------------------------------------    
 523 UniRef50_Q7PV09        AGAP011970-PA (Fragment) n=...   142     3e-32  1  11.8%  52:415    19:337     ---------------------------------------------------ALVCVAFFLRLIRWLQVSYWVPNAR------PHPPDTLNHSKWGTHRYITVHNIKLHYVEQGSSSKPLMLFLHGLPDFWYSWRYQMHEFSKDYWTV-ALDLPGFGRSEPPAHSVT----YKLSNLARLVCSLITALGKSECVLVGNGAGSIIGWHIVNQYPDRVSRYVMLGMSSEAILQQLYQRGAIPLATLLKSAFLLYAGELPVLLARTD--------------------------------------DYAMFNKLLGPDAKPQDLEAYKYTFAQPAALGHALTAFRENFADFFLEEYEYRVRkaNIPGLFLFQEEDCftPEEYMGLLTDAYEPLETrvARVGRFMHQDDPKTVNKLIGEFL----------------------------------------------------------    
 524 UniRef50_B4UBY3        Alpha/beta hydrolase fold n...   142     3e-32  1  10.8% 101:415    20:298     ----------------------------------------------------------------------------------------------------QANGLRFAALADGPGDGPLALLLHGFPELARSWRHQLPALAAAGWRAVAPDLRGYGGTEK-------RGPFDLATLAADAAGLVRALGRERAVVIGHDWGGAVAWAVAGRHPEVVSRLVVLNAPHPSAFARELRRNRRQLLRSSYMLLFQVPLLPEWLLTRDRAAGVARALRGGSYVRSAWP---------------------------------AEELEPYRRAFLQPGAARAALGYYRAAFRGRRSPGPRPR--PISAPTLILWGVEDRFLGVETIAR-AKLAPYLsvPIEGAGHFVQNEAPEQVNAALLRWL----------------------------------------------------------    
 525 UniRef50_A6GE89        Alpha/beta hydrolase n=1 Ta...   142     3e-32  1  11.6%  92:419    43:330     -------------------------------------------------------------------------------------------AFPFERHELAVLDSTMTYVDEG--EGPPIVLLHGQPASAYLWRNVIPHLSEDR-RVIAVDLIGFGGSGKP------DIDYRIVDHAAYLEAFIDALALdpLTLVVHDWGSFLGFDYAMQHQEQIHAIVFMEAMLQPIPGYDFWDPDT----------------------------------------AAFMEAIRTPGVGEAMIFEQNMFIEALLPAMVMRELSPAEHDAYREPFLDPDTRAPMLSFPRELPVGGEPADVhdALRDSNVPKLHLYGTPGVLNTAADVEWAQANLPNITSVEVGPghFLQEDHPHAIGEAIVDWLDGID------------------------------------------------------    
 526 UniRef50_Q2T1T3        Epoxide hydrolase-related p...   142     3e-32  1   9.4%  72:421     2:316     -----------------------------------------------------------------------TRRSRSYNNVTTIRENKTMSFEEFAPFRVTAQDVDIFGVKGGA--GPPLLLLHGHPQTHMIWHRVAATLA-RHFTVIATDLRGYGASGKPPSDA-RHAPYSKRTMAADQVAVMRHFGFARFYVCAHDRGARVAHRMALDHPDAVERMLLLDIA--PTLAMYERTDRAFATAYFHWFFLIQPEPLPETLIGGHSDAYVDAVMGN--------------------------------RSAGLAPFAPEALHAYREALRQPGAVHAMCEDYRASATIDLDHDRAdrGHKLACPLRVLWGEHGVVArfAPLDEWRRVARDVSGRALACGHYIPEEAPVALIDEIVSFFEAREAA----------------------------------------------------    
 527 UniRef50_Q3IVD1        3-oxoadipate enol-lactonase...   142     3e-32  1  10.1% 105:420     8:266     --------------------------------------------------------------------------------------------------------HWIYYDLIGAPDAPVVCMSHSLTSDHGMWAEQVPALLEAGFQVLRIDTRGHGGSSAPPG------DYRIEELAGDVLSVLDALGFESGV---------------------------------------------------HMIGLSMGGMIGQVIAADHPGRLASLMVCCSASKWMGDTELMKGRIRAVKESGTLESIVAANMERRYGPGFRERRPLRWEALRQT-FLGTKLDGYFGCMNACLTHNVEPRLGQIDIPVLVVAGSEDPTTPPADNRLIASCIPGARYAEIAGghFPNVEFDGEFNRIMLDWLKERRG-----------------------------------------------------    
 528 UniRef50_A4QZG2        Putative uncharacterized pr...   142     3e-32  1   8.0%  86:408     2:325     -------------------------------------------------------------------------------------PALTPDDHRVTHHSVPIDNHVYHYIQSVPTEPrtILLLHGWPDTALTWRHQIPFLtpLSLHVVAPDMLGYGQTSAPADPA----EYSLKKMALHMQALVEHvpGAPLFLAGHDWGAALAWRMAALwpELFAAVACLNVPYLPPDAGEFVDMQAYVDEIPSLRYQVQLSGDEAVAIIDD----ASDHHANLRGFLNGIYDGRGPNGEESFTVHEGVRQPEILRLVGPAKLMGEEWVDYYVAQFAARSFRGPTNWYRTRRVNYEDEK-GMHDAVVTTPAMVVMGDKDEALPPVLADGMEKWVKCLRREIVDAGHWAHWEEADRVN-----------------------------------------------------------------    
 529 UniRef50_A0QVB5        Hydrolase, alpha/beta fold ...   142     4e-32  1  10.7%  96:422     2:293     -----------------------------------------------------------------------------------------------IRSEVDLSGGRVSYLTW-SPERptVVLLHGGGVDNasWGGIAPGLAAAGYRVVCPDHPGYGHSPLPALPV------TQERLVAYVGDFVDAVVDGRYAIGGLSLGGGMTIGHVLDRPDRVTGAMLLGSYGIMARLSDGPFSAIRQALTWAMVRTGVMGSVMRWTARNR--------------------------------TAMNQSVAAIIRNRVYR--TDDLLQAVYAAAAQPGGFIAFEQWQrqVRWNRQRTDYTDRLRTFPRPALIVHGGRDTGVPVAHARAAARLIPDATlvVAEAGHWVQRDAPDVVLDAMTTFLARISATA---------------------------------------------------    
 530 UniRef50_C0WHY6        Hydrolase n=2 Tax=Corynebac...   141     5e-32  1  16.1%  82:417     4:302     ---------------------------------------------------------------------------------APLPPSTVELEGPFAHEFVHTRGIRLHVAIAGDSADPLVVFIHGAFGGWFDFQEVIGPLAQCGYHVAAVDMRGFGMSDKPPIDPGQD----IRTLVGDLDGLIQALGHEDAYIVGADTGGAVAWCLAAERPNRVRGLVSVSAAHPVDIRRAIAARPW-----------DFGWMILRSLLCHLPKVTRANTLLLSPRAYRKELSLDTGPALADA--------------------TLESILALRLRASQIGKVRRGILWNHRMRTAVVPLNWSEL--AVKRPVLFIHAQQRLWNPVIQRAALRARAGFSATSIPGAKNLPFLEAPEEFVDELRRWLDA--------------------------------------------------------    
 531 UniRef50_A0Z514        Alpha/beta hydrolase n=1 Ta...   141     5e-32  1  12.7%  90:419     9:304     -----------------------------------------------------------------------------------------DGTWPFPPCFSTAAGFKQHYIDEGPRDGEVVVLLHGEPTWGYLYRNMIPGLTAK-YRVIVPDHMGFGKSETP-----QDKEYTLRTHVENLEALIEELRLTDITFVGQDWGGPITGAFTARNPKKVQRLFLMNTVLGFGGGKASGGKTPWFQWIEKHANAGTLEGILGEM------------------------------------GSTVLSVMKIIGFQNSRDVSDTWIDAYSAPFPDRASCIGAISFPLDVHEGRFLPFVRETlaLRAKPAMLISGDKDFGIAKDHasDFKGLFPSSPIvvEGVGHFCQEDIPDTLVALIDSFIQSNP------------------------------------------------------    
 532 UniRef50_Q7W1M3        Putative hydrolase n=2 Tax=...   141     5e-32  1  10.0% 104:423    12:285     -------------------------------------------------------------------------------------------------------GLDFHVTEWGSPQGLPVVMLHGIRGYAETFAGIAAALQPE-YRVIAFDQRGRGRTDW-----DADCNYYTDTYVADLAAVADQLSLARFDLLGHSMGGINAIVYAARHPGRVGRLVVEDAGPGAFED-------------------SDGARRIRRELAETPAGFASWDAASDFMRSLRPSVSEQARQQRLESMLKPAPEGGYVWRYD----------------------HAGIARTRLHPDPARVVDLAAYVARIGCPTLLLRGGRSDYLQPAMVERMRALNPRIevEIADAGHYIHDDQPAAFARHVCRFLKPHAPAGA--------------------------------------------------    
 533 UniRef50_Q65IJ7        YisY n=38 Tax=Firmicutes Re...   141     5e-32  1  10.0% 105:415    37:285     --------------------------------------------------------------------------------------------------------VNLYVEDINPQSGKAIVFLHGWPLSHLQFEYQFDDLSRKGYRCIGIDWRGFGRSDKPAT------GYNYNRLADDIRAVVEALQLNNFTLAGHSTGGAIALRYMSRYRGavSKLVLID---------------------------------AAAPVGFTEETAAKLLQQASNDRP------------------------KMMREVTDTFFFQYITQPFSDCFFQLGLQAAGWST-KAVIEMLRDEKLYDDPEKVAAPALIIHGIQDKVIPFAQAKELSRKIRNSYLVPFqsGHGPFWEERKMFNRILTQFI----------------------------------------------------------    
 534 UniRef50_C2AKJ9        Predicted hydrolase or acyl...   141     5e-32  1   8.6%  76:423    32:347     ---------------------------------------------------------------------------RAPAVPQGAKLIEDYAGPRDDGFVVSGDGTRLHVRSWGPVDGPVAVFVHGWTCNVANFPGQVEHFVGRGYRVISYDQRGHGESE------PGAFAYTTGVLADDLHAVLGAAVprKALLVGHSMGAITIMAWAGKYTDEVA-----------ERAHHAVLVSTYARDAVPGFIGATLLAPANRFAPRAVGRLGASVLGSPIKVRHNRLHTAVLRYTALCGYSSYGAVR----------------------YTEDMVATCPSDVRAEWGRVLAGLDVTDGLQKLTIPTSVVVGQFDHLTPPADAETIAAELrdRFAVIRDAGHMLPIEQPEKLNALLDSIVDEEIPAPA--------------------------------------------------    
 535 UniRef50_Q15UK5        Alpha/beta hydrolase fold n...   141     6e-32  1  11.8%  93:415    17:298     --------------------------------------------------------------------------------------------YSFAPHYIEVDdlRMHYLDEGSKEGEVVLMLHGEPSWSYLYRDMIKPIADKGYRVIAPDLIGFGRSDKPTQR----CDYTYQRHLDWIRNILTQLNLKQVTLVCQDWGGLLGLRLVAEHPELFARVLAANTMLPTGDHPPGEAFMKWRAFSQEVPEFPVAG---------------------------------------------------IIKGATVTALSTDVLNAYDAPFPTEAHKAGVRQFPLLVPATTDDPqaWQVLQQFNKPFITAFSDSDPVT--AGGDkmQKLIPgsHTTITQGGHFLQEDQPQQLAKVLLQFI----------------------------------------------------------    
 536 UniRef50_B3R9D3        Putative Hydrolase, alpha/b...   141     6e-32  1  10.7% 100:418     4:262     ---------------------------------------------------------------------------------------------------IASDGVSIHYEVKG--GGSPVLLLAGLAGVGASWGPQIDLFA-AHHQVIVPDHRGTGASEHTA------RGMTIAQHALDMVRVIEAVGCGPVHAVGSSTGGAIVQWLAIHHRERLRSATILSS-------------------------------IARADAFYRRQFDMRRRMLADSGLRASTEANALFLFDPEFQREHPEQVQAWVNTASACPFEPEIVFARIDMIA--------------------GHDAFDDLPSITTPTLVLVGERDFCAPPYFSEELAGRIPGAEFAILNGGHFIFLEKPVMLHDAVEAFIARH-------------------------------------------------------    
 537 UniRef50_Q89HB1        Blr6083 protein n=2 Tax=Bra...   141     7e-32  1  10.3% 100:420     8:269     ---------------------------------------------------------------------------------------------------ITKDG-RLAYEAAGDPGAIPLIFLHGIGGAARAWRHQLATFGDR-FRAIAWDMPGYGGSAPLARV-------SIAALADALQQFIEQLGATRPILVGHSIGGMIVQKWLEQSPKLARAVVLAQTSPAFGKADGDW-------QKSFIAARLGPLDRGETMRSLAPSLVKELVGDNP--------------------------------------------DAEGMELARACMGSVPEASYRAMMLALIGFDQRSTLKDISVPTLLLSGSKDNNAPAPMMAKTATYIPSAQYVELAggHLANLERPEAFDEALGKFLDSVVA-----------------------------------------------------    
 538 UniRef50_A4JPA6        Alpha/beta hydrolase fold n...   140     7e-32  1  10.3%  96:415    14:297     -----------------------------------------------------------------------------------------------TRGAIDANGIEIHYLRQGS--GPLVICSHGFPDHARSFLPLLAELAHAGFTAVAPYLRGY-----APSGTAPADQYHTCYMAHDLRALIDALGVEQAILVGHDWGAQASIAAAILFPERVTKLVALGWS--------------------------------------RPDAAQRLHYD--YLKGIWHTFFFQSPSAEQVLAHDDFAfaWWRDASRNWDVPRDIVEAVKTTFRQPGVLDAALAQYRCRHVALRDPERREQQARitVPTLLMHGTHDREGRLEagDGVPEYFSAarEIVDGAGHFLHLEKPADVNRSIIDFI----------------------------------------------------------    
 539 UniRef50_B5JFV9        Hydrolase, alpha/beta fold ...   140     7e-32  1  12.5%  76:411     2:288     ---------------------------------------------------------------------------SVSKIPDSIREEYP----FEPKRLKLSCGHEMSYVDEG--EGFPVLMVHGNPTWSFYYRNVIKKLSGK-FRCIALDHIGCGLSDKP----GSDYSYRLEQRIEDLGELIDHLDLERFDIVVHDWGGVIGLGAALKRFEKLRRVTILNTAAFVDSRIPARISLCRAPVFGKLIVQGLNGFAGPAASM----------------------------------------------SVKKKPLSKAVKEGMLFPYGNWSDRRAVYEFVKDIPMHVSHPSfnDLLKLRQRDVTILWGGKDFCFNIHFYDRWRRFLPDAtfRYPQSGHYILEDETESCCEAI--------------------------------------------------------------    
 540 UniRef50_C1AC35        Putative hydrolase n=1 Tax=...   140     7e-32  1  14.1% 100:419     3:265     ---------------------------------------------------------------------------------------------------AQLPGFQLGYDDSG--DGLPVVFLHGFPHDRTLWTAQKASLASQ-VRCILPDLRGFGHSS-------THGPFSVDQYADDVVGLMDHLGIERAAICGLSMGGYVAMAMWRRHADRIAAMVFCDTKATADSEEAKGRRDELIAVVK-----------------------------------------------RDGARAIAEAQLDGMVGSTTRARRIEVVNGLRAMM----GRQPVAGIIGALQALRDRPDSRETIGTITVPSLVVVGEEDSLTPIKEARAIAELLPAAARVRLeaGHVPCLERPAATNHALSDFFATLS------------------------------------------------------    
 541 UniRef50_C1E4J4        Hydrolase, alpha/beta fold ...   140     8e-32  1  11.2%  63:420    34:380     --------------------------------------------------------------SAPRARAAVEGARPSLSMPP-LERDDNGVERLALQtfWEWRGHRCNYISAgtNDGPIVVLVHGFGAHSYHWRYTVPALARRGFRVYALCMLGYGWSPKVEEP------YSMEFWGQQvrDVAGASPSDKAVVAGNSIGALAALYAASTAPEQCRGLCLVNSAGNFEEGAKPGPEKPTLAQKA--VGQTKGPDGIRDPTDPNPRPYTALERAQEAIGRVVATGIFYFTKVR---------IKTILEQVYEYRVDEELVRSIALAAEDPGAIGTFYQLSLAGGRTRVTA--GELLEKFDGPLMLLWGEKDPWMTPSKAARILEIKPEAYYAPVVAGHCPHDDAPVECSAKLADWAEALPA-----------------------------------------------------    
 542 UniRef50_B2APA8        Predicted CDS Pa_7_40 n=1 T...   140     8e-32  1  11.9%  96:413    10:324     -----------------------------------------------------------------------------------------------ARKAILSDGTTYGYIHVPagSKPTFLLLHGAPSSSYIWHHQVELLPKAGFGILVPDLLGYGDTDKPES----YEPYQMKCLVPQVHELVTklDTPKVIGVGHDFGAGLLSHLYVHHKELFSQLVFIATGFMFLDSPFDP--DSVIQMSKEILGYSTSGYvgATLVEKNDRRVDSLFY---AQDPKVWIEYFGEPG------GFTKFLESDIEIPVAHWISPAELEMHNRIL-RAGGYTGPFNWYKAagPAKEDQDLPAEEKTINIPTLFIATLKdaVITDMHIQDLRGLAKDLRVEKLDVGHWAMLEGKERVEALLEE------------------------------------------------------------    
 543 UniRef50_Q6CI59        YALI0A01441p n=1 Tax=Yarrow...   140     8e-32  1  10.2%  97:421    14:350     ------------------------------------------------------------------------------------------------HKYAVLGDKRWHYLDIPpgSGKTLMLCHGFPDSWYGWRKQIPILRNLGYRLLVPSQMGYTRSEAPLYPTPEPNeyTGKFYAQCMDELLTQLGIPTVTIIGHDYGAYLGPKLYLYFPHRVEAIATSCWHYVPALKKFFAIPDFIKLAPSLSYQAYLIGDASKDFATR---------------------------EGSEPFLRQVFGGEGWGGSDSSVHMTEPEFQNYMTEFATGGRFLsmVSTyrRLTFEIEKQDFLDKGrqVDVPYLHVGAEQDMAFRPPMIKNLRKYVKPGRlvWIDASHWLMFEKADEFNHELVKWLDKIHGS----------------------------------------------------    
 544 UniRef50_Q1CW78        Hydrolase, alpha/beta fold ...   140     8e-32  1  11.3% 101:426     5:274     ----------------------------------------------------------------------------------------------------TVNRTDLYYEDSG-GTGVPVVFSHGLLWSCRLFDPQVEALCGR-YRCIAYDHRGQGQSAVPPARVI-----DMETVYLDAVALIERLGVGPCHFVGLSMGGFVGMRIAARRPDLLRSLVLMETSADREPLHNVPRYTLLNL-------------------------TARLAGLRPVADPVMRIMFGRSFMTDPNRAEERSRWRTRLMENRRD-------------------------IWRAVNGVIERRGVAHEIPHIRVPTLVVVGAEDTATVPAKAQRLHRLIPGSRLvlPRGGHSSTVEEPALVNATLGTFLDAQLETRTSRA-----------------------------------------------    
 545 UniRef50_UPI0001C324B4 alpha/beta hydrolase fold p...   140     9e-32  1  13.3%  90:412    41:315     -----------------------------------------------------------------------------------------PDEPRRSGSEAQLGDVRVRYVER-PGVRPEVLLLHGLPGTAEDFERVTPLLA--GHRTIALDRPGFGFSDGGYHP--------LTEQLTAIEALLDQLAIRRVIVVGHSYGGTLALAFAARHPERVRGLVLVDAAAAGASTT----------------------GFERAQARLVQGLSLPVVQPLADVTFAQVMRKASAQMGAEEAFGPDPVDDAYERRLLAVTMQHDDLDAYAAETLVAGDV---------------IEQTDEQLRRIEVPAVVIHGDRDRSVTPEHGRRLAAELPRARFVGVPGGHMVPLVHPDVVAEAVR-------------------------------------------------------------    
 546 UniRef50_Q1LSA6        3-oxoadipate enol-lactonase...   140     9e-32  1   9.5% 101:415    16:270     ----------------------------------------------------------------------------------------------------RVGDVHLQVRVDGT-EGPWVILAHALGANLTLWDDTARHLAAR-YRVLRFDMRGHGGSDAPVG------AYTMTRLADDVVALMDELDIAQAHFCGVSVGGMVAQTLGVRHPERLLSLTLVDTIHHTPLEARAMWADRIGQVEAHGMGGTVESTLNRWLTAPFRER------------------------------------------------HPEIVERIRKML-----LETPVRGYVGVAQAIEAFDLARAISRIHCPTLVVVGDKDEGSPVSIAEAIAREIHGSRlvLPDAAHLSFIEQPERFHAMFDAFL----------------------------------------------------------    
 547 UniRef50_A0JTD9        Alpha/beta hydrolase fold n...   140     9e-32  1   9.1%  99:418     3:262     --------------------------------------------------------------------------------------------------TVEISGLRIGYQRAG--QGPPLVLLHGAYEDSRVWSRQLEGLSGE-FTVIAWDAPGCGRSDDPP------PDFSGRELGDALAGFLREVSPRKPHVLGLSWGSGVALELYRSHPNMAASLLLVSAYAGW-----------------------------------------------AGSLPPEEVERRYAQILAELDQPPEQFIPVWMPTLFTARADPAVVQEASDIMADFHP-----AGMRALLSASGHADYRDVLPTISVPTLLLYGEEDVRSPLAVARDMHRQIPGSELVVIPggHMVAAETPEAFNSEVRRFLHGI-------------------------------------------------------    
 548 UniRef50_B5H5W4        Short chain dehydrogenase n...   140     9e-32  1  12.2%  93:452     2:322     --------------------------------------------------------------------------------------------PTARERRISTGGIELCVAELGDATRPTVVLVHGYPDSKEVWSKVATRLAEK-FHVVLYDVRGHGRSTAP---VPLRGGFTLEKLTDDFLAVADAVSPDrvHLVGHDWGSVQAWEFVTVGRTEGRIASFTSMSGPSLDHFGHWIKRRTVRPTPRRVGQLLGQGA-----------------------KSWYVYMLHTPVLPELAWRGPLGKYWPGILQRIEKVPSGDYPT----ASLPQDAARGAWLYRDNIRARLRRPRADAHA--HAPVQLITPTGDAFLSERLYDDLDEWVPQLTRRSLAAKHWVPRTRPDQLSAWIADFVETHE-------ARDGGSRGQETQARPATGPYAEHFAG---------------------    
 549 UniRef50_A0QIV9        Short chain dehydrogenase n...   140     9e-32  1  11.8%  96:467     4:349     -----------------------------------------------------------------------------------------------TSTITADDGVTLAVHRYTdpARPTILAIHGFPDNHHVWDGVADELTGAPYNFVAYDVRGAGESSCPATRS----GYGFAQLARDMGAVIDSLGVGRVHLLAHDWGSIQAWAAVTDQAIMGESVMDKVASFTS------------------ISGPHLNYAGAFLRSARTPRAVARVVR---QLIASGYIGFFLCPGAAELSFRAGIGVKVIAALERIGHTSTRSQRRDTQRSLR--DYLNGLQLYRQNMPAPMLAP-GPRLPETTVPVQTLVPRRDVFVTPALQRFTGAIPQRARVIEIEGGHWVVTSRPDVIARLTTEWVDLQSAGAVAPQLTGERRELPGERREVRGRLALVTGAGAGIGRATAVELARQG------    
 550 UniRef50_Q0RXV9        Epoxide hydrolase n=2 Tax=C...   140     1e-31  1  10.9%  98:418     2:287     -------------------------------------------------------------------------------------------------RSTPVDGFRLTYDRAGS--GWPVVLLHGWPGDRTDYRDMVPLLSE-SLDVVIPDLRGFGTSDKHDADPA--EQYSAKAQARSIAGLIEELDLERPVIAGYDIGSRTAQTLATCRPDLVGALVVA-----PPLPGVGRRALDESAQREFWYQAFHQSPLIECLIDGKPDAVRDYL-----------------------------AYIWGRWSGPHYAVHSDQLEHLVSVYGAPGAFAASIAWYRAGAGAVaaEATPEPGRRVGMPTKVLWPEHDPLFPREWSDRLDDFFSDVELRYLDggHFSPLEAPQPFAAEVLSAADRL-------------------------------------------------------    
 551 UniRef50_B1MFP8        Putative hydrolase, alpha/b...   140     1e-31  1  11.5% 107:419    18:274     ----------------------------------------------------------------------------------------------------------LHVRIGG--EGPVILFWPSLLMTGSMWAAQAEYFADR-YTVILVDPPGHGDSQ------SLTRMFRFEDCAHAITQILDSLGIERAHVVGNSWGGMIGGTFTAMYPHRAGASVLMN---------------------------------ATASRASMRQRVEFRLLTEIVRILGRFREPLTTRAVKAFVGPTIMRERPHVEQSIRAELRDLRVDSV---------------YWAVNSVVPARPDQRELLGRITTPILVIAGREDPTFPVAETELMAEAIPGAELVieNTGHLAALERPEEVNALIDDFLKRHP------------------------------------------------------    
 552 UniRef50_Q0S3C8        Possible hydrolase n=4 Tax=...   140     1e-31  1  10.5%  46:417     7:340     ---------------------------------------------LGLVGGLLSALALGSVAGVNVLRTATRPGREIY-----GDEDFTLMSRDRRSVVVTEDGVALAVREVGPEDAptAVFVHGYCLDMTSWHFQRRQLAErdVRMVFYDQRGHGDSGVPSTASC-----TIAQLGADLATVIEaaPTGPVVLVGHSMGGMTVLAFAGQRPELVASRV-----------------------------------VGVGLVATAAAGLSETGLTRNLQNPVIDGFRMAVRTSPELVQHARGAARVLITPILRAASYGTDVSPRLHKFSDSMLDRTSVVTIVNFLRTLELHDESASLEAirTPSVVVCGDSDMIIPFGSSRKLAADLPESELVRvgAGHLVQLEFPSVVSDAVDRVLHR--------------------------------------------------------    
 553 UniRef50_Q0K2B3        Predicted hydrolase or acyl...   140     1e-31  1  10.6% 100:419     4:263     ---------------------------------------------------------------------------------------------------ITSDGVSIHYEVKG--QGSPVLLLAGLAGIGASWGLLIELFAE-HHQVIVPDHRGTGASEHTA------RDMTISQHARDMARVIEAVGCGPVHVVGSSTGGAIAQLLAIHHRELLRSASIVSS-------------------------------IARADAFYRRQFDMRRRMLTDSGLRASTEANALFLFDPRFMREHPEQVQAWVNATAAGTFEREIAFARIDMIVGHDAFD--------------------ELPSIATPTLVLVGERDFCAPPYFSAELAKLIPEAEFAILDSGHFIFLEKPELLHDTVEAFIAKHE------------------------------------------------------    
 554 UniRef50_D2LB54        Magnesium chelatase accesso...   140     1e-31  1  12.0%  84:434     6:307     -----------------------------------------------------------------------------------WERDGLHWPNRAASRFVSAGGLRWHVQEMGS--GPTVLLLHGTGASTHSWRHFMPILARR-FRVIAPDLPGHGFTEAPRTSSLL----TLPHMAQAIATLLGTLHVKPDLAIGHSAGSAILCRMALDGKLGAQAIISLNGAFLPFPGIASQLFPMIA----RALFLNPFVPGYFAWKARDKVTVRRLI------------------------------------EGTGSHLDAEGLAQYGLLLGNSTHIASAL----GMMANWDLQSLSNDLPKLKTKLTLIACTEDKAVPSQVAFKVYNMVPGSKVILLrmGHLAHEEKPQEVVDIV---FAEAEraKDEYPAEPQPAAE---------------------------------------    
 555 UniRef50_D1UN06        Alpha/beta hydrolase fold p...   140     1e-31  1  13.2%  94:418     2:303     ---------------------------------------------------------------------------------------------IRETLSVDAGEVRLAVYVSGPRDAPPIVLVHGYPDSAAVWEPLRAQL-DGGHRVISYDVRGAGASDAPAARS----GYRLQRLAADLSAVVDacGGKPFHLVGHDWGSIQSWQAVTDPQLKGRIASYTSISGPCLDHAFDHALDRASRGSRHADSAANKDRMTKQLRGTRIRQTLRSWYIFFFHLPWLPELAWRVGGARLWPHWLR-------------------VTEHVQAPRDPAQLRNALNGLNLYRANFLDKLLHPQARRAHAPVQFIVPLRDRYVGPELSLGQEIWLGSYERIEIDAGHWAVLREPELIAAYIARFVARH-------------------------------------------------------    
 556 UniRef50_C4DIM0        Predicted hydrolase or acyl...   140     1e-31  1  13.2%  74:445    42:390     -------------------------------------------------------------------------RARSDPNDPYAEEPFANLHADDIRTITTADGIDVHVEtrGVGEAetAVFVHGFCLDMGTFHFQRRALTEStlRAVYYDQPGHGQSGELPLP-----EYDIESLAEALYAVLaaVPTGPVFLVGHSMGGMTILAFARRYPELFASRVRGVALLSTTAGGLDKV--------------SFGAPKALARARKV-----------FLPALSKAIALTPGVIDGARRVASDLAWRLTRRYGFATRKPSPSLVSYVEKMNTATPIKTVIGFSKALLE-HDEHDVLPQLASI--PVFISCGDSDQFTPPEHSVALAEALPHAKliVPEAGHVALLEKPELVTDPLFAHLSECLDarDRQPAEPVKRRKPWLRKKKRTGD----------------------------    
 557 UniRef50_D2B2R7        Short-chain dehydrogenase/r...   140     1e-31  1  11.0%  98:461     2:326     -------------------------------------------------------------------------------------------------RWVRSGDVRLALFEEGDPAAPTVLLLHGYPDTHRVWDEVAGLLRDR-FHVVRYDVRGAGRSGAPRDG----GDYTYDHLAADMAAVLDAVGKPGVHLVGHDWGSIQGWELIGRPEIKGRVTSFTSFGGPGLDQAAHFMRHGRR--------------------AEVAGQLLRSWYIGVFQAPVAPELAWRTVMPRVLERTMRISEGIAPRPGHPADTLRED-------------GVNGLGLYRANMSTVRRTPRAE--PVVETPVQLIETTRDPFVSPALLASLRPWVPRMWHRRIAAGHWAQRSHPEVIARMIAEFAEHVELAEGAGPAEGVGGAEgrGVRPSRELRRARTGAGERGPYEDRL------------    
 558 UniRef50_A5G0V0        Alpha/beta hydrolase fold n...   140     1e-31  1  10.0%  90:418     3:278     -----------------------------------------------------------------------------------------EHEAGRVHRVMGSGGVGLHVREWGSEHAQPILFIHGWSSCYLAWRSQFDSALRERFRLVCVDLRGHGFSDKPLDPAMYQ---TSAHWADDIAAVMIALGLRRPVLVGASYGGYVIGDYLARHGTKNVSAVNFVGAAVISSNPPVNIGRRF-------------------------------------------IEGALMAASDDPAVMIEGARSVIHATTHRPLPPADAERALVAFALTPPAIRAFL--------LMRTLDFTRTLAELDRPVLITQGDEDAIVLPSMAEHIAATVPEGRllYPECGHTPQYEFAERFNGELAEFAGAV-------------------------------------------------------    
 559 UniRef50_Q8W3F1        Os10g0498000 protein n=12 T...   140     1e-31  1  11.4%  96:419     6:311     -----------------------------------------------------------------------------------------------EHSYLPIRGLKLHIAHIGKGEAATLLFVHGFPEVWYSWRHQMIAAAAAGFRAIALDFPGYGLSEPPADLT----QASWQGLMNDLLAILDSLSIPKVFLVAKDFGVKPAYDLALCHPDRVCGIVSLGVPPLVESLSFSGLPEGFYIHRWR-----EPGRAEADFGRFDTRRILRTIYI-----------LFSRSEIPVAKQGQEIMDLADESTPMPQWFTEEDLSAYTDLYEKSGLMTAIQIPYRTKAAKAEGAN-----PRFEMPMFVIMGQKDYILKFPasEKLKEIAPDYGItiPEGSHFVQEQFPDLVNQLVIDFVSKHA------------------------------------------------------    
 560 UniRef50_D2RTL8        Alpha/beta hydrolase fold p...   140     2e-31  1   8.7%  98:419     2:288     -------------------------------------------------------------------------------------------------PRATRDGVSIYYedESEGDGTPVVFVQGLGYGRWMWRWQREAVADDR-DVIAPDNRGTGRSDAGLPPLLPraGYSIGGLAADLEEVLADAGIRRAHVVGASMGGMIAQRYALEY-TRAETLTLCCTSHGGPDAVPVPDETQEFIFDVP----------------------------DGANERETIRHRMRPAFTERFTNRNPHLMDRIIEWRLEQDADDPAREAQAAAVLD--------------------FDVSDRLEGLRVPTLVLHGTDDRVVPFENGLLLEEAIPNARLERFEGghCFFIEHADRVNEELRAFLADHD------------------------------------------------------    
 561 UniRef50_D1ADZ9        Alpha/beta hydrolase fold p...   139     2e-31  1  12.5% 105:423     3:263     --------------------------------------------------------------------------------------------------------VQLYARDVG--EGTPLVLLHAFPLSSAMWLAQREGLGGR-FRVITPDLRGFGGS------MLGEQEPSVDVMADDVAHLLRRKGIDRAVIGGLSMGGYVAMALCRRHPDLVLGLILANT-----------------------------------KASADTEQGRRNRLRQAERLEREGTSRVLVEEVLPLLVGPTTMRQRALVYGRVRGLVQAAPAAAAAWAQRAMAAR---------------PDSFETLRGVHAPALVITGTEDELSPQADARAMVEALPNAElvIPRTGHLSAVEQPDLFNQIVAEFVAALARTPR--------------------------------------------------    
 562 UniRef50_Q2BJ40        Putative arylesterase n=1 T...   139     2e-31  1  12.1%  97:418     2:278     ------------------------------------------------------------------------------------------------EHFTRPDGTEIHYISLGEGE-TTLVFLHGWTASVREWLPFASELAE-NHRVVSWDARGHG-----AHSYHEESEISLPAMADDLEAMLEHLQIEDAVLVGHSMGALTSWEYLRRHGdrLRGLCIVDQSPKLVTDDE----------WQHGVYSDFDKATNTAFLHRLTENFAEAVLEL--------IAFGKNRRSRENYEQNSRGFQQMREYLQKQ----------------------PGDLLTRCWDSITQQDYREVVAGIKLPTLLIYGDESQFYSAELQQWVKQNIESSELHIYesDHSPHLWHKERFIYDLNRFIDSL-------------------------------------------------------    
 563 UniRef50_B1M6J6        Alpha/beta hydrolase fold n...   139     2e-31  1   8.9%  92:416     7:294     -------------------------------------------------------------------------------------------PGFDRHRIETAPGVTINARSAGS--GPPVLLLHGHPQTLSTWLQVAPRLAER-HAVVAMDLRGYGDSSKPPGG-ERHANYAKRAMAADAAALMRALGHERFAVVGHDRGGRVAHRLALDHSRAVERIAVFDIA--PTATMYARTDKDFATRYFWWFFFIQPHPLPETLIGADPEYFLRHHVDG--------------------------------QSKTPGSTPPELFAEYLRCYADPACRHAICEDYRAAAGIDLEHDAADAEARITAPLLALWGAKGVvrTYDVLETWREKATDVSGRALDCGHTLQEERPDAVLSELAAFLR---------------------------------------------------------    
 564 UniRef50_D1CAV4        Alpha/beta hydrolase fold p...   139     2e-31  1  12.1%  72:425    22:329     -----------------------------------------------------------------------TVARRRADPAPPRTLLPPEALADDDSVFIDVDGLRVHAKLAGQGD-PAVVLIHGFAAGAFIWRRVLPPLARRG-TVVAFDLPGYGLTARppDAWPRGNPYDPEVQADLTIALLDRLGIARAVLVGHSAGARIAMLAALKYPERVSGLVLVTPALDPP---------------------SLRRSLALLVRAPGLDRVLPRVAQSAASRAAYILRYAVY---------------------DRAVVTDDLIKGYLTALQVDGWDAALACALRATRPLG----LLDRFGELQVPFMAIFGMYDRIVNPRQVLRMTA-VPDlfVFMEHTGHLPQEEKPEDFVEFIEDFLDHIEQRASER------------------------------------------------    
 565 UniRef50_Q394Z8        Alpha/beta hydrolase n=10 T...   139     2e-31  1  12.2%  90:418     9:306     -----------------------------------------------------------------------------------------DGTFPFAPHFDDASGFRMHYVDEGPHDGETILCLHGEPTWGYLFRHLIAALSP-THRVVVPDHMGFGKSATP-----QDRSYWLQDHIDNLERFVLARGLDRITLVLHDFGGPVGMGLAARHPDRIRRIVSANGPTPFGQPDLADRLNANGRD------APWFQWIMRAAADDTLETVLGQLGFNILSTL------------------------KLNGFENHAIINDTWIAAYGAPFAQPKDCVGAIGWARGFAarFEEPDAAALRAIREKPALAIWGDADRTLGAEhlPLFTALFPSAPVERLAggHYCFEDAPDAIAARIAEFIRTT-------------------------------------------------------    
 566 UniRef50_D2PX09        Alpha/beta hydrolase fold p...   139     2e-31  1  11.6%  89:417    10:299     ----------------------------------------------------------------------------------------TSFEGFAHHRVTVADGVTLSAVVGGT--GSPIVLLHGFPQTHLMWRHVAADLAAD-HTVICPDLRGYGASDKPAETDGST--YSKRTMAADVVALAQALGHERFALAGHDRGALVAFRAGLDHPDVITQLACLDVLPTLDMWSV--MQGTSATVGFHLYLMALPPGLPEQLIAGSPDAFFGYFLD--------------------------------LWVKDPAAIPADVRTAYLDACR--AAVPSIVADYRASAGIDITHDEADLAaqLSMPVSVVQQDWGAALGFDAAARWRAWAPDLDHTTTTSGHFMAEESPAEIAKILRTLLQR--------------------------------------------------------    
 567 UniRef50_C6CXK3        Alpha/beta hydrolase fold p...   139     2e-31  1  10.6%  90:420     7:298     -----------------------------------------------------------------------------------------RADYPFESRYLNVNGHNLHYIEQGSGN--PILLLHGNPTWSYMYRNLIPYLDKLG-RCIAVDLIGMGRSEKP------DIGYTFLEQVDYVSKFIEQLGLKDIIIVGHDWGMAIGLQYAGSHSENVTAVAMLEPQALYPCPDWEAFTPAESAELFQKLRDPEEGWPFMR--------------------------------------DNNVFVEGMPHIIINRTFTVEEHEFYREPFRVPDSRKPSWVFPNQIPIEGKPDEVaeWFTQTPIPKILFYADPGCTIREPQMAWCREHLKNLTLFPIGKghHLLEENPHVIGQELQRWAGTLSR-----------------------------------------------------    
 568 UniRef50_D2S9T6        Alpha/beta hydrolase fold p...   139     2e-31  1  14.9%  92:419     4:275     -------------------------------------------------------------------------------------------PTTHPQRTVQVGDISVAVQEYG--EGEPLLIVNGTTQSLGFWAEPAPVLASR-HRVVTYDLRGMGGSER------GSGEISVASLAEDARGLLEALDIERAHVLGYSLGSAIAQELALAAPERVASLVLYCTWARTDGFQRAVITG-----------------------LAHPWRTGDMEAALGALGIAFSPQLLDSPEFGELVEQLLPLF----------PSTEQQVRTTVEQW-----------------DADLAHDTLDRIGGITAPTLVIAGEQDLLTPPWHGRQVAESIPGARLHmpGSSHALGVERAEEFLPLVQDFLAEHP------------------------------------------------------    
 569 UniRef50_B5WEU5        Alpha/beta hydrolase fold n...   139     2e-31  1  12.2%  99:418    20:287     --------------------------------------------------------------------------------------------------WVDVGGVGTRYLEAGPADAPVVILLHGTAGSLENFSTNYAAYA-RHFRVVGLDMLGCGWTDKP------DHDYLIKDYVEHVRGFMDALGIAKASVVGVSLGAWVSAALALAYPTRVEKLVLVAPAGIITDP---------------------------EEEKRYGDAVRKSRSSAAAEPTWEMVSGALTKL-----------------MLNPETLSDELIAVRLEIYRDPRMKAAMPRLLA--FSLGGQALSEEQWRSLSQPMQVIAAVDAPNMFLGNAHAIARIAPNAELLElgCDHWAQYEQPDVFNEHSIAFLQGV-------------------------------------------------------    
 570 UniRef50_D0LIN8        Alpha/beta hydrolase fold p...   139     2e-31  1  12.5%  91:417    83:381     ------------------------------------------------------------------------------------------PYPVAVRRfaQGQELEMAYMDVAPSGgqTVLLLHGKNFSGAYWADTIAALSQRGYRVVAPDQIGFGKSSKPRA-----FQFTFQALATHTAALLDELGVERAAVVGHSMGGMVATRFALMFPQRSAKLVLVNPIG--------------LEDWKRVVPYQPVDAWYANELKKTPEGIKQYMTKSYFDGVWKPEYEPLLAIQAGWAQGPDKDITSWVSALTYDMI--------------------------------FTQPVLYEFGELRMPVLLIMGQRDrmVPAEVaeRTRDAIPNAELVELEggHIPQFESFDAYIAALTDFLGR--------------------------------------------------------    
 571 UniRef50_A6GG16        Putative uncharacterized pr...   139     2e-31  1  15.1%  99:415    55:325     --------------------------------------------------------------------------------------------------TALLGEANVHYWDSGdaGAVPVVLVHGFGASawQWHEQVGPLAAER-RVIVPDLLWFGES------WSRKRDFSIDHQVETLVALLDHLGLQRAHFVGISYGGIVVHELAAMHPDRVAKLAIMDSPGRVYTEADHA----------ALLARFEVEDFADVLVPTEPEDIETLMALGYHKPPRAPRW-----------------VHRQVLEGMYGEFRDEKAALLARLLEQ-------------------LDELDERPGKVTQETLLIWGEHDPVFPVEIGERLAAELPEGtrVVEGASHAPNLEHGALVAKWLVEFL----------------------------------------------------------    
 572 UniRef50_D2PNL5        Alpha/beta hydrolase fold p...   139     2e-31  1  10.6%  99:420     8:341     --------------------------------------------------------------------------------------------------VTTTDGVRLHVETTGSADAptVVLGHGWTCSTRSWHHQLEGlgPDAVRVVAYDHRGHGRSGAAPTGTT-----RIEQLAEDLVTVLDELVpgPVVYAGHSMGGMTLMALADQRPdrITGAVLVSTSSGQITSRAFGLPARLDGAAAVVAPRAVNLAGARLERRELRRTGRGAEYGVRAARNEAAQDAAEQAAEQAAIRARLAVWrrVRRPAVKRFVFGKKVDPAEVDILLADLDALPGRSFSGFFEAITRHDLEHALEVltIPVEVMHGTRDRLLPPRHGNRLAELIPSARLWmpGAGHMLMQERPRDVTHRLASLARKATA-----------------------------------------------------    
 573 UniRef50_C1C0X6        Abhydrolase domain-containi...   139     2e-31  1  11.3%  42:420     3:344     -----------------------------------------RPLLLRAIGFLIAIVVFIPLVIQASFKFI--LKEAFVTKPRKDRPIVFDSYG--THRYLElgNIGIHVVDSGTGerPLMLFVHGFPDFWYSYRHQIKYFSSQ-FRCVSMDNRGYNESDKPE----NIRDYAVDKLANDVKEVVQLLGYDKCILVGHDWGGSICYRVCALFPEIISFYIVLNMPHPLSLQDSLKSSWAQKLSSWYFFFFQCP--------------VLPEVLINSFDMKNWDKIFQDIPYDSKEEQKQDS-------------------EAYKYVFSQRNALRGPVNYYRCSFQYPEDMRRLND-KIIRVPVCSIFGNADKFFLRESFMGSVKYVEDLTIKELKgsPWVHLHAKEDVNRTMDDYITQRRG-----------------------------------------------------    
 574 UniRef50_A1ULJ5        Alpha/beta hydrolase fold n...   139     2e-31  1  12.1%  33:413     3:312     --------------------------------VETPKRRRFVRFCLWSLALAAIATVVVNLTV-----------AELPPMPAA------------EGRYISVQGRDIHYVER-PGQGMSVVMLHGLPGTSEDFDPLIPKL--PGLHLVSIDRPGFGWSRGGWLP--------YQDQIEVVHAVLNRLELAPAILVGWSFGGSLALGVARRYPEDVARMVLIAPA-----------------------------------AGGMRSYTRDLVQARYLQFSQQPVVKSVISRTYGNIATRVSAYFGARGAFAPAPVDPTYQERLLAVSLTPGNLDAFSRDQLEYNATMQWLD--DEVAEVRVPSTVIAAEGDQLVPVEHARRLAKTLPHTELITVDGNHMIPYTHPDVVADEIRA------------------------------------------------------------    
 575 UniRef50_D1SRE1        Alpha/beta hydrolase fold p...   139     2e-31  1  10.2%  95:419     3:281     ----------------------------------------------------------------------------------------------RREQVMSLQGLRFHLTEWGSPAARPVVMLHGIRGYAETFAGLAAALQPD-FRVIAFDQRGRGQTDWDPAG-----NYYTDTYVADLEGIVDALGLDAFDLLGHSMGGMNAIVYAGRHRERVRRLVIEDAGPGAFDASEGAT------------------------------RIRRELAMTPASFPDWEAAADFMRALRPTVTEEARLQRLQSMLSPLPQGG-----------FTWRYDHAGITATRLHPDPTRAVDLEASLRAIACPTLVVRGGRSDYLQPAMAERMRALNPRIeiEIPDAGHYIHDDQSALFAQAVAGFLRSAA------------------------------------------------------    
 576 UniRef50_C4DU41        Predicted hydrolase or acyl...   139     2e-31  1   9.7%  88:418     1:281     ---------------------------------------------------------------------------------------MTTLPSPQRTRVTTSAG-TFDAIAAGPSEGQPVLFLHGFPELSTEWAHYVALFGSLGYRAVAVDQRGY----SPDARPPDPKDYHLDYLAKDVVDIADSLGWNDFHLVGHDWGAAVAWVVAARYADRIKTLCAVSVPH---------------------------LGAFAKALRTDPEQQKASAYMQLLRQPGKAETVLLGEDAAALRGAYVG-------------VPDSNVEVYVEHLSQPGALTAALNWYRVDGFDGRDE-------KVAVPTLFIWGTKDSAVAVSGVDDTVNWTTgyRLeiEGARHFTPEECPEIVGPMIQAHLESH-------------------------------------------------------    
 577 UniRef50_A0LMJ6        3-oxoadipate enol-lactonase...   139     3e-31  1   6.6%  99:416     2:260     --------------------------------------------------------------------------------------------------RVRVNDVLIRYEMQGPPGAPVVTFSHSLAAALESWDLQLPPLRDA-YRVLRMDTRGHGGSSAPPGP------YTMEMLSSDVIGLLDHLDIARTHFVGLSLGGMIGQVLAVGYPERLDRLVLCDTSNRMPSETAPVWE----------------------------ERIRTAETQGMTALARETLERWFSEAFRRDQPQATERIRNMI-------------------------VRTPVAGYTGCCRAISRFDLSGELSRVKVPTLIMVGEKDEGTPVSAAETIQRQIEGSElvIPGALHLSNIEGAAHFNRRLLSFLS---------------------------------------------------------    
 578 UniRef50_A4XF38        Alpha/beta hydrolase fold n...   139     3e-31  1  12.7%  95:417     7:274     ----------------------------------------------------------------------------------------------TTHRV--SGGYDIVIAEAGDRAAPAVVFIHGSgsGASNFRQNIDAFVAAGYRVILPDLIGYGGSSKPEGL-----DYTLQLFTDTLYEALVAHGISAASLVGNSLGGGIALLMTLDHPEFTRNLVLMAPGCVAEREAY-------------------------------------------FVMPGIAKMVSNFGGPDFNLAEQKRLVSNLVHPDFAPNIPDTLVAERFEVART---------QPKDVIVRMRTPDLSPRLGEIDKPVFVLWGLNDEFCPEAHSRLFLDHCPDVRAITFgtGHWVQVERAAEFNAYAIEFLDA--------------------------------------------------------    
 579 UniRef50_C7Q5U3        Alpha/beta hydrolase fold p...   139     3e-31  1  11.5%  98:418     2:285     -------------------------------------------------------------------------------------------------PFTMSNGVRLAYDEAGTAggPAVVMTHGGLADRRMWDHQFHALAE-HCRVVRYDMRGLGESEDSRGAFARCDDLLGLL---------DALDIERAILVGNSIGGAHSIEAALSAPERVSGLALICSGLTEYDWPAEMTTE---------VGHLIGDRVPPERLARYWDRSAESIDPADVAVLAEVNVSYMVAGPKRSLQDLDPAVRELALEMCRNNFMRDwvPQYQERFLQP--------------------PIIDRLRDIAVPTLIVNGVHDPSAIQEIADLMAVRIPRVTRVDLEAGHLPPMEAPEETTRVLLEFIAAL-------------------------------------------------------    
 580 UniRef50_UPI0001C31034 alpha/beta hydrolase fold p...   139     3e-31  1  11.0%  88:415     1:276     ---------------------------------------------------------------------------------------MSAALESARGLTIDAAGHRTNYMEEG--EGPPVVLLHGSgsAHANWADVIGPLAAAGHRVVAPDIAGFGLTE-----LKPDGAYDIKVWVSHLIGVLDALGIERAALVGNSFGGGISLAAALRHSDRISRMVLLGTPAGE--------------------FALTPGLRAGWEYEPSPAAMRALLELFPHDRAF---------------------------------VTDAMVQARYEASARPGAQEAFRKLIPAPAPDGAETIVRgaLRTIELPTLVVHGREDHVVPFELGLRIHRCIAGSELHAfdCGHWVQVERRRQFLRLVTDFL----------------------------------------------------------    
 581 UniRef50_B5HTB8        Alpha/beta hydrolase n=1 Ta...   139     3e-31  1  10.6%  96:415     4:259     -----------------------------------------------------------------------------------------------TRQTLELGNGAISYRTQG--EGPPLVLV---STLAGTWIRQIPELS-QHFSVITYDMRGFGDSPSSQGFPSN------AEHADDLDRLLEELGIPRASVLGLSHGGLVAQHFALRHPHRVHRLALVATFGRPGSSTGIFLNMLNGFLDRDDLPNFWEVLKSFLFSAAGWERMTRM----------------------------------------ETHLRRAMFDQY------------TVAALRSIYGQAREHDLLAALGAIHSPTLVIGGAEDMLFPPSVTEALAAAIPGASLELLPAAHVPPVEAPLPFNKIVTEFF----------------------------------------------------------    
 582 UniRef50_A9WPL6        Haloacetate dehalogenase H-...   139     3e-31  1  11.9%  92:419     8:296     -------------------------------------------------------------------------------------------DGFKYHRVPGVNGVNINVAVGG--EGPAVVLLHGFPQTHYMWRHVAEKLAQE-HTVIALDLRGYGNSDKPEASSAGT--YSKREMAMDVVVVAAQLGFAHFGLIGHDRGALVAVRAGLDHPGVVDYLGILDVLPTLDTWAV--LQGVNAKVAWHLYLMAQPVGLPEKMIRAVAAEFFGSFLTAGDP--------------------------------TGSTFTKQEHDYYVD--SSTAAIDSIAADYRASASVDLEMDLADrgAQLSMPVGViwGSQ---LGFAAAALWRAWAPGSTYQPTTSGHFMAEENPDEIAAFITGLAARAE------------------------------------------------------    
 583 UniRef50_P59337        Haloalkane dehalogenase n=4...   139     3e-31  1  13.0%  98:427    10:310     -------------------------------------------------------------------------------------------------RRAPVLGSSMAYRETGAQDAPVVLFLHGNPTSSHIWRNILP-LVSPVAHCIAPDLIGFGQSGKP------DIAYRFFDHVRYLDAFIEQRGVTSAYLVAQDWGTALAFHLAARRPDFVRGLA---------------FMEFIRPMPTWQDFHHTEVAEEQDHAEAARAVFRKFRTPGEG---------------EAMILEANAFVERVLPGGIVRKLGDEEMAPYRTPFPTPESRRPVLAFPRELPIAGEPADVYEALQSAHapKLLFTGEPGALVSPEFAERFAASLTRCALIRLGAghYLQEDHADAIGRSVAGWIAGIEAVRPQLAA----------------------------------------------    
 584 UniRef50_C1B326        Putative hydrolase n=1 Tax=...   139     3e-31  1  11.6%  40:444    63:421     ---------------------------------------RTDAATLGDLLAGRVSVADAFLSGALEIRGSMTTVLAIGGTLAPDVELPTRAQARETSAY----GVRTGYLEVGAPGTRPVVLLHGLGANNSSMLPVLADLASD-HRVICPDLPGFGSSAAPA------WRYTPEQLHRWLRAFLDVVDARGAALIGHSLGGRVALELALRDPDAVTGLVLLCPAM-----------------------AFRRRRRLTSLARLLPQDVARLPLAVPPRLLHAGTRAGLRALLADPDVVPRHWYEAAA---------DEWELSLRHASRRRALWSALLGLY--LDEPFGETGLWDRISGLEVPALFLWGDRDTLVPARFDRHVTSAVPTARSVTLrcGHVPQFEQPETTLRLIREQLA--VTSRRGAVGKRRPKRARPGAGTGRIA-----------------------------    
 585 UniRef50_Q5LU02        Hydrolase, alpha/beta fold ...   139     3e-31  1   8.3%  99:422     7:299     --------------------------------------------------------------------------------------------------TIDLNGNPFFLRRWGDPALPPLLMLHGFPEYGGAWADLAPHLAHR-FHCIAPDQRGYGQSWAPEGVAH----YATSHLVADMAALVGTLG-TPLTVLGHDWGAAVAYGLAMFRPELVDRLIIANGVHPVPFQRAMAAGGAQSAASQYM------------NALRAPEATEHFAANDYKALTDFFTRH----------------------MGGHDWLSPARLAEYKAEWARPGRLDAMLNWYRASppRTDLPDLPLDRLRVRCPHLLLWGPDDRALLPEATEGLEDFAPDlrITIPGTDHWLCHQAPDRVAAAILDWMPQPETAR---------------------------------------------------    
 586 UniRef50_B6A024        Alpha/beta hydrolase fold n...   139     3e-31  1   9.6% 104:417     8:291     -------------------------------------------------------------------------------------------------------DLEITFMETGTQHGKAVLLLHGWPDDASTWEAVIERLKDRKFRFVIPYLRGFGPSRFRNDHAPRTANGGI--LAMDAIALMNGLGIERFSVVGHDWGSHVAECLAIGWPDRVERIALLSSTPRMGGLRTPPFKQAQLYWYQWFMATKRGA----EAIDKDRAGYARLQWENWAPEGWFDEATFGK------VSKSFDNPDWLGVTLHSYRVRWEEVEPDPRSVWLEKQVKAT--------------------KSLSLPAIFVHGMEDGVTPVATSEDLYKKFTGPFERIlnVGHFPQREDPEAVARELSVFLEA--------------------------------------------------------    
 587 UniRef50_A8LKE2        Haloacetate dehalogenase H-...   139     3e-31  1  10.8%  95:417     5:294     ----------------------------------------------------------------------------------------------FTRSRFATNGITLSVHQAGR--GTPLILLHGYPQNHACWSRIAPTLAE-HFHVIIPDLRGYGDSDAPPDNADHST-YAKRTMARDITGLMDALDLPRAHILGHDRGGRVAYRLALDAPDRVDRLGIIEIV--PTGAFWANWDATLAMKAYHWTFLAQPAPLPERMIGADP------------------------------VAYIDWTLAAWTRAKSLDVFPPEALDSYRAQARDPARIHAMCADYRAGAtrALDDADAAAGRKISAPLMFLWAKGGFPapANHWRAWATDVRDASCV---SGHFAMEEAPEDVLATFLPFFKG--------------------------------------------------------    
 588 UniRef50_Q5MD37        Putative uncharacterized pr...   138     4e-31  1  11.9%  96:414     2:293     -----------------------------------------------------------------------------------------------AERMVKSNGIELWTESFGEPGNPPILLVMGASAQGLLweEFIELLVAGGHYVIRYDHRDTGQSTCLDFQTNP---YTLEDLARDALGVLDAYGIAAAHLVGASLGGMICQLMSIRHPERVRSLTVMMSTPLRPGIMESFQQAFQGKTPEGAFPPPAPRAIAVQLAAASNPPRNREEAIDFQVKMARTMAASAAP------------------------FDEQECRRMVERMFDRA--RNPAAAMNHGFVPSPTHEQAEALRHLRVPTLVIHGTDDPMFPAAHGVAVAERISGAKLLMLEggHDLPRPLFGEISRALLTH-----------------------------------------------------------    
 589 UniRef50_B5JW83        Short chain dehydrogenase n...   138     4e-31  1   9.4% 100:419     6:296     ---------------------------------------------------------------------------------------------------VLNRGVELYAQVDGPEDAPTLLFLHGFPDDHRTWHRQVEGL-KHRYQVITFDMRGVGRSSTPTDR----GAFQIEPILSDIEAVINAVVggKVHLVGHDWGSVIGWSFVAEAYYASRVLSYSSMSGPHLGLMLDWGRRNALSGN-----------------------LTRMAKGAKQFLFSWYVYFFNVPYVPEFLIRRVGKRLWQTVLMQNGV---DRHDPYIQEVDQAQVESIMLKPLELYRQNPIRPPSIPMKGSIDVPVQLIIAAQDRFISEDLFEFYDEYVNHLVRHPIDAKHWAHHSHADQFNRWVSQHVDGVE------------------------------------------------------    
 590 UniRef50_A0R6Y9        Epoxide hydrolase n=1 Tax=M...   138     4e-31  1  11.1% 104:417    19:285     -------------------------------------------------------------------------------------------------------DVSLHAVIAGS--GPAVFLLHGWPQTWQEWLPILEDLA-RHHTVVAVDLKGAGGSSKP------LLGYDKVTMAEELDILREKLGFDTVQVVGHDIGGMLAYAWAATHRDTVTRLAVFDVPIPGASLWDGILTDPLA---WHFAFHQCRDVPEFLIAGRERGYVEYFLR---------------------------------GRTANQDAFTDAAVDHYAHALAQPGAVRSTLEWYRAFAQDADDNRKLAREP-LTVPVLALGGEH-RWGpvDVLREFATDVRGGS--IPDCGHWVVEERPDVVLAELEAFLQR--------------------------------------------------------    
 591 UniRef50_B9JLA0        Epoxide hydrolase protein n...   138     4e-31  1  10.6% 105:416    15:297     --------------------------------------------------------------------------------------------------------LDIAFEEGGPRDGFPLILVHGWPDDVRTWDRIVPQLQSAGFRTFVPYLRGVGPTRFRDPDAMRSGQ--LSALGRDVLDFAEALGLGRFGVIGHDWGARAAYIAACLAPDRVACCVAMSVGWGTNNPDQPLSLRQSQNYWYHWLMALPRGEALVRN--------DRLVFTRYIWTIWNPDW-----------AVPDEEFDATATSFGNPDWADVVLHSYRVRWDLADPDPSYAA----------LEDRLKADPIIHVPTLVLHGGSDPCNDVSTSDGKDEFfpYQRVVLPGLGHFPQREEPDAVLKHMMGFLS---------------------------------------------------------    
 592 UniRef50_C1B3S8        Hydrolase n=1 Tax=Rhodococc...   138     4e-31  1  12.7% 104:435    11:285     -------------------------------------------------------------------------------------------------------GVRVKVLELGVGD--PVLLIHGWSLSSAVWDRQIRVLAEAGHRVLAMDLRGHGESDAPLSR------YDIDRLADDGAAVLDAFDARGATVVGWSLGGMTALRMAHRFPGSVDRLVLVASNGVAGAR-----------QPDYPFGVPADAVEGGMHAAEHTGRLDYRRRAVGDPFGTPP-------------------------------------DGQTLDWLQRISLQTPSWAANACMTTLLRTAQAFALDGLDIPVTQIIGTADPALSVRGARWVQERI-GSTLVELDCGHYPMLERPDLFDEALLRAVRKPKEPTVRRVEAPSSHSVQ--------------------------------------    
 593 UniRef50_B1XNL6        Hydrolase, alpha/beta fold ...   138     4e-31  1  13.0%  94:417     5:280     ---------------------------------------------------------------------------------------------PPKKHTAAINGLPLSYYEW-HQGGEPVVLLHGLADHGLVWASLGESLGKK-YHVVALDLRGHGDSGKPET------GYTFADYNADLGGICQHFGWSQVNLLGHSWGAKIATVWATQNPEKVKSLILADPFFMGQLPLWWRYTFPIG-YRVLPFLKLLGQFESYHQAEAIAKKLKQYRGWNEWQQAICRESFLENPDG-----------------------------TWTSKFVKRACDEIFLDILR----------VKGLTQPLGMPSLFVQPEGGLNRLEFQLKPYRQYLTNLQWQKVPGNHWAHMVEPEAFNQAIANFLTR--------------------------------------------------------    
 594 UniRef50_B1M9D3        Alpha/beta hydrolase fold n...   138     4e-31  1  10.5%  82:413    82:372     ---------------------------------------------------------------------------------AARAELRPAQDGEVRHGTVRADGVDVFYREAGRPDAPLLLLLHGFANSSFYFRHLMPKLADR-FRLIAPDLPSFGFTQVPEDRAYV---YDFASLSRTMGAFVDALGLDRYILYAFDYGAPVAWDLALTRPDRVAGIVSQNGNA-------------------YLDGLGEAAWAPLRAYWAAPDETA-------------------VEAIRARMTLDGVKAAYVHGVADPSRIEPEAYTLDAAILARPGNADRQVTLKLDYKRNLERYPLIQAFFRARRPKLlvWGRNDPFFVPPGAEAFRRDIPEARVTFLDTGHFALETDGDAIAEEIRR------------------------------------------------------------    
 595 UniRef50_UPI0001BCD9D5 alpha/beta hydrolase fold p...   138     4e-31  1  12.7%  81:427     4:308     --------------------------------------------------------------------------------PEQSGAGRAGLDRESTRRSAKAGELTLNYYEAGApgGLPLVMLHGGGPGASAWSNFGPALpaEDFRTILVDQPGFGQSDKPPVVGNY-----FRFSATVLKDFLDELGIDRIHLLGNSLGGGTAMRFALMFPERVGRLVLMGPG--------------------------------------------GLSLNLFHADPTEGVKRLMDFSMAPSEDALRAFISTMV--VDQSLVTAELVAQRFADATAPGAQEALQSMGMSFWNPDSYEdlWRDAHQLRRPTLLTWGREDRVNPLDGALVALKMIPKATLHvpNCGHWAQIEAAEEFRQVCTAYLSRHVERPRGQGD----------------------------------------------    
 596 UniRef50_C5AL68        Predicted hydrolases or acy...   138     5e-31  1   9.7%  99:428    17:300     --------------------------------------------------------------------------------------------------WVDNDGLRLHYVIWGRDDAPTVVMLHGLRSYAQTWAPVADTLIDR-YRVVALDQRGRGLSDWDPGR-----DYHAAAYVRDLEALVQALDLRRFVLVGHSMGGANAFVYASARPERIAGLAIEDMGPGASAGSPGSARIKRELQDTPNAFASW--DEARAFWRRQRPRIAQAALDSRLVHSLKADARGRIVWRHDAEG---------------------------------------IAAARLAATPEQLVDLWPLIRDLRVPTLLLRGGDSDFLSAQVAAEMAAANACIERiiAGATHYVHDDQPAAFNRALRGWLDRLDDAAWRAGGR---------------------------------------------    
 597 UniRef50_B3T287        Putative alpha/beta hydrola...   138     5e-31  1  10.9%  94:415     6:277     ---------------------------------------------------------------------------------------------TPTDHNVEVNGLNIHYVDWGGDSNRNLLLVHGQGGNARNWDHIALALRDE-FRVVALDQRGHGESDH------TREGYAVTAFASDLAEFAEKIGITPYSYVGASLGARNGISYAGDHSDHLKHFVCLDY----------------------------GPEMSVASARNQISGMNRRALGWRSIDEYVDQQRINNPRLSDEYVRHQAQHALRLNYAGKY-VTKSDPDMF---------------WINGSFGVNEVPHLWEQWAKISCPILELKGGESDFLSPEIWDRMHETQPSLRFvvPESGHGITGDNPEFLLKELRGFF----------------------------------------------------------    
 598 UniRef50_A7EDZ1        Putative uncharacterized pr...   138     6e-31  1  11.8%  96:418    15:345     -----------------------------------------------------------------------------------------------TSTYIPTLNLTYHILRAGNPSHPLLLLLHGFPELAFSWRKLMPLLASSGYHVVAPDQRGCGRTTGWDTRAFSSVDLNSTTLVRLEYRVVIMSHPFKGTPILPTNTSTTNPTPASPTQDIHEALAHLPSPRKHYKWYYSTPTAATEMDNPKSELLPFLRGYFyhWTTNTPHPLTSWTASELQYLPPYYIMPLHSSMREVILQSMQNESSSLVQQSMNKWLTNEELSVYVDEYSRTGF-QGMLNWYRVITDPYWMKDTefAGRKIDVPLLFISGEKDWGMfpGVLEKMEDvfRGVK-VLEGAGHWVQQERAEEVAGIMERFLRGV-------------------------------------------------------    
 599 UniRef50_C1PHW8        Meta-cleavage compound hydr...   137     6e-31  1   9.4%  99:417    20:283     --------------------------------------------------------------------------------------------------FIDAAGVETHYLTCGDPSKPPIILIHGGGAGAtnWQGTFPLFTEAYY-VIAPEMLGFGQTGKPD-----DSDYSQDARVEHMKAFLDAMGLSKVPLVGNSMGGATSLGICMKWPELVSDLVLMGSAGLNVEITPSMM----------------------------------------PILGYDFTVEGMRKLIDALTGPLYSASDEIIQARYEGSIDPEARKAYTKTM-DWIKAEGGLAY------------TEDAIAQVKTRTLVVNGKDDIVVPMAYGIRFMELLENStyFMPHIGHWVMIEAPEEFAEIVMGFLAR--------------------------------------------------------    
 600 UniRef50_Q2JHQ0        Hydrolase, alpha/beta fold ...   137     6e-31  1  12.6%  71:420    35:357     ----------------------------------------------------------------------SSVDAPSSSPTGSDRDPLSALPRYGYSQTWHWRGWPIHFTYTPSRaaAPALLIHGFGASVGHWRHNILPLGSQR-SVYALDLLGFGKSAKPE------IAYSVDLWVEQVHEFWQTHIQQPSILVGHSIGGLVGVIAAARYPQMVKGLCLISCADGPHPEELPPPWDALVRALCEGIL---------------------SLLGCPLTYPHLFNWLRQTEVLRAWIKN---------VYKRDEQVDDELVQIFQRPAFEPGAAHVFLDGLRAILCRRFDSPKR-LLPTLKMPILLLWGREDPAVPSFLADQFKRWQPALTLvlPGVGHCAHDELPHWVNTLIGEWAASLET-----------------------------------------------------    
 601 UniRef50_B8Y557        Lipolytic enzyme n=4 Tax=Ba...   137     7e-31  1  11.1% 100:414     4:290     ---------------------------------------------------------------------------------------------------TNANGLQIEYDTFGKPGEPALLLIAGLANQLIHWdaLCQQLARRGHYVIRFDNRDAGLSTKLAEAGIPdpPYTIEDMANDAVGLLDALGIEAAHICGMSMGGMIAQTIALNHRQRVLSLISIYSHTGNPALPEPKPEALE-------ILFTRPPEERQANIEYALKVFRTLSGSGFPLDEEWH--------------------RNIAAQAYDRAFYPEGVA-------------------RQLAAVLTQKNRKSELGSVCVPTLVIHGADDLLVPVEGGKDTAAAVPEAELIIIDgghlPHAGAWPQIIDAIVGH-----------------------------------------------------------    
 602 UniRef50_B5I653        Alpha/beta hydrolase n=1 Ta...   137     7e-31  1   9.3%  49:413     1:342     ------------------------------------------------MGTGAAAVSLAGLQTNAAATTATTALDEPGGGPTPAVPSITPGTHTEFGthVKAGVLDIGYAEVGPQHGPVVVLLHGWPYDIHSFVDVAPLLADLGYRVLVPYLRGHGSTRFLSRHTPRTAEQS--AIALDIIAFMDALKIEKAVLAGFDWGSRTADIIAALWPERVKSLVSTSGYLITDRKMQLEP-AAAGVEHNWWYQWYFATDRGVKAMENVDERIALCRYVWTLVSPNWN-------------------------------FSDATFARTADAFRNPDYAAIVLYNYRWRISLIDGDRRYDRypSIGVPTVTLDAALDPFTAPGDGSGYRSHfpYLHRTIANIGHNLPQEAPTVFAQAVVD------------------------------------------------------------    
 603 UniRef50_D0NHJ3        Epoxide hydrolase, putative...   137     7e-31  1  10.2% 147:411    28:279     --------------------------------------------------------------------------------------------------------------------------------------------------VIAPDMRGFGRSSIPQTV----EGYGTKKVTGDLAGLLDFLNISRAVFVGHDWGAVITWRMCMFHPERVIAVCSVCTPYMAPSDVYVDTNTLVRAVPQFSYMSLLSQPVKAAKLLDV--APRRLFTATFRLYTDIDASLSFTELLRGVPESQNPIF-----TNPSKLLNLAELDYYVAEYERSGF-AGGCNYYAARFINFN--DERELPPSISHRALLISPAEDRVLKPEMAAGMRKLVPNLRQQIvnAGHWVLWEQKDEITRIL--------------------------------------------------------------    
 604 UniRef50_A3P4R2        Hydrolase, alpha/beta fold ...   137     7e-31  1   9.7%  62:413    26:330     -------------------------------------------------------------AGRSVCAAERAAGMRETRDRSRKSGEPTLDEARRVTSF-HHDGLTFDAVDTGPLDGEVIVLLHGWPQTAKCWARVAARLNADGYRTVAPNQRGY----SPLARPRRVGAYRMPHLVGDVVALIERLGGGPVHVVGHDWGAAVAWALAGRHPAAVRTLTTVSVP--------------------------------------HSGAFMRSMLSSDQLFRSYYMGLFQLPKLPELFVTRCRGL--FEKMLTGTGMTRDEVDTVYADIVEAGALTTSLNWYRAMMLTPPGYLT----RKVPVPVLHVWGARDAALSRRGAELAREFapYRLEVLPHATHWIPEHDAERLAALVRE------------------------------------------------------------    
 605 UniRef50_B1XNS1        Hydrolase, alpha/beta fold ...   137     8e-31  1  11.4% 100:415     8:276     ---------------------------------------------------------------------------------------------------ITVGNLTWFYRETEKQdrLPILFLHGLPAHSYGWRGVLERLGAANFHCIAPDWPGSGFSSYPSSR---EFSYRAEVFQGALKDFLAALKIERCHIVTQGFLGHVGVLFALNEPELCDRLVILNSPILPGSKLPWVMQQW------------------------------------------------GLPLAGEMLTQDPLLVDRTLEKGSGFVIGDSDLATYRKPYKTSSAvrALMTTVRRFELAKVLPDLGDRLKNRPAPTLFLWGTQDPWLDNDQIENWVKttAHRWEALPEAKHYPQEHFPEAIAPVLSEFL----------------------------------------------------------    
 606 UniRef50_C7Q0C0        Alpha/beta hydrolase fold p...   137     8e-31  1  11.7%  96:428    44:368     -----------------------------------------------------------------------------------------------THHHADVNGTTLHYVAAGT-SGSPILLVHGFPETWWAFRKLIPLLATE-HRVFAVDLRGFGDSGrsSDSSDEPSSYDSATSAEDLHQLIAHLDVGPVHLTGQDISGATVFRLATTHPQDVLSLTAIETGL--PGFGVEALADVTRGGAWYIGVLTTPGIAEMLLAGREREFFGQFLFPALCASPE--------------------------------AVSSEDIDEFVRTYSRPDGWRGATGLYRSMLREGAEIQALAQEPGLTMPVLAVGARGGAFTANTMSQTTSAEI--SSVSLDGVGHYVAMEAPDELAKALLEFTTTVDAAATPDAVE---------------------------------------------    
 607 UniRef50_C8XEE5        Alpha/beta hydrolase fold p...   137     8e-31  1  14.1%  94:433     2:296     ---------------------------------------------------------------------------------------------PEQTRFLELHGHRMALTDVGS--GPSILLVHGMMSARTTWADQWDRLAAD-HRVLAPDLFGHGESDKPLG------DYSLGAHAASLRDLLDALDVPSATVVGHSLGGGIAMQLAYLFPERVDRLVLVSSG--GLGRDLNPLLRAATLPGSELVLPVLASGWLHGVGDSALRLWRRVGLPAVSPSSTQAWQSLTSLADADTRRAFLATSRSVIDAGGQTVSARSRLSGLTARE-----------------------------------VLLIWGAGDRMIPSSHLEAARAELPHSRVEipRSGHFPHLDEPDRFAAVLADFVRAPDRPAGEITAPAGPSR----------------------------------------    
 608 UniRef50_C9SKM0        Epoxide hydrolase n=1 Tax=V...   137     9e-31  1  10.5%  96:419    33:352     -----------------------------------------------------------------------------------------------TRHYTRIGRNTYHYLLS-KPKGPPlvLLHGFPDLSYGWRYQIPSLTAAGYQVVAPDMLGYGRSSAPTNLA----RFTLKNMSDDLAALVRSirHEQVILGGHDWGGALVYRAALWHPTSTGASSACDAVLPADSNYVDSRQIAPASPPSFGFQRSSATRPWRQSARHQQDPTAPLALYGGRAPEGAFGF----------DGRTGLNFSNLDRLGASPLVKAAGLEYYVAEYAR-NTMRGPLSWFRTAEANFKGRSW-PLLRrfTMPGLYIGATMDTALRPSMSAGMERYFPeaRGEVV-SSHWALWQAAAGVNKIVGEWIAQLA------------------------------------------------------    
 609 UniRef50_C7QDV2        Alpha/beta hydrolase fold p...   137     9e-31  1  12.5% 105:417    14:281     --------------------------------------------------------------------------------------------------------VDIHYEDRGS--GRPVVLIHGWPLSVGQWEFQIPDLLAAGHRVVAYDCRGFGVSAKPRG------GYDPDTLAADLHMLLEHLDLREATLVGFSSGACTAVRYLSRFgqRVSRLALVS-----------------------------AAGPYLRRTDAHPEGFLEDTKLREYQRCLTDDRV-------SFLHRFATKFFEVARSSAARPVMPQPLHTRVVMLAAAASHRGAIQTLVQAV----TTDYRNDLRAITVPTLVVHGEADVVLPVEagARIAEAVADSRLVRMQGahGLIVTRAAEFNRELLAHLEG--------------------------------------------------------    
 610 UniRef50_B2IJM2        Alpha/beta hydrolase fold n...   137     1e-30  1  10.9%  51:418     5:330     --------------------------------------------------LLPIPFLLLGLPVSAEPSLPpgIGLEAVDYPYPVAFFDLTLEGQT---------LRMAYMDVPPAqgGTVVLLHGKSFSGDYWGPTIRFLTTKGYRVIVPDQLGFGKSAKP------DIRYSFDLLARATKQLLDHRGVTKAAILGHSFGGMLAVYFARDYPDLTAALLLENPIGLEDYRSAIP-----------------------------PQTLETLVQTEMAQTPESYRKFMQAfgWPGTVERQVEIFTRLLQSGEYPRAAKASALIYQMIYEQP---------------------IRHEYSLLHMPVLLTIGQGDRSvwPQLGRSALKDLPDGKLVEinAGHVPHVEQPEAFLTAIDAFLAEH-------------------------------------------------------    
 611 UniRef50_C8XJ50        Alpha/beta hydrolase fold p...   137     1e-30  1  13.9%  86:446    33:361     -------------------------------------------------------------------------------------PIPPLAQPAPAGRTHLILGGGVHVRRqgPAGAPPVWYVHGLGGSSTDWTRLSAALASRA-TGYSLDLPGSGRSDPPPGG-----RYSPQVDARVVAATIERVGDGPVHLVGNSYGGVVVTEVAAGWPHLVRTLTVLAPAVPDLRLTGDRGADPR----------------------------LGMLLMPGTAGLAYQRLGSIAPTARARGMGELCFGRPEVVTEQDYALAAREHAWRARLPWVYESMIGNLRGLMSSYLRTGQRSFAAVAGRITVPTLVVWGTRDRLVDVRLSRRAAAAYPDAqlVIAGCGHVPQMEEPETTARAMLAHWARTVTGSDFAPTEPDLAGAPSRSVPAAVGTS---------------------------    
 612 UniRef50_A8IIV7        Alpha/beta hydrolase n=2 Ta...   137     1e-30  1  11.8%  70:415     2:288     ---------------------------------------------------------------------RPCASAESAFLRPDKAIHRSGIQYPHKARAVSvaGEHFRFDVQGPEGAPPLVLAHSLGTTLELFDAVTPLLASR-FRVVRYDLRGHGESTAPDAVHG------MGDLGRDFINLVEGLKLGPVHFCGLSLGGMVGQWLAIHAPKRLKRIVLSNTTAypPRIWEARIKAVRRTGTDHIADAVIDSWFSSGVKARMPDTVARVREMIAATPAV--------------------------------------------------GYAATSCAMRDMDFRAALH-------KVTTPTLVIVSDEDRSTPPEWGEAVAQGIPGAQLARLPGGHLSCLEQPEAFAKTVLDFL----------------------------------------------------------    
 613 UniRef50_B4RH78        Hydrolase, alpha/beta fold ...   137     1e-30  1  10.5%  95:417    23:299     ----------------------------------------------------------------------------------------------PQDRYAANGAVKIHYVVEG--EGPLVVLLHGFPDYWATWKPLMAELNRAGWRTAALDLRGYNLSDKPDGVA----AYAMPNLIGDVAAVIAAEGAKDAVVIGHDWGAAIAWQVAMNRPDLVNRLVILSVP--------------------------------------HPAGFAREMATNAEQQksQYARNFQQPGFEKTLTAEGLA---------GWVKDAAEKPGYVEAFKRSDF-AAMLNYYRANYPSGTGAATAApaARIKAPTLVIHGMKDTALNAAGHAGTWDHvaDTTIVMFpaAHFVQHDAKDQVNRTVKGWLAA--------------------------------------------------------    
 614 UniRef50_B7S3G1        Hydrolase, alpha/beta fold ...   137     1e-30  1  11.3%  82:399     6:319     ---------------------------------------------------------------------------------TPDERFDKLPDYPFKPNYLEVDdlRIHYLDEGPADGEVVLLLHGQPAWSYLYRHMIPPLVEAGFRVIAPDLVGFGRSDKPTEI----EDYTYARHVAWMSDWLTQLDLSGITVFLQDWGSLIGLRLVAAFPERFAGVVLANGALPAGMVPEEFAALAKEAYKTLPVVKASELDACFR-------------GTYEGGAPGVPGFLYWRKFCAESEEIQRPGV-AIDSVGNASSLTDAEKEAFNAPYPDASYVAGARRFPSLVPVFHDEPEvwEVLKQFDKPFMCAFSDDDP-VSAGGDKPFIKKVPGCQGviSPAGHFL--------------------------------------------------------------------------    
 615 UniRef50_D2B3G9        Alpha/beta hydrolase fold p...   137     1e-30  1  10.3%  59:417    30:348     ----------------------------------------------------------IGAAACSTPTATSTPTATGVPPYAVATQDDPEFENTFRHEFADVDGVRMHYVTGGS--GTPVVLIHGWPQTWYGWWPIMPELA-KHHTVYAVDLPGLGDST------GSPTGYDKATLARYVHTLidRLNIRDTAVVGHDFGAAVAFQYASQFPADTARLGYLDLPLPGPAVDAAT-------------YRSLSWHIAFHSQRRVPEAV---------------VGDDVREYLALFYPQVAFGGTAFGGTSDRSPFTDTEINEYARTYRRPEALSGGFELYRALGKDVRDT---AAAAPVRVPTLLMTAQGQLDAIRATAAPRMTNIVRA-LDVPHAGHWLVEENPRFVTAELVRFLDG--------------------------------------------------------    
 616 UniRef50_A5FF96        Alpha/beta hydrolase fold p...   137     1e-30  1   9.1%  99:415    20:290     --------------------------------------------------------------------------------------------------YTTVNGVNLHYVTGG--QGSPLVLIPGWPQTWWSYRKIMPILAEK-HSLIVVDLRGMGSSEKPLD------GYTKKNMAQDIQLLIAHLGYKKINIAGHDIGAAVAFSYAANFPENTDKLIILDTPHPDENIYKLPMMPIGNPVFPWWLAFNQVKQLPEELLEGRYDILQNYIFNHLLIDPE--------------------------------AINEFDRKVYAEAYNNKDAIRSSNGWYQAFTEDIQD---IKQYKKVQIPA--------IGIGSQEGAKMLSYFFSVYleIENSGHFIHEEKPELTALLINNFL----------------------------------------------------------    
 617 UniRef50_Q2RZ52        3-oxoadipate enol-lactone h...   137     1e-30  1  11.2%  98:419     7:266     -------------------------------------------------------------------------------------------------PYDTVDDTRLHYTERGR--GAPLLFVHGLGSSSRDWAAQVDDFAKR-YRVLRVDLRGHGRSERGEGP------YSIAQFAREVAVLLRKHAHEPAHVVGLSMGGMVALELAASAPRLLRSLVVVNSVADMRLHSWRDV------------------------------------------------WFYVSRRLAVQALGMRRVGRLLARQLFVKPDQGALRRTLVERWAGTDK-----QAYLWSMDAIMRWSVADRLPRIDTPTLLVSSDED-YTPVAEKRKMKAQMPRAElvVHDARHALPVERPEAFNAVVDDFLRRVE------------------------------------------------------    
 618 UniRef50_Q4P052        Putative uncharacterized pr...   137     1e-30  1  11.0%  98:424    14:308     -------------------------------------------------------------------------------------------------RTEEGQTLRMHYVEALPanGELVLLIHGFPQTCYQYRHVLTPLAQAGFHVVAPDYRGAGRTSRPR------LGYDKATMAQDLLTLVREhfAAPVHLVGHDIGGMVAHAWAAQWPETLHSVVWGEACLPGSAFYE---RTKASIDKFHFIFHRLPDGLAERLVQGNEEAYLQ------------------------------QFFDRLIH--NTDAITADDLAEYVGAYSQPDAMRAAMDIYRALPEDARWneCKATLGKSKVPCLLLHGKQSDHDQEAMaqFYSQFSCDS---VSESGHYVAEENPTGFVNALLRfvEQVDSASAR-------------------------------------------------    
 619 UniRef50_A8LSD5        3-oxoadipate enol-lactonase...   137     1e-30  1  11.1% 101:424     5:267     ----------------------------------------------------------------------------------------------------TRDGVRLHYRDEGPRDGPVVVFANSLGTDLRLWEPILPL-LPPGLRIVRYDMRGHGQSDVPPAP------YTMGALVSDAEGLLDHLGIRDCVFVGLSIGGMVAQGLAVKRLDLVRAMVLSNTAA--------------RIGSPKLWQDRIDGVRAGGVAALTDAILERWFSRAFLTDPRLPFW-------------------------------------------RDMVLATPDEGYIGCSAAISGTDFYTPTSGLRLPTLGIAGSEDGSTPPDLVRETVNLIPGSRFVLlrAGHLPHVEQPEAYAEALTDFLRehLGAATP-------------------------------------------------    
 620 UniRef50_B7RTS1        Hydrolase, alpha/beta fold ...   137     1e-30  1  10.6%  97:417     2:266     ------------------------------------------------------------------------------------------------SHLAREDGKQVYYEDYGSGDS-AIVLVHGWGANTRAWDYTLPALVAAGHRVVLIDHRGCGQSSK------DFADVSIEAISGDVVALVEHLKLSRVVLNGWSLGGAVVVAAAVALGERCSGLVLTCGATPC--------------------------------------------YLQKPDYSHGGTDDALAETLAAMSADRVNFLSALAGGVCASDVSPQVVDWMVGMFMQSSPLAA------ASLGALGPLDQRADLAALEVPILSFVGAQDAVVDPAVCRSVADYAKDVSLVeaNSGHAPFVEESELYLRELNDFIAA--------------------------------------------------------    
 621 UniRef50_Q47C05        Alpha/beta hydrolase fold n...   137     1e-30  1   9.1% 101:417     7:292     ----------------------------------------------------------------------------------------------------TTDGLSLWSEAFGKPGDRPILLIMGAMNQGIFwdAFCARLAGLGYYVVRYDHRDTGLSSKIDYQAQP---YRLAELTQDALAALHGHNLSKATVVGLSMGGYIAQLLAIDHPEAVERLVLISSS---------------ADQRPYMAATMGQPAVGLTLPGPGPALLD------------YIQATIARPPQSLPEVEQNLLTGWAITYAGQREFPRAQMT--VALRQAAGRTDNPLAAFNHALAVAASPDRLESVKTIRAPTLVIHGSHDTCLPLAHGEYLARNIPGARLHILPMGHSFMWSWDDEVLSSLTSFLGE--------------------------------------------------------    
 622 UniRef50_Q218G8        Alpha/beta hydrolase fold n...   137     1e-30  1  14.6% 100:401    12:284     ---------------------------------------------------------------------------------------------------AKANGIEVCYEIFGATDAEPLLLIMGLGAQMILWDdfCRQLAHRGFRVIRFDNRDIGHSSKFSghIPIAAPYRLQDMANDVAGLLDALGIASAHIVGASMGGMIAQETAILHPERVRTLTSIMSTTGNPRLPYPTREAAAMLM-------------------------------------------APPPTTREAYIARFAQTWKVLRVGHFPQDEAKDRERAERSFARGLNPAGVARQLRAVLASG---NRRARLRQIKAPTLVIHGSVDPLVRPAAGKDTVAAIPGAKWLLIDggHALPM------------------------------------------------------------------------    
 623 UniRef50_A9F4A3        Putative hydrolase n=1 Tax=...   136     1e-30  1  14.3%  95:416     4:281     ----------------------------------------------------------------------------------------------PELVRFEIEGAEMAVRLAGDREKPAILLIHGFPSSSESFRNVIDTLARDCF-VITPDLPGFGGSEPVERP-------SFSRFADRIDGLLARLGAGSFHLYLHDYGTAVGLHLATRAPRRIRSLIVQNANAHESGMG--------------------------------PQWSATRAYWDDPTPEREAEATAHLTFEGTRDQYLGGVPRDIVE-----RIDPRLWEEDWRIMSLPGRlrALVLDYRSHVARFG--EIADYLDRWQPPALMLWGRHDIFFDLEETLSWMKALPRMEAHILDGPHFLLETHPAECAALMSAFVS---------------------------------------------------------    
 624 UniRef50_Q1AWZ8        Alpha/beta hydrolase fold n...   136     1e-30  1  12.3%  95:445    33:329     ----------------------------------------------------------------------------------------------PETEFLELEGTWVRYGVTG--GGPPVVLVHGWLSSSRIWDQLAGRLAQR-FTVYTLDLSGFGDSDKPLS------GYGIRYGSRLLYAFCAHFGLTHASVVGHDLGANMAIKLAADHPDVVGRLVVVAAPAQEDQIDLP-----------------------------------------------TPLWLATLPVVGPLFYMLGKVLRPVRVLWMRPFvdPDDLPEDVVEDAARSTPAAVSKTLRVARRELSGGRLARQARMVRVPALIVAGEEDQIVDPHSAAVWGRSLDQSEVCLMdcGHLPMVERVAEFNAQVLAFLTGDARYLDYVERFPGPPAGEEPTEPVEPVP----------------------------    
 625 UniRef50_D0MFD0        Alpha/beta hydrolase fold p...   136     1e-30  1   9.9% 105:417     9:265     --------------------------------------------------------------------------------------------------------VDLAYRSYGT-DGPPLLILHGLLGSSGNWHTLASKAFAPHFRVFTLDLRGHGRS-------PHAHPIDYPTMAADVLAFMDAHEIDRAHVLGHSMGGKVAMELALTAPERVDRLVVVDIAPRAYEPRHRVILD--------------------ALQAIDPARYDSRRAIDEALAAHVPEAPIRQFLLKNL-----------------------QYDPDTRRYTWQMDLEGLIRYYDRINEAI------ADGRRFTGPVLFVKGERSDYITDADLPAIRRLFPAARLviPGAGHWVHADAPEAFAREVLAFLTE--------------------------------------------------------    
 626 UniRef50_B5JX25        Hydrolase, alpha/beta fold ...   136     1e-30  1  12.1% 105:419    14:301     --------------------------------------------------------------------------------------------------------IQLYYECWGHSDHPALLMIMGLGAQMTVwqRLLEALVEAGHYVVLFDNRDIGLSTATPEVQSRtaPYNLSDMAGDAVAVLNHLGIEKAHVVGASMGGMIAQLLAVEHPSRVASIVGVMTSPNENDLPRPPLKLLMQLT-----------------------GLRGEPIVDADSAARNRIQLWRMINSPEFLTDEDVVYSRAA-------------ASYTRSFRPQGAAKHSMAVMATGG-------FEDRLKAVKVPSLFIHGSADRLVHSDGGRRSARAIPGAQFELIQggHEIPDALCEFVAAKIAQHIATIS------------------------------------------------------    
 627 UniRef50_C7NYU2        Alpha/beta hydrolase fold p...   136     2e-30  1  11.5%  98:418     2:267     -------------------------------------------------------------------------------------------------PTATHDGVTLAYERSGPADAETVAFVEGLSYGTWMWRWQREALS-ASYETLVWDNRGTGDSDEPPGP------YTTEQMAGDFEAVLDDAGVETAHVVGASLGGMIAQQYALDY-DRADSLTLIATTPGGEAAVPI-------------------PAATRQQMLNVPEEYDEAATIRHKMRPAVTDEFWEERS--------------------------DVVDRIVEWRLETDPSEAAYEWQSAAAVGFDESD---RLGEIDLPTLIVHGTADEVIPFENATLLDEAIPDSRLVAVDGghLCFIERADTVNEHLRSFLDDV-------------------------------------------------------    
 628 UniRef50_Q93S12        Putative epoxide hydrolase ...   136     2e-30  1  11.1%  98:413     4:277     -------------------------------------------------------------------------------------------------TRIGLDGLVFDVDVTGPEDGEPVLLLHGFPHNKESWTETAPLLHAAGLRTVAPDQRGY----SPDARPAAVADYRLPHLAADALGVLDALGVSSAHVVGHDWGAAVAWYLAARHPGRVRTLTALAIPHLDAYQHAYRVDEEQQHSSQYVEFLVSDGA------------------------------------ADHFLADGAAQLHAWFTQAGEGVLTDAQIARYVATHTAAGTLDAALNWYRANNLLGDPLDFGP----VTVPTTFVYSRTDTAVSELAVAKTEEYvpYRLLTLEKTSHWQPQQDPDTVAAEILA------------------------------------------------------------    
 629 UniRef50_Q2J8R5        Alpha/beta hydrolase fold n...   136     2e-30  1  10.5%  73:414     2:311     ------------------------------------------------------------------------VQEPTSTPSLGAPADPAPPIRGLRSEYARVGGHRLHYWTGGPDDGTPVLLWHGFLATGYAWRGVAPALADAGMRVLVIDMLGYGDSDKPGGT----DPYSARALAEQVRALTARSGfrPILHVGHDMGAPPALLWAASHPEETRGLVYAEAPTMLGGPLRDVLAYAPGTmsMWWWILPLAPGVPQALIVGREEAFLRWFYDGPAGAP--------------------------------RAFDEATVAEYLRTFSGPAGVLGSMGVYRAAFASIAQTEPL-LVHPTPVPVLAVGGERG--LGQQVGRMLSPVASRLSTSILdaGHFLPEEEPERLAAAITLF-----------------------------------------------------------    
 630 UniRef50_A5V0Q6        Alpha/beta hydrolase fold n...   136     2e-30  1  13.7% 104:416    19:279     -------------------------------------------------------------------------------------------------------DISYHVTETGAGD--PLVLLHGFTGSSESWAEVTDQLSHR-FRVIRIDLPGHGRTPAPPDPARC----TLPLVAGDIVARLRDLGATPAHLVGYSMGARLALGIALLYPYAVRTLILESGSPGLATEVERATRRALDEALATRIEQNGIVAFVEEWER--------------------------------------------LPLWESQRRLPEAVQQRLRAQRLRNTPAGLAASLRSMGTG-AQPSFWDDLPCLSIPTLLITGALDEKFTV-IARDMCTRCPVMthVIEGAGHAPHLECPSEFVKRVVEFID---------------------------------------------------------    
 631 UniRef50_B5WEV8        Alpha/beta hydrolase fold n...   136     2e-30  1  10.2%  84:416   239:509     -----------------------------------------------------------------------------------ASRLAAPAPFVPShrFLTVAGERIAYIDVG--KGPAVVLLHSLGTSSALWEDVLPTLTAK-HRVIAIDARGHGDSTK-------HSAWNADDVANDVVAVADAVGLGRFGLVGISMGGLTAIRVAAKLGQRVAVMVLSS-----------------------------------AYAGVSGPAVEKRLAAAEGMLKKLPLHLFARMYAEHTLHRDTAYAKR------------EKLAQQIASMSQRD--------YLEISRAVCTDDVSGLLHDIDVPTLVLNAELDGSVPKAISAKLTQAIQGATEQTLhaAHLACVDTPDAYAEALVNLFE---------------------------------------------------------    
 632 UniRef50_B7P6W6        Soluble epoxide hydrolase, ...   136     2e-30  1  15.7%  32:229     2:189     -------------------------------ACERLRRALYRALYI-VIGTCISLYVGIRVGIQVLIHGKAILQSKERKEEPACLRDPALGQ----HEFVDLEDVRLHYVSAGDRSNSLMLFLHGFPDFWFTWKEQILDF-KKDFWVVAPDMRGYGLSSKPAQV----EDYGMSYLIEDVRGLITTLGRKKAIVVGHDWGGFIAWVFASKYEHMVDRLVTLNSAHPLAM----------------------------------------------------------------------------------------------------------------------------------------------------------------------------------------------------------------------------------------------------    
 633 UniRef50_Q1LDN9        3-oxoadipate enol-lactonase...   136     2e-30  1   9.3% 105:416     7:256     --------------------------------------------------------------------------------------------------------RKLHYVIEGAADKPVLVMSNSLGTTLDMWGPQMAGLT-AHYRVLRYDTRGHGRSALPSS------SFGMRELAEDVIAIMDHAGIERAHFCGLSMGGMTGMYLARYHAGRFGRFVLANTAALIGPESVWN---------------------------TRIETVRR-----------DGMAAIVNGVLARWFTDHYVLTRRDEYEPVRRMLTGTSAEGYAA-----------------NCAAVRDADLRGLLPGIQAPVLVIAGEQDLATTAAQGQEVVRAIPGAEYLELAAAHLSNWELPEAFGNAVIGFLR---------------------------------------------------------    
 634 UniRef50_Q1AVN8        3-oxoadipate enol-lactonase...   136     2e-30  1  11.9% 105:422     3:259     --------------------------------------------------------------------------------------------------------VELHHVVEGPADAPVLVLSSSLGTTHRMWDPQVPALRER-FRLVRYDHRGHGSSPVPPGP------YSIAELGQDVLA----------------------------------------------------MLDGLGVERFSFCGLSLGGMVGMWVASEVPERVERLVLCCTSALLG-PRELWDERARVARSEGMEALVEGVVERWFTPALHQERPEDVERA--KRMLAATPPEGYAGCCEAIREMDLRDRLGRIQAPTLVISGSDDPATPPEHGERLREAIPEArvVIERAAHLANIERPEPFARALLEHLSPVLEGR---------------------------------------------------    
 635 UniRef50_A3TJP7        Probable hydrolase n=1 Tax=...   136     2e-30  1  11.4%  98:412     2:289     -------------------------------------------------------------------------------------------------TSAEIGDQRIEYVIDGPDDGEPILLIMGMGAQLVAWPpfVELLVAHGFRVIRYDNRDQGLSSFTDGPPPRRGdyTLVDLAGDAAGLLTHLGIDSAHVVGASMGGMIAQELALNHPDRVLSLCSIMSNTGHR-------------------------RFGRTSARLLPALVRQIRAVPPTTRDEALERSV-----------------DAFRVIAGSDFDDAEMRVMAEAHVARGAVTATSRD-RQLNAINASADRTSRLHRITVPTLVIHGLKDLLVLPSGGVATARAIPGSRLLmpEMGHDLPRGRRVEITDAIA-------------------------------------------------------------    
 636 UniRef50_A3TQ27        Putative hydrolase n=1 Tax=...   136     2e-30  1   9.8%  99:445    56:374     --------------------------------------------------------------------------------------------------VIASDGLNLHVEVDTPDDlpTIVMSHGYCLSLRGWVLQRRAFVQAGYRVVLWDQRSHGESEKSELAHC-----TIDQLANDLRAVIDATCPdpLVLVGHSMGGMTTMAFTRLHSELVRERVTAVAFVSTAAGGEG------------LTDLGMGPLVGRAIGQAGPRVLSRLAPHQKWLAPVRRIGRNVEDLLVDRFSFDSPVSEATVR------------------FAADLILATPFEVMAAFLPALRMHDEHETLAalDKQVLVINGTGDLLTPPQRSEEIVERLPGAevVVEESGHLIMLEHPEVVTGQILALIDRAEADAAAGIAVSDKPRVRRKVIDIAKGR----------------------------    
 637 UniRef50_B6IVM6        Hydrolase, alpha n=1 Tax=Rh...   136     2e-30  1  13.8%  85:418    39:323     ------------------------------------------------------------------------------------RADHDGSPGVRVHRVQGGGGIGLSVLEAGDPAGPPILFLHGFCQSALAWRPLFQGPLAARFRLLAMDLRGHGGSDRPDAAGAPGPYADGDLWADDVAGALAGLGMEKAVLVGWSYGGAVLADHLRRHgaRVAGIVLAGACPRLGAAARPFMGPLGRQHFPALMGGDFAAQVAACRALAHGMTVAPVAPEDLET------------GIAATMSVPPAVRRGMMR-------READFDPVLAAYDGPA--------------------------------LIVHGAEDRVVLPDLARHLAGLLPQARLHLYDggHAPFQERPDRFAADLAAFMDAL-------------------------------------------------------    
 638 UniRef50_D2BCL0        Alpha/beta hydrolase fold p...   136     2e-30  1  11.3% 100:418     4:283     ---------------------------------------------------------------------------------------------------IRIGGTEFGFDEAGS--GPAVVLVHAGCADRRMWDHQFRTLSER-YRVIRYDWRGYGESGDATGEFAHH---------EDLLALLDAFGVDRAVLVGSSDGGKISLDAALTAPERVTALTLVAPGLSGYEWPPSMPARYRERVHGVIGVDRLRSYRAGEVETVDAAELEAYSEAETEFLVAGPGRTRDDLAPQVWELALKMDRLLNERSWTGPHGSERDL----------------------------WPPAKGRLAEIEVPTLVVTGTADVPEILEVSDLLAREIRKARRVepETGHLPPLERPAEFTAALLDFLATL-------------------------------------------------------    
 639 UniRef50_C3JVR6        Hydrolase, alpha/beta fold ...   136     2e-30  1  11.9%  67:418    12:333     ------------------------------------------------------------------CVTTTTPRCILAGMESAVSAQLVDGSTLTHPWGGTSngGDVHWIDFGGPSgpPIVLVHGLGGSHLNWVRVAPALAART-RVYALDLAGFGLTSARGRHTGVDANTVL------LNRFLDTVVGEPAVLFGNSMGGMVSAMSTHASPESVAGLVLVDPALPLPVQLPDLTVAAQFALYSMPYVGEQVLGFGRRKMSDRQLAAQMTRLCFADPTRADPAVLDAGAALTAIRRNEPTQDAEFLQAARSLLMTLARPASY-----------------------------RKTLREITTPTLLMHGDRDRLVPVEAARAAAAAHPHWTTIIldTGHTPQLEIPDEFTRHALAWLDRT-------------------------------------------------------    
 640 UniRef50_D2SAY0        Alpha/beta hydrolase fold p...   136     2e-30  1  10.7%  92:417     5:294     -------------------------------------------------------------------------------------------PGVAETRQDGADGVRLRVRVGGA--GPPVVLLHGHPRTHTTWHRVAPLLAAAGHTVVCPDLRGYGRSSEPP-TTEDHAPYAKRAMARDVVALMRGLGHERFAVVGHDRGSYVALRTALDHPGAVTHLAVLDCIPIVEHLDRVDARFAAAWWHWFFFAQPEKPERA--------------------------------------IGADPDAWYGALAPGKAAQMGEENHADFRRAIADPATVHAMLEDYRAGLtrAAEEADRAAGRRVACPTLVLWSAHDDLEDLHglRIWAAWTGDLrgGRIDSGHHMAEEAPEELVAALVPFVAG--------------------------------------------------------    
 641 UniRef50_B9NWP7        Haloacetate dehalogenase H-...   136     2e-30  1   8.6% 114:416    32:301     -----------------------------------------------------------------------------------------------------------------AGSGPALLMLHGDPQTHLCWHHIAPKLSER-FSVVLTDIRGRGETHKPDRDAREN-AYTKRAMAEEQLAVMQALGHDTFAIVSHDRGARVARRLTLDHPETVTQLVVMDI--IPALDFYEALNAEIAQDYFYFSFLTQDPPVPERLIAGDREGFLRLILEGLSDKSVQYDQMA--------------------------------MAAYLKANATPEAISAMCECFRAGIHIDRLHDarRSGRQITCPTLIMWGDKGVVGKHFDVErwHGWCSDPSFASMPSGHFIPEEAPGAALAAIQNFLK---------------------------------------------------------    
 642 UniRef50_A7IG07        Alpha/beta hydrolase fold n...   136     2e-30  1   9.4%  67:418     1:320     ------------------------------------------------------------------MRTSPSPESRWRRSRPSAPRGPAEapDAPASHRIQAGGLDVHYLRAGR--GRPLVLLHGWPEFSGVWEPLMARLAPD-FDVIAPDLRGFGDTQKPDAGPS--EKVSADLLAADLIALLDGLGLepVGLVSHDVGAVVGQSLGRSHPERFRGLFFFNCPY---------------------------PGIGRRWAE--PDHIREIWYHSFHCLPW-----AAAMVGSSREACRQYFSHFLRHWSADPHTFDDVLERWVDNFMKPGNLQGGFNWYLSMRAGRLALMKGEavAPITVPTRVFWGDRDPVLRSEWMDRLPEYFLDLEASVAEggHFVHYEDPDRAAAEVRAFFHRV-------------------------------------------------------    
 643 UniRef50_A8ZZL4        Alpha/beta hydrolase fold n...   136     2e-30  1  12.2%  90:418    21:290     -----------------------------------------------------------------------------------------AEAGLERKTITLPSGETYVYLEGGT--GEPVLLLHGFGANKDNFTLVAKYLTPR-YHVVAPDHIGFGESDRPAGA-----DYTPAAQAVRLRGFVRALGLSKIHIGGSSMGGHIAMTYAALWPDEVKSMWLLDPGGVWSAPESEMRQIIRETGRNPLIAKTPEEFV----------KIFDFVMTDPPFIPTPILHVMAKERVENVGLEEKIFIQ------------------------------------------LTGDSVERRIQGLAVPALIVWGDQDRAIRVESAGILHGLLPVSEVIIMKggHLPMLEAPKQAAHDYMAFLDRL-------------------------------------------------------    
 644 UniRef50_UPI000023D83C hypothetical protein FG0574...   136     2e-30  1  10.5%  88:412     1:296     ---------------------------------------------------------------------------------------MSTTRRNTKQNTPLTRTLEVGYYDHGPPSGWLVLLYHGFPYDIHAYDDVVPKLVSNGARVIVPYLRGFGPTRFLSSQTMRSGQ--QAALGSDVIELMDALNIEKAILGGFDWGGMSCCVASALWPERVIGLVSYASYDIADISASQIPLEPSLECTVWYQHLFQQERGIKCLAENRRDLCRILW-----------------EQWSPSFSFTESFYQRTAESFDNHDFVDVVIHAYRFCFGNAKGDPALQR----------LEDVLATKPKITVPTVALDGKRDPLKKGGTAAHRQYFTGQFERRVADVGHAFPMEAPDEFADAIL-------------------------------------------------------------    
 645 UniRef50_Q7NGS9        Glr2809 protein n=1 Tax=Glo...   136     2e-30  1  13.4%  96:416     4:283     -----------------------------------------------------------------------------------------------RESTVRAGGRQWFYRSAGSESGPPVLLLHGLPSHSHGWREAVPVLAERGLWVIAADWVGFGGSDKPDKSA---FNYTPDAFIAALAAFVDALALERFSLVVQGFLGSAGIQYALREPRRIARLAVINAP------------------------------------------------IATSAKLPWNLRQLGLPLVGEMLTQNPLSVDQTLEGGGYTVISNGDLGAFRRPYSESgaGRALMLTVRNLDLPRAMAEIEAGLTPWrvPtaLIWGLRDRYLGEAMAKSFVAAHPQVEFfvAQAGHYPLEQDPAAVNQALVRFVA---------------------------------------------------------    
 646 UniRef50_A7HTR3        Alpha/beta hydrolase fold n...   136     2e-30  1  13.5% 100:446     4:317     ---------------------------------------------------------------------------------------------------AQANGIELEYESFGSEDDETILLIMGLGAQLTQwvELCEALVARGYRVIRFDNRDTGLSSRIErgKPAFVAYSLSDMAADAVGLLDALGIEKAHIAGASMGGMIAQHVAAEYPERTHSLTSIMSSSGNPMLP--------------------------------PAKPRVLGLFLSPSPAPHDTDAIVARGVRTYETIGSPGFPTDEVTLRDWVMRDATRAYY------------PAGVVRQMAAVMTDGDRRPKLRAIRAPAVVLHGADDPLVPVAAGVDTAENIPWADLRIVEggHDMPLALLPAIADAI------VSAAERATGVKTIAPEERIELAPALTGPP---------------------------    
 647 UniRef50_A5FVX7        Alpha/beta hydrolase fold n...   136     2e-30  1   7.4%  95:417     8:297     ----------------------------------------------------------------------------------------------FSESTADLGDGR-HFRLRSGGSGPPLVMLHGMPQTHCMWHRIAPALAAR-FTVICPDITGYGNSYKPAASADH-EAYSKRRMAQDIVDLAAHLGIGRFALVGHDRGARVSHRLALDHPDRISRLALLDIIPTIEHFERTDMRFAMGYYHWFFLAQPAPLP----------------------------------EDLINRDAGYWLLSQTARHKTARAVFDEAAMADYRRAMADPATVTAICEDYRAAATIDLEHDRESRArrIAAPLKVLWGSRGLvaLYDPLEIWRGYASgvEGQAIESGHFLAEEAPEATLAALQEFLAD--------------------------------------------------------    
 648 UniRef50_C2CR97        Hydrolase n=3 Tax=Corynebac...   135     2e-30  1  15.2%  78:419     2:306     -----------------------------------------------------------------------------AQTPAPLPPSVVELEGPFTHEFVHTRGIRLHAATAGSPSDPLVLLLHGSFGGWFDFKDVIAPIAEQGFHVAALDMRGFGMSDKPPVEVGQD----IRAMVGDITGAIQALGHDSAYLVGADSGGAIAWAVACERPERTLGLVSVSASHPVDLRRAMAARPWDFGWLLLRAGLCRLAG----------------------------------PIRRHNPLMRPSTYEKELALNTDSKFPAedVAKLRIQASQIGNVRRGILWNHRMRTAVPPLSWID--SKVEAPVLYLHPKQQLWRprRAEKRTASAFRNAT--IAQTKNLPHIESPEAFATMVSEWLNSHD------------------------------------------------------    
 649 UniRef50_C1XNY7        Predicted hydrolase or acyl...   135     2e-30  1   9.5%  91:417     2:267     ------------------------------------------------------------------------------------------PQERPIEGFFSRGSAQIYYQSYG--QGVPVVFLHGFGLDSRMWEPQLEPFAQA-FRVIRYDLRGYGRSSLPAEPY---------AQTDDLLWLLRYLDAIPAHLVGLSMGGQFALRFALTYPQAVRSLVLAGSALDGYRWSVQWQERWQGILSVARAGDLEEAKRRWLSHPLFAPALEQPLVAASLATQINGYSGWHWINRDPDQPPNPPVLDRLEQVVQP--------------------------------------------------ALVVVGERDLADFQTIAHTLASRLPHSrvVIPKAGHMVNLEAPDRFNQEVLEFLSR--------------------------------------------------------    
 650 UniRef50_B8EQ01        Magnesium chelatase accesso...   135     2e-30  1  13.2%  84:469     7:349     -----------------------------------------------------------------------------------WDKDGADWPNRATSRFVTAGELRWHVQIMG--DGPVLLLLHGAGSSTHSWRDLAPLLARR-FTIVAPDLPGHGFTQAPR-----PRDMSTDGMAKAVSSLVAALDVDPTYAAGHSAGAAILCRMRLERAIEPRLLFSLNGALLPFDGFAGRVFSPLAKMMF-------------------------------------KNTLIPAPRLFAWLASDRSSVERTLRNV-GAHLDRRGVEFYARLFRNAGHVGATLGMMAH----WDLKSLRRDLPRLDSHLVLVATEDDRAIPVRDAHAAAGLVPGSRKIIlrGGHLLHEERPEEAFDLIVAAiaSLAPSVFPPA--PPARHVGGVGKSArqKNPHPAPPQAGQGGAQCEDQAKAGQE----    
 651 UniRef50_C1XWN5        Predicted hydrolase or acyl...   135     2e-30  1  11.6% 100:418     7:264     ---------------------------------------------------------------------------------------------------VLLGDLRIAYAEAGY--GPPLVLLHGGMDDSRSWRRQMDALSDE-FTVLAWDAPGCGRSS------DVSQSWRMPNYADALAGWLVALGVERPHILGLSWGSSVALEFYRRHSHVPASLIL--------------------------------AGAYAGWAGSLPPEEVASRLESVLAAADLPREQLLTGLPGVLSSAAPA----------------ELVDELSAIWADNAGRSHP-EGYRAMAES----DLRDVLPGIHLRTLLVYGELDQRAPLQVANDLHQRIPRAKLVVIPggHLANVEAPDEFNAQVRLFIRSV-------------------------------------------------------    
 652 UniRef50_D1V634        Alpha/beta hydrolase fold p...   135     2e-30  1  13.4%  98:417    15:280     -------------------------------------------------------------------------------------------------RHVDVGPWSTRVLEHGA--GTPFILMHGTGGHLEAFTRNLRAL-GAHYRLIAYDYPGHGWTT------TTTRDLEIDDYSEHLVGLMDTLGIEKAHLSGESLGGWVAVKFAARYPERVDRLVLNTPGGTMATPEVM-------------------------------ERIRSL-----------------SQAAADDPSEERIRTRLEWLMADSSSVTDELVAIRRGVYSRPGFAASMRHILCLQDPEVRRRNldEELAAITAPTLVIWTSDDPSGPAKAGLDMAERIPAGEFRLieAGHWPQWEQQEEFDGIALEFLGR--------------------------------------------------------    
 653 UniRef50_A6GEU6        Probable epoxide hydrolase ...   135     2e-30  1  18.4%  83:419     2:310     ----------------------------------------------------------------------------------SLAEWKARGRELELEGF----GRRLFVVERGpdEAPTLLLVHGFPTSSHDFHRVLDRLA-QTHRVIAHDQLGFGLSDKPrgGGEGEYGYSLFEQAEVALALWRQLGVRRARLLAHDYGTSVVTEILAR---RAFSPVAGAPELDAVILCNGSVNLELASLTWPQKLLAGPLPVARAFARVGPRAL------------------------------FEARIRKTLAPASRARFDPADIEAMWAGNVRAGGRERLADLSRYLHERRrwARWREGLRRTQVPALVLWGRADPIALPAIAECLAAELPRAQLRWLEggHFPMLEDPGRWAEALLEFLDGLD------------------------------------------------------    
 654 UniRef50_Q026V3        Alpha/beta hydrolase fold n...   135     2e-30  1  10.5% 100:431     4:284     ---------------------------------------------------------------------------------------------------AQANGVQLHYQQFG--EGPPVVMVHGITGNLAIWheIVPGLMSD-FRITTYDLRGHGYSDVPPT------GYTTADHAMDLKHLLETLGIERAHVMGHSFGADIALHFTILFPERVDRLVLVEPGI-AALTPLRESEDWVGWKYWRDKLALGGVVIPPEKWYDAEYLVRASVGLPMLFGFRRGRARRAAPLVRLMDTTTAAKDYCDLAGMTLDKID-----------------------------------------QIRHQTLVLYGEDSVFLG--TYEYLRDHLVNSvfLMPDSEHFGPIERPEILLQHVRTFLSEAEKPAQARTAASSG------------------------------------------    
 655 UniRef50_A3VDE0        Alpha/beta hydrolase fold p...   135     3e-30  1  10.3%  84:413    29:314     -----------------------------------------------------------------------------------VLAAQAEAAVPPVGKFLTISTGRLHYTDTG-GDGRPVILIHGLGGQLRSLTMTLAEPLSHDFRVIALDRPGMGYSDRPESASA-----RIDDQAGYVEELIDALGLEKPILMGHSLGGAISCATALRAPDKIGGLALIAPLLRPSE---------------------MQAKSFAALGITNPRMRRFVAHTVAVPTSAKNADLTRTEIFGPDPVPDGYTVKGGGLLS-----------------LRPKSFINTSRDYCAVGEVIVKQ--WKRYDEITCPVRVLYGTEDRILdrKQGVEMADRYPHFHllIEGAGHMLPLTQVDRTVETIRA------------------------------------------------------------    
 656 UniRef50_UPI0001B54DB6 hydrolase n=1 Tax=Streptomy...   135     3e-30  1  11.0% 111:419    15:269     --------------------------------------------------------------------------------------------------------------DFGPRGAEAVLLVHGHPFNRSIWAPQATALAAAGYRVIAPDLRGYGE------NPAVGERTLLADFADDLAALLRHLEIERAVVGGVSMGGQIALELRLRHPGLVRALVLSDTSPVPETEDGKEFRRKLAE----------------------------------------------RLLAEGMRPYTEEVIDKMLAPYNVTGQPEA--AARVTAMMCATDPGGAAAALRGRAERPDYRPVLRALPE-EVPCLVLVGEDDVYTPVAEAEAMCALAPHARlvIERAGHLPGVEQPEAFNRALLEFLAVLP------------------------------------------------------    
 657 UniRef50_D0WAK0        Proline iminopeptidase n=1 ...   135     3e-30  1  10.0% 105:413    56:341     --------------------------------------------------------------------------------------------------------HQIYWEESGNPDGVPVIFLHGGPGAGAS-PECRGFFNPDVFRIVIIDQRGCGRS----RPYACAEDNTTWDLVADIEKVREMLGIEKWLVFGGSWGSTLSLAYAQTHPERVKGLVLRGIFLCRPSETAWLNEAGGVSRIYPEQWQKFVAPIAENRRNRLIEAYHGLLFHQ------------------DEEVCLSAAKAWADWESYLIRFEPEDVDEDAYASLSIARLEN--HYFVNGGWLQDDKAILNNIGKIriPTVIVQGRYDLCTPMQSAWELSKAFPEAELRVVQAGHcpPL--ADALVQAVED------------------------------------------------------------    
 658 UniRef50_C7JDV2        Esterase/lipase n=12 Tax=Ac...   135     3e-30  1  11.1%  79:421   101:378     ------------------------------------------------------------------------------PQDAAATEDANAGEPKP----VTVGEHTLNVRDVGTHTGTPIVLVHGFGGDISNWLLTQDALAADR-RVIAFDLPGHGASSKNVGTG------TLAFLAGVVSELLKTLKIEKAHVVGHSLGGGIALTLLRDHPDQVASLNLLAPAGLGKDVNSDFISAFVDS----------------ESSRDMKAVLQMLVHNKALVGRKMVDAVLRARRLD-------------------------------------GARDALHVIAKACFPnhQADDLRPVLAGAETPTQIFWGKEDEILSVSNASGLPDVIP--VTVYEETGHLPQLERATDVNKAIAVFVKDPEAA----------------------------------------------------    
 659 UniRef50_Q0VPA9        Hydrolase n=2 Tax=Alcanivor...   135     3e-30  1  10.9% 114:415    19:286     -----------------------------------------------------------------------------------------------------------------AGNGEPVILLHGFPDTYENWAVQINALADAGYTAIAPALRGY-----SPGCQPGNGDCSLYAAVDDLMVFAAQLG-GRVHLIGHDWGAVVGYLACAQSPETFCSFSALAIPPVRRIPQ---------------------------ALLKVPEQVLLSGYMQFFQLPLAPEAWLQRGDLD--------GVTTLWQRWSPDWEPGLYLDNAKHTLAEPGVIPAALSWYRSLFKVweHRQVRGWLGliATPTQILIGENDGCMSPKLLEHTldFSGGVElvLPNAGHFLHLEQPEAVNHHLIRLL----------------------------------------------------------    
 660 UniRef50_C1YUY2        3-oxoadipate enol-lactonase...   135     3e-30  1   8.7% 105:425     3:263     --------------------------------------------------------------------------------------------------------VDLHHTESGPADAPPLLLGGSLGTTTRMWDPQVEALST-VFRVIRFDHRGHGDSPVPEGP------YSMADLGGDVIALMDRLGIERAHYAGLSIGGMVGQWLAVHHPGRIDRLALLATSPHMGP------------------------------ARNWRDRAALVRAEGTGAVADSVVERWFTPAFAEDRPAEVAVLRDQVAATAP-------------------------EGYAGCCEAIETHDVREDLHRISAPTLVVAGADDPSTPPAgaELIAERVPGARLVVleAAHLLSWQRSAEVNTLLAQHFASADRHGAGT------------------------------------------------    
 661 UniRef50_Q1B014        Alpha/beta hydrolase fold n...   135     3e-30  1  11.7% 101:425     9:290     ----------------------------------------------------------------------------------------------------RAGGVGVRCLAAGEGE-PAAVLLHGASLDCasFGHLIGPLSGRR-RVYAPDWPGYGGSERPPEAA-----YDLAYYERFLERLLDALGLERADLVGLSLGGGVALSLALREPRRVRRLVLAGSYGLGRRVPWGPLGAALGR-------SALAARLAYGLMRRSRPALRLGLRN---------------------------------VVCDPAAVTEELLEELARQAALPGAGRAFLAFRRsvGWRGLRSDLSGDLHRLAVPTLLVHGSRDRIVPAGWAVEAHRRIPRSELLIlgCGHWVPRERPEEFSRAVERFLSRPDPAGRPR------------------------------------------------    
 662 UniRef50_Q9HZR3        CFTR inhibitory factor, Cif...   135     3e-30  1  10.1%  83:417    22:317     ----------------------------------------------------------------------------------GFAAEEFPVPNGFESAYREVDGVKLHYVKGG--QGPLVMLVHGFGQTWYEWHQLMPELAKR-FTVIAPDLPGLGQSEPPKT------GYSGEQVAVYLHKLARQFSPDRPflVAHDIGIWNTYPMVVKNQADIARLVYMEAPI---PDARIYRFPAFTAQGESLVWHFSFFAADDRLAETLIAGKERFFLEHFIKS----------------------------HSSNTEVFSERLLDLYARSYAKPHSLNASFEYYRALNESVRQNAELAK-TRLQMPTMTLAGGGHGGMGTFQLEQMKAYADDVehVLPGCGHWLPEECAAPMNRLVIDFLSR--------------------------------------------------------    
 663 UniRef50_B2GHE7        Putative hydrolase n=1 Tax=...   135     4e-30  1  10.7%  92:418     3:270     -------------------------------------------------------------------------------------------RSAFITHTAHVNDTELAYTDEGR--GEPVVLLHGHAYDRSMWDGQVATLTEQAWRVIAPDLRGFGESQVTEGIV-----YT-EEFAADIVALLDELVLETVILLGFSMSGQVAMQIAASYPERVGALIINDTVPTSEDAAG-----------------------------------RRRRHVAADGIMRDGMGTYAQNVLSKMIAEENVERLPEVAECVREMIRTAPLEGSTAAMRGRAERNDFTETLRAWEK----------------PALVIVGDSD-AFDNGAGQAMRELLRHGELAVVPagHTPNMENPAEYDAALGAFLSRL-------------------------------------------------------    
 664 UniRef50_C7P252        Alpha/beta hydrolase fold p...   135     4e-30  1  12.2%  94:413     1:288     ---------------------------------------------------------------------------------------------MPITETVTADGLAIHFLEAGDPAAPTIVLLHGgdAARVSWGEVIEPLAADCH-VVAPDLLGYGRSGVDSEGTdpPGSYPVGRHVDAMTGFLDELAVDTFSIAGLSLGGAVGLGLALRRPALVDDLLLIDSYGLGRALPNGRLSYGLARVQ-----------LFNRIAIALFRRSRRLTRASLGGI-----------------------------VADLDGLSEAAVDAVYEEVQRPTAGVAFRRFREAEVTRSGYRTvvDELPELAVPTRLLHGRHDEVVPLSWAERAADRIPDGELVVLdcAHWPPREAPSTVVEHARE------------------------------------------------------------    
 665 UniRef50_P26174        Magnesium-chelatase 30 kDa ...   135     4e-30  1  13.2%  84:417     3:284     -----------------------------------------------------------------------------------PRALPANWPYRSAAARRRVGPIDWWVIDTGPADGPVLLLLHGLGASGHSFRKMIPGLSAR-YRVIVPDLPGHGCS-----RSTARNRFGLKPMAEDLWKLCQHLNVTPAAVIGHSAGGAIALQLALDTPV--------------------------------------PRVVGINAALDHFEGVAGVVF------PMMARGLAALPFTAPLVTRFGASRQRIGQLLDMTgiDAAGKAYYTALIQTPEHVDGGLRMMAQ----WELGPLIGALPRIAKPVFLIAGNGDRAVPAHVSADAARFLPMATLRRIDGGHLIHEVAADGLSGMILDWLEG--------------------------------------------------------    
 666 UniRef50_B2GL34        Putative hydrolase n=1 Tax=...   135     4e-30  1  13.0%  99:421     3:273     --------------------------------------------------------------------------------------------------TQTWNG--LNWRETG--EGTPVLLIHGIGRSLADWDEQHERLAAQ-HRVISVDLPGFGGSEVPT------EGMSLPNLASRVAEFVRAQGIASAHVAGNSLGGAVAMQLTAEHPELVRSLLLVDSA-----------------------GFGRGVSPALRLATLPGVGERALT-----------------------PSRKGALRSERAMYHDPALATPERVERGYELAQRPGGSAAMLQMLRSIGsrSWRRRLLPRVRATGVPVFIVWGDKDTVLPFRHLARGAKALDASTHRFPDTGHGPMIERPDEFARLATEFWADVDRA----------------------------------------------------    
 667 UniRef50_A9WBN1        Alpha/beta hydrolase fold-c...   135     5e-30  1   9.5% 100:427     4:267     ---------------------------------------------------------------------------------------------------IVIDNQRVHYEVFGR--GKPVVFLHGWLGSWRYWYTSMEI-VAQHFRTYSFDFWGFGESRTNEMP-------TIQGYSNQVIRFLDAMGIDRAALVGHSMGGMVALKTALDHPGRVVRVATVGAPINGNSL---------------------------------------SWMLKLVDRPFFAEFFARRPWLR----------RSLFRFFFGDSLDPEVDEVF------EDSTKSTADTIRSAIHSMWRTDLTPLLDRLQVPALVVHGARDEIVNPNQLQLFV-HVPMAQVvmERSRHFPFVDEAEQFHNILLDFLKQPDpaPTRKAA----------------------------------------------    
 668 UniRef50_C2AQS9        Predicted hydrolase or acyl...   134     5e-30  1  11.4% 112:419    19:278     ---------------------------------------------------------------------------------------------------------------AGPTDGRPVLFLHGFPEAAVEWEYQLEALGDRGFRAVAPDQRGY----SPGVRPERPAEYAMTALVGDVIAVADALGWPTFDLVGHDWGGAVAWWTADEHPDRLRSLTVVSTP--------------------------------------HPAALAESLRTDEEQRASS-QYMLRWRETPATENAMLADEGRALRLMFDGAVPRGTVDDYIQRLREPGALTAALNWYRSGRPAT-------AIGSVTVPTLYIWPTDDAAFRSTVAFATQRFcpYRFEMLEDVTHWAPEQVPEAITGMIVEQLEATR------------------------------------------------------    
 669 UniRef50_Q7V4K8        Possible alpha/beta hydrola...   134     5e-30  1  11.9% 104:421    17:319     -------------------------------------------------------------------------------------------------------GHDVHcvEHGGRQRPALLLVHGFGASTDHWRHNIPKLS-LTHEVHAIDLLGFGRSAKPGGL-----DYGGDLWKDQLVAYVNERIGRPTVIVGNSLGGYAALAAAAALESQSAGVVLLNAAGYFSDEKFSPKPKDMSSRLRQMIGQGLSRDLVVKW------------LIYPLIQRMIFENLRRPGVIRRTLQQ---------VYIDPANVDDDLIESIRRPSLDPGAFQVFRNVFQA--RGLRGKAIDELFNDLQAPLLLLWGDGDPWLRNAKAKqfRTYAREASlvLLNAGHCPHDEVPDLVNSELLEWLNGLSIA----------------------------------------------------    
 670 UniRef50_Q0B7D0        Short-chain dehydrogenase/r...   134     5e-30  1   9.8%  81:456    26:361     --------------------------------------------------------------------------------PLSDEAPLALFELLHSETAVASGDLTLAVRTWGDPARSPVVLVHGYPDDSSVWQHVAPLLARKHY-VIAYDVRGAGLSGVPKRTA----DYHLAKLTDDFIAVIDTLCPGRahLIAHDWGAIQGWEFVTDPRLAGRIASYTSCSGPCLDHVGFWLRERV-----------------------LRPTPASLAKLGGQLVRSWYVVLFHLPFIPELSWRLWLGRAWPRLLRRIEKTPAAPRATQADD---------GAHGVRLYRANFIRCLFAPRERYAHAPVQTIVPLGDKYVSPALSENLSRWVPQYYRREVAAGHWLPLADPARFAGLAEQLIDAVESGDEPPALANARRRATS----GRFSGKVVVVTGAGSG-----------------    
 671 UniRef50_Q1J1Y8        Alpha/beta hydrolase fold n...   134     5e-30  1  11.9%  91:417     1:305     ------------------------------------------------------------------------------------------MSTPDESNFERLNGADLYFEVTGPQdePPIVFLHGGPgnSYSFRELFGD-RLQGRRVVYLDQRGSGRSGPLQDTEQGGETLDLDTLVADVEALREHLGAEQIVPLGHGFGALVALEYARRHPTRTARVIVVNPWVHFPELALTLLREA--------------------------AALRGVPLDDPAQAVRARTPEGQYPAVGEARVEAAFALLNARDLLNTLQFRDNASRMRLEFIDAEGQLSGGGEVQEALVNQGLWeeYPPFLQELRRPLFVIAGVHDRTSYPEQVEWLVD-LGGADVTVLDAGHYPWLDDEDAFAEALEEALTR--------------------------------------------------------    
 672 UniRef50_A8LGN7        Alpha/beta hydrolase fold n...   134     5e-30  1  10.6%  99:418     3:271     --------------------------------------------------------------------------------------------------TIDVRGVSIAYGdaGRPDAPTIVFGHGLLFGGWVFRAQVAALRE-HYRCVTLDWRGQGDT------PPTPGGYDMDTLTADAVAVIQRLGLAPVHWVGLSMGGFVGLRIAARHGELLRSLTLLDSSADAEEPTEAGAYRRLA-------------------------------------------------LVARLVGVRPVLGRVRPTMFGPAFLADPDGRPVVAEWAGRLDRVDRSALRKAVLGVADRPPVDHEIPGIAVPTLVVVGADDVATPPERAERMAARIPGARlvVADCGHTSPLEQPDVITGLLRGFVAGA-------------------------------------------------------    
 673 UniRef50_A9VYS6        Alpha/beta hydrolase fold n...   134     5e-30  1   9.4%  78:418    31:343     -----------------------------------------------------------------------------AEEPSPLGIGLEGFAYPFPVRFlsRDgqRLAYMDVPAAdgRTVLLLHgfPSS--YWEPVIRTLSNAGYRVVVPDQLGFGKSSKPIG------SYTFDRMAADTLALADSLKIQRFDIVAHSMGGMLAVRMARNYPQRVNSLVLEAPIGLEDYRFTVPPVS-------------------------DEALIAREADLTADAYRKQLMTSYALSIPASAIEPFVSIRERVKGSGEYPRWLKSFVNSYQMIWGQPIVHEIPL---------------------VKAPTLFIMGANDHNAptGHARALAGRMPNGKAEVlnVGHLVHMEATEMFNTLTLEFLNTH-------------------------------------------------------    
 674 UniRef50_Q67R99        Putative uncharacterized pr...   134     6e-30  1  13.9%  86:415    13:277     -------------------------------------------------------------------------------------QHFPLTGGTHMATVTLPSGLRLFYRERGHG-GQTVLLIHGNTASSLWWERVMAHL-PGHVRTLAPDLRGCGDSDKPAPP------WSIADLAEDVYQ----------------------------------------------------FTQAMGVQRCFVVGHSLGGGVAMQLAVAHPDLVERLVLINSAPAEGLVTPPERYAQLEAAVKA-PELLKAALALMMPTAPRDEFYQAVLEESVQKSAGALIPN-----GRALDGMNLVEQVGGLRVPTLILYGRQDGLVTLDMMERTRNQIPGAQlvWPEVGHSAPVEAPERLAKRLIEFF----------------------------------------------------------    
 675 UniRef50_Q5YP33        Putative hydrolase n=1 Tax=...   134     6e-30  1  10.0%  57:425     7:344     --------------------------------------------------------AVLAQLPAKIAELLTAHRANLRARTYPTTALNPPTAQREVIPVTRPDGARLRVHAYGPAGAPTIVLVHGWTCAIEYWNPQINAFAGD-YRVVAYDQRGHGESERGTAPL------TMDLLADDLAAVLDAAVpeKAVLVGHSLGGMTLQAWAARNPGEVTRRAHAVLLTNTAPHGLVDTTT--------VVPFFNRPMRFKRTGVSLPLAVGKVGLGTPIVFP--PVRPVRWLFARQVMCLACDSDRVDFGMAIVRSCPAAVRAEFGKLLAVM--------------------DLGEAARNLVVPTTVLAGSCDDLTPPVHSELIAEMLESvrYAVFETGHLGNVEMFERFNDELARVLDSVHSRRAEA------------------------------------------------    
 676 UniRef50_B8M0H5        Hydrolase, putative n=3 Tax...   134     6e-30  1  11.2%  96:417    28:314     -----------------------------------------------------------------------------------------------EHGRVALQDVSIHFRYAGS--GPPLLLVHGNPQFSLTWQFIGPILAE-HYTVIAPDNRGAGDSSIPP-----DGDYTSEASAGDLKGVLDFLNITSAYVFAHDKGGGMATALAIQHPSMVKRLVLAEYLFPGFGYEQASAPAPFWDLYGNPQLALFSIPDFAEF------------LINGKEKPFLQWYFYH------------------GSYSGVTSFSEDTVNRYTTSISKPGFLRAMLGPFSTATVNADAQFFRSHLGqlEIPVLNIGGEASF-----GSEDILSLISssVAVVPKAGHWLGDENPSWTANRVAKFFSE--------------------------------------------------------    
 677 UniRef50_Q2RX53        Alpha/beta hydrolase fold n...   134     6e-30  1  11.9%  84:419    10:304     -----------------------------------------------------------------------------------WEREGKTWPHRDCSRFVTVGGFHWHVQSMGpgDAPLLLLVHGTAATTHSWRDLMGPLA-RSFRVVAIDLPGHGFTRAPFSFRP-----TLPSMAKALSALLAAEGLSPDGVVGHSAGAAILARMVLDGSVTPRMLVSINGAFMPFPGMAGTLFPYMA----RVLFCNPLTPYMMSWGAADQQRVERLIR------------------------------------DTGSLLDKPGMTYYGRLMRSPAHVSSALSMMAH----WDLAPLNRDMRRLTIPLHLIVGEEDKAVSPDEAKRLATRVATATlvVPGGGHLVHEEQPDLVVGLIEQAAREVA------------------------------------------------------    
 678 UniRef50_D2PTS4        Alpha/beta hydrolase fold p...   134     6e-30  1  11.4% 102:416    18:292     -----------------------------------------------------------------------------------------------------APGVRLHYARAGVGE-HTVVLLHGYPQTWHSWRHVAAPLVRAGNSVVCIDYRGAGSSSRPAS------GYDKWTMAGDVRVLLREivNEPISLVGHDIGSMVSLAYALRFRDELSTLTLMEATLPGTDVYERLRVD----MRHWHFFFHQALDVPEQLTAGRERTYLKLFFDGAT--------------------------------FNPEAITLEDLSVYAAHFEQPGAMRAGFELYRAFARDDRDNRAavDAGRLTLPVLGVAGAA-NLFSDVNGEMLREVATNVQvvVPDAGHYVQEENPAGLLDVLIPFID---------------------------------------------------------    
 679 UniRef50_D2SB32        Alpha/beta hydrolase fold p...   134     6e-30  1  10.9%  84:422     1:304     -----------------------------------------------------------------------------------MDAQTDAAGSPGHSRTVDIDGPVHHVDFGGRADGPIVVLVHGLGGSHLNWDLFAPLLTP-HARVHALDLPGFGRSE------PEQRTANVRDNVVVLDRFVREVTGGPVVLVGNSMGGMVSVLEAQHSPAKVSGLVLVDAALPGPRGRP------------------------------DPRVAAAFGLYALPGAGQRYLAGRRRRLGARGTVREMLELCGVDPDDVPSSLIDRSVALVDERRDVAGMDRAFLTAARSLLLLLADPRlrRAMAGVQVPVLLVHGDRDRLVPVSVARDVARRHPDWGYVElgVGHVPQLQCPGLLAEHVLAWLPSVVPTP---------------------------------------------------    
 680 UniRef50_Q8TJZ1        Hydrolase n=2 Tax=Methanosa...   134     6e-30  1  11.6%  99:418    17:282     --------------------------------------------------------------------------------------------------YADFGDGKLYYEIEG--NGETLVLIHAGFVDSRMWDEQWKAFT-QHYRVLRFDMLGFGKSDPVTGPVSRR---------QDLYRLLQELGIEHARILGCSLGGETAIDFTLEHPELVRSLIVVSAAPGGFE--------------------MRGEPPALLLEMLQALEQGDLARVSDLQIRLWVDGSFRQPEQVDSRVRQRATEMNRIPVENGTWAIDD-----------------------ANPLSPLAPPAAGRLGDISIPVLVMAGALDNPEILRAADLLANEIEGAevIIPDTAHLPNMEKPAEFNRIVLGFLSRL-------------------------------------------------------    
 681 UniRef50_D1U5K8        Alpha/beta hydrolase fold p...   134     6e-30  1  14.3%  99:419    19:287     --------------------------------------------------------------------------------------------------VTTTDSARLWVEVRG--QGRPIVFVHGWTMSSRFWRRQ-QSLADA-FQVVTFDLRGHGRSD------TQLRGHTMPRYARDLREVIRALDIKGAVLAGWSMGGAVVLEYWHQFgdRLSGIALVESSPHPMADAPWNTHRYRGLGP---------------------------------------------EAVREDMEAMTRDRAGFGVRFINEMFLTGQAPAHALRWMRDEHLLATDQTAAAIYQDYVARDYTPILPTVTLPGLAVYGrrHMCFGPSTGRFVAGSIPDARFVILesGHMPFYEEPDTFNTEMRQFLTRLP------------------------------------------------------    
 682 UniRef50_Q2HWH9        Meta-cleavage compound hydr...   134     6e-30  1  10.6%  88:417     1:275     ---------------------------------------------------------------------------------------MTDVRDPGREHVVDAGGVKTRYWESGT--GEPLVLIHGGGAGAdnWSGCFPYLTP-HFRVIAYDMVGFGQS----VPDNPDFAFTQPSRIDHLAAFLDALGLERASFVGNSMGGATALGLAMRNPERIATLTLMGSAGLTRQ-------------------FSAELSTMLDYTEPSLDGMRRIVGALTHPR---------------------------------YQPSEELVQYRYQLTQDNRVMDAYRATMAWIREQGGLFYPEEDIARVKSKTLVVSGREDQVVPLDLSVRFHQLLDNSRLciAHCGHWAMLEHPDKLASAVVGFIDE--------------------------------------------------------    
 683 UniRef50_B5JWT0        Hydrolase, alpha/beta fold ...   134     6e-30  1  13.8%  99:425    23:318     --------------------------------------------------------------------------------------------------YVQANGQSLYYEDWGRESDPAIVMVMGLAAQLIVwtELVRSLAAQGYRVIRFDNRDIGWSskYRLGRPVEAPYTLYDMVEDSIGLMDVLGIQKAHWVGASMGGMIAQLAASLHPHRALSLTTIMSSTNERYLQWPALSVLRGLLRGRGPIHSA-------------------------------------EQAGEALLAFWQHIKSPALPADPQSVYRRAMAAYQRSFRPKAPLRHTLAVMATGGFAH-------LLNRVRVPALVIHGSVDPLIRLDGGQDSARHLPNARLKVVDggH----ELPEALIDDFAAWIGDHVSATTAP------------------------------------------------    
 684 UniRef50_C7R044        Alpha/beta hydrolase fold p...   134     7e-30  1  12.5%  92:420    18:309     -------------------------------------------------------------------------------------------DGPWDHEFVSAHGARFHVVTAgaNSGPLITLVHTTPLMWWQWRHTIPALADAGWRVAALDMRGTGASDKPPT------GYDQITRTRDVAGVIRSLGASKAIVVGHGLGAVTAWSMPSLQPAITVGVAALSAP--------------------------------------HPARLHASLRTSLNKLPWRRLPALHLPSLGERTLTRPETMSRLFTRFGHQPVPGQVIDTYANALRIPFAAHNSLEPLRWYTKGIAGPVGRRYLqgITVPALQLQGAHDGLLRRElaADSSALCSNLRFETidAGFFLTEEVPNTVNDLLIDWLSKSFP-----------------------------------------------------    
 685 UniRef50_C8QZ22        Alpha/beta hydrolase fold p...   134     7e-30  1  12.3%  57:453     5:346     --------------------------------------------------------LAMIFRVLAVMGLVALTTPALADMKPE-AEGPEAMTIKEHRLEEPIFGGQAYLLEAGRGNSPTLLLVHGLGEQASgwHDLLPELAVE-YHVLAVDLPGFGRSDKLNAL------YSPQNYAAYLNWLAESFSSGPMLVVGHSMGGTVALRFAADWPQQVKRLVLANVAGVLHRKVITQELLEPD-------LQQRLPGLPAAPLSRLDNWLSGIIAGLPDLPLDINQVLNNDTLRARLLGGDPAKIAALAV---------------------------------------AEENFNRDLRGLATPAFIIWGAEDHVTPLRTGLLLETLLPKAQlvIPEAGHVPMREQPELFRSALQQALTAPTPAAK---AKPWPGVAENTASQAERVGSCQDQHGL--------------------    
 686 UniRef50_UPI000169973B alpha/beta hydrolase fold p...   134     7e-30  1  11.2% 105:417    37:292     --------------------------------------------------------------------------------------------------------LQLHYREAGDPTAPPLILLHGLFGSSANWLGIARRLAAD-FRLIIPDLRNHGRS-------AHAMPMDYPVMVEDLLALMDQLGISAAHLCGHSMGGKVAMWLALQHPERLERLVVADIAPVSYSHRFDAIFDGL-----------LGVDLSPAATRQQLDQQLARWVDDAGVRGYLLQNLLR-------------------------------IDGEWQWRLNLSGLQAALPEWRSFPEVGGQAFPGEA--------LFLHGSCSDYVRESYRPIILRHFPFARLriEGAGHWLYAEQPDKFTALCCSFFSA--------------------------------------------------------    
 687 UniRef50_D0LX47        Alpha/beta hydrolase fold p...   134     7e-30  1  11.7% 100:423     4:296     ---------------------------------------------------------------------------------------------------ARVGDIELHYESEG--EGLPLVLIMGIGTQLTAwaGLRAALVERGLRVIRFDNRDIGLSSKLTHLPVpdVPYTLEDMAADTAGLLDALELDSAHILGASMGGMIAQVMALHHPQRMRSLCSVMSHPGNLRSMVARPKAYRALMSP-------------------------------------------LPKEHEAAVTHMVGVMRALSGSGFPFDEDAARAYLDLAIARSVHPQGFLRQFAAIAAA---PNRIPALRSLRMPSLVIHGTDDPLVPVSGGRATAKAIPGARLHLVPggHDFPRGAWPQIADAVSEHVRDAERARA--------------------------------------------------    
 688 UniRef50_A1SKE2        Alpha/beta hydrolase fold n...   134     7e-30  1  13.6%  96:418     8:294     -----------------------------------------------------------------------------------------------AQRSAKAKDITLSYYEAGtgGGLPLVMLHGGGPGASAWSNFGSALpaASFRTLLVDQPGFGASDKPPVVGNY-----YRHSADHVVALLDELGIDRVHLLGNSLGGGTAMRLALDHPDRVGRLVLMGPG--------------------------------------------GLSLNLFHADPTEGVQRLMDFGAEPTRENLRAFISTMV--VDQRLVTDELVEERFADATAPGAQDAMRSMGMSFWNPETAEdlWREAHRLRKHTLLTWGREDRVNPLDGAFAALKLIPKAQLHVfnCGHWAQVEAADEFAEVSIAFLARH-------------------------------------------------------    
 689 UniRef50_A8LPZ7        Magnesium-chelatase 30 kDa ...   134     8e-30  1  15.7%  89:431     8:302     ----------------------------------------------------------------------------------------AHWPHAWASRFLRVSPHQWHVQVMGpgQAPTLLLLHGAGASLHSFADLMDRLAPR-FRLVAVDLPGHGFTTK-----GSSMRSSLDAVAEDLTKLLVKQDLLPDAIIGHSAGAAVGLELTTRLPTPPRGLVALNGAFAEFDGVAGWLFPMLAKM---LALNPFTALVFAN--TTTPRSVRQLI------------------------------------EATGSELTEAQLGLYFRLVRDRAHVSGAL----AMMSRWDLRPLNRKMPNIDTPALLLTGARDKAVAPQVSIDAARKLPNATTRTLPagHLMHEERPDLIAAEIEGFLAGLDLHPARSQTSRSA------------------------------------------    
 690 UniRef50_A4X3T6        Alpha/beta hydrolase fold n...   134     8e-30  1  12.2%  99:417     6:270     --------------------------------------------------------------------------------------------------VFEWRGRKVRWRQMGS--GPDVVFCHGTPWSSQLWGPYAEALSSE-FTVYLWDMPGYGQSSKFP-----DHQVSLDLQSELLTDLVSHWELQSPHVVAHDYGGAVALRALLLHGCAFASLALVDV-----------------------VALAPWGSDFFRLVGDNTEAFHAL-----------------------PSAVHEGALRAYIAGASHVGLTPGQMQDLASPWLDTDGQAAFYRQIAQADQAY-TDEVQGLYPRLDLPVLVVWGKNDTWIPVDRAHRLAELIPGAQLELVdaGHLIQLDQPVQLATTLHRWLTS--------------------------------------------------------    
 691 UniRef50_A0YDX7        Haloalkane dehalogenase n=4...   134     8e-30  1  12.3%  77:417     3:360     ----------------------------------------------------------------------------ILRTPDHCFDGIKDYPFEPNYTVIQtdglRIHHIDEGPADGPILLAMHGQPVWSYLYARMIPYLVKSGVRIIAPDLPGYGKSDKPASRA----DYSYQCQVDWMNQWLVKNDFSQLTFFGQDWGGLIGLRMVVDNSHRFDRVAMGNTGLPYNPDTPAHVVDDILAFrmMKQVSTMDGFSNEDNPYAGPQKFAYWqyTWDTQDVPAGIIASSQMETRNKLAMAAELLMGNLGLRNLSPFRTDITRAYEAPFPSPAFKMAVRAMPSQVPSIPDRSlaWEFFAEFDKPFLCVGAGDDPVTN-GFEKTFLEKVPGTkhEMIGGGHFFQWARAEKLSEILSRFVHD--------------------------------------------------------    
 692 UniRef50_Q1CVN3        Hydrolase, alpha/beta fold ...   134     8e-30  1  10.0% 101:419    10:265     ----------------------------------------------------------------------------------------------------ETNGINIHYLRTGGAN-PPVVLLHGLMGNGACWTPLARVLEGQ-FDVVMPDARGHGGSSTP------HHGYRYDDHANDVVGIIRGLELSRPVLLGHSMGGMTAAVVASRGTGGLRGLILVDPTFLSPERQREVRDSDVADQHRWALGLHKSDLVAQARARN-------------------------------------------------PHRSPEIIDLLAEA--RLNTRLGAFDVL-----TPPNPEYRDVMSAIGVPTLLVIGDRSPVVTLEMAMGLRSLNPRVRIevQNAAHGLPFDQPERLGAVVVQFLRELP------------------------------------------------------    
 693 UniRef50_C3KLY2        Dihydrolipoyllysine-residue...   134     8e-30  1  14.0%  54:417    69:371     -----------------------------------------------------APVGALAGVIADEAATPEEIDAFIASYADRLGSGDEGEGGASVPRTITVDGGTVNVLEAGPESDETVVLLHGFGGDLSTWLFNQTALADK-MRVVAVDLPGHGASSPIAGEDV------FLKIVAAVEAAVDAVAPGKLHLVAHSFGGAVAAAIATNRPSRVGSLTLIAPIGLSRQMSRDFLVD---------------------FIAAERRRQLSSVLERLFADP--------------------------------SKITSDMVEGTLRFKRLEGVPEALSAIADTIADDSGqqSIGGQLKDLPCPVTILWGDRDEIVPLPQSTEVPA---NARLriPDVGHMPQMEASSAVNDAILETFQR--------------------------------------------------------    
 694 UniRef50_A4XBH9        Alpha/beta hydrolase fold n...   134     9e-30  1  11.0%  73:418    45:364     ------------------------------------------------------------------------RRLKADPTDRYAGEDFGAQRFDEALRVEMPDGTDIHVETVEPARapTIVLVHGFCLDQGTFHFQRKLLAAQghRIVAYDQPGHGRSGQLETG-----EYDLTALGRTLRAVIDRTAPepLVLVGHSMGGMTIMAFAELFPELFGDRVVGTVLLATSGGLLAETK-----------------LVAPALLGRVGAPVLHMMSNAARYGGPVIDRARRS--------TSNVAWLLTRRYGFGTGHPSPALVSYVEEMNSRTSADTVTRYLRTLATHSRFPALAALASAP---VLVLVGENDMITPVAHSEEIVRRLPHAEFviPNSGHVVMLEHADEVNAALTRFLEEL-------------------------------------------------------    
 695 UniRef50_A5GI16        Alpha/beta hydrolase superf...   134     9e-30  1  14.4% 101:420    14:316     ----------------------------------------------------------------------------------------------------DGRGLDVVWRQQgsSDSPAVVLVHGFGASSGHWRYTMPSLATQT-PTFALDLIGFGGSSQPKAVLPSDPdyGFDLWAAQVEAFCRQIVQRPVLLVGNSIGGVVVLRAAQQLGAHCQGVVLIDCAQRLMDDKQLASQPAWMAWIRPLLKTLVSQRWLSTALFRNAARPRVIRSVLGQAYPSGAN----------------------------------VDDALVDLLYQPTQRPGAAEAFRGFINLFDDHLAPELLATLEQPVHLIWGERDPWEPVAEAQDWAERFaqSLTVLPRVGHCPHDEAPEAVNARLLEILKAQAA-----------------------------------------------------    
 696 UniRef50_UPI0001C31346 alpha/beta hydrolase fold p...   134     9e-30  1   9.7%  80:418     1:289     -------------------------------------------------------------------------------MRTPEDRFAGLPDFPFEAHYRELDGLRLAHLDEG--DGPPVLLMHGEPTWSFLWRHAVAPLRDAGYRAIVPDHAGFGRSDKPTELEW----YSYDRHTSIAAELLERLDLRDVTVVVHDWGGPIGLRLAVEQPERIARIVVTDTGLFTGRQRMTPAWEAFRGFVEATEDLPIGMLVRGGCHRDPGDAVVAAYDAPFPDAASKAGARAFPLLIPRTPDEAGAA---------------------------------------------TGQRTLDALAADTRPTLVLWAEHDPIIPLRTGERFAQAiaEVAHVVPDASHFLQEDQGPLVGRLIVEWLGST-------------------------------------------------------    
 697 UniRef50_Q01TS5        Alpha/beta hydrolase fold n...   134     9e-30  1   8.4%  66:424     5:331     -----------------------------------------------------------------LIVASFAAAFVVGLPSDSARAFQAMRTAAPREGRISVGKTSLYYREIG--QGQPIIVLHGGPDFDHSYlpDLDRLAD-VFRLIYYDQRGRGRS----ADQVLPEEVTLASEIDDLDMVRRHFRLELPALLGHSWSTVLALEYALRHPERVSHMILLNPAP-ASTSDLAMFRKVYVQKLGADMDRQREILASPAYQEGDPEAVAARYRLHFKPALKRPEDYEKLMATMKAAFVRQGKEGIVKARAVEDRLMRESWQ-------------------------VESYDLLPKLQTLNIPTLVIWGDHD-FIPAEIAAHIARAIPNAQLVTldCGHFAYMECPGEVRNAVDDFFRRTRATGHP-------------------------------------------------    
 698 UniRef50_UPI000185C21A hydrolase or acyltransferas...   134     9e-30  1  13.4%  93:421    10:324     --------------------------------------------------------------------------------------------GPWRHRLIHVRGQRLHIAECGRPSDPLVLFIHGSTGGWFEWREVLPLLNDDsvHAVAISMRGYGQSDRTP------QGYDPFEAAQDIASSIRSLGHSTALLVCQGFGTWVAWTLASRQPNLVAGII------GCGAAHPKVWLNQLKSPWSEDFRSSLAPLLRRSWWDSRPRVPRLRRNKFSTQAAEARQQRLIDRIVRESMSTTGQGFAEAPKGKQTAELMADSLASGALrwWTRPAPVAFRKWLGAMEGSSTSDEH----------TVLLVGEQDTRTPRALVKLSSTHVR----VIPGCGHFLPVEKPQAVVQAIRDHLHWLPEG----------------------------------------------------    
 699 UniRef50_A4FN37        Similar to hydrolase or acy...   134     1e-29  1  13.1% 106:419    19:289     ---------------------------------------------------------------------------------------------------------KIRFHRAG-GSGPAIVLLHGGGVdmLSWRHTVPVLAVD-HTVFVPDLPGQGGSRPWHGRA------DQRTCEEALRWLLDAWRVPDATVVGMSMGGSIATGFALRHPQRVRGLVLAAPTGMQ-------------------------------------ARLDRHLLTYLLVKLRFPGMLWARAMGMSSALTRWVMTRSTFTGSQPAADLESILDEVRAEVRSRGTVFSDWQH-GAIDRRSMRINHLPHLDRIRCPTMVIHGEKDGIVPMPASQEAARAIPGAMFRWidAGHWPSREKPNEFNALLREFVNAHP------------------------------------------------------    
 700 UniRef50_Q5WGJ6        Proline iminopeptidase n=3 ...   134     1e-29  1   8.9% 102:415     6:293     -----------------------------------------------------------------------------------------------------VRGKKIHVEIFGDRSLPAVLFLHGGPgsCYEfyHQSNRFSDQ-FQLVAIDQRGVCRSE----GLSETEPFGLQDLVEDCEDLRKQLGINRWAVIGHSFGGYLALLYASVYPNAITNVVFECPTFDFAWTAKSLLRRAASVFLELGIETKSEECLNLATSNLSSEHLFDSYLRLGEELGEHKEDIYRV---------------NADTSIDYSAYTDEEWEAF--------DDRTDIHLERLREEGRIFQSIVSLLPSLSVPSLLVIGDADPVTCDKHVEAYNQMANGHKVVMNNCGHTPHAECPDVYYRIVSAFL----------------------------------------------------------    
 701 UniRef50_D0L2W8        Alpha/beta hydrolase fold p...   134     1e-29  1  10.0%  69:418    23:340     --------------------------------------------------------------------ARDAFRAEPAVDDGPDELLREPARAPERRQVRSADGTRLNVEVYGPDAAdiVVMVHGWTCNTAHWYPQINALAERT-TVVAYDQRGHGRSERGRARP------TVAMLGQDLDAVLDAvdGRRAVLVGHSMGGMTIMSWAAQYADKVPTTVSGVALTSTAAKAV-------------LQNHTLIPMNLPRYTRPFEATVGKLFTSAPVPIPKTPYGGRLSHYIALGPNARKAHVD----------------------FVDDMIGACPPRSRAGWGAAMGKLDVTAGLEALTVPTTVIVGTDDRLTPASHAEQMAEVLRrrDLVIYDggHMSSIEAAERFNELLADILAEV-------------------------------------------------------    
 702 UniRef50_A1AQT0        Alpha/beta hydrolase fold n...   134     1e-29  1  11.6% 104:414     9:266     -------------------------------------------------------------------------------------------------------GRRLWYEDQG--EGIPLVLLHGWCMSSQVWRFQLETL-QRDFRLIAPDLAGHGRSDLSPD------GFTLSALCADLDELFRHLDLRSALLAGWSLGAQLALQAWGTLRERLSGLVLVSGTPRFIADD-----------DFPHALEARELRGMQARMRRNP-----------------------------LITLRDFVSRMFAEAERDVCFPDGRIQRMLASIPLPEGEVAL-----QGLRLLGETDLRSLLPAVDLPTLIMGGDRDAICLPLASDYMARRIPECrvVFAGCGHAPFLTRCTEFNDAITRF-----------------------------------------------------------    
 703 UniRef50_C7PWP0        Alpha/beta hydrolase fold p...   134     1e-29  1  13.8% 107:417    11:284     ----------------------------------------------------------------------------------------------------------IHYRESGDPNGLPFVFLHGNPTSSHLWRDVLPRVAAPGRRLLAPDLIGMGASGKP------DLAYSFDDHARYLDAWFDALGVDEAVLVGHDWGGALAFDRAARLPERVRGLAFAETI------------------------------------------VKPLVGDEFPVAGRELFEQLRMPGVGEEMVLEKSLFIEGVQGTLATPLDPADLDVYRRPFPTPESRRPILAWTRMMPLDGEPADVVARVERYdvPKLLVAFEPGpmTDAGAVAWCEANIAGLEVSRHgaGHHCPEDRPEEMAAAINAWARR--------------------------------------------------------    
 704 UniRef50_A1B0V9        Alpha/beta hydrolase fold n...   134     1e-29  1  11.2%  99:419     4:264     --------------------------------------------------------------------------------------------------HVADDGCRIHCQLDG--NGPPCLLIPGLGGEAGFWQGVASTL-ENDYRLIRIDHRGAGASDRPTG------GYSIPRIMRDVLGVLDDLHIPRAHVVGHSTGGMIAQTLAVEAPERVAGLVLSGTWERVDTRFTRLFQ-------------------------------ARLALLEQAGPIAYHKLTQALGHDAGWIEANRAALD--------------------AELEQAEARLQPIAVQAARIRMLMEHDVFDRLGRITAPTLVIGGSDDALIPFASSERLAAAIPGARLASLPGGHFFPRAYPAPYAALLAGFLKEID------------------------------------------------------    
 705 UniRef50_D2PUV3        Alpha/beta hydrolase fold p...   133     1e-29  1  14.2%  97:414    63:325     ------------------------------------------------------------------------------------------------QQIVRVPGGDLHVVQDGPANAPAVVLLHGLAGSTRWWDPVLPAL--RDLHVIRIDLLGHGESAKPA------NGYSIAEHAARVGAVLDQLGVRRATVVGHSTGGAVATSLAEQRHELVTGLALIDTGPRVDAFLGESFVAKLMTTPV----------AAQLLWRLRTESAIRTALSTAFTREVQIPDQLVADVRRVTYRSLTATDEASTAYLTERPIPD-------------------------------------RAARLDLPTLVIFGSADRRWQPSSAEDY-RRVPRARIEILDggHTPMYEDPGTTGALLHQF-----------------------------------------------------------    
 706 UniRef50_A3M7C7        Epoxide hydrolase n=2 Tax=A...   133     1e-29  1  11.9%  96:422     5:306     -----------------------------------------------------------------------------------------------EQLTLNRDGLTLHGTLYGEADASLVVLIHGFPDTPHSWDHVYPLLVKAGYQVFVPWLRGYTQDSVNRQAHYGLASALADLAAWLELLKAKERLKNKAHLVGHDWGAAIAMSFASGQSEALHSMSLLAVPPIPN---LKDIVKAIPNFPRQLYMSSYMLLMQSRFAEQV------------------------------LSRNNAGFVEKIWRKWSPTwfTESDFSPTRAAFQNPNIAWAATRYYHNLFAVLNSDNLRalLSPVTTPTLALAGLDDGCMNIRLHQALAqyFPQgsVYVPGCGHFLQAEQPNIIAQYLIQHFQKTDRST---------------------------------------------------    
 707 UniRef50_Q0B7K0        Alpha/beta hydrolase fold n...   133     1e-29  1  11.5% 101:423    18:280     ----------------------------------------------------------------------------------------------------TVDGVRLAYRLQGEGPRE-LVCIHGVGSYLEAWQGAINAL-GRGFRVLSFDLRGHGRSERVKGR------YEIDDFVGDVLALADHAGFSRFDLAGFSLGGLIAQRLALTRPARLRRLVLLAT-------------------------------VAGRTAEERAGVAERLAALQAGERGAHYDASLSRWLTEDFQARHPALVAELRRRNAQN-----DPDCYAAAYRV-----------------LAQTDFGGLIDQIAMPTLIATGEHDAGSNPRMARYMHERIPDSRLVILplRHSLLNEAPEQVAALMREFLIDSGEAAR--------------------------------------------------    
 708 UniRef50_B1MKZ7        Probable epoxide hydrolase ...   133     1e-29  1  11.7%  99:415     8:297     --------------------------------------------------------------------------------------------------TLDLPNVRLRALAWGPQDGPLVICGHGFPDSAHTWRLLGPRLGAEGWRVVAPFTRGYAPSEIPA-----DAEYGLGALMQDVLDIHTVLGGdrAIYIGHDWGALVGNALARSGRSPFARIVTMAVPPFEVLGSNMASIGPGGW----------------------PAVLARQLFMS--------WYTLFHQLPLLPEWLLPWLLRLYWRRWSPGYDARADLLYTGEAMLQKGNRRAVIGYYRafPARKYWPTQRSLLDGARTPMLYLHGRNDGCMTYRLIDSARPDLPDRSVEIigGGHFVHLECPDVVYELVREFL----------------------------------------------------------    
 709 UniRef50_Q1IE44        3-oxoadipate enol-lactonase...   133     1e-29  1   7.2%  98:415     3:260     -------------------------------------------------------------------------------------------------HVQLADGV-LHYQLDGPADAPVLVLSNSLGTDLHMWDAQVAAWSE-HFRVLRYDTRGHGES------LVTDGPYSIEQLGGDVLALLDALDIEKTHFVGLSMGGLIGQWLAINAGHRLLSLTLCNTAAKIGSDEVWN---------------------------TRIDTVLKGGRQAMGELRDASIARWFTPAFAAAEPEQAQRICQMLAQTS------------------PEGYAANCGAVRDADLRD-------QLNHIQVPTLIVAGTADAVTTPEHGRFMQAGILGAEYVEFPAAHLSNVEIGAPFSRRVLDFL----------------------------------------------------------    
 710 UniRef50_A0LP61        Alpha/beta hydrolase fold n...   133     1e-29  1  12.4% 104:418    18:268     -------------------------------------------------------------------------------------------------------GVRMHYVERGPAGAEATVFIHGYADSWFSFSRVLSALSPR-HRAVAVDLRGHGDSEKPMG------AYSLDAFARDVVSFMDAVGVERANLAGHSMGSFIAQRVAMIAPQRVKSLTLISSAPTSKDHPLLLELK--------------------GAVDRLSDPVDRAFVAEFQATTNPVPADFMEGIISESLKIPARIWRETLHTLLLEDHSARLPEIVVPTLIAWGKQDGF-------------------------------------FTREFQDGLRELIPASNIKTYAAGHALHWEKPAEFTRDLEAFIAEA-------------------------------------------------------    
 711 UniRef50_B2HSI1        Lipase/esterase LipG2 n=3 T...   133     1e-29  1  12.1%  96:426     8:308     -----------------------------------------------------------------------------------------------ARRETTTGDVELFYEELGDPSGQPVLLIMGLGAQLPMwdEFCARLVTAGYRVIRFDHRDTGLSAKMRggRPSPVPYTLLDMTEDVAALLDHLSIARAHVVGASLGGMIAQILAADRPQRVASLGIIMSTTGKAFSAPPA-------WRVIKLAFGQPGPNASAEEKLAFEVRNVAVFNGPNFLPPQSDLRQRVRLLADRCNYPPG-------------------------------------MLRQFDAVLGTGSLLGYSKAITAPTVVLHGSADPMVRLRNGRAVAAAIPDARFVVVDggH----DLPQPVWRPILETLTENFSRAAPPH-----------------------------------------------    
 712 UniRef50_A5V570        4-carboxymuconolactone deca...   133     1e-29  1  11.8%  99:471     3:315     --------------------------------------------------------------------------------------------------FTLRGDVRLHWRLDGSDERPPLVLLNSIGTDISLWDRTVPHLLP-TFRLLRIDVRGHGASDAPAG------DYDLAMLA----------------------------------------------------DDVAAVMDDAGIAVAPVAGVSLGGMIAMEIALRHPDKVAALALICTSGAMDPAAWAARVDAVRDGGTGAIADMAIERFLSSDFIVRHPEIAESLKRAIRAQADDG----YVGAAAAIRDMALADRIGAIAVPTLVVTGAVDISTPYehGDRLVHSIPGAVRAVVAGAHLPPIEAPGQLAARIQSFldAAIVEAADTLFEAGLVHRRRVLGDDWVDRSLTKRTAFNADFQALITRIAWGEIWS--    
 713 UniRef50_C7YX47        Putative uncharacterized pr...   133     1e-29  1  11.1%  95:419    56:340     ----------------------------------------------------------------------------------------------FTHHFVESPgvSFHYVTCGSEGKqpVVFLHGLPDSWFEWHQHMASLAEDGYFCIAPDLKGYGQSSKEPG------DYRHEGAAEQLAAMLFKIGIEKFYLVTHDRGTVQGDYIAALFPDRVLGYI----------------------------------RGEQHLIHYNPALSSQHALFLHAPYNGIMEDPQRLALTAYMWFTSREIPDGAAT-----------LQRVAQELAYEGISKAVPRYYGASTFHQEWLDRRRRlgKWGCPIVIMQGYDSSTQPREwlEEIQGFVPRaeVKFIPGGHFWPLESPVETTAALCEALKALR------------------------------------------------------    
 714 UniRef50_C6H1Y9        Putative uncharacterized pr...   133     1e-29  1  10.0% 118:417    37:353     ---------------------------------------------------------------------------------------------------------------------PPLLLLHGFPDGPLLWRPLLPYLLSLPHRLIIPSLLGY----HPTSTPRDPTAYNSKSMSDDLAELLTAEDAPTAIAIGHDWGSFLANRLYLWHPERVAGLALLNLAYMPPNQTTPFDLDQTNALMEQLFGHPILAYWElrATGDVPALLEgeLLHAARPQPEWMRKLLCERGATEAFLTAGTVDGEAVAGDIKLRDYAGHDGVYWEDfrEAGGIAPALCWYRAMSENATFEVERTLEKelKVPTLFVACPDDAVCRAELIEAPKqmLPDLTVVEvgCGHwiIYEKAEETAGVIGGFLKE--------------------------------------------------------    
 715 UniRef50_Q21BV3        Alpha/beta hydrolase fold n...   133     1e-29  1  10.3%  69:418    28:326     --------------------------------------------------------------------PAVAPRRAELHPKPCWERDGHDWPNRDSSRFVQAAGFRWHVQQMGR--GPALLLVHGTGAATHSWRKLAPLLA-QHFTVVAPDLPGHGFTETPA-----WKRLSLDGMAEDLAALLRVLDHAPVLVAGHSAGAAVLARMCLNGSITPDGLFGLNGAMLPI------------------------GGMAGRLMTPFAQVLAGSRLVPRLFARFASSDKFIERMIADT----------------GSVLDPAGLEFYRRLTCSPGHVAAALR----MMGNWKLRPLAAELPRLLTKLVLVTGGNDKTIAPKDAARVHAMVPGSSVvmPGLGHLAHEERPHEVSALMIEFARRA-------------------------------------------------------    
 716 UniRef50_Q3A3Z9        Biotin biosynthesis protein...   133     1e-29  1  15.0% 103:416     8:266     ------------------------------------------------------------------------------------------------------DGRTISYREQG--QGPVVVMLHGWGMSSSVFLPLMQNLSD-SFRILAPDLPGHGHSE-------PGSGYDLPQLAADMEEWLGIIGITDSYLLGWSLGGMVALELLEHLGGRLKKLILISTSPCFVQRDTW--------------------------------------------KSGQPATQLKVLGRQFSRDSQAALHDFFARQFAGEDLDDHEILNLRHALLDSSPPPTKAAALGGLDTLGRSDLRRRCWPR--VPALVIHGACDAIIPVDAGRYLSRHLPDARWVELevGHAPFVTRTGQCAALFKDFLA---------------------------------------------------------    
 717 UniRef50_A5VE53        Alpha/beta hydrolase fold n...   133     1e-29  1  10.4%  99:425    45:327     --------------------------------------------------------------------------------------------------TIDARDMHWRVLRWGPRDGVGVLLLHGFPQDADSWRPVASTLAAAGYNVVAFDQRG-----ASAATLSRPGDYVFDNFMADALAVADATGLRRFHVVGFGWGGAMAWMLAAYHPDRVRSMTTLRYPH---------------------------PAAFVDGIEHDPEQRASWEAVRKEIGPDNIDKRAAALLADDATG--------LRAFLRKSGLPEDATDRYVARLSQPGVLVGGLSWGTALVPAQF-----AAVPPVRVPALFVW-SKGPAVSESTVQASARYapYRVVELQDVGHFMIETAPQTVAAPILDFLRETDAPPAAR------------------------------------------------    
 718 UniRef50_D0L5Z5        Short-chain dehydrogenase/r...   133     2e-29  1  12.3%  96:469     5:344     -----------------------------------------------------------------------------------------------RRRSVTNGGVNLAVFELGeaERPIVLLVHGWPDTHHLWTHVAPTLSEQ-FHVVAYDCRGFGESDHPTG----DAAYRLSEMADDLFAVAEAVSPDrvHVLAHDWGSVSAWEAVTRPGAERRIASFVSVSGPNLDYLGVWARKNF-----------------------SRPTPRNLARAMAQTGSSAYTAFFQLPILPKLFFTLTGSPRLWREFLHRIEGTPRENATFQANLRED-----MISGLRLYRANIRGKLRRPDPRPTSVPVLEIVNERDIALRAAIFDDTYLYTDRIWRRRTPTGHWLPLTKPDYLADAATEFI---AAHSGDAAAASAIERLRVFGAPKTLSGKLAVITGAGSG-IGRETAYALAEQ----    
 719 UniRef50_Q1I3R3        Proline iminopeptidase n=11...   133     2e-29  1   9.4% 107:413    24:307     ----------------------------------------------------------------------------------------------------------LYVDESGSPEGLPVVFIHGGPgsRCYFDPNL-------YRIITFDQRGCGRST----PHASLENNTTWHLVEDLERIREHLGIEKWVLFGGSWGSTLALAYAQTHPERVHGLILRGIFLCRPQE-ISWFYQEGASRLFPDYWQDYIAPIPPEERGDLVKAFHKRLTGNDQIAQMHAAKAWSTWEGRTATLRPNPL----------------VVDRFSEPQRALSIARIECHYFTNNAFLEQDQLIRDMPKIAHLPAVIVHGRYDVICPLDNAWELHQAWPNSElvIRDAGHAASE--PGITDALVRA------------------------------------------------------------    
 720 UniRef50_Q1GYV5        Proline iminopeptidase n=4 ...   133     2e-29  1  11.8% 105:401    23:298     --------------------------------------------------------------------------------------------------------HALYYEQSGNPAGQPVIFLHGGPGSGCN-PGQRRYFDPGHYRIILVDQRGCGRST----PQGEIRENTTSHLVNDLDTLRKHLGIDRWLVFGGSWGSTLALNYALAYPQHVTGLILRGIFLSRPSELEWFLHDVQHFFPESWHRLLSYLPVAER--------------HDPLTAFAARVFSDDPAVNAPAAIHWNAFESSIMTLLPVTATSEQGLNPDIELARARVQIH-YIKHQCFLEGRNLIAEASAQLRHI--PTVIVQGRYDMVCPPLTAYELHQAMPHAEFhiPDAGHSGME------------------------------------------------------------------------    
 721 UniRef50_P42786        Proline iminopeptidase n=45...   133     2e-29  1   9.2% 105:418    19:309     --------------------------------------------------------------------------------------------------------HQIYWEESGNPDGVPVIFLHGGPGAGAS-PECRGFFNPDVFRIVIIDQRGCGRS----HPYACAEDNTTWDLVADIEKVREMLGIGKWLVFGGSWGSTLSLAYAQTHPERVKGLVLRGIFLCRPSETAWLNEAGGVSRIYPEQWQKFVAPIAENRRNRLIEAYHGLLFHQ------------------DEEVCLSAAKAWADWESYLIRFEPEGVDE--DAYASLAIARLENHYFVNGGWLQGDKAILNNIGKIriPTVIVQGRYDLCTPMQSAWELSKAFPEAELRVVQAGHcpPL--ADALVQAVEDILPRL-------------------------------------------------------    
 722 UniRef50_P25026        Non-heme chloroperoxidase n...   133     2e-29  1  11.3%  99:417     4:278     --------------------------------------------------------------------------------------------------VTTKDNVEIFYKDWGPKDAQPIVFHHGWPLSGDDWDAQMLFFVQKGYRVIAHDRRGHGRS------AQVSDGHDMDHYAADAFAVVEALDLRNAVHIGHSTGGGEVARYVaqPAGRVAKAVLVSAVP-----------------PLMLKTESNPEGLPIEVFDGFRKALADNRAQFFLDVPTGPFYGFNRAG-------------------------ATVHQGVIRNWWRQGMEGSAKAHYDGIKA-FSETDQTEDLKSITVPTLVLHGEDDQIVPIADAakSIKLLQNGTLKTYPgsHGMLTVNADVLNADLLAFVQA--------------------------------------------------------    
 723 UniRef50_A1K6W9        2-hydroxy-6-ketonona-2,4-di...   133     2e-29  1   8.9%  89:415     3:285     ----------------------------------------------------------------------------------------TAHLETPASRFVTVDGLRLHYQQigPQDSLPVIFTHGGgtGWSNFRLSAQAFA-KHHRCYFLDLPQYGQSDMVVVDGPV-----FSWHAQKLLGFMDALGIARAHLVNQSFGGCVAIRLAVDHGERLGRLVMIGSQPVAHGVINPLPLASKHARGIFPDYFRETGGPSLEKMRALIAR---------------------------------------LEFHDDSRVDDENVRLRYEASINP----AFLDLIKTPGFFGRPEDLTALLPQVKVPTMIFWGLHDWFGGIDVPMLMLNRFRDARLHVVgaAHHLQTERPDEFNAAALAFL----------------------------------------------------------    
 724 UniRef50_Q39MF9        Alpha/beta hydrolase fold p...   133     2e-29  1   9.4%  72:422     2:303     -----------------------------------------------------------------------HPSTTPFTPAPTNSGSRLPGTPDPMHRWEGADGVRLAGDTWGHPDAPPVVLLHGGGQTRHAWGGTGERLADAGYFALAYDARGHGDSDW-----SADGDYDQDAFVRDLRQIVATLGGRRPALIGASLGGGTSLVAIGEHQVDARALILVDIVPYTEPTGVARIRTFMAQNADGFGSLDEVADAIGR-------------YRSNRTRPGNPAGLSKNVRLGDDGRYHWHWDPQFLA--------------------------------RTIDLPSRHARFSACARNLTLPTLLVRGGSSDVVSETGVREFLELCPHAEYvvAAAGHMVAGDRNDVFGEAAEAFLMRHVNAA---------------------------------------------------    
 725 UniRef50_Q31KF6        Putative uncharacterized pr...   133     2e-29  1  12.8% 102:421    16:300     -----------------------------------------------------------------------------------------------------WRGLRVRYAFRRSpgAVPVIFLHGFGAGWRHWRDNIPALAEER-DVYAIDLVGFGDSEK------GYLHYGPAFWSELVRDFCQQFVGSAAVLIGNSLGSVVAMVTAHRFPEQVHGLILLNLPDTSLLRSPAAHDRFKSL------------RQALLWALTPPWLIEPLLLWLRSPKRLKPW--------------------LALAYSDRDRIDADLLDLIARPARSEEAGPALRAMTRFNAEVPRDWRADRVLPQLSQPILLIWGESDRLVPFSLAKRCQQLNPQlwLPMPATGHCPHDDRPAFVNQSLNNWLSQHDPA----------------------------------------------------    
 726 UniRef50_A1WXH2        Alpha/beta hydrolase fold n...   133     2e-29  1  11.0%  83:427     6:301     ----------------------------------------------------------------------------------AWERDAAGWPNHEASRFVQAGGLRWHVQVYG--EGPPALLLHGSAASVHSWRDFGPRLAE-HYTVVALDLPGHGFSAAPSRRLQ-----TLSGAGEGIAELLDALGVAPRVAIGHSVGAALLARMALDGRIQPDVLISINGAFLPFSG----------------------------IAGHLFPTTARLLTYNPLVPFWLAFRARNRVFLADVLARTG------------SRIDEAGIDLYQRLATHPGHVAAALGMMARTH--DGLYALMEDLPQLTVPLVLFAASGDRTVPPAQAERVRLRLPSARLEILQggHLAHEEAPDRVTEQVLAAIRATAPKGRGGAD----------------------------------------------    
 727 UniRef50_Q13H58        Putative hydrolase n=1 Tax=...   133     2e-29  1  13.4% 113:417    18:269     ----------------------------------------------------------------------------------------------------------------GGLDGPVVVLLHGLSARADRWKHNLDALGQAGVRAMAIDLPGHGFASK-----HEDFDYSAHGYSRWLDQFVQTLGVEKLVLVGTSFGGFVAAHYAADHPERVAGLMAVGAIGLVPAGEARRMKTV------------------QWLGEMQKEQIRS---------------------------------RMYRGVLDPQLISEELVEEDYRINNSAGAAEAFAALARYYRERLDDDAVAARLADarFPVSIVWGRDDPSVSPDYGARAHQIIAGSTLTFvdCGHFPYWERPAEFNALLTNFVKR--------------------------------------------------------    
 728 UniRef50_B0ULS3        Alpha/beta hydrolase fold n...   133     2e-29  1   8.2%  88:417     2:301     ---------------------------------------------------------------------------------------PADLFPGFDSHWIDLPDGRFFARAGGPAEGPPVLLLHGFPQTHACWHRVAPALARR-HRVICLDLKGYGWSAAPKGDPA-HEAYSKRRMGEEAVAVMERLGHARFALVGHDRGGRVGYRLALDHPGRVARLALLDILPTLVQWRRIEADPALYPHWRFLAEPAPGPE---------------------------------TRIGRGPDAYYEGLLRGWTGDGTLDAFAPGALALYRQSWSVPERIHAMCEDYRAGAGPDRAADEADLarTLACPTLILPGTGFvgLDPVLGAWRETFAPAAAGAAIASGHFVAEENPEETLRALVPFLRE--------------------------------------------------------    
 729 UniRef50_C8WXW7        Alpha/beta hydrolase fold p...   132     2e-29  1  12.1%  98:420     3:276     -------------------------------------------------------------------------------------------------RVALIRGVRYAYRLV--PGGEPCLFLHGFTGAGDVFVPVLERLASLGCraILPDLLGHGASDVPDDAARL----SMDETVRDLDALLDELGIPSCRVVGYSMGGRVALAFAISRPHRVRALVLES--------------------------ASPGIEDASEREARRHEDDRLADEIEARGLDWFVSEWERRPIFATHEGL------------------PDVEKARQRAIRRSGSARGYAQSLRGLGTG-RQPSYWDALGRLTMPVALVTGALDAKFT-GIAERMQSRLPNAVHviDGAGHTPHLEQPDRFATWLAQFLSASGR-----------------------------------------------------    
 730 UniRef50_A8TSZ7        Possible epoxide hydrolase ...   132     2e-29  1  11.5%  95:417    12:297     ----------------------------------------------------------------------------------------------FDTRRIVVDGVDVPVRTAGS--GPPVLLLHGFPQTHTAWHQVAPALSGE-FTVVVPDLPGYGAS---RGLEPTAEKFAKRNLARLMVGLMDTLGHGRFALAGHDRGARIGYRLALDHPGRITRFAPIDILPTLEVWEAMNWQAALGSYHW-------------------------LLLAQPAPLPENLVGADGPGYVRHLIDR---------WAGHHDRLDPTAVAAYARAFADPAVVAAACADYRAGATLDVEHDrrDAGRKIDCPVLLIWARrlKERGRRHLDVWRSWAEDVRELPLDCGHFVAEEAAQDCAAALLSFFSE--------------------------------------------------------    
 731 UniRef50_B7VA17        Probable epoxide hydrolase ...   132     2e-29  1  11.3% 106:423    21:300     ---------------------------------------------------------------------------------------------------------RLRVAVKGS--GPPLLLLHGYPQTHLAWHRIAPRLAED-YSVVLADLRGYGESRALDE---EGADYSKAALARDQLETMGQLGFERFAVIGHDRGARVGYRLALDHPQAVAAFVSL--TVVPILDNWAAVNKAFALNAYHWFLLAQPYDLPERLIGADPEHFLDYTLRRMAQGRDIYH--------------------------------PQALESYRRAFRDPAVRHAMCEDYRAavDADADQADRDAGRRLQCPVQVLWQERPYAAGQHPLEIWKTWAGQVEGAAIGASHMLPEDAPDAVLEHLLGFLASHREALR--------------------------------------------------    
 732 UniRef50_UPI0001AEF324 alpha/beta hydrolase fold p...   132     2e-29  1  11.1%  84:425     1:290     -----------------------------------------------------------------------------------MPAAPHPPTRESTSRFVTVDGTRLHYHEAGTGP-EVLLCIHGGAPGASGWGNFGTNLAerHARVLVVDLPGYGRSDTIDLTGGKYRP-----HADVFAAMLTELGIERASVVGLATGGAVAIAMALHHAERVDRLVLVSSAGGVPLFSTMPSEGQKAIRSYY---------------------------------------------AGEGPSREKMRAYLRMMMFDPGLVTDALveRYLESVAGGEERRAAAE-----SAGVPAEPLWQELGRIAAPTLIVWGTENRVQGFDNALFLLKQIPDAQLHLfrTGLWVPHERASEFTRLVLGFLAERPAAPAPV------------------------------------------------    
 733 UniRef50_D0LBG4        Alpha/beta hydrolase fold p...   132     2e-29  1  11.7%  81:412     6:285     --------------------------------------------------------------------------------PQSRRPSPIVGRVETELRSVTVGDLTFDVRVGGPDRGPWVLLLHGFPVNGSCYDDVVPRLHESGLRTIVPDQRGY----SPGARPEGVEPYRLDHLVADAIGILDALEVRYAILVGHDWGGIVAWHLAARHPDRFTGLVVASTGH----------------------------PVATREALANSDQRERSSYIKDFVTDGFEEKLLARDGV----------------LLRRAGVTAEEL----APLTEPGALTPPLNWYRANFLGDIKATMS--CPPVEIPTTMVWSDGDPALGREQAVGSARYvsDFRFCELPgdHWIPQKASAALASEIA-------------------------------------------------------------    
 734 UniRef50_Q9FFZ1        AT5G13800 protein n=10 Tax=...   132     2e-29  1  10.3% 105:445   102:439     --------------------------------------------------------------------------------------------------------LRVHYEKAgnLDAPAVLFLPGFGVGSFHYEKQLTDL-GRDYRVWAIDFLGQGLSLPTEDPTTMTEefSLDLWRDQVQYFVEEVIGEPVYIAGNSLGGYVALYFAATHPHLVKGVTLLNATPFWGFFPNPVRSPKLARLFPWPGAFPL------------PERVKKITEL-------VWQKISDPESIAEILKQ---------VYTDHSINVDKVFSRIVEVTQHPAAAASFASIMLAPGGELSFSEALSRCKENNVQICLMYGREDPWVRPLWGKKIKKEIPNayYEISPAGHCPHDEVPEVVNYLMRGWIKHLESG--GFEALPLLEDTEEDWEESRIGR----------------------------    
 735 UniRef50_B9L4M3        Haloalkane dehalogenase 1 n...   132     2e-29  1  15.2%  99:420    11:287     --------------------------------------------------------------------------------------------------FVYVEGWRWFFRESG--EGEPVVLLHGIPTSSFLWRKVLPVLA-RERRVIAPDLLGFGRSDKPRHGAE-----TVAQLAERLSALLDRLRVERCALVGHDFGVLVAAALVERWPDRVTHLVVTNTSF--------RIERWRGGSLSPLQLLRIPVLGEIALALARPWMLAAALR----RYLNDQSVLDRAMLARYWEPFELGYRRTLIRSMRVPPFSADDLARWRATLVERGRG--------------------------GLPLLLAWGARDPQFGVDEARELASAIPGARFLSfqASHFLPEERPRALGRVIAVFLERPAA-----------------------------------------------------    
 736 UniRef50_B8KEV0        Putative magnesium chelatas...   132     2e-29  1  12.6%  86:411     5:279     -------------------------------------------------------------------------------------ELPGDWPDAATSRFVDAAGLRWHVQVRGT--GPDVLLLHGTGASSHSWVPMARLL-ERNFRVIAPDLPGQGFTQLAPAQQC-----SLAGMAQAIAALLDVLGAEPELIVGHSAGAAIAATLCLRGSFDPKAIVSINGAMLPFGRAAAPVFSK-----------------AAQLLAASPVFTQLVALHAVPRKPV-----------------------ERMLRQTGSKTPPEMVRCYRELLGKPRHVAATLR----MMANWDLAQLEGNLDRLNPALCLLVCDKDSVVLPAQGEELARRVPQAQLHRIDggHLGHEEHPRWFVDQV--------------------------------------------------------------    
 737 UniRef50_B1LSE6        Magnesium chelatase accesso...   132     2e-29  1  11.9%  77:413     5:291     ----------------------------------------------------------------------------LDAERPDWAVEGRDWPHREASRFVEAAGLRWHVQEFGSPDAPTLLLLHGTGAATHSWRGLAPLLA-RDFFVIAPDLPGHGFTDPLP-----DADLSLPGMARAVAGLVAALGRPPALAAGHSAGAAVLARMCLDGAIAPRLLAAFNGALAPLPGAANLLFPSM----------------------------ARLLFLNPFTPRIFAWTADRSAAKR-------------LIEGTGSRLDPEGVDLYRRLFARAGHVRGAL----GMMANWDLAGLHRALPGLATRTLLIVGGDDKAILPDTAFSLRDRLPDARVRLIRggHLAHEEAPERVAEVLRA------------------------------------------------------------    
 738 UniRef50_B5I881        Hydrolase n=14 Tax=Streptom...   132     2e-29  1  13.8%  80:456    10:354     -------------------------------------------------------------------------------PPATVLPKVAPVRVGEGERLRSVrgITLTVRSRPPagLPPALFVHGLGGSSQNWSALMEQL-EGDVDGEAVDLPGFGDSPPPD-----DGNYSVTAHARAVIRLLDASGRGAVHLFGNSLGGAVSTRVAAVRPDLVRTLTLVSPALPEIRVQRSAVPTGLLALPGVAGLFTRYSKGWTAE-----QRVRGVTALCYGDPGRVSPEGFQYAV--------------------------EEMERRLQLPYFWDALARSARGLVNAYTLGGQHALWRQAERVLAPTLLVYGGRDQLVGFRMAQKAARAFRDSrlTLPEAGHVAMMEYPETVAAAFREFAADKGASDGSVGGSERRDGSAGASGEGNSGDGADAATASGDG-----------------    
 739 UniRef50_UPI0001BC9A2A alpha/beta hydrolase fold p...   132     2e-29  1  10.3% 103:414    18:289     ------------------------------------------------------------------------------------------------------NGVRLRYAVGGTGPKN-ILLIHGYPETAIAWRRIVGPLMESGYRVIAPDFRGAGGSTRPAD------GYDKAQMARDCFAVldAEIKAPTFVVGHDIGMMVAYAFARQFPGSTKALAVLDAPLPGTEVFDHVLLDDKRVWHFH---FHQAPDIPEALTAGKEAFYLERFWHDLA--------------------------------YDAGAIDLATKQRYLESFSGPGGMRAGFQLYRAFAQDAAFNRTqtEHGKTAVPVLAVAGESSAFAPV--MERMMKEVAKSvvCLARAGHWLAEENPAGLVEALLNF-----------------------------------------------------------    
 740 UniRef50_C1D4I7        PcaD n=1 Tax=Laribacter hon...   132     3e-29  1   9.6% 107:417    11:264     ----------------------------------------------------------------------------------------------------------IHYVLDGHPGQPVVVLLNGITMTTRCWEEQVSGLASR-FCILRLDFRGQGESDKPD-----CDYYPLSRQADDVAQVLDRLDIGRAHVVGLSYGGMVAQHFAQRYPDRLDRLVLAATMAYSDAANDAIAASWLAAW--------QTGDAALRFDLSKPWLYGSRFLTGA--------------------------------------------AAQVEAIRTANDESTDWAAIARLMLGVTRHDSRPWLSAIQAPTLVLVGEEDRLTPLYQARALVRGIRLARmeLAACGHALHAEVPQLFSQHVCNFLTA--------------------------------------------------------    
 741 UniRef50_A5UW59        Alpha/beta hydrolase fold n...   132     3e-29  1  14.9%  74:415    47:351     -------------------------------------------------------------------------RLSIHDQASAPQPATLLRELLGAPQVVELSHGPLAYRAAG--HGPPLVLLHGWAASSRYWLTTLADLSSD-FRVYAPDLPGFGDSPALPEPA------TVARQAQVVTEFADALGLTTFDINGHSYGAAVAASLAAAQPLRVKRLVMTSLGAIGNE--IERLIFALARAPLDLSLRVGYPWLNLIAPWVELWRPFATALLCIPPLPQLMAARFIANGLREKWMLQEGIVD----------LTKMDLRAHLMAVASVG-----------------DPQVFDALRSAPQPALLIGGVGDQIMPPDSLQAAAQIMRQARLALIdcGHIPMIEQPEAYHAALRSFL----------------------------------------------------------    
 742 UniRef50_Q2CB28        Putative hydrolase n=1 Tax=...   132     3e-29  1   9.4% 109:417    10:268     ------------------------------------------------------------------------------------------------------------VTLAGREDGPAMVFVHGFGCDQSMWRQVVPAFADR-YRIVTYDLTGMGRSD--LAAYDFDRYDRLEAHADDLIGILAALELEDVILVGHSIGASIAVLAANAAPERVARLALVSPSP-------------------------------------------AFVNDDASGYVGGFTREELEGLIAFLEENHLGWSSQMAPTIMGQPEDGEEAQELTQSFCRTDPDIAA-----HFGRLTFLTDCRDAMARAARPSLIVHCREDALVPMAAAEWMEREIPDSTLTVLEagHCPHMTVPGDVVAAIRDGLGR--------------------------------------------------------    
 743 UniRef50_A0LVU0        Alpha/beta hydrolase fold n...   132     3e-29  1  13.9% 110:446    54:357     -------------------------------------------------------------------------------------------------------------RVAGSAESppLLFVHGLGGDSLDWAELAGVLRAHDPtlIAIDLPGFGFSLPPA-----DGDFSLDAHGRAIAGCIEALDCGPVHLVANSMGGSIATRLAARRPELFTSLTLISPALPDPLPPRGSW---------------------QLLPAALPAIGPRLVSAGLRVDPEWVVHHAIRLCFGNPNRIDDTRRRLLFDIVRRRAGTAHGVDAY-------------CASLRSLIASYGRRRLWADAAAVDVPTLLVYGGRDRLVSPRSAYRALRAFPHARLvlPDAGHVPHLEAPEVVAGALMAFFRGApvAGTGRPAPPAGMVAAHATVDVGATGP---------------------------    
 744 UniRef50_Q0SCQ2        Haloalkane dehalogenase n=2...   132     3e-29  1  11.3%  89:416    10:283     ----------------------------------------------------------------------------------------ADTDRWHLPDRLTVDGATIAAGVFG--DGPPVVLVHGTPSSSYLWRAVIPRLAEQN-TVYVWDLPGYGDS-----PSVPGEPVAIRTHARVLARLVEHWGLDRPVLVGHDIGAATVLRAHLVEGVPVRRIALLDAAVLSPWVTPVA----QHMQRHLDVYRTMPTHIFQRITEAHLDTATRRRLPAS------------------------------------------VAEAYLGPFAGASGQQRYLDQVQYFDER-DTADVVEKLGTVKVLVQIVWGADDEWLDRSFADDLARRIPGARAtvPGAGHFLTEDEPALVAQALADFLA---------------------------------------------------------    
 745 UniRef50_Q2W7Q0        Predicted hydrolase or acyl...   132     3e-29  1  10.6%  77:416     9:301     ----------------------------------------------------------------------------ATRYTQPLHNAKGGAMTLPRSRYLSVMGREFHLTEWGDDAAPALIMWHGLARTGRDFDTLAAHFSDR-YRVICPDTLGRGLSGWSRAP---RRDYTLDTYMRHAVALLEALGIERLDWIGTSMGGALGMLLAAGPLkrMTRLVMNDIGPALNPVAVERIRAYLAQHP------------SFRTMTEFEAFVRQVYKPYGFQTDAEWRRMTETSARRRGDGRITPHYDPAV--------------------------------MRVFAETAGDYEVWAEYDKVECPTLVLRGVDSDLLLPEVADEMTRRGPRARLvvPGCGHAPALNVPEQIA-VLEEFLD---------------------------------------------------------    
 746 UniRef50_A8I8U0        Hydrolase n=19 Tax=Bacteria...   132     3e-29  1  11.9%  82:417   114:405     ---------------------------------------------------------------------------------AGPSSPTSANGHTAMPSVTTKDGVEIFYKDWGPKEAQPIVFHHGWPLSSDDWDAQMLFFVSKGFRVVAHDRRGHGRSS------QVSYGHDMDHYAADAFAVVEHLNLKNAVHIGHSTGGGEVARYVAKHGqrVAKAVLVSAVP-----------------PLMLKTAANPEGLPLEVFDGFRKATAENRAQFFLDVPTGPFYGFNRPNAK-------------------------VYPGVIQNWWRQGMMGSALAHYEGIKA-FSETDQTEDLKTITVPTLVLHGEDDQIVPIADSakSIKLLKHGTLKTYPgsHGMLTVDADVLNADILAFIKA--------------------------------------------------------    
 747 UniRef50_B5JS78        Non-haem bromoperoxidase BP...   132     3e-29  1  14.5%  99:415    13:303     --------------------------------------------------------------------------------------------------YTTRDAQPLFVRELGKGD--PVVLLHGLALDSLSWLPF-TALHQRHKRFYIPDLRGWGKSRHLP----LDDHY-VAQMADDVQDLILKIGAPKVTLVGLSMGAMVGLEYLKRHGdrVSRYMHIDAPAHLIRADNPKVVTPDLIDTGLEMLEESRYFDTSNTLAQLPKSYQRKHHKMIFGVLRNSLKMPWQRALALSVEYNP------------------VLRNNIARFTSQGCWYSVLRLIESLVN--DQYDTREALPLLQLPVTNLVGTHDTLFPLEETQAVTQLFPNatEVFEDSGHLLMLTESPKFAKIWREFL----------------------------------------------------------    
 748 UniRef50_A3UIZ8        Probable prolyl aminopeptid...   132     3e-29  1  11.8%  84:423     1:337     -----------------------------------------------------------------------------------MTPLFPALEPYASATFFSAEGYRIFYECSGNAQSPTVLFIHGGPGSGASARH-RRYFNPGKWNIVLFDQRGCGRST----PLFELETNTLTAQIDDMERLREELGLERWTLFGPSWGSTLALAYAQAYPERVDTLVVEGVLLGEREEIDWFHDCAGAGGIWPDAQHRLMMDTPEAF--RRPPAFRQWAlrEELAEGRPHLEGLEDPDTPIESLRQSIIYRWSEYEETLAWlpEDIREDFAERGRDWLTGHSLLewYFSHDCFLEPGQLLRDAHRLTMPMHVIQSRYDLVCPGRAAYRLAEAAPDARLHWVesGHI--MTAP--VHREVRTLFEALAASRR--------------------------------------------------    
 749 UniRef50_D2LBR0        Alpha/beta hydrolase fold p...   132     3e-29  1  11.0%  98:425    62:336     -------------------------------------------------------------------------------------------------RTATVDGHELRYWDAGPANKPAAVLLHGFSASKENWLNVVLFLA-RSHRLLVPDIPGFGESSF-----VPDASYGLAAQADRLKAWFAQTGAEKAHWVGSSMGGALAGLVAAKSPDLVRSLILMDSAGVAGEGLSPFEAGLLDGRN--------------GLIAEKPEDMEAIFTLLSGKNGGGFQNAILAALVARDQIARAPVYRHLFREMI---LSPELPATHWA-------------------------------PNIAAPTLVVWGEADKILDPAEASVLASLIRGCEILMmeVGHLPMLEAPARTAALLKLFWSRFESSDAAA------------------------------------------------    
 750 UniRef50_C8X969        Alpha/beta hydrolase fold p...   132     3e-29  1  12.1% 122:418     2:267     -------------------------------------------------------------------------------------------------------------------------LLHGFGQYWRSWRYQIPGLAQAGFRVVAPDLRGYGDSDKTP------RGYDAFTLADDVAGLVRSLGERDAVLVGHGYGGVTAFNTAVMKPDQVRGVVAIAAPHPIRMAKVRRPVRSDRYGRL-LSWASIPFYPEHRLAAANGALLERIVRSQSGPV------------------------------WKASRDFGETMAKMRLAIRIPGAARGAVEHLRWVARSPLRHdrEALTRPVTAPVLHMVGDADRFTPASSLTDAREQCTGWYttVRGVGHYPAEEAPDLVTRLIIAHATRA-------------------------------------------------------    
 751 UniRef50_C4DDT2        Predicted hydrolase or acyl...   132     3e-29  1  13.2%  81:421     8:317     --------------------------------------------------------------------------------PESAVRSLQRLAPPWPGREHRVGQQRLFVRETpsDDAEPALYVHGLGGSSQNWTDLADVLSDR-FAGQAIDLPGFGHSE-------PTARYTIPALAQQVAAWITQSGRGPVHLIGNSLGGAISVYVAATRPHLVRTLTLISPAMPFADVRRSTQSRLLPLLALPRADRI----AARAMAGLSPEKIVEQVVANCWAEPDTVPPQRRVEAIAEVRRRLNVPWNTQVYVRTFRSLVGHFLQSYL----------------------PGSSSLWRLATQVTAPTLVIWGKQDRLVDVRLAPQVARAVTDSRLLILdvGHVAMMERPEVTGRAVVGMLDELEAA----------------------------------------------------    
 752 UniRef50_Q0AQK4        Alpha/beta hydrolase fold n...   132     4e-29  1  11.1%  45:413     4:314     --------------------------------------------VLLAVLGGIILIFILALFIAWRFSHMVAKKASAALPPKG--------------HFTEVSGGRLHWIEKG--EGQPVVMIHGLGGNHHNFTYAmeRLAAAGYRAIAVDRPGCGWSER-----DSDEQARVPEQARMIAEMLEKEGIDKPLLVGHSLGGAVSLALAVNHPDRVGGLALVSALVTPPDTPAEAFAG--------IDIANPGMRRFIAETIAIPMSIRNGAKTLAIIFGPDPAPEDFRTRGGGLLSMRPDAFYAAATDLT--------------------------------AVGKDMHAITARIGEIQVPVGMFYGMADRILspEQIAALSAALPDADVETMEagHMPPVIEPERLEAFIVR------------------------------------------------------------    
 753 UniRef50_A7HU12        Alpha/beta hydrolase fold n...   132     4e-29  1   8.3%  99:422     8:288     --------------------------------------------------------------------------------------------------FILAQNLKIAADAFGPDQARPVMLAHGGGQTRHAWQACGRRLGENGYYALSVDLRGHGESDW-----SPDGDYRMERFAEDLLDVADGFSVPPVLVGASLGggMIAQGEYAKAGRrfAALVLVDITPQMNMEGVQKILGFMAS-------NLEEGFASLEEAADTISR-----YMPGRPRPKNLSGLAKNLRLRENGRYYWHWDPRFITGTQKPQGSRE------------------------------PERLQMAARSLKIPTLLVRGRDSDLVTVEAARQFAEIVPHSEFivANAGHMVAGDKNDIFTSAVLDFLTRMAPAA---------------------------------------------------    
 754 UniRef50_A3VR44        Putative hydrolase n=1 Tax=...   132     4e-29  1  12.8%  92:420     4:287     -------------------------------------------------------------------------------------------PYFITTRTFTVGNQSLAVRECG--DGLPVLLLHGFPDTAETWDDTLPVLADAGFRAIALHLRGY-----RPSSLAADGDYSVATIAEDVAALADALGLGRFGIIGHDWGASVAWAAAALWSERVAFVVALAIAPSRYVHASLR----------ELLARPHNLYLAWGPIARWWLRRRNLAFVESCYRKWSPNW----------------------------HVPEAHMERVKSALSPTNRAAAAVAHYRH--ARLGRPLPPECGSKVRVPSLAILGDDEPSSRKRMFDTFldAQPDQRVVTYDggHWPHLEVPERFHDDLLGFLADIDA-----------------------------------------------------    
 755 UniRef50_C4KBD1        Alpha/beta hydrolase fold p...   131     4e-29  1   8.9%  95:432    20:303     ----------------------------------------------------------------------------------------------EIGRSIHAAGILTNYHDLG--EGFPALFIHGSgsAWANWRLVLAPLAQR-MRVIAPDMAGFGFTERRA-----DLRYDIDTWVAQALGLMDALGLERADVVGNSFGGALALALAIRHPQRVRRLVLMGSVGVHFPITP--------------------GLDAVWGYTPSFENMRRIMDCFAWDRSLVNDELAELRYRASVRPGFQESFASMFPAPRQRWVDA-------------------------------LASREEDIRALPHETLIIHGRDDQVIPLQTSETLLRWIERAQLHVFgcGHWTQIEHSTRFVRLVGDFleEGERRAWPAAGRAEGG-----------------------------------------    
 756 UniRef50_C1BMA2        Serine hydrolase-like prote...   131     5e-29  1  11.7% 111:417    37:312     --------------------------------------------------------------------------------------------------------------VWGPDHGRPVLCLHGWADNCGTFNTLVPLL-PQDFRYVAVDMAGHGLSSHRP----PGVFYSFPSYVADIRRVIDALHWKKFSIIGHSMGGNVAGMFSALYPEMVESVVLLDSYGFLP-TDLKEIGRVMRQGFDEMLQFEKKTEEMKERVYTHEKAVQRLMVANPT-LSEQSACILLERGLAEVEGGVVFTRDFRINLKNIARVGLEQCLELQSLIQAKVLLVLADEGFEKMFSEPDQKKFTSAL---------IQGYKD-----------HKGI----VMTVPGDHHVHLNNPERVATFVADFLQS--------------------------------------------------------    
 757 UniRef50_Q5LV17        Hydrolase, alpha/beta fold ...   131     5e-29  1  13.2%  99:417    15:309     --------------------------------------------------------------------------------------------------RVEANGIYLEVEQSGPADGVPLILIRGQGSQLVHwkELGAGFAAAGFRTIAFDNRDVGLSRRCPAPGVPDspYGIEDMVDDITGLMDALGIERAHFFGISMGGAVLQQLCIDHPGRVLSATIVMTACRPFTERAGEGREALLALAANLLVRDTSQPEYLEAQIA----------------------------------------EHGLWGSPGYPMPEADIRAMAERAWARGVDAAGKN--RQVLAILHAPDRRPGLRQLDLPCLVIHGRQDTLVPLEMGEEIAAHIPASEFHAIEggHIITPKLAPVMVDLVRNFITR--------------------------------------------------------    
 758 UniRef50_D0MF68        Alpha/beta hydrolase fold p...   131     5e-29  1  12.5%  89:415    21:304     ----------------------------------------------------------------------------------------PLVGYPDRLDTAVVHNYRMVYHDS-RTFGPPVVLVHGLGTNLSVWREVIPRLATQA-RVLAPDLPGFGLSDKDGVPATPSF------YADVLAAWLDTLQLTQVDVVGLSMGGQIALMMALRHPNRIRRLVLAAPAGIETFTPEAAAQLK--------------------------------ALFTAEAIAAMPPALYAQNVRRNFARWNPDRFGWLLTQRAQMQERPD--------------FRAYAEANARAVAGMLDEPVFEHLPQVQHPVLVVFGENDLLipADMLRLALERLPNAQGVmpEAGHLLVLEQAEAFVAQVRRFL----------------------------------------------------------    
 759 UniRef50_C5CU02        Alpha/beta hydrolase fold p...   131     5e-29  1   9.8%  93:419     3:302     --------------------------------------------------------------------------------------------RMEPLRKIEAGVLEIAYYETGPSDGPPVLLMHGFPYDIHTYAEVAPMLAGQGCRVIVPYMRGYGSTRFLHDATPRSGE--QAAFGADLLALLDALEIDRAVLAGYDWGGRAACVAAALWPGRCAGLVSLNSYNIQNIAKAMEPDTPENEHSLWYQYYFHSERGRAGLAKDRRALTRLLW-----------------RLWSPTWKFDDATFERSAAAFDHPDFVEVVIHSYRHRFGLVAGDPAYADIERRL----------AAQPAITVPTITFDGIDDGVRPPADASAHAQRfpRSHRLVPGAGHNLPQEAPRIFADAVLELVPALR------------------------------------------------------    
 760 UniRef50_D2Q3T3        Alpha/beta hydrolase fold p...   131     5e-29  1  11.5% 102:413    11:280     -----------------------------------------------------------------------------------------------------VHGVELCVETFGDPADPPILLIHGAAASMDYWeaFCELLAAGPRYVVRYDHRDTGRS---VSYPPGKPGYTGTDLVRDPVGILDALGIGRAHVAGLSMGGGIAQQLVLDFPDRVRSLTLIATSPLGDDLPPAA------------------------------DRIKAAFATEATPTDWSDPDAAVDALIAAEKLYAGTL-----------PYDEPARRALVRAMIARTTDLESAMTNHWILPDDDTPPLRPRLGEIRVPVLVLHGTEDPLFPPAHGEALAREIPDARLVLlgAGHELPEPTWDTAVPAILA------------------------------------------------------------    
 761 UniRef50_A1TED7        Alpha/beta hydrolase fold n...   131     6e-29  1   8.6%  87:422     3:300     --------------------------------------------------------------------------------------DQPPPIDGVRRHFVTARGVNFHVTEAGPEDGRPVLALHGWPQHHWVYRSLLarLPEPGLRIIAPDLPGYGWSGPAPHKWAK------EDVVSDLVALMDAMGLDRVLLIGHDWGGYIGHLLAMRVPERIDGYLALNIAHPWVPPKVMAPHLWRFLQYQPLV------------------------------------------AFAGVPLQRHTSFLKRAYKLAAPKLDPQTVRVYSERFRDPVVARTARDTYRTFLLREAPAAIrgERRRSKVPTRALFGRDDAAIHPSLAAAETARADDYTIDVVDGGHFILDEQPELVRAKLIA-LAEEFPAR---------------------------------------------------    
 762 UniRef50_C9YXP4        Putative hydrolase n=1 Tax=...   131     6e-29  1  10.0%  99:397     4:252     --------------------------------------------------------------------------------------------------VTTPTGVSLNYDTFGDPDAPPLLLIQGLGAHMLGWhdFCRLIADAGFHVVRFDNRDVGLSQKFP-----QGGYTLADMANDAASLLTALEIPAAHIVGQSMGGMIAQQLTLDHPTQVLSLALIYTAPN------------------------AGFLVGRDLVDERTQRPRARDQDEAIALYLDNEAACASPG-------YPQDIDWLRELGGQTYRRDYDPDGIV----------------RQMEALDNSPDRAPHLHRITVPTTIVHGDGDQLIDSGASQAMHELIPDSTLTIHHggH----------------------------------------------------------------------------    
 763 UniRef50_A5G2W2        3-oxoadipate enol-lactonase...   131     6e-29  1  11.3%  99:471     2:313     --------------------------------------------------------------------------------------------------FVMAGDINVHVRIDGRDGSPPLVLLHSLGTDHRVWDDQAAALA-RSFRVIRPDMRGHGLTESTPG------DYTMAQLAGDVAAVLDALGVGAAVIGGISIGGMIAQEFAARHPGRTEALMLCDTAMAIpeSWRERVASVRGQGISAIEDGVLARWVTPGFIDAPPARGLRAMLRGTSIDGYAGCAMAIAMADLGPTTRRLKVPALVLVGDGDVSTPVASAQALMTALVEAGAPDVRFAVIEN----------------------------------------AAHIPT-------------VEKSADVTRAMLDFLA---ARGGGDAYEAGLGVRRAVLGPAHVARSLDGATEFDRDFQRFITETAWGKVWT--    
 764 UniRef50_C2AN24        Predicted hydrolase or acyl...   131     6e-29  1  10.6%  99:427     4:288     --------------------------------------------------------------------------------------------------TIAVNGRWARVRAEGDESKPTVLLLHGITRSLEDWDPQFESLSGD-FRLIATDLPGYGWS------APHPDGAGLPALARGVGETLDALKvgPVHVVGNSLGGAVTMTLLTQRPEQVATITLVDSAGFGKEVTPLLRLLGLPLIGK--------------LMATTPSRLSAIIQERLIFADKSFATRARIDHAMKIARETDAGLTTWRTADSLGSVLGGVQQEWR-------------------------DELQATIAKTPRPTMVLWGDRDKILPPKHLSAARALIPHAEGhiPGVGHMPQLECAPEFDALLTDFLERNATPELHPAA----------------------------------------------    
 765 UniRef50_C4LLK3        Putative hydrolase n=1 Tax=...   131     6e-29  1  12.4%  83:413     5:310     ----------------------------------------------------------------------------------PTNPSAIELDGPWTHHLYHSRGVRIHCADSGSPDAPLVLLVHGFAGGWFDWQSVLPLLAED-HHAVAIDLRGYGLSDKPP------HGYDLGTTALDIAGLVLALGHTSATVVAHGESVPAAVIAGLTEPQRIKRVVALNPSHPRQNVDVVTRHPIRHRDRIKVGMATRFPRLGERWLKADHSLFLEQIVAHLAGSAFRYSDSFDEYLAIRRFAMGVERV------------------AYYACLRHRKAAVATMPRGTAQTVLADWSGWNELECSTPPAVRVLHGAEDPLITASKTAR--ETIADADhvVPSAGHYVHMEAPHAVRDAVAA------------------------------------------------------------    
 766 UniRef50_Q5V106        2-hydroxy-6-ketonona-24-die...   131     7e-29  1  12.2%  39:416     5:349     --------------------------------------ETQSVLFIVSHGTLIRPALDSLRMSGGTTVPEDRALSPDDWPHPAERRYADAQARLADhrVTETDAarVHYLTGGNPDGDPVLLLHGVGTPAATWLPLFPALTDE-YRVYAPDRPGLGLSAAPSYRDRDLRSF----LVTYLLELLDDLGIDRPHMVGNSHGGLQSFLLAIDH-DRVDRLQLVGAPGGVSRDLPLLFRLLTVRGLNRVLLWLLNRGDPVENARQSMQRV------------------------------------NVVDGSAIPEALYELLAAGQALPGRPESQRSLATAQGSYGRAHPLFDIRNEIVRIDRPTQFVWGTEDAFYPPAVGRPVTEKMSHAEFHELPggHTPWLEPNDDAVTLVREFLA---------------------------------------------------------    
 767 UniRef50_P33912        Non-haem bromoperoxidase BP...   131     7e-29  1  12.0% 100:415     5:273     ---------------------------------------------------------------------------------------------------TTRDGVEIFYKDWG--QGRPVVFIHGWPLNGDAWQDQLKAVVDAGYRGIAHDRRGHGHST------PVWDGYDFDTFADDLNDLLTDLDLRDVTLVAHSMGGGELARYVGRHGtrLRSAVLLSAIPPVMIKSDK----------------------------------------NPDGVPDEVFDALKNGVLTERSQFWKDTAEGFFSANRPGNKVTQGNKDAFWYMAMAQTIEGGVRCVDAFG----YTDFTEDLKKFDIPTLVVHGDDDQVVPiaTGRKSAQIIPNAElvYEGSShmVPGDK-EKFNRDLLEFL----------------------------------------------------------    
 768 UniRef50_Q31RQ3        Putative uncharacterized pr...   131     7e-29  1  10.8%  91:423    36:330     ------------------------------------------------------------------------------------------AAHDPIDRFWDWQGQSIRYWQLGDRGAP-VLLVHGFGACCEHWRQNVEAIAQHS-TVYVIDLLGFGQSAKPD---PIAVNYGIELWAQQIEAFRQAVIGQPTRLIANSIGCVVALQAAVDQPEGYAALLLIDCALRQIDDKKLA------------------------------QQPLGRRLGRPLLKAAVRQRGLSNWLYRQLAKPSIIERILKLAYPSSERIDPALVEALYAATQSPGAAGVFW----AFINLFDAPLAEQLLPQVQAPVTFFWGDRDPWEPIALGRQLADFPSVKAFVPLAggHCPHDEAPKQVNPQIVAWLQEGVAAAG--------------------------------------------------    
 769 UniRef50_A5V6H5        Alpha/beta hydrolase fold n...   131     7e-29  1  10.8%  84:417     2:276     -----------------------------------------------------------------------------------MRKSMSSAELARMPSFTTGRP-RIAYEAEG--HGAAIIFIHGIGGDRSNWDRQMAHFGDR-YRVISLDVRGYGESD------NFDGSLTFDAMAADVAAVLDAENVDRAHLVGLSMGGMIAQWFWHRHPERVTSLTLADSTVGPAASLTEEQLDAFLESRRAPLLA-------------------------------------------------GATMAEIAERNTPSLLSEDAAPELLVEARASFAGVPATTYLRSLECVTRFAGV-PDFGAMTVPVLVMVGESDRSLPVPLSRELAAKIPSAELHIigAGHLSNMETPGRFNRLLEDFLAA--------------------------------------------------------    
 770 UniRef50_B1J5X6        Alpha/beta hydrolase fold n...   131     7e-29  1   8.8%  52:424    92:397     ---------------------------------------------------NVVAAWQYVDVMARAFDAMKRVGKTDTNPPLSYSPGPKPSGKETVGRYITVDGIRVYYEEAG--EGRPLVCFHAASQDSLMYRHVLDGLSDE-FRVIAIDAPGHSKSELPESGPFH----SLTRHAE----------------------------------------------------FNEHLMEALGLEKPAIMGCSMGGNLVLELGARRPDAYSAIISAEGADFTPTVSEFFLDMLLMNGPEIIGAWSRSMTGNRTPPDRAKEVVWQISRT--TPQVMKGDLTGYANF-------DQRQQVGKIKAPVLLLRGDADWLVYQEKVEETASRIPGSKIAVLAggHYPMTENPLEFCDTVRAFLREAGHGHAD-------------------------------------------------    
 771 UniRef50_B0FSQ0        Epoxide hydrolase n=1 Tax=P...   130     7e-29  1  10.3%  99:417     9:328     --------------------------------------------------------------------------------------------------FKTSRGFNYHYFYSPagPRPTLFFLHGNPSVATDWAQIVPHFTSRGYGVLAPDMLGYGDTDKPTDPAL----YVSSGMCRNLVDLLDHEGLEKVIAIGHDWGCLSVSRLASYYPERVIAYAFLAASFIGPIPPIP--FDAFLEYIKQQVGYELYGYWKFYSEPGLDQFVRDHLDTFLSILWPSDPAVWKTRLAPTGALKQSALEGWTAPLA--PYITEEFKKHHVEVFNKNGF-EAPACYYRIMTSGLQAEDDKQILPERAvpVFFGAANKDYICIPALAYAtkDEWKDHKITiyDADHWLILSHADQVSRDLEAWIEG--------------------------------------------------------    
 772 UniRef50_Q1D796        Hydrolase, alpha/beta fold ...   130     8e-29  1   7.5%  99:418     3:260     --------------------------------------------------------------------------------------------------TVTVDGVPLHYRDEG--KGPPVLLLHAFPLNGSTFDKQVKALSGR-YRFIIPDIRGFGESALGDGPTE------MFRIARDALSLLDALNLDTVVVGGVSMGGYAAMALLREDAGRVSGLVLMDTQATADDAEGKARRETSAIQALEVGVEPIIQAMLPKMVAAGPDS------------------PVAQEVAALMRAASPASVAAALRGMALRPDSKDMLARYAG------------------------------------PALVIVGEHDALTPPAKAKEIAGLISGAKlvIPDAGHLANQEQPDRVNAVLDAFLTSL-------------------------------------------------------    
 773 UniRef50_A4S5M0        Predicted protein (Fragment...   130     8e-29  1  15.6% 118:418     1:280     ---------------------------------------------------------------------------------------------------------------------RAIVLIHGFGVSSFQFRDNVRALGER-HRVYALDLVGFGTSDQPDVP------YAMEFWRDQVIDFVENVVGEPAVLVGNSIGSLAAVHVAAESPKTTAGIALINCAGGMNNKVKRLDGDFDGYGW----------------------QYKAVVPIFSVVLAIIDAVLKVEPIAKPLFESVRGEENVRGALANVYMNPSRVDDGLVNSICGAANREGAFKAFVNILTGPAGPRPEELMPRVACPMLILWGSKDTITPLdlGQYFLN-LPKTriQFEGEGHCVQDDNPKLVNDALTDWVAAL-------------------------------------------------------    
 774 UniRef50_B2GIX1        Putative hydrolase n=1 Tax=...   130     8e-29  1  12.3%  95:419     5:290     ----------------------------------------------------------------------------------------------FETHRIPVPGATIHVRTGGR--GEPLLLLHGFPQTHAMWHRLGPALARDHRMVVA-DLRGYGESTA------DSEDFSFRAMAADMVAVMRHLGHESFHVVAHDRGARTAHRMTLDAPDAVRSVALLDILPTPTVWEL--MDDWLAIRYYHWLFLAQPAPMPQGLISAGPLTFLHAALGGLSGTP-----------------------------GSLDTFDPEALAAYEAAAQNPSVVAAWCADYAAAAGVDREHDDADRGTSRNLPALLLWGDDGVvaQTDPLEAWRPWLPRITGRAVPTGHFMVEERPDEVLAEIQEHLAAAA------------------------------------------------------    
 775 UniRef50_Q1DCJ1        Hydrolase, alpha/beta fold ...   130     8e-29  1   9.0%  98:419     8:267     -------------------------------------------------------------------------------------------------PIAELNGQGIYFEDSG-GNGRPIILGHGFLMDHRMFDAQVAALAPE-FRVIRWDARGFGQTRWDGKP------FTPWDSAADCVALLDHLGIERAVVGGMSQGGYSALRVALKHPERVRALVLLSTRGTNDDAPTRAA-----------YQQSADIWETQGPIDPLVQGLAQAIIGDTRYFDTWLPRWRQIPGG---------------------------------------------HFANAIRSLIDRDDISGRLTEIRCPAIVFHGLEDHGMPPVHGEHLHQTLPGsrFvvPGAAHAANMTHPEAVNPALLEFLRAHA------------------------------------------------------    
 776 UniRef50_A5V6J3        Alpha/beta hydrolase fold n...   130     8e-29  1  10.3%  91:421     2:285     ------------------------------------------------------------------------------------------PREMREAVVELPNGLSTHYYEW-PGSGPKLVFLHPSAGYGRMWEWTVDALGDR-FHVFAADQRGHGFSGRPEG------DYSAEEYADDVALFMDAVGIERAVIVGHSLGGRVAQVFAGRYPERIEGMVLVASPHLSNFHATREAARAVLANAADTLG--------------HPEEFASVDEALAFMKARWPWSPESDGSLRHRIAYNFEHRDGGRVAPRYD------------------------NIRVAQGLAHLTANLRPYAARAECPVMILLAKQGSLTRP-QAEELAGYWRDARIVEIDGTYALQMDNPQALAGAIADFAATVGAG----------------------------------------------------    
 777 UniRef50_C1DKB2        Alpha/beta hydrolase fold p...   130     9e-29  1   8.7%  85:419    30:318     ------------------------------------------------------------------------------------RTRRAEREHPPSGRFVTVDGVRLHYLDQGR--GSPVVLLHGNAVTAEDysGVFER-AAKVHRVLAFDRPGYGYSERPLGTL-----WTASRQAELLHRALRQLGVERPVIVGHSWGTLVALKMALDHPDDVAGLVLLGGYY--------------------------AATVRLDVLAAAPPAIPLLGGALRHTLTPILNGSLLQLTFKVMFAPLPVPERFRREFPHQFPLRPLQIRA---------------ESQDAVTLIPAVITMRQRYSELRLPTVIMAGTRDLVVDERHADWLHRRLPGSHllIPEAGHMLHYAAPGQVVAAIERVAAEAP------------------------------------------------------    
 778 UniRef50_Q0UV86        Putative uncharacterized pr...   130     9e-29  1  11.5% 103:416    42:334     ------------------------------------------------------------------------------------------------------DGIQLQYSTHGSASSPPLILLHGFTGSSAVFTRNVPTLSKDYY-VIVPDLRGHGLSGKPKA------GYHVSRLAMDLKNLIEHLKfgEVSAIGTSLGAAILWSYalFTTSTFKKLIFVDQAPLQNYTSDWGKEFGNRGCNSAEALDRVQHLLEFGPKAAHIGTISACLGYRSHPQPGDP------------------------TIGSKEWVEDED-------FFLGEAMNGDGRWYGKLMSDHTSLDWRDAIAQNFGpvLVVASTRSGCFPAAGPLKVVDLVNGGkvAVEWGGHWCYWEKPDTFNELVLGFLK---------------------------------------------------------    
 779 UniRef50_D2B540        Alpha/beta hydrolase fold p...   130     9e-29  1  10.9%  96:417    10:282     -----------------------------------------------------------------------------------------------VTRTVEVGGIATTVIDTGEpsAPPLLLLHGSgtALANWRPIIPAFAASR-RVIAPDQLGFGGT-----ATGEARTYGRASWTRHALALLDELGIDRIDIIGNSMGGAIALSLAAARPYLSRRIVLMGS---------------------------------MGIAMPLPDGLDGVW-------RYEPSIEESRRVIGLFAHNRDLITDDLVQMRYEASIRPAVRDSWQAMFPPPRQR-----------WVDDLALTGAELNAINQPVLLVHGRDDKVVPWSSSAALLDLLPDSRLHVlgCGHWTMIERTTDFLAVVEPFLAA--------------------------------------------------------    
 780 UniRef50_C7RTU4        Alpha/beta hydrolase fold p...   130     1e-28  1  10.2%  83:434     8:328     ----------------------------------------------------------------------------------APPGQQADPGQVGGEAMARANGIDICYQTLGDRGSSAILLIMGLGMQLIAwdAFCESLVGSGFRVIRFDNRDVGHSTHIDWRQRPrsPYRLSDMADDAIGLLDALQVARTHLVGVSMGGMIAQCMAARYPDRLLSLTSIMSSSGN-----------------------------WRVACGKPAVLRQL---------------LRRPARPDDPSSQLDHLMRVFGVIGSPGFPSDQADLREQLARAIRRANYAAGHYQQLLAIIAAGDRRRELAKIRTPTLVIHGADDPLVPVAAGYDTAKHIAGARLAIIRggH----DLPPAVQKLLLQAVVSHCRDAEASAASGLGPRR---------------------------------------    
 781 UniRef50_Q07KG3        Alpha/beta hydrolase fold n...   130     1e-28  1  11.3%  89:433    47:345     ----------------------------------------------------------------------------------------AERRNPPAGRFLKIDGVWLHYIERGA--GEPLLLLHGNGSMIQDFdgLID-LAAANHRVIVFDRPGFGHSDRPRDVT-----WTPALQARLFKCALEQLGIRQTLVLGHSWGASVAIAMALDDPDLVRGLVLASGYYYPTLRADVVALSAPALPVIGDLASNTVSPMLSRLL--------------------WPLLMKHMFGPARVPEKFGGFPKEMAVRPSQIRASAEE----------------------SALMIPDAAEYRERYAELKMPVVIIAGEQDRLVDVdqSARLHGDIPGSKFhiAQSGHMIHQTATADVLAAIHEVDARAGAHRTIDGAAPALTQ----------------------------------------    
 782 UniRef50_D1AAH2        3-oxoadipate enol-lactonase...   130     1e-28  1  10.4% 107:471    10:315     ----------------------------------------------------------------------------------------------------------LHHRIEGPAWGEPLLLGPSLGTSLAVWEPQVGALA-RTHRVV------------------------------------------RWDLPGH-GGSPAALLPAGSSIAELGRLVL-------------DLADSLGIGRFAYAGISLGGAVGAWLAVHHPDRVGSLALVCSSARFGDPDAWRRRAAAVRAEGTGPLADAAAARWFAPGFAADPVAAALAEEL-TGDLRAADPEGYAACCEVLAGYDLRDRLGRITAPTLVIAGRDDPATPPAHARELADGIPGATLVEvgAAHLAGVQRPEAVTTALLGHLG--AGSADGPRHAAGTAVRRAVLGDAHVDAALAGADEFTAPFQDLITRYAWGEIWT--    
 783 UniRef50_P27747        Dihydrolipoyllysine-residue...   130     1e-28  1  11.8%  53:415    73:372     ----------------------------------------------------VLPVKALLGVLAPAEVSDAQIDDYVAAYETPADDAGEEDAAAAYQ-FADVDGIRVRYARKG-GGAETVLFIHGFGGDLDNWLFNLDPLADA-YTVVALDLPGHGQSSPRLAGT------TLAQMAGFVARFMDETGIEAAHVVGHSMGGGVAAQLAVDAPQRVLSVALVSPVGFGDAVNSGYTEGFVSAQ----------------------------------------SRRELKPVVELLFADAGLVSRQML----------DDLLRYKRLDGVTEALTALGQGL--FGGGRQSEQPGQRLANSGKRVLVVWGGQDQIIPAAHAEAAPPG--ATVKVFADAGHMSQMEKANDFNALLKKHL----------------------------------------------------------    
 784 UniRef50_Q8U671        Haloalkane dehalogenase n=2...   130     1e-28  1  13.1% 100:420    25:303     ---------------------------------------------------------------------------------------------------VPAFGLQIHTVEHGS--GAPIVFLHGNPTSSYLWRHIFRRLHGHG-RLLAVDLIGYGQSSKP------DIEYTLENQQRYVDAWFDALDLRNVTLVLQDYGAAFGLNWASRNPDRVRAVAFFEP------------------------------------------VLRNIDSVDLSPEFVTRRAKLRQPGEGEIFVQQerFLTELFPWFFLTPLAPEDLRQYQTPFPTPHSRKAILAGPRNLPVDGEPASTVAFLEQadTPKLLLTFKPGFLLTDAILKWSQVTIRNLEIEAAGAghFVQEEQPETIARLLDAWLTRIAG-----------------------------------------------------    
 785 UniRef50_A9AT93        Alpha/beta hydrolase n=24 T...   130     1e-28  1  11.1%  84:425    20:306     -----------------------------------------------------------------------------------LASAPHAFAAPNSYTVTSADGVRIAVQENGNPNGQPIILVHGLLGSRLNWDAQVRSRELSRYRIITYDLRGHGLSDKPAGPEPYHDG---SRWGDDLAAVIRGSHARKPVVVGWSLGGVVISNYLAKYGDsiAGAVYVDGVV----------------------------ELAPGQIVAHPDVYRDMNSAD--------------------LKTHLDGERTFVGLCFNRRPDADTFARLLAN-------AAMASWDMQREVPAMTVFATEGLAKARVPLLFIYGGRDVLvtQATLARAAALNPhvSHVYADAGHAPFIEEPERFNRDLDAFVRSASQQGRDV------------------------------------------------    
 786 UniRef50_Q2JB56        Alpha/beta hydrolase fold n...   130     1e-28  1  11.9%  96:448     2:320     -----------------------------------------------------------------------------------------------ERFVRTRDDLMLWTESFGRSADPAILLVMGGPAQGITwdALCRMLADSGYRVVRYDHRDVGLSTKTDSRLSC---YDLSTLAGDAADVITGLGLASAHVVGQSAGGIVAQLLALEEPSLVRSLVLLSCSPDANGDVHLPPLTGLPGPERPLLDHVAA-------------------LNDNPPTTRAARVAAAVRGWRVLVGAGAPFSETYWR------------DLVERSMRREAGSDLGSNHLRALDR---TPPLTARIGAIRVPALVVHGRRDTVFPLAHGETLAREIAAARLVVVdlGhfPPQWCP-LVHGLVTRHLREAGAHPAGQVRSpaGGTSDRRGATAADVPPAA-------------------------    
 787 UniRef50_A8L8I4        Alpha/beta hydrolase fold n...   130     1e-28  1   9.7%  99:416     4:275     --------------------------------------------------------------------------------------------------VTVRDGTELYYRDWGR--GPLVVLLGAAVMNSRMWESLAYHLAGNGFRCVAYDRRGTGRSDWP------WSGYDYDTLADDLAHLLEHLDARDAALVGYggGGEAVRYLARDGDARVSSLVLASSTTPIIMK-----------------------------TADNPDGTDVAVFEQTIAAAEQDRARWVAELVAPFFGGSDGSPTRLG------------LSSYGATFFESVALDSSLRATVEIYRTLFMADLRADVARVVVPTLVVHGEADVVAPIDlgRRTAALLPNSTFvyEGAAHGLFATHADRLNRDVVAFIA---------------------------------------------------------    
 788 UniRef50_C7Q1L8        3-oxoadipate enol-lactonase...   130     1e-28  1  12.3% 156:471    11:307     -----------------------------------------------------------------------------------------------------------------------------------------------------------GPSDAPPLLLAPSLGTSLAVWDGHVKALARDHRVIRWDLPGHGGSAAT--------------LVGERATVAELADLVLALADHLGLERFAYAGISIGGAVGAHLAVRHPDRITSLALVCSSAHFGEPQA-----WLERAARVRREGTEFLAASAPARWFTSDFPGGEALEKLISDQRAADPVAYAACCEALADCDLRGELDRITAPTLVIAGREDTATPPPHSRELADGIAGAglEIPGAGHLAPVERPRPVLDALRAHFGAAPSDDAERHAAGTAMR-RAVLGDAHVDRALAAATPLTAGFQDFITRYAWGEIWT--    
 789 UniRef50_B0SY51        Haloalkane dehalogenase n=3...   130     1e-28  1  12.4%  76:413     2:294     ---------------------------------------------------------------------------QVLRTPESRFAGLADWPFAPHYAEITDAglRLHYVDEGPRDGAPVLLMHGEPSWAYLYRKIIPALAARGHRVIAPDLIGFGRSDKPAAR----GDYTYERHVAWMSAWLEGLDLRGLTLFCQDWGGLIGLRLVAAFPERFAGLVIANTGLPTGAGMTDGFKA---------------------------------WLDFSQNVPEMPIGLIVNMGTGRDL-------------------TPAEIAAYDAPFPDETYKEGARQFPMLVPVTPEHASVAENLAAWKvpVVTAFSDGDPVTRGGEA-IFQSRMPGAlhRTLRGGHFLQEDCPDDIVDIVDA------------------------------------------------------------    
 790 UniRef50_A7IBP7        Alpha/beta hydrolase fold n...   130     1e-28  1  10.6%  99:420    30:313     --------------------------------------------------------------------------------------------------RVQAGEVSLAWRQWGEGD-TIVLFIHGNLASK-DWIELAAPHFPRGVRTIAIDWRGCGDSDKPAALPDFS-NYSIAQHADDMLAALDALDIGFCHLATHSTGGIISTRMLLKQPERFGRVFSLSPVGPQGLAFTQE--------NIALFARMKASRDFARYIMALTAPSLFEPDSLAAGAPRFRSDIGDRAGLFDHIVEQVCAVSDGIWFGTPDTLTKEA----------------------------ASGALKALMGTITHPHLVVWGMNDGVIPEADLKEMAALMPHCRFvvPGIGHCLNLEAPEIYAGYFGAWFGGLSG-----------------------------------------------------    
 791 UniRef50_A8LQ44        Proline iminopeptidase n=14...   130     1e-28  1  11.1%  83:425    10:327     ----------------------------------------------------------------------------------AAEYLYPPLDPYDQRVLPVSGGHRIYVEQCGNPQGIPVVVLHGGPGGGCS-PAMRRYFDPDTYRIVLFDQRGCGRS----RPHASVEQNTTWDLVDDIEAIRTTLEIDAWDVFGGSWGATLALIYGQTHPDRVTHLIL---------RGVFLMTDAELDWFYGGGAAQFWPDVWKRFVNLIPEEERGDLIAAYNKRLFSGNMMEETRYARAWS-----AWENALASIHSEGLTGESPAEYARAFARLENHY----FLNKGFLDEDGQILRDLPRLADVPITIVQGRFDMICPPAGAWQIAEALPQTDLRMIpaGHALSES---GISAELVRVMDRLRYGRRPS------------------------------------------------    
 792 UniRef50_Q0RR69        Putative epoxide hydrolase ...   130     1e-28  1  10.3%  98:426     3:303     -------------------------------------------------------------------------------------------------TTVRTEVLDISVEVTGPADGPPVVLLHGWPDAARGWHGVQAGLNERGWRTIVPDLRGSGATRFRDAGTPRDGQA--VALAQDALDLADALGLGRIAVVGHDWGARAAYTLAALAPRRLTAIAALALAYQPRGSFTMPGFEQARAFWYQWLMFVDAGARAVREDPVGFARI------------------------------------QWDTWSPPGWFDDDEFAATAPAFTRPDWAAITLNAYRArpRDPRYDDLRRRLarLGVPTLMIQGGDDRCDSPAASAGQAAYFTGgrVVLDGVGHFPHREATAAVADLVADHLSVADGRARASG-----------------------------------------------    
 793 UniRef50_O28567        2-hydroxy-6-oxo-6-phenylhex...   130     1e-28  1  16.2%  99:271    11:173     --------------------------------------------------------------------------------------------------FFDSDGLKLRYFECG--EGEPLILIHGLGDCIEGWTFQYDDFS-KHFRVVALDLRGFGMSDVPESI-------SVEDFARDVKNLIDHLGIERANLLGLSMGGVVCMEFYRQYPEMVKSLVLANTLHKLPDAGRAMFEQRLKLLESSPDMTQIAEFIAEMSIHQDRPELKELV----------------------------------------------------------------------------------------------------------------------------------------------------------------------------------------------------------    
 794 UniRef50_A5WLF1        Epoxide hydrolase ephC n=18...   130     2e-28  1  11.5%  85:415     7:312     ------------------------------------------------------------------------------------ERESTWRTTMAEPHWIDVKggDLKALTWGPAGAPVALCLHGFPDTAYGWRKVAPRLAESGWHVVAPFMRGYAPSSIPA-----DGSYHVGALMHDALRVraAGGTERDVIIGHDWGAIAATGLAAMPDSPFAKAVIMSVPPSAAFRPLGRVPER------------------GRLLRELPHQLLRSWYILYFQLPWLPERSASW------------VVPLLWRRWSPGYHAEEDLRHVDAAIGTPEGRRAALGPYRATMRNTRAPADYADLNRlkLPVLYLHGHDDGCATSAFTHWTARVLpgSEvvVEHAGHFLQLEQPDKIAELIVAFI----------------------------------------------------------    
 795 UniRef50_UPI0001AF64BC hydrolase n=1 Tax=Mycobacte...   129     2e-28  1  11.6%  91:419    55:337     ------------------------------------------------------------------------------------------GAVTAVHRFVDAPgvRWHFVESGDASAPTVVFLHGLPDSWWQWHYALEGL-GRRYHCLAVDLKGYGQSDKRSG------DYRQAGVAAQLGALLGELGIGEFVLITHDRGSPVGDHLVAAMGARVRG---------YGRGQQHLWHLHPRLHPQEQLMQSAEAPAMLREARR--------------------------------------FVTTVYTWLTERPVAREDLTRTIEEFSHPGIATAVPRYFHSSSFRQEWVDRRTRlrAWTAPVLLLQGAHDPLQPREFYRelAMLPPGSDVHLFDTGHFWPFEAPRATVDVLTEFCDRVA------------------------------------------------------    
 796 UniRef50_B8H3S9        Haloalkane dehalogenase n=2...   129     2e-28  1  11.8%  76:421     2:302     ---------------------------------------------------------------------------DVLRTPDERFEGLADWSFAPhtEVTDADglRIHHVDEGPKDQRPILLMHGEPSWAYLYRKVIAELVAKGHRVVAPDLVGFGRSDKPAKRT----DYTYERHVAWMSAWLEQNDLKDIVLFCQDWGGLIGLRLVAAFPERFSAVVVSNTGLPIG------------------VGKSEGFEAWLNFSQNTPELPVGFILNGGTARD----------------------LSDAERSAYDAPFPDESYKEGARIF------PALVPITPEHASVEENKAAWAVLETFDKPFVTAFSDADPITRGGEAMFLAR-VPGTkhTTLKGGHFVQEDSPVEIAALLDGLVAGLPQA----------------------------------------------------    
 797 UniRef50_D0WK53        Alpha/beta hydrolase family...   129     2e-28  1  10.6%  80:418     1:304     -------------------------------------------------------------------------------MSAPADNSAVFLPGPWQHRLINANGCQFHIAYMgrNDRPLVLLVHGFPEYWWTWRHQIEAVALAGYEVAAIDQRGIGGSDKTPDSADGM------LLTQDLAAIVRSLGTSRAVVIGQGRGGFLAWSVAALEPDVVEGIMTVSAP--------------------------------------HPRTLQRLGTHLTLKTWRQVLKTLIPHYAAKRLTDEEVLKRLLTDWSAPGNAGAseAANYTAALRLPEAASISLEQLRWSYLSTSKINGREHMALTraTVWAVRGERDPLLP---ARAWRKDLEFAriEVPDAGHFVQEEQPSRVTDLVLEFLAQV-------------------------------------------------------    
 798 UniRef50_Q0BXR5        Hydrolase, alpha/beta fold ...   129     2e-28  1  11.7%  98:422     9:291     -------------------------------------------------------------------------------------------------HWTSPDGLTLYAKAYGPEDArtVLCLHGLTRNHQDFEPMIAAL-PRHHRYIAVDVRGRGKS----AYDPKPDNYQPPVYARDMFALLDTLGIARAALIGTSMGGLISLLMARSAPKRVAGIVLNDVGPVVEKAGLARIGSYAGKVAPVTDWESAAAAVKTLQGAAFPDMPEGRWMDFA-------RRTYKELPSGEVVLAYDPGIARSLGKVKPGPVTNFILWRLFAASR-------------------------------KAPLLIIRGETSDILSAGTAEMMVRRHPDARltVPRVGHAPILDEPQAVS-AISDFLSRLETTA---------------------------------------------------    
 799 UniRef50_D0VYN1        Putative uncharacterized pr...   129     2e-28  1  13.0%  98:420     2:261     -------------------------------------------------------------------------------------------------PTSQVGDLKRHYLEQGR--GEPVVLIHGNTSSSVWWEPTLARLGDD-YHLIAPDLRGRGDXEGPSA------DWTVEMLADDVRGLVKHLDVGPSHLVGHSLGANVALQIALDQPGWVKSIVLLNPGWVAGDMPAE---------------------------LADPERVRQMV-------------------------ENKDLLKLALRGVAMLHPVDEQWQR-LEAASLKQRDEASLRGPLALKEWVVADRLHELA---SIPTLVVRGAQDQYltQAVCQEILDRLPDARYvlPGASHSPNVETPDAWVALLREHLARVSA-----------------------------------------------------    
 800 UniRef50_A6GRP2        Putative short-chain dehydr...   129     2e-28  1  11.0%  92:426     8:306     -------------------------------------------------------------------------------------------QRQMSQMVVHSSDVELAVTSHGEPHNPTIILVHGYPDSSSVWDPVVDIL-KHHFHVVTYDVRGCGKSTEP----GWMWDYSLGKLSEDLQAVMRAVCpqPVHLVAHDWGSIQTWESVTDHRLKSRIASYTSISGPCLDHIGMWMRNSFAANNRKALGNVMG-------------QLMHSWYVYAFQLPFLAPAAWKMGLDKAWPGIVKKM---------------EGVEHDANPTQRKDGVNG-INLYRA---NMLPRISNPRLRQTEVPVQLLVPTKDKFVTTALVESCYPYAHNVWRRDIDAQHWVVQSHSEWVANCVSEFVEFAETGRENPG-----------------------------------------------    
 801 UniRef50_UPI0001C31954 alpha/beta hydrolase fold p...   129     2e-28  1  11.8%  99:421     2:296     --------------------------------------------------------------------------------------------------RIETNGVELWAEAFGAEDAPALLLI---GNSMLTwdELCERLAAGGLRVVRYDQRDTGRST---TVDPDAPDYSLRDLVGDAAGLLDALGAGHAHVAGFATGGWIAQLLALDRPERVASLTLIATRPTAPGRADADLPD------------------------HDPELMKWIMSTPEPDWSDRAAVVAHMAESGRRIAGSRPFDDAAASERAGRVFDRACAAAPELAAANPRALQRSNQIASVFAVIKTKPRWRERLGEVAVPALVIHGEDDPFFPLGNGEALAREIPGAelTLPGTGQELPRDSWDEVVPAIVRLTRAAAPA----------------------------------------------------    
 802 UniRef50_Q82FK0        Putative dehydrogenase n=3 ...   129     2e-28  1  12.3%  96:453     7:333     -----------------------------------------------------------------------------------------------RERWVRTGGIELCVAELGDETQPTVVLVHGYPDSKEVWSEVAGRLSER-FHVVLYDVRGHGRSTAP---QPLRGGFTLEKLTDDFLAVADAVSPDrvHLVGHDWGSVQSWEFVTVKRTEGRIASFTSMSGPSLDHFGHWIKRRVKRPTPRRVGQLLGQGA-----------------------KSWYVYLLHTPVLPELAWRGPLGRRWPKILQRVEKVPA---DGYPTPSLPSDAAHGAW-LYRDNVRARLSRPRTDAYA--HAPVQLVTPLGDAFLSEKLYDELELWAPQLVRRTLPAKHWIPRTRPDQLASWITEfiASEGGRPAPVASGPYADRFGGQLVLVTGAGSGIGRATA--------------------    
 803 UniRef50_A3NJ20        Alpha/beta hydrolase family...   129     2e-28  1  12.1%  92:447     2:313     -------------------------------------------------------------------------------------------ATVRDTLTVRSGDVRLAVYLSGPRNAPPVILVHGYPDSARVWERVREHL-DKRFRVIAYDVRGAGASDAPKRRA----DYKLSVLAQDLLAVAdtCGDRPFHLVGHDWGSIQCWEAATDPAVKGRLASYTSISGPCLDH------------------------------------VFRAPLNLRQTLKSWYIAFFHLPLVPELVWRLGGAKLWPLWLRRTERIVAD---------ADPDQLRNGLNGLKLYRANFIERARRPRERYAQAPVQIIVPRYDRYVTPALTRGLDRWLGAHRRDEIAASHWTVIRDAALIAARIGRFAAAHEPKRHAIAAGGapRAKQRTNQQASAGRRL--------------------------    
 804 UniRef50_C9Z486        Putative hydrolase n=1 Tax=...   129     2e-28  1  12.5%  96:414    16:308     -----------------------------------------------------------------------------------------------RERIVPVDGIEVWAEEFGAPEHPAVLLVMGAQAQGVQwdGIVRRLVDGGRRVIRYDHRDTGRSSVVDFAVRP---YTVADMASDALAVLDAFGVEQAHLVGASLGGIIAQRIAVTDPERVLTLTSLSSQPLGTDTAAAVGRDLAGEPPEPGELPPPSAELLAVLATTLPDPEAGPDRYLAARLPLW----------------------RVLHGSVLPFDEEEYRRMERRVYERARDLAAGFHHTLAGSAGGDDT---AALASVTAPTLVLHGTEDPMFPPAHGEATAAAIPGARLVVVEggHTLPAALDARLAEEILRH-----------------------------------------------------------    
 805 UniRef50_D1RS09        Putative uncharacterized pr...   129     2e-28  1   9.6%  96:417     7:301     -----------------------------------------------------------------------------------------------RRGLTEQGEVPIAYEDWGNIAHPPLLLIMGIGAQMLLwdDFCRELVDKGFRVIRFDNRDVGLSGKTQggWQTQVPYSLEDMAQDAVHLLDHLQIAQAHVLGASMGGMIALVLAAEYPQRVKSLTILFSSTNQPLLPPPAPSLLKLLLRRPPA--------------------------NATQQQRCQSLKDGLRALG----------------SPAYPLEEAELDMLVRRLLKRGMDAEGLQ--RQLSALLGSGDLRRYSHRITAPTLVIHGSADRLVRKAGGVAVARAIAHSQlvIPKMGhlPPQLRPQLI-EMISAHAAR--------------------------------------------------------    
 806 UniRef50_C4RBW1        Hydrolase n=1 Tax=Micromono...   129     2e-28  1  14.1% 114:417    42:312     -----------------------------------------------------------------------------------------------------------------APGAEPALYVHGLGGSSQNWTDLAGLLADR-FDGQAIDLPGFGRSE-------PSRRYTVPAFAEHVVRWIEHSGRGPVHLVGNSLGGAISVRVAALRPDLVSTLTLISPAL----PFLDFRRSLQGRMLPLLAVPRGERLVGWQLARLDPKVMAQQVMESCVADLTRISEQRRQEALEEILVRYQAAHYASVYVRTFRGLVSSFLRSYL----------------------PGPDSLWRLAAAVRAPTLVIGGSEDRMVDVRVAPQAARVIPDSRLLMLRggHVAQLEVPRTVARAVLGLLTE--------------------------------------------------------    
 807 UniRef50_B0U9F3        Alpha/beta hydrolase fold n...   129     2e-28  1  11.6%  86:448    41:354     -------------------------------------------------------------------------------------AREAERRHPPIGRFMTVDGVRLHYVERGQGD--PLVLIHGNGTMIQDfsGIVEDLATR-HRVIIIDRPGYGYSARPRALWTPRAHATLFQKA------LKRLGVSQAVVLGHSWGSLVAVALALQAPHLVRSLVLASGYYYPTLRADVVLFSPPAIPVVGDVMRYTVSPVVSRLIL--------PGLFKAMFAPAAVPERFDEQMPKELMLRPSQLRAAAEDAALMTPVTVGLQEHYRDLML---------------------------------PVVIVTGADDQIADvrQSERLHRELPDSELIVVPggHMIHHLAPDQVIKAVA---RAAEQTSVKAGHERRPVKVGANEASFRMSKPAA-------------------------    
 808 UniRef50_A8TT96        3-oxoadipate enol-lactonase...   129     2e-28  1   7.9%  98:415     2:258     -------------------------------------------------------------------------------------------------PHATINGIQLHYKVSGNADGPTLLLSNSLASTLNMWDPQMPAF-EKRFRVVRYDSRGHGQSEAPDG------AYSIDTLTADAVGLLDHLDIAKTHFCGLSKGGMVGQRLATLHPGRVDRLILCDTASYMGPAELWEGRIQLAEDK--------------GMAGVVDATIERWFTESFRVNDPTAVATVRAMILGTPVAGFAGCCRAI------------------QAMDQRESIRSIAAPTLIIVGADDPSTTPDKAREIQ-----------------------EQIRGSKLVvpEAAHLSNIEQPEKFDQAVLHFL----------------------------------------------------------    
 809 UniRef50_B5HTP3        Hydrolase n=2 Tax=Streptomy...   129     2e-28  1  10.9% 117:420     3:246     --------------------------------------------------------------------------------------------------------------------AIPLVLVHGHPFDHTMWRPQIEAFSASR-RVVAPDLRGYGTSG------PASEVNRFERFAQDIEALLVELDVRACVVAGLSMGGQIAMDCYRRFPERIRGLVLADTFP----------------------------------AAETPEGVK----------------VRHAMADRLEREGMRGYADEVLEKMVAPYASPEVKSHVHGMM----TATRPASAAAALRARAARPDYRDLLTRVTVPALVLVGADDTYTPVADAEAMHAALPESELHVFeaAHLPNLERAGEFNAVLGEFLAKADA-----------------------------------------------------    
 810 UniRef50_UPI0001B52502 hypothetical protein SvirD4...   129     3e-28  1  12.8%  75:411    24:307     --------------------------------------------------------------------------SWIERQPDPVPYERLRREPEGQEAFlrPDGTVLRTIVAG--EGPTVVLAHGYGASLLEWNLVQATLVVGGHRVIVFDQRGHGGSTLGADGIGSGP------MAQDYVAVLEHFDVRDAVLVGHSMGGFLALRAVLDHPPVARRL----------------------------RGLVLFATWAGRIYDGAPHNRLQIPLLEMGIL--------------QRLARTRTGGILFGAAQCGKRPSPAMIEVFLEVFLQQAH-----ASLVPIVRAFAREDRYPRLAEIAVPTVVMVGSADRSTPPSHARRLAAGIPGARLivPDAGHMLNWEDPHALVQAV--------------------------------------------------------------    
 811 UniRef50_D2S4E3        AMP-dependent synthetase an...   129     3e-28  1  13.8%  67:450     1:341     ------------------------------------------------------------------MTAPDTTAEEAARLPPALPGLDPAWSRLVAVADSTGVKHTWHVLDNGVPDPvtLLCVHGNPTWSYLWRRLLAA-APPGWRVVAPDHLGMGFSDRLPGPRP------LRQRVADLGDLTAALGvgPVVTVGHDWGGVLSLGWALAHRQDLRGVVLANTAVAMPEGDLGPALIRMAS----------APGVRATVTVGTPVFVRATTALSRPALPAGVRSAFAAPYRSPARRRSVGDFVADIPFSPGHPSRPA------------------------------QEAIAEGIRTLDVPALLLWGPRDPVFGERYLADLRSRLPQARLhyEGASHLLPEDAP-RYAEAVAQWVTDLGTGTTEPPDRPAGHAARRLWtdERSGDDAPAV-----------------------    
 812 UniRef50_Q0C8P3        Predicted protein n=1 Tax=A...   129     3e-28  1   7.7% 155:415    11:266     ----------------------------------------------------------------------------------------------------------------------------------------------------------HARTFKKDAPTDSIGPYTFKSLADDFVSLAKQLGCVNIIVGGHDWGCMIAYRMATWHPDFVTHMFTACVPYLLPNAEWVEIERIAKIYPTFGYQLQFGSGV-IEQETQTKEGIRKFLNSTYDGR--------TSDNQSAIDATMGIDFDLVAQLEKTALLSEEELEYYVNEFARNG-LHGACNYYRNHRQNFLDDSYFTraAVIKCPTLFLCATEDKFIRPEMTEGIKKSISNLTFKKINASHWILWEKPGVVNGVLREWF----------------------------------------------------------    
 813 UniRef50_A4FIX9        Oxidoreductase n=5 Tax=Acti...   129     3e-28  1  14.9% 106:420    16:280     ---------------------------------------------------------------------------------------------------------EVRWEKLGAPDAEPLVLLHGTPFSSYIWRDIAATLAHR-YRVHVWDMPGYGVSEMSEA-----QDLSLGAITSVFAELLDHWGLAEPLVVAHDSGGAIALGAHLLHNARYRRLALVDA-----------------------VALGPWGSAFFEVVGGHADVFAGLPL-----------------------RVHQALLREYVNSASSRGLHPATLDALAAPWMDEPGQAAFYRQlqRTNDPSFIDEIQGRYERIGIPVKICWGQDDTWMPEARGRELAARIPEATFhvPRAGHLVQEDNPADLTATLVDFLRGWGP-----------------------------------------------------    
 814 UniRef50_C1YV72        Predicted hydrolase or acyl...   129     3e-28  1  15.3%  90:415     8:279     -----------------------------------------------------------------------------------------SGDAWTLDRSFEAPNGTVRWARFGDGD--PVVLLHGTPFSSHVWHHVARALSAR-YTVHVWDMPGYGASAKHEG-----QDVSLAAQQAAFTALLDHWGLERPAVVAHDFGGAVALRSAVLDGAAYDRLALVDAVSVRPW-----------------------GSDFFRLVNANADVFAAL-----------------------PPHLHEALVRAYVSSAAHREPRGDVLDALVRPWLGADGQPAFYRQIAQADERH-TAEVEDAYADLDLPVLVVWGEEDTWLPLDRGRRLAAAIPGARlvLPGAGHLVQEDAPAELTAALLDFL----------------------------------------------------------    
 815 UniRef50_Q4KEJ0        Hydrolase, alpha/beta hydro...   129     3e-28  1  10.2% 113:417    58:305     ----------------------------------------------------------------------------------------------------------------GPAQAPVILLLHGLSADKSIWLRFARHF-NRDYRLLIPDLGGHGET-----AYAAHQDYRVPAQAQRLLRLLDACGIQRVQVIGNSMGGYIAAWLAAHAPQRISGLALFDPAGVEAPETSDLQHLLEQGKNPFLVRSRADFQHFYSLTMAAP-----------------------------------------------PWVPEAVLAAIAERYQARRGQLA-----RIFAELQASPPMEPELAKIQAPTLLLWGREDRLLHPSSAQVWAKGLPQAQVQLWEggHMPMVERPVRSARLYQQFLER--------------------------------------------------------    
 816 UniRef50_C9NEX8        Alpha/beta hydrolase fold p...   129     3e-28  1  17.1%  94:286     1:188     ---------------------------------------------------------------------------------------------MEDQSVVDVGDVRLAYRTWGDPFGSPVVLLHGLGGSSLSWEAVAALLGEE-WRVYAIDLRGHGESDWPD-------EYGFEQMRDDVLEFLDACELDRVGVVGHSMGGVVAYLLAEEHADRVERLVLVETPPPFPGQPAAEGPPGGPVDYDENAVDEVRAQVapRWAEELGQIVAPTLMIAGGPESTMPQARL-------------------------------------------------------------------------------------------------------------------------------------------------------------------------------------------    
 817 UniRef50_B8G485        Alpha/beta hydrolase fold p...   129     3e-28  1   9.9%  93:415    17:291     --------------------------------------------------------------------------------------------PFQISAYTTPSGV-IRYVDEGV--GPPVVMLHGNPTWSFFYRRLITALHNRR-RVIAPDHLGCGLSDKP-----QNYHYCLANHIANLEQLLVHLATGPVDLVVHDWGGAIGMGWAVRHPELVRRIVVLNTAAFLSPHIPLRIAAAKMPRFGEW-----------------------------------------------TVRRFNAFALAATVWAVERPLPPEVRAGYLWPYDTPAHRIAIARFVQDIPLHPAHPTwrELVCLRDKPMRIVWGGRDWCFDDRFLAGWLKRFPDAEVTRldAGHYVLEDASDDVIARIARWL----------------------------------------------------------    
 818 UniRef50_C1ABD8        Putative hydrolase n=1 Tax=...   129     3e-28  1  11.8%  79:433    22:322     ------------------------------------------------------------------------------RNPESRDLDDAARRAAPGKFARLTDGIT-HYELAGPDSGARVVLVHGFSVPAYIWDSTFVALSGAGFRVARYDVFGRGWSDRPDAP------YDRTMYNRQLGELLDTLGWrdVHLVGLSAGGPIVASYATQDPTRIRSFTLVDPAAGTAGDVP-------AVFQWPLVGGFLWQGIA-----------------------------------------VPTMADGQASDFNEPTRWPDWADKYRVQQQYRGFGRALLRTAIAQRGMSFDSIFTQAAAA-KRPSLLIWGIDDKTVPIARSEGVRQALPGIEYhiERAGHLPHIERADAVNPILIDFLRRHSAAPPDSAGGATRPQ----------------------------------------    
 819 UniRef50_B1ZCN1        Alpha/beta hydrolase fold n...   129     3e-28  1  11.7% 104:403    20:280     -------------------------------------------------------------------------------------------------------GLRIHHGVAGTGE-RTVLLVHGYPETAYAWRRVVPRLVAAGLRVVLPDYRGAGGSSKPPG------GYDKHSMAGDLHALldHLGlgPVTVVGHDIGMMVAYAFARRFPARTERLVVMEAP--------------------------LPGTAAYEESLRNTDRLWHFQFHRAPDLPELLTAGREQLYLERFYQDLAFDTEAIGL---------EAVARYVRAFSRPGAMRAGFEVYRAFPADAERNRalDRDGKLKVPVLALAGAHSAFAPMTEG-MMREVADDVTFRTIdaNHWVPEEN----------------------------------------------------------------------    
 820 UniRef50_A4YLX7        Arylesterase (Aryl-ester hy...   129     3e-28  1  11.3% 106:414     2:256     ---------------------------------------------------------------------------------------------------------TLHHEEWGS--GRPVLFVHAWSLTSAMWTYQISDLSRRGLRCIAFDRRGHGRSEVP------GDGYGLDRLADDIAEVIAQLDLNDVALVGMSMGCNEILNYIARHGTARIARIAMLGPTTPF-----------------------------------PLQTADNALGAPRAYFEQCWAAWQSDFPKWIEDNKLPFFTK--------DTSPQMMDWVAGMMRQM-----PIQVAVATNRALIATDLRPVLPKIDRPVLILHGDSDVSAPLdtGRPTAKGIKGAELKVYPGghGLFVTHMAQINADLAAF-----------------------------------------------------------    
 821 UniRef50_A1KLS7        Haloalkane dehalogenase n=4...   129     3e-28  1  13.2%  99:423    14:299     --------------------------------------------------------------------------------------------------YLEIAGKRMAYIDEGKGDA--IVFQHGNPTSSYLWRNIMPHLEGLG-RLVACDLIGMGASDKLS--PSGPDRYSYGEQRDFLFALWDALDldHVVLVLHDWGSALGFDWANQHRDRVQGIAFMEAIVTPMTW------------------------------ADWPPAVRGVF------------QGFRSPQGEPMALEHNIFVERVLPGAILRQLSDEEMNHYRRPFVNGgdRRPTLSWPRNLPIDGEPAEvrSWLEETDMPKLFINAEPGAIITGRI-RDYVRSWPNQTEITVPGVHFVQEDSPEEIGAAIAQFVRQLRSAAG--------------------------------------------------    
 822 UniRef50_B8KT15        Putative magnesium chelatas...   129     3e-28  1  12.7%  84:413     1:281     -----------------------------------------------------------------------------------MSPRPASASGTESgtFHRAAGHYWRVTEGGTGSD--ILLLHGTGASAHSWDYLSPILHEAGFRTVAPDLPGHGATDLLPARRM-----TLEAIATGVGELIEECGYEPKLVIGHSAGAAIALQMTMKGLIDPRGIISINGAFQPFGS----------------------------FAAPLLSGAARLLAHTPLVSFMLSMKGNRNETVSRVIEQTG------------SQLQPEALDAYRLLAGRPRHVTGALRMMAA----WDLRPLWYALPHLEVPLHLITGLEDKAVPPRQAHELADRVHGAKVHEinAGHLGHEEAPEIFAACVLE------------------------------------------------------------    
 823 UniRef50_A9DAM7        Esterase, putative n=1 Tax=...   129     4e-28  1  11.1%  51:420     8:316     --------------------------------------------------LIIIALFLFAFFLWTLIVSSGARRA-----------------VPKHGKTLTVAGNEIHYTDEGPRDAPVLVMIHGLAGNLNNLGYALTAQLSSEFRVINLDRPGSGYSTR-----SSDEAATLPAQARILGAFLDQLGVEKPVLVGHSLGGAVSLAMALERPEKIGALALLCPLTH-AEGKPHAVFEILMIRPKLLRLFLAYTLAAPVGKMTAKATLEQVFAPEAAPGDFMIKGGFALNSRPKTFLGASADLAATTASL------PALVARYKSDLKTPGA--------------------------------VLFGAEDPILNPdhGRQMEAYGLNYE-ELEGAGHMIPMTAPEACADFIRRVARQVER-----------------------------------------------------    
 824 UniRef50_C1BB33        2-hydroxy-6-oxo-7-methyloct...   128     4e-28  1  11.9%  85:437     5:317     ------------------------------------------------------------------------------------DENLVDVPGLASRWVRLANGARAHYMTAGT-SGPAVILLHglPGSsaGWRFMAPYLGENGFRVYCPDMPGFGLSDTREEYWPD----GMESFVDFIDQFATALCLDEFHLAGNSMGCMNTVNYTVAHPDKVKSFILI--AGDVGDVVPEHLTPPAGEFRLTAYDGTRDGMRTMMEAIIHRGEAISEDLID-----------MRYLSARDRMDAHAKFWPTLLQYRRITDWTDENRAARLV--------------------------TKGRLDRLSTPGLYLYGRQDILTPVEWGYVQEDHLPNVQFFYPdcGHQGQTDRPDLFNPVFLEFFRDGKVSGDLADAAGVSDRRPEL------------------------------------    
 825 UniRef50_UPI0001C479B5 UPI0001C479B5 related clust...   128     4e-28  1  11.6%  91:417    44:314     ------------------------------------------------------------------------------------------ASDHFISRRVDIGRITLNVREKGS--GPLMLFFHGITSNSAVFEPLMIRLSDR-FTTIAVDQRGHGLSDKPET------GYEANDYADDIAGLIRTLARGHAILVGHSLGARNSVTAAAKYPDLVRSVVAIDFTPYIETEALDALEARVNAGSQ---LFEDIKAVEAYLAGRYPNIPADAIRIRAESGYQP-------------------------------------VDGGLRPLASSAAMAQTARGLRS--------DLVPAYRDVTKPVLIVRGESSKLVSAAALAKTSRLRPDlvVVVPGADHYVNEVSPEITLKAITNFIDA--------------------------------------------------------    
 826 UniRef50_Q13PH5        Putative alpha/beta hydrola...   128     4e-28  1  10.6%  97:416     2:259     ------------------------------------------------------------------------------------------------EKYVSADGLELRYTIQGA--GPYVTLVHGIGADHSSWDVIAQAL-EPFFTVVRPDLRGHGQSSR-------MTRCSLDDFVSDIDRLLDAVKIDKTHLVGFSLGGLISQKYAIEHAQRLNRLVLISS------------------------------------VAERTDQERERVLKRADQIDRDGIASVVSAAEDRWFTPAFKAANPDRVAFRLQQLIANDHRSYAAAYRVFAEADRGMAFER-----------------IQAPTLVTTGENDSGSSPRMSRLLHEKIRNSKliLPELRHSVLMEAPDTIADMLLEFLR---------------------------------------------------------    
 827 UniRef50_D2B6D0        Hydrolase, alpha/beta fold ...   128     4e-28  1  10.5% 106:418     3:258     ---------------------------------------------------------------------------------------------------------DLFWLDTGT--GRPLVLLHGGFLDHGMWEDQIPFLASR-YRVITPDARGHGRSANATEPFRPT---------DDLAALLRHLGTGPAILVGISMGASVAVDTALEHPELVSAVVVSGAGTSEPYFTDPWTIQTMTAWHSAMAVGDLDASVEA----------------------------------------------FTLFAAGPYRTLDDLDPGVVAHLRRMTRDTMSKHTAGEPNLLLQVRDTWERVAKIDVPLLAVNGSADSPDHIGMAERLARTVANGRAIPVDgaHYPNMERPDVFNEILEGFLRTL-------------------------------------------------------    
 828 UniRef50_Q1YGF6        Alpha/beta hydrolase family...   128     4e-28  1  10.5% 112:416    18:270     ---------------------------------------------------------------------------------------------------------------CGVNAGVPVLLLHGAVQTRAVWAGQVEALVDR-HRVIVPDLRGHGAT------PLGGERLTIDRMAMDCLALLDRLGIGRFAVCGVSLGGMVALEIAARAPDRVTALVLANTPRSLSGIGW----------------------------------VRRLVDWLDPQRLLHPTFHILGQA-----RTARIGLALAARLVGPFWVGRDARRHFIAGF-RAMSPDAIVATYRAIVESRPLD-----LGRIHCPCLVIDTLHDATTIREQAADIVAEAPNAMSVVLEAGHVANLDNPAVFNRVLAEFLA---------------------------------------------------------    
 829 UniRef50_D2PUS0        3-oxoadipate enol-lactonase...   128     4e-28  1  10.6% 106:415     2:251     ---------------------------------------------------------------------------------------------------------RLNYQATGPEDAPVVLLASSLGTNHAMWAPQLDALSD-HFRVVAFDHRGHGASEAPPGP------YTIDDLGGDVLELLDELGAEQASYVGISLGGAVGLWLAQNAPDRFHRFALLCPPVNPAANAQTW--------------------------------IDRAAQVRAEGTQSITEATLGRWFLPEYAEAHPDEVDLIRQQLLATP---------------AEGYAACCEALSALD-------LTPDLGSITAPVLLVTAESDASVPPETVVPLATQIPGAHLEipGAAHLVTWSHPDTVNPLLLTHL----------------------------------------------------------    
 830 UniRef50_C1A7S3        Putative hydrolase n=1 Tax=...   128     4e-28  1   9.9%  93:416    10:286     --------------------------------------------------------------------------------------------PWPAKRHQTVDGA-LHYIDVGA--GPAVCFVHGTPTSSYEWRHQIRAL-QDTHRCIALDHLGFGQSERPAHAS-----YTPEAHAVRFRAFMdhAPTEPFTLVVHDFGGPIALDWALDHPSCLARLVVVNTWMWSFEHDVAMWKQ---------------------------ARLANSWLVRQLYRHLNASLRILMPGAYGHRARLDPAVHGYYQSLFPDADSRERVLFALARALTQ-------------SSAFFDRLWQRRMALDQVPMTILWGLADRAFPPPVLAQWRQAFPHADVTTFDggHWPHEESAPAFTAALVSALQ---------------------------------------------------------    
 831 UniRef50_D2B450        Hydrolase or acyltransferas...   128     4e-28  1  13.0% 107:414    64:316     ----------------------------------------------------------------------------------------------------------LHVVHDGPRQAPPLLLIHGSGASGGFWSPMVPALVG-HHHVIRVDLPGCGQS-------PPAPPYDVPAQAGRVAALLDDLGLRHVAVGGHSSGGYVATALAEQRPDLVGSLALISSGPSM---------DALLRQPFILRVLLAPPLGPLLWSRRSDAMIRRGI---------------------------------SATAARPVDVPDDVVAGLRDT-----TYRVMRTVLRRNTAYIAERSVPERLAALKVPVLVIFGAADPRWEPSSAHRY-DAVPNARVELLPggHLPMLEAPETTSKLLLGF-----------------------------------------------------------    
 832 UniRef50_A7HC18        Alpha/beta hydrolase fold n...   128     4e-28  1  10.0%  67:425     1:321     ------------------------------------------------------------------MVLRTGRRRAPHARPPPAEPLSAARRTltEHRATALDGTRLHWTSAGAG-GPAVVLCDGIGCAGYVWRALEPLLA-RERRVLHWNYRAHGRSAAPSDP----ERMTVDDCVSDLLAVLEAAGEERAVLAGHSMGVQVALELQRRHPDRVAGLLLLCGAPGHLLDTFHDSSVLRYAFPWAKQLVLRYPDLARLAFRTVVPTDFALSYAMTF-------------------------------EVDGARVRRADLERYLEDLS-----EVDPALFVRLLASAEDHDTRPHLPHVTAPTLVVAGERDSFTPLRLSEAIHHAIPGSELLVVPGghVAPLEDPELVGERTLAFLGVHAPVRRPR------------------------------------------------    
 833 UniRef50_A0QW20        Alpha/beta hydrolase fold n...   128     4e-28  1  14.5% 104:420    19:303     -------------------------------------------------------------------------------------------------------GFDIFYRVDgpGTGPNLLLIHGYPFNSWDWSLIWPTLTQR-FTVIAPDMIGMGFSDKPVA-----YEYRVTDHADIHEALLAHLGITSTHILAHDLGDSVGQEMLARHEFGGQA---------------------YGGFQIESVTWLNGGLFNEAYRPRLMQKVMSRT----------PLGDILSPLQGSPLSRRVFESTLDQMFGVNTKPSREMIEKFHQILDHNDGKRVLHKVGRFVTDRYTHrrWVRAMRETSVPMRLIDGPSDPNSGRHMAQRYLEVIPDPDVVMldIAHWPQIEAPDAVLAHFLEHIDRVVA-----------------------------------------------------    
 834 UniRef50_C4RCX7        Alpha/beta hydrolase n=1 Ta...   128     4e-28  1  10.1%  90:415     7:277     -----------------------------------------------------------------------------------------AVRTFPVVTKIEINGAKLAYDDAG--DGPPVLLLHAGIADRRMWREQVPALTAR-HRVIAVDLRGYGDSGLPTDPFAHR---------DDVVGLLDALELPRAALVGCSFGGAVAIDTALAYPDRVSALALFGTAVSGNEWS--------------------------EEANDLWDDLVGETDPEDLAASAAGEVRFWVVGPRRRPEDVDPALVRFAEEMDRRALAAELALSAV-------------------DVAELDPPAIERLGELRVPVLVTAGAADVPDIGRIADRIAAEVPGAVRlvPDAGHLLPLERPEAVNSALLAFL----------------------------------------------------------    
 835 UniRef50_UPI0001B56501 alpha/beta hydrolase fold p...   128     4e-28  1  12.3%  93:416    10:298     --------------------------------------------------------------------------------------------PAREHRFVTSDGCALHVELSGPADAatLVLVHGWTQDHRTWDGVMEHLGP-GVRTLRYDLRGHGGSAPATKETG-----TIPRLADDLAELIAavPSGPLVLAGHSMGGMTMMALAERHPELVRDRVVGAAFVATSSGQMDRITLGL-------------PGMAGQFSARFERRLAKALSHRKGERLPLSPAMVRSGA-------------RFLVFGDHPRRADLRLVADQLLCAHPASLGAFQDAISTHDRRVALATLRG------TPSVVLAGEKDRLCPLPHAKVIADELPDAEFvfPGAGHMLPQERAGEVATRIGALLR---------------------------------------------------------    
 836 UniRef50_B9L396        Alpha/beta hydrolase fold p...   128     4e-28  1   8.9%  94:419     2:276     ---------------------------------------------------------------------------------------------WDEERTIAVRGVT--YRVCAAGQGPPLALLHGFAGSSAHWAPLAPELVARGWRVIAPDLLGHGRTDAPSAPT----RYAAAEQVADLASLLGELVSGPVRLLGYSMGGRLALHLALAHPERIAALVLESASP-GLDDPAERAARQRADEELATAIEQRGMDWFADYWERLPLFASRQRLSAERRAALRAEWLAQRPH--------------------------------------------GLGASLRGFGTGSMPAVWDRLSELRMPVLVIAGALDTRY-AALSQAMAARIPASQLVIVpaGHTVHLEQPAAFLAALERFFRQLP------------------------------------------------------    
 837 UniRef50_B0T6Q0        Alpha/beta hydrolase fold n...   128     4e-28  1  12.4%  95:416     8:284     ----------------------------------------------------------------------------------------------FRHQTMDGGNARVQVAVGGV--GPPLVFIHGFPQTWWEWRRMLPGLAKTN-TVVAVDLRGAGHSDCP------QEGYDKATMAADVHGVMLALGFERYAVCGHDVGARVALALAVAYRGAVTRLAVLDAS---------------------------QPGSGAWEANCAKPGLWRLAFHPKRNLPERPPYGRKYDYVSTFTFIADHAF-------GPDAHTVEDLPVFAGDLARPGNTRGGLEWTRAFPNDHRNAQTRKR-ERLAIPVLALGGDHngPTILALLEAFAETVSGGG--IADCAHWLPEERPAETADALVEFLK---------------------------------------------------------    
 838 UniRef50_Q1GLC8        Alpha/beta hydrolase fold n...   128     5e-28  1  14.1%  97:407     2:271     ------------------------------------------------------------------------------------------------SRFETSDGLSLYFEEHGSADGIPLICLPGLTRDGQDFRYALPHLGD--YRVILMDLRGRGRSDYAQDPAS----YNILTEAGDVIALMDHLGLTRAALLGTSRGGLVAMAIAGTARDRLQAVILNDVGPDIAEEGLGRIFEYLGHRPAAKTHAAAARILEAAM---------------------------------RDSFANVPEGRWLEEAQTFYKETPDGLELRYDARLR----EAMLAQAKALSEMEEKPSLWPWFAALdlPCGVIRGAHSDILSRETFDEMRRRLPALQAVEldRGHIPFLDEPDAL------------------------------------------------------------------    
 839 UniRef50_Q0SAG0        Possible epoxide hydratase ...   128     5e-28  1  12.1%  91:436     3:312     ------------------------------------------------------------------------------------------PSTSRTARRITNGSVELAVFEEGNPDGETIVLVHGWPDTHDLWNRVVPFLTE-WFRVVSYDSRGAGRSTVPTRV----EDYRLPALAGDLFAVIDAVSPGrvHVLAHDWGSVEGWEAVCEPGARERIASFTSVSGPNLDHLGKWMRRRISD-------RSFGGPLAQAVA-------------------SGYTVLFQIPGLVTLPLRLWFSRNWPAFLKFFDRLDPALVRP------APTLADDMVNGLKLYRANIRTSLLRPRERYTDVPVQLILNKNDKAVRPVGYEDTEKWAHDLRRREVEAGHWSPISHAEDIAQLAAEFVLDVEDRRWADLPDGPSESASA-------------------------------------    
 840 UniRef50_C3KE34        Putative family S33 unassig...   128     5e-28  1  10.5%  98:420    60:337     -------------------------------------------------------------------------------------------------RFVEVDGERFHYYEEG--KGRPLVMIHGLMGSSRNLTYALSGQLREHFRVITLDRPGSGYSTRHTGTAA-----DLPAQARQVAAFIHTLGLDKPLVLGHSLGGAISLALALDHPHTVSGLVLVAP-------------------------------------LTHPQPTLPLVFWSLAVRPAWLRRWVSYTLTVPMGLLTRRAVVKGVFAPDSAPEDFATRGGGLLGMRPENFYAASSEI---ALVNDELPDMVKRYPQLTLPISLIYGARDKVLDFrhGQALADKVPGLKLQVVEggHMLPITATERVVEAVHQLAKRARP-----------------------------------------------------    
 841 UniRef50_C9YAI0        Putative uncharacterized pr...   128     5e-28  1  13.5% 102:420    31:292     -----------------------------------------------------------------------------------------------------IAGFDIPYLEGG--QGEVLVLVHGFGGDKDNFTRMARFLTP-HYRVIQPDLPGFGD-----ATRDPAARYRMADQVERLHAFFQALGVQKMIFGGNSMGGFIACEYAARYPDQVKALWLLDAAGTATAHDSPMLQHYLATGESPLLLRSQ----------ADVARLIRATMARPPYFPGFLKRTLGARAIADYPLHSEIIKD----------------------------------------LSQHSPMLETRYTTLPTPALIVWGAEDGILNPAASQVQEQLFPRHQTIIMQggHLPMLEAPAQTAQDFMKFAKTLPA-----------------------------------------------------    
 842 UniRef50_Q3JY25        3-oxoadipate enol-lactone h...   128     5e-28  1   8.9%  98:422   101:372     -------------------------------------------------------------------------------------------------PFVTIDGQPLHYQIRGA--GAPVLFGHSYLWDSSMWEPQLDALS-KSYRVIAPDLWGHGRSGPLPDGTR-----SLDDLARQMSELLDHLDIDTCSIVGLSVGGMWAVPLAHRAPQRIDRLVLMDT--------------YVGVEPDATRNQYFQMLEAIDAQGAIPAPLLDAIVPIFFRPGIDPASELPTGFRRALQAFTTERLRDSVIPLGKITFGREDARAQLSALPADR-------------------------------TLVMCGANDVARPPEEADEIAALIGCEKAFVPNAGHISNLENPAFVTQALSDWLGRGAARA---------------------------------------------------    
 843 UniRef50_A9F250        Hydrolase alpha/beta fold f...   128     5e-28  1   9.8%  94:418     1:289     ---------------------------------------------------------------------------------------------METRRVTSFDGTDITYRVTPPpgAKTVVLVNGLGGPYLAWRAQVEYLSDQ-YRFITWDYRGMYASKRPS--PDVPGAYAVANHVRDLEAILAAEGIRHGALMGWSVGVQVALEAFRRLPGFAANLVLLNGTYRRPLETIGPLPGLGRLFPPFVAF------------AARAHTLAARVTRSTIGPPDAVLWLKRMGLLGKT-------------------LDSEAFAELVQPFGRLDMDALFSNL-----RALDAHDAESVLEQIDVPTLLITGDRDAFTPRALAQSMARRIPMAEILVVRGghTTAVEFPELVSLRIERFFAEH-------------------------------------------------------    
 844 UniRef50_A7YVV4        Putative uncharacterized pr...   128     5e-28  1  12.0%  91:415     3:272     ------------------------------------------------------------------------------------------APPSPSRETVDVNGITLSYLTVGSPGASPLLLLHG-TFWSRVWQPVLPALAGQ-LHCMALDFPGFGHSE----GELGVTEASIPALAQIVLDTADALGLDRFAVAGHDIGGGVAQHLAA-HSGRVTRMVLINA-----------------------------------------------VMFDSWPVPAVGKFRDPAVRAAINPADLLAARTKTMQLAVDRQLATEEIDDYVQPWRSPSRTRSWMAMAAAADSRYTRQ-LLPALTAAAVPTRLIWGRDDDFQKVSFAQHFVTVVPSSDLIEVAGKHIPMEDSPQNIAAAMLEHL----------------------------------------------------------    
 845 UniRef50_Q0VT81        Hydrolase, alpha/beta fold ...   128     5e-28  1  10.6%  82:421     7:305     ---------------------------------------------------------------------------------PTTRNQSASSAPPETGRDIqnGGLALSATRFGDPKSPQVLLLHGGGQTRHAWTHTATVLAKAGYCATIIDARGHGQSQWCPKG-----DYSATALVSDLRAIIQSLPSSPYIVGASMGGLTAMLALGEEASLNCRGLVLVDVAPKLERNGVRRII---------------------EFMRRHRDGFDSLEQVRDAVAAYNPGRKAP---------ANSQGLRKNLREDVNGRLHWHWDPAFLDHAEIPETDESMFGGAR----------LNRAARHLSMPILLIRGYHSDVLSETGAQELLALVPQAQYRVIeaGHMVAGDRNSIFTGAVMDFLQSIDPP----------------------------------------------------    
 846 UniRef50_UPI000050F83A putative epoxide hydrolase ...   128     5e-28  1  10.5%  79:421    20:319     ------------------------------------------------------------------------------SVAARLAELPVTAEARELRVRTQRGDFR-GFRATPARPDPAllLLHGWPEFASCWESTAALLLEQGMSIVAYDQRGY----SPDVRPESVDEYTMAHLVADLDVISREVGLERFHLVGHDWGGMVAWHYAANHPERLLTSTVVSTAHPIAHAKQVRADPD-----------QYERMAYMRSIQDHPEGVARTLLRNDGQR---------------------------LIELYGGDVPDGMAASYVERLSEPDAMLSVLKYYQAADGRERTASTP-----ITVPTTFVWGSEDVAFTRGSAELSASYVEgyRFvlVGASHWLPESRPEDIARAVLERVREHAAS----------------------------------------------------    
 847 UniRef50_C5D1J4        Alpha/beta hydrolase fold p...   128     5e-28  1  11.3%  94:419     1:282     ---------------------------------------------------------------------------------------------MKTERLRLSGGTELSFTTAGEASRPALLLLHGTPNSARLFRAVVPALSQAAF-VIAPDLPGHGESEPLPAP-------SFAAIGQAIAELLDSLEIGPRFIYLHDWGAPVGLQIAMQAPGKVLGLIVQNANAHRTGWGPGWAATQAYWSQPNAENQAAATAHLTLAGTRD-------------------------------------TYIAGVPPEVAERIPAEHWEEDWRVMNLPGHMDTQRALIADYGnaARFGAIAQYLERWQPPALMAWGRHDIYFDLAEVLSWMRALPRMEAHVLDAGHLLLETHAAEAVALMLDFIRRTP------------------------------------------------------    
 848 UniRef50_A3UC51        Putative uncharacterized pr...   128     6e-28  1  11.6%  93:419     3:288     --------------------------------------------------------------------------------------------PFSEHFYTAPDGIRTYYRRygDSGKTPVLCLHGLTRNSRDFEDLAPIVAESGRSVIALDVRGRGRSD----YDSQIENYNPAKYVEDVIGVLDELGWSQVISIGTSMGGLMTMILSTLRPKLLTGVVMNDIGPELDPAGLKRIQGYVGGAGKFANWDEAG---------------EALRAINGVAFPRETGADFWIAFAKRTCRALD---------------TGEIVLDYDANISKPVK-----------DGDVAPPDLWPFFEGLeiPLLLVRGAISDLLAMDCVEEMSRRHGDMELavPDVGHAPLLTEPEAL-EPIKAFLNRLD------------------------------------------------------    
 849 UniRef50_UPI0000DB6D6D PREDICTED: similar to epoxi...   127     6e-28  1  13.6%  76:376     2:255     ---------------------------------------------------------------------------QQRDKPPPCLIDNNLG----IHSYIKIKNVKFHYVEAGNKNESLILLLHGFPDCWLSWRKQIPCLA-KYYRVIAIDLKGFGDSDKPAAKSC----YKIQVLIEELKQIILTFGVKQCSIIGHDLGGLLGWYIVALYGDMIDKFVAVSCPHPN-----------------------------FYWNRRLGDSIFDLKWIHFSRLPFFPEIDALKEDLSIINDAFQHL------QLNNTNTEKDYVEAYKYAFSRKEDWTGAINYYRNLPFIKLNTDSCD---QISTQTLLIIGNIDPIVTIE-------------------------------------------------------------------------------------------------    
 850 UniRef50_A4XXL7        Alpha/beta hydrolase fold n...   127     6e-28  1  12.0%  59:417     6:327     ----------------------------------------------------------VILISGARHACNSPATRPVRCRPVNAPLHRRELPAQRCH--VQGDGVRLQAYVWGKADGPTLLLVHGYPDSHEIWLPLVSELAAD-YRILAYDVRGHGASQAPRRL----RDYRLERLANDLEAVIRALSpqPVHLVAHDWGSIQSWEAVTEPRIQPLLASYTSISGPCLDHVGHWMRQRISLRSPGALRQVLGQLLSSWYIA-----------LFHIPLLPELCWRLGLDRAWPWLLRRVEGVHGLPPSSTQRSDGMRGVQLYRANFVRS--------------------LFSPRARSTRVPVQLIVPLADRFVRPQLFDDLSHWAPTLTRRDVRGGHWRVLAEPKALAGWIREHVTR--------------------------------------------------------    
 851 UniRef50_UPI0001AF557A haloalkane dehalogenase n=2...   127     7e-28  1  13.2%  91:423    10:296     ------------------------------------------------------------------------------------------AGYRASGREFTAAGVRSFALDAGAPDAEPVVCVHGVPASSYLYRKVVAAVAHRGLRGIAVDLPGLGYAERPA-----DFDYTWTGLGRWLLAAVDALELERFHLVVHDIGGPIGFEVAAAQPDRIRSLTMLNTTVAVESFQRPWMMEPFA---------HTGVGEAWLASLQLPGVFLSL----------------------------------MRYTGVSRKVPAAEIAAYVPLLLGDDGGRAFLKIMRGFEPTAdqRRYVSAVRNTPYPVQIVWGARDHALTLRhhAQLASGIDDP--ILLPAKHFLQEDYPEQIADAVCRLASAVDGQRR--------------------------------------------------    
 852 UniRef50_B2UJ57        Alpha/beta hydrolase fold n...   127     7e-28  1  10.3%  77:445     2:340     ----------------------------------------------------------------------------SAPAASAHSRHGPPVARQQSSHVVHSGDVRLFAreAAARQRPAVILVHGYPDDSSVWNGVRDALA-RHCRVLTFDVRGAGLSDAPTDTA----AYRIAQFVDDLAAVADLlpGERFHLVGHDWGSIHSWESVTTDRLRGRIASYTSISGPCLDHVGHWMRSQLDVGTW-------------AAKRAVWKQRLQSWYVGYFHLPWVPEWSWRLWAARAWPWWLRQT------------------EGVHGAGRRPSLTRDACNGLWMYRANFRERLAHPQVRAPHAPVQLIVPTHDRYVSPAVTRAVEAWVSHYWRHEIDAGHWLPMRRPEWVADCIRRFADYVDSGHEPAALQQARVPGVSAALEDPVQR----------------------------    
 853 UniRef50_Q4QE86        Hydrolase-like protein (Est...   127     7e-28  1  11.0%  67:412    23:374     ------------------------------------------------------------------TATKQPTIPEDMDLPPAIFEELRrfPHPPDDAFVSVgdITLCYSAFGDPSDPCILLIMGMNVTSLLWdrFCGYLAAAGFYVIRYDNRDVGLSTHFDEYPSpsLAYTLEDMAEDAAGLLKALKISQAHIVGCSMGGMIAQLLTLRHPDMVASLCLHSTSAGPVWPRASLLMNILDQPESTRDVHSVLDY-----------RVRFFKAVAGDMTFHEHEFRLGMWFDFARAPYQGGGRRHLAAIVRSKDRSAALKAHINRLTSTTAS---------GSERGSSSLPASGTAPSRCIPVVVLHGGKDPLVVLSNAQHLASCINGARLVVFpmGHYFSKEMYRAIADEII-------------------------------------------------------------    
 854 UniRef50_Q4K713        Hydrolase, alpha/beta fold ...   127     8e-28  1   8.4%  97:419    86:379     ------------------------------------------------------------------------------------------------TQRVDLPQVSLAYQSIGRTSDPALLLVMGLGGQLIHwdEVVVALCQQGFRVIRYDNRDVGLSTWRQAPvpVAAPYTLTDMADDALGLMDALHIEQFHVLGASMGGMIAQHLAAMEPRRVESLTLIMTSS---------------------------GAEGLPAPNAALVQLLSRRNAPSREAALEQQADLLAALGSPKVSDDRQVLLQQAAVAYDRAFNPEGVK-------------------RQIMAILAEPSRVALLKQLRVPTLVVHGTADPLLPVMHGVHLAAQIQGSQLKLIPgaHRFQEAFKAPLLAAVLPYLQTHR------------------------------------------------------    
 855 UniRef50_A8LZ67        Alpha/beta hydrolase fold n...   127     8e-28  1  11.4%  94:425     2:303     ---------------------------------------------------------------------------------------------PDVHELHLTDTVRLHVEIDGPTDAetAVLLHGWTLDANTWHRQLAYLRDhaLRVVTYDARGHGRSSCMALPTA-----TLGQLGDDLAAVLDAVApgRVVLAGHSMGGMTIMEYAHRHPTHFADRIAGLVFVSTSAEGNARTVYGLSP------------------------RVARLIQIAESTGAGVLARCGTRPPPRSLLRALRPSFRWLL---FGDRYNPADLRLVTRAMA-----HASLRSIGGFRASIGTHDQLATLPGlhLPTATLVGDRDRLTPPPCTESIAAALPTTALTvpGAGHMLMMERPGAVNAALAGVLGRVLDSPAAQ------------------------------------------------    
 856 UniRef50_Q13Y29        Alpha/beta hydrolase fold n...   127     8e-28  1  12.0%  97:429    43:324     ------------------------------------------------------------------------------------------------SRFVKIMGADVHYVDEGSGD--IIVMIHGFASSLHTWNRVADEL-KREHRVIRLDLPPFGVTG----------------------------------------------------PLRSSSGAIETMDLPTYRRFIDTFMQALGISRATLIGNSLGGLISWDYAARHRDAVERLVLIDSAGFPMKLPIYIGLFNSALVRVSSPWWLPEAIvyGDPRKIDAVTLRRYVEFFHGEGTRAAIGKMVPTLDFRQVDTNV---LKTFDVPALVLWGAKDRWIPTAHAAEFASRIPGAKSVMYAggHIPMEEAPERVMVDLRAFL-GIRPAHSAAAEQA--------------------------------------------    
 857 UniRef50_Q3B5G0        Dihydrolipoamide acetyltran...   127     9e-28  1  10.1% 104:421    19:292     -------------------------------------------------------------------------------------------------------GAKIRYREYGPSdgTPILFLHGYGAMLEHWNLNIPEFSG-HRRIYAMDLIGFGRSEKP------NVRYSLELFAAQIEAFLQLKKLQRIVIVGHSMGAASAIWYADHRPEKVEALILANPSGLFGDTMDGMNSVFFGLVGSPLIGEVLFTAFA------NPVGVSQSLAPTYYNQAKVNL--------------------KLITQFTRPLQDRGAAWSYLSPSRRPGDFR---------------LDDLEKPSHYKGKAFLIWGAEDTALPPhiIPEFQQLLPQaaWIIPKAAHCIHHDADEAFNERLQAILDSIESA----------------------------------------------------    
 858 UniRef50_Q3J186        Magnesium-chelatase, BchO n...   127     9e-28  1  11.5%  84:421     3:288     -----------------------------------------------------------------------------------LDRMPPDWPGRESMRRIDCPPHQWCVRDEGR--GPTALLLHGAGGSSHSWRHLVPLLAP-HFRVVAPDLPGQGFTRA------GQRRFSLEAMAEDLARLCAAEDWRPSVLIGHSAGAALALRLAEILPVRPRAVVGINAALGNFEGTAGWLFPLIAK----LLALNPFIPPMAARLFGAEDRVRRLI------------------------------------GSTGSDLDEAGLRQYMTLIGDHVHVDGTLSMMAQ----WRLDALQRRLPAIDVPTLLIAAAKDGTVPPRISQEAAGRMpgSYVEMPGLGHLAHEEDAAGVAAILLPFVERHLRT----------------------------------------------------    
 859 UniRef50_D2QEL9        Alpha/beta hydrolase fold p...   127     9e-28  1   8.9% 107:419    11:288     ----------------------------------------------------------------------------------------------------------IHADAWGDPQNPPVLLLHGGGQTRHSWGDTATVLADAGWYAVALDARGHGDSDWSDKA-----HYSIDYLADDLRKICTTFDQKPA-LVGASMGGMTAlrGPSHPEPICSAIVLVDIAPRSEQKGIERIFAFMSQNLDGFATLDEAAEAVAAYLPNRPRPSDNSRLEKNLRLRPGPDGDQRY-----------------------YWHWDPTMLAIWRHSTGPDKQTG------------NADRLYQAARSLTVPTLIVRGGISDVVSEKVMTEFLDAVPHVQSivADAGHMVAGDSNHAFTRAVIDFLSGVS------------------------------------------------------    
 860 UniRef50_A5DUP2        Putative uncharacterized pr...   127     9e-28  1   9.3%  40:417     2:342     ---------------------------------------SSTIHILRQWARCFSTKRLLFYSNCLRHRKQFTPNFDISKIPdpTQKHVKLSFKQLRPHRTEID-----------PNATPVLFLHGLFGSKLSFNKAGRHVSELSKrvFAVDLRNHGDS-------PHALPHTYIQMAHDVSQFIEERNWEECVLVGHSMGAKVSMLVSLLKPNVISKLIVVDNTPHSMVLGDEYANGLLGMC-----------EVETRASEFRKENDKDQMVVNFKEVRDFLSNYEANDLMRSLLVSNLVHKSKDFEVLKATGQLREMFKVPVMNFWKHNVIEAMGSW----------PDDFQFVKKYDKPVLVMYGNRSEFVKPEYFPLFENYFDQLQFEEFDSGHWILQEQPTKFVKSVLDFISE--------------------------------------------------------    
 861 UniRef50_D1A1X8        Alpha/beta hydrolase fold p...   127     1e-27  1  12.3%  91:431     6:314     ------------------------------------------------------------------------------------------GEQVARSRVRSRSgvELAVYRQGDPGRPVVVLVHGYPDTHAVWDGVAARLAER-FHIVRYDVRGAGASSHPAGTAW----YALEHLVADLRAVleAAPGGPVHLVGHDWGSIQAWEAVCTMPERFASFTSISGPCLDHVGHWLR---------------SRLARPSPRNLRMAAEQAVRSWYISWFQVPVLPEALWRAGLAQPLRRILRRGAGPLRHGNRPSGTSAADAVAGLGLYR----------------ANVRARLRDPRERRTDVPTQVIVPMRDVFVSPYLVGGLRAWAPNLTLRRIEAAHWVPRTHPSVIARWVSEHIARVEDGAETPRRGQAP------------------------------------------    
 862 UniRef50_Q93K00        Haloalkane dehalogenase n=8...   127     1e-27  1  12.7%  77:414     3:296     ----------------------------------------------------------------------------VLRTPDSRFENLEDYPFVAHYLDVTARdlRMHYLDEGPIDGPPIVLLHGEPTWSYLYRTMITPLTDAGNRVLAPDLIGFGRSDKP----SRIEDYSYQRHVDWVVSWFEHLNLSDVTLFVQDWGSLIGLRIAAEQPDRVGRLVVANGFLPTAQRRTPPAFYAWR----AFARYSPVLPAGRIVSVGTVRRVSSKVRAGYDAPFPDKTYQAGARAFPQLVPTSP-----------------------------------------ADPAIPANRKAWEALGRWEKPFLAIFGARDPILGHADSP-LIKHIPGAahARINASHFIQEDRGPELAERILSW-----------------------------------------------------------    
 863 UniRef50_B1VHQ7        Putative hydrolase n=1 Tax=...   127     1e-27  1  11.5%  55:428    14:363     ------------------------------------------------------SVSSLATEVRSRVEEKKARLFPKSSTPRQVRPGIRDLDATGVLTVADHgdIPLHWYEVGPGHglTIVFIHGFTLGADSWYRQFRGLRKkgVRLLTLDLRGHGKT-----GAVPPSLCSTETAAEDVLAVleRAPAGKLILVGHSLGGQIAFAALRHAPEDVRQRIA-------GFVLVSIAIDRFAANGIPELLNLRSVGWLAGLLKASPKRVRKLRDKIAGLIAP-----------------------ALAIAVFSRPTNRQVIDLHAYMIN-----ETPLDTYLGFLDDLRGHDEYDAVPYLslPGTVLVGDNDDVTSREQAEKIVAAWPGAELIEvnAGHMLPLEAPKEVNAAIARLAERVAGGTTHPQQR---------------------------------------------    
 864 UniRef50_B0T6K8        Alpha/beta hydrolase fold n...   127     1e-27  1  11.6% 102:419    41:320     -----------------------------------------------------------------------------------------------------AGGVRFAGVIGGS--GPPLLLLHGYPETHIAWRKIAGDLS-RHYTVIAPDLPGYGAS----RPAAMSPRWTKRRVGEALVALMRTLGYERFTVIGHDRGARSGYRLVLDHAGVVKAFVSLTVA--PTLDVLEGVDYRFAQKNYHWFLFAQPPPLPETLLAAGPDAVINWEF-------------------------------DVMRAADDAVIEPAARDAYRSAFRDPAVQHAMCEDYRAALDEDLVLDRADrgRKLDCPVLVLWPQVEAVGKPPPLEAWKRWADNVVGATTTGDHLQAEDRPAEVLAALIPFLARHA------------------------------------------------------    
 865 UniRef50_A6RBD6        Predicted protein n=4 Tax=O...   127     1e-27  1   9.3% 118:417    37:353     ---------------------------------------------------------------------------------------------------------------------PPLLLLHGFPDGPLLWRPLLPYLLSLPHRLIIPSLLGY----HPTSTPSDPTAYNSKSMSDDLAELLTAEDARTVIAIGHDWGSFLANRLYLWHPERVAGLALLNLAYMPPNQTTPFDLDQTNALMEQLFGNPILAYWElrATGDVPALLEgeLLHAARPQPEWMRKLLCERGATEAFLTAGTVDGEAVAGDIKLRDYAGHGGVYWEDfrEAGGIAPALCWYRAMSENATFEVERTLEKelKVPTLFVACPGDAVCRAELIEAPKqlLPDLTVVEvgCGHwiIYEKAEETAGVIGGFLKE--------------------------------------------------------    
 866 UniRef50_A1CLN8        Alpha/beta fold family hydr...   127     1e-27  1  14.1% 109:421    50:334     ------------------------------------------------------------------------------------------------------------YYEAGPSSGPAVILVHGFPYSIDAFVSVVPLLVNQGYRVIVPYLRGYGGTRF--VHPDTLRSAEQAALGYDIIALMDAIHLDTAICAGYDWGTVAVNVATALWPERCTRMVAANSYLIQNRSTAWQPSDPNSEATKWYYYLFLTPRGAAGLAEAPKEWTQTLWEKNSVG----------------------------------WNFTEEYLDLTVTSLLNPDFVDIAVNFYRNrpAFVDLADKLDHQPPITVPSVTLDPENSVVLTPNNGSATAKFfpRCHYQIPDVGENIPYQAPKTFANAIME-VSRLSAS----------------------------------------------------    
 867 UniRef50_C1XGY5        3-oxoadipate enol-lactonase...   127     1e-27  1  11.9%  99:418     6:262     --------------------------------------------------------------------------------------------------FAALHHLTLHYRLEGEGT-PTLVFLNSLGSDLRIWDGVVASLEGR-YRVLRYDLRGHGLSDAPVGP------YTLEDHSADLLALLAHLGVPRAVLVGISVGGLIALDLARRQPERVEALVLCDTGARIGSQD------------------SWNERIAAIQHKGLPEVARTV--------------------IARWFTEDFFVQRKAEAAGYYNMLSRTPVEGYL-----------------GTCAALRDGDLRPWLRSIRAPALVLCGELDKATPPELSQELAQGL-GARLeiPQTAHLPCVEAPALVGQHIRSFLKEL-------------------------------------------------------    
 868 UniRef50_D2RY22        Alpha/beta hydrolase fold p...   127     1e-27  1  10.7%  91:416     7:283     ------------------------------------------------------------------------------------------WGDEQATTTVTVDGHDlaYHEDGPSAgpPVVFLHGIPTWSFLWRDIVPAVAKER-RTIAPDLVGYGNSAMQDG-----FDRSIRAQEVMLEGLLEDLDIDRVVLVAHDIGGGVALRFAAHNPDAVEQLVLSNAVCYDSWPVEFV-----------------------------------SELGLPSTADREREELEARLESAFVDGAYGEADPEFVEGMKTPWLTDE----------------GHLSLVRDAVATNTNHTTEIDYGAIEAETLLLWGEDDVMQPYDYAERLAEDIDDAALavSDAYHWVPEDRSDAYGDRLLDFLK---------------------------------------------------------    
 869 UniRef50_A3VK87        Alpha/beta hydrolase fold p...   127     1e-27  1  12.7% 103:418    19:289     ------------------------------------------------------------------------------------------------------DGVTLVGEAFGAADAPPVILAHGGGQTRYAWSRVAQRLGDAGWQAVALDLRGHGESDWHEGG-----DYSIPRFGHDLDHVARQFGQKPVIVGASLGGNAGLLVAGQGADAPCRGLVLVDITPKIDERGVDKIIGFMGRHMDEGFASYDEAAEAIA------------GYTNRKGRKSNRDSLRRYLREGEDGRLRWHWDPAFIVGRKSSRMAPESL-----------------------------GQLEGALAAVTVPILLVRGSNSDLVNDESVAEFLKIAPQAQFrvTGAGHMIVGDRNDVFAETLDDFLSGL-------------------------------------------------------    
 870 UniRef50_Q3LFL9        ScmB n=1 Tax=Alcaligenes sp...   127     1e-27  1  13.0%  97:418     8:283     ------------------------------------------------------------------------------------------------RDSIPSGDAQIFYRRFGTksRPPLVIAHGLSYFSYDWVEIAAELSSDR-EVVAFDLRGFGDSTW-----SQTRSYELNHFSQDIVALLDHLGWSDAVLIGHSMGGRICLVTAGWKPERVRGLICLDFAPDVAAAGRRKVAERIG---------------------RQPERFESIDAAMA------YHGHHGVAADSPLRRRWEAFLRSAPDGWVLK--------------RDLHFRDAFRQVLAGGSAPALPVDLWAIVSDLRMPALFVRGADSDMFASATMAKLLAANDQVSVVEIAGGHALMEDNPGGVIAQVQRFLKTL-------------------------------------------------------    
 871 UniRef50_A9WR45        Epoxide hydrolase n=1 Tax=R...   127     1e-27  1   9.0% 122:421     7:262     -------------------------------------------------------------------------------------------------------------------------LLHGWPEFADSWAEELIALAHAGYRAVAVDQRGY----AVGARPIGVEHYSMEDLVADALAFADAQGARKFHLVAHDWGGMVAWTLASSNPDRLHSLSVLATP--------------------------------------HPKALQKAAENDADQRERLNYVRFFRQDGGVAESTLLANDAAKLRATYGGKFPAELVEQNLVRLTEPNALTATLNWYRGA----TDDHFNSAADRVTTPTLYIWGSDDRALGEVAARATGDFVDgyRFEVLdaSHWLTEEVPEKVIPLLLAHLTAHTAA----------------------------------------------------    
 872 UniRef50_B2TC21        Alpha/beta hydrolase fold n...   126     1e-27  1  11.2% 100:420     8:303     ---------------------------------------------------------------------------------------------------VEAGDVRLAVYMSGPRDAPPIVLVHGYPDSAAVWEPV-RALLDTRYRVIAYDVRGAGASEAPATR----HAYRLERLAADLAAVAgtCGARPFHLVGHDWGSIQSWEAVTDPAFKGRIVSYTSISGPCLDHASFGLRGE------------SEGESDAAARTAPPRTKRPFGSGVRQTLKSWYIFFFHLPWLPELVWRAGAARLWPLWLRMTERVRPA---------PDPAQVRNALNGLNLYRANFIDKLLRPRARHTHAPVQFIVPLRDRYVGPALSLGLEGWLGAYERNEIDAGHWVVLRDPERIAAGIERFVERHRA-----------------------------------------------------    
 873 UniRef50_A0PUM4        Haloalkane dehalogenase Dha...   126     1e-27  1  12.4%  99:428     3:290     --------------------------------------------------------------------------------------------------RVDVLDSAMSYIDVGQGD--PIVFLHGNPTSSYLWRNVIPHLSDVG-RCLAPDLIGMGAS-----GTSPTFSYQFADHVRYLDAWFEAVGieNVVLVVHDWGSALGFYRALRYPEQIAGIAYMDAPVQPRTWAGFTDYEPL-------------------------------------------MRALRTEQGERMALAENVFVEKVVPGGVQRQLTEEEMAVYRKPYPTPQSRIPTLLWAREIPVEGEPADVQAmlSRSDIPKLLIVAEPGAILHEGGSedFARSWPNQREVKVAGRHFLQEDSPDAIGAAVRAFVLDVRERQDGADRA---------------------------------------------    
 874 UniRef50_C5S6R8        Alpha/beta hydrolase fold p...   126     1e-27  1  12.5% 104:431    35:311     -------------------------------------------------------------------------------------------------------DGTVHYREAGA--GPAVVLLHGLFAQKEQWDALLCELAASGRRALAPDLPGYGES-----RDFPLAVYPLEFQAERLHTLILELGLERLDLAGNSMGGTIAAEYAARYPDRVRTLAFIGAPLGATDWSEGVQSAIRAGINPFIPLDRDQFALEMRLLFAAPPEI-------------------------------------------PESIVETAVAEYREHQLHYRQVWDIVNLDADLLQ--TRHDERPHHNPPPPPTLILWGERDGIFAVTAARPFQAHRPRSRLEipETGHLPMLEHPAETAAAYRRFLEAPEPAERDRHRGPGG------------------------------------------    
 875 UniRef50_Q47B21        Alpha/beta hydrolase fold n...   126     1e-27  1   9.8%  92:417     2:289     -------------------------------------------------------------------------------------------SPTSTTEHIDIRGLRYHVRHWGAVDAPKIFLLHGWMDSSATFQFVVDAF-EKSWHVIAPDWRGYGDSEWLGRPYWFPDYY------ADLEALLQHYSPdpAQLVGHSMGANIAATFAALRPHRVARLAMLDFLGLKPEPSIDAPTQ------IGKWLDEIADQPRLRVYPDHAALARRLRFANPRLTAERAEFL-------------------------SRAVSRTTADGSVEMACDPWHKVASPALYR-------VEDAMAAWRKITCPVLMLIADKGFVrtPEFQQRIESFADVQAKDIPEAGHNLQHDQPELVAAELEGFLQR--------------------------------------------------------    
 876 UniRef50_A3K4U8        Alpha/beta hydrolase fold p...   126     1e-27  1   9.1%  88:429     1:307     ---------------------------------------------------------------------------------------MAAEHMKTTRREIEVDGIRTTLYVAG--DGPALVLLhgYMgsAVMWAPVFHRLAE-HFTVIAIDRLGQGRTDNPASA----DDYSLEASKDHALKVIRALGHDRVHLVGHDEGAFVAAQLAIEEPELVASVALVVANALTPGVSRRNVVHA---HPPRPYLGRTSLRWMYETACHSILAVRSEWMN---------------------EAEEIATLDKTGEAIRVMSDGETHLRTYVAQW--TAKRSALHRQFHDTG--------------LPCPTLLVWGLNDPVAPLENGKYLLELlrDTEIRIFnaGHFPHWEHPETFARVVGSYAKEAERHAAGRAASG--------------------------------------------    
 877 UniRef50_C6WTL0        Alpha/beta hydrolase fold p...   126     1e-27  1   9.0% 106:417     2:257     ---------------------------------------------------------------------------------------------------------QLHYRIFGSPTshPLVLLHGLFGSGDNWGAVAKHFAQQ-YQVISVDLRNHGRS-------PHNESQTYADMANDLLELCDALNLDRIHLLGHSLGGKTAMQFATHHPDRLEKLIVVDMAIRAYADAYTHMIDAMLAVDLNAMQSRGDADKALVSAIPH-TTVRQFLLMNLIKIENKLAWRINLAALRNNYPHMIKAICENTHYEKPSLFIRGERSDYVRD--------------------------------------------------ADIQHIKTHFTNAQFASLATNHWVHAEQPQAFIALVDNFLAD--------------------------------------------------------    
 878 UniRef50_Q1ATA8        Alpha/beta hydrolase fold n...   126     2e-27  1   9.9% 114:417    16:259     -----------------------------------------------------------------------------------------------------------------RGEGPPLVLIGGLGFGRWSWFRQVPALS-RHFRTVTFDVRG-----------EQRLRRGVPDLAAEVLALMDHLGLPRAHLMGSSLGGFVAQQLALERPERVNRLVLVGTSPGRGSPKTMSPQALLDMAGWPGLSAERAVRRGLETATSQDYRNRR---------------------------------------------PQEFEHLVRWRMADSPSAATYYEQLRAGARFDSSRD----LGRITAPALVIHGTEDRYVPPANGAALAAGIPNSRLRLLdaGHLVFIERFADVNRETVTFLKA--------------------------------------------------------    
 879 UniRef50_A4FIZ9        Acetoin dehydrogenase, dihy...   126     2e-27  1  11.8%  54:416    71:371     -----------------------------------------------------APVGATIAVVAPAEVPDSEIDEVVAEAQEQLASGEVEEAAGPAVAEVDVGGRMISYASMG--EGpaVVLVHGFGGDKNSWLFVQEPLAE-GRTVYALDLPGHGASTKDVGDG------SVNELAATLIAFLDELGIERAHLVGHSLGGAVVTNAAASVPDRVRSLTLIAPAGIGSEVDAEYLRGFVAAS----------------------------------------TRRELKPHLRKLFADESQVTRRLVDDLLKYKRIDGVQEALETLLGTLLHGEA------------QAIDTGPMLAEADVPLAVVWGRQDAVLPSANASALGDRV---EVRFVdaGHMVHMENPHATREAIESVLR---------------------------------------------------------    
 880 UniRef50_D2BF33        Hydrolase protein n=1 Tax=S...   126     2e-27  1   9.5%  78:414     2:295     -----------------------------------------------------------------------------STVPPASSPFRAAPE-----KIVRANGVDLATQAFGDPAAPPILLVSGAEASMDWwdDFAARLAATGRHVIRYDTRDTGGSTTFPVGAPT---YTQDDLLADALGVLDGYGLAAAHIVGVSMGGGLAQCLAVRHPGRVLTLTLISTSPGGPGGPENPDLPPM-----------------------------------SEKLAKMFADPAPGPDWADRDAVIGYFLDAERVFAGTIPVDEERVRRTAGRAFDRSPVPAAAS---NHWMIEGGEPVRSRLGEITAPTLVLHGTADPLFPYGHAEALAREIPGATLilPGVGhmPPYEVWDTVIPAIGAH-----------------------------------------------------------    
 881 UniRef50_C2AUH1        Predicted hydrolase or acyl...   126     2e-27  1   9.9%  97:430     7:308     ------------------------------------------------------------------------------------------------RFAVLPGGTRICFRVDGPAGGTPVLLVAGLAEDLTTwdRFVSALVATGFRVIRMDNRDCGRSsrQLAARPRRDAYTLADMAADAAQLIDHLGVGPVHLVGRSMGGMIAQTVAARYPHLATTLTSLYSTTGDPKVG--------------------------------------------QPAPSTWALIAAPPPRDRVGAVRAHLRMTAHLAGTAYPIDEAEEAAHAVATFERTAGDAVAGTSRQIQAIQASGDRAAELARITAPTLVINGDRDLIVAPSGGEATAAATPDARHIVIPggHHLPDALALRIADLVVGHLERAAPPSRDPQRRTA-------------------------------------------    
 882 UniRef50_A4XXG7        Alpha/beta hydrolase fold n...   126     2e-27  1  12.2%  86:414     5:289     -------------------------------------------------------------------------------------PGIALDAWRGQRQALDFGGHAIRYWVAGDAEAEPLLLIHGFPTASWDWHRLWAPLAGR-YRLIACDMLGFGYSAKPRG-----HAYSLLEQADLQQALLAHIGERrvHVLAHDYGDSVAQELIARhrLQLASCVFLNGGLFPETHHPVRVQKLLLGPLGPLIG--------------------------------------------RLFSRRKLAQSFARIFGPQSQASEEELDALWQLIQCNNGPAVMHRLIRYIPERrqRERWVTAMQATAVPMRVIDGAFDPISGAHMVARYRELIAGADTVLLDggHYPQLEAPSQVLEHYLQF-----------------------------------------------------------    
 883 UniRef50_A6G2P3        Predicted hydrolase or acyl...   126     2e-27  1  12.6% 105:413    10:266     --------------------------------------------------------------------------------------------------------HRVHYERFGPADAPALVLIQGLGLSGRFWMRMPDMLAGDawRVLVPDNRGVGKSDRPRRP------WTMANMADDVVAMLDAEGVQRAIVAGISMGGMIAQQVALRHAERVAGLILLATWPGLPSGQLPSFATLNDLFGSAIAQ------------RKDPQLIARLLLPDSLHDRSSELLSDWFQLMRETRPSRATFLGQF--------------------------------------GAIASHSTGKRLSAIQAPTRVITGDQDRLVVPVNSMRLADAIPGAQLQipNVGHAIPLLDREVVRRHALD------------------------------------------------------------    
 884 UniRef50_A4FPF4        Alpha/beta hydrolase fold n...   126     2e-27  1  11.0%  49:421     3:348     ------------------------------------------------WTTGVLGAAVTGAAVGVAARSSRIATQRKADDPLADEPLGA-LKPDRQSTVAADDGVPLVVEEIKPSDglTVVLVHGYTLDARCWHFqlPKLTDPRVRLVQYDQRSHGRSGRSSKKRS-----TIEQLGRDLDAVLRATASrpVVLVGHSMGGMAIMALAERQPRLFRDRVCAVALIGTSAGEIGAS------------------GLARPWLSKHNPLTRGLALLAGIQPGLVERARSTGGQLTWSIIRGLAFGDGSVPPKLVDLMAD-------------MIGATSIEVVTDFLDTIGSHDRKAALAGLrcEVLVLGGDADRLTPFSHAEVIAAELPEAELvvEGAGHMVMMERHELVTEHLVALVRRSAPG----------------------------------------------------    
 885 UniRef50_C5V3Z5        BioH protein n=1 Tax=Gallio...   126     2e-27  1  13.5% 107:417     3:295     ----------------------------------------------------------------------------------------------------------LHVDRHG-VSGAPLLFIHGWGAHSGMWGDVLPQLAT-HFKVMAVDLPGHGYSRIEDRGSRIEGSAPSA---------LKGEGDASLVLKGEEFGAAV--------------LPFPLPPSRFSLDRIVDQLSAQFTEPLSLCGWSLGGQVALRWAMRYPEQVQRLVLVAATpkADWPCAMENDTLAAFSDALRQDSAQTLRRFLGLQVRGGERERELLAILRRSQALrpDFVSLQAGLEILRDSDLRDVLVKIKQSALLIAGSCDTLTPLAAMQFMAAKLPQARLvvEGAAHVPFLSHPEEFVKQVADFLHE--------------------------------------------------------    
 886 UniRef50_Q938B4        Haloalkane dehalogenase n=1...   126     2e-27  1  12.8%  88:432     1:306     ---------------------------------------------------------------------------------------MPGSEPYGRLQYREINGKRMAYIDEARGDA--IVFQHGNPSSSYLWRNVLPHTEGLG-RLVACDLIGMGASDKLDG--SGPDSYHYHENRDYLFALWDALDldRVTLVLHDWGGALGFDWANRHRDRVAGIVHMETVSVPMEWD------------------------------------------DFPDEVAQMFRGLRSPQGEEMVLENNAFIEGVLPSIVMRTLSEEEMIHYRRPFLNAgdRRPTLSWPRDVPLAGEPadFGEWLATSDIPKLFIRADPGVIQGKQRILDIVRSWPNQTEITVPGTHFLQEDSADQIGEAIASFVREI-RAGDNLHREAAPE-----------------------------------------    
 887 UniRef50_A7BAE2        Putative uncharacterized pr...   126     2e-27  1  13.1%  84:419     5:300     -----------------------------------------------------------------------------------IPASAIELEGPWTHRHVSAGGAAFHVADMGEGMDHALVLLHGFPEHWWAWRDVLPALAESTSRVFALDLRGFGTSDLTRG------DCDLQQMANDVIGVVRALGVASFSVAGMGIGGTVAWMIGALAPLELRSVAVLSAP--------------------------------------HPLGVQPVIGHAPWAGGRLLQGRLALPTGRERALRSGSLVTTVFRAWASPGNVNALVstYRAALRRPFAAHTALRGLSA-ARKISREERRLLSEPVRVPVLSLVGRDDGAWSALDHAADAQFvpLTQIVIREAGHFLPEEAPQVVAEALSEHVCTHA------------------------------------------------------    
 888 UniRef50_A4ACR9        Magnesium-chelatase 30 kDa ...   125     2e-27  1  13.0%  86:417     5:285     -------------------------------------------------------------------------------------ELPENWPDSDSSEFIESSGLRWHVQIRGS--GPDLLLLHGTGASSHSWVPMARLL-ESDFRVIAPDLPGQGFTELAPAAQC-----SLTGMSRAIAHLLQTLDAKPVVIAGHSAGAAIAACLCLRGSLNPRSVVSINGAMLPFGRAAAPVFSK-----------------AAQLLAASPVFTQLVALHAVPRKPV-----------------------ERMLRQTGSRTPPEMVRCYRDLLGKPRHVAATLR----MMANWNLEQLEANLDRLNPALSLLVCDRDSVVLPAQGEELARRLPGSELhiADLGHLGHEESPEWFAEQVRRAAAR--------------------------------------------------------    
 889 UniRef50_UPI0001C31C18 alpha/beta hydrolase fold p...   125     3e-27  1   9.6%  82:413    45:338     ---------------------------------------------------------------------------------DPAHALLADVPRGERLDLFSRDGTRLTAELFGPADAPPLVLVHGWTCARRFWTCQLHALAD-HYRIVTYDLRGHGDSDAPA-----DGDFSTDALAEDLEAVLEAALPsrALLAGHSLGAMSIVAWAGRFPREVERRAAGVALLNTGIGDLIDESLIFKLPSGMRRAKAVAGGVFL-------------------------------SASGPLPPASPISLRAVQYVALSPSASPAQV-----AFCQEMVLQCRRDVRAGFGRTLSRLDLADALSNLTVPALVLGGMRDRLTPPPHLERMAAALPHLsvELPRSGHMSPIEDPGSVDAALER------------------------------------------------------------    
 890 UniRef50_A8L161        Alpha/beta hydrolase fold n...   125     3e-27  1  10.9%  98:446     2:312     -------------------------------------------------------------------------------------------------RIVPVNGLELAVEVHGDPDGLPLVLVSGMGQHLVGWHplLASMVRRGYRVIVFDNRDVGHSTHLDWHGEPdaPYRLEDLAADTLGLMDVLGLDSAHVLGLSLGGMVAQILALTSPERVRSITSVMSHP-----------------------------------------------GDPTVKPTDEAVDLLLRPSPTNLAEFVLAAEESARVIGSPEFALDVEWLRTRSAVLWERRQNPAGVARQLAAVLVAPNRTEALRALRVPALVVHGTLDPLIPVRGGRLTAAAIPSAELMEIDggH----DLPRQIWSRLLDRLEDVTHHGETVRADAARTRAPDSMVRTPVSPP---------------------------    
 891 UniRef50_Q12FG6        Alpha/beta hydrolase fold n...   125     3e-27  1  11.1% 103:416    11:318     ------------------------------------------------------------------------------------------------------HGITLSCRSAGEKGRPVLMFLHGFPEAAFVWDSLLTHFAQpgFRCVAPNLRGFEHSSAPADVA----AYRPKHLVQDIAALidKPGGQLACLVAHDWGGAVAWNLAASRPHLTRKLAIINSPH--PGTFLRELQHSAAQQAASAYMNFLIRPDAEALLAE--DDFRRLWEFFSNMGAVDGPHAWLTETVR---AQYREVWRLGLTGPCNY---------YRASPLRPPRSGPPHHDPAAAGVTLPREML-----EVHLPTLVLWGTEDTALPTALLDGLGDYVPQMDLRRMGGahWIVHEQPERVAAHLQQFLQ---------------------------------------------------------    
 892 UniRef50_C8WRX1        Alpha/beta hydrolase fold p...   125     3e-27  1   9.7%  98:416     2:264     -------------------------------------------------------------------------------------------------PFAERSDARLYYDVHG--EGYPLLLIMGVGGNIRWwsGFVRRLAEK-HTVIAFDNRGAGQSEADPAKP-----WTLEQMADDARAVLDAAGVGRAHVLGYSMGGMIAQELALRHPAAVASLVLAATSCGGPQMV-------------------------------QPDEVKQEMRSRPASVAERAEWYKRLFFPEAFRAKNEAYLQSAFRLLLRADMPEAV-------------------YQAQLDAVDRWRGSWERLPSLSCPALVMHGLEDRVLPYINGERLAYRIPGARLKLygCGHGFAMQAGSAVLRDVLAFLD---------------------------------------------------------    
 893 UniRef50_A6R0V5        Predicted protein n=5 Tax=E...   125     3e-27  1  12.9% 120:453    39:371     -----------------------------------------------------------------------------------------------------------------------ILFLHGFPSSSYDWRHQITFFITNGYGVLAPDLLGYGQNTSASGADANDpdYKAKTMSADIIALLDHENigPVHAVGHDTGCYLLSRLGNYYPTRLASLSFLEVPYQKPGEGFH--LDAINEMVKQVLGFERFGyvSEGAAGLIEEHVDSFFTLFYPAEADLWIEHLGPTGALETWLRND------RKAPLAPYITEEEKSTQFKIMR--GHYKSALMWYRALVGNINVDdqAQLDSKLYQPVLMLTSQPSRLNIPGAAAGMMKQFanLTVREVDagHWIQLEARDEVREAVA---SKLPV----DAELTRPKQVRHLNLKSLIHSPAPILHTH--------------------    
 894 UniRef50_B8KL65        Putative uncharacterized pr...   125     3e-27  1  10.8%  99:421    17:297     --------------------------------------------------------------------------------------------------YDTADGLRLYARDYPGPDglPVLCMHGLTRNSADFAWIAAHLA-KTRRVISVDQRGRGLS----AYDSNPANYTPVTYVGDMFTLLDKLAIDRVLVIGTSMGGLMSMLMANMQPERFAAVVLNDIGPELDPVGLERIKNYVGKHPQ------------IRTWDDAVAQTRQINEGAFPRYTDEEWLRFTQGLYREVEGVPVLAHDAAIAQAMLDADTNAVPPDLWPLFANMAA----------------------------MPLLLVRGALSDLLAMDCVKKMQELSPGLQVvvPERGHAPMLDEPLAVA-AIDRFFAEVDPG----------------------------------------------------    
 895 UniRef50_D2SBY2        Alpha/beta hydrolase fold p...   125     3e-27  1  12.4%  94:415     1:268     ---------------------------------------------------------------------------------------------MRLEHTFAWRGRRVRWDAWG--DGLPVVFCHGTPWSSALWHPFADALSSR-FRVHLWDMPGYGQSS-----MEAEHRVSLDVQGELLSDLIGYWGLDRPHLIAHDYGGAVALRAHLLHGALYSSLALVDV-----------------------VALAPWGSEFFRLVRDNAEVFAAL-----------------------PAAVHEGVVRAYIEGASHRGLDERQTRMLVEPWLGSLGQAAFYRQIAQADQTY-TDEVQPLYGSLALPVLVTWGVEDTWIPVDRAHSLAEAIPGARllVPGAGHLIQMDAPESLATVLHGWL----------------------------------------------------------    
 896 UniRef50_Q93HH2        Putative carboxylase n=3 Ta...   125     3e-27  1   9.5% 102:417     6:257     -----------------------------------------------------------------------------------------------------VDGAGMTYDDEGPHDgvPLVFIHGWTADRHRWDHQMAHFADKR-RVVRLDLRGHGESGGSAR--------TIDELAGDVIALLDHLEIDRFIPVGHSMGGMIAQTLALAHPERIERLVLVNSISRMTYSRGRGLLMAASTLVPFKLFVAANIQRAFAPGHPREEVREYIRASSATPRDVVMTYYAAMRSFDVLDRVGEIRMPTLLVHGYYD---------------------------------------------IQLPV-------------SQMLRMAKDYPDAVVRIVDAGHELPVEKPAELTSALDRFVTG--------------------------------------------------------    
 897 UniRef50_D0DVZ9        Alpha/beta hydrolase n=2 Ta...   125     3e-27  1  17.1%  99:285     9:185     --------------------------------------------------------------------------------------------------TVNVDGLDVFYREAGDPSKPAFVLLHGFPSSSSMFRNLMPLLA-RDFHVLAPDLIGFGNSAAP---SRESFEYTFENLTKNVAGFLAALKVDQYFLYVFDYGAPIGFRLAMRQPERMLGIVSQNGNIYQEGLGPKWAE------RVKYWANPTPEPRAQYESAFAPETIKGQYLTGEKPGTVGPDGF--------------------------------------------------------------------------------------------------------------------------------------------------------------------------------------------    
 898 UniRef50_D1S7H2        Alpha/beta hydrolase fold p...   125     3e-27  1  12.0%  77:433     3:328     ----------------------------------------------------------------------------RAALWPEHSLPAHHVPPPWPGRHVRLDGTLVHVRDTPapGAEPALYVHGLGGSAQNWTDLAGLLADR-LDGQAIDLPGFGRSE-------PGPRYTIPSFAERVIRWIEHSDRGPVHLFGNSLGGAISVRVAALRPDLVRTLTLVSPAL----PFLDFRRSLQGRMLPLLAIPRGERLAAWRLAQFSPEVTAQQVMEACVADLTRISDQRRQEAIDEIRVRYEAAHYAAAYVRTFRGLVGSFLRSYL----------------------PGEGALWRQAAAVRAPTLVVGGRQDRLVDVRVAPQAARVIPDSRLLMLDggHVAQLEVPRTVARAVLGLLAEATQSLTTGMGESGTER----------------------------------------    
 899 UniRef50_Q607M2        Proline iminopeptidase n=1 ...   125     3e-27  1   9.3%  84:418     1:314     -----------------------------------------------------------------------------------MKPLYPPLEPYVFHRFGVGGGHEIYVEECGNPEGIPAVFLHGGPGSGCR-HHHRSFFDPERYRAILVDQRGCGRST----PHGALRNNTTRHLIDDLESIRGRLNIPKWLLFGGSWGAALALLYAQAFPERVSGLILRGSFLARKRDVDWFVRDGASRFHPEAWQRFSDNFDARERADPVRAIHRRIKGADELEQRRMAKEWWLWSSRVTLGSGFNPADDDPLPPGALAQCRIE------------------LHYAAARYFIREGQILEDCPKIAHLPAIIVHGRQDLVCPPEAAWLLHRALPRSELTipNAGHLAqeEMTDALVRALDGMAERL-------------------------------------------------------    
 900 UniRef50_C6C4Y3        Alpha/beta hydrolase fold p...   125     3e-27  1  11.1% 102:416     6:276     -----------------------------------------------------------------------------------------------------VNGASLVYAQAGESHPQAIIILHGggDHRGDFQAFLP-LADR-YHLIAYDQRGCGRSSLTP-------PLTFDQLVDDLDAVRRQlgDKPVIVIGGSFGGMIALSYALKYPQGLSHLILRGTAPSH---------------------HHEAQAIANFNARLHKAPSASLAMIDKLFSDRVTDDVELR------------LIWLALQPLYYEQFDADAALERTRTLSLHVTTHNAL-------FADKAYDLRHRLADIQAPTLVVVGGEDWICPPAQSRLIAEGIPGArlEIAGANHPVHIEQNARVLAAIRAFLS---------------------------------------------------------    
 901 UniRef50_B3S723        Putative uncharacterized pr...   125     3e-27  1  12.2% 104:341   217:438     -------------------------------------------------------------------------------------------------------GIKIHFVEKGS--GQVILFLHGFPDFWYGWRYQIPYFTAIGFRAIAIDHRGVGLSSCPPNV----EDYSMDLVTED---------IDQAILVGHDIGGDVAWNCCLQYPDRVRAVASLNFPYIPP-HPNMNLLTYIKQNPSPLQYYIYFSESGKFEKCLAKDIRVSFQNIYTSTDENDEKALDGREFNLLDMDCSSYKLKLIEGYQRSCFMTKEELDYYVKAFQVTGFTPSLNRFRNA------------------------------------------------------------------------------------------------------------------------------------    
 902 UniRef50_A0YDP1        Putative hydrolase n=1 Tax=...   125     4e-27  1   8.5% 100:416     9:272     ---------------------------------------------------------------------------------------------------VESNGINIHYHRTG-GKGPALLLIHGFTDNGLCWSRVAREM-QAHYDLVMLDSRGHGLSDKP------HQGYSTDDYADDVAGVIAALNLGTTVLVGHSVGATTAATVAAKYPQLVRGLILEDPIWRDQPGQSD---------------------------SASPEKKAAMVTAVRQQIVVQQQMNAQQ-------------IETSGRENCPTWDAEEFPAWVQAKQQVSPDVAATLVSLRG--------DWQHMASEIQCPTLLITgdKDVVVSPTVVAQAQAINPLIQHTQLaaGHNIRRESFQPYLAAVRQFLA---------------------------------------------------------    
 903 UniRef50_A4FC94        Alpha/beta hydrolase fold n...   125     4e-27  1  15.0% 113:425    28:299     ----------------------------------------------------------------------------------------------------------------GAHDADVVVLIHGIAGSGRTWQPVLEELARTGfrVLAPDMPGHGESAAPRA------EYGLGAFASTVRDILALEGHSHATLVGHSLGGGVALQFAYQFPEMCGRLVLVDSGGLGSE----------------------------------------VALALRATALPGARATLAVAASRPVLAALRGVGRLAAAVGVRASGEARELLRHYASLGDRGRRQAFLATARGvlRGQRASALDRLYLAEKMPTLVVWGERDQLIPVGHGHRAAEAIPGSRVEIFESahFPHVADPARFVAVLSGFLRETTPARLRT------------------------------------------------    
 904 UniRef50_B8G5F0        Alpha/beta hydrolase fold p...   125     4e-27  1  11.0% 107:416    23:276     ----------------------------------------------------------------------------------------------------------ISFLAAGT-QGPAIVFLHGWGAFKELWWSALRDL-GRDYRCFALDMPGHGESRIGRAD-------RIEAIAELVAEFCHDHGLSQIILVGHSMGGSVAVEVTLRDPELVARLALIDAAVDAYRMPVYTRTYLLPNVGWAVF--------------RMTQTIGRTFRPLGQRIPHEHGGGWIRPWLRR--------------------------ASYLATFDPEGLYR--------ILRSLFATRADDRLRQIRVPTLVMTGQLDSLAPPTHARRLAACIPGAQYVMIPAshNPMDERPRAFTRALRRFLD---------------------------------------------------------    
 905 UniRef50_D1WQV5        Alpha/beta hydrolase fold p...   125     4e-27  1  11.1%  98:439     2:291     -------------------------------------------------------------------------------------------------PSAPLNGLDLVYDDSG-GHGEPVVLVNGSGATRTAWrhQTPALVAAGYRVIAPDNRG------IPPNALPPGGITLEAMAADLAALIEHL---------------------ALPPCRVVGFSLGASVVGELLLSRPELVGQAVLMACRARLDTFGGAVARAAADLH-------------------------DSGAAVPASHRAVFQALCYLSPHTLRDGAAVRDWLEVFTYAGGVTGP--GVRAQYEAMGDVDQLPRYAKLDVPLLCVGFADDVMIPAAATREIADTVPGARYAELecGHYGHLERPAEVNALMLDFFARHPRAARPapGRPAPVTASARGG----------------------------------    
 906 UniRef50_A8TSY8        Alpha/beta hydrolase fold p...   125     4e-27  1  10.4%  82:419    18:295     ---------------------------------------------------------------------------------ASARGQADNRPGIRHGVATLPGNLRLPYAERGDPDGHPVIMLHGYTDSWRSFEPVLPYL-PRSVRVIVPTLRGHGDAGRP------DAGYGMDDFADDVAGLMDVLGIGSAVVAGHSMGSMIARRLALDHPRRVTGLVLVGTFAAIRGNPDIEGLWIEVVSGLLDPVSSAFVREFQEGTLARP-----------------VPAGFLETVIGESLKTPARVWRAALQGLLQDDRAADFAGIASPTLLVWGERDAFA-------------------------------------PRADQDALLAGIPGSRLLVygAGHGLHWEEPARFARDVSAFVQDVA------------------------------------------------------    
 907 UniRef50_A7HAN8        Alpha/beta hydrolase fold n...   125     4e-27  1  11.9% 105:416    89:368     --------------------------------------------------------------------------------------------------------LRMAYIDaaAPNGRTVMLLHGKNFHAGTWATTIASLNRAGFRVIAPDQIGFGKSSKPE-----RYQFSFTQLAANTRALLVSLGISRSAVVGHSMGGMLAARYALDYPEATERLVLVNPIGLEDYAALIPPRTVDDWYAQEL--------------KQNPEQVREYQRKAYYDGAWKPEYEEHTRLLAGWTLHPEWPRMAWISALTYDMV--------------------------------FTQPIVPHLGRVRVPTLLVIGLRDRSAptRLGKDTQRRIPGAALVELPggHVPQVEAFPAFEKALLEFLR---------------------------------------------------------    
 908 UniRef50_UPI0001BC9A36 hydrolase n=1 Tax=Pseudomon...   125     4e-27  1   9.2%  93:417    10:302     --------------------------------------------------------------------------------------------TPATHHHIQVNGIELHLVTAG--QGEPLLLLHGTPKTHIYWQKLFPILTP-YFTVIAPDLRGAGLSGHP----YAEKGYLSSTVAEDLVQVLDTLEIEQAYVHGEDRGAEYGFVLAASHPDRVKALSFAEMMLSGFGLEERSFLTEKNVKEAGDHSGVWEWHVPFFFTPDVPE-----MLLAGKAHEFWTYWLNKQ-------------------SYDPTAYPEELKEEWITLLSTPHGLRGTLDTYKATLENArnKSLHGKLSG--KPVMTIGASH------FFGDLVRQSipSHNVIFERCGHGLALEQPLKLANELKHFFKA--------------------------------------------------------    
 909 UniRef50_Q223F3        Proline iminopeptidase n=16...   125     4e-27  1  12.2%  73:401     2:313     ------------------------------------------------------------------------PNSKPKKEPPPMSADALNPLYPAIAPYRtvDdhTLYWEECGNPDGVPALFLHGGPGAGLSPQH-RQFFDPAYYRIVLFDQRGAGQST----PLGEALENTTDLLIHDIERLREQLGIAQWLVFGGSWGSTLALAYGEAHPERCTGFVLRGIFLCTRSEIDWFLNGIRLFFPQEHA----------RFVADIPETERGNLLQAYARRLFSGNPAENMRAARTWSRYEGSCLHLLPH--------PEVAETFGSAAVGLGLGRLEAHYFLHDAFLSEDQLVRNVDRIRHLPAVIIQGRYDVVCPPVSAWRLHEAWPEASFqiEDAGHAAFE------------------------------------------------------------------------    
 910 UniRef50_D2B0U2        Carboxylesterase n=3 Tax=Ac...   125     5e-27  1  10.2%  99:422     3:292     --------------------------------------------------------------------------------------------------TASHGDIELAYETFGPPDGEPLLLISGTGVQMLIfeDLCHALVDLGFQVARFDNRDTGLSTHLADAPAPGwpYRLEDMAGDAVAVLDALGWESAHLVGTSLGGMIAQTLAIRHPSRVRTLTSIMSTPAARIGTMPKMATLKAILK-----------------------ISGMPVTSPDQAAQEAVAMKRLIGSPRYPFDEREVGDIGRRSYERHPGTPEGDARQRAAVAVSG-------------------DRRRALAKVRIPTLVLHGEDDPIIRLRAGRATADAIPGARLVTYPggH----DLPRALWPSILDQIRSLALAR---------------------------------------------------    
 911 UniRef50_D2B9N9        Alpha/beta hydrolase fold p...   125     5e-27  1  15.8%  98:414     5:297     -------------------------------------------------------------------------------------------------RMVNVGDVELWTEEFGDPSHPPVLLVMGAMSQGVLwdEFVGRLVAGGRRVIRYDHRDTGRSD---TVDFDSHPYTWADIKADVLGLLDAYGIGAAHIVGHSAGGLLGQWIAAEQPDRVLTLTAIGSSPLGGREGQVLMRALMGQEQPPGSLPPPTPEFVEFFTKA---------------------AVNPPPAGRREQIDFQIELARVLHGPALPFDEDEQRRVEERLHDRAVDPAAVVNHQRAGTTASDFEPVG-ILHRVVAPTLVIEGTHEP-VKPGHGAFIAQEIPGAELLMIEggHTLPPQVHRQLADAILEH-----------------------------------------------------------    
 912 UniRef50_C7Q209        Short-chain dehydrogenase/r...   125     5e-27  1  10.4% 100:456    10:334     ---------------------------------------------------------------------------------------------------VTVEGGKLSVRTYGDPSKPIVLLVHGYPDTQAVWDGIVAELVDDFY-VVTYDTRGIGASKGP----LFKHGYTLEHLADDLYAVADVVAPDrvHLVGHDWGSIQAWEAVTRADSAQRFLSYTSISGPCLDHAALWTRSGIRKPTPKAAARSL-----------------------KQALASWYIVVFHLPVGPRVAWRLGLAKQWPRIIKAI-----EGTRLDPSPTLHRDGSRGVLYYRANMLPKMR----RPQERSTTVPVQVLTPTGDRFVTPALAAQavPWTERLYVRKVNGGHWIVVKKPALIAERIREFVQsaTPGGPEPVRSLLRAEARQGTKDRReeDKLVVVTGAGSG-----------------    
 913 UniRef50_C5CBG0        Predicted hydrolase or acyl...   124     5e-27  1  11.3% 104:413    18:275     -------------------------------------------------------------------------------------------------------GLTFDVRDGGPAEGEVVLLLHGFPQDATSWRHVEPLLHAAGLRTLAPDQRGY----SPGAAPRATAAYRLPALVGDAVALADAAGARRVHVVGHDWGGAVAWGLATAHPERVASLTVLSTPH----------------------------PAALARALRGPDQLRRSWYIAAFQAPVLPERILSRRLGSLLQRGQVPG-----------------AEHYARRFSTPQSLRGPVNWYRANPDLVTTPRSAP----VSVPTTYVWGTRDAALGPRAAELTAGQVAgyRFVKLDADHWLPEREADAVARAILD------------------------------------------------------------    
 914 UniRef50_B6H4V7        Pc13g11500 protein n=1 Tax=...   124     5e-27  1  11.1% 119:416    82:347     ----------------------------------------------------------------------------------------------------------------------TAVLLHGGNFCGVTWSDTARQLSAAGYRVILPDDIGFCKSSKPQG-----YSYNVQQQALNIHSLLTALGIDQVTVIGHSMGGMVAARYGLMYPNNVQQLFLVNPLGLEDWVAKGVPYLSVDASYESGVVSNFTTLKAYEHANYFP------------NAPWKPEYDAWVHMLADIYAGDYGSEYSYVMALVTDALLSQPVIYQLPLLQTRTYLLVGDSDVSALGKAWSSPAVAAKLGHYH----------------ELGPAAAAMIPNCKLHMFPggHAPFLQDPKAFYDVLLSWLD---------------------------------------------------------    
 915 UniRef50_A8I111        Alpha/beta hydrolase n=1 Ta...   124     5e-27  1   9.7% 100:420     9:269     ---------------------------------------------------------------------------------------------------IEIDGLTFHYRVDGPEGAHWLVFSNSLMTDLSLWDDQAAAFGE-SFRILRYDQRGHGRTGIPA------EDCTMERLAADLIGLMDALGIARTHLVGISMGAATCLGAALAQPRRVASLVMADGQAATPTGGAQAWEERIALARTQGMAGLADPTIARWFQPASVETNGEGVRRVAGMIAATPLDGFLR------------AVRALQRFDFRPGLTA-----------------------------------------LACPALMLAGAADGALP-KTLAGLAEAVPDGRFviPDAGHLPNIEQPGAFNAALATFLKRGAA-----------------------------------------------------    
 916 UniRef50_B1LSP8        Alpha/beta hydrolase fold n...   124     6e-27  1   8.4% 100:433     5:287     ---------------------------------------------------------------------------------------------------TTDDGIRLHYEESG--EGTPLIFVHEFAGDHRSYEAQLRHF-GRRYRAVAFNARGYPPSDVPEAVS----AYAQARAADDILAIVDHLGAPKAHVAGISMGAFATLHFGLRHPDRALSLCLGGCGYGAEPERQALFRAEADATAEMLRRDGMAAFAERYAVGPTRVQFETK--------------------------------------------DPRGHAEFKRMLAEHSALGSANTQAGVQKGRPSLYDLTDELGRMTVPTLIVAGDEDWPC-LAPSLMLKRVIPSAALAmpNTGHALSVEEPDAYNRLLDDFLAQVESGRWPMRDPRATSA----------------------------------------    
 917 UniRef50_UPI0001C31949 alpha/beta hydrolase fold p...   124     6e-27  1  11.2%  94:433     1:307     ---------------------------------------------------------------------------------------------MSERLYDVGRGVTLCAEGFGDPADPPLLLVMGLGMQMIRWHafVRELTDRGLYVIRFDNRDSGRSTFMKDVPLppRQYLLADMARDTVALLDALNLDRVHIAGASMGGMIAQTIAAEQPRRVRSLTSIMSNT-------------------------------------------GSRFSGQPALKTYPLLLRRAAPGRDGYIAHQVALHDAIGSPGSPADDPLFREIAECSYDRNPDPTGTPRQLAAVLASG---DRTRALHRVSAPTLVIHGDADRMISISGGRATARAIPDSELVVVPggHDLPRGVWPEVIGALAHHVRAAERAGRAGRANDAPTR----------------------------------------    
 918 UniRef50_A6V1Z6        Probable epoxide hydrolase ...   124     6e-27  1  11.0%  83:419     3:293     ----------------------------------------------------------------------------------AALPGIPLEAWRAQGRSVDFKGFPIRYWEAG--QGKPLLLIHGFPSAAWDWHYLWEPLARR-YRVLACDMLGFGDSAKPRG-----HRYSLLEQADLQQALLGRLGIDQphVLAHDYGDSVAQELLARHHEGRLRLASCVFLNGGLFPETHRPVLSQKLLLSPLGG-----------------------------------------LFARLFDRRALARNFARVFGPRSQPGEAELDAFWSLIESNQGQRVMHRLIRYidRREQRERWVAALQHGGVPLRVIDGAVDPISGAHMVERYRQLVRDADCVLLDggHYPQIEAPGAVLEHYLAFRARVD------------------------------------------------------    
 919 UniRef50_C6WLW9        Alpha/beta hydrolase fold p...   124     6e-27  1  10.6%  52:428    13:362     ---------------------------------------------------GVLGAAATGVAVGVAANHRKVTGERNQDDPLADEP-LGRLRPTRESTVAADDGVPLSAEEVDPEDglTVVLVHGFALDRRCWHFQRRDLadPRVRLVLYDQRSHGRSDRSPA-----ESNTIDQLARDLDAVIRSMApgPLVLVGHSMGGMTIMALAEENPtrVRGVALVGTAAGAVGGAGLPKSVLSRYNPVTLGVGRLAG-----------------------LQPSAVEWARRRANILTWSGIRALAFGDRGVPTSLVDLMDEMIAGIK------------VSVLTEFLETLGTHDRYKALAGLrcEVLVVSGDADRLTPFSHAERMAEEMPHSELVRagAGHMVMMEQAELVTAHLVDLVRRSAAVEDEDGRR---------------------------------------------    
 920 UniRef50_Q47J59        Alpha/beta hydrolase fold n...   124     6e-27  1   8.0%  94:416     3:295     ---------------------------------------------------------------------------------------------PSRSEYLDLPDIRLHIRRWGNPKAPTLFLLHGWMDVSASFQFVVDEL-QKDWNIIAPDWRGFGSSEWLNRP------YFFAEHLGDLEAILDRYAPekVKLVGHSMGGILACLYAGIRPERVESVISlgIAPTTPDMATERYQKWLGVLKSPPRMHLYSDRAGFARRLMHTDRFLTQERANFLSR--------HLARIGDGETRSGERRHGIIWNGDPWHKAPA-------------------------PYLFRLEESMAIWRQITCPVLWVAGRQSWVirPGDWAARQACFADVREAWVdaDHMLHHDQPEEVARIVETFIA---------------------------------------------------------    
 921 UniRef50_D0MFA2        Alpha/beta hydrolase fold p...   124     6e-27  1  11.5% 106:419    19:273     ---------------------------------------------------------------------------------------------------------RLHLAVQGPEDGPPVLLLHGWGSSAWLMAPIAEALADR-YRVFNVDLPGHGLTPPPPAP------WGVAEHAALVAELIRTRIGRPVVLIGHSNGGRIGLYLASEppELLRALVLISPSGMRPRRPWHYYVR---------------------------ATLARLLKAPFQVLPQPLREPALDWLRHTVVWKALGSSDYRRLEGVMREVFVRIVNTYVE----------------------------DRLDRIRIPVLVFWGERDRAVGRTQMERLVQALPDAGLVVlnAGHYGYLDDPDTFIAATRYFLEHLP------------------------------------------------------    
 922 UniRef50_C4LHK4        Proline iminopeptidase n=1 ...   124     6e-27  1  11.8%  82:403    17:338     ---------------------------------------------------------------------------------PETTPFNEGTVERPVDPDSPASTQHIAYAEYGNPEGIPAVFIHGGPGGGTS-AKEARFFNPDKYRIILIDQRGCGKSTPHLADPDTNIDatTSELIGDIEAIRETLGIDKWVVFGGSWGSTLSLAYTQAHPERTLAIVL--RGIFMLRRTELDWYYNGGAAYMFPDKWERFLSVIPDDKKPEPLNPAGMTHLPDVNLIDVYHELLRS---DDHDTAVAAARSWSVWEGSTSYLHDQPTDTHEDEWFALAFARIENHYFVNHGFLEGGQLLRDVDKIREIPAVIAQGRYDVVCPPITAWQLHQAWPEATFVwpTSGHASYEEE----------------------------------------------------------------------    
 923 UniRef50_B9L394        Alpha/beta hydrolase fold p...   124     6e-27  1  11.6%  96:424     4:271     -----------------------------------------------------------------------------------------------RERVETLGDLWLWVAEAGPSEAPPILLLHGLYDRWETWEPVVPALAER-FRVIAFDMRGHGRSSQPAG------GYTLRDYADDAVRLL-----------------------ARLRPSR----------------------------PIVVIGFSLGALVAIVLAAEHPELIRGVVLVDPPLVEPDEGTRLWLRALLEAKRAGIEAAYELARELDPDGTPEEWQQSALWLCSTADGP------ILALLDEGRRPDPWELLPHLSQPVLVLQADpgGVLDDETAQRALAQLRQGELvmPGCGHAIHRTCPDDFLEVVLEFLARLDRPATG-------------------------------------------------    
 924 UniRef50_B2HDF1        Haloalkane dehalogenase n=1...   124     6e-27  1  13.8%  92:417    12:291     -------------------------------------------------------------------------------------------QHRASGRRFTAAGIESFVLDSGARDAPPVICVHGVPASSFLYRKVVAAMAQRGLRGIAIDLPGLGLAERP-----SDADYTWSGLGRWLGAAIDALGIGRFHLVVHDIGGPIGFDVANAAPDRVLSMTLLNTII--------AVQRFHRPWPMEPFAHRAVGEMWLGLLR-----------------------------------VPGAFESIMRLVGVSSRVPSAEIACWKPLLFGDDGGRAFLEIMRGFELTPeqHNYIAAVQDTRYPVQIVWGVRDTMlwRRYGAEARQLAGTQRVTTLPAKHFLQEDFPEEIAAAVQRLAQS--------------------------------------------------------    
 925 UniRef50_Q988D4        Alpha-(N-acetylaminomethyle...   124     6e-27  1  12.2%  91:417     8:278     ------------------------------------------------------------------------------------------ASDHFISRRVDIGRITLNVREKGS--GPLMLFFHGITSNSAVFEPLMIRLSDR-FTTIAVDQRGHGLSDKPET------GYEANDYADDIAGLIRTLARGHAILVGHSLGARNSVTAAAKYPDLVRSVVAIDFTPYIETEALDALEARVNAGS---------------------QLFEDIKAVEAYLAGRYPNIPADAIRIRAESGYQP-------------------VDGGLRPLASSAAMAQTARGLRS--------DLVPAYRDVTKPVLIVRGESSKLVSAAALAKTSRLRPDlvVVVPGADHYVNEVSPEITLKAITNFIDA--------------------------------------------------------    
 926 UniRef50_B1JF37        Alpha/beta hydrolase fold n...   124     7e-27  1  11.7% 103:417     7:265     ------------------------------------------------------------------------------------------------------GPHRHYYYDSGA--GSPVLLLHGLGNSGRAWAPQASALLSLGHRVIVSDLLGHGASS------EAPEGITAHAQALEMLALLDHLGLESTHLIGLSLGGMVALEMACHAPEAVQQLVVAGTFASMNSTYRQGLLNDWAAN------LAQADGCLKRFKATWPALVGADYAASAEGQCLYQAWHAQAAQLS-------------------------------------------AQSQIRWCNGMKRYDLSNQLPAIQAATLVLAAEGDLISPRSEAEDITQRIDHARQITLPggHVFNIPYAQAFNQAICTFLQE--------------------------------------------------------    
 927 UniRef50_Q5WI80        Epoxide hydrolase 1 n=5 Tax...   124     7e-27  1  12.4% 102:416    75:378     -----------------------------------------------------------------------------------------------------IDGIDVHFveRGKGPDppLILTHGWPDSSLRYQKIIPLLTddSFDVIVPSLPGFGFSSRPKHPRVNNF-----RVAEMWAKLmeKLGYSKFAAAGGDMGSGVTRYLAANHPERLYGIHLTDIGIIRdsSDQGTLSEEEWQYKNKASAWMAQEGGYMSIQSTRPQTLAYGLSDSPVGLAGWITEKFRSWSDCNGDLAQKFSEDELLTHIMVYWVT--------------NTIGSTAHMYYDNAHSLPP------IGYIEVPTGLALFPADILLPPKE-WAMRNLNVTRWTSMPRGGHFTALEEPELFADDIRAFFR---------------------------------------------------------    
 928 UniRef50_B8LVK9        Hydrolase, putative n=1 Tax...   124     7e-27  1  11.5%  54:411     3:328     -----------------------------------------------------LKTFSLFLWPFWLVFAPAATTKAVDAGNTSLPWHLTPLKYANTSTL-----LNVAYYEAGPPNGSAVILVHGFPYSIDAFASVVPLLTGKGYRVIVPYLRGYGETSFL--YPDTPRSAEQAALGSDLIALMDAIHIDKAIFAGYDWGTVVVNVAAALWPERCNGHVAANSYLIQNRSTAWVPLDPDSEALRWYYYVFLTARGAAGLASNPKAWARSLWQKNSIG----------------------------------WNFSEEYLDLTATAFENTDYVDIVLNFYRNRLLYAPGDPKYSAlpPISARSVTFDPQNSVVFPPTNGTATAKYFTGSryVLPNVGENVPYQAPKMFANAI--------------------------------------------------------------    
 929 UniRef50_Q0RQ32        Epoxide hydrolase n=5 Tax=A...   124     8e-27  1  11.9%  97:424    75:391     ------------------------------------------------------------------------------------------------HVLTTVDGTPVHAIHARSPhaVPLLLIHGWPGSVIEFLDVIDRLVdeAFHVVCPSLPGYGWS-----GPTREGGWHIRRVADALVELMAQLGYPRFAAQGGDWGGIAASLLGAHHPDRLLAIHllVLAPPPDEATMAVLSAPERAALEKVVRFTETETGYQAIQGTKPQTLAHGLADSPAGLAGWIVEKFRAWSDCDGDVEKAISRDDLLTNITTYWVT--------------GTIGSSVRLYR---ESMRAGLFGPPDRPVTVPTGVAVFPREIVTPP---RRCVEAHYDLRyeLPRGGHFAALEQPELFAEDVRAFFRLLRGGAHE-------------------------------------------------    
 930 UniRef50_C4RK21        Hydrolase/acyltransferase n...   124     8e-27  1   9.2%  96:420    25:340     -----------------------------------------------------------------------------------------------RHEFAELDGIVLSYLR-GPEAGPPLVLVPAQMGTWTTYAKVMPALAER-FSVFVVELRGHGNSSWTPG------NYCWDSVGGDLRLFLERMVGRPAVVSGNSSGGILALWLAANAPGLVSGIVLEDAPVFSVEMPRFAERDRFVYNGLAHIVEVLGDRE-----NRHLADYFRGQGLPVSERRTKRMPGWFVDLLDRAITRYEQRHPGQPSGADAWWLPASMGELFRSlmFDPDFARAFVD-----RRFYGDFSHEAALRAVRCPLLLLHaeHGLvmDDDDARRVCALVPGAVYRRIPANHVIHRRKPEEFVRAIEEFIAEIRG-----------------------------------------------------    
 931 UniRef50_C1DG75        Alpha/beta hydrolase fold p...   124     8e-27  1  12.1% 105:425    14:301     --------------------------------------------------------------------------------------------------------LHLHYADSGPADGPVAVLLHGWPDSILGWSDLVAALNAAGWRTLVPFLRGFGPNRFLAPDTPRSGQAC--AIAADVEALADGLDIERFALIGHDWGARASYALAALRSERIERLVALSVGHAPPAP------LGLRQAQNYWYHWYFALPRGEAALREDPRALCRHLWQSWSPGWAFP----------------DSLFEAAAQAFDNPDWIAVTLHSYRQRWGFAAGDPAHATWEGRLAQGLP----------IPVPTLVLHGADDRCNDPSTSADASLFVGrrRLLLPGVGHFPQREAPETVSAAVLDWLGTPGQAGNRA------------------------------------------------    
 932 UniRef50_Q9XD79        4-carboxymuconolactone deca...   124     8e-27  1  11.8% 107:471     6:298     ----------------------------------------------------------------------------------------------------------LNFRAEGSDSAPPLLLGPSLGTSYTLWDKVAPELS-ITHRVIRWDLPGHGGS----APGLIGPGATVGDLADLVLALADSLGIDQFAYAGVSLGGAVGLHLAVHHPERVASLAVICSSAHFNGAKPWEERAARVRSEGVGWLVESADSRWFTPGFTVPELVADQRDADPGAYAACCRDALAAFDLRERLSEISAR-----TLAVAGREDPATPPAHLREIADAVPGAGLVE-----------------------------------------------IPGA-------SHLAPAQCPEAVLTALRAHL--------GGGAKRGMEVRREVLGDTHVDGSQSRQTPFTARFQDFISRYAWGEIWT--    
 933 UniRef50_A4EGL4        3-oxoadipate enol-lactonase...   124     8e-27  1  11.8%  99:472     3:306     --------------------------------------------------------------------------------------------------VALINGAPVAYRHR-PGLGRTLVFSNSLGSDQSIWDKVIAALP-----------RGYG--------------------------------IVTYDLRGHGQSGGTA------------------DSIEGLADDISQLIDQLGLKNVLFCGVSIGGMIGQVLAARRSDVVRGAVLSNTAVQIGTAER-----WTTRISTVEHQGVAAIAAEIVGNWFGPEYaqTDRMALHQTMVARTTNAGYAAACSAIQDADLQGMAQAIKVPVLCVGGTHDQSVQAEAVARLSASIPDAELHIMDggHLPCLEAPAQLAALITAF-----DPRDKDTATAGLETRRAVLGGAHVDQAADKTTTLDQAFQTLITTGAWGQVWAS-    
 934 UniRef50_A1K4P7        Proline iminopeptidase n=2 ...   124     8e-27  1   8.3%  84:431     8:324     -----------------------------------------------------------------------------------MATQPSSPPPPCRDGLLDVGdhTLWFEQCGNPAGIPLLFLHGGPGSGCSPRH-RDLFDLTRFRVILFDQRGCGRST----PRGGLDANTTAHLVADIERLRAHLGIARWLVSGGSWGSTLALAYCARYPAACLGAVLRGIFLARREDIDWFFEGAAPRLPEAHAAFAACAPGPGRLATRVFDAVLGADDARALQVVRHWMQWEESLTLGRAAPLPVLGDDDAARMLDKYRVQAHYLRH--DCFLGHDGWRGLAQQIAAAG----------------LPVTVLHGQDDRICQPAAAQLLRATIPHSRLTMVAAGHDP-------FAPAMRRAL--LAACADPLPPLPGP------------------------------------------    
 935 UniRef50_C6B6D0        Alpha/beta hydrolase fold p...   124     9e-27  1  12.1% 107:428    11:295     ----------------------------------------------------------------------------------------------------------ISYEELGEGD--PIVFLHGNPTSSHLWRNIMPGIGPGRY--LAPDLIGMGRSGKP------DIGYRYGDHIAYLDAWFDALDLDDVVLVGHDWGGALAFDWASRHVEQVRGIAFMETILRPMG-----------------------------------------WYDLPGGGKARYELLRGAGTGETKVLDENFFIEQALRATTLKGLSDADWDVYRAPYPDRNSRRPLLEWPRAMPINGEPADVVARIEAYdtPKLLLTFdeTLLIGSEMISWCRDNIAGLEIR--GCghIAPEDQPEAIAAEIKSWIDrlRTAQRKVASQ---------------------------------------------    
 936 UniRef50_Q2G8L4        Alpha/beta hydrolase n=1 Ta...   124     9e-27  1   9.3%  94:427     7:289     ---------------------------------------------------------------------------------------------MTTIRIPVSGGLHIAADELGPRGAPTVILGHGGGQTRHSWDRAGHELADAGYHVINYDLLGHGDSCW-----EPEGDYSYQRRAADLAAIVRYAGPVFAFVGASLGG--LSSMAAACHGTVPKALVLVDVVARLSEQGVERIVG---------------------FMTANPDGFASLEDAADAISAYYPDRPRPSRLDGLRKNLRLGDDGRFHWHWDPKFLTGG----------------------RDHGSIPDMLDTAEWTA--HVPTLLVRGMKSDIVTDEGVSDLSARIDRLEVADIgaGHMVAGDRNDLFNAAVIEFLERVMPATAPNPA----------------------------------------------    
 937 UniRef50_B1KEG5        Proline iminopeptidase n=2 ...   124     9e-27  1   8.2% 105:397    27:308     --------------------------------------------------------------------------------------------------------HQLYVEECGNPEGVPIVFLHGGPGshRCYFN--PEL----YRIILLDQRGCGRSKPYACLENNNTDY----LIDDLEQIRDRLNIDKWVVFGGSWGATLALVYAEHYPERVLAMILRGVFLGRQQDIDWVYSDGGAANVFPEAwqLMSVLTDDEQLMPLKSLYALLVGTDYIRRSQVLQAFNQWEQQIVTLREESFSLTEVLAETLPEVMISEPYE---------GAAAIIQLYYSLKLCFIEHKPILSSIDKIriPTYIVHGRYDMVCPLRQAWTLSKAWPEARLTVLpaGH----------------------------------------------------------------------------    
 938 UniRef50_Q21NB3        Proline iminopeptidase n=17...   124     9e-27  1  10.2%  88:412     5:306     ---------------------------------------------------------------------------------------FPEIKPYATHELAVDDVHTLYVEESGDPGGIPVLFVHGGPGAGCS-KHDRRFFNPELYRIILFDQRGAGRS----KPHAELEHNTSQHLVEDMEKIREFLSVDKWVLFGGSWGSTLSLLYAQAYPQNVLYMILRGIFLCREQD-----------------LQWFYQAGADRIFPDYWQDYLAPIAENERDDMIGAYYKKLTGSNELAKMSAAKAWSQWEGRCATLRPNPDVVDRFTDPHMAVSLARIEAHYFVNCGFMSPNQIINNAQTLAGIPATIIHGRYDMVCPLDNAFALAEAWPTAKLHIidAGHSSSE--PSVVDALVR-------------------------------------------------------------    
 939 UniRef50_UPI0001C31F64 alpha/beta hydrolase fold p...   124     9e-27  1  10.1%  95:400     4:262     ----------------------------------------------------------------------------------------------PRERFAELTKVTACYRDEGNENGEPLLLVMGLSSQLIHwqEIVDALGERGYRVIRFDNRDSGLTRW----KVTPAQYFLQAMADDAVELLDHLGIEQAHVVGASMGGMISQLIAIGRPERVRTLCSIMSTTGDPSVG------------------QPADGVIGELLRPLPE------------------------DPREAIIQIAHVYDVIGSRTHAASERPGRLELAGEAYRRAVHPDGGARQIAAILMAL---DRTRSLQALRVPALVIHGGEDPLIDISGGEATHAAVAGSTYllPDMGHDLP-------------------------------------------------------------------------    
 940 UniRef50_Q1D2H8        Hydrolase, alpha/beta fold ...   124     9e-27  1  10.6%  97:425     3:290     ------------------------------------------------------------------------------------------------ERIIESGDIRLCTEGFGREGDAPVLMLMGAASSMLGwdGLMEQLARAGRFVIRYDHRDTGRSSH---GVPGATPYTLDDLAADVISVLDGYGIERAHLVGMSLGGLLCQLVALKYPERVLSLTLISAQIFSEPDFEDPGMDP--------------------AVLAHFQRAATLNWSDEAEAIGFQVELSRLCVGRARRSFDEARVRARAVQDYRRALAPQ-------CALNHAGLSGGLEWY-------------GRTREIEAPLLVIHGSVDPVIDHAHGVALSRAVKGACLVTldAGHDLHPDDWETMTRAITAHTSAASPPHGAR------------------------------------------------    
 941 UniRef50_A5FQ95        Alpha/beta hydrolase fold n...   124     1e-26  1  10.7% 101:418    11:317     ----------------------------------------------------------------------------------------------------DTGEVLIHYL-AGPDNGPPLVLIPGQGLSLESYQRVMTPLA-KNFQVFAVDVRGHGKSGWTTG------KYNFPNMGNDFKIFLEQVVRRPAIISGNSSGGLIALWLAANVPELVSGIILEDTPVF--SAEWPRLRDDCWVYRIFKRNAETIGSAEGRNLAAFFKGME--VPIEGKQRVIQFPVWLTGAMAGMVRLHQWLKPGKPVDLALLPAETRMLIKGLSQY--DPDFTKAFVD-----GRACEAFDHAEALKKVKCPVFVlwFRHPDFGLVEAMdhLLALTPQAQFARITSGHMIHFEQPPEYLKHLNAFAAGI-------------------------------------------------------    
 942 UniRef50_D1S7G2        Alpha/beta hydrolase fold p...   124     1e-26  1  11.4% 100:415    18:278     ---------------------------------------------------------------------------------------------------IDINGAQLAYDESGT--GTAVVLLHAGIADRRMWRGQVPALAQR-HRVIALDLRGYGESELPTTPFAHH---------DDVVGLLDALGVERAALVGCSFGGRVAVDTALAHPERVSALALFGAPVSGNE-----WSEETEQLWEELVGDVDPEDFAATAA--------------------------------------GEVRFWVVGPTRRPEDVDPELIRFAEEMDRRAL--AAEQALSAIEVGELDPPAIDRLGELRVPVLVGTGADDLADIRRLADRIADEAPHG-VRMPdaAHLLPLERPTETNATLLPFL----------------------------------------------------------    
 943 UniRef50_D2PUK6        Alpha/beta hydrolase fold p...   124     1e-26  1  10.0% 100:428     3:300     ---------------------------------------------------------------------------------------------------TEIRGTRLYVDPRGAADAPPLLYIHGGPggSYDfaYQGDRLAER-LRVIGVDQRGVQQSD------PIDEPATEEDVIADFEALRDQLRVDHWAVLGHSYGGRLALRYAVTRPETVSKVIFENPPWDMVLATRTLVEAA-------------GPLLSELGLENEAKRLLAHQGPATSETWDERIALLHQLGDRRMEVYLGPTSHGLR---LPSNDLPEEVQERAGYFAETITRAPSFN-----------ESLLPYLAQLTQPALLIKGEYDPVTSSEEIARFQADAPNATYRQVDggHFVHAEQPAEYAELVTAFVLGSAAALQQPVRD---------------------------------------------    
 944 UniRef50_A9VXC7        Alpha/beta hydrolase fold n...   124     1e-26  1  12.7%  92:413    98:374     -------------------------------------------------------------------------------------------ARKPPGQFVTVDGLPVHVIVRGR--GRPVVLVHGNGTMAEDFagLIDRLAAR-YRVIAIDRPGFGYSGRPRHRI-----WTAQAQAVLLHRVLEQLGVERPLIVGHSWGTIVALALAADGRRPLRGLVLLSGSYFPGHRTDAALIAPLAV-----------PGLGDAMRAMVPAKVSASLTGQSFRQVFWPQPVPARFTARFSIEIAAAATQLRAVSEDAASMSSAV------------------------------TGLQRRYAGLTLPVSVLAGDADAIVKaeHSVRLHTTIPGSTLRLLPggHMIHYGARLKVERAIDD------------------------------------------------------------    
 945 UniRef50_A8KXK6        Alpha/beta hydrolase fold n...   124     1e-26  1  13.9%  89:425    19:324     ----------------------------------------------------------------------------------------ASGEAGMVSKWVDLDG-PVHYAEFGAPgsPPVVCVHGLGGSYTNWLALAPLLASTS-RVLAPDLAGHGRT------PLGNRGADVSANHLLLDRFLAEVVGEPVILVGNSMGGLISMLQAVRRPESVCGLVLLDPA----------LPLRRGGWPEPLVVASFAAVVLPGLGAWVLARRRARV------GPAGIVAQTLRLCTTDPARIPSVAVEALVDLGQERAGMDGVERAYVAAARS------------VVRRVVRGGPLRRLIRQVDVPTLLVHGSDDRLIPVALARDAAGLRPDWQlvVAGCGHLPQLEDAAGTAGLLTGWWEQTRDASGGA------------------------------------------------    
 946 UniRef50_A4EUQ3        Esterase, putative n=3 Tax=...   124     1e-26  1  10.9%  86:426    30:326     -------------------------------------------------------------------------------------AREAREAVPPIGHFLDTAKARIHYVDLGPREAPPLVFIHGLSGQLQHFTYALTDLLSDEFRVIALDRPGCGYSTR-----SSDAMARLPEQADILLEFLRRLDIHQPTLIGHSLGGAVALAMALQAPEDIRGLALLSPLTHPAAEGPEAFKGLIVHSSLIRHL------MAQTVAIPTAQRTAGPVLDQIFAPERCPDDFLTKAG-GILGLRPEAFVAASADASFLYPVIANQAASYA--------------------------------KDLQCPGAVLFGAEDRILDPTHQGTSMEALGYPCQLLPERGHMLPITSPKACEEFIRNTVSKLPAYAHKDA-----------------------------------------------    
 947 UniRef50_C8SHZ0        Alpha/beta hydrolase fold p...   124     1e-26  1  11.1%  84:434    30:331     -----------------------------------------------------------------------------------WIAAKAERLVPPVGKFIEVDGNRIHYVDVG--DGQPIVFLHGLGAQLHHFRHTLFAHFGPGYRLIALDRPGSGYSVRARGATG-----RLPEQAELVRRFIETLRLEKPLVVGHSLGGAITLTLAVEHPEAISGIALLAP-----------------------LTHMQAGMREKSGLLNIPSPLRRWIMAYTVAIPTSLRYARPTMEFIFAPQAFPA---------------DYMVDGGGWLGLRPAHFQATSADVVAIERDLG--RIEQRYGEIAMPAGILFGTSDQVIDIghGEPMQGKIKGLGFEPVDggHMPQFVEPERVAA----FIQRMAAHAFCDATSPPLHKE---------------------------------------    
 948 UniRef50_A4RUY3        Predicted protein n=1 Tax=O...   124     1e-26  1  13.0% 100:430    11:312     ---------------------------------------------------------------------------------------------------VDGGRVRLRAVVGGarDRPVVVLVHGWSGTAAYWSPAMRAARDDFPATIAFDLRGHGGSDRP------GRGHTVARLAMDLREVLEqlSPSGFVLVGASMGCAVVWSLLQLCGARWRVGGGSDATEVSVIGAVLVDQAPLQNRRPDWELGSLGCYDAASYEALAAAVRRDMSTFADGNAEACFHDL-------------------------------SVVDPDVLAMVKSETLECCPEALCELMYDHTHQDWRSECRHMTIPALVLAGRETKIFPIDGIKAVAEMIPDSELKIFecGHWLYLEEPKKFADEVHDFVTRCDDKQRAATSEGS-------------------------------------------    
 949 UniRef50_Q0AST3        Alpha/beta hydrolase fold n...   124     1e-26  1  11.3%  92:419     4:290     -------------------------------------------------------------------------------------------AHFTDHFFETADGPRIHWRDygKEAGLPVICLHGLTRNVRDFEDFAPMVAATGRRVISISSRGRGQSDHDPVVT----RYQPPTYAGDVMALLASIGLSRAVFVGTSMGGLMTFIIASLQPSLVAAAVINDIGPEIATEGLDRIKSNVSSRDPVTSWADAAARTRQSNLAAFPKR---------DGDAAFWDDFARKTWVEREDGSIALAYDGALVDQMEDGGVLPDLWSFWAPFA-------------------------------DIPTLVVRGGISDLLTPATVAEMKRRKPdaTVEVPDVGHAPFITEPEAW-QAVEAFLGRVD------------------------------------------------------    
 950 UniRef50_C6QED9        Alpha/beta hydrolase fold p...   124     1e-26  1  10.9%  46:422     7:350     ---------------------------------------------FKLLFGGLIAVVLFGIGNMDGKAQGGATHEQAQAQDADASPHRSG--------FVEGDGVRLNYLDWGS-DGPPLILIHGIANSPHIFDELAPLLRDR-FHVVAYARRGHGQSEAPVGP------YDSNALVGDLVHLLDNLKIERANFLGWSMGGNEVTEFAGRYPDRVEKIVYLEGGYDWSDPA-----------------FFKAFTEMLAVNSPKPETLASFDALKAWYRAAWIGRDVQWTPALEAFLRDAVRIDADGRVDPVpeVFAALHQTLGTWQRDYAKVRAPALAIYGSSFFPEDASGTDLARKVR------DFEQSAATPFRRaeRIQRELRDVqlELPDRSHmiGVIAPDVLAASIRAFLLSGSAAR---------------------------------------------------    
 951 UniRef50_C3JWA1        Hydrolase, alpha/beta fold ...   124     1e-26  1   9.2%  95:420     5:266     ----------------------------------------------------------------------------------------------TEGFITVADGERVWYETAG--EGPDLILTHGLGGNAAVWYQQVPYFA-QHYRVISWDQRGFGRSTNENGNHGPVAAVS------DLIEIMDLLDVDRAHVVGQSMGGWAALGTAIAVPDRVESVVLACTTGGIPVGFGPDSAPPL-----------TAAPVTARPLGEHPAVGGRLPSLDMA----------------------RAYLYQALGTFGHRPPDTEFFRILKAHNYSPSDLAAV-----------------------TAPVLLIAGELDDLMTPERIRSAAEFLPHAEVVEldRGHSPYFEDPHAWNELVAHFLADVRA-----------------------------------------------------    
 952 UniRef50_A4AG85        EphB, putative n=1 Tax=mari...   123     1e-26  1   9.3%  95:416     4:297     ----------------------------------------------------------------------------------------------PALHSVRTPSLSISYESVNDDAETTVVLLHGFPYDVRAYDEVVPILAKRGLRVIVPYLRGFGQTRFASSAIVRSGQ--QAALAQDLIDLLDALGIEKAVVAGYDWGGRAACIASALFPERIIGLVSVDGYNIQNIASAHQPAPPEEEATYWYQYYFQTQRGRYGLEQNRDEL----------------CQLLWRNWSPTWEQAETAFLR-----TSPSLHNPDFVDVVIHSYRHR---------YGAADGDPHYQELEDRlpQITVPTIVLDCAADG-VGVSYAEADSAKFEgyEFRTLDggHNAPQESPNDFARAALDVLD---------------------------------------------------------    
 953 UniRef50_D1C835        Alpha/beta hydrolase fold p...   123     1e-26  1  14.5%  89:419    25:305     ----------------------------------------------------------------------------------------AADEFAARGQWVIADGLRLFLLDEGT--GFPVLLLHGVPTSSFLWRDVVPV-VARTNRALAPDLPGFGYSDKPRGRS-----YAVPAQADAVAALLDRLGIDRCALVGHDLGALVAADLIAREPERYTHLVVTNTSL--------RVTGWHGASPLTLLRLPLVGECAMALARR------------------WMLGLAMRPYLART-----------------SRLTPEALDHYWWPFAHGFKSVLLSMVRERIGSPDDFARWRAALAAYPGAALIVWGLRDPTFGRGELDDVRSLLPRATvaFPNANHFIPEDRPLALGRLIALFVAGEP------------------------------------------------------    
 954 UniRef50_Q47P85        Putative hydrolase n=2 Tax=...   123     1e-26  1  10.3%  90:418     9:294     -----------------------------------------------------------------------------------------PPRPTRVLRVRSADGTMLHAEVHGPEDAPTVVFSHGWTCSTLVWLPILRVL-GDSIRAVLYDQRGHGRSETPR-----RSGYSTTALADDLCAVLHATVPrrVAVVGHSMGGMTLMAAADRPEFRSRVAAALLASTGSHELVPGA---------HVVGGPSLLRSAFHRMLLTAPLPLGPITPLTRAGLRYVTMSPGSSPEMVDLCVRMVHACRALPR--------------------------------ARWGAVLRDLDLSAAVPRLDIPTRVLVGTADRLTPPFHARRLAEALPACelTLLPggHMTPLEAPEQVSAELQELLRSH-------------------------------------------------------    
 955 UniRef50_B2IZH4        Alpha/beta hydrolase fold p...   176     2e-42  1  11.5% 101:421     5:267     ----------------------------------------------------------------------------------------------------QVNGIDLFYDIKGT--GEPLLLIAGFLCDHAYWSLIMPSLISQ-YQVIRLDNRGMGRSSAPETP------YSLKQMANDVAALLNHLAIDKVHLVGHSMGGQIAQELVLAHPEKVQSLMLLSSLAKGDGLFNSIIETWGELCANV-----------------------DLKLYEKVVLPWIFTDSFY---------AIPGTIEGLIEFAIRYPFPPAT------------------HSLHHHSQAMLDFDTTDRLQKIHCPTLVLVGKQDILTPLKFSQQLAQGIPNAELVVlgGGHGFLIESPDTVISAMLNFLRKLKPA----------------------------------------------------    
 956 UniRef50_B5ITW3        Hydrolase, alpha/beta fold ...   163     2e-38  1  13.0%  98:419     2:269     -------------------------------------------------------------------------------------------------PFIKVDGIKIHFYDSgeENLPALLFLHGSPGQISNWKHILPCF-EGSYRVVAVDLRGYGKSDKPLNVA-------LEDYIRDIDAIRSELGLENIVLIGHSFGAMIAIEYAARRH--VNGVVLIGPVAYLKTDAIDKIIMHLPPAIWKPILFK----------------------SNLLTRRMYRKMFFSPSTPDEVFLEFMKDNKDYIESLPPQSF------RYLIHLAGYNAKPSA--------------------ERVKVPTLIIVGEDDVVTPPEHAKLIHKWVENSKLVIIpaGHLVLYEKPGEICRLIKEFIKGAD------------------------------------------------------    
 957 UniRef50_A4A1W1        Alpha/beta hydrolase fold p...   159     2e-37  1  11.9%  99:417     5:266     --------------------------------------------------------------------------------------------------RIPIGEVTLNVVIRG--EGEPVLLAHGFPLDHSMWEAQINVLAAEGYQVIAPDLRGFGESDPATDKT------TMAQFADDLSRLLAKLNvqPVTFCGLSMGGYIAWQFFQRHRTRLARLILCDT-----------------------------------RANDDDEKVARGRLVMA----------------AEVERFGVAKVPSTMVPKLLSPISFEKNAAVVQSLEAMILRQDPAGVAAAQRGMAERENVVHLLPTIDVPTLVICGEADSITPPDVMQEIAAAIADSQYVEigAGHMAPMERSVEVNTAILNFLKT--------------------------------------------------------    
 958 UniRef50_B7R992        Hydrolase, alpha/beta fold ...   158     3e-37  1  11.4% 100:416     4:264     ---------------------------------------------------------------------------------------------------ITVNNVSLYYEIHG--NGSPLVLIEGLGCSKWMWFKQIDEL-KKHFKVIVFDLRGVGDSEKP------DMEYSIKLLADDTAALVAELGFKKVHVLGVSMGGYIAQELALEYPDLVDRLILCSTHYGGPNIVPIPLSTL--------------------------------------------NIILNGAGLRNALENLRIAMSLNFSDEYLSTHKDEFEQIVKWKFEKPQPFYA----YRRQLYAALTFDEEARIHLIKHPTLIMAGKDDKVVPYENALLLHSKIENSEIELfnAGHMFFIEKAEEVNRKIIEFLT---------------------------------------------------------    
 959 UniRef50_A2T3U9        Esterase n=1 Tax=uncultured...   157     6e-37  1  10.4%  98:415     2:264     -------------------------------------------------------------------------------------------------PIVSANGIEIYYETAGSPDAPPLLLIQGLSGYTEGWLMQVPALKEDFY-VIYFDNRGAGKSTQ------SEPGYMMVDMADDTAALLDTLEIPQAYVLGVSMGGMIALNLAMQHPQKVNKLALGCTTAGGASAVW-----------------------------ADEKVSAALITPSSGDLRQDFYDSAWFLLAPDTIENNSRLVEQLAENAGNNPQTP----------------TGFMGQLQAIS----THDVAGALPELSMPTLVMHGDLDLLIPLQNGRFLAEQIPHAEFkyPNTGHLFFVEQAVPVNDDLREFF----------------------------------------------------------    
 960 UniRef50_B7K9W4        Alpha/beta hydrolase fold p...   156     2e-36  1  10.1%  99:416     9:280     --------------------------------------------------------------------------------------------------FVDIEGLSIRYYQAGT-KGLPLVLLHGTGDSALDWSWVLPMLAS-NYCVYVPDLPGHGESAKP------IRDYSLSFFTEFVIKFLDALKLTTVVMVGNSLGGLISLQVALTDQKRVAALVLADSTGLGQWANPLLCGLTLPIYGESAV----------------------------------------IAGKTPLGAKLRARSRSILLFAHPERIPQEWYLEQEHIAQIPGFMEADLSALRTQLNLFGQriLLDMLPQLSMPILLVWGIKDLIVPKTQAEIALRYLKQGQIaiPDCGHVCPLEQPDAFVSALDKFLT---------------------------------------------------------    
 961 UniRef50_Q9AJM9        BioH n=1 Tax=Kurthia sp. 53...   155     4e-36  1  12.5%  98:418     2:267     -------------------------------------------------------------------------------------------------PFVNHDNESLYYEVHGQGD--PLLLIMGLGYNSLSWHRTVPTLAKR-FKVIVFDNRGVGKSSKPEQP------YSIEMMAEDARAVLDAVSVDSAHVYGISMGGMIAQRLAITYPERVRSLVLGCTTAGGTTHIQPSPEISTLM-------------------------VSRASLTGSP---------RDNAWLAAPIVYSQAFIEK------HPELIQEDIQKRIEIITPPSA-------YLSQLQACLTHDTSNELDKINIPTLIIHGDADNLVPYENGKMLAERIQGSQFHTVScgHIYLTEAAKEANDKVIQFLAHL-------------------------------------------------------    
 962 UniRef50_Q2SQ02        Predicted Hydrolase or acyl...   153     2e-35  1  11.3%  56:410     9:307     -------------------------------------------------------FFRAGFLVAALTLLSACSEIKYGLYELAMDFERSQAGVTPKS--LETQGRHFAYLqqAAPDAETILLLHGFGASKENWLRFIRHL-PARYHIVAVDLLGHGDSSKDPSIP-----YDIDDQVGYVRAFTEAAGLTRFHLMGNSMGGAISSMYAAEYPDTVASLVLIDPAGVYEFSSELETRLQKGENP---------------LIVRKPEDFG--VLMDFAMEDKPFIPWPITSVLTEKAIANTAIYDHI---------------------------------FAELVRDRHDYDFKQVLQNVKCPTLVLWGKEDRVINYRNAEVFTRLITNSRKMIIDggHAPMIEAPEKTAEI---------------------------------------------------------------    
 963 UniRef50_C0ZJ24        Pimeloyl-CoA synthesis prot...   151     7e-35  1   9.7%  99:416    11:270     --------------------------------------------------------------------------------------------------FLEANGARIYYEVAG--EGEPLLLIHGFNLDTRLWDAQLQAFA-QTYKVVRFDIRGFGKT------LATDVPYTLY---DDVKAVLQGLGIEKAHVAGLSFGGMVAQEFALAYPQMVNSLILVASGLFGHPRSEQRLHDV--------------------------ERFNQVCQRGTTEEALEMTTQMWFDGPGQPVNEQAREARNRFKEINRHAFSLPEFGVGLETLS---------------------PSPIERLEEIKAPTLVIAGGRDYPDFLQIADVLGERITGakVILPDSAHIPPMDQPEVFNKLVLEFLA---------------------------------------------------------    
 964 UniRef50_A5J2A5        Meta-cleavage pathway hydro...   147     6e-34  1  11.7%  95:419     5:279     ----------------------------------------------------------------------------------------------FESKFIDCDGIRTHYIEMGEGD--PLVLVHGGGAGAdnFADNFPIFA-RHMRVIAYDMVGFGQTDAPD---PAGFAYTQAARTDHLISFIKALGLSKICLIGNSMGGTTACGAALKAPELIDRLVLMGAAVNISPDDMVANRDDLAAVMSY--------------------------------------------------DGSEEGMRKIIAALTHSYQPTDDIVHYRHEASLRPTTTAAYKATMGWAKQNGLYYSPEQLASLTMPVLVLGGKNDVMVPVRKVIDQILAIPQAqhVFPNCGHWVMIEYPEEFCTQTLHFFGKLD------------------------------------------------------    
 965 UniRef50_B5JUK2        Putative uncharacterized pr...   143     1e-32  1  10.6%  95:415    17:290     ----------------------------------------------------------------------------------------------EHSRYIRVDGIDVHYRDEGRRdaPILLMIHGLLASLHTWDGWVEHLADQ-FRLIRMDVPGFGLT-----GPHPNLDHTPEYAARFCREFIEAVGITqfYVAGSSLGGFVAWNYAVRFPESIERMVLLDPIGYPQPLPQVLKLVALPGVRH--------------------------------AARYITPKFMIDRNLREI-------------YGQTHRLTEERRQLYYELLKRQGNRKNMVAMFQTLTDYSTHPTLKDNIRHLKVPTLLMWGELDRWVPVSLVDHWQSDVDGLKvvYPGVGHIPMEEIPYRSAKDARQFL----------------------------------------------------------    
 966 UniRef50_Q1YKE5        Putative hydrolase or acylt...   142     2e-32  1   9.8%  99:423     3:266     --------------------------------------------------------------------------------------------------FERVNGVVLHHKVEGNAAGPTLVFANSLGTDFRIWDAMVADLATD-YRIVRYDKRGHGLSEATPSP------YAMEDHVADLKALIDHLGLVEPLVVGLSIGGMIAQGLASQHPGVAKGLVLMDTAHRIGSDELWNQRITAIE-------------------------ADGIAAISDQILQRWFTPDFRAPGRPDFVGYSNMLLR------------------------------TPLAGYLGSCCALRDADYTEATRALTLPVLCIVGDHDGSTPPDLVRSTADLIAGARfiVENAGHLPCIEQPQATTRLVADFAAEVFGGAG--------------------------------------------------    
 967 UniRef50_A6V9P1        Esterase V n=1 Tax=Pseudomo...   141     5e-32  1   8.9%  80:415     1:277     -------------------------------------------------------------------------------MAPSVFANTSKPIPIEQGSITTPNGLRIAYERQRNG-GHPVLLIPGTMCDRGIWRPVQEAF--QRFDTILLDNRDSGQSSLSPS------NYTLRDMAEDALALLDELRIERVHLVGHSMGGQIAQELALMAPARVSRMLLANTWARTDD--------------------------------------------------NMNAMFDSWEVLRNYQSDEAFAKSMTIYGNGSSTLSRRTVDELLEASAKILAPQEPAAFLRNVEAD-RKADTLSRLGEIKARTLVLWSEEDRCLLPPHAKQLEKGIPGAkvRIRNCGHNSMGERSNVFMKIAEDFL----------------------------------------------------------    
 968 UniRef50_B2JKJ7        Alpha/beta hydrolase fold p...   138     5e-31  1   9.5% 100:416     3:259     ---------------------------------------------------------------------------------------------------INVNGIDTRYVMSNEGGGPWLTFVHQLGGDLSIWDQLAGYFRDD-FTVLRYDVRGHGQTS------VSDRQFSVADLSQDLRALLNALGVENTHIVGMSMGGMIAQQFALDNPARVATLTLADTHAASPVDGRGTWEQRAAKAREEGVAALSQATMERWLTAGFRDA------------------------------------------------HPEVVEQIREVFEHTSS-----EGYAQACIALRDFDVRSRLKEIALPTLTVAGRHDAGTPPAATQEIANAIRDAQFELLDAAHLAPIEQSQRFTALLESFVQ---------------------------------------------------------    
 969 UniRef50_A4JQ03        Alpha/beta hydrolase fold n...   137     9e-31  1  14.7% 104:415     8:263     -------------------------------------------------------------------------------------------------------DHDLYYADVGS--GQPIILIHDLGSCGLAWTRQIPFLRQAGYRVIVPDLPGHGASSYP------DRTLDVRELAGSLQFLLDYLGLESAALVGISLGSAIALTIALDAPARVGKLVVCNGFSNLVDDDFVRRFEFWKCGFARDDGASRW-------------------------------------------------FEEMWPLMVSAEFAQSEAGEIAYQIGHARAATTDSAQLARLCIGIANYDVRRRLNLVRSETLVLQSEDGSIFPATEGQRLAAMIPGARytLPGGGHHANLDRAGAFNWAVLRFL----------------------------------------------------------    
 970 UniRef50_Q0FYU2        Alpha/beta hydrolase n=1 Ta...   137     1e-30  1  11.4%  80:411     1:280     -------------------------------------------------------------------------------MRTGAGRPPADWPLSEHSRSVDAGGIRWHFQIMG--EGPAIMLIHGTGASTHSFRDLAPILAE-NHQVLMADCPGLGFTDRP------SRSLTLQTMAAMHAELLQKLGVNPAVIVGHSAGAVIAIEMALDGRVAPQHLVSLNGALLPFPG----------------------------LAGRLFPKIAKVVYRNPIVPRFFAYRAKSENAVSGLLDGTGSA------------LTPGGKSLYARLFQDRHHVAGTL----SMMSHWDLDRFSARLDRLETPLLLVTGDRDKAIPVSVSHRVAAQVKNGKVVVLNggHLAHEEAPGLIAELI--------------------------------------------------------------    
 971 UniRef50_B3QPC0        Alpha/beta hydrolase fold n...   136     1e-30  1   9.3%  88:419    11:297     ---------------------------------------------------------------------------------------MASKDRWFIWQFSDKDEAKIRYREYGPADSppLLFVHGYGGMIEHWNDNIPAFSDR-YRIYAMDLIGFGQSTKP------NVRYCLELFAAQIKAFMHLKKLDKIILVGHSMGGAGSVIYAHLNPERVRGLILANPSGLYGDSMDGMAKAFFGLVGSPLIGEMLF------------AAFANPVGVSQSLTPTYYNQKKVDLNLINQFSRPLPDRGAMFSYLSPSKRPHDFMLEGIKPCNYKGD------------------------------AWLMWGAEDTALPPhiIPEFQELLPQaaYIIPKAGHCIHHDAHETFNARLAHILGRLE------------------------------------------------------    
 972 UniRef50_B8EJC1        Alpha/beta hydrolase fold p...   135     3e-30  1  13.3%  53:412    70:367     ----------------------------------------------------IYPVKALIGVLADSDVPDAEIDAFVAGYVTPAAEEGSDEDAGPQYEFAETPAGSLRYAKRGSGP-ETIVLVHGFGGDLDNWLFNIDALAE-HATVYALDLPGHGQSTK------SLADASLSGMSKALADFLDKLGVSAAHLVGHSMGGAICLRTAIDRPDLVKSLTLISSAGIGEDINID-----------------------------------------------YIERFAGASSRRELKPALEQLFE------DPNLVSRQLIDDLLKYKRLDGVSEALNALAKSMFPGGKQTslADEAARTKRPVTVIWGASDKIIPASHAATLPAGW-RAE-VINGAGHMVQMEKAAAVNALIR-------------------------------------------------------------    
 973 UniRef50_B8LT59        Epoxide hydrolase, putative...   134     6e-30  1  12.1% 104:416    15:281     -------------------------------------------------------------------------------------------------------DVTIHGIHGGS--GPALLLLHGFPQTHHIWHCVADELT-KSYYVVAIDLRGYGRSSKPDGG-EGHVQYGKKAMAQDAVRVMDELLKRrfYICAHDRGARVTHRLCVDYPHLVKKVILLDI--CPTLAMYTQTTQEFAQSYFHWFFLIQQSPLPEISILTNPQVWVESFMGG--------------------------------RYAGLAPFEEECLKEYKANLADYETVHAMCEDYRA-SASVDLREAEEDVAEGKC-----------FDPLEEWRKVSSQSVDGKAV--DSGHYIPEERPEEVIANVLEFFK---------------------------------------------------------    
 974 UniRef50_D0E8E2        Polyketide synthase n=2 Tax...   134     6e-30  1  14.6%  82:402 1916:2199     ---------------------------------------------------------------------------------PQVSTTPSTEQPIMEEKFLEFGGNQICLCSWGSPEHPVVLCIHGILEQGLAWQEVALPLAAQGYRVVAPDLFGHGRSSHLEMVTS----YSSLTFLAQIDRVIQELPDQPLLLVGHSMGAMLATAIASVRPKKIKELILVELPLPAEESKKESAVNQLTTCLDY--------------------------LSSTPQHPIFPDVATAASRLRQAIPSLSEEFSYILAQRITQPNQGGVRWSWDAIIRTR-------SILGLNNLPGGRSQYLEMLKSIQVPTTLVYGDSSKLNRPEDLQQQKMTMTQAKRVFLSGGHNLHID-----------------------------------------------------------------------    
 975 UniRef50_C0N3R7        Hydrolase, alpha/beta fold ...   134     6e-30  1  11.2% 106:418     2:254     ---------------------------------------------------------------------------------------------------------KLHYQQLG--EGQPLLIIHGLFGSSDNWRSMARYFS-KFFQVISLDLRNHGQS-------PHSEKQDFTLMAEDVRALCDSLGISKAHVLGHSLGGKVAMQFAAHYPQSVDKLVVVDISPRQYFSQHTPMMDTMMALDMDQYASRSEIDEALSASISD-KTVRQFLLMNLRTDESGFSWRINLPALKQNYQQLMAPVCTTAVLDMPSLFVYGALSDYVN-------------------------------------------TQDRTL-------IQQHFTQAEFviEKAGHWVHAEKPQQFKQIVEEFLRDA-------------------------------------------------------    
 976 UniRef50_C1XPW6        Predicted hydrolase or acyl...   134     9e-30  1  10.7%  98:416     2:267     -------------------------------------------------------------------------------------------------PRIAINAAELYYQIDgdPDQPVLVLANGIFQRVEAWEPLMPHL--PGFRVLRYDMRGQGRSSIPPG------AYTPELHADDLEALLEALNIERYHLLGLSNGGIVAQVHAARQPAGLHKLILLCTTSRIDPLIRAKVESW------------------------------RLGLEWGSTEGRLRVALPWIWGRAYLEAHPEVAGAASLEQMKLAAPTVEAQQNLMAGFFTLG-------------------DLRPQLRSVTAPTLVLSGQEDLLFPPLYAQEIAQAIPGAKHRVLptGHAAPIEVPALLAREIREFLE---------------------------------------------------------    
 977 UniRef50_A4ADA0        Carboxyl esterase, a/b hydr...   133     1e-29  1  11.5% 100:413     4:287     ---------------------------------------------------------------------------------------------------VSVNTINLEYESHGNPENPTVLLIMGLGTQLTGWPpfCDYLVDHGYHVLRFDNRDIGLSSRLDHErpAPAPYTLEDMAADAIGLLDALKIDKAHVVGASMGGMIAQLIAVHYPERTLSLTSIMSTTGHRS-----------------------------LPRADRAATKALLLKP------------DNPDDPASIIERNVRVRKALQSRTHPKDDSEIRQTATEAVNRGGYY--PEGVARQLAAIIVAKDRRKLLARVDAPSLVIHGEEDTLVKVECGIDTAKHLNDSELRILPG---MAHDLPTPLLQEIAD------------------------------------------------------------    
 978 UniRef50_B8MCZ0        Epoxide hydrolase, putative...   133     1e-29  1  11.7% 105:413    32:419     --------------------------------------------------------------------------------------------------------LSYHILEAGAPTDPLIILLHGFPELAFSWRKVILPLarRGYHVIAPDQRGFGRTTGWDSRHYEQVdfTTTMYVRDTIALVNALGYSKvcIVGHDAGAVTAAACATIRPDVFKSVVLLTHPFSGMPRLPFNTVDIQGSSSFPPASAGIAApaGPTTAGGLREFLRGYFHVKSASSKGnmPYYYIMPLEATMPQAIERLMTDEPHDASKDWLPDEDLEVYVNEYART-TFQGGLNWYRAQTADGdkPELRHDlkRITIPCAFIGGEKDWGTypGAIEKmkEVCDDFrrMVEGAGHWIPQEKPDEVVQAILE------------------------------------------------------------    
 979 UniRef50_Q9FFW9        Genomic DNA, chromosome 5, ...   133     2e-29  1  11.4% 114:419    87:360     -----------------------------------------------------------------------------------------------------------------TPASQTVLLVHGFGASIPHWRRNINALS-KNHTVYAIDLLGFGASDKPPG-----FSYTMESWAELILNFLEEVVQKPTILIGNSVGSLACVIAASerGDLVKGLVLLNCAGGMNNKAVFDDW-----------------------------RIKLLMPLLLLIDFLLKQRGIASALFNRVKDRENLKNILTNVYGNKDNVD----DTLVEIIAGPANTEGALDAFVSILTGPPGPNPIKLIPEITKPVLVLWGDQDGLTPLdyFTSLPDQLPNFNLYVLQggHCPQDDRPDLVHERLLPWLAQLS------------------------------------------------------    
 980 UniRef50_Q39NS4        Alpha/beta hydrolase n=8 Ta...   131     4e-29  1   9.8% 103:418    18:298     ------------------------------------------------------------------------------------------------------GGVRLCYRIYGPVHGEPLLLIAGMTLQLTFWPplIDALIQCGYRVIVFDNRDVGRSSRIPRPGPSvdGYDLEDMAQDTIGLLDSLQLKRAHVVGMSMGGMIGQVLAARYPQRVLSLTSIISTTGARKVGQPAMSSMLMLT-------------------------------------------APPPRTREAAVEQYSRIMAHIGTQAYPADDAARRAYAAHAWDRGQQARDHESTTRQIGAIIKSGDRTDEIRRIQAPTLVVHGDRDLLVAPSGGIATAAAIPGADMVTVRggH----DIPDGVVPLLTSLIDGH-------------------------------------------------------    
 981 UniRef50_A0QDY7        2-hydroxy-6-ketonona-2,4-di...   130     1e-28  1  14.2% 100:417    41:308     ---------------------------------------------------------------------------------------------------IAVNgvRTRYAEAGSPDKPHAILLHGTGGHWETFAPNLEALSE-HLHCVAIDMVGNGFSDKP------DYDYEIAVYVEHVLGVMDHFGMTSANFVAMSLGAFVASAVAVGHPDRVDKVILMSPAGREASASNMARIRAERTKAVNEPTWGSLHAVFAHLIADESNRL------------------------------------------------PDLIGLRQAIYRREDTRRTIDRLLILQDEKVRNRNLIPdwRGITAPVMIVASGKDHGVYQDTARTIADLIPNSEveMPSVRHWPHFEDPESFNAAAVEFLTR--------------------------------------------------------    
 982 UniRef50_Q7UF99        Haloalkane dehalogenase n=2...   130     1e-28  1  11.7%  93:418    26:321     --------------------------------------------------------------------------------------------FPHPSSELSIDGHTLRYIDtgSSDgpTFLCVHGNPTWSFYYRRIIERYGKQQ-RVIAVDHIGCGRSDKP---SEDEFPYTMAAHRDNLIRLVDELDLKNVILIAHDWGGAIGLSAMHARRDRLAGIGLLNTAAFPPPYMPQRI-------------------------------------------AACRMPVLGTPAVRGLNLFARAAVTMAM---SRTKMKPDVAAGLLAPYDNWKNRVAIDRFVRDIPLNDSHpqLESDLPDlsLPISLIWGMKDWCFRPECLRRFQSVWPDAEVTELatGHYVIEDSPEETLAAIDSLLARV-------------------------------------------------------    
 983 UniRef50_B6QDZ2        Epoxide hydrolase, putative...   130     1e-28  1  12.2%  80:416     4:415     -------------------------------------------------------------------------------LATASEPAPLPLPPGIISRQIDlgHLSYHILEAGAPTDPLIVLLHGFPELAFSWRKVILPLarRSYHVIAPDQRGFGRTTGGDSRPYEWVdfATTMYVRDVIALVNALGYSKvcIVGHDASAVTAAACAVIRPDVFKSVVILTHPFDGVPRLPFNTIDTQGSSSFPPvyYSTHDANGDMAGPTTAGGLRGFLRGYFHVKSGSWKGNSPYPLIrpLEATMPQVIEKLmqDASKDWLSDADLEVYVNEYART-TFQGALNWYrqTADGGKKPELKHDlkRISIPCAFIGGDKDWGTypGAIEKMaeVCDDFRMlvEGAGHWIPQEKPDEVVRAILEFID---------------------------------------------------------    
 984 UniRef50_P23133        2-hydroxy-6-oxo-2,4-heptadi...   129     2e-28  1   9.0%  95:417     7:274     ----------------------------------------------------------------------------------------------EIGRMVLAGGIETNLHDVGAGN--PVVLVHGSgtAWANWRTVMPELS-RHRRVIAPDMVGFGFTQRPHGI-----HYGVESWVAHLAGILDALELDRVDLVGNSFGGALSLAFAIRFPHRVRRLVLMGAVGVSFE--------------------LTDGLDAVWGYEPSVPNMRKVMDYFAYDRSLVSDELAELRYKASTRPGFQEAFASMFPAPRQRWVDA-------------------------------LASSDQDIRDIRHETLILHGRDDRVIPLETSLRLNQLIEPSQLHVFgcGHWVQIEQNRGFIRLVNDFLAA--------------------------------------------------------    
 985 UniRef50_Q11G88        Alpha/beta hydrolase fold n...   129     2e-28  1  13.5%  97:414     3:277     ------------------------------------------------------------------------------------------------TRIVKANDIEIATEAFGDQSHPPVVLIMGGMASMLWweEFCRRLAGRGRFVIRYDQRDTGLSTK---YPPGQPRYGYGDAVDDVFRVLDGYGISAAHIVGFSLGGMAGQGAALKKPERVLSLTAISTSPLGTDTSHLPAAGEAWLEHMKLDVDWSDRAEAVRYLVD----------------------------------------DARLTAGTAHPFDEARTRAFIERDFDRSG--GYLSATNHSILFEIGEPWRGRLHEMKVPLLVIHGTADPVFPVEHGETLAAAVRGARLLKLdgGHELHPGHWDGIVGAIAEH-----------------------------------------------------------    
 986 UniRef50_C1N8H0        Predicted protein n=1 Tax=M...   128     5e-28  1  11.1%  94:417    86:378     ---------------------------------------------------------------------------------------------PVTSHEWRWEGHKIRYTAAGPddAPAVILVHGFGGNADHWRQNTPVI-GRKCRVYAIDLLGYGYSDKPDPLSKPRNsyNFENWSRQLAAfaRSVVGGPAFVFCNSVGGVAGLQLAVDAPELVRGVVLINIS------------------------------------------LRGLHVTKQPALARPFIAALQSTLRETDIGRKFFGNTVRNILKEAYGNPDRVTDELVDCILKPGLQPGAAEVFLDFISYSGGPLPEELLPKitVPVRILWGQADPWEVVTEGRAYGKfaVDRFIELPGVGHCPMDEAPELVNPLLMEFVED--------------------------------------------------------    
 987 UniRef50_D2RYK1        Alpha/beta hydrolase fold p...   128     5e-28  1   7.2% 104:421     8:275     -------------------------------------------------------------------------------------------------------DGTLWYETRG--DGQPLVFIHGGWMNGQSWQPQVDRF-DDDYEVVTFDVRGHGQTGATEA-----DQYSVELFTDDLEALLSHLDLEAPILCGLSLGSMVVQEFMDRHPDRAAGAILGGAVRSMPPLNMPAELKP--------------------FMSPLPALTTSLSLTGSKTTFRSMLGSIRATTGERWLSVNPEIQSQAIDA--------------VDEIGRDEFRKVFDALYRYEPSN---------LSHVETPTLVVHGEQEAPLVKRQGRQLVSEVGGarLELSDSGHLVNLDRPNAFNSASETFLETASAA----------------------------------------------------    
 988 UniRef50_C3JIL0        Hydrolase n=4 Tax=Rhodococc...   128     5e-28  1  10.2%  50:413    10:339     -------------------------------------------------LRVLAGAVGVGALVAAVAGIRHRRSMRGLTLSSDVEPNatPTLKADISELTTDDGAIINVRAYGPVDGDPIVLSHGWTCSTEFWYPQVNALAGE-YRVITYDQRGHGRSTVGNLPLGPDV------LADDLSAVLAATVRekAVIVGHSMGGMSIIAWAGRHPDEvaSAVMLASTASERLIAETTVIPLPNRFPRVPVPVGRAILSSPVPFVSSGAMTKAIQYVSMSPNATKAEIQFCENIVRECKPRIRGGWGAALSTL----------------------------------------DIHEGLDNLEVPTTVLVGSLDRLTPPVHARKLAQVLDDarLIVLPggHMSSIENIDEFDKEIVR------------------------------------------------------------    
 989 UniRef50_C6WFU0        Alpha/beta hydrolase fold p...   127     7e-28  1   8.8% 101:418     6:266     ----------------------------------------------------------------------------------------------------SADDVQLHYQVSGS--GEPLLLLSGQANNLHWWDRVRPDF-EREFTTVVLDHRGTGLSGKPEG------GYATRTFAEDALSVLDDLGVDRCHVYGTSMGGRVAQWLAADHPERVRGLVLGCTSPGPP----------------HGVERSAEVRRSLAQPDRLKARRALLELMYTPAWLERHDEPFTTHGG---------------------------------PFTTLGDPDMPPHARKGHLLASGNHNSWDALPSITAPTLVVHGTDDEFNPTANAPLLAKRIPDTRLHLVEGahAYFEEFHHETGPLVADFLDAH-------------------------------------------------------    
 990 UniRef50_A2QN70        Catalytic activity: epoxide...   127     1e-27  1  10.8%  94:418    17:403     ---------------------------------------------------------------------------------------------ITSRFIPTTPSLTTHILESNAPtrKLILLLHGFPELAYSWRHVLPQlaGADYHIVAPDGRGFGRTTGWERDAPNYTDSNLTtlVRDVVSLVHALGYTTvcVVGHDAGAVTAAFCAVARPDMFRSVVLLSHPFPGVPSPVIplKNLGLKHYKWYYSTEIASPEMENPAQGLHDFLRGYFHlmGAISSWTGEQLARMPgmPETVEEMMRQADAQGVARSKEWLSDEELSVYVGEFARTGF-QGALNWYRVRTTPGRQYTWdfAGRRIEIPCAFCSGESDWGVypGALENMRNgcGDLREviPGVGHWAPQEKPEEVVEVILGVVRGL-------------------------------------------------------    
 991 UniRef50_A0PM12        Lipase/esterase LipG1 n=26 ...   127     1e-27  1  10.8%  96:411    17:306     -----------------------------------------------------------------------------------------------TSERQPAGDLKLYYEDMGDINAPPVLLIMGLGAQLLLWrgFCQKPVDQGLRVIRYDNRDVGLSTKMEQPSARQppYTLEDMADDAAALLDHLDIERAHIVGASMGGMIAQIFAARFAERTNSLAVIFSSNNRPFLPPPAPRALLA------VLNGPPPGSPREVIIDNVVRVTRITGSPGYQ-------------------------------PTEEWIRADAAENYDRSYYPWGIP-------RQFSAVLGSGSLLGYNRRTVAPTVVIHGRADKLMRPFGGRAVAKAINGARMVEFDggHDLPRQLWDQVIGVL--------------------------------------------------------------    
 992 UniRef50_B1MEH5        Probable hydrolase, alpha/b...   126     1e-27  1  11.8%  96:433     2:296     -----------------------------------------------------------------------------------------------TSARFTFRGDTLAYSRTGT--GPPILFLHNGGSAKEIWTRQVEVLRQR-YEVICLDHLGYGESDMPE------RGYTIDQHVERLSAFIEHLGHERISVVGNCMGSAMALLLADRRPELFDSLVLINPLSKNTAR------RGVLGWLLPLVSRFPTLSMAISRRVRIPQALTNIVITVQFGPRNW--------------LRGIRAPRPGTAQAGKGWSVRGRLAALAEMFCDQTSLGAV-------DEIRPGPDFP--------PFAVIWGDSNLGLSPRAGRALNRTlpDREEFLTRCGHLPMMESPEEVTAIITEFIARPPARRLGDLATSPGSA----------------------------------------    
 993 UniRef50_C8SG21        Flavin reductase domain pro...   126     2e-27  1  10.9% 114:444    23:296     -----------------------------------------------------------------------------------------------------------------AGSGAPVLLIHGVGMNATIWHDQIASM-QDNHDLIAIDMLGHGGSPLPP------EDAGLTDFADQALRLIDHLGLASVSIVGHSMGALVALEIALHAPLRVRSLVCLNAVF------------------------------------RRPATLAQAVRERAAVLGSHGDASAIAATLARWFGDPLPESLAGASATARAALEGVDPEGYARTYR---------------LFARADAEHADTLARLAAPALFMTGSQDSNSTPAMSAAMARLAPHGqlVLSGERHMMAIASPERVTRHINAFLDTGQAAKPQADGAFDSSEFRRALGSFLTG-----------------------------    
 994 UniRef50_A4X5W3        Alpha/beta hydrolase fold n...   126     2e-27  1  11.4% 100:415    70:334     ---------------------------------------------------------------------------------------------------VPVDGGTLRYTMTGS--GSPLVLLHGWPQTSWSWQPVIPALAGQ-HTVITLDLPGLGGSD------PTTAGYDKATTARLVRQAVNNLGYTQVALLGHDLGAMVAFNYARDYPTEVTRIGVVESPLPGFG-----LEDLYGISWHFLFNASPAPIPETIMDNDDVSTYLGMLFEKAQRPELIPQNV------------------------------------YFRAYGNPDKRSAGYEYYRAFVTDAADYQEHAESKRLEIPVMAMGAEH--VFGSLVAESFQQVADDVRTVlpDSGHWIPEENPDFFGDCALLFF----------------------------------------------------------    
 995 UniRef50_Q6MRP5        Hydrolase, alpha/beta fold ...   126     2e-27  1  11.8% 103:415    15:286     ------------------------------------------------------------------------------------------------------DGTPIYYEVRG--EGEPLVLVYGIACLLNHWHHQIEYFSKK-YQVISFDLRGHQKS----CPVADVSQLTVEALSKDIIGLMAHLNIRKAHFAGHSFGAPLLLDLYEKKSELFLSMVFINGFAKNPIKGMFGL-----------------------------DVVEPFFYFVKSQYESQPDVWNTLWKLA-VDNPMSMYIAALAGGFNLKVTHFKDIEVYARGVARMN-----LEVFLRLFEELMKYDGEPVLEKIEVPVLIISGEKDMVTPIRFQYHFKEKIKHSEFVLVpgSHCTQLDFPDYTNLKIEKFL----------------------------------------------------------    
 996 UniRef50_Q0FZG0        Putative carboxylesterase n...   125     2e-27  1  10.2%  98:419     9:280     -------------------------------------------------------------------------------------------------RIVQIGDTRFAIDDSGVpqGAPLVFLHGAVQTRQVWESQWRAFSGAR-RVVVPDLRGHGETT------GSFEDLSIETHARDVVALLDRLEIDRALVCGVFLGGMVALAMAAKAPERITGLILADTPLALSMTGPVRALIEWLG-------------------------------------PQRILWPFFRVLGRRGTARLGLALARLM--LSRKWVGPMGGRHFVDGFSQMAPAAIVATYAGIVAADPITVEMPDK------PCLLILGRHETKTVVAQASEIASRLGQAEIVLVDGGHVPNIDAPYEFNRAVHQFLEKIR------------------------------------------------------    
 997 UniRef50_A4AJ39        Putative hydrolase n=1 Tax=...   125     3e-27  1  13.9%  96:427    10:282     -----------------------------------------------------------------------------------------------ATQYLAVPGGSIAFEERGT--GPLVLLVPGMGDLRSTYRFLAPALAAAGYRVVSTDLRGHGDSDA------GFLSYGDIATASDISALLRHLD-APAIVVGNSMGAGAAVISAAETPELVAGLVLVGPFVREPASANP------------LTKLFMRVLMARPWAAGVWKAYLPKLYAGAKPSDFTVYR-----------------------------------DAVAAAIKRPGYARAFSETTRT-----DHVRAGESLTSISTPTLVVMGDKDPDFPdaEADWISSALGGTTVMIEDAGHYPQSQQPEQTATAILDFLSAAGHTATGHRA----------------------------------------------    
 998 UniRef50_B8EST9        Alpha/beta hydrolase fold p...   125     4e-27  1  11.1%  93:424     5:278     --------------------------------------------------------------------------------------------RMYESKFIKTGAFTTHYFEAGSGD--PVLLIHGGGAGADSWGNWapMFAER-MRVIAMDMVGFGRSDAPD---PSLFSYTQDARNDQAIAFIEALGCESIHIVGNSMGGATALGVAMKRPDLVRSIVLMGSGG--------------------LNHELSAALAPVVNYDDTFEGMRKIVAVLANPDLEMPEELLRYR---------------------------------YELSIDPARKAAYKATMGWVKQRGGLHYEEREVAAVKTRCLVFAGKDDLVIPMKENVRFLELLENStyFLPHCRHWAMMEYPEIFTNVVIYFLQ---PAARP-------------------------------------------------    
 999 UniRef50_C0VUT9        Hydrolase or acyltransferas...   125     5e-27  1  10.3%  85:423    27:328     ------------------------------------------------------------------------------------DTERPGLVGVTHKGTIDRDGFQLVYKEIGPADAdtVVFIHGFTLAADSYFMQTDYLRDHYPNvlLMDLRGHGET-----GEIPADKLTIEACGDDILALVRERSpsKIIFVGHSMGGLIALNAIKKHPENVVGLVLIASSI-----------ESLSSQGLPQVLASPLADKAQEAVELAP---------------------------GDAQKIKDAFATVLAPALSATVFKRNGTDYNLIEFHASMIDNTPLETFVGFFDDLQHHDEVDAASSLqiPGYVLVGKKDDVTPESQADRIKELWPQAQEKRlkAGHMLILETPDAVNAAIGDLVKAAQAAPE--------------------------------------------------    
1000 UniRef50_UPI0001AF73E7 hypothetical protein MkanA1...   124     1e-26  1  12.1%  96:417     4:280     -----------------------------------------------------------------------------------------------HRRTAVVDGLVTSYLESGTGD--PVVLLHgfGATAElwEHNIVALAAR-YRVLAPDLLGFGQSAKVVDFVDGRG-----MRIRHVARFCDVVGVDSAHFAGNSMGAIMLLTDATSDAPRL--------PIRTMAIICGGGEIQQNRHFDALQRYDATLPGMRRIVEALFH-----------------------------DARYPADEDYVRRRYQSSIAPGAWEAVAAARFRRPGAGPGSE----PESAPASESSSARRYQCITVPTLVVEGRNDKLLPAGWAAQLAEQVKDGrvVIDDAGHCPQIEQSSAVNELLLDFFSR--------------------------------------------------------    
     consensus/100%                                                                                        .........................................................................................................................................................................................................................................................................................................................................................................................................................................................................................    
     consensus/90%                                                                                                                                                   ................................................h.h...s...t..llhhpGhs.....at..h..h....hphhh.D..GhG.o.............t.....t.h.thhttht.t.h.hhs.shGu.hs..hh....t.h.thh..t.......................................................................................................................................h..Phhhh.u..s.h........h.t.h.t..h....suHh...p....h.t.h..h...............                                                
     consensus/80%                                                                                                                                                                           ....................tshphhh...G..ts.sllhlHGhst....ap..h..lstt.apsls.Dh.GhG.ot...........hphtthstth.thhpthshtthhlhGtshGuhhsh.hsh.hsphhtthhhhs......................................................................................................................................ththPshhlhuttD.hh..t.ht.htt.hsthphhh.tsuHh...-tst.h.thl.tah.......                                                       
     consensus/70%                                                                                                                                                                                 ...........h.htshphthhttG..tu.sllhlHGhstshh.Wct.hshLsttta+lls.DhhGaG.Sshs........tashtthstslhsllctlshppshllGpshGuhluhthAhthPptlptlshhsss.........................................................................................h..t.ht.hht.h.t.t..ts.hthhtth.......th.t.htplphPsLhlhGppD.hhs.thspthtphhsssphhh.ssGHh...-tPpthsthlhtalt....
```

MView 1.51.1, Copyright © 1997-2009 Nigel P. Brown
